# Effects of Simulated Microgravity on the Proteome and Secretome of the Polyextremotolerant Black Fungus *Knufia chersonesos*

**DOI:** 10.3389/fgene.2021.638708

**Published:** 2021-03-18

**Authors:** Donatella Tesei, Abby J. Chiang, Markus Kalkum, Jason E. Stajich, Ganesh Babu Malli Mohan, Katja Sterflinger, Kasthuri Venkateswaran

**Affiliations:** ^1^Department of Biotechnology, University of Natural Resources and Life Sciences, Vienna, Austria; ^2^Biotechnology and Planetary Protection Group, Jet Propulsion Laboratory, California Institute of Technology, Pasadena, CA, United States; ^3^Department of Molecular Imaging and Therapy, Beckman Research Institute of City of Hope, Duarte, CA, United States; ^4^Department of Microbiology and Plant Pathology, Institute of Integrative Genome Biology, University of California, Riverside, Riverside, CA, United States; ^5^Department of Biotechnology, Centre for Research and Infectious Diseases, SASTRA Deemed University, Thanjavur, India; ^6^Institute for Natural Sciences and Technology in the Arts, Academy of Fine Arts Vienna, Vienna, Austria

**Keywords:** microgravity, black fungi, extremophiles, secretomics, proteomics, astrobiology, *Knufia chersonesos* (syn. *K. petricola*)

## Abstract

Black fungi are a group of melanotic microfungi characterized by remarkable polyextremotolerance. Due to a broad ecological plasticity and adaptations at the cellular level, it is predicted that they may survive in a variety of extreme environments, including harsh niches on Earth and Mars, and in outer space. However, the molecular mechanisms aiding survival, especially in space, are yet to be fully elucidated. Based on these premises, the rock-inhabiting black fungus *Knufia chersonesos* (Wt) and its non-melanized mutant (Mut) were exposed to simulated microgravity—one of the prevalent features characterizing space conditions—by growing the cultures in high-aspect-ratio vessels (HARVs). Qualitative and quantitative proteomic analyses were performed on the mycelia and supernatant of culture medium (secretome) to assess alterations in cell physiology in response to low-shear simulated microgravity (LSSMG) and to ultimately evaluate the role of cell-wall melanization in stress survival. Differential expression was observed for proteins involved in carbohydrate and lipid metabolic processes, transport, and ribosome biogenesis and translation via ribosomal translational machinery. However, no evidence of significant activation of stress components or starvation response was detected, except for the scytalone dehydratase, enzyme involved in the synthesis of dihydroxynaphthalene (DNH) melanin, which was found to be upregulated in the secretome of the wild type and downregulated in the mutant. Differences in protein modulation were observed between *K. chersonesos* Wt and Mut, with several proteins being downregulated under LSSMG in the Mut when compared to the Wt. Lastly, no major morphological alterations were observed following exposure to LSSMG. Similarly, the strains’ survivability was not negatively affected. This study is the first to characterize the response to simulated microgravity in black fungi, which might have implications on future astrobiological missions.

## Introduction

Based on their nature as settlers in extreme environments, microbial extremophiles are of great interest to studies aiming to elucidate stress adaptation and survival mechanisms. Successful examples of extremophiles can be found in the fungal domain, alongside bacteria, and archaea, which for a long time were considered to be the sole colonizers of habitats previously considered uninhabitable. Some of these fungal extremophiles show even higher resistance than that of prokaryotes (Shtarkman et al., 2013; Aguilera and González-Toril, 2019). Black fungi in particular represent a group of highly melanized microfungi, whose ability to survive in a variety of extreme environments has in recent decades attracted increasing attention (Selbmann et al., 2005; Sterflinger, 2006; Gorbushina, 2007; Onofri et al., 2007). Desiccation, low nutrient availability, excessive radiation, extreme temperatures, salinity, and pH are some of the multiple sources of stress characterizing the habitats where these organisms have been shown to thrive (Gunde-Cimerman et al., 2000, 2003; Selbmann et al., 2008). The highest diversity of black fungi has especially been observed in rocky environments, ranging from mountain summits to the Atacama Desert and the Antarctic cold regions (Gonçalves et al., 2016; Ametrano et al., 2019). Rock represents a harsh habitat and a quite ancient niche for life, believed to reflect early Earth conditions, and thus considered to be a model for extraterrestrial life (Selbmann et al., 2014).

The discovery of melanized fungi in extreme environments has prompted researchers to investigate microbial physiology at the absolute edges of adaptability, aiming at a deeper understanding of the limits for life (Dadachova and Casadevall, 2008; Tesei et al., 2012; Zakharova et al., 2013). Other studies have focused on testing fungal survival in space conditions through simulations in ground-based facilities or in space missions that enabled the assessment of the habitability of extraterrestrial environments and, hence, the possibility of life beyond Earth (Scalzi et al., 2012; Onofri et al., 2019). Space and outer space conditions are by definition hostile, as they include enhanced irradiation, microgravity, and temperature extremes (Rabbow et al., 2012, 2017; Senatore et al., 2018). Nevertheless, the isolation of melanized fungi from spacecraft and space stations—e.g., the International Space Station (ISS)—has been reported frequently (Checinska et al., 2015; Checinska-Sielaff et al., 2019). In this respect, a number of studies have examined the molecular adaptations of ISS-isolated strains to space conditions, showing alterations in metabolome and proteome (Knox et al., 2016; Blachowicz et al., 2019b; Romsdahl et al., 2019). Investigations of microbial survival in space are therefore relevant also in the context of space missions, to prevent contaminations and to develop strategies to reduce hazard, especially in the case of opportunistic species (Urbaniak et al., 2019).

In black fungi, astrobiological studies have evaluated the potential effects of Mars or ISS conditions on fungal viability and metabolism. One investigation revealed that a rock isolate from Antarctica, *Cryomyces minteri*, could survive simulated Martian atmosphere and pressure, temperature fluctuations between −20 and 20°C, ultraviolet radiation, and vacuum (Onofri et al., 2008). Comparative 2D-PAGE proteomics was carried out for other rock-inhabiting fungi (RIF) (i.e., *Cryomyces antarcticus*, *Knufia perforans*, and *Exophiala jeanselmei*) exposed to these conditions and showed a decrease in protein complexity, followed by recovery of the metabolic activity after 1 week of exposure (Zakharova et al., 2014). Further, survivability of *C*. *antarcticus* in outer space was shown via colony counts following a 1.5-year-long exposure on board the EXPOSE-E facility of the ISS (Onofri et al., 2012). A more recent experiment of *C*. *antarcticus* exposure on rock analogs under space and simulated Martian conditions revealed only slight ultra-structural and molecular damage and pointed out the high stability of DNA within melanized cells (Pacelli et al., 2016). In other investigations, the resistance to acute ionizing radiations was demonstrated in RIF (Pacelli et al., 2017, 2018). Together, these studies have revealed the ability of rock-colonizing black fungi to endure space conditions; however, to date, reports that specifically evaluate the response to microgravity have not been produced.

Microgravity is an important factor influencing microbial life in space environments, a condition in which the gravity level is almost zero but not neutralized. Due to the technological and logistical hurdles linked to studies of microgravity in space, different methods have been developed to simulate microgravity and analyze microbial responses (Herranz et al., 2013). Accordingly, the term low-shear simulated microgravity (LSSMG) is used to describe the environmental condition created by these devices, resembling the low-shear effects of the fluid on the cells (Yamaguchi et al., 2014). Spaceflight and ground-based microgravity analog experiments have suggested that microgravity can affect microbial gene expression, cell morphology, physiology, and metabolism, also triggering increased virulence in pathogenic bacteria and fungi (Altenburg et al., 2008; Taylor, 2015; Sathishkumar et al., 2016; Huang et al., 2018). Although the effects of microgravity on microbes have been studied for several years, only the responses of a few typical prokaryotic and eukaryotic model organisms—e.g., *Escherichia coli*, *Candida albicans*, *Saccharomyces cerevisiae, Penicillium sp., Aspergillus sp.*—have hitherto been investigated (Huang et al., 2018). Hence, studying the reaction of black fungi to microgravity holds potential for the elucidation of the molecular basis of tolerance in extremotolerant and extremophilic fungi, and can also contribute to unearthing the biological uniqueness of these species and their adaptability to space conditions.

In the present study, the qualitative and quantitative proteomic characterization of a black fungus response to LSSMG was carried out for the first time. The rock-associated *Knufia chersonesos* (syn. *Knufia petricola*) was selected as the model organism due to its reported poikilo-tolerance, e.g., the ability to endure xeric conditions, desiccation, high UV-radiation and temperatures (Sterflinger et al., 2012) and to feed on alternative carbon sources like monoaromatic compounds (Nai et al., 2013) and synthetic polyesters (Tesei et al., 2020). *K. chersonesos* aptitude to withstand levels of gaseous ozone up to 11 ppm was also shown (Tesei et al., unpublished). Furthermore, being the only black fungus known to have a melanin-deficient spontaneous mutant (Tesei et al., 2017), *K. chersonesos* allows comparative studies attempting to evaluate the stress-protective role of melanin. Cultures of *K. chersonesos* wild type and mutant were grown in HARVs and analyzed for changes at the proteome—whole-cell proteome (mycelia) and secretome (culture supernatant)—and at the morphological level, with an eye toward the impact of cell-wall melanization on the physiological response to the stress.

## Materials and Methods

### Fungal Strains

The fungal strains used in this study included the non-pathogenic rock-inhabiting fungus *K. chersonesos* MA5789 wild type (Wt) and MA5790 mutant (Mut), both obtained from the ACBR fungal culture collection of the University of Natural Resources and Life Sciences, Vienna, Austria. The Wt, characterized by a highly melanized mycelium, was isolated from red sandstone in Ny London, Svalbard, Norway. The pink mutant, whose pigmentation is due to unmasking of carotenoids resulting from the lack of melanin, originated spontaneously under laboratory conditions (Tesei et al., 2017). *K. chersonesos* (syn. *K. petricola*) is an emerging model organism for analyses aiming at the elucidation of the rock-lifestyle and RIF physiology (Nai et al., 2013). Along with its ascertained thermo-, pH-, UV- and desiccation tolerance (Gorbushina et al., 2008; Sterflinger et al., 2012), the fungus was recently reported to have an aptitude for using synthetic polymers as an alternative carbon source (Tesei et al., 2020). These features and the ability to endure high levels of gaseous ozone (Tesei et al., unpublished), altogether make *K. chersonesos* particularly suited for astrobiology studies. Further, the availability of a mutant strain allows comparative studies attempting to evaluate the role of melanin in stress protection. Fungal cultures were maintained in flasks containing 50 mL of 2% malt extract broth (MEB, pH 5, 2% malt extract, 2% glucose, 1% peptone) at 21°C with shaking at 63 rpm (Innova, Eppendorf). Cultures at exponential phase were used for inoculation of media for all experiments.

### Exposure to Low-Shear Simulated Microgravity (LSSMG)

**FIGURE 1:**
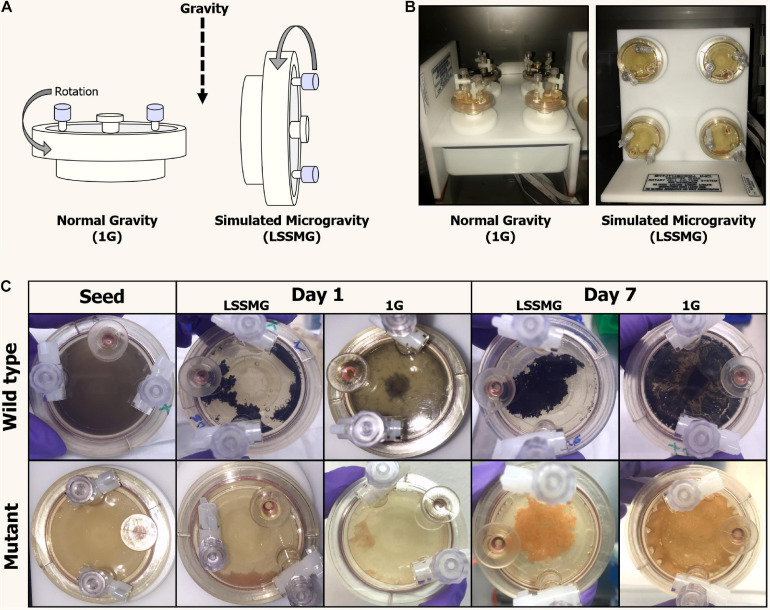
Rotary Cell Culture System (RCCS^TM^, Synthecon) and High Aspect Ratio Vessels (HARVs) used in the LSSMG experiments. **(A)** Schematic diagram of mechanical principle of the different RCCSs (design: Emily Klonicki). In normal gravity, the vessels (HARVs) rotate around a plane parallel to the gravity vector. Microgravity is simulated by rotating the samples around a plane perpendicular to the gravity vector. **(B)** The RCCS used to generate 1G condition (control) on ground (left); the RCCS used to generate LSSMG condition on ground (right); **(C)** Colony and cultural features of *Knufia chersonesos* MA 5789 (wild type, Wt) and MA5790 (mutant, Mut) under LSSMG and 1G on the first and last day of cultivation. The HARVs provide oxygenation via a flat, silicon rubber gas transfer membrane located at the base of the vessel and directly underneath the cultures.

Fungal pellets were obtained from 5-day-old cultures by centrifugation, washed in 1X phosphate-buffered saline (PBS; Thermo Fisher Scientific) and subsequently mildly ribolyzed (3 × 20”, speed 4; MP Biomedicals) to separate cells. As clump-like growth is an inherent characteristic of black fungi, mechanical disruption of cell clusters is a prerequisite for both inoculation of cultures and cell counting (Voigt et al., 2020). Following the cell count (Neubauer), the cell number was adjusted to the initial concentration of 2 × 10^5^ ml^–1^ with 10 mL fresh 2% MEB and used as seed culture. High-aspect-ratio vessels (HARVs; Synthecon Inc) were filled with the cell suspensions (10 mL) and rotated at 30 rpm (initial rotation rate) in the vertical axis to provide LSSMG conditions inside a chamber with 60% humidity and 22°C, for 7 days. Control runs were set up by rotating the bioreactors in the horizontal axis to provide normal gravity condition (1G) (Rosenzweig et al., 2010; Kim and Rhee, 2016). During cultivation, the rotation speed was adjusted to 43 rpm in order to keep the cells pellets orbiting within the vessel in continual fall and to prevent their contact with the vessel walls (Figures 1A,B). A total of two biological replicates were maintained throughout the experiments. An additional set of experiments identical to the conditions mentioned above was established for measurement of cell concentration and microscope observations. Cell concentration was assessed via hemocytometer count at different time points until completion of the experiment, using two biological replicates (i.e., biomass from two different vessels) for each experimental condition. Following removal of the vessel from the rotator base, samples of cells were collected under sterile conditions in a laminar flow hood using a luer-lock syringe and the syringe port in the culture vessel. The sampling was carried out one vessel at a time while the remaining vessels were kept rotating on the rotator base. Cell survivability at the end of the LSSMG exposure was assessed by enumeration of colony forming units (CFU) using ImageJ software (Schneider et al., 2012), according to Choudhry (2016).

### Microscopy Studies

#### Scanning Electron Microscopy (SEM)

For SEM studies, samples from the two strains were collected from all the established cultures—LSSMG and normal gravity condition (1G)—at various timepoints, i.e., 1, 3, 5, and 7 days from the beginning of the cultivation. A 1:10 dilution of each sample was prepared using 1X phosphate-buffered saline (PBS; Thermo Fisher Scientific) and thereafter transferred onto electron microscopy coupons. For each sample, two biological replicates (and two technical replicates each i.e., aliquots of 20 μl) were prepared and let dry at 35°C before examination. A Sirion (FEI, Hillsboro, OR, United States) field-emission scanning electron microscope (FE-SEM) was used for examination of the fungal cells. No specimen preparation procedures, such as sample coating with a surface-conducting (carbon or metal) layer, were performed as they were not necessary when using the FE-SEM. The samples were analyzed using an acceleration voltage of 10–20 kV, beam current of 40–50 mA and positioning the detector 6–10 mm away from the coupon. Secondary electron images were acquired in the high-vacuum mode. For each sample coupon, a minimum of 10 fields was observed, and images were acquired with various magnification (100×–5,000×).

#### Fluorescence Microscopy

For the cell integrity and viability assay, wheat germ agglutinin (WGA) and propidium iodide (PI) in combination with SYTO 9 were used. The carbohydrate-binding lectin WGA has a known affinity for β-1,4-N-acetylglucosamine (GlcNAc) oligomers present in the fungal polysaccharide chitin. PI, a membrane impermeant dye that is generally excluded from viable cells, was applied together with the nuclear and chromosome counterstain SYTO 9 for a dead/live stain. 200 μl aliquots were collected from all the established cultures after 1, 3, 5, and 7 days from the beginning of the cultivation. Fungal cells were washed twice in 1X PBS and suspended in 500 μl. Following a 1:10 dilution, 100 μl cell suspension was incubated with 50 μl Alexa Fluor 350 conjugate of WGA (Thermo Fisher Scientific) and 15 μl of FungaLight containing equal amounts of PI and SYTO 9 solution (Thermo Fisher Scientific), for 15 min at room temperature in the dark. The chosen amounts of the fluorescent probes are justified by the thickening and the increase in melanization of the cell walls, which hindered the staining process. Cell suspensions were mounted over glass slides and analyzed under an Axioplane microscope equipped with an AxioCam camera (Carl Zeiss). For each sample, two technical replicates were prepared for observation.

### Tandem Mass Tag (TMT)-Based Quantitative Shotgun Proteomics

#### Sample Preparation

The content of each vessel was spun at 7500 × g (5810, Eppendorf) and at 4°C for 15 min to separate fungal cell pellet (mycelium) from culture supernatant (secretome). Protease inhibitors (10 μl:1 mL v/v, Halt, Thermo Fisher Scientific) were thereafter added—and mixed with 1 mL 1 × PBS in the case of biomass samples—and samples were stored at −80°C prior to protein extraction. Proteins were extracted as previously described by Romsdahl et al. (2018) with some modifications. In brief, 1 mL lysis buffer consisting of 100 mM triethylammonium bicarbonate (TEAB) with 1:100 Halt Protease Inhibitor Cocktail (Thermo Fisher Scientific) was added to fungal cell pellets (i.e., ∼0.5 g) and culture supernatants (i.e., ∼0.5 mL), the latter previously concentrated in a SpeedVac system. Sample homogenization was achieved using a bead beater (Bertin) at 4°C (3 × 5500 rpm for 1 min., with 15 s. breaks in between) and following centrifugation at 17,000 × *g* and 4°C for 15 min, protein concentrations were determined by BCA assay (Thermo Fisher Scientific). The protein extracts were processed for a tandem mass tag (TMT) labeling as previously described (Romsdahl et al., 2018). For each sample, 250 μg proteins were precipitated in 20% TCA at 4°C, reduced by tris(2-carboxyethyl)phosphine (TCEP), alkylated with iodoacetamide (IAA), and digested with Trypsin/LysC (Promega, Madison, WI, United States) overnight at 37°C. Peptide quantitation was performed using the Pierce Quantitative Colorimetric Peptide Assay (Thermo Fisher Scientific). The proteomic and secretomic profiling of *K. chersonesos* Wt and Mut were carried out in two separate TMT LC/MS experiments. A total of 40 μg peptides from each cell pellet sample or 13 μg peptides from each culture supernatant were labeled with the Thermo Scientific TMT10 plex (TMT^10^) Isobaric Mass Tagging Kit according to the manufacturer protocol. Eight TMT tags were used to label samples from the same experimental set. The TMT^10^-131 label was used as a reference that contained a pool of 5 μg of peptides from all samples. All nine labeled-peptide samples were combined into a single tube, mixed and fractionated using the Pierce High pH Reversed-Phase Peptide Fractionation Kit (Thermo Fisher Scientific). The fractionated samples were dried using a SpeedVac concentrator and dissolved in 1% formic acid prior to LC-MS/MS analysis.

#### LC-MS/MS Analysis

The samples were analyzed on an Orbitrap Fusion Lumos mass spectrometer with a Dionex UltiMate 3000 RSLCnano system, a 300 μm × 5 mm PepMap100 C18 precolumn, a 75 μm × 50 cm PepMap RSLC C18 analytical column, and an Easy-Spray ion source (Thermo Scientific). The column temperature was maintained at 45°C, and the peptides were eluted at a flow rate of 300 nL/min over a 110 min gradient, from 3 to 30% solvent B (100 min), 30 to 50% solvent B (5 min), 50 to 90% solvent B (1 min), 90% solvent B (1 min), and 90% to 3% solvent B (3 min). Solvent A was 0.1% formic acid in water and solvent B was 0.1% formic acid in acetonitrile. The full MS survey scan (400–1,600 m/z) was acquired in the orbitrap at a resolution of 240,000 and with an automatic gain control (AGC) target of 4 × 10^5^. The maximum injection time for MS scans was 50 ms. Monoisotopic precursor ions were selected with charge states 2–7 within a ± 10 ppm mass window using a 60 s dynamic exclusion. The MS^2^ scan (400–1,200 m/z) was performed using the linear ion trap with the CID collision energy set to 35%. The ion trap scan rate was set to “rapid,” with an AGC target of 1 × 10^4^, and a maximum injection time of 30 ms. Ten fragment ions from each MS^2^ experiment were then simultaneously selected for an MS^3^ experiment. The MS^3^ scan (100–500 m/z) was performed to generate the TMT reporter ions in the orbitrap at a resolution of 30,000 and using HCD at a collision energy setting of 65%, an AGC target of 5 × 10^4^, and a maximum injection time of 54 ms.

#### Quantitative Proteomics Analysis

All MS/MS spectra were analyzed using Proteome Discoverer (version 2.2.0.388, Thermo Fisher Scientific) with the Sequest-HT searching engines against an in-house annotated draft genome sequence of *K. chersonesos* MA5789 Wt (GCA_002319055.1, assembly ASM231905v1, NCBI) (Tesei et al., 2017) consisting of 9.818 predicted protein coding gene models. The genome was annotated using funannotate (v1.3.4) (doi: 10.5281/zenodo.1284502), which combined predictions from *ab initio* gene predictors with Augustus trained by BUSCO gene models (fungi_odb9) (Zdobnov et al., 2017) and GeneMark.hmm (Ter-Hovhannisyan et al., 2008) informed by protein evidence from Swiss-Prot (Boutet et al., 2007) together into composite gene models with EvidenceModeler (Haas et al., 2008). Functional predictions for genes was assigned by protein homology to Pfam (El-gebali et al., 2019), Swiss-Prot/UniProt (v 2018_05), and EggNog (v1.10) databases (Huerta-Cepas et al., 2019). The following parameters were selected for the search: A maximum of two missed cleavage sites, a minimum peptide length of six residues, 5 ppm tolerance for precursor ion masses, and 0.6 Da tolerance for fragment ion masses. The static modification settings included carbamidomethyl of cysteine residues, and dynamic modifications included oxidation of methionine, TMT modification of lysine ε-amino groups and peptide N-termini, and acetyl modification of protein N-terminus. A false discovery rate (FDR) of 1% for peptides and proteins was obtained using a target-decoy database search. The reporter ions integration tolerance was 0.5 Da while the co-isolation threshold was 75%. The average signal-to-noise threshold of all reporter peaks was greater than 10. The quantitative abundance of each protein was determined from the total intensity of the detected reporter ions. For statistical analysis, the sum of reporter ion intensities for each protein was log2 transformed, and the technical triplicate measurements for each protein were averaged. Only the proteins that were identified with at least one peptide and quantified in all technical (*n* = 3) and biological replicates (*n* = 2), were considered for the statistical analysis. Student’s *t*-test was performed to identify differentially expressed proteins between each LSSMG-exposed and 1G-exposed group as well as between all groups. To compare all four experimental conditions, each condition was normalized to the reference channel, which contained equal amounts of peptides from each group. To evaluate changes between treatment (LSSMG) and control (1G), the protein abundance levels of each LSSMG-exposed sample were normalized to the 1G-exposed counterpart. Proteins with *p*-values of ≤0.05 were further evaluated for increased or decreased abundance using a cut-off value of ≥ ± 1.5-fold change (log2 fold change of ≥ ± 0.584).

### Protein Identification and Bioinformatics Analysis

Protein identifications and functional insights were obtained from searching for sequence homologs using OmicsBox v. 1.4.11 (BioBam Bioinformatics S.L). To characterize proteins with respect to the biological process they are involved in, Gene Ontology (GO) terms were assigned to domains. The sequences were blasted (cloud BLASTP 2.10.0+, *E*-Value 1.0E-3, Filter: Fungi), and the blast hits were mapped and annotated with GOs using the GO database (Goa version 2019_11^1^; *E*-Value 1.0E-6, Filter GO by Taxonomy: taxa: 4751, Fungi) (Götz et al., 2008). GOs were additionally assigned using InterProScan (IPS) to retrieve domains/motif information in a sequence-wise manner, and EggNOG using precomputed EggNOG-based orthology assignments. Corresponding GOs were then transferred to the sequences and merged with already existing GO terms. The annotations were validated based on the True-Path-Rule by removing all redundant terms to a given sequence. A GO-Slim analysis was run to summarize the GO annotation using the *Aspergillus* slim. Existing GO terms were additionally mapped to enzymes codes, when possible. A pathway analysis was performed to retrieve metabolic pathways based on the GO terms and the enzyme codes using the Load KEGG Pathway tool (Kanehisa and Goto, 2000). To elucidate the identity of the uncharacterized proteins, a search for homology was further performed in the UniProtKB database^2^ (BLASTP parameters: E-Threshold: 10; matrix BLOSUM62). In cases of blast results where the most significant match was represented by an uncharacterized protein, the first match in the list of homologous proteins where a protein ID was available was considered. Information about the predicted protein localization was obtained using BUSCA^3^, based on the identification of signal and transit peptides, GPI-anchors and alpha-helical and beta-stranded transmembrane domains (Savojardo et al., 2018). Protein-protein interaction analyses were performed using STRING v11.0 with high confidence (0.70) (Jensen et al., 2009), selecting the proteome of the black yeast *Exophiala dermatitidis* as reference database based on its phylogenetic proximity to *K. chersonesos* (Tesei et al., 2020).

## Results

### Growth Behavior

Cell concentration in the LSSMG-exposed and unexposed samples was measured via hemocytometer at four different time points: (1) start (seed, day 0), (2) acceleration phase (in between lag and exponential phases, day 3), (3) exponential phase (day 5) and (4) at the end of the experiment (stationary phase, day 7). In *K. chersonesos* Wt_LSSMG_, ∼23.5-fold increase in cell concentration was observed during acceleration phase when compared to the original inoculum (2 × 10^5^ cells/mL). Such an increase during acceleration phase was also noticed in *K. chersonesos* Mut_LSSMG_ (10-fold), *K. chersonesos* Wt_1__G_ (22-fold) and *K. chersonesos* Mut_1__G_ (19-fold). During stationary phase the increase in cell concentration was 28-fold (*K. chersonesos* Wt_LSSMG_), 30-fold (*K. chersonesos* Mut_LSSMG_), 42-fold (*K. chersonesos* Wt_1__G_), and 36-fold (*K. chersonesos* Mut_1__G_) when compared to original inoculum. Among LSSMG and Earth gravity grown cultures, higher values were recorded at normal gravity condition. Overall, only ∼one log growth in 7 days might be due to the clumping nature of *Knufia* cells. Cell survivability in simulated microgravity was measured by CFU using ImageJ software (Schneider et al., 2012) according to established protocol (Choudhry, 2016) and were: *K. chersonesos* Wt_LSSMG_ = 2.8 × 10^6^ cell/mL, *K. chersonesos* Mut_LSSMG_ 2.5 × 10^6^ cell/mL, *K. chersonesos* Wt_1__G_ 2.6 × 10^6^ cell/mL, *K. chersonesos* Mut_1__G_ 2.3 × 10^6^ cell/mL (Supplementary Table 1). Potential mechanical damages or cell lysis caused by cell separation via mild bead beating (i.e., ribolyzer, 3 × 20 s, speed 4) prior to cell count are to be excluded since optical microscope observations and tests involving repeated bead beater treatments indicated that it does not decrease cell viability. However, underestimation of cell survivability by CFU due to cell clumping, an inherent characteristic of black fungi, should be taken into account.

Under LSMMG conditions, fungal cells showed an increased clumping, resulting in the aggregation of cells to form a mycelial growth in the center of the vessel. However, growth morphology under Earth gravity resembled a biofilm of cells adhering to the vessel’s oxygenation membrane (Figure 1C).

### Microscopy

**FIGURE 2:**
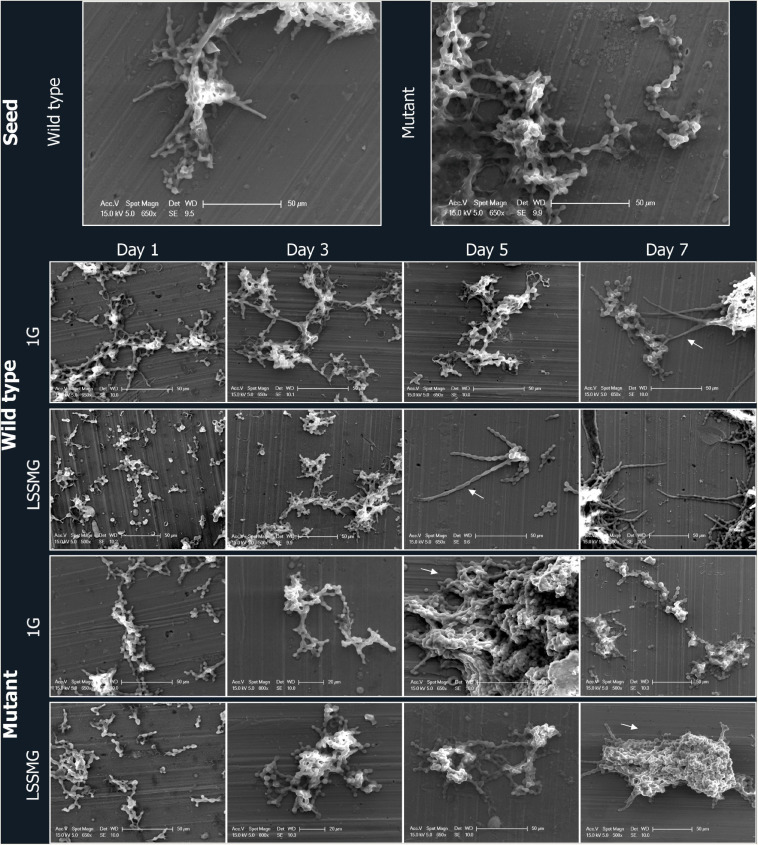
Field emission scanning electron microscopy (FE-SEM) micrographs of *Knufia chersonesos* Wt and Mut on aluminum coupons during a 7-days exposure to LSSMG or 1G (control). Arrows indicate hyphal growth in the wild type and dense cell aggregation in the mutant strain.

**FIGURE 3:**
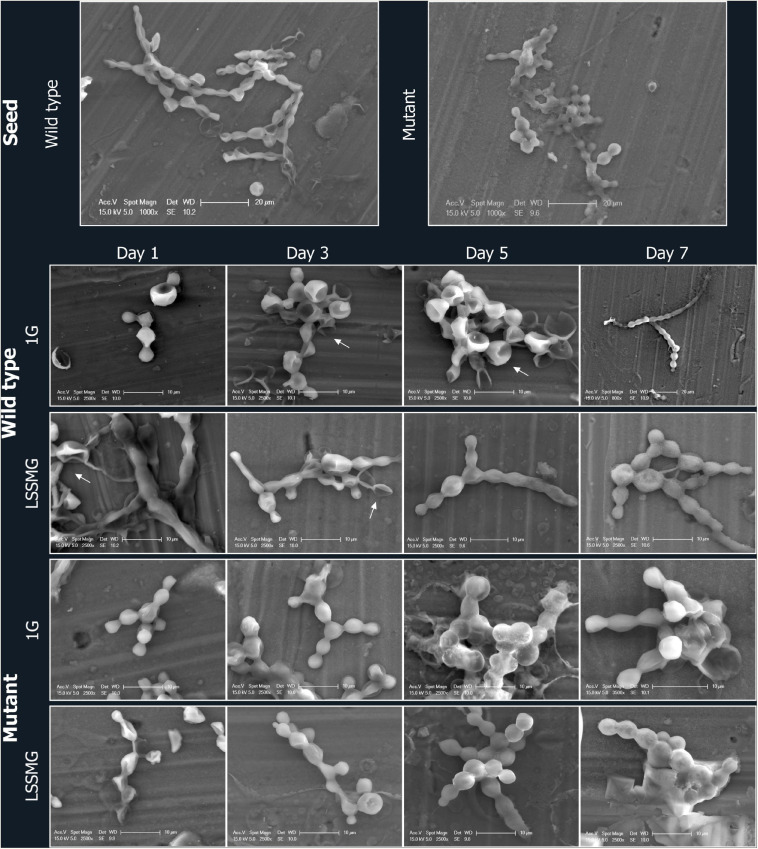
FE-SEM micrographs of *Knufia chersonesos* Wt and Mut on aluminum coupons during a 7-days exposure to LSSMG or 1G (control). Details of cells morphology are shown; arrows indicate biconcave cell morphology.

To evaluate the presence of LSSMG-dependent alterations of fungal cell morphology in the melanotic (Wt) and non-melanized spontaneous mutant (Mut) strain, we examined cellular differentiation by growing cells at low density. In order to analyze single cellular morphology of the Wt and Mut strain before LSSMG-treatment, exponentially growing fungal cells were spotted on top of aluminum coupons and visualized using FE-SEM. The observation of the control fungal cell morphology in non-coated FE-SEM specimens revealed that the Wt produced biconcave cells, pseudohyphae and hyphae, while the mutant showed smooth spherical cells, pseudohyphae and cell aggregates (Figures 2, 3).

Low-shear simulated microgravity-exposed and 1G-exposed fungal cells were collected at various time points, as described in the section “Materials and Methods.” A detailed examination of fungal cellular morphology in non-coated FE-SEM specimens revealed 1G and LSSMG Wt and mutant strains showing similar morphology at day 1 and 3. Remarkably, the 1G Wt hyphae formation was delayed until day 7, whereas LSSMG induced hyphae formation at day 5 (Figure 2). Notably, the mutant showed no evidence of hyphae formation in LSSMG condition, but the aggregation of cells was evident at day 3 and increased until day 7, similarly to what was observed in the 1G cells (Figure 2). Furthermore, a closer observation of Wt and mutant fungal cells in LSSMG and 1G condition at 2500× magnification clearly shows that the Wt cells form biconcave morphology compared to the mutant cells (Figure 3). LSSMG treatment led to no significant change in cell size relative to 1G conditions, in either strain (Supplementary Table 2). Similarly, images of PI-SYTO 9 and WGA staining generated by confocal microscopy did not reveal changes in cell viability and integrity in response to LSSMG exposure. Greatest fluorescence was seen with WGA at the beginning of the cultivation, although increasing concentrations of the dye were applied; this is most probably due to the thickening of the cell wall and increase of melanization, which naturally occurs in the fungus over the course of cultivation (Supplementary Figure 1). Collectively, these findings uncover that the LSSMG and 1G conditions did not influence the fungal cellular growth and morphology.

### Overview of Whole-Cell Proteome and Secretome Analysis

**FIGURE 4:**
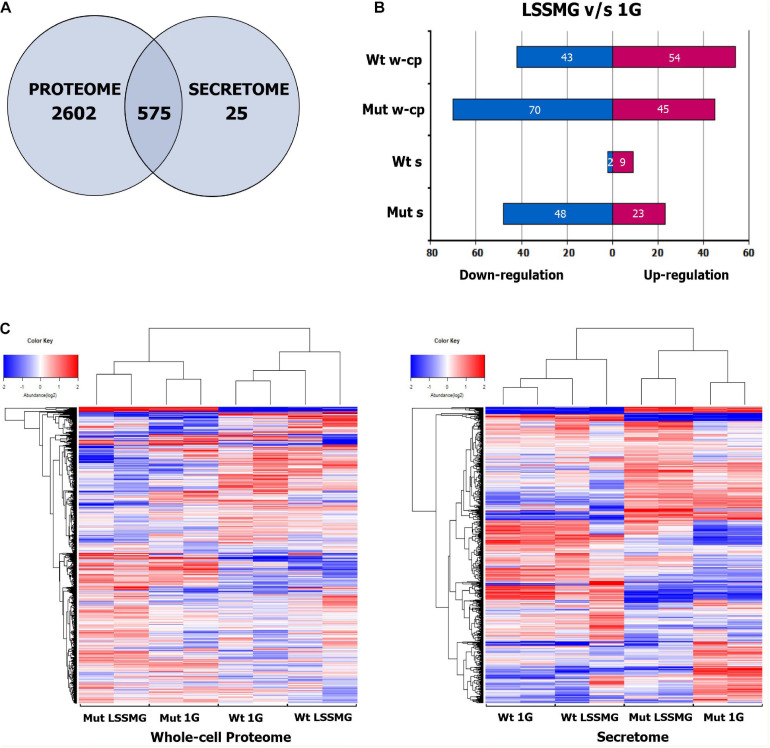
Proteins identified and detected as regulated in the whole-cell proteome and secretome of *Knufia chersonesos* Wt and Mut following exposure to LSSMG. **(A)** Overlap of proteins between whole-cell proteome and secretome, identified in two separate TMT LC/MS experiments. **(B)** Number of differentially abundant proteins for each strain under LSSMG compared to 1G (control) (fold change of ≥ ± 1.5, *p* ≤ 0.05). **(C)** Heat map hierarchical clustering of co-varying proteins quantified in each strain. The color key indicates the values that represented the protein abundance ratio (log2) and the number of proteins counted in each value. A total of 6 runs – 3 technical replicates and 2 biological replicates – were performed for each sample. Wt, wild type; Mut, mutant; w-cp, whole-cell proteome; s, secretome. Heatmaps were generated using R.

The 16 samples, comprised of four experimental conditions with two biological replicates each for each of the analyzed strains, yielded 3777 proteins as detected by isobaric TMT labeling-based LC-MS/MS. Of these, 3177 were in the whole-cell proteome and 600 in the secreted fraction. Furthermore, 2602 proteins were unique to the proteome and 25 to the secretome, while 575 were found to overlap (Figure 4A). A total of 3163 were identified by homology search. An overview of the biological and molecular functions of the detected proteins was obtained through an annotation statistics analysis. Distribution of the GO terms for all 3 categories (i.e., biological process BP, molecular function MF, and cellular component CC), with the highest number of associated protein sequences in the whole-cell proteome and the secretome, is displayed in Supplementary Figures 2A,B. Several GOs were common to proteome and secretome, whereas others were instead unique to each set of proteins. GOs for different types of cell metabolic processes were the most represented in both secretory and intracellular proteins, whereas BPs such as response to chemical, cell cycle, and negative regulation of cellular processes were found exclusively within the latter. Conversely, proteins involved in cell communication and positive regulation of cellular process could solely be detected in the secreted fraction of the proteome. Proteins with hydrolase activity (MF GO:0016787) represented the most abundant group in the secretome and second most abundant in the whole-cell proteome, immediately followed by proteins with organic cyclic and heterocyclic compound binding, oxidoreductase and transferase activity. In both sets of samples, proteins with transcription and translation regulation activity were instead detected as the smallest groups. A distribution analysis for cellular component GO terms indicated a prevalence of intracellular, membrane, and cell wall proteins (i.e., CC GO: envelope) also in the culture supernatant—i.e., the ratio of total proteins with predicted extracellular to intracellular localization was approximately 25%:75% in the wild type and 6%:94% in the mutant—where the detection of cytoplasmic proteins is possibly due to mechanical stress and cell death (Miura and Ueda, 2018). Nonetheless, the presence of these proteins in the secreted fraction could also depend on active secretion of proteins with no predicted signal peptide, i.e., non-classical protein secretion, which has been recognized in various organisms including fungi, bacteria and plants based on the large number of leaderless proteins detected in extracellular compartments, including extracellular vesicles (EVs) (Regente et al., 2012; Vallejo et al., 2012; Sun and Su, 2019). The 25 proteins uniquely detected in the secretome encompassed a number of carboxylic ester hydrolases with predicted extracellular localization alongside cytoplasmic, cell wall– and plasma membrane-associated proteins (Supplementary Table 3).

In order to detect significant rearrangements in the protein pool upon exposure to LSSMG, a quantitative analysis was carried out by comparing experimental groups. The clustering of the samples, biological and technical replicates included, was confirmed by Hierarchical Cluster (HC) analysis (Figure 4C). However, one of the LSSMG-exposed secretomes from *K. chersonesos* Wt appeared to deviate from the biological duplicate of the same condition. The number of differentially abundant proteins for each strain is summarized in Figure 4 (fold change of ≥ ± 1.5, *p* ≤ 0.05), where (B) shows the count of regulated proteins following direct comparison of treatment and control whole-cell proteome and secretome samples. The mutant proteome contained the largest number of modulated proteins (115) compared to the mutant secretome (71), the wild type proteome (97), and the wild type secretome (11).

### Effects of LSSMG on *Knufia chersonesos* Whole-Cell Proteome

**FIGURE 5:**
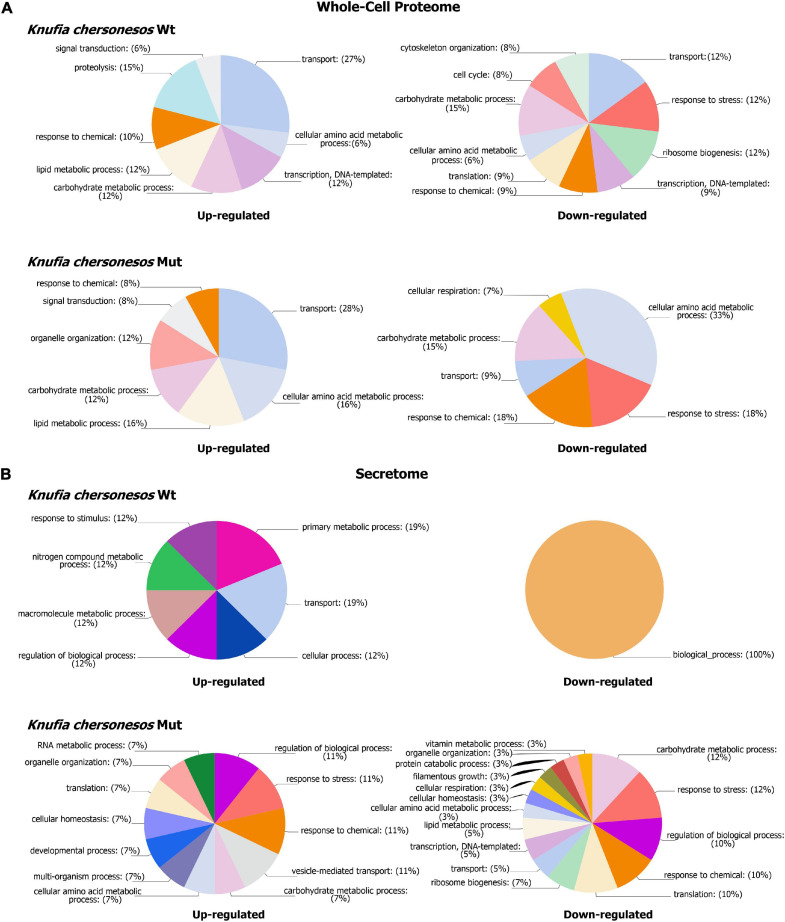
Biological processes GOs categories of differentially expressed proteins in *Knufia chersonesos* under LSSMG. **(A)** Whole-cell proteome **(B)** Secretome. Proteins with changed abundance (FC ≥ ± 1.5, *p* ≤ 0.05) were annotated with terms representing various biological processes using OmicsBox v. 1.4.11 (https://www.biobam.com). GO terms were thereafter summarized using sequence distribution/GO multilevel pie charts (Filtered by sequence count, Cutoff = 2).

The proteomic quantitative analysis of LSSMG-exposed *K. chersonesos* Wt and Mut revealed 54-up and 43-down and 45-up and 70-down regulated proteins, respectively, when compared to the 1G-exposed counterparts (fold change of ≥ ± 1.5, *p* ≤ 0.05) (Supplementary Tables 4, 5). Distribution of over-represented BP GO terms among differentially expressed proteins is displayed in Figure 5A. Most significantly upregulated biological processes included “lipid and carbohydrate metabolism” in both strains (24 to 28% of all upregulated proteins). However, 15% of downregulated proteins were also in the carbohydrate metabolism category. By contrast, proteins involved in lipid metabolic processes were not found to be decreased. Further, “transcription” (12%) and “transport” (27%) were additional highly represented categories of upregulated proteins in the wild type, whereas “transport” (28%) and “amino acid metabolic process” (16%) were well represented categories of upregulated proteins in the mutant.

Proteins involved in lipid metabolism included CF317_002955-T1/C7ZGD6_NECH7—predicted to be phospholipase A2—an acetyl-CoA desaturase (CF317_004579-T1/A0A072PJ62_9EURO) and the uncharacterized protein CF317_000532-T1/A0A0D2C8U2_9EURO upregulated in both strains, along with an inositol-3-phosphate synthase (CF317_006213-T1/A0A0D2GI50_9EURO), which was instead solely observed in the mutant. These proteins participate in cellular pathways for the biosynthesis of unsaturated fatty acids (KEGG pathway 01040) and the metabolism of phospho- and glycerophospholipids (KEGG 00564), inositol phosphate (KEGG 00562), arachidonic and linoleic acid, among others (Table 1; Passoth, 2017). Pyruvate metabolism (KEGG 00620), glycolysis/gluconeogenesis (KEGG 00010) and starch and sucrose metabolism, were possibly also involved in the response to LSSMG, as revealed by the KEGG pathway analysis based on the increased abundance of proteins like alcohol dehydrogenase 1 (CF317_005767-T1/A0A0N0NIW7_9EURO) and alpha/beta-glucosidase agdC (CF317_006579-T1/A0A178BWA1_9EURO) in the wild type. Glutamine synthetase and protein FYV10, the first involved in glyoxylate and dicarboxylate metabolism (KEGG 00630) and in nitrogen metabolism (Zhang et al., 2017) and the latter mediating the degradation of enzymes of the gluconeogenesis pathway (Braun et al., 2011), were instead detected as upregulated in the mutant. Decreased levels of a number of hydrolytic enzymes destined for secretion were observed in the LSSMG-exposed proteomes for the carboxylic ester hydrolases CF317_0002308-T1/A0A1J9RJA8_9PEZI, CF317_007618-T1/A0A6A6HNE7_9PEZI, CF317_007621-T1/A0A0D2AG04_9PEZI (in Wt) and CF317_002086-T1/A0A0D2BHT9_9EURO (in Mut). The same was observed for the cell wall–degradation enzymes endo- and extracellular glucanases CF317_0009683-T1/W9ZBZ7_9EURO and CF317_009779-T1/H6BQE2_EXODN (in Wt) and the 3,2-*trans-*enoyl-CoA isomerase CF317_000932-T1/A0A1C1D1N7_9EURO (in Mut), the latter of which is involved in the metabolism of unsaturated fatty acids in beta oxidation (Janssen et al., 1994).

**TABLE 1:** Most abundant biological process GO categories regulated under LSSMG in *Knufia chersonesos* Wt and Mut whole-cell proteome. Differentially expressed proteins included in each category are also shown.

*Protein* accession No.^a^	Putative Protein function	Protein relative abundance*	*p*-value
**Carbohydrate and lipid metabolic process**			
*Wild type*			
CF317_003648-T1	GP-PDE domain-containing protein	1.1133	4.79E-02
CF317_002955-T1	Phospholipase A2	1.1032	3.26E-01
CF317_005767-T1	Alcohol dehydrogenase 1	0.8542	9.37E-02
CF317_006579-T1	Alpha/beta-glucosidase agdC	0.8177	6.11E-03
CF317_009031-T1	Carboxylic ester hydrolase	0.8024	4.22E-02
CF317_000532-T1	Uncharacterized protein	0.775	3.78E-01
CF317_007434-T1	Serine/threonine-protein kinase ppk6	0.6797	1.44E-01
CF317_004579-T1	Acyl-CoA desaturase	0.6334	1.95E-05
CF317_0002308-T1	Carboxylic ester hydrolase	−0.6053	1.47E-02
CF317_0009683-T1	Endo-1,3(4)-beta-glucanase	−0.6918	2.10E-02
CF317_007618-T1	Carboxylic ester hydrolase	−0.7365	4.22E-02
CF317_009779-T1	Extracellular cell wall glucanase Crf1/allergen Asp F9	−0.8811	2.70E-01
CF317_009003-T1	Serine/threonine-protein kinase TOR	−0.9558	9.75E-02
CF317_007621-T1	Cutinase	−1.0366	7.69E-04
*Mutant*			
CF317_009031-T1	Carboxylic ester hydrolase	1.1163	4.22E-02
CF317_007501-T1	Glutamine synthetase	0.9516	2.84E-01
CF317_006213-T1	Inositol-3-phosphate synthase	0.8851	2.34E-01
CF317_004579-T1	Acyl-CoA desaturase	0.8258	1.95E-05
CF317_000532-T1	Uncharacterized protein	0.7363	3.78E-01
CF317_007133-T1	Protein FYV10	0.6298	1.26E-02
CF317_002086-T1	AB hydrolase-1 domain-containing protein	−0.6016	1.98E-01
CF317_009017-T1	NodB homology domain-containing prote**i**n	−0.6135	5.38E-02
CF317_005813-T1	4HBT domain-containing protein	−0.6213	7.10E-02
CF317_001523-T1	Glucose-6-phosphate 1-epimerase	−0.6226	6.42E-02
CF317_000932-T1	3,2-*trans-*enoyl-CoA isomerase	−0.6255	1.20E-02
CF317_009683-T1	Endo-1,3(4)-beta-glucanase	−1.0502	2.10E-02
**Transport**			
*Wild type*			
CF317_004246-T1	Phosphate transporter	1.3345	4.85E-01
CF317_007988-T1	VPS37 C-terminal domain-containing protein	0.8635	5.08E-01
CF317_0005675-T1	Mitochondrial thiamine pyrophosphate carrier 1	0.7750	8.05E-03
CF317_001330-T1	Phosphate transporter	0.7548	1.35E-01
CF317_001709-T1	WD_REPEATS_REGION domain-containing protein	0.7277	5.37E-01
CF317_003773-T1	Zinc-regulated transporter 1	0.6633	1.42E-02
CF317_009191-T1	Choline transport protein	0.6590	9.34E-03
CF317_007500-T1	Ammonium transporter	0.6548	2.80E-01
CF317_008807-T1	HemS domain-containing protein	0.5860	2.75E-01
CF317_0006744-T1	Ribosomal protein L37e	−0.6649	1.96E-01
CF317_0001542-T1	POT family proton-dependent oligopeptide transporter	−0.6830	8.37E-03
CF317_0009683-T1	Endo-1,3(4)-beta-glucanase	−0.6918	2.10E-02
CF317_001911-T1	MFS transporter, SP family, major inositol transporter	−0.8427	1.84E-02
*Mutant*			
CF317_007500-T1	Ammonium transporter	1.6152	2.80E-01
CF317_009191-T1	Choline transport protein	1.2684	9.34E-03
CF317_005369-T1	AA_permease domain-containing protein	0.9504	4.30E-01
CF317_003222-T1	CNT family concentrative nucleoside transporter	0.7381	4.62E-01
CF317_008807-T1	HemS domain-containing protein	0.7308	2.75E-01
CF317_002497-T1	SEC7 domain-containing protein	0.7191	7.38E-02
CF317_009070-T1	Stress response protein NST1	0.6604	1.86E-01
CF317_002132-T1	AA_permease domain-containing protein	0.6505	4.55E-02
CF317_001911-T1	MFS transporter, SP family, major inositol transporter	−0.7301	1.84E-02
CF317_001542-T1	POT family proton-dependent oligopeptide transporter	−0.8932	8.37E-03
CF317_009683-T1	Endo-1,3(4)-beta-glucanase	−1.0502	2.10E-02
**Transcription, RNA and cellular amino acid metabolic processes**			
*Wild type*			
CF317_004755-T1	Glutamate dehydrogenase	1.7627	3.54E-01
CF317_004246-T1	Phosphate transporter	1.3345	4.85E-01
CF317_008227-T1	Alkaline phosphatase	1.2107	4.76E-01
CF317_003892-T1	Alkaline phosphatase	1.0475	4.25E-01
CF317_005207-T1	Ribonuclease T1	0.9813	3.38E-01
CF317_005767-T1	Alcohol dehydrogenase 1	0.8542	9.37E-02
CF317_004551-T1	Carboxypeptidase	0.6315	5.66E-01
CF317_009661-T1	Fungal_*trans* domain-containing protein	−0.5936	4.26E-01
CF317_009392-T1	Fungal_*trans* domain-containing protein	−0.6382	1.93E-01
CF317_0006744-T1	Ribosomal protein L37e	−0.6649	1.96E-01
CF317_000112-T1	60S ribosomal protein L34-B	−0.7009	9.84E-02
CF317_000184-T1	Large subunit ribosomal protein L24e	−0.7218	2.77E-01
CF317_003175-T1	Poly(A) polymerase	−0.7450	9.79E-03
CF317_006402-T1	4-hydroxyphenylpyruvate dioxygenase	−0.7730	4.57E-04
CF317_009779-T1	Extracellular cell wall glucanase Crf1/allergen Asp F9	−0.8811	2.70E-01
CF317_009003-T1	Serine/threonine-protein kinase TOR	−0.9558	9.75E-02
CF317_002178-T1	Indoleamine 2,3-dioxygenase	−1.1838	5.88E-04
*Mutant*			
CF317_004755-T1	Glutamate dehydrogenase	1.9810	3.54E-01
CF317_007501-T1	Glutamine synthetase	0.9516	2.84E-01
CF317_001165-T1	Malic enzyme	0.6220	4.41E-01
CF317_004816-T1	D-3-phosphoglycerate dehydrogenase	0.6044	5.51E-03
CF317_002693-T1	Ornithine transcarbamylase	−0.5899	2.51E-02
CF317_000519-T1	Arginase	−0.6439	1.12E-02
CF317_006712-T1	Histidinol dehydrogenase	−0.6459	2.39E-02
CF317_008030-T1	D-amino-acid oxidase domain-containing protein	−0.6799	2.92E-02
CF317_008924-T1	Homogentisate 1,2-dioxygenase	−0.6970	5.01E-01
CF317_007970-T1	Multifunctional fusion protein	−0.7153	4.07E-01
CF317_000649-T1	Delta(3,5)-Delta(2,4)-dienoyl-CoA isomerase, mitochondrial	−0.7416	1.03E-02
CF317_007412-T1	Histidinol-phosphate aminotransferase	−0.7514	5.09E-02
CF317_003892-T1	Alkaline phosphatase	−0.8825	4.25E-01
CF317_007070-T1	Multifunctional fusion protein	−0.9090	6.03E-02
CF317_003175-T1	Poly(A) polymerase	−0.9882	9.79E-03
CF317_006402-T1	4-hydroxyphenylpyruvate dioxygenase	−1.1274	4.57E-04
CF317_002178-T1	Indoleamine 2,3-dioxygenase	−1.4328	5.88E-04
**Response to stress and chemical**			
*Wild type*			
CF317_004755-T1	Glutamate dehydrogenase	1.7627	3.54E-01
CF317_006388-T1	Nitric oxide dioxygenase	0.9459	3.16E-01
CF317_005767-T1	Alcohol dehydrogenase 1	0.8542	9.37E-02
CF317_003242-T1	Catalase	−0.6330	7.85E-02
CF317_009003-T1	Serine/threonine-protein kinase TOR	−0.9558	9.75E-02
CF317_007018-T1	3-phytase	−0.9947	4.67E-04
CF317_000687-T1	Catalase	−1.1410	6.34E-02
*Mutant*			
CF317_004755-T1	Glutamate dehydrogenase	1.9810	3.54E-01
CF317_006388-T1	Nitric oxide dioxygenase	0.7290	3.16E-01
CF317_004909-T1	Adenylosuccinate synthetase	−0.6917	8.11E-02
CF317_007970-T1	Multifunctional fusion protein	−0.7153	4.07E-01
CF317_008612-T1	Aldedh domain-containing protein	−0.7312	2.05E-01
CF317_007412-T1	Histidinol-phosphate aminotransferase	−0.7514	5.09E-02
CF317_008426-T1	Glutathione reductase	−0.7680	8.19E-02
CF317_007018-T1	3-phytase	−0.8087	4.67E-04
CF317_000687-T1	Catalase	−0.8987	6.34E-02
CF317_008773-T1	Catalase-peroxidase	−0.9459	3.42E-02
**Proteolysis**			
*Wild type*			
CF317_008840-T1	Carboxypeptidase	1.0950	6.46E-01
CF317_004157-T1	Peptidase_M14 domain-containing protein	0.8180	2.52E-01
CF317_008998-T1	Putative fumarylacetoacetate hydrolase	0.7082	7.54E-02
CF317_000103-T1	Zinc carboxypeptidase	0.6512	1.88E-01
CF317_004551-T1	Carboxypeptidase	0.6315	5.66E-01
**Cytoskeleton and organelle organization**			
*Wild type*			
CF317_009683-T1	Endo-1,3(4)-beta-glucanase	−0.6918	2.10E-02
CF317_009003-T1	Serine/threonine-protein kinase TOR	−0.9558	9.75E-02
*Mutant*			
CF317_008503-T1	Thiamine thiazole synthase	0.8255	2.30E-01
CF317_007856-T1	WD_REPEATS_REGION domain-containing protein	0.7141	1.88E-02

*Log2 fold change of SMG-exposed compared to unexposed proteome (*p* ≤ 0.05). ^a^Protein accession number in the *K. chersonesos* database of *ab initio* translated proteins.

A differential abundance of proteins involved in transport was also observed (Table 1). Transmembrane ammonium (CF317_007500-T1/A0A072PWW7_9EURO) and iron transporters (i.e., HemS domain-containing protein CF317_008807-T1/L7HNA7_MAGOY) and the choline transport protein (CF317_009191-T1/A0A2P8A4S9_9PEZI) were present in the LSSMG-exposed whole-cell proteome of both strains, but they were more increased in the mutant (over 3-, 2. 4-, and 1.5-folds, respectively) than in the wild type (slightly over 1.5-folds). Additionally, two AA permease 2 domain-containing proteins (CF317_005369-T1/A0A0D2FEL3_9EURO and CF317_002132-T1/A0A438N399_EXOME) responsible for transmembrane amino acid transport were also upregulated in the mutant, whereas proteins with a predicted role in vesicle-mediated transport—i.e., VPS37 C-terminal domain-containing protein (CF317_007988-T1/A0A0D2CPQ3_9EURO) and WD_REPEATS_REGION domain-containing protein (CF317_001709-T1/A0A438MTR2_EXOME)—were exclusively detected in the wild type counterpart. Downregulated proteins included POT family proton-dependent oligopeptide transporter (CF317_001542-T1/A0A072P870_9EURO) and major facilitator superfamily transporters (MFS) (CF317_001911-T1/W9Z1V7_9EURO), involved in transportation of substrate molecules, including sugars, drugs, metabolites, amino acids, vitamins, and both organic and inorganic ions, or small peptides (Pao and Paulsen, 1998). Together with ABC transporters, MFS transporters are often detected among the cell wall components undergoing changes in response to the shift from normal gravity to microgravity (Sathishkumar et al., 2016).

Low-shear simulated microgravity additionally triggered upregulation of some proteins involved in cellular amino acid metabolic processes and downregulation of others (Table 1). Glutamate dehydrogenase was threefold and fourfold upregulated in *K. chersonesos* Wt and Mut, respectively. Together with the glutamine synthase, whose levels were also found to be increased in the mutant, the enzyme is reportedly involved in the primary nitrogen metabolism and in the biosynthesis and metabolism of several amino acids (i.e., arginine biosynthesis, glutamine, alanine and aspartate metabolism) (Meti et al., 2011). Protein D-3-phosphoglycerate dehydrogenase (CF317_004816-T1/A0A0D2CHE2_9EURO), participating in cysteine, methionine, glycine, serine, and threonine metabolism, was also more enriched in LSSMG-exposed mutant samples. However, a higher number of enzymes involved in cellular amino acid metabolic processes was found to be decreased under LSSMG. Nine were downregulated exclusively in the mutant, while protein CF317_006402-T1 was observed also in the wild type. According to the KEGG analysis, some of these proteins are involved with more than one pathway: 4 proteins are implicated in arginine and proline metabolism (CF317_000519-T1, CF317_007970-T1, CF317_007070-T1, CF317_008030-T1), 3 in tyrosine metabolism (CF317_007412-T1, CF317_006402-T1, CF317_008924-T1), and 2 in histidine metabolism (CF317_007412-T1, CF317_006712-T1), arginine biosynthesis (CF317_002693-T1, CF317_000519-T1) and phenylalanine biosynthesis and metabolism (CF317_007412-T1, CF317_006402-T1). The putative indoleamine 2,3-dioxygenase, involved instead in tryptophan catabolic process to the NAD precursor kynurenine, represented the most decreased protein in both strains, with an over 2-fold downregulation.

Some proteins falling into the GO category “DNA-templated transcription” were enriched exclusively in the LSSMG-exposed wild type proteome. They were mostly phosphatases—known to also regulate pathways important for stress (Serra-Cardona et al., 2015)—having abundances nearly 2.5-fold higher than in the control samples (i.e., Alkaline phosphatase CF317_008227-T1/A0A0D2BGD8_9EURO and CF317_003892-T1/A0A0D2BGD8_9EURO) (Table 1). Within the same category, downregulation was observed for a putative serine/threonine-protein kinase TOR and two *trans* domain-containing proteins involved in DNA binding and transcription. Decreased levels of structural protein constituents of the large ribosomal subunit—i.e., ribosomal protein L37e, L34-B and L24e—were instead detected upon LSSMG exposure exclusively in the wild type, alongside the upregulation of the ribonuclease T1 (CF317_005207-T1/W9XBV2_9EURO) involved in RNA degradation. Also significantly decreased was poly(A)polymerase (CF317_003175-T1/A0A1C1CN69_9EURO), an important component in mRNA synthesis, responsible for the addition of the 3′ polyadenine tail to a newly synthesized pre-messenger RNA.

In the group of proteins involved in response to stress and chemical, most of the modulated proteins were decreased by LSSMG exposure (Table 1). Downregulated stress response proteins included catalases (i.e., CF317_000687-T1/R8BNJ4_TOGMI, levels 2.7- and 1.9-fold lower than in the unexposed wild type and mutant proteome, respectively; CF317_003242-T1/R4XE87_TAPDE and CF317_008773-T1/U1G9G0_ENDPU), notably involved in the response to oxidative stress, and the 3-phytase CF317_007018-T1/A0A0D2IHR9_9EURO—a phosphatase enzyme with a predicted role in counteracting phosphate deficiency and osmotic stress (Belgaroui et al., 2018)—in both strains. Further, the glutathione-recycling enzyme glutathione reductase (CF317_008426-T1/A0A0D1ZJU8_9EURO) (Couto et al., 2016), the histidinol-phosphate aminotransferase (CF317_007412-T1/H6BQ73_EXODN) and the adenylosuccinate synthetase (ASS; F317_004909-T1/W9W243_9EURO), showed lower levels in the mutant under LSSMG conditions. The latter, best known as the first enzyme in the *de novo* synthesis of AMP from inosine-5′-monophosphate (IMP), is responsive to salt stress, as reported in plants (Zhao et al., 2013). Among the proteins with increased abundance in both fungal strains, was glutamate dehydrogenase (CF317_004755-T1), involved in amino acid metabolism and for which a stress-dependent regulation has been demonstrated (e.g., abiotic stress generated ROS) (Skopelitis et al., 2006). Further, the putative cytosolic nitric oxide dioxygenase (CF317_006388-T1/S7Z8A1_PENO1)—reported to play a role in nitric oxide detoxification mechanisms by the conversion of this signaling molecule to nitrate (Cánovas et al., 2016)—was present in the wild type and mutant LSSMG-exposed proteomes at levels around 2-fold higher than that of the unexposed ones. Along with the above-mentioned proteins, a number of enzymes, specifically peptidases involved in proteolysis, were found to be upregulated exclusively in *K. chersonesos* wild type (Table 1). The putative carboxypeptidase CF317_008840-T1/W9XS38_9EURO and CF317_004157/W2S3B3_9EURO; and CF317_000103/A0A6A6DDQ8_9PEZI and CF317_004551/A0A0D2GVY9_9EURO, known to catalyze reactions that are important to various physiological processes, such as the cell cycle, cell growth and differentiation, apoptosis, and stress response (Neto et al., 2018), were 2-fold and 1.5-fold upregulated, respectively. Lastly, proteins involved in cytoskeleton and organelle organization showed opposite regulation in the two *K. chersonesos* strains: The first was decreased in the wild type, whereas the latter was upregulated in the mutant (Table 1).

### Effects of LSSMG on *Knufia chersonesos* Secretome

The proteomic characterization of *K. chersonesos* secretome upon exposure to LSSMG for 7 days revealed solely 9-up and 2-down regulated proteins in the wild type and 23-up and 48-down regulated proteins in the mutant, when compared to the 1G-exposed counterparts (fold-change of ≥ ± 1.5, *p* ≤ 0.05) (Figure 4B). Among these proteins, the ratio of proteins with predicted extracellular to intracellular localization was approximately 20%:80% in both wild type and mutant (Supplementary Tables 6, 7). Out of 14 proteins with predicted signal peptides in the mutant secretome, 12 were regulated following LSSMG-exposure. In the wild type secretome, 2 out of 61 proteins with predicted extracellular localization were regulated in response to microgravity.

The distribution of differentially expressed proteins among BP GO terms is presented in Figure 5B. In the wild type, the regulated proteins were prevalently involved in transport and metabolic processes, whereas in the mutant strain, regulated proteins were mostly involved with biological processes such as carbohydrate and lipid metabolism (15 proteins), response to chemical and stress (13 proteins), translation, and ribosome biogenesis and transcription (15 proteins). Interestingly, the majority of proteins involved in the above-mentioned GO BP categories exhibited downregulation in LSSMG-exposed secretomes of the mutant and exhibited upregulation in the wild type (Table 2). One protein exhibiting opposite regulation in the two analyzed strains, was the putative scytalone dehydratase CF317_002654-T1, homolog of *Phialophora attinorum* A0A0N1P280_9EURO. This enzyme, which is reportedly involved in the biosynthesis of dihydroxynaphthalene (DNH) melanin from endogenous substrate (Eisenman and Casadevall, 2012), was 1.5-fold increased in the wild type and 1.6-fold decreased in the melanin-deficient mutant. In the wild type, upregulated proteins also included the adenylosuccinate lyase CF317_004119-T1 and the M20_dimer domain-containing protein (Carboxypeptidase S) CF317_006825-T1, both implicated in amino acid (alanine, aspartate and glutamate) metabolism (KEGG pathway pae00250), and the plasma membrane ATPase CF317_002664-T1, partaking in oxidative phosphorylation (Table 2). Also in the wild type, the casein kinase I 1 CF317_004827-T1 and the histone H2A CF317_001633-T1, both involved in DNA repair (Skoneczna et al., 2018), were found in the LSSMG-exposed secretome at levels 1.7- and 1.6-fold higher than in the unexposed one. The two downregulated proteins were protein CF317_008131-T1, homolog of the arabinan endo-1,5-alpha-L-arabinosidase from *Aspergillus wentii DTO 134E9*, and protein CF317_002949-T1, homolog of the feruloyl esterase A0A177BY00_9PLEO from *Paraphaeosphaeria sporulosa*, both with a predicted extracellular localization and reportedly involved in polysaccharide degradation (Tesei et al., 2020).

**TABLE 2:** Most abundant biological process GO categories regulated under LSSMG in *Knufia chersonesos* Wt and Mut whole-cell secretome. Differentially expressed proteins included in each category are shown.

*Protein* accession No.^a^	Putative Protein function	Protein relative abundance*	*p*-value
**Carbohydrate and lipid metabolic processes**			
*Wild type*			
CF317_004827-T1	Casein kinase I 1	0.656	4.63E-02
CF317_002949-T1	Feruloyl esterase	−0.859	1.16E-03
CF317_008131-T1	Arabinan endo-1,5-alpha-L-arabinosidase	−0.998	4.32E-02
*Mutant*			
CF317_003266-T1	Malate dehydrogenase	1.244	3.48E-02
CF317_000614-T1	1,3-beta-D-glucan-UDP glucosyltransferase	0.893	2.26E-02
CF317_004588-T1	Alpha, alpha-trehalase	0.813	2.42E-02
CF317_003037-T1	Acyl-CoA-dependent ceramide synthase	0.797	1.16E-03
CF317_000059-T1	PSDC domain-containing protein	−0.634	7.14E-03
CF317_004317-T1	3′(2′),5′-bisphosphate nucleotidase	−0.650	4.52E-02
CF317_008979-T1	Epimerase domain-containing protein	−0.727	2.36E-02
CF317_003714-T1	Mannitol-1-phosphate 5-dehydrogenase	−0.739	9.36E-05
CF317_008448-T1	Phosphotransferase	−0.790	4.34E-04
CF317_003513-T1	2-phosphoglycerate dehydratase	−0.870	1.53E-03
CF317_006165-T1	Esterase/lipase	−0.917	9.78E-03
CF317_003202-T1	Concanavalin A-like lectin/glucanase	−0.918	4.79E-02
CF317_005481-T1	6-phosphogluconate dehydrogenase, decarboxylating	−0.960	7.57E-04
CF317_004751-T1	Phosphoglycerate kinase	−1.143	8.27E-04
CF317_007096-T1	6-phosphofructo-2-kinase	−1.494	5.59E-03
**Transport**			
*Wild type*			
CF317_001911-T1	MFS transporter, SP family, major inositol transporter	0.747	2.92E-02
CF317_002664-T1	Plasma membrane ATPase	0.702	3.25E-02
CF317_004827-T1	Casein kinase I 1	0.656	4.63E-02
*Mutant*			
CF317_000614-T1	1,3-beta-D-glucan-UDP glucosyltransferase	0.893	2.26E-02
CF317_001401-T1	t-SNARE coiled-coil homology domain-containing protein	0.843	4.08E-02
CF317_004472-T1	Endoplasmic reticulum transmembrane protein	0.742	4.09E-04
CF317_006347-T1	Inorganic phosphate transport protein PHO88	0.588	4.10E-02
CF317_002531-T1	Putative inorganic phosphate transporter C8E4.01c	−0.591	1.01E-03
CF317_007808-T1	NTF2 domain-containing protein	−0.756	2.95E-02
CF317_008448-T1	Phosphotransferase	−0.790	4.34E-04
**Transcription, RNA and cellular amino acid metabolic processes**			
*Wild type*			
CF317_001633-T1	Histone H2A	0.752	9.78E-03
CF317_002935-T1	Aromatic amino acid aminotransferase	0.633	1.18E-02
*Mutant*			
CF317_008030-T1	D-amino-acid oxidase domain-containing protein	0.783	1.10E-02
CF317_003734-T1	Ribosomal_L23eN domain-containing protein	0.771	4.09E-02
CF317_007665-T1	Guanine nucleotide-binding protein subunit beta	0.775	2.98E-03
CF317_005931-T1	Aspartate–tRNA ligase	0.742	2.84E-02
CF317_004317-T1	3′(2′),5′-bisphosphate nucleotidase	−0.650	4.52E-02
CF317_003885-T1	Putative RNA-binding protein	−0.668	4.83E-03
CF317_001861-T1	40S ribosomal protein S20	−0.709	4.62E-03
CF317_008924-T1	Homogentisate 1,2-dioxygenase	−0.730	4.76E-02
CF317_005516-T1	60S ribosomal protein L7	−0.734	1.03E-03
CF317_008448-T1	Phosphotransferase	−0.790	4.34E-04
CF317_004501-T1	40S ribosomal protein S24	−0.796	1.53E-02
CF317_001633-T1	Histone H2A	−1.084	9.78E-03
CF317_007960-T1	60S acidic ribosomal protein P1	−1.277	9.18E-03
CF317_008933-T1	Elongation factor EF-1 beta subunit	−1.706	2.09E-02
CF317_000379-T1	Elongation factor EF-1 gamma subunit	−1.838	2.56E-03
**Response to stress and chemical**			
*Wild type*			
CF317_001633-T1	Histone H2A	0.752	9.78E-03
CF317_004827-T1	Casein kinase I 1	0.656	4.63E-02
*Mutant*			
CF317_000051-T1	Plasma membrane phosphatase required for sodium stress response	0.906	1.73E-02
CF317_004588-T1	Alpha, alpha-trehalase	0.813	2.42E-02
CF317_007665-T1	Guanine nucleotide-binding protein subunit beta	0.775	2.98E-03
CF317_006969-T1	HRXXH domain-containing protein	0.696	4.88E-02
CF317_004317-T1	3′(2′),5′-bisphosphate nucleotidase	−0.650	4.52E-02
CF317_003570-T1	S-formylglutathione hydrolase	−0.720	1.69E-02
CF317_008979-T1	Epimerase domain-containing protein	−0.727	2.36E-02
CF317_003714-T1	Mannitol-1-phosphate 5-dehydrogenase	−0.739	9.36E-05
CF317_003513-T1	2-phosphoglycerate dehydratase	−0.870	1.53E-03
CF317_005481-T1	6-phosphogluconate dehydrogenase, decarboxylating	−0.960	7.57E-04
CF317_000980-T1	Thioredoxin domain-containing protein	−0.983	2.08E-02
CF317_001633-T1	Histone H2A	−1.084	9.78E-03
CF317_007147-T1	Nucleoside diphosphate kinase	−1.745	8.70E-04
**Biological processes and secondary metabolic process**			
*Wild type*			
CF317_001633-T1	Histone H2A	0.752	9.78E-03
CF317_004119-T1	Adenylosuccinate lyase	0.703	1.58E-02
CF317_006825-T1	M20_dimer domain-containing protein	0.588	1.22E-02
CF317_002654-T1	Scytalone dehydratase	0.587	3.70E-02
CF317_002949-T1	Feruloyl esterase	−0.859	1.16E-03
*Mutant*			
CF317_000614-T1	1,3-beta-D-glucan-UDP glucosyltransferase	0.893	2.26E-02
CF317_006969-T1	HRXXH domain-containing protein	0.696	4.88E-02
CF317_003885-T1	Putative RNA-binding protein	−0.668	4.83E-03
CF317_002654-T1	Scytalone dehydratase	−0.679	3.70E-02
CF317_006992-T1	Serine/threonine-protein kinase	−0.682	2.90E-02
CF317_008448-T1	Phosphotransferase	−0.790	4.34E-04
CF317_003513-T1	2-phosphoglycerate dehydratase	−0.870	1.53E-03
CF317_000980-T1	Thioredoxin domain-containing protein	−0.983	2.08E-02
CF317_006154-T1	Pyruvate decarboxylase	−1.138	6.87E-03
CF317_003229-T1	Putative versicolorin reductase	−1.176	2.61E-03
CF317_007960-T1	60S acidic ribosomal protein P1	−1.277	9.18E-03
CF317_006677-T1	HIT domain-containing protein	−1.770	8.90E-03

* Log2 fold change of SMG-exposed compared to unexposed proteome (*p* ≤ 0.05). ^a^Protein accession number in the *K. chersonesos* database of *ab initio* translated proteins.

In the *K. chersonesos* mutant, LSSMG triggered the upregulation of just a low number of proteins. The malate dehydrogenase CF317_003266-T1/A0A1C1CNC5_9EURO, 1,3-beta-D-glucan-UDP glucosyltransferase CF317_000614-T1/A0A438MTW5_EXOME and alpha, alpha-trehalase CF317_004588-T1/H6C927_EXODN, involved in the metabolism of starch, sucrose, and sphingolipids, were 2. 4-, 1.9-, and 1.8-fold upregulated, respectively. Conversely, proteins with decreased abundance encompassed, among others, enzymes catalyzing different steps throughout the process of glycolysis/gluconeogenesis (KEGG pathway hsa00010)—such as the putative phosphotransferase CF317_008448/W2RU25_9EURO, the phosphoglycerate kinase CF317_004751/W2RJE3_9EURO and the 2-phosphoglycerate dehydratase CF317_003513/H6BNI5_EXODN—fructose and mannose (hsa00051), pyruvate (hsa00620) and glycerolipid metabolism (hsa00561), and the pentose phosphate pathway (hsa00030) (i.e., phosphotransferase, epimerase domain-containing protein CF317_008979 and 6-phosphogluconate dehydrogenase, decarboxylating CF317_005481). Further, the CF317_007096/A0A0U1M0E3_TALIS 6-phosphofructo-2-kinase, acting as an activator of the glycolysis/gluconeogenesis pathway (Raben and Wolfgang, 2019), was the most decreased protein in the GO category of carbohydrate and lipid metabolism, with a 3-fold downregulation.

Increased levels of proteins with a role in the response to stress and chemicals (Table 2) were observed in the exposed secretomes for the plasma membrane protein CF317_000051-T1/A0A178DA36_9EURO with reported phosphatase activity and the guanine nucleotide-binding protein subunit beta CF317_007665-T1/A0A0D2CV56_9EURO with a role in signal transduction, among others. However, as already observed for the whole-cell proteome, the majority of proteins involved in stress response showed decreased abundance upon exposure to LSSMG. The DNA-repair protein histone H2A (CF317_001633-T1), upregulated in the wild type secretome, was at least twofold downregulated in the mutant, together with the nucleoside diphosphate kinase CF317_007147-T1/A0A178C9G0_9EURO. The latter, ubiquitous enzyme involved in nucleotides biosynthetic process (Janin and Deville-Bonne, 2002), has been shown to participate in the metabolism of selective drugs like Isoniazid and Fluorouracil (KEGG map00983) in *Homo sapiens*. The same was observed for the S-formylglutathione hydrolase, whose activity is mainly linked to methane metabolism and the recycling of glutathione in cell detoxification pathways (Haslam et al., 2002; Yurimoto et al., 2003). Similarly, the thioredoxin domain-containing protein CF317_000980-T1 and the 6-phosphogluconate dehydrogenase, decarboxylating CF317_005481-T1/A0A0N0NLG1_9EURO, both involved in cell redox homeostasis and glutathione metabolism (Li et al., 2019), showed an around twofold downregulation.

Also in the categories of transcription, translation, and ribosome biogenesis, downregulated proteins outnumbered the upregulated ones (Table 2). The most increased protein (almost twofold) was the putative D-amino-acid oxidase (DAO) domain-containing protein CF317_008030-T1/A0A0D1YYX4_9EURO, also involved in penicillin and cephalosporin biosynthesis (KEGG 00311). Elongation factor EF-1 beta and gamma subunit (CF317_008933-T1 and CF317_000379-T1), homologs of *Exophiala dermatitidis* CBS 525.76 H6BVG8_EXODN and H6BY84_EXODN proteins and involved in purine metabolism (KEGG 00230), were detected in the mutant LSSMG-exposed secretome at levels at least 3.5-fold lower than that of the unexposed ones. Further, four ribosomal proteins—i.e., 40S ribosomal protein S20 and S24 (CF317_001861-T1 and CF317_004501-T1); 60S ribosomal protein P1 and L7 (CF317_007960-T1 and CF317_005516-T1)—showed decreased abundance.

Additionally downregulated in response to LSSMG were the uncharacterized HIT domain-containing protein CF317_006677-T1 (over 3-fold regulated) and the putative versicolorin reductase CF317_003229-T1 (over 2-fold regulated), the latter known to mediate aflatoxins biosynthetic processes (Nakamura et al., 2011). Modulation was also observed in proteins involved in transport such as the above-mentioned 1,3-beta-D-glucan-UDP glucosyltransferase CF317_000614-T1 with a role on starch and sucrose metabolism, the endoplasmic reticulum transmembrane protein CF317_004472-T1, and the inorganic phosphate transport protein PHO88 CF317_006347-T1, which all appeared to be upregulated. Conversely, the transmembrane transporter “putative inorganic phosphate transporter C8E4.01c” CF317_002531-T1, the uncharacterized NTF2 domain-containing protein CF317_007808-T1, and the glycolytic enzyme phosphotransferase CF317_008448-T1 were detected as downregulated.

### *Knufia chersonesos* Wt and Mut Exhibit Opposite Regulation of Several Differentially Expressed Proteins

The comparative analysis of all examined samples to a reference sample—a pool of all established experimental conditions—revealed qualitative and quantitative differences between *K. chersonesos* Wt and Mut at the proteome and secretome level. This was especially evident when examining the top 10 differentially regulated proteins i.e., the 10 most up- and downregulated proteins at each experimental condition. A number of these proteins were found to undergo opposite modulation in the two strains, mainly in the whole-cell proteome. Distribution of the top differentially regulated whole-cell proteins is summarized in Supplementary Table 8: out of 41 different proteins, 6 were found to be top regulated in wild type and mutant under both normal gravity and microgravity condition, 10 only in the Wt and 10 exclusively in the Mut. Around 25% represented ribosomal proteins; 10% were proteins whose identity or function could not be elucidated after homology search; and the remaining proteins were involved in RNA binding, transcription, translation, transport, and carbohydrate metabolism. Out of the 49 top differentially regulated proteins from the secreted fraction, 2 were found in both strains at all experimental conditions, 17 only in the Wt and 17 only in the Mut (Supplementary Table 9). Similar to what was observed for the proteome, 26% of the detected proteins were ribosomal, followed by enzymes involved in carbohydrate metabolism (18%)—secreted esterases and lipases included—and in transport (10%).

Altogether, the eight top differentially regulated proteins common to wild type and mutant in both normal gravity and microgravity—i.e., the large subunit (LSU) ribosomal proteins L28 (CF317_000935-T1), L32 (CF317_002712-T1), L35 (CF317_001879-T1) and L36 (CF317_007709-T1), the small subunit (SSU) ribosomal protein S30 (CF317_005321-T1), the U1 small nuclear ribonucleoprotein C (CF317_001622-T1), the murein transglycosylase (CF317_006383-T1) and the uncharacterized protein CF317_007965-T1—displayed an LSSMG-dependent regulation that also appeared to be specific to the Wt strain (Supplementary Figure 3). As shown in Supplementary Figure 3, all proteins that are upregulated in the Wt strain appear to be downregulated in the Mut strain and vice versa.

A protein-protein interaction analysis was performed to verify the occurrence of these 8 proteins in common pathways, using STRING. A 6-node network was obtained for the proteins which matched homologs (sequence homology) in the STRING database (Supplementary Figure 4) and an interaction was confirmed for ribosomal proteins S30 (homolog of *E. dermatitidis* XP_009156842.1) and L35 (homolog of XP_009155966.1). As a component of the SSU, S30 has a role in mRNAs binding and selection of cognate aminoacyl-transferase RNA (tRNA), whereas L35 is required for polymerization of the amino acids delivered by tRNAs into a polypeptide chain (Ben-Shem et al., 2011). The involvement of protein L35 in cell growth regulation and apoptosis has additionally been reported (Bommer and Stahl, 2005). The proteins’ functional link (combined score: 0.999) was supported by evidence such as the co-expression of orthologs (score 0.963) and their interaction in other organisms (experimental/biochemical data, score: 0.904). Further interactions could not be detected possibly because functional characterization of these proteins in *E. dermatitidis* is yet to be thoroughly achieved.

### *Knufia chersonesos* Wt and Mut Comparative Analysis Under Normal Gravity (1G)

A comparative analysis of wild type and mutant was performed to evaluate different responses at the proteome and secretome level under control condition, i.e., normal gravity. The analysis of the whole-cell proteome yielded a total of 100 proteins with increased and 40 proteins with decreased abundance in *K. chersonesos* Mut, compared with the wild type (fold change of ≥ ± 1.5, *p* ≤ 0.05).

As shown in Supplementary Figure 5A, “protein translation,” “organelle organization,” and “regulation of biological processes” were detected as the prevalent processes in the mutant, encompassing more than 50% of all upregulated proteins. This includes several ribosomal proteins and ribonucleoproteins. Ribosomal proteins L32 (CF317_002712), L36 (CF317_007709), S30 (CF317_005321) and L37 (CF317_004524-T1) and the small nuclear ribonucleoprotein C (CF317_001622-T1) were among the most upregulated proteins, being present in the mutant at level 8.7 to 3.6 (3.1 to 1.85 log2 fold change) higher than in the wild type (Supplementary Table 10). Increased levels were also observed for proteins involved in RNA metabolic processes and in transcription such as the U3 small nucleolar RNA-associated protein 11 (A0A0D2DFT3_9EURO), the U6 snRNA-associated Sm-like protein LSm4 (CF317_002219-T1) and the pre-mRNA-splicing factor isy1 (CF317_006097-T1),—partaking in nucleolar processing of pre-18S ribosomal RNA and in pre-mRNA splicing (Zhou et al., 2014)—the transcription factors C2H2 and RfeG (CF317_009591-T1 and CF317_000265-T1) and the 2′-phosphotransferase (CF317_002731-T1). Protein modulation also affected enzymes involved in the oxidative phosphorylation, whose levels resulted to be increased in *K. chersonesos* Mut, i.e., the cytochrome c oxidase assembly factor 6 (CF317_000645-T1 and CF317_000273-T1), cytochrome b-c1 complex subunit 7 (CF317_008279-T1), NADH dehydrogenase ubiquinone 1 alpha subcomplex subunit CF317_000197-T1. Regulation of proteins involved in chemical and stress response was also observed. The histone H2A (CF317_001633-T1)—reported to have a role on DNA repair (Moore et al., 2007)—and the Nuclear transcription factor Y alpha (CF317_006017-T1)—with multiple roles in development and stress response (Zhao et al., 2017)—were found at levels at least 1.6-fold higher than in the melanized counterpart. Conversely, downregulated processes at normal gravity condition prevalently encompassed carbohydrate, lipid and general cellular metabolism (Supplementary Figure 5A). Decreased levels were observed for glycoside hydrolases and esterolytic enzymes including the alpha-galactosidase (CF317_004369-T1) and cutinases (CF317_007621-T1)—levels 9.7- and 7-fold lower than in the wild type—for the glucan 1,3-beta glucosidase (CF317_002004-T1; 1.69-folds decreased) and for the murein transglycosylase (CF317_004960-T1, CF317_003460-T1, CF317_006383-T1; 1.83-, 2- and 4-fold decreased), enzymes responsible for polymers and microbial degradation by cleavage at the peptidoglycan or the cell wall polysaccharides level (Lincoln et al., 1997). Additionally, proteins involved in lipid metabolic process—i.e., acetyl-CoA C-acetyltransferase (CF317_005988-T1), isocitrate lyase (CF317_004212-T1) and lipase (CF317_005885-T1)—and in protein catabolism—i.e., proteasome subunit alpha type (CF317_008861-T1), proteasome endopeptidase complex (CF317_004788-T1) and peptidase S53 domain-containing protein (CF317_005049-T1)—showed decreased levels between 1.8- and 1.5-fold.

At the secretome level, the quantitative analysis of *K. chersonesos* Mut v/s Wt under normal gravity conditions, resulted in the detection of 104 proteins with increased and 87 proteins with decreased abundance in the mutant secretome. Of these proteins, 24% have predicted extracellular localization based on the presence of a signal peptide (Supplementary Table 11). Distribution of over-represented biological process GO terms among differentially expressed proteins is displayed in Supplementary Figure 5B. Most of proteins upregulated in the mutant were involved with carbohydrate metabolism and regulation of biological process (24% of all upregulated proteins), transport (9%), response to stress (8%) and transcription (8%). A number of hydrolases required for the breakdown of β-glucan chains and other cell-wall components— i.e., carboxylic ester hydrolyses (CF317_004653-T1, CF317_008040-T1, CF317_002308-T1, CF317_005799-T1), feruloyl esterase (CF317_002949-T1), alongside glucan 1,3-beta-glucosidase (CF317_002004-T1), murein transglycosylase (CF317_006383-T1) and cutinase (CF317_007621-T1) which were downregulated in the mutant proteome—were present in the secretome at levels comprised between 1.5- and 4-folds higher than in the wild type (Supplementary Table 11). The same for mannan endo-1,6-alpha-mannosidase (CF317_001276-T1) and chitin deacetylase (CF317_003258-T1), which play multiple roles in the function of the fungal cell wall (Spreghini et al., 2003; Mouyna et al., 2020). In the category transport, the multicopper oxidase (CF317_009669-T1; 1.9-fold), the chloride channel protein (CF317_004136-T; 1.7-folds) and the nitrate/nitrite transporter (CF317_004846-T1; 1.6-fold) represented the most upregulated proteins. Increased levels of stress-response proteins were observed for the Histone H2A protein (CF317_001633-T1; 1.7-fold)—found to be overexpressed also in the mutant proteome—alongside the redox protein thioredoxin (CF317_006109-T1; 1.7-fold), catalase A (CF317_009216-T1; 1.7-fold) and the housekeeping enzyme nucleoside diphosphate kinase (CF317_007147-T1; 2.3-fold), whose involvement in the signaling pathway of oxidative stresses has been reported (Desorption et al., 2000). Normal gravity condition was also characterized by increased levels of peptidyl-prolyl *cis-trans* isomerase (CF317_005659-T1), EF-1 gamma subunit (CF317_000379-T1), and alkaline phosphatase (CF317_008227-T1; CF317_003892-T1), all involved in transcription. Conversely, decreased levels were observed in the secretome almost exclusively for proteins involved in translation and ribosome biogenesis (Supplementary Figure 5B). Out of 87 downregulated proteins, more than 50 encompassed ribosomal proteins. Of these, the most downregulated ones— the 60S ribosomal proteins L32 (CF317_006070-T1), L35 (CF317_001879-T1) and L33-A (CF317_005595-T1)—were present at levels below 5-fold than their counterparts in the wild type secretome. Other proteins were prevalently involved in stress/chemical response, e.g., plasma membrane phosphatase (CF317_000051-T1), CipC-like antibiotic response protein (CF317_003991-T1), small heat shock protein (SHSP) domain-containing protein (CF317_001628-T1). These findings pose interesting questions regarding the presence of intracellular proteins in *K. chersonesos* secretome: while it may be indicative of cell lysis during cultivation, it may also suggest that ribosomal and other non-classical secretory proteins are found in the culture supernatant due to EVs-mediated secretion (Sun and Su, 2019). The co-isolation of these and classical secretory proteins during sample preparation can occur if no prior separation of the vesicle containing fraction from the secretome, is performed (Vallejo et al., 2012).

## Discussion

To date, investigations into the responses of microorganisms to space and Mars-like conditions have been performed with few black fungi species; some of them involved stress simulation in ground-based facilities (Pacelli et al., 2017), while others carried out the exposure of fungal strains inside or outside the International Space Station (Onofri et al., 2012, 2019; Pacelli et al., 2016).

With only one exception (Zakharova et al., 2014)—the comparative study of 2D protein patterns under Mars-like conditions—the majority of astrobiological work carried out with black fungi has mainly focused on the analysis of ultrastructural alterations and DNA integrity. Hence, this study represents the first qualitative and quantitative proteomic characterization of a black fungus response to simulated space conditions, i.e., microgravity, which has significance to exobiology and implications to planetary protection policy. In-depth understanding of proteome-related alterations in cell physiology is crucial to gaining new insight into the evolution of extremophiles and the actual limits for life. Further, the comparative analysis of a melanin-deficient mutant and the wild type is important to evaluate the role of melanization in stress survival.

Under microgravity conditions, a development toward a more clump-like growth was observed in both *K. chersonesos* Wt and Mut and may be due to the low shear and low turbulence suspension culture environment created by LSSMG. However, morphological differences were not detected via FE-SEM in cells grown under LSSMG compared to those grown in 1G. This is not surprising, as black fungi reportedly resort to strategies to minimize efforts at both the morphological and physiological level when exposed to stress conditions (Sterflinger, 2006). Microcolonial growth and the switch among different growth forms—i.e., budding and hyphae formation—ensure that these organisms have a lifestyle versatility to cope with a variety of stress in their natural habitats (Sterflinger et al., 1999; Gostinčar et al., 2011). Unaltered morphology upon exposure to LSSMG was previously described in filamentous fungi in both suspension (e.g., *Aspergillus niger* and *Penicillium chrysogenum*) (Sathishkumar et al., 2014) and agar cultures (*A. niger*, *Candida albicans*) (Yamazaki et al., 2012), albeit LSSMG-induced phenotypic changes have been to date substantiated by a higher number of studies on fungal species e.g., *Pleurotus* sp., *Candida* sp., *Cladosporium* sp., *Ulocladium* sp., *Basipetospora* sp., etc (Miyazaki et al., 2010; Searles et al., 2011; Gomoiu et al., 2013). Interestingly, the sole morphological response to microgravity detected in the present study was the early switch to hyphae formation observed in *K. chersonesos* Wt (at day 5 instead of day 7, as in normal gravity; Figure 2). This is consistent with previous reports of increased filamentous growth under LSSMG in the opportunistic fungal pathogen *C. albicans* (Altenburg et al., 2008). The observation of early hyphal development in non-pathogenic species in response to LSSMG is rather suggestive of biofilm formation as a strategy for enhanced resistance to stress and for the forage for nutrients (Searles et al., 2011). Both LSSMG-cultured *K. chersonesos* Mut and their 1G controls showed extensive cell self-aggregation, i.e., cell clumping and occasional filamentation that are characteristics of black fungi (Nai et al., 2013). Significant variations were also not found in the total cell number, and cell size was not affected by microgravity in both strains (Supplementary Tables 1, 2), unlike what has been documented by studies on a variety of bacteria (Huang et al., 2018) and yeasts (Crabbé et al., 2013).

The results of proteomics analysis revealed that exposure to LSSMG altered the proteome and secretome of both *K. chersonesos* Wt and Mut when compared to the 1G counterparts, having an impact on different pathways. Interestingly, the mutant response mainly involved protein downregulation, which might suggest a general slowing of the metabolic rate (Figure 4B). In contrast, more subtle rearrangements in the protein repertoire were observed in the wild type, especially in the secreted fraction, which possibly reflect a fine-tuning of the regulation of protein expression. Regardless, both strains showed increased abundance of proteins involved in carbohydrate metabolism, especially at the whole-cell proteome level (Figure 5A). Glycolysis/gluconeogenesis and pyruvate metabolism were found to be promoted in the wild type, whereas the glyoxylate shunt, ancillary cycle to TCA cycle and essential for growth on two-carbon compounds (Lorenz and Fink, 2001), was upregulated in the mutant. Similar alterations in carbohydrate metabolism were previously observed in melanotic filamentous fungi exposed to simulated Mars conditions (SMC) or to ISS-conditions (Romsdahl et al., 2018, 2019; Blachowicz et al., 2019a). Here, increased abundance was also observed for several starvation-induced glycoside hydrolases with roles in nutrient acquisition from biopolymers and in the recycling of cell wall components to support cell maintenance. Starvation response was thereby suggested as crucial adaptation to space conditions, especially oligotrophy. Conversely, one characteristic of both *K. chersonesos* Wt and Mut was decreased levels of glycoside hydrolases and cell wall–degradation enzymes such as the endo-1,3(4)-beta-glucanase (CF317_0009683-T1) and the extracellular cell wall glucanase Crf1 (CF317_009779-T1). The same was observed for carbohydrate-active enzymes like cutinases and for lipases at both the proteome and the secretome level, which suggests that, under space conditions, black fungi opt for strategies different than those adopted by filamentous fungi.

GO analysis and pathways prediction further revealed upregulation of proteins involved in the biosynthesis of unsaturated fatty acids (USFA) and in the metabolism of phospho- and glycerophospholipids. This may be explained by modifications in membrane lipid composition aimed at maintaining membranes stability against stress (Xia et al., 2019). More specifically, an LSSMG-dependent increase in membrane fluidity attributable to a higher USFA/SFA ratio was previously demonstrated by studies in plain lipid membranes, various microorganisms e.g., *Escherichia coli* and plants upon exposure to microgravity (Sieber et al., 2014; Kim and Rhee, 2016; Kordyum and Chapman, 2017). Potentially, this could affect the function of membrane-integrated proteins, thereby leading to altered transporter activity. Whether the activity of uptake transporters is altered in microgravity conditions is currently unknown (Eyal and Derendorf, 2019). However, similarly to what was observed in other fungi e.g., *C. albicans* (Crabbé et al., 2013) and bacteria e.g., *Bacillus subtilis* (Morrison et al., 2019) in space, *K. chersonesos* Wt and Mut ion-channels and integral membrane proteins were found to be upregulated. The increased abundance of specific transporters generally maximizes the uptake of nutrients (e.g., phosphate and nitrogen). As such, it possibly represents an adaptive response to temporary starvation caused either by zones of nutrient depletion developing around the colony or by the partial loss of contact of the cells with the culture medium, which may occur under microgravity conditions (Mazars et al., 2014; Senatore et al., 2018). Other types of transporters—i.e., ammonium transporters and permeases for transmembrane amino acid transport—could provide a link between nitrogen assimilation and proteins synthesis (Martzivanou et al., 2006). Indeed, upregulation of proteins involved in amino acid biosynthesis and metabolic processes was recorded under LSSMG conditions, but a higher number of these proteins was found to be decreased, especially in the mutant proteome.

Decreases in the levels of structural ribosomal proteins involved in cytoplasmic translation (i.e., L23a, L37a, and L34) and of the poly(A)polymerase, as well as the increase in RNA degrading ribonuclease T1, were additionally detected in microgravity. Together, the regulation of these proteins may suggest that protein translation via ribosomal translational machinery is reduced upon exposure to LSSMG, a phenomenon that has been indicated as a widespread response to microgravity, space, and Mars-like conditions not only in fungi (Sheehan et al., 2007; Willaert, 2013; Feger et al., 2016; Kamal et al., 2018, 2019; Blachowicz et al., 2019a). Remarkably, in our study, a decrease in translation and ribosome biogenesis was only observed in the wild type whole-cell proteome and, to a minor extent, in the mutant secretome (Figure 5B). However, ribosomes are very dynamic organelles and additional tests will be needed to confirm that the 2-fold regulation of the above-mentioned proteins is actually indicative of reduced protein synthesis under microgravity.

In a similar fashion, proteins taking part in functional organization of cell organelles and cytoskeleton—for which a wide spectrum of microgravity-dependent changes has been reported (Zhang et al., 2015)—showed opposite regulation in the wild type and the mutant. The same was observed in the secretome for the DNA-repair Histone H2A and the scytalone dehydratase, the latter involved in the biosynthesis of DNH melanin from endogenous substrate (Eisenman and Casadevall, 2012), which were upregulated in the wild type and downregulated in the mutant. This is suggestive of increased measures for cell protection and is in line with the fact that melanin pigmentation and enhanced melanin synthesis is most often a feature of fungi living on space stations (Dadachova and Casadevall, 2008; De Middeleer et al., 2019). Further, versicolorin reductase (CF317_003229-T1), a protein mediating aflatoxins biosynthetic processes, was also decreased only in the mutant. Although production of aflatoxins in *K. chersonesos* has hitherto not been reported, this finding can be indicative of reduced production of allergenic or toxic metabolites (e.g., polyketides). Also in the mutant, the upregulation of hydrolytic enzymes such as the alpha, alpha-trehalase suggests a recourse to store carbohydrates as a carbon source. By contrast, production of compounds that increase the osmotolerancy (e.g., trehalose) represents a quite common protective measure implemented by microorganisms under microgravity and other types of stress (Willaert, 2013).

Opposite protein regulation was especially evident when examining the top 10 proteins differentially regulated in both strains at all experimental conditions, which included several ribosomal proteins (Supplementary Figure 3). These discrepancies suggest that strategies to cope with suboptimal conditions of growth may be strain-specific and that diverse rearrangements of proteome repertoire are possibly related to the presence or absence of melanin in the cell wall. Indeed, differences between wild type and mutant proteomic profiles were even observed at normal gravity condition. Proteins involved in translation, transcription and RNA metabolic process were significantly more upregulated in the mutant proteome than in the wild type (up to 9-fold, as in the case of ribosomal proteins L32, L36, S30 and L37), whereas the mutant secretome showed increased abundance of a number of hydrolases required for the breakdown of biopolymers and cell wall components (Supplementary Figure 5).

A number of proteins differentially expressed under simulated microgravity were involved in stress response, including glutamate dehydrogenase (GDH; CF317_004755-T1) and nitric oxide dioxygenase (NOD; CF317_006388-T1), over-represented in the proteome of both fungi, and chemical stress component proteins with proteolytic and phosphatase activity. While its role as a stress-responsive enzyme was speculated for GDH (i.e., ROS) (Skopelitis et al., 2006), NOD participates in mechanisms of detoxification of nitric oxide, a gas with multiple roles in cellular metabolism ranging from defense to signaling. The observation of peptidases, which were enriched in *K. chersonesos* Wt proteome under exposure to LSSMG, may instead suggest the removal of damaged proteins to enhance cellular fitness and maximize survival (Bonham-Carter et al., 2013; Zhang et al., 2015). In this respect, it should be noted that a role of altered gravity in the breakdown of protein structures has been previously reported (Trotter et al., 2015). However, a major part of detected stress response proteins involved in cell redox homeostasis, glutathione metabolism and recycling and osmotic stress defense—i.e., catalases, glutathione reductases, 3-phytases and s-formylglutathione hydrolases—appeared to be decreased under exposure to LSSMG. As previously suggested in plants (Zhang et al., 2015), downregulation of general stress response proteins under microgravity might indicate an impaired activation of the defense response components. However, decreased levels of common stress proteins and lack of a heat shock response (HSP) represent a key component of black fungi response to a variety of suboptimal conditions of growth and have been attributed to an energy-saving strategy that relies on a fine-tuning regulation of protein abundance (Tesei et al., 2012, 2015; Zakharova et al., 2013, 2014; Blasi et al., 2017).

One further interesting aspect was the high number of proteins traditionally recognized as cytoplasmic in *K. chersonesos* secretome, especially in the mutant (i.e., 6% of total secreted proteins with predicted signal peptide v/s 25% in the wild type; 20% of regulated secreted proteins had signal peptides in both strains). Such a discrepancy between wild type and mutant was also reported in a study of *K. chersonesos* secretome aimed at the screening for novel polyesterases (Tesei et al., 2020), hence it seems to suggest that a low number of extracellular proteins may be a peculiarity of the mutant secretome. The presence of cytoplasmic proteins in the culture supernatant may indicate that the mutant is more prone to cell lysis than the wild type (Miura and Ueda, 2018). However, given that proteins with no signal peptide (leaderless) can also be found in the secreted fraction as a result of non-classical protein secretion, e.g., via EVs-mediated pathways (Vallejo et al., 2012; Sun and Su, 2019), it may as well indicate co-isolation of vesicle proteins and classical secretory proteins during sample preparation procedures. These proteins (e.g., ribosomal proteins, proteins involved in carbohydrate metabolism, response to stress, signaling, cell division and transport, etc.), for which vesicular transport might be the only route of extracellular delivery, are generally predicted as non-secretory (Bleackley et al., 2019).

Together, the aspects of protein modulation observed in *K. chersonesos* Wt and Mut suggest that the basic energy metabolism was upregulated in the Wt strain. Here, rearrangements of the protein repertoire resembled the classic response to microgravity, but with no evidence of significant activation of stress components or starvation response. The mutant mostly engaged in protein downregulation, without affecting their cell growth and survivability. This study therefore indicates the ability of black fungi to cope with microgravity conditions and suggests that cell wall melanization may ultimately influence the metabolic response to microgravity. Point mutation will be needed to confirm whether mutagenesis played a role in protein downregulation in *K. chersonesos* Mut. To further our understanding about the impact of microgravity on ribosome biogenesis and protein translation, future work shall involve proteomics workflows such as radio-labeled protein expression assays.

## Data Availability Statement

WGS data for *K. chersonesos* MA5789 are available in NCBI GenBank (GCA_002319055.1), under BioSample accession number SAMN07326825 and BioProject accession number PRJNA393270. The proteomics datasets generated during the current study are accessible through the ProteomeXchange Consortium (http://proteomecentral.proteomexchange.org) via PRIDE with the data set identifier PXD022898.

## Author Contributions

DT designed the study, drafted the manuscript, carried out the LSSMG exposure experiments, and performed data analysis and interpretation. AC and MK conducted protein sample processing, LC/MS analyses, and proteome data processing. JS annotated the genome of *K. chersonesos* MA5789 for proteome analysis. GBMM conducted FE-SEM analysis. KV designed the study and critically reviewed the manuscript. DT, KS, and KV contributed to funding acquisition. All authors contributed to the review, editing, and approval of the final version of the manuscript.

## Conflict of Interest

The authors declare that the research was conducted in the absence of any commercial or financial relationships that could be construed as a potential conflict of interest.

## Footnotes

1 http://geneontology.org

2 http://www.uniprot.org/blast

3 http://busca.biocomp.unibo.it
