# Supplementary material for: Effects of Simulated Microgravity on the Proteome and Secretome of the Polyextremotolerant Black Fungus Knufia chersonesos
Source: Front Genet. 2021 Mar 18;12:638708. doi: 10.3389/fgene.2021.638708 (PMC8012687; doi:10.3389/fgene.2021.638708)
Supplement: Supplementary file 1 [file Data_Sheet_1.docx]

Supplementary Material

**Supplementary Table 1_**Cell concentration in the LSSMG-exposed and 1G-exposed samples (control) as measured via hemocytometer count at four different time points in the experiment: start (seed, Day 0), acceleration phase (in between lag and exponential phases, Day 3), exponential phase (5- to 6-days-old) and at the end of the test (stationary phase, Day 7). Survivability at the end of the LSSMG exposure was assessed by enumeration of colony forming units (CFU) using ImageJ software (Schneider et al., 2012). Values are means of two biological replicates.

| **Strain** | **Seed** | **Day 3** | **Day 5** | **Day 7** | **Survivability** |
| --- | --- | --- | --- | --- | --- |
| *K. chersonesos* Wt LSSMG-exposed | 2 x 10^5^ cells/mL | 4.7 × 10^6^ cell/mL | 5.2 × 10^6^ cell/mL | 5.7 × 10^6^ cell/mL | 2.8 × 10^6^ cell/mL |
| *K. chersonesos* Wt unexposed |  | 4.4 × 10^6^ cell/mL | 4.8 × 10^6^ cell/mL | 8.4 × 10^6^ cell/mL | 2.6 × 10^6^ cell/mL |
| *K. chersonesos* Mut LSSMG-exposed |  | 2 × 10^6^ cell/mL | 2.8 × 10^6^ cell/mL | 6.1 × 10^6^ cell/mL | 2.5 × 10^6^ cell/mL |
| *K. chersonesos* Mut unexposed |  | 3.1 × 10^6^ cell/mL | 3.3 × 10^6^ cell/mL | 7.3 × 10^6^ cell/mL | 2.3 × 10^6^ cell/mL |

**Supplementary Table 2_**Cell size of LSSMG-exposed and 1G-exposed (control) *K. chersonesos* Wt and Mut, recorded at 4 timepoints of the 7-days long cultivation. Each value represents the average of ten measurements. The p-values are the result of a non-parametric Mann-Whitney U-test comparing cell size of control (1G-exposed) and treatment (LSSMG-exposed) at each individual time point. The null hypothesis of the test states that the two groups which are compared have the same mean size. In all the cases where the p-value of the test is not smaller than the significance level of 0.05, we fail to reject the null hypothesis. We therefore do not have sufficient evidence to say that the true mean size is different between the two groups.

| **Strain** | **Day 1** | | **Day 3** | | **Day 5** | | **Day 7** | |
| --- | --- | --- | --- | --- | --- | --- | --- | --- |
|  | Cell size (μm) | | Cell size (μm) | | Cell size (μm) | | Cell size (μm) | |
|  | width | length | width | length | width | length | width | length |
| *K. chersonesos* Wt LSSMG-exposed | 4.6±0.2 | 5.4±0.9 | 3.3±0.7* | 4.5±1.6 | 3.9±0.7 | 4.7±0.9 | 4.7±0.8 | 5.4±1 |
| *K. chersonesos* Wt 1G-exposed | 3.5±0.4 | 3.9±0.6 | 4.6±1.5* | 5.4±2.2 | 4.5±1.6 | 5.1±1.6 | 4.8±0.7 | 5.2±1.4 |
| *K. chersonesos* Mut LSSMG-exposed | 3.6±0.3 | 4.7±0.7 | 3.8±0.5 | 4.9±0.5 | 4.1±0.3 | 4.9±0.7 | 4±1 | 4.6±1.1 |
| *K. chersonesos* Mut 1G-exposed | 3.4±0.5 | 3.7±0.4 | 4.7±0.5 | 3.9±0.4 | 5.1±0.7 | 5.1±0.8 | 3.8±1.1 | 4.5±0.4 |

Mann-Whitney U-test

*K. chersonesos* Mut cell length day 1 1G-exposed v/s LSSMG-exposed, p-value: 0.14

*K. chersonesos* Mut cell width day 1 1G-exposed v/s LSSMG-exposed, p-value: 0.26

*K. chersonesos* Mut cell length day 3 1G-exposed v/s LSSMG-exposed, p-value: 0.57

*K. chersonesos* Mut cell width day 3 1G-exposed v/s LSSMG-exposed, p-value: 0.57

*K. chersonesos* Mut cell length day 5 1G-exposed v/s LSSMG-exposed, p-value: 0.73

*K. chersonesos* Mut cell width day 5 1G-exposed v/s LSSMG-exposed, p-value: 0.14

*K. chersonesos* Mut cell length day 7 1G-exposed v/s LSSMG-exposed, p-value: 0.28

*K. chersonesos* Mut cell width day 7 1G-exposed v/s LSSMG-exposed, p-value: 0.60

*K. chersonesos* Wt cell length day 1 1G-exposed v/s LSSMG-exposed, p-value: 0.33

*K. chersonesos* Wt cell width day 1 1G-exposed v/s LSSMG-exposed, p-value: 0.33

*K. chersonesos* Wt cell length day 3 1G-exposed v/s LSSMG-exposed, p-value: 0.11

*K. chersonesos* Wt cell width day 3 1G-exposed v/s LSSMG-exposed, p-value: 0.02*

*K. chersonesos* Wt cell length day 5 1G-exposed v/s LSSMG-exposed, p-value: 0.43

*K. chersonesos* Wt cell width day 5 1G-exposed v/s LSSMG-exposed, p-value: 0.26

*K. chersonesos*Wt cell length day 7 1G-exposed v/s LSSMG-exposed, p-value: 0.94

*K. chersonesos* Wt cell width day 7 1G-exposed v/s LSSMG-exposed, p-value: 0.79

**Supplementary Figure 1_**Confocal microscopy images of *K. chersonesos* Wt and Mut at the beginning (day 1) and end (day 7) of exposure to LSSMG or 1G (control). Viability was analyzed by LIVE/DEAD staining kit FungaLight and cell wall integrity by wheat germ agglutinin (WGA). Cells with intact membranes stain fluorescent green, cells with damaged membranes stain fluorescent red, cell wall stains fluorescent blue. Lower staining intensity in the wild type is related to the presence of melanin in the cell wall.

**
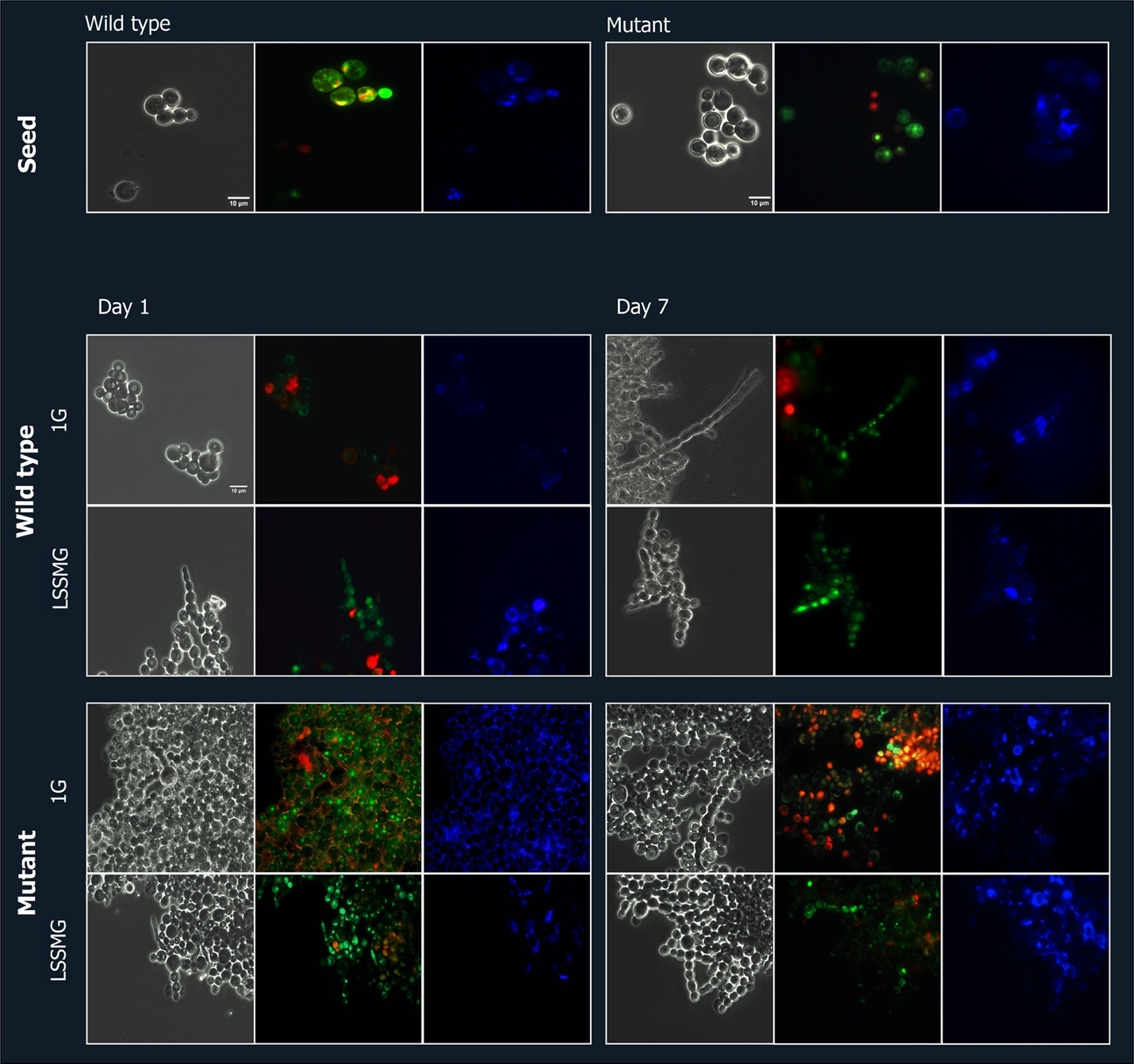
**

**Supplementary Figure 2_**Distribution of the GO categories with the highest number of associated proteins among the 8 different experimental conditions in *K. chersonesos* Wt and Mut whole-cell proteome (A) and secretome (B). The bar graphs were created using OmicsBox v. 1.4.11 (<https://www.biobam.com>). BP: biological process; MF: molecular function; CC: cellular component.

**
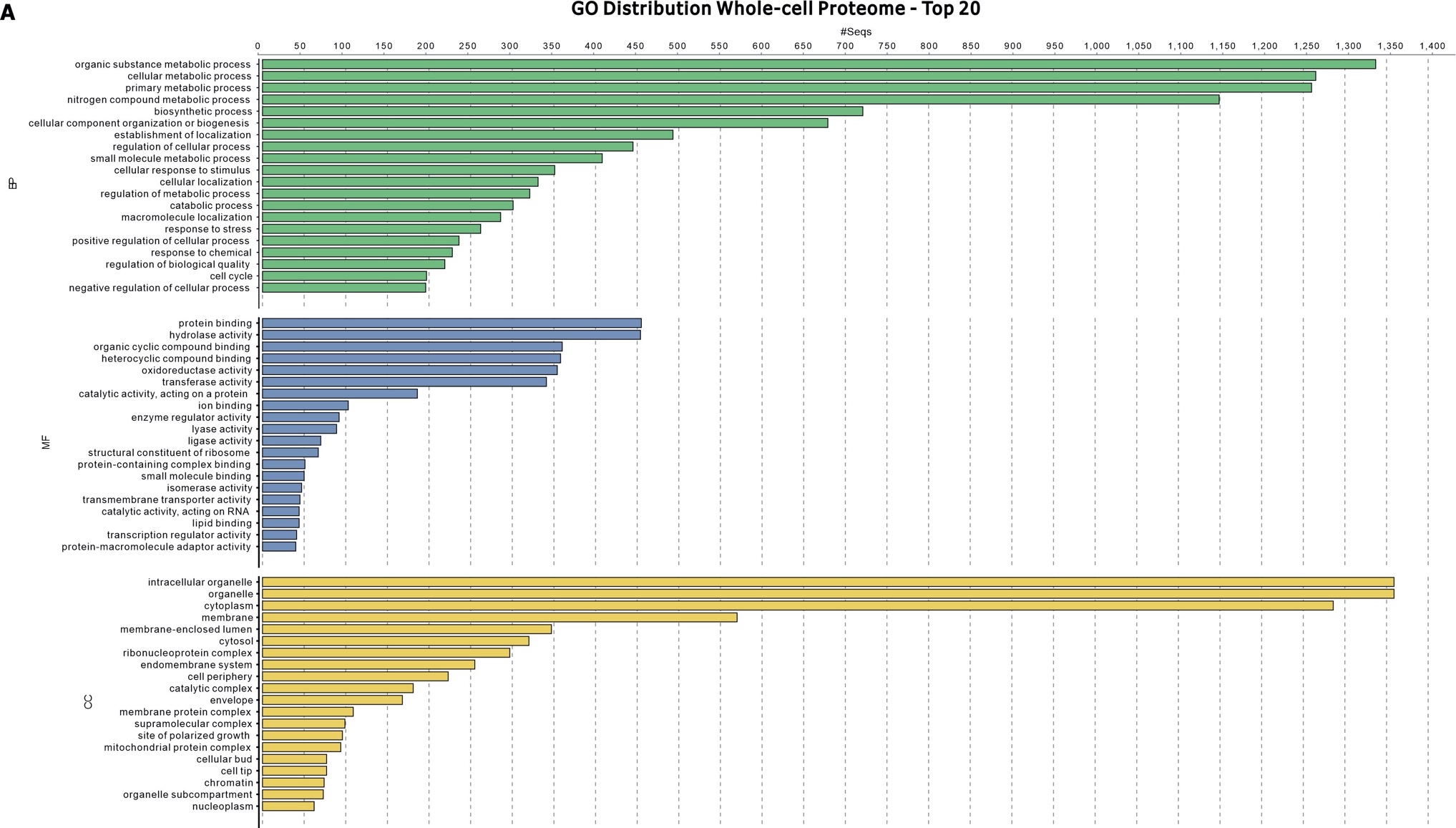
**

**
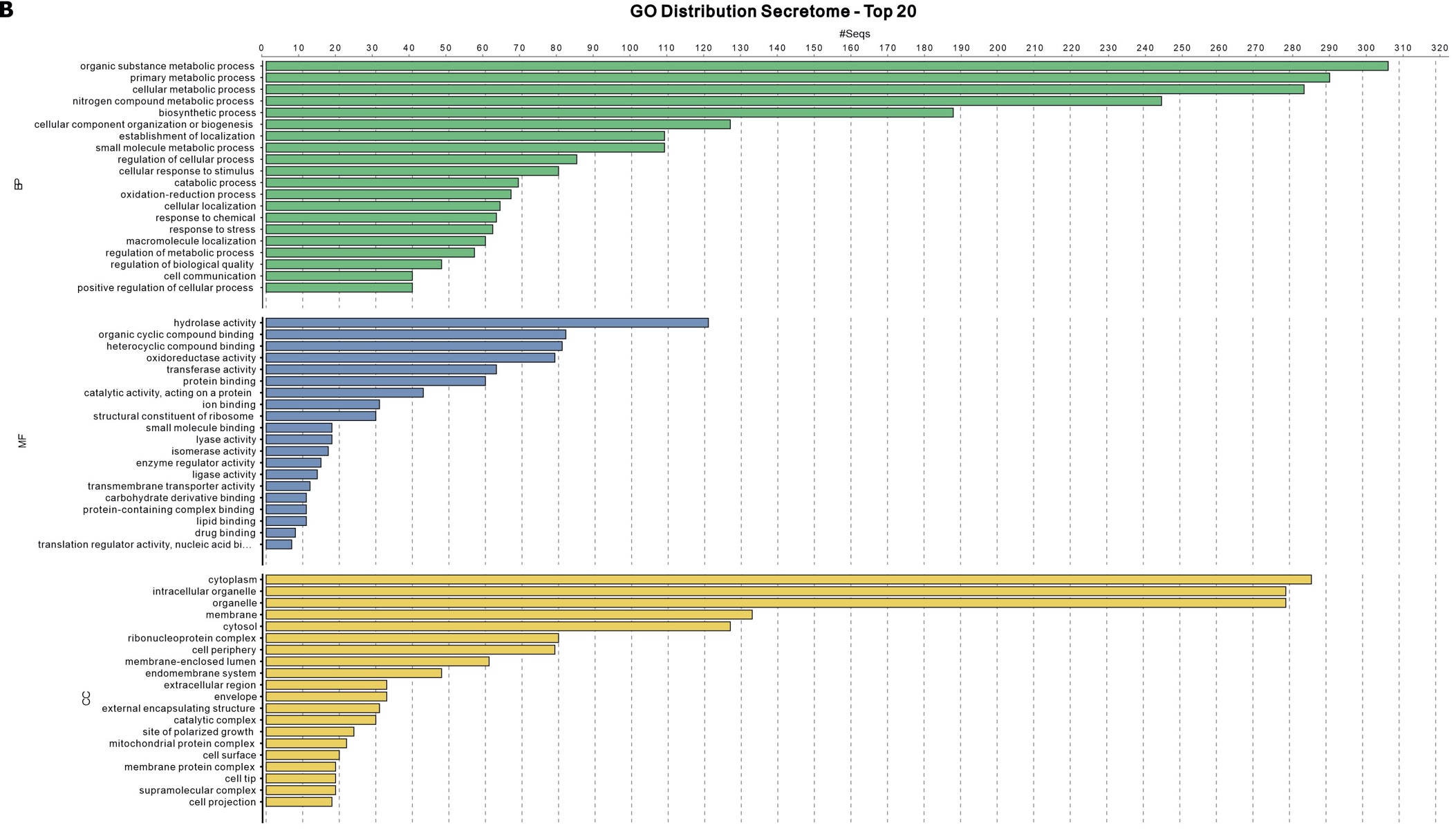
**

**Supplementary Table 3_** Homologues of the 25 proteins unique to *K. chersonesos* Wt and Mut secretome (BLASTP algorithm). Only the matching proteins with known identity (when available) and the highest max score are displayed.

| ***Protein* accession No. ^a^** | **UniProtKb accession**  **No.** | **Protein name** | **Max**  **Score** | **Identity (%)** | | **Expected**  **value** | **Predicted Localization** | **BUSCA Score** | **KEGG**  **Enzymes**  **code** | |
| --- | --- | --- | --- | --- | --- | --- | --- | --- | --- | --- |
| CF317_000257-T1 | A0A3M7NIC9_9EURO | **Ydc2-catalyt domain-containing protein** *(Chaetothyriales sp. CBS 132003)* | 295 | 35.2 | 4.2E-28 | | Extracellular space | 0.99 | - | |
| CF317_001898-T1 | A0A0N1P0W6_9EURO | Allergen Asp f 7 *(Phialophora attinorum)* | 380 | 55.9 | 6E-43 | | Extracellular space | 1 | - | |
| CF317_002120-T1 | A0A1B9I857_9TREE | Ribosomal protein L17 *(Kwoniella pini CBS 10737)* | 95 | 26.6 | 1.3E-2 | | Cytoplasm | 0.7 | - | |
| CF317_002949-T1 | A0A177BY00_9PLEO | Feruloyl esterase *(Paraphaeosphaeria sporulosa)* | 730 | 54.5 | | 1E-93 | Extracellular space | 0.99 | | EC:3.1.1.73; EC:3.1.1.1 |
| CF317_003202-T1 | G3JMK8_CORMM | Concanavalin A-like lectin/glucanase *(Cordyceps militaris* CM01*)* | 1.038 | 56 | 1.6E-138 | | Cytoplasm | 0.7 | - | |
| CF317_003654-T1 | H6BW33_EXODN | Uncharacterized protein *(Exophiala dermatitidis* CBS 525.76) | 587 | 64.8 | 1.8E-75 | | Extracellular space | 0.99 | - | |
| CF317_003764-T1 | A0A0D2DVN3_9EURO | Uncharacterized protein *(Exophiala oligosperma)* | 283 | 45.6 | 1.2E-30 | | Extracellular space | 0.99 |  | |
| CF317_003942-T1 | H6BWR3_EXODN | **Chitinase** *(Exophiala dermatitidis* CBS 525.76) | 1,166 | 34.7 | 5.2E-144 | | Anchored component of plasma membrane | 1 | EC:3.2.1.14 | |
| CF317_003956-T1 | H6BVV8_EXODN | Uncharacterized protein *(Exophiala dermatitidis* CBS 525.76) | 431 | 50.5 | 1.1E-51 | | Extracellular space | 1 | - | |
| CF317_004417-T1 | A0A178D5J7_9EURO | **Zn(2)-C6 fungal-type domain-containing protein** *(Fonsecaea nubica)* | 1,675 | 44.3 | 0.0 | | Plasma membrane | 0.71 | - | |
| CF317_004588-T1* | H6C927_EXODN | Alpha,alpha-trehalase *(Exophiala dermatitidis* CBS 525.76) | 3,189 | 58.0 | 0.0 | | Extracellular space | 0.94 | EC:3.2.1.28 | |
| CF317_004761-T1 | A0A0B4FHY0_METBS | Lysozyme-like domain protein *(Metarhizium brunneum* ARSEF 3297) | 430 | 52.2 | 8.1E-51 | | Extracellular space | 0.99 | - | |
| CF317_004776-T1 | A0A0D2C3R4_9EURO | **Glycosidase** *(Exophiala xenobiotica)* | 1,250 | 65.0 | 2.3E-168 | | Extracellular space | 0.98 | EC:3.2.1.73 | |
| CF317_004846-T1 | A0A0D2JAB3_9EURO | **Nitrate/nitrite transporter** *(Rhinocladiella mackenziei CBS* 650.93*)* | 1,820 | 66.0 | 0.0 | | Plasma membrane | 0.79 | - | |
| CF317_005782-T1 | A0A0G2E357_9EURO | Putative mfs phospholipid transporter *(Phaeomoniella chlamydospora)* | 1,831 | 73.5 | 0.0 | | Plasma membrane | 0.78 | - | |
| CF317_006179-T1 | W9YVA3_9EURO | **Glyco_hydro_cc domain-containing protein** *(Capronia epimyces CBS 606.96)* | 777 | 38.2 | 3.2E-96 | | Extracellular space | 0.92 | - | |
| CF317_006206-T1 | A0A3D8RPE2_9HELO | ER-derived vesicle protein ERV15 *(Coleophoma crateriformis)* | 699 | 95.7 | 6.4E-94 | | Endomembrane system | 0.92 | - | |
| CF317_006800-T1 | A0A0D2D5R0_9EURO | **Aa_trans domain-containing protein** *(Exophiala oligosperma)* | 2,325 | 72 | 0.0 | | Plasma membrane | 0.86 | - | |
| CF317_007158-T1 | W9W7C9_9EURO | Aquaglyceroporin like protein *(Cladophialophora yegresii* CBS 114405) | 1,475 | 78.1 | 0.0 | | Plasma membrane | 0.69 | - | |
| CF317_007259-T1 | A0A1Y1VG99_9FUNG | **Protein kinase domain-containing protein** *(Piromyces finnis)* | 140 | 35.8 | 1.1E-8 | | Extracellular space | 1 | - | |
| CF317_007396-T1 | A0A2T6ZR25_TUBBO | **Autophagy-related protein** *(Tuber borchii)* | 590 | 95.8 | 7.3E-78 | | Cytoplasm | 0.7 | - | |
| CF317_007887-T1 | A0A0D2K5L7_9EURO | Pep2-like protein (Fonsecaea multimorphosa CBS 102226*)* | 430 | 82.4 | 5.1E-52 | | Organelle membrane | 0.88 | - | |
| CF317_008029-T1 | A0A0D2GM27_9EURO | **DUF1996 domain-containing protein** *(Phialophora americana)* | 1,326 | 57.9 | 2.4E-175 | | Extracellular space | 0.98 | - | |
| CF317_009111-T1 | A0A1C1CJS1_9EURO | Endo-1,3(4)-beta-glucanase *(Cladophialophora carrionii)* | 1,191 | 52.1 | 1.9E-156 | | Extracellular space | 0.99 | - | |
| CF317_009382-T1 | A0A072P5M7_9EURO | Murein transglycosylase *(Exophiala aquamarina* CBS 119918) | 125 | 34.2 | 1.5E-5 | | Cytoplasm | 0.7 | - | |

^a^ Protein accession number in the *K. chersonesos* database of *ab initio* translated proteins

**Supplementary Table 4_**Homologues of differentially abundant proteins in LSSMG-exposed *K. chersonesos* Wt whole-cell proteome (BLASTP algorithm). Only the matching proteins with known identity (when available) and the highest max score are displayed.

| ***Protein* accession No. ^a^** | **UniProtKb accession**  **No.** | **Protein name** | **Max**  **Score** | | **Identity**  **(%)** | **Expected**  **value** | **Predicted Localization** | **BUSCA Score** | | | **KEGG Enzymes code** | | **LSSMG/**  **Control**  **FC ^b^** | | **p-value** |  |
| --- | --- | --- | --- | --- | --- | --- | --- | --- | --- | --- | --- | --- | --- | --- | --- | --- |
| CF317_007479-T1 | A0A0F4GNL1_9PEZI | Carbonic anhydrase like protein *(Zymoseptoria brevis)* | 956 | | 60.8 | 4.4E-128 | Extracellular space | | | 0.99 | EC:4.2.1.1 | | 2.066 | 8.70E-02 | | |
| CF317_001470-T1 | A0A1Y2U7H4_9PEZI | Alpha/beta-hydrolase (*Hypoxylon sp. EC38)* | 2.660 | | 69 | 0.0 | Extracellular space | | | 0.97 | - | | 1.9891 | 3.93E-01 | | |
| CF317_004755-T1 | A0A0D2EW74_9EURO | **Glutamate dehydrogenase** *(Exophiala xenobiotica)* | 2.044 | | 85.3 | 0.0 | Cytoplasm | | | 0.96 | EC:1.4.1.3; EC:1.4.1.4 | | 1.7627 | 3.54E-01 | | |
| CF317_004246-T1 | A0A0D2IWB8_9EURO | **Phosphate transporter** *(Rhinocladiella mackenziei* CBS 650.93*)* | 2.389 | | 78.4 | 0.0 | Plasma membrane | | | 0.95 | - | | 1.3345 | 4.85E-01 | | |
| CF317_008227-T1 | A0A0D2BGD8_9EURO | **Alkaline phosphatase** (*Exophiala spinifera)* | 2.767 | | 81.7 | 0.0 | Extracellular space | | | 1 | - | | 1.2107 | 4.76E-01 | | |
| CF317_004099-T1 | F9XH01_ZYMTI | Deacylase-like protein (Zymoseptoria tritici CBS 115943) | 1.287 | | 66.0 | 8.3E-176 | Extracellular space | | | 0.99 | EC:3.5.1.83; EC:3.5.1.81 | | 1.2069 | 1.48E-02 | | |
| CF317_003648-T1 | A0A0D2CPX4_9EURO | Glycerophosphoryl diester phosphodiesterase **domain-containing protein** *(Exophiala xenobiotica)* | 1.663 | 72.6 | | 0.0 | Extracellular space | | 1 | | | - | 1.1133 | 4.79E-02 | | |
| CF317_002955-T1 | C7ZGD6_NECH7 | Phospholipase A2 (Nectria haematococca strain 77-13-4) | 360 | 49.3 | | 2.5E-41 | Extracellular space | | 0.98 | | | EC:3.1.1.1; EC:3.1.1.4 | 1.1032 | 3.26E-01 | | |
| CF317_008840-T1 | W9XS38_9EURO | **Carboxypeptidase** *(Capronia coronata CBS 617.96)* | 2.033 | 67.8 | | 0.0 | Extracellular space | | 0.98 | | | EC:3.4.17.11; EC:3.4.21; EC:3.4.16; EC:3.4.16.2; EC:3.4.16.5; EC:3.4.16.6; | 4157 | 6.46E-01 | | |
| CF317_003892-T1 | A0A0D2BGD8_9EURO | **Alkaline phosphatase** (*Exophiala spinifera)* | 2.244 | 66.2 | | 0.0 | Plasma membrane | | 0.82 | | | - | 1.0475 | 4.25E-01 | | |
| CF317_005207-T1 | W9XBV2_9EURO | Ribonuclease T1 *(*Cladophialophora psammophila CBS 110553) | 455 | 59.9 | | 3.1E-56 | Extracellular space | | 1 | | | - | 0.9813 | 3.38E-01 | | |
| CF317_008208-T1 | V9DPN8_9EURO | **GFA domain-containing protein (**Cladophialophora carrionii CBS 160.54) | 384 | 49.6 | | 2.6E-45 | Nucleus | | 1 | | | - | 0.9514 | 1.71E-01 | | |
| CF317_004810-T1 | A0A1C1CCK9_9EURO | NAD(P)-binding Rossmann-fold containing protein *(*Cladophialophora carrionii) | 1.252 | 78.5 | | 9.8E-173 | Cytoplasm | | 1 | | | - | 0.9491 | 3.48E-01 | | |
| CF317_006388-T1 | S7Z8A1_PENO1 | **Nitric oxide dioxygenase** (Penicillium oxalicum strain 114-2) | 1.018 | 46.1 | | 6.4E-133 | Cytoplasm | | 1 | | | EC:1.14.12; EC: 1.14.12.17; EC:1.5.1.30; EC:1.5.1.41 | 0.9459 | 3.16E-01 | | |
| CF317_001251-T1 | A0A1C1C701_9EURO | Protein HbrB *(Cladophialophora carrionii)* | 2.211 | 60.2 | | 0.0 | Nucleus | | 1 | | | - | 0.9412 | 2.29E-01 | | |
| CF317_008503-T1 | W9YGG3_9EURO | **Thiamine thiazole synthase** *(Capronia epimyces CBS 606.96)* | 1.393 | 81 | | 0.0 | Cytoplasm | | 1 | | | - | 0.9397 | 2.30E-01 | | |
| CF317_004702-T1 | A0A179FUL4_PURLI | Quinoprotein amine dehydrogenase beta chain-like protein *(Purpureocillium lilacinum)* | 439 | 39.6 | | 1.8E-48 | Extracellular space | | 0.77 | | | - | 0.8862 | 5.04E-01 | | |
| CF317_002486-T1 | A0A4Z1PGH2_9PEZI | Alkaline phosphatase D-related protein *(Venturia nashicola)* | 2.671 | 75.7 | | 0.0 | Extracellular space | | 0.83 | | | EC:3.1.3.2 | 0.8673 | 4.69E-01 | | |
| CF317_007988-T1 | A0A0D2CPQ3_9EURO | **VPS37 C-terminal domain-containing protein** *(Phialophora americana)* | 713 | 57.7 | | 1.51E-91 | Nucleus | | 1 | | | - | 0.8635 | 5.08E-01 | | |
| CF317_005767-T1 | A0A0N0NIW7_9EURO | Alcohol dehydrogenase 1 *(Phialophora attinorum)* | 1.690 | 91.8 | | 0.0 | Extracellular space | | 1 | | | EC:1.1.1.78; EC:1.1.1.1; EC:1.1.1.71 | 0.8542 | 9.37E-02 | | |
| CF317_007148-T1 | A0A438MVJ9_EXOME | **WW domain-containing protein** *(Exophiala mesophila)* | 618 | 43.9 | | 1.5E-75 | Cytoplasm | | 0.7 | | | - | 0.8464 | 6.09E-02 | | |
| CF317_004157-T1 | W2S3B3_9EURO | **Peptidase_M14 domain-containing protein** *(Cyphellophora europaea*CBS 101466*)* | 1.871 | 67.4 | | 0.0 | Cytoplasm | | 1 | | | EC:3.4.17; EC:3.4.17.11; EC:3.4.17.22; EC:3.4.17.10 | 0.8180 | 2.52E-01 | | |
| CF317_006579-T1 | A0A178BWA1_9EURO | **Alpha/beta-glucosidase agdC** (*Fonsecaea multimorphosa)* | 3.262 | 66.3 | | 0.0 | Anchored component of plasma membrane | | 1 | | | EC:3.2.1.20; EC:3.2.1.21; | 0.8177 | 6.11E-03 | | |
| CF317_009031-T1 | A0A1Q8RG26_9PEZI | **Carboxylic ester hydrolase** *(Colletotrichum chlorophyti)* | 2.111 | 69.3 | | 0.0 | Extracellular space | | 0.74 | | | - | 0.8024 | 4.22E-02 | | |
| CF317_004606-T1 | A0A4S9CUW4_AURPU | Uncharacterized protein *(Aureobasidium pullulans)* | 776 | 53.9 | | 1.2E-99 | Anchored component of plasma membrane | | 1 | | | - | 0.7866 | 1.52E-01 | | |
| CF317_000532-T1 | A0A0D2C8U2_9EURO | Uncharacterized protein (Exophiala xenobiotica) | 2.998 | 63.7 | | 0.0 | Nucleus | | 1 | | | - | 0.7750 | 3.78E-01 | | |
| CF317_0005675-T1 | H6C9L0_EXODN | **Mitochondrial thiamine pyrophosphate carrier 1** *(Exophiala dermatitidis* CBS 525.76) | 1.395 | 79.9 | | 0.0 | Mitochondrion | | 1 | | | - | 0.7750 | 8.05E-03 | | |
| CF317_0006944-T1 | A0A0D1ZPI5_9EURO | **Ig-like domain-containing protein** *(Cladophialophora immunda)* | 295 | 36.1 | | 4.5E-28 | Mitochondrion | | 0.96 | | | - | 0.7661 | 8.12E-02 | | |
| CF317_001330-T1 | A0A6A6JUG5_9PLEO | **Phosphate transporter** *(Westerdykella ornata)* | 2.255 | 72.1 | | 0.0 | Plasma membrane | | 0.95 | | | - | 0.7548 | 1.35E-01 | | |
| CF317_000948-T1 | A0A1C1D2R0_9EURO | 4-coumarate--CoA ligase-like 7 *(Cladophialophora carrionii)* | 2.247 | 72.8 | | 0.0 | Cytoplasm | | 1 | | | - | 0.7354 | 4.19E-01 | | |
| CF317_001709-T1 | A0A438MTR2_EXOME | **WD_REPEATS_REGION domain-containing protein** *(Exophiala mesophila)* | 3.676 | 68.5 | | 0.0 | Cytoplasm | | 0.96 | | | - | 0.7277 | 5.37E-01 | | |
| CF317_008998-T1 | A0A6A6HAZ9_9PEZI | Putative fumarylacetoacetate hydrolase *(Viridothelium virens)* | 3.578 | 68 | | 0.0 | Cytoplasm | | 1 | | | EC:3.4.13; EC:3.4.13.18; EC:3.4.13.21 | 0.7082 | 7.54E-02 | | |
| CF317_007254-T1 | A0A2S6CHT4_9PEZI | **Metallophos domain-containing protein** (*Cercospora berteroae)* | 2.542 | 75.2 | | 0.0 | Extracellular space | | 0.93 | | | - | 0.6949 | 4.87E-01 | | |
| CF317_007527-T1 | A0A0D2F3V2_9EURO | **CBM21 domain-containing protein** *(Phialophora americana)* | 2.042 | 57.2 | | 0.0 | Cytoplasm | | 0.7 | | | - | 0.6921 | 3.33E-01 | | |
| CF317_008598-T1 | A0A0D2FAU6_9EURO | **1,3-beta-glucanosyltransferase** *(Phialophora americana)* | 1.578 | 61.6 | | 0.0 | Anchored component of plasma membrane | | 0.99 | | | - | 0.6803 | 9.74E-02 | | |
| CF317_007434-T1 | A0A0N1P1F3_9EURO | Serine/threonine-protein kinase ppk6 *(Phialophora attinorum)* | 3.281 | 59 | | 0.0 | Cytoplasm | | 0.7 | | | EC:2.7.11 | 0.6797 | 1.44E-01 | | |
| CF317_003122-T1 | A0A0S7DZQ1_9EURO | 2-(R)-hydroxypropyl-CoM dehydrogenase (Aspergillus lentulus) | 354 | 45.6 | | 5.5E-39 | Extracellular space | | 1 | | | - | 0.6658 | 3.01E-01 | | |
| CF317_003773-T1 | A0A0N1HPS7_9EURO | Zinc-regulated transporter 1 *(Phialophora attinorum)* | 1.437 | 55.4 | | 0.0 | Anchored component of plasma membrane | | 0.85 | | | - | 0.6633 | 1.42E-02 | | |
| CF317_009191-T1 | A0A2P8A4S9_9PEZI | Choline transport protein *(Elsinoe australis)* | 1.780 | 61 | | 0.0 | Plasma membrane | | 0.88 | | | - | 0.6590 | 9.34E-03 | | |
| CF317_004634-T1 | A0A0D2GDP2_9EURO | **Fe2OG dioxygenase domain-containing protein** *(Phialophora americana)* | 1.566 | 83.2 | | 0.0 | Cytoplasm | | 1 | | | - | 0.6562 | 2.35E-01 | | |
| CF317_007500-T1 | A0A072PWW7_9EURO | **Ammonium transporter (**Exophiala aquamarina CBS 119918) | 2.250 | 77 | | 0.0 | Plasma membrane | | 0.82 | | | - | 0.6548 | 2.80E-01 | | |
| CF317_005438-T1 | A0A178ZAV1_9EURO | **FAD-binding PCMH-type domain-containing protein** *(Fonsecaea erecta)* | 1.253 | 49.8 | | 2.4E-165 | Plasma membrane | | 0.84 | | | - | 0.6547 | 7.00E-02 | | |
| CF317_000103-T1 | A0A6A6DDQ8_9PEZI | Zinc carboxypeptidase *(Zopfia rhizophila CBS 207.26)* | 1.605 | 64.4 | | 0.0 | Extracellular space | | 0.97 | | | EC:3.4.17.11; EC:3.4.17.22; EC:3.4.17.10; EC:3.4.17.21 | 0.6512 | 1.88E-01 | | |
| CF317_006618-T1 | T0KY66_COLGC | Geranylgeranyl pyrophosphate synthetase (Colletotrichum gloeosporioides strain Cg-14) | 788 | 44.8 | | 6.2E-98 | Mitochondrion | | 1 | | | - | 0.6426 | 5.85E-02 | | |
| CF317_004271-T1 | A0A178D2A0_9EURO | 40S ribosomal protein S19 *(Fonsecaea nubica)* | 132 | 23.4 | | 1.8E-6 | Extracellular space | | 1 | | | - | 0.6355 | 2.66E-01 | | |
| CF317_004579-T1 | A0A072PJ62_9EURO | **Acyl-CoA desaturase** *(Exophiala aquamarina*CBS 119918) | 2.333 | 87.7 | | 0.0 | Endomembrane system | | 0.77 | | | EC:1.14.19.1; EC:1.14.19 | 0.6334 | 1.95E-05 | | |
| CF317_004551-T1 | A0A0D2GVY9_9EURO | **Carboxypeptidase** *(Rhinocladiella mackenziei* CBS 650.93) | 2.133 | 73.6 | | 0.0 | Extracellular space | | 1 | | | EC:3.4.17.11; EC:3.4.21; EC:3.4.16; EC:3.4.16.2 | 0.6315 | 5.66E-01 | | |
| CF317_002106-T1 | W9XJQ8_9EURO | **BAR domain-containing protein** *(Capronia coronata CBS 617.96)* | 1.188 | 58.6 | | 2.1E-159 | Cytoplasm | | 0.7 | | | - | 0.6190 | 1.67E-02 | | |
| CF317_008679-T1 | A0A1C1D1Y5_9EURO | Pdp3-interacting factor 1 *(Cladophialophora carrionii)* | 1.305 | 74.8 | | 7.5E-180 | Cytoplasm | | 1 | | | - | 0.6152 | 6.00E-02 | | |
| CF317_003440-T1 | A0A2T6ZJW0_TUBBO | **ARID domain-containing protein** *(Tuber borchii)* | 123 | 26.1 | | 6.8E-5 | Nucleus | | 1 | | | - | 0.6109 | 1.11E-01 | | |
| CF317_007869-T1 | A0A3M7NMJ9_9EURO | **Adaptin_N domain-containing protein** *(Chaetothyriales sp. CBS 134916)* | 288 | 27.9 | | 1.3E-25 | Nucleus | | 1 | | | - | 0.6092 | 3.87E-02 | | |
| CF317_003324-T1 | W2S990_9EURO | **ATP citrate synthase** *(Cyphellophora europaea CBS*101466) | 2.252 | 88 | | 0.0 | Cytoplasm | | 1 | | | - | 0.6024 | 5.38E-03 | | |
| CF317_003301-T1 | A0A0D2FAU6_9EURO | **1,3-beta-glucanosyltransferase** (*Phialophora americana)* | 1.541 | 58.7 | | 0.0 | Anchored component of plasma membrane | | 1 | | | - | 0.6008 | 4.13E-03 | | |
| CF317_008807-T1 | L7HNA7_MAGOY | **HemS domain-containing protein** *(Magnaporthe oryzae strain Y34)* | 132 | 38.6 | | 5.3E-8 | Cytoplasm | | 0.7 | | | - | 0.5860 | 2.75E-01 | | |
| CF317_000256-T1 | A0A0D2EBC5_9EURO | **ABM domain-containing protein** *(Exophiala xenobiotica)* | 534 | 45.5 | | 8.7E-65 | Cytoplasm | | 0.72 | | | - | -0.5912 | 3.63E-02 | | |
| CF317_009661-T1 | A0A4U0UWB0_9PEZI | **Fungal_trans domain-containing protein** *(Friedmanniomyces endolithicus)* | 236 | 67.2 | | 6E-25 | Cytoplasm | | 0.7 | | | - | -0.5936 | 4.26E-01 | | |
| CF317_005063-T1 | A0A1C1CIL8_9EURO | Mitochondrial intermembrane space cysteine motif-containing protein MIC17 *(Cladophialophora carrionii)* | 511 | 66.5 | | 2E-64 | Cytoplasm | | 0.7 | | | - | -0.5963 | 4.48E-01 | | |
| CF317_0002308-T1 | A0A1J9RJA8_9PEZI | **Carboxylic ester hydrolase** *(Diplodia corticola)* | 1.819 | 63 | | 0.0 | Extracellular space | | 0.99 | | | - | -0.6053 | 1.47E-02 | | |
| CF317_008545-T1 | A0A177EVH9_9EURO | **Gamma-glutamylcyclotransferase** (*Fonsecaea monophora)* | 1.035 | 58.1 | | 8.9E-126 | Endomembrane system | | 0.77 | | | EC:4.3.2.9 | -0.6291 | 9.64E-03 | | |
| CF317_003242-T1 | R4XE87_TAPDE | Catalase *(Taphrina deformans* PYCC 5710*)* | 2.389 | 77.5 | | 0.0 | Cytoplasm | | 1 | | | EC:1.11.1.7; EC:1.11.1.6 | -0.6330 | 7.85E-02 | | |
| CF317_004619-T1 | A0A1C1CJM0_9EURO | **Flavin_Reduct domain-containing protein** *(Cladophialophora carrionii)* | 322 | 41.2 | | 1.5E-31 | Cytoplasm | | 0.7 | | | - | -0.6345 | 4.31E-02 | | |
| CF317_006443-T1 | A0A178Z4C4_9EURO | **ABM domain-containing protein** *(Fonsecaea erecta)* | 281 | 50 | | 4E-31 | Cytoplasm | | 0.7 | | | - | -0.6352 | 2.50E-02 | | |
| CF317_009392-T1 | A0A4U0UWB0_9PEZI | **Fungal_trans domain-containing protein** (*Friedmanniomyces endolithicus)* | 551 | 79.3 | | 1.9E-66 | Cytoplasm | | 0.73 | | | - | -0.6382 | 1.93E-01 | | |
| CF317_003231-T1 | A0A6G1KVW9_9PEZI | Putative isomerase YbhE *(Teratosphaeria nubilosa)* | 1.282 | 62.8 | | 5.3E-174 | Extracellular space | | 0.84 | | | - | -0.6430 | 3.99E-02 | | |
| CF317_000372-T1 | A0A1M3THN7_ASPLC | **GPI ethanolamine phosphate transferase 2** *(Aspergillus luchuensis CBS 106.47)* | 203 | 40.6 | | 8.1E-17 | Nucleus | | 1 | | | - | -0.6548 | 8.68E-02 | | |
| CF317_0006744-T1 | A0A0F0IPQ9_ASPPU | Ribosomal protein L37e *(Aspergillus parasiticus* ATCC 56775) | 242 | 49.5 | | 8.2E-27 | Mitochondrion | | 1 | | | - | -0.6649 | 1.96E-01 | | |
| CF317_0001542-T1 | A0A072P870_9EURO | POT family proton-dependent oligopeptide transporter (*Exophiala aquamarina CBS 119918)* | 2.605 | 75.3 | | 0.0 | Plasma membrane | | 0.92 | | | - | -0.6830 | 8.37E-03 | | |
| CF317_0009683-T1 | W9ZBZ7_9EURO | **Endo-1,3(4)-beta-glucanase** *(Capronia epimyces CBS 606.96)* | 2.620 | 65.7 | | 0.0 | Cytoplasm | | 0.7 | | | EC:3.2.1.21; EC: 3.2.1.58; EC:3.2.1.39; EC:3.2.1.6 | -0.6918 | 2.10E-02 | | |
| CF317_0008007-T1 | A0A178ZBB8_9EURO | **ATPase_AAA_core domain-containing protein** *(Fonsecaea erecta)* | 1.353 | 55.2 | | 0.0 | Cytoplasm | | 0.7 | | | - | -0.6952 | 3.08E-02 | | |
| CF317_004480-T1 | A0A178CTP1_9EURO | **WW domain-containing protein** *(Fonsecaea nubica)* | 670 | 54.2 | | 6E-86 | Cytoplasm | | 0.7 | | | - | -0.6957 | 1.58E-01 | | |
| CF317_000112-T1 | A0A0D2EKS0_9EURO | 60S ribosomal protein L34-B *(Exophiala xenobiotica)* | 529 | 84.6 | | 1.1E-68 | Mitochondrion | | 1 | | | - | -0.7009 | 9.84E-02 | | |
| CF317_001574-T1 | A0A072P3G5_9EURO | **EthD domain-containing protein** (*Exophiala aquamarina CBS 119918)* | 330 | 54.8 | | 1.3E-38 | Cytoplasm | | 0.7 | | | - | -0.7129 | 3.02E-02 | | |
| CF317_000184-T1 | A0A072PMQ4_9EURO | Large subunit ribosomal protein L24e *(Exophiala aquamarina CBS*119918) | 704 | 89.4 | | 5.6E-94 | Nucleus | | 1 | | | - | -0.7218 | 2.77E-01 | | |
| CF317_0008154-T1 | H6C3S8_EXODN | Alcohol dehydrogenase *(Exophiala dermatitidis CBS 525.76)* | 1.336 | 70.9 | | 0.0 | Cytoplasm | | 1 | | | - | -0.7220 | 4.98E-03 | | |
| CF317_0008800-T1 | A0A0L0UZR5_9BASI | **GRIP domain-containing protein** *(Puccinia striiformis* f. sp. tritici PST-78) | 99 | 29.2 | | 5.6E-3 | Nucleus | | 1 | | | - | -0.7329 | 3.48E-03 | | |
| CF317_007618-T1 | A0A6A6HNE7_9PEZI | **Carboxylic ester hydrolase** *(Viridothelium virens)* | 1.873 | 60.8 | | 0.0 | Extracellular space | | 1 | | | - | -0.7365 | 4.22E-02 | | |
| CF317_003175-T1 | A0A1C1CN69_9EURO | Poly(A) polymerase (*Cladophialophora carrionii)* | 340 | 39.8 | | 1.6E-37 | Cytoplasm | | 0.7 | | | - | -0.7450 | 9.79E-03 | | |
| CF317_008131-T1 | A0A1L9R4I8_ASPWE | **Arabinan endo-1,5-alpha-L-arabinosidase** *(Aspergillus wentii DTO 134E9)* | 84 | 41.7 | | 4E-1 | Extracellular space | | 1 | | | - | -0.7621 | 4.71E-02 | | |
| CF317_006402-T1 | H6BYH4_EXODN | **4-hydroxyphenylpyruvate dioxygenase** *(Exophiala dermatitidis CBS 525.76)* | 1.643 | 76 | | 0.0 | Extracellular space | | 1 | | | EC:1.13.11; EC:1.13.11.27 | -0.7730 | 4.57E-04 | | |
| CF317_002864-T1 | A0A0N0NHK1_9EURO | Tripeptidyl aminopeptidase *(Phialophora attinorum)* | 1.121 | 45.4 | | 2E-145 | Anchored component of plasma membrane | | 0.99 | | | - | -0.7762 | 1.10E-02 | | |
| CF317_000904-T1 | A0A4V3UMQ9_9EURO | **RRM domain-containing protein** *(Aspergillus tanneri)* | 141 | 29.1 | | 5.6E-8 | Cytoplasm | | 0.7 | | | - | -0.7774 | 1.32E-01 | | |
| CF317_002933-T1 | H6BVJ2_EXODN | 2,2-dialkylglycine decarboxylase (Pyruvate) (*Exophiala dermatitidis CBS 525.76)* | 1.961 | 79.4 | | 0.0 | Cytoplasm | | 1 | | | - | -0.8184 | 1.91E-01 | | |
| CF317_001911-T1 | A0A3M7N5U6_9EURO | MFS transporter, SP family, major inositol transporter *(Capronia coronata CBS 617.96)* | 2.232 | 74.9 | | 0.0 | Plasma membrane | | 0.95 | | |  | -0.8427 | 1.84E-02 | | |
| CF317_001497-T1 | A0A067MPR6_9AGAM | Uncharacterized protein *(Botryobasidium botryosum* FD-172 SS1) | 81 | 31.2 | | 1.1E0 | Extracellular space | | 1 | | | EC:2.3.1.5 | -0.8512 | 1.21E-02 | | |
| CF317_009385-T1 | A0A397G8Q6_9EURO | **N-acetyltransferase domain-containing protein** *(Aspergillus thermomutatus)* | 643 | 51.2 | | 1E-81 | Cytoplasm | | 1 | | | - | -0.8626 | 2.93E-04 | | |
| CF317_001796-T1 | A0A072P3Q3_9EURO | **DJ-1_PfpI domain-containing protein** (*Exophiala aquamarina CBS 119918)* | 522 | 42.9 | | 1E-60 | Extracellular space | | 1 | | | - | -0.8773 | 6.08E-03 | | |
| CF317_009779-T1 | H6BQE2_EXODN | Extracellular cell wall glucanase Crf1/allergen Asp F9 (*Exophiala dermatitidis* CBS 525.76) | 986 | 38.9 | | 3.1E-122 | Extracellular space | | 1 | | | - | -0.8811 | 2.70E-01 | | |
| CF317_006732-T1 | A0A177D101_9PLEO  - | Gluconolactonase *(Paraphaeosphaeria sporulosa)* | 1.498 | 75.3 | | 0.0 | Extracellular space | | 1 | | | - | -0.9092 | 2.33E-02 | | |
| CF317_002368-T1 | B6H4B7_PENRW | Pc13g08300 protein *(Penicillium rubens*  ***ATCC 28089)*** | 214 | 39.3 | | 2.6E-19 | Extracellular space | | 0.93 | | | - | -0.9208 | 1.67E-04 | | |
| CF317_004743-T1 | A0A1C1CS71_9EURO | STF2-like protein *(Cladophialophora carrionii)* | 479 | 71.4 | | 2.5E-60 | Nucleus | | 1 | | | - | -0.9343 | 2.35E-02 | | |
| CF317_009003-T1 | A0A0D1W724_9EURO | **Serine/threonine-protein kinase TOR** *(Exophiala sideris)* | 10.168 | 78.4 | | 0.0 | Plasma membrane | | 0.71 | | | EC:2.7.11 | -0.9558 | 9.75E-02 | | |
| CF317_007018-T1 | A0A0D2IHR9_9EURO | **3-phytase** *(Rhinocladiella mackenziei*CBS 650.93) | 2.064 | 67.4 | | 0.0 | Plasma membrane | | 0.97 | | | EC:3.1.3.2 | -0.9947 | 4.67E-04 | | |
| CF317_007621-T1 | A0A0D2AG04_9PEZI | **Cutinase** *(Verruconis gallopava)* | 704 | 54 | | 3.8E-91 | Extracellular space | | 1 | | | EC:3.1.1.1 | -1.0366 | 7.69E-04 | | |
| CF317_000687-T1 | R8BNJ4_TOGMI | **Catalase** *(Togninia minima* UCR-PA7) | 2.663 | 83.6 | | 0.0 | Cytoplasm | | 0.59 | | |  | -1.1410 | 6.34E-02 | | |
| CF317_002178-T1 | A0A365NCF7_GIBIN | **Indoleamine 2,3-dioxygenase** *(*Gibberella intermedia*)* | 1.512 | 56.1 | | 0.0 | Cytoplasm | | 0.7 | | | - | -1.1838 | 5.88E-04 | | |
| CF317_003479-T1 | A0A0L0SYM3_ALLM3 | **Centrosomal protein of 19 kDa** *(Allomyces macrogynus* ATCC 38327) | 97 | 32.1 | | 3.7E-2 | Extracellular space | | 0.99 | | | - | -1.2918 | 3.83E-03 | | |
| CF317_009090-T1 | B8M229_TALSN | **Spindle assembly checkpoint component MAD1** *(Talaromyces stipitatus* ATCC 10500\|) | 96 | 32 | | 2.1E-2 | Nucleus | | 1 | | | - | -1.5467 | 1.52E-03 | | |

^a^ Protein accession number in the *K. chersonesos* database of *ab initio* translated proteins

^b^Up- and down-regulation of proteins (expressed as log2 fold change) detected in the LSSMG-exposed whole-cell proteome of *K. chersonesos* Wt compared to the unexposed proteome.

**Supplementary Table 5_**Homologues of differentially abundant proteins in LSSMG-exposed *K. chersonesos* Mut whole-cell proteome (BLASTP algorithm). Only the matching proteins with known identity (when available) and the highest max score are displayed. The predicted protein subcellular localizations are also shown.

| ***Protein* accession No. ^a^** | **UniProtKb accession**  **No.** | **Protein name** | **Max**  **Score** | **Identity (%)** | | **Expected**  **value** | **Predicted Localization** | **BUSCA Score** | | **KEGG Enzymes code** | | | **LSSMG/Control**  **FC** **^b^** | **p-value** | |
| --- | --- | --- | --- | --- | --- | --- | --- | --- | --- | --- | --- | --- | --- | --- | --- |
| CF317_004755-T1 | A0A0D2EW74_9EURO | **Glutamate dehydrogenase** *(Exophiala xenobiotica)* | 2.044 | 85.3 | | 0.0 | Cytoplasm | 0.96 | | | | EC:1.4.1.3; EC:1.4.1.4 | 1.9810 | 3.54E-01 | |
| CF317_007479-T1 | A0A0F4GNL1_9PEZI | Carbonic anhydrase like protein *(Zymoseptoria brevis)* | 956 | 60.8 | | 4.4E-128 | Extracellular space | 0.99 | | | | EC:4.2.1.1 | 1.8852 | 8.70E-02 |  |
| CF317_007500-T1 | A0A072PWW7_9EURO | **Ammonium transporter** *(Exophiala aquamarina CBS 119918)* | 2.250 | 77.0 | | 0.0 | Plasma membrane | 0.82 | | | | - | 1.6152 | 2.80E-01 |  |
| CF317_009191-T1 | A0A2P8A4S9_9PEZI | Choline transport protein *(Elsinoe australis)* | 1.780 | 61.0 | 0.0 | | Plasma membrane | | 0.88 | | | - | 1.2684 | 9.34E-03 |  |
| CF317_006944-T1 | A0A0D1ZPI5_9EURO | **Ig-like domain-containing protein** *(Cladophialophora immunda)* | 295 | 36.1 | 4.5E-28 | | Mitochondrion | | 0.96 | | | - | 1.2649 | 8.12E-02 |  |
| CF317_009031-T1 | A0A1Q8RG26_9PEZI | **Carboxylic ester hydrolase** *(Colletotrichum chlorophyti)* | 2.111 | 69.3 | 0.0 | | Extracellular space | | 0.74 | | | - | 1.1163 | 4.22E-02 |  |
| CF317_004810-T1 | A0A1C1CCK9_9EURO | NAD(P)-binding Rossmann-fold containing protein *(Cladophialophora carrionii)* | 1.252 | 78.5 | 9.8E-173 | | Cytoplasm | | 1 | | | - | 1.0313 | 3.48E-01 |  |
| CF317_009434-T1 | H6C9Y9_EXODN | Urease accessory protein *(Exophiala dermatitidis CBS 525.76)* | 1.180 | 82.3 | 2.9E-162 | | Cytoplasm | | 1 | | | EC:3.6.1.15 | 1.0239 | 1.32E-01 |  |
| CF317_001445-T1 | A8NA52_COPC7 | **DUF2183 domain-containing protein** *(Coprinopsis cinerea*Okayama-7) | 99 | 25 | 8.5E-2 | | Extracellular space | | 1 | | | - | 0.9560 | 4.86E-02 |  |
| CF317_007501-T1 | A0A0D1Z9Z1_9EURO | **Glutamine synthetase** *(Exophiala sideris)* | 1.771 | 86.7 | 0.0 | | Extracellular space | | 1 | | | EC:6.3.1.2 | 0.9516 | 2.84E-01 |  |
| CF317_005369-T1 | A0A0D2FEL3_9EURO | **AA_permease domain-containing protein** *(Exophiala xenobiotica)* | 2.248 | 75.5 | 0.0 | | Plasma membrane | | 0.85 | | - | | 0.9504 | 4.30E-01 |  |
| CF317_004400-T1 | A0A0D1ZSU6_EXOME | **Lipocln_cytosolic_FA-bd_dom domain-containing protein** *(Exophiala mesophila)* | 476 | 48.2 | 8.4E-58 | | Extracellular space | | 1 | | - | | 0.9048 | 6.58E-04 |  |
| CF317_006213-T1 | A0A0D2GI50_9EURO | **Inositol-3-phosphate synthase** *(Fonsecaea pedrosoi CBS 271.37)* | 2.385 | 83.1 | 0.0 | | Cytoplasm | | 0.7 | | EC:5.5.1.4 | | 0.8851 | 2.34E-01 |  |
| CF317_004579-T1 | A0A072PJ62_9EURO | **Acyl-CoA desaturase** *(Exophiala aquamarina CBS* 119918) | 2.333 | 87.7 | 0.0 | | Endomembrane system | | 0.77 | | EC:1.14.19.1; EC:1.14.19 | | 0.8258 | 1.95E-05 |  |
| CF317_008503-T1 | W9YGG3_9EURO | **Thiamine thiazole synthase** *(Capronia epimyces CBS 606.96)* | 1.393 | 81 | 0.0 | | Cytoplasm | | 1 | | - | | 0.8255 | 2.30E-01 |  |
| CF317_007680-T1 | A0A3M0W8S3_9EURO | **V-ATPase_H_C domain-containing protein** *(Chaetothyriales sp. CBS 134920)* | 1.557 | 44.7 | 0.0 | | Cytoplasm | | 0.7 | | - | | 0.7915 | 8.08E-03 |  |
| CF317_003663-T1 | A0A177FN24_9EURO | **BAH domain-containing protein** *(Fonsecaea monophora)* | 317 | 62.6 | 1.5E-32 | | Extracellular space | | 1 | | - | | 0.7599 | 1.16E-01 |  |
| CF317_003222-T1 | H6C0Q5_EXODN | CNT family concentrative nucleoside transporter *(Exophiala dermatitidis CBS 525.76)* | 2.814 | 83 | 0.0 | | Plasma membrane | | 0.81 | | - | | 0.7381 | 4.62E-01 |  |
| CF317_009400-T1 | W9W6L5_9EURO | Urease accessory protein *(Cladophialophora yegresii*CBS 114405) | 1.169 | 82.2 | 4.6E-161 | | Cytoplasm | | 1 | | EC:3.6.1.15 | | 0.7376 | 1.37E-01 |  |
| CF317_000532-T1 | A0A0D2C8U2_9EURO | Uncharacterized protein (Exophiala xenobiotica) | 2.998 | 63.7 | 0.0 | | Nucleus | | 1 | | - | | 0.7363 | 3.78E-01 |  |
| CF317_008346-T1 | A0A1J9RUB5_9PEZI | Conidiation-specific protein *(Diplodia corticola)* | 126 | 41.4 | 4.4E-8 | | Nucleus | | 1 | |  | | 0.7326 | 2.32E-03 |  |
| CF317_008807-T1 | L7HNA7_MAGOY | **HemS domain-containing protein** *(Magnaporthe oryzae* Y34) | 132 | 38.6 | 5.3E-8 | | Cytoplasm | | 0.7 | | - | | 0.7308 | 2.75E-01 |  |
| CF317_006388-T1 | S7Z8A1_PENO1 | **Nitric oxide dioxygenase** *(Penicillium oxalicum 114-2)* | 1.018 | 46.1 | 6.4E-133 | | Cytoplasm | | 1 | | EC:1.14.12; EC:1.14.12.17; EC:1.5.1.30; EC:1.5.1.41 | | 0.7290 | 3.16E-01 |  |
| CF317_005866-T1 | W9W6L5_9EURO | Urease accessory protein *(Cladophialophora yegresii* CBS 114405*)* | 1.176 | 82.2 | 4.3E-162 | | Cytoplasm | | 1 | | EC:3.6.1.15 | | 0.7283 | 4.24E-02 |  |
| CF317_004702-T1 | A0A179FUL4_PURLI | Quinoprotein amine dehydrogenase beta chain-like protein *(Purpureocillium lilacinum)* | 439 | 39.6 | 1.8E-48 | | Extracellular space | | 0.77 | | - | | 0.7259 | 5.04E-01 |  |
| CF317_002497-T1 | A0A178CKS4_9EURO | **SEC7 domain-containing protein** *(Fonsecaea multimorphosa)* | 5.291 | 64.3 | 0.0 | | Endomembrane system | | 0.92 | | - | | 0.7191 | 7.38E-02 |  |
| CF317_007856-T1 | A0A0D2AWB9_9EURO | **WD_REPEATS_REGION domain-containing protein** *(Exophiala oligosperma)* | 1.486 | 80.8 | 0.0 | | Nucleus | | 1 | | - | | 0.7141 | 1.88E-02 |  |
| CF317_008070-T1 | A0A438MS86_EXOME | Urease accessory protein G *(Exophiala mesophila)* | 1.157 | 82.1 | 2.8E-159 | | Extracellular space | | 1 | | EC:3.6.1.15 | | 0.7050 | 3.58E-02 |  |
| CF317_007205-T1 | V9DNR6_9EURO | **NFACT-R_1 domain-containing protein** *(Cladophialophora carrionii CBS 160.54)* | 971 | 82.6 | 9.3E-133 | | Nucleus | | 1 | | - | | 0.7013 | 1.95E-01 |  |
| CF317_006807-T1 | A0A3M6XH93_HORWE | **But2 domain-containing protein** *(Hortaea werneckii)* | 178 | 37.1 | 1.6E-12 | | Mitochondrion | | 1 | | - | | 0.7011 | 5.89E-02 |  |
| CF317_007246-T1 | A0A0D2CQE8_9EURO | **Aldo_ket_red domain-containing protein** *(Cladophialophora immunda)* | 1.092 | 72.3 | 3.7E-149 | | Cytoplasm | | 1 | | - | | 0.6898 | 5.79E-02 |  |
| CF317_009070-T1 | A0A0D2GZY4_9EURO | **Stress response protein NST1** *(Fonsecaea pedrosoi CBS 271.37)* | 3.380 | 59.9 | 0.0 | | Plasma membrane | | 0.89 | | - | | 0.6604 | 1.86E-01 |  |
| CF317_001440-T1 | A0A3M7MUU7_9EURO | **C2 domain-containing protein** *(Chaetothyriales sp. CBS 135597)* | 3.065 | 52.2 | 0.0 | | Cytoplasm | | 0.7 | | EC:3.6.1.3; EC:3.6.1.15 | | 0.6590 | 3.57E-01 |  |
| CF317_003266-T1 | A0A1C1CNC5_9EURO | Malate dehydrogenase (*Cladophialophora carrionii)* | 551 | 45.7 | 1.2E-67 | | Extracellular space | | 0.99 | | - | | 0.6530 | 5.07E-03 |  |
| CF317_002132-T1 | A0A438N399_EXOME | **AA_permease domain-containing protein** (*Exophiala mesophila)* | 2.182 | 75.8 | 0.0 | | Plasma membrane | | 0.88 | | - | | 0.6505 | 4.55E-02 |  |
| CF317_007794-T1 | A0A3M7NY82_9EURO | **Cysteine dioxygenase** *(Chaetothyriales sp. CBS 134916)* | 671 | 64.6 | 5.9E-87 | | Cytoplasm | | 0.7 | | EC:1.13.11 | | 0.6403 | 5.07E-04 |  |
| CF317_001627-T1 | A0A5M3YSQ2_ASPTE | Uncharacterized protein *(Aspergillus terreus)* | 546 | 48.5 | 1.3E-63 | | Cytoplasm | | 1 | | - | | 0.6348 | 5.96E-01 |  |
| CF317_002650-T1 | A0A0W7VBN3_9HYPO | **Tautomerase_3 domain-containing protein** *(Trichoderma gamsii)* | 374 | 45.4 | 3.1E-43 | | Cytoplasm | | 0.7 | | - | | 0.6333 | 2.00E-03 |  |
| CF317_007133-T1 | A0A1C1CV53_9EURO | **Protein FYV10** *(Cladophialophora carrionii)* | 1.311 | 46.5 | 6.7E-174 | | Nucleus | | 1 | | - | | 0.6298 | 1.26E-02 |  |
| CF317_001165-T1 | A0A0D2DJ12_9EURO | **Malic enzyme** *(Cladophialophora immunda)* | 2.057 | 69.1 | 0.0 | | Cytoplasm | | 1 | | EC:1.1.1.38; EC:1.1.1.39 | | 0.6220 | 4.41E-01 |  |
| CF317_008621-T1 | A0A254TSD8_ASPNG | CENP-Q, a CENPA-CAD centromere complex subunit family protein *(Aspergillus niger)* | 179 | 36.8 | 2.7E-15 | | Nucleus | | 1 | | - | | 0.6126 | 2.82E-01 |  |
| CF317_007996-T1 | B6H865_PENRW | Pc16g03980 protein (*Penicillium rubens* ATCC 28089) | 469 | 47.7 | 4.3E-56 | | Nucleus | | 1 | | - | | 0.6070 | 3.44E-05 |  |
| CF317_004816-T1 | A0A0D2CHE2_9EURO | **D-3-phosphoglycerate dehydrogenase** *(Cladophialophora immunda)* | 2.181 | 73.1 | 0.0 | | Cytoplasm | | 1 | | EC:1.1.1.95 | | 0.6044 | 5.51E-03 |  |
| CF317_008544-T1 | R1GAU1_BOTPV | Putative fad binding domain-containing protein (*Botryosphaeria parva* UCR-NP2) | 932 | 48.7 | 9.2E-122 | | Extracellular space | | 1 | | - | | 0.6039 | 2.83E-02 |  |
| CF317_003380-T1 | W9WEU4_9EURO | Uncharacterized protein *(Cladophialophora psammophila* CBS 110553) | 483 | 24.7 | 1.8E-48 | | Mitochondrion | | 1 | | - | | 0.6017 | 9.50E-02 |  |
| CF317_009073-T1 | W9YH46_9EURO | Ureidoglycolate hydrolase *(Capronia coronata CBS 617.96)* | 298 | 47.8 | 4.5E-31 | | Extracellular space | | 1 | | EC:4.3.2.3; EC:3.5.3.19 | | -0.5861 | 3.14E-02 |  |
| CF317_002049-T1 | A0A0N1HG33_9EURO | Uncharacterized protein *(Phialophora attinorum)* | 687 | 70.6 | 3.7E-86 | | Extracellular space | | 1 | | - | | -0.5870 | 3.67E-02 |  |
| CF317_001965-T1 | M7SN88_EUTLA | Putative glutathione s-transferase ii-like protein *Eutypa lata UCR-EL1)* | 872 | 68.8 | 1.6E-117 | | Mitochondrion | | 1 | | - | | -0.5882 | 2.60E-03 |  |
| CF317_002844-T1 | A0A4U0VAR5_9PEZI | **Enhancer of polycomb-like protein** *(Friedmanniomyces endolithicus)* | 157 | 36.3 | 1.2E-10 | | Mitochondrion | | 1 | | - | | -0.5893 | 1.31E-03 |  |
| CF317_002693-T1 | A0A0D1Z4D5_EXOME | **Ornithine transcarbamylase** *(Exophiala mesophila)* | 1.238 | 68.6 | 5.5E-169 | | Mitochondrion | | 0.72 | | EC:2.1.3.3 | | -0.5899 | 2.51E-02 |  |
| CF317_001796-T1 | A0A072P3Q3_9EURO | **DJ-1_PfpI domain-containing protein** *(Exophiala aquamarina CBS 119918)* | 510 | 42.9 | 1E-60 | | Extracellular space | | 1 | | - | | -0.5957 | 6.08E-03 |  |
| CF317_000691-T1 | A0A0N1HQP3_9EURO | NEDD8-conjugating enzyme UBC12 *(Phialophora attinorum)* | 849 | 86.7 | 2.6E-115 | | Nucleus | | 1 | | - | | -0.5990 | 2.10E-01 |  |
| CF317_004248-T1 | H6BMU7_EXODN | **5-aminoimidazole-4-carboxamide ribonucleotide formyltransferase** *(Exophiala dermatitidis CBS 525.76)* | 2.573 | 83.3 | 0.0 | | Cytoplasm | | 1 | | EC:3.5.4.10; EC:2.1.2.3 | | -0.5992 | 1.23E-01 |  |
| CF317_002086-T1 | A0A0D2BHT9_9EURO | **AB hydrolase-1 domain-containing protein** *(Exophiala xenobiotica)* | 1.310 | 60.3 | 1E-177 | | Extracellular space | | 1 | | - | | -0.6016 | 1.98E-01 |  |
| CF317_000385-T1 | A0A438NHN6_EXOME | **AMPK1_CBM domain-containing protein** *(Exophiala mesophila)* | 142 | 36.4 | 1.5E-10 | | Extracellular space | | 1 | | - | | -0.6064 | 5.11E-02 |  |
| CF317_001222-T1 | H6BRE4_EXODN | ATP-binding cassette, sub-family F, member 3 *(Exophiala dermatitidis CBS 525.76)* | 1.709 | 51.2 | 0.0 | | Cytoplasm | | 0.7 | | - | | -0.6133 | 2.88E-03 |  |
| CF317_009017-T1 | A0A0D1VLY3_9EURO | **NodB homology domain-containing protein** *(Exophiala sideris)* | 1.402 | 83.5 | 0.0 | | Cytoplasm | | 1 | | - | | -0.6135 | 5.38E-02 |  |
| CF317_005813-T1 | U1GS43_ENDPU | **4HBT domain-containing protein** *(Endocarpon pusillum* Z07020) | 609 | 69.9 | 2.9E-79 | | Mitochondrion | | 1 | | EC:3.1.2.20 | | -0.6213 | 7.10E-02 |  |
| CF317_001523-T1 | W9VUL7_9EURO | **Glucose-6-phosphate 1-epimerase** *(Cladophialophora psammophila CBS 110553)* | 1.258 | 74.8 | 2E-173 | | Extracellular space | | 1 | | EC: 5.1.3.15 | | -0.6226 | 6.42E-02 |  |
| CF317_000932-T1 | A0A1C1D1N7_9EURO | 3,2-trans-enoyl-CoA isomerase *(Cladophialophora carrionii)* | 992 | 67.7 | 6.2E-134 | | Cytoplasm | | 1 | | EC:5.3.3.8 | | -0.6255 | 1.20E-02 |  |
| CF317_005543-T1 | A0A2J6PML4_9HELO | Amidohydrolase 2 *(Pezoloma ericae)* | 919 | 54.1 | 6.3E-121 | | Cytoplasm | | 1 | | - | | -0.6281 | 4.33E-02 |  |
| CF317_005472-T1 | W9YAM1_9EURO | NADPH2 quinone reductase *(Capronia epimyces CBS 606.96)* | 1.326 | 74.9 | 0.0 | | Cytoplasm | | 1 | | - | | -0.6289 | 2.00E-01 |  |
| CF317_000519-T1 | A0A0D2EGE0_XYLBA | **Arginase** *(Cladophialophora bantiana CBS 173.52)* | 1.253 | 65.3 | 8.5E-171 | | Cytoplasm | | 1 | | EC:3.5.3.1 | | -0.6439 | 1.12E-02 |  |
| CF317_006712-T1 | A0A3M7MQZ8_9EURO | **Histidinol dehydrogenase** *(Chaetothyriales sp. CBS 135597)* | 1.863 | 83.6 | 0.0 | | Cytoplasm | | 0.61 | | EC:1.1.1.23 | | -0.6459 | 2.39E-02 |  |
| CF317_001167-T1 | A0A1V6UYP5_9EURO | **Aldo_ket_red domain-containing protein** *(Penicillium coprophilum)* | 1.306 | 68.7 | 1.5E-179 | | Cytoplasm | | 1 | | - | | -0.6475 | 2.73E-01 |  |
| CF317_002961-T1 | W9VX79_9EURO | Carboxymethylenebutenolidase *(Cladophialophora yegresii CBS 114405)* | 1.164 | 78.1 | 2.6E-160 | | Cytoplasm | | 1 | | - | | -0.6486 | 4.58E-02 |  |
| CF317_000215-T1 | A0A0D1XB61_9EURO | **Fumarate hydratase** *(Exophiala sideris)* | 2.206 | 91.6 | 0.0 | | Cytoplasm | | 1 | | EC:4.2.1.2 | | -0.6543 | 2.38E-01 |  |
| CF317_004274-T1 | A0A438MZU1_EXOME | L-xylo-3-hexulose reductase *(Exophiala mesophila)* | 1.264 | 73.2 | 4.7E-174 | | Mitochondrion | | 1 | | - | | -0.6548 | 1.31E-01 |  |
| CF317_008800-T1 | A0A0L0UZR5_9BASI | **GRIP domain-containing protein** *(Puccinia striiformis* f. sp. tritici PST-78) | 99 | 29.2 | 5.6E-3 | | Nucleus | | 1 | | - | | -0.6562 | 3.48E-03 |  |
| CF317_002385-T1 | A0A1C1C7M6_9EURO | Agmatine deiminase *(Cladophialophora carrionii)* | 1.033 | 61.4 | 9.2E-139 | | Cytoplasm | | 0.7 | | EC:3.5.3.15 | | -0.6624 | 1.37E-02 |  |
| CF317_005236-T1 | B6QUH0_TALMQ | **T5orf172 domain-containing protein** (Talaromyces marneffei ATCC 18224) | 802 | 60.4 | 1.1E-99 | | Endomembrane system | | 0.73 | | - | | -0.6632 | 4.69E-02 |  |
| CF317_004968-T1 | W9XMZ0_9EURO | **Uroporphyrinogen decarboxylase** *(Capronia coronata CBS 617.96)* | 1.585 | 78.5 | 0.0 | | Mitochondrion | | 1 | | EC:4.1.1.37 | | -0.6744 | 1.52E-02 |  |
| CF317_004743-T1 | A0A1C1CS71_9EURO | STF2-like protein *(Cladophialophora carrionii)* | 479 | 71.4 | 2.5E-60 | | Nucleus | | 1 | | - | | -0.6796 | 2.35E-02 |  |
| CF317_008030-T1 | A0A0D1YYX4_9EURO | **D-amino-acid oxidase domain-containing protein** *(Exophiala sideris)* | 1.042 | 60.6 | 6.6E-140 | | Extracellular space | | 1 | | EC:1.4.3.3 | | -0.6799 | 2.92E-02 |  |
| CF317_009650-T1 | A0A319F479_9EURO | M6 metalloprotease *(Aspergillus ellipticus CBS 707.79)* | 1.158 | 55 | 2.1E-153 | | Extracellular space | | 1 | | - | | -0.6800 | 1.22E-01 |  |
| CF317_004909-T1 | W9W243_9EURO | **Adenylosuccinate synthetase** *(Cladophialophora yegresii* CBS 114405*)* | 1.642 | 65.8 | 0.0 | | Cytoplasm | | 1 | | EC:6.3.4.4 | | -0.6917 | 8.11E-02 |  |
| CF317_008924-T1 | W2S109_9EURO | **Homogentisate 1,2-dioxygenase** *(Cyphellophora europaea CBS*101466) | 2.182 | 81.7 | 0.0 | | Extracellular space | | 1 | | EC:1.13.11; EC:1.13.11.5 | | -0.6970 | 5.01E-01 |  |
| CF317_004252-T1 | W9YTM0_9EURO | Mesaconyl-C4 CoA hydratase *(Capronia coronata CBS 617.96)* | 1.220 | 66.7 | 7.4E-166 | | Mitochondrion | | 1 | | - | | -0.7017 | 4.01E-02 |  |
| CF317_005928-T1 | A0A0G4NFS0_9PEZI | **Pectinesterase** *(Verticillium longisporum)* | 102 | 37.7 | 1E-3 | | Cytoplasm | | 0.7 | | - | | -0.7042 | 2.48E-01 |  |
| CF317_001952-T1 | A0A2P2HDW9_ASPFA | Zn(2)-C6 fungal-type domain-containing protein *(*Aspergillus flavus ATCC MYA-384 / AF70) | 1.092 | 46.6 | 1.3E-133 | | Cytoplasm | | 0.7 | | - | | -0.7066 | 1.38E-01 |  |
| CF317_007970-T1 | A0A0D2CQV2_9EURO | **Multifunctional fusion protein** *(Exophiala xenobiotica)* | 2.324 | 78 | 0.0 | | Cytoplasm | | 1 | | EC:1.2.1.88 | | -0.7153 | 4.07E-01 |  |
| CF317_001911-T1 | W9Z1V7_9EURO | MFS transporter, SP family, major inositol transporter *(Capronia coronata CBS 617.96)* | 2.232 | 74.9 | 0.0 | | Plasma membrane | | 0.95 | | - | | -0.7301 | 1.84E-02 |  |
| CF317_008612-T1 | A0A438NJK2_EXOME | **Aldedh domain-containing protein** *(Exophiala mesophila)* | 1.978 | 74.5 | 0.0 | | Mitochondrion | | 1 | | EC:1.11.1.7 | | -0.7312 | 2.05E-01 |  |
| CF317_007784-T1 | A0A6A6HSY0_9PLEO | Cytochrome P450 (*Trematosphaeria pertusa)* | 2.048 | 68 | 0.0 | | Plasma membrane | | 0.71 | | - | | -0.7316 | 7.06E-02 |  |
| CF317_008154-T1 | H6C3S8_EXODN | Alcohol dehydrogenase *(Exophiala dermatitidis CBS 525.76)* | 1.336 | 70.9 | 0.0 | | Cytoplasm | | 1 | | - | | -0.7407 | 4.98E-03 |  |
| CF317_000649-T1 | A0A1C1CGB9_9EURO | Delta(3,5)-Delta(2,4)-dienoyl-CoA isomerase, mitochondrial *(Cladophialophora carrionii)* | 1.048 | 69.9 | 2.5E-142 | | Cytoplasm | | 1 | | EC:3.1.2.4 | | -0.7416 | 1.03E-02 |  |
| CF317_003168-T1 | A0A5N5WLY4_9EURO | Conidiation protein 6-domain-containing protein *(Aspergillus leporis)* | 243 | 56.3 | 6.4E-26 | | Nucleus | | 1 | | - | | -0.7419 | 2.97E-02 |  |
| CF317_002645-T1 | A0A2J6PYC1_9HELO | NAD-dependent epimerase/dehydratase-like protein *(Pezoloma ericae)* | 1.395 | 83.5 | 0.0 | | Organelle membrane | | 0.74 | | - | | -0.7498 | 4.55E-02 |  |
| CF317_007412-T1 | H6BQ73_EXODN | Histidinol-phosphate aminotransferase *(Exophiala dermatitidis CBS 525.76)* | 1.772 | 76.6 | 0.0 | | Cytoplasm | | 1 | | EC:2.6.19 | | -0.7514 | 5.09E-02 |  |
| CF317_007620-T1 | A0A1C1D1B3_9EURO | MmgE/PrpD family protein *(Cladophialophora carrionii)* | 1.200 | 51 | 8.4E-160 | | Mitochondrion | | 1 | | - | | -0.7561 | 2.28E-01 |  |
| CF317_008426-T1 | A0A0D1ZJU8_9EURO | **Glutathione reductase** *(Exophiala sideris)* | 2.057 | 79.5 | 0.0 | | Cytoplasm | | 1 | | EC:1.8.1.7 | | -0.7680 | 8.19E-02 |  |
| CF317_004619-T1 | A0A1C1CJM0_9EURO | **Flavin_Reduct domain-containing protein** *(Cladophialophora carrionii)* | 277 | 42.9 | 1.3E-25 | | Cytoplasm | | 0.7 | | - | | -0.7708 | 4.31E-02 |  |
| CF317_007720-T1 | A0A010Q717_9PEZI | Uncharacterized protein *(Colletotrichum fioriniae PJ7)* | 1.664 | 71 | 0.0 | | Cytoplasm | | 1 | | - | | -0.7786 | 1.67E-02 |  |
| CF317_002851-T1 | A0A3M0W9D5_9EURO | **HSF_DOMAIN domain-containing protein** *(Chaetothyriales sp. CBS 134920)* | 957 | 62 | 5E-116 | | Cytoplasm | | 1 | | - | | -0.8000 | 9.79E-02 |  |
| CF317_007018-T1 | A0A0D2IHR9_9EURO | **3-phytase** *(Rhinocladiella mackenziei CBS 650.93)* | 2.064 | 67.4 | 0.0 | | Plasma membrane | | 0.97 | | EC:3.1.3.2 | | -0.8087 | 4.67E-04 |  |
| CF317_006992-T1 | A0A178C5N3_9EURO | **Serine/threonine-protein kinase** *(Fonsecaea multimorphosa)* | 1.067 | 51.4 | 8.8E-131 | | Cytoplasm | | 1 | | - | | -0.8607 | 1.26E-02 |  |
| CF317_006089-T1 | A0A2D3V5I9_9PEZI | Related to zinc-binding oxidoreductase ToxD *(Ramularia collo-cygni)* | 1.512 | 78.3 | 0.0 | | Cytoplasm | | 1 | | - | | -0.8630 | 1.83E-01 |  |
| CF317_009172-T1 | H6BZ20_EXODN | Uncharacterized protein *(Exophiala dermatitidis CBS 525.76)* | 448 | 49.7 | 6.4E-54 | | Extracellular space | | 1 | | - | | -0.8672 | 5.51E-01 |  |
| CF317_003892-T1 | A0A0D2BGD8_9EURO | **Alkaline phosphatase** *(Exophiala spinifera)* | 2.244 | 66.2 | 0.0 | | Plasma membrane | | 0.82 | | - | | -0.8825 | 4.25E-01 |  |
| CF317_001542-T1 | A0A072P870_9EURO | POT family proton-dependent oligopeptide transporter (Exophiala aquamarina CBS 119918) | 2.605 | 75.3 | 0.0 | | Plasma membrane | | 0.92 | | - | | -0.8932 | 8.37E-03 |  |
| CF317_000687-T1 | R8BNJ4_TOGMI | **Catalase** *(Togninia minima (strain UCR-*PA7*)* | 2.663 | 83.6 | 0.0 | | Cytoplasm | | 0.59 | | EC:1.11.1.7; EC:1.11.1.6 | | -0.8987 | 6.34E-02 |  |
| CF317_007070-T1 | A0A072NZJ3_9EURO | **Multifunctional fusion protein** *(Exophiala aquamarina CBS 119*918) | 2.348 | 78.3 | 0.0 | | Mitochondrion | | 0.84 | | EC:1.2.1.88 | | -0.9090 | 6.03E-02 |  |
| CF317_008773-T1 | U1G9G0_ENDPU | **Catalase-peroxidase** *(Endocarpon pusillum* Z07020) | 3.237 | 78.2 | 0.0 | | Cytoplasm | | 1 | | Ec:1.11.1.7; EC:1.11.1.6 | | -0.9459 | 3.42E-02 |  |
| CF317_000904-T1 | A0A2H3I859_9EURO | Nucleotide-binding, alpha-beta plait *(Penicillium sp. 'occitanis')* | 144 | 35.9 | 2.2E-8 | | Cytoplasm | | 0.7 | | - | | -0.9636 | 1.32E-01 |  |
| CF317_003175-T1 | A0A1C1CN69_9EURO | Poly(A) polymerase (*Cladophialophora carrionii)* | 340 | 39.8 | 1.6E-37 | | Cytoplasm | | 0.7 | | - | | -0.9882 | 9.79E-03 |  |
| CF317_009385-T1 | A0A397G8Q6_9EURO | **N-acetyltransferase domain-containing protein** *(Aspergillus thermomutatus)* | 643 | 51.2 | 1E-81 | | Cytoplasm | | 1 | | EC:2.3.1.5 | | -0.9956 | 2.93E-04 |  |
| CF317_002858-T1 | A0A420YNS7_9PEZI | **But2 domain-containing protein** *(Coniochaeta pulveracea)* | 95 | 26.2 | 1.1E-1 | | Anchored component of plasma membrane | | 0.99 | | - | | -1.0404 | 1.49E-03 |  |
| CF317_009683-T1 | W9ZBZ7_9EURO | **Endo-1,3(4)-beta-glucanase** *(Capronia epimyces CBS 606.96)* | 2.620 | 65.7 | 0.0 | | Cytoplasm | | 0.7 | | EC:3.2.1.21; EC:3.2.1.58; EC:3.2.1.39; EC:3.2.1.6 | | -1.0502 | 2.10E-02 |  |
| CF317_006732-T1 | A0A177D101_9PLEO | Gluconolactonase *(Paraphaeosphaeria sporulosa)* | 1.498 | 75.3 | 0.0 | | Extracellular space | | 1 | | - | | -1.0745 | 2.33E-02 |  |
| CF317_003065-T1 | W9YWI5_9EURO | 3-oxoacyl-[acyl-carrier protein] reductase *(Capronia coronata CBS 617.96)* | 1.255 | 77.3 | 7.1E-173 | | Mitochondrion | | 1 | | - | | -1.0848 | 8.91E-02 |  |
| CF317_006402-T1 | H6BYH4_EXODN | **4-hydroxyphenylpyruvate dioxygenase** *(Exophiala dermatitidis CBS 525.76)* | 1.643 | 76 | 0.0 | | Extracellular space | | 1 | | EC:1.13.11; EC:1.13.11.27 | | -1.1274 | 4.57E-04 |  |
| CF317_008285-T1 | A0A0U1M4Z9_TALIS | Mitomycin radical oxidase *(Talaromyces islandicus)* | 1.601 | 51.6 | 0.0 | | Extracellular space | | 0.99 | | - | | -1.1286 | 5.04E-01 |  |
| CF317_002864-T1 | A0A0N0NHK1_9EURO | Tripeptidyl aminopeptidase *(Phialophora attinorum)* | 1.121 | 45.4 | 2E-145 | | Anchored component of plasma membrane | | 0.99 | | - | | -1.2167 | 1.10E-02 |  |
| CF317_009090-T1 | B8M229_TALSN | **Spindle assembly checkpoint component MAD1** *(Talaromyces stipitatus* ATCC 10500*)* | 96 | 32 | 2.1E-2 | | Nucleus | | 1 | | - | | -1.3172 | 1.52E-03 |  |
| CF317_002178-T1 | A0A365NCF7_GIBIN | **Indoleamine 2,3-dioxygenase** *(*Gibberella intermedia*)* | 1.512 | 56.1 | 0.0 | | Cytoplasm | | 0.7 | | - | | -1.4328 | 5.88E-04 |  |

^a^ Protein accession number in the *K. chersonesos* database of *ab initio* translated proteins

^b^Up- and down-regulation of proteins (expressed as log2fold change) detected in the LSSMG-exposed whole-cell proteome of *K. chersonesos* Mut compared to the unexposed proteome.

**Supplementary Table 6_**Homologues of differentially abundant proteins in LSSMG-exposed *K. chersonesos* Wt secretome (BLASTP algorithm). Only the matching proteins with known identity (when available) and the highest max score are displayed. The predicted protein subcellular localizations are also shown.

| ***Protein* accession No. ^a^** | **UniProtKb accession**  **No.** | **Protein name** | | **Max**  **Score** | **Identity**  **(%)** | **Expected**  **value** | **Predicted Localization** | **BUSCA Score** | **KEGG Enzymes code** | **LSSMG/Control FC^b^** | **p-value** |
| --- | --- | --- | --- | --- | --- | --- | --- | --- | --- | --- | --- |
| CF317_000354-T1 | A0A4V1M521_TREME | | **C2 domain-containing protein** *(Tremella mesenterica)* | 138 | 26 | 5.7E-7 | Mitochondrion | 0.87 | - | 0.822 | 2.06E-02 |
| CF317_001633-T1 | B8MNS2_TALSN | | **Histone H2A** *(Talaromyces stipitatus* ATCC 10500) | 650 | 97.7 | 1.3E-86 | Mitochondrion | 1 | - | 0.752 | 9.78E-03 |
| CF317_001911-T1 | W9Z1V7_9EURO | | MFS transporter, SP family, major inositol transporter *(Capronia coronata CBS 617.96)* | 2.232 | 74.9 | 0.0 | Plasma membrane | 0.95 | - | 0.747 | 2.92E-02 |
| CF317_004119-T1 | A0A438NIP5_EXOME | | **Adenylosuccinate lyase** *(Exophiala mesophila)* | 1.713 | 70.4 | 0.0 | Cytoplasm | 0.82 | EC:4.3.2.2 | 0.703 | 1.58E-02 |
| CF317_002664-T1 | A0A0D2I7E3_9EURO | | **Plasma membrane ATPase** *(Rhinocladiella mackenziei*CBS 650.93) | 4.514 | 86.7 | 0.0 | Plasma membrane | 0.85 | EC:7.1.3.1; EC:7.1.2.1 | 0.702 | 3.25E-02 |
| CF317_004827-T1 | A0A438N3D5_EXOME | | Casein kinase I 1 *(Exophiala mesophila)* | 2.102 | 86.4 | 0.0 | Nucleus | 1 | - | 0.656 | 4.63E-02 |
| CF317_002935-T1 | H6BT56_EXODN | | Aromatic amino acid aminotransferase (Exophiala dermatitidis CBS 525.76) | 1.912 | 56.2 | 0.0 | Cytoplasm | 1 | - | 0.633 | 1.18E-02 |
| CF317_006825-T1 | A0A3M7NNV6_9EURO | | **M20_dimer domain-containing protein** *(Chaetothyriales sp. CBS 134916)* | 1.973 | 70.7 | 0.0 | Endomembrane system | 0.76 | EC:3.4.17.11; EC:3.4.17.22; EC:3.4.17.10; EC:3.4.17.21 | 0.588 | 1.22E-02 |
| CF317_002654-T1 | A0A0N1P280_9EURO | | Scytalone dehydratase *(Phialophora attinorum)* | 807 | 78.5 | 1.1E-108 | Cytoplasm | 1 | EC:4.2.1.94 | 0.587 | 3.70E-02 |
| CF317_002949-T1 | A0A177BY00_9PLEO | | Feruloyl esterase *(Paraphaeosphaeria sporulosa)* | 730 | 54.5 | 1E-93 | Extracellular space | 0.99 | EC:3.1.1.73; EC:3.1.1.1 | -0.859 | 1.16E-03 |
| CF317_008131-T1 | A0A1L9R4I8_ASPWE | | **Arabinan endo-1,5-alpha-L-arabinosidase***(Aspergillus wentii DTO 134E9)* | 84 | 41.7 | 4E-1 | Extracellular space | 1 | - | -0.998 | 4.32E-02 |

^a^ Protein accession number in the *K. chersonesos* database of *ab initio* translated proteins

^b^Up- and down-regulation of proteins (expressed as log2fold change) detected in the LSSMG-exposed secretome of *K. chersonesos* Wt compared to the unexposed secretome.

**Supplementary Table 7_**Homologues of differentially abundant proteins in LSSMG-exposed *K. chersonesos* Mut secretome (BLASTP algorithm). Only the matching proteins with known identity (when available) and the highest max score are displayed. The predicted protein subcellular localizations are also shown.

| ***Protein* accession No. ^a^** | **UniProtKb accession**  **No.** | **Protein name** | **Max**  **Score** | **Identity**  **(%)** | **Expected**  **value** | **Predicted Localization** | **BUSCA Score** | | **KEGG Enzymes code** | **LSSMG/**  **Control**  **FC** **^b^** | | **p-value** |  |
| --- | --- | --- | --- | --- | --- | --- | --- | --- | --- | --- | --- | --- | --- |
| CF317_002674-T1 | H6BUN2_EXODN | Cell wall glucanase (Scw11) *(Exophiala dermatitidis CBS 525.76)* | 117 | 27 | 2E-5 | Plasma membrane | 0.76 | | - | 1.484 | | 5.39E-03 |  |
| CF317_003266-T1 | A0A1C1CNC5_9EURO | Malate dehydrogenase *(Cladophialophora carrionii)* | 499 | 45.4 | 4.1E-60 | Extracellular space | 0.99 | | **-** | 1.244 | | 3.48E-02 |  |
| CF317_007601-T1 | A0A5K1K211_9APHY | Protein SEY1 (EC) *(Ganoderma boninense)* | 99 | 34.9 | 2.4E-2 | Extracellular space | 1 | | - | 1.080 | | 4.38E-03 |  |
| CF317_004400-T1 | A0A0D1ZSU6_EXOME | **Lipocln_cytosolic_FA-bd_dom domain-containing protein** *(Exophiala mesophila)* | 476 | 48.2 | 8.4E-58 | Extracellular space | 1 | | - | 1.017 | | 7.45E-03 |  |
| CF317_000051-T1 | A0A178DA36_9EURO | **FCP1 homology domain-containing protein** *(Fonsecaea nubica)* | 1.427 | 57.8 | 0.0 | Nucleus | 1 | | EC:3.1.3.16 | 0.906 | | 1.73E-02 |  |
| CF317_000614-T1 | A0A438MTW5_EXOME | **1,3-beta-D-glucan-UDP glucosyltransferase** *(Exophiala mesophila)* | 9.090 | 87.8 | 0.0 | Plasma membrane | 0.93 | | EC:2.4.1.34 | 0.893 | | 2.26E-02 |  |
| CF317_001401-T1 | A0A0D2ARD5_9EURO | **t-SNARE coiled-coil homology domain-containing protein** *(Exophiala oligosperma)* | 937 | 57 | 1.8E-123 | Plasma membrane | 0.75 | | - | 0.843 | | 4.08E-02 |  |
| CF317_004635-T1 | U1GGM2_ENDPU | **Clr5 domain-containing protein** (*Endocarpon pusillum* Z07020) | 1.503 | 52.7 | 0.0 | Nucleus | 1 | | - | 0.814 | | 4.34E-02 |  |
| CF317_004588-T1 | H6C927_EXODN | Alpha,alpha-trehalase *(Exophiala dermatitidis CBS 525.76)* | 3.189 | 58 | 0.0 | Extracellular space | 0.99 | | EC:3.2.1.28 | 0.813 | | 2.42E-02 |  |
| CF317_003037-T1 | H6BN96_EXODN | Acyl-CoA-dependent ceramide synthase *(Exophiala dermatitidis CBS 52576)* | 1.613 | 68.7 | 0.0 | Mitochondrial membrane | 0.81 | | EC:2.3.1.24 | 0.797 | | 1.16E-03 |  |
| CF317_008030-T1 | A0A0D1YYX4_9EURO | **D-amino-acid oxidase domain-containing protein** *(Exophiala sideris)* | 1.042 | 60.6 | 6.6E-140 | Extracellular space | 1 | | EC:1.4.3.3 | 0.783 | | 1.10E-02 |  |
| CF317_007665-T1 | A0A0D2CV56_9EURO | Guanine nucleotide-binding protein subunit beta *(Cladophialophora immunda)* | 1.810 | 95.2 | 0.0 | Nucleus | 1 | | - | 0.775 | | 2.98E-03 |  |
| CF317_003734-T1 | A0A0D2EUP4_9EURO | **Ribosomal_L23eN domain-containing protein** *(Exophiala xenobiotica)* | 680 | 86.3 | 2.1E-90 | Nucleus | 1 | | - | 0.771 | | 4.09E-02 |  |
| CF317_003823-T1 | A0A1C1C932_9EURO | Putative carrier protein pet8 protein *(Cladophialophora carrionii)* | 222 | 43.9 | 5.4E-22 | Mitochondrion | 0.97 | | - | 0.760 | | 7.17E-03 |  |
| CF317_006800-T1 | A0A0D2D5R0_9EURO | **Aa_trans domain-containing protein** *(Exophiala oligosperma)* | 2.325 | 72 | 0.0 | Plasma membrane | 0.86 | | - | 0.755 | | 3.54E-03 |  |
| CF317_004472-T1 | A0A0D2AKG4_9EURO | **Endoplasmic reticulum transmembrane protein** *(Exophiala oligosperma)* | 791 | 72.4 | 1.7E-105 | Endomembrane system | 0.89 | | - | 0.742 | | 4.09E-04 |  |
| CF317_005931-T1 | A0A0D2D4H8_9EURO | **Aspartate--tRNA ligase** *(Exophiala oligosperma)* | 3.007 | 62.2 | 0.0 | Cytoplasm | 1 | |  | 0.742 | | 2.84E-02 |  |
| CF317_009248-T1 | W9X9W8_9EURO | Isovaleryl-CoA dehydrogenase *(Cladophialophora psammophila CBS 110553)* | 1.691 | 83.1 | 0.0 | Cytoplasm | 0.7 | | - | | 0.740 | 2.63E-02 | |
| CF317_002505-T1 | A0A1C1C7M4_9EURO | Gamma interferon inducible lysosomal thiol reductase GILT *(Cladophialophora carrionii)* | 748 | 58.6 | 3.6E-97 | Extracellular space | 0.59 | | - | | 0.699 | 1.88E-02 | |
| CF317_006969-T1 | A0A177FDD4_9EURO | **HRXXH domain-containing protein** *(Fonsecaea monophora)* | 976 | 63 | 3.7E-131 | Extracellular space | 1 | | - | | 0.696 | 4.88E-02 | |
| CF317_002546-T1 | A0A423WI20_9PEZI | **HET domain-containing protein** *(Valsa malicola)* | 525 | 63.2 | 4.5E-61 | Extracellular space | 1 | | - | | 0.684 | 1.96E-02 | |
| CF317_001440-T1 | A0A3M7MUU7_9EURO | **C2 domain-containing protein** *(Chaetothyriales sp. CBS 135597)* | 3.065 | 52.5 | 0.0 | Cytoplasm | 0.7 | | EC:3.6.1.3; EC:3.6.1.15 | | 0.588 | 3.77E-02 | |
| CF317_006347-T1 | A0A1C1C7L8_9EURO | Inorganic phosphate transport protein PHO88 *(Cladophialophora carrionii)* | 811 | 81.4 | 2.6E-109 | Organelle membrane | 0.86 | | - | | 0.588 | 4.10E-02 | |
| CF317_002531-T1 | A0A0N1NY53_9EURO | Putative inorganic phosphate transporter C8E4.01c *(Phialophora attinorum)* | 2.647 | 75.5 | 0.0 | Plasma membrane | | 0.73 | - | | -0.591 | 1.01E-03 | |
| CF317_003425-T1 | A0A507QRN3_MONPU | Uncharacterized protein *(Monascus purpureus)* | 368 | 60.2 | 4.5E-44 | Mitochondrion | | 1 | - | | -0.603 | 4.74E-03 | |
| CF317_000059-T1 | A0A4U0XGJ4_9PEZI | **PSDC domain-containing protein** *(Cryomyces minteri)* | 1.499 | 61.6 | 0.0 | Cytoplasm | | 1 | EC:4.1.1.65 | | -0.634 | 7.14E-03 | |
| CF317_004317-T1 | A0A0D1Z0W5_9EURO | **3'(2'),5'-bisphosphate nucleotidase** *(Exophiala sideris)* | 1.542 | 85 | 0.0 | Extracellular space | | 1 | EC:3.1.3.7; EC:3.1.3.57; EC:3.1.3.31 | | -0.650 | 4.52E-02 | |
| CF317_003885-T1 | A0A1C1C894_9EURO | Putative RNA-binding protein *(Cladophialophora carrionii)* | 1.648 | 79.6 | 0.0 | Cytoplasm | | 0.7 | EC:2.3.1.5 | | -0.668 | 4.83E-03 | |
| CF317_000247-T1 | A0A0D2FR36_9EURO | **Formate dehydrogenase** *(Phialophora americana)* | 1.876 | 89.5 | 0.0 | Mitochondrion | | 0.87 | EC:1.17.1.9 | | -0.675 | 7.30E-04 | |
| CF317_002654-T1 | A0A0N1P280_9EURO | Scytalone dehydratase *(Phialophora attinorum)* | 807 | 78.5 | 1.1E-108 | Cytoplasm | | 1 | EC:4.2.1.94 | | -0.679 | 3.70E-02 | |
| CF317_006992-T1 | A0A178C5N3_9EURO | **Serine/threonine-protein kinase** *(Fonsecaea multimorphosa)* | 1.067 | 52.4 | 8.8E-131 | Cytoplasm | | 1 | - | | -0.682 | 2.90E-02 | |
| CF317_002925-T1 | A0A0D1WC07_9EURO | **Proteasome subunit alpha type** *(Exophiala sideris)* | 1.314 | 96.3 | 0.0 | Cytoplasm | | 1 | EC:3.4.99; EC:3.4.25 | | -0.683 | 8.77E-03 | |
| CF317_008191-T1 | A0A1C1D2P1_9EURO | Putative 4-hydroxy-2-oxoglutarate aldolase, mitochondrial *(Cladophialophora carrionii)* | 1.230 | 72.8 | 1E-168 | Cytoplasm | | 1 | - | | -0.699 | 6.28E-03 | |
| CF317_001861-T1 | A0A072PSJ3_9EURO | 40S ribosomal protein S20 *(Exophiala aquamarina CBS 119918)* | 568 | 97.4 | 1.2E-74 | Nucleus | | 1 | - | | -0.709 | 4.62E-03 | |
| CF317_003570-T1 | W2RN32_9EURO | **S-formylglutathione hydrolase***(Cyphellophora europaea*CBS 101466*)* | 1.334 | 83.4 | 0.0 | Extracellular space | | 0.95 | EC:3.1.2.12; EC:3.1.1.1 | | -0.720 | 1.69E-02 | |
| CF317_008979-T1 | W2RR18_9EURO | **Epimerase domain-containing protein** *(Cyphellophora europaea* CBS 101466) | 1.344 | 77.8 | 0.0 | Extracellular space | | 1 | EC:1.1.1.265; EC:1.2.1.5; EC:1.2.1.4; EC:1.1.1.184 | | -0.727 | 2.36E-02 | |
| CF317_008924-T1 | W2S109_9EURO | **Homogentisate 1,2-dioxygenase** (Cyphellophora europaea CBS 101466) | 2.182 | 81.7 | 0.0 | Extracellular space | | 1 | EC:1.13.11; EC:1.13.11.5 | | -0.730 | 4.76E-02 | |
| CF317_005516-T1 | A0A0D1XDH6_9EURO | 60S ribosomal protein L7 *(Exophiala sideris)* | 1.200 | 89.3 | 1.6E-166 | Nucleus | | 1 | - | | -0.734 | 1.03E-03 | |
| CF317_003714-T1 | A0A0D1WRJ8_9EURO | **Mannitol-1-phosphate 5-dehydrogenase** *(Exophiala sideris)* | 1.635 | 79.4 | 0.0 | Cytoplasm | | 1 | EC:1.1.1.17 | | -0.739 | 9.36E-05 | |
| CF317_007808-T1 | A0A0D1WXA6_EXOME | **NTF2 domain-containing protein** *(Exophiala mesophila)* | 515 | 77.4 | 2.9E-66 | Cytoplasm | | 0.7 | - | | -0.756 | 2.95E-02 | |
| CF317_005306-T1 | A0A2T2N8K6_CORCC | HAD-like protein *(Corynespora cassiicola* Philippines) | 995 | 76.3 | 2E-135 | Cytoplasm | | 0.7 | - | | -0.776 | 5.21E-04 | |
| CF317_008448-T1 | W2RU25_9EURO | **Phosphotransferase** *(Cyphellophora europaea CBS 101466)* | 1.902 | 72.8 | 0.0 | Cytoplasm | | 1 | EC:2.7.1.7; EC:2.7.1.4; EC:2.7.1.2; EC:2.7.1.1 | | -0.790 | 4.34E-04 | |
| CF317_004501-T1 | A0A0D2F446_9EURO | **40S ribosomal protein S24** *(Exophiala xenobiotica)* | 632 | 92.5 | 9.5E-84 | Mitochondrion | | 1 |  | | -0.796 | 1.53E-02 | |
| CF317_006512-T1 | A0A072PPW0_9EURO | **NTP_transf_9 domain-containing protein** *(Exophiala aquamarina CBS 119918)* | 993 | 64 | 4.6E-134 | Mitochondrion | | 1 | - | | -0.800 | 1.29E-03 | |
| CF317_003513-T1 | H6BNI5_EXODN | **2-phosphoglycerate dehydratase** *(Exophiala dermatitidis CBS 525.76)* | 2.067 | 89.5 | 0 | Cytoplasm | | 1 | EC:4.2.1.11; EC:2.3.2.13 | | -0.870 | 1.53E-03 | |
| CF317_009346-T1 | A0A4U7B682_9PEZI | Serine/threonine-protein kinase-like protein 3 *(Elsinoe australis)* | 222 | 69.4 | 1E-19 | Mitochondrion | | 0.58 | - | | -0.871 | 1.94E-02 | |
| CF317_006165-T1 | H6C9K5_EXODN | Esterase/lipase *(Exophiala dermatitidis CBS 525.76)* | 976 | 72.1 | 3.2E-132 | Cytoplasm | | 1 | - | | -0.917 | 9.78E-03 | |
| CF317_003202-T1 | G3JMK8_CORMM | Concanavalin A-like lectin/glucanase *(Cordyceps militaris* CM01*)* | 1.038 | 56 | 1.6E-138 | Cytoplasm | | 0.7 | - | | -0.918 | 4.79E-02 | |
| CF317_009408-T1 | A0A1C1CTV9_9EURO | Cyanamide hydratase *(Cladophialophora carrionii)* | 711 | 56.7 | 3.9E-92 | Cytoplasm | | 0.94 | - | | -0.921 | 3.78E-03 | |
| CF317_005481-T1 | A0A0N0NLG1_9EURO | **6-phosphogluconate dehydrogenase, decarboxylating** *(Phialophora attinorum)* | 2.384 | 89.2 | 0.0 | Cytoplasm | | 1 | EC:1.1.1.44 | | -0.960 | 7.57E-04 | |
| CF317_000980-T1 | A0A0D2CV88_9EURO | **Thioredoxin domain-containing protein** *(Cladophialophora immunda)* | 530 | 75 | 8.3E-68 | Cytoplasm | | 1 | EC:1.11.1; EC:1.11.1.7 | | -0.983 | 2.08E-02 | |
| CF317_001758-T1 | A0A178ZL52_9EURO | **Citrate synthase** *(Fonsecaea erecta)* | 2.218 | 89.9 | 0.0 | Mitochondrion | | 0.97 | EC:2.3.3.1 | | -1.005 | 4.76E-03 | |
| CF317_005124-T1 | A0A178C1A2_9EURO | Spermidine synthase *(Fonsecaea multimorphosa)* | 1.424 | 90.8 | 0.0 | Cytoplasm | | 1 | EC:2.5.1.16; EC:3.6.1.15 | | -1.020 | 2.48E-03 | |
| CF317_001933-T1 | H6C3E2_EXODN | **CMD domain-containing protein***(Exophiala dermatitidis CBS 525.76)* | 543 | 59.7 | 2.7E-68 | Nucleus | | 1 |  | | -1.064 | 5.94E-03 | |
| CF317_002908-T1 | A0A1C1CAZ9_9EURO | Conidial pigment biosynthesis protein Ayg1 *(Cladophialophora carrionii)* | 1.760 | 76.6 | 0.0 | Cytoplasm | | 1 | EC:3.6.1.3; EC:3.4.21.54;  EC:3.4.21.62 | | -1.069 | 5.16E-04 | |
| CF317_003302-T1 | A0A4U7B707_9PEZI | Transferrin receptor-like dimerization domain-containing protein *(Elsinoe australis)* | 2.536 | 77.5 | 0.0 | Cytoplasm | | 0.7 | EC:3.4.17.11 | | -1.082 | 1.07E-02 | |
| CF317_001633-T1 | B8MNS2_TALSN | **Histone H2A** *(Talaromyces stipitatus ATCC 10500)* | 650 | 97.7 | 1.3E-86 | Mitochondrion | | 1 | - | | -1.084 | 9.78E-03 | |
| CF317_000574-T1 | W9XSL0_9EURO | **Cytosolic nonspecific dipeptidase (**Cladophialophora psammophila CBS 110553) | 2.215 | 86.2 | 0.0 | Cytoplasm | | 1 | EC:3.4.13; EC:3.4.13.18; EC:3.4.19.9; EC:3.4.19 | | -1.086 | 2.26E-03 | |
| CF317_008862-T1 | A0A178ZQK6_9EURO | **6,7-dimethyl-8-ribityllumazine synthase** *(Fonsecaea erecta)* | 944 | 80.9 | 2.2E-128 | Cytoplasm | | 0.7 | EC:2.5.1.78 | | -1.087 | 6.05E-05 | |
| CF317_006154-T1 | W9Y727_9EURO | **Pyruvate decarboxylase** *(Capronia coronata CBS 617.96)* | 1.980 | 68 | 0.0 | Cytoplasm | | 1 | EC:2.2.1.6 | | -1.138 | 6.87E-03 | |
| CF317_004751-T1 | W2RJE3_9EURO | **Phosphoglycerate kinase** *(Cyphellophora europaea CBS 101466)* | 1.900 | 88.5 | 0.0 | Cytoplasm | | 1 | EC:2.7.2.3 | | -1.143 | 8.27E-04 | |
| CF317_005766-T1 | A0A178CPL1_9EURO | **Aldedh domain-containing protein** *(Fonsecaea multimorphosa)* | 1.743 | 68.7 | 0.0 | Cytoplasm | | 1 | - | | -1.172 | 8.22E-04 | |
| CF317_003229-T1 | A0A194X6D2_9HELO | Putative versicolorin reductase *(Phialocephala scopiformis)* | 788 | 61 | 8.8E-103 | Cytoplasm | | 1 | - | | -1.176 | 2.61E-03 | |
| CF317_007960-T1 | A0A0D1YWP7_9PEZI | 60S acidic ribosomal protein P1 *(Verruconis gallopava)* | 479 | 86.8 | 3.4E-61 | Extracellular space | | 1 | - | | -1.277 | 9.18E-03 | |
| CF317_007096-T1 | A0A0U1M0E3_TALIS | 6-phosphofructo-2-kinase *(*Penicillium islandicum*)* | 463 | 37.6 | 4.1E-49 | Nucleus | | 1 | - | | -1.494 | 5.59E-03 | |
| CF317_008154-T1 | H6C3S8_EXODN | Alcohol dehydrogenase *(Exophiala dermatitidis CBS 525.76)* | 1.336 | 70.9 | 0.0 | Cytoplasm | | 1 | - | | -1.632 | 8.10E-03 | |
| CF317_008933-T1 | H6BVG8_EXODN | Elongation factor EF-1 beta subunit *(Exophiala dermatitidis CBS 525.76)* | 914 | 74.3 | 9.2E-124 | Cytoplasm | | 0.7 | - | | -1.706 | 2.09E-02 | |
| CF317_007147-T1 | A0A178C9G0_9EURO | **Nucleoside diphosphate kinase** *(Fonsecaea multimorphosa)* | 743 | 90.3 | 4.1E-100 | Cytoplasm | | 1 | EC:2.7.4.6 | | -1.745 | 8.70E-04 | |
| CF317_006677-T1 | A0A0D2CVP1_9EURO | **HIT domain-containing protein** *(Cladophialophora immunda)* | 559 | 76.7 | 1.1E-72 | Cytoplasm | | 0.73 | - | | -1.770 | 8.90E-03 | |
| CF317_000379-T1 | H6BY84_EXODN | Elongation factor EF-1 gamma subunit *(Exophiala dermatitidis CBS 525.76)* | 1.704 | 76.7 | 0.0 | Cytoplasm | | 1 | EC:2.5.1.18; EC:3.1.4.17 | | -1.838 | 2.56E-03 | |
| CF317_007359-T1 | A0A0D2F5Y1_9EURO | Cytochrome c *(Phialophora americana)* | 571 | 92.9 | 3.2E-75 | Nucleus | | 1 | - | | -1.926 | 1.11E-03 | |

^a^ Protein accession number in the *K. chersonesos* database of *ab initio* translated proteins

^b^Up- and down-regulation of proteins (expressed as log2fold change) detected in the LSSMG-exposed secretome of *K. chersonesos* Mut compared to the unexposed secretome.

**Supplementary Table 8_**List of the most up- and down-regulated proteins detected in *K. chersonesos* Wt and Mut whole-cell proteome as revealed by comparative analysis of all samples. Proteins found at all experimental conditions are highlighted in bold. The hyphen (-) indicates that the protein was not detected amongst the 10 most differentially regulated proteins at the given experimental condition.

| **Protein accession number ^a^** | **UniProtKb Name** | **UniProtKb accession number** | **Down-regulation** | | **Up-regulation** | |
| --- | --- | --- | --- | --- | --- | --- |
|  | | | **1G** | **LSSMG** | **1G** | **LSSMG** |
| CF317_002712-T1 | Ribosomal Protein L32 | A0A0D2EEJ7_9EURO | **Wt** | **Wt** | **Mut** | **Mut** |
| CF317_007709-T1 | Ribosomal Protein L36 | A0A1C1CK07_9EURO | **Wt** | **Wt** | **Mut** | **Mut** |
| CF317_007955-T1 | NmrA domain-containing protein | A0A0D1XL03_9PEZI | Wt | Wt | - | Mut |
| CF317_005321-T1 | 40S ribosomal protein S30 | A0A6A6AL45_9PLEO | **Wt** | **Wt** | **Mut** | **Mut** |
| CF317_001622-T1 | U1 small nuclear ribonucleoprotein C | A0A179A0T0_9EURO | **Wt** | **Wt** | **Mut** | **Mut** |
| CF317_007794-T1 | **Cysteine dioxygenase** | A0A3M7NY82_9EURO | Wt | Wt | - | Mut |
| CF317_003555-T1 | RRM domain-containing protein | W9Y725_9EURO | Wt | - | - | Mut |
| CF317_000935-T1 | Ribosomal_L28e domain-containing protein | A0A177EQW0_9EURO | **Wt** | **Wt** | **Mut** | **Mut** |
| CF317_007284-T1 | Uncharacterized protein | W9WGT8_9EURO | Wt | - | - | - |
| CF317_000197-T1 | NADH dehydrogenase ubiquinone 1 alpha subcomplex subunit | A0A178C0C7_9EURO | Wt | Wt | Mut | - |
| CF317_004524-T1 | Ribosomal protein L37a | A0A072PAA2_9EURO | - | Wt | Mut | - |
| CF317_000101-T1 | 40S ribosomal protein S21 | A0A0D2H943_XYLBA | - | Wt | Mut | - |
| CF317_005549-T1 | HABP4_PAI-RBP1 domain-containing protein | A0A0D2D8I3_9EURO | - | - | Mut | - |
| CF317_008166-T1 | Translation machinery associated tma 7 | A0A166NDY3_9PEZI | - | - | Mut | - |
| CF317_007362-T1 | Uncharacterized protein | A0A0D2B6Z7_9EURO | **-** | **-** | **-** | Mut |
| CF317_007965-T1 | Uncharacterized protein | A0A438NEL9_EXOME | - | - | - | Mut |
| CF317_002368-T1 | Pc13g08300 protein | B6H4B7_PENRW | Mut | Mut | Wt | - |
| CF317_007621-T1 | Cutinase | A0A0D2AG04_9PEZI | Mut | Mut | Wt | - |
| CF317_003479-T1 | **Centrosomal protein of 19 kDa** | A0A0L0SYM3_ALLM3 | Mut | Mut | Wt | - |
| CF317_004369-T1 | Alpha-galactosidase | A0A2H3IQM1_9EURO | Mut | Mut | Wt | - |
| CF317_004176-T1 | AAA domain-containing protein | A0A409XRE1_PSICY | - | Mut | Wt | - |
| CF317_008131-T1 | Arabinan endo-1,5-alpha-L-arabinosidase | A0A1L9R418_ASPWE | - | - | Wt | - |
| CF317_001497-T1 | Uncharacterized protein | A0A067MPR6_9AGAM | - | - | Wt | - |
| CF317_009090-T1 | Spindle assembly checkpoint component MAD1 | B8M229_TALSN | - | - | Wt | - |
| CF317_008822-T1 | HET-domain-containing protein | A0A2V1C428_9HELO | - | - | Wt | - |
| CF317_006383-T1 | Murein transglycosylase | A0A072PG02_9EURO | **Mut** | **Mut** | **Wt** | **Wt** |
| CF317_004099-T1 | Deacylase-like protein | F9XH01_ZYMTI | Mut | - | - | Wt |
| CF317_003266-T1 | Malate dehydrogenase | A0A1C1CNC5_9EURO | Mut | - | - | Wt |
| CF317_007265-T1 | Inorganic diphosphatase | A0A2G5HZC2_CERBT | - | Mut | - | Wt |
| CF317_004400-T1 | Lipocin_cytosolic_FA-bd_dom domain-containing protein | A0A0D1ZSU6_EXOME | Mut | - | - | Wt |
| CF317_006579-T1 | Alpha/beta-glucosidase agdC | A0A178BWA1_9EURO | - | - | - | Wt |
| CF317_005867-T1 | Urea amidohydrolase | A0A3M2T8E7_9EURO | - | - | - | Wt |
| CF317_003773-T1 | Zinc-regulated transporter 1 | A0A0N1HPS7_9EURO | - | - | - | Wt |
| CF317_007869-T1 | Adaptin_N domain-containing protein | A0A3M7NMJ9_9EURO | - | - | - | Wt |
| CF317_001423-T1 | Uncharacterized protein | A0A135TBW5_9PEZI | - | - | - | Wt |
| CF317_009191-T1 | Choline transport protein | A0A2P8A4S9_9PEZI | Mut | - | - | - |
| CF317_004099-T1 | Deacylase-like protein | F9XH01_ZYMTI | Mut | - | - | - |
| CF317_004960-T1 | Murein transglycosylase | W9X9S3_9EURO | Mut | - | - | - |
| CF317_002178-T1 | **Indoleamine 2,3-dioxygenase** | A0A365NCF7_GIBIN | - | Mut | - | - |
| CF317_002858-T1 | But2 domain-containing protein | A0A420YNS7_9PEZI | - | Mut | - | - |
| CF317_001542-T1 | POT family proton-dependent oligopeptide transporter | A0A072P870_9EURO | - | Mut | - | - |

**^a^** Protein accession number in the *K. chersonesos* database of ab initio translated proteins.

**Supplementary Table 9_**List of the most up- and down-regulated proteins detected in *K. chersonesos* Wt and Mut secretome as revealed by comparative analysis of all samples. Proteins found at all experimental conditions are highlighted in bold. The hyphen (-) indicates that the protein was not detected amongst the 10 most differentially regulated proteins at the given experimental condition.

| **Protein accession number ^a^** | **UniProtKb Name** | | **UniProtKb accession number** | | **Down-regulation** | | **Up-regulation** | |
| --- | --- | --- | --- | --- | --- | --- | --- | --- |
|  | | | | | **1G** | **LSSMG** | **1G** | **LSSMG** |
| CF317_007965-T1 | | Uncharacterized protein | | A0A438NEL9_EXOME | **Wt** | **Wt** | **Mut** | **Mut** |
| CF317_009675-T1 | | Uncharacterized protein | | W2RWQ2_9EURO | Wt | Wt | Mut | - |
| CF317_003655-T1 | | MFS domain-containing protein | | A0A0D1W809_9EURO | Wt;Mut | Mut | - | - |
| CF317_003654-T1 | | Uncharacterized protein | | H6BW33_EXODN | Wt | Wt | **-** | **-** |
| CF317_004702-T1 | | Quinoprotein amine dehydrogenase beta chain-like protein | | A0A179FUL4_PURLI | Wt | Wt | - | Mut |
| CF317_006512-T1 | | NTP_transf_9 domain-containing protein | | A0A072PPW0_9EURO | Wt | Wt | - | - |
| CF317_004653-T1 | | Carboxylic ester hydrolase | | A0A1L7WLC8_9HELO | Wt | - | - | - |
| CF317_001497-T1 | | Uncharacterized protein | | A0A067MPR6_9AGAM | Wt | Wt | Mut | - |
| CF317_004119-T1 | | Adenylosuccinate lyase | | A0A438NIP5_EXOME | Wt | - | - | - |
| CF317_004027-T1 | | Tyrosinase_Cu-bd domain-containing protein | | A0A438NAZO_EXOME | Wt | Wt | Mut | - |
| CF317_002949-T1 | | Feruloyl esterase | | A0A177BY00_9PLEO | - | Wt | - | - |
| CF317_001423-T1 | | Uncharacterized protein | | A0A135TBW5_9PEZI | - | Wt | - | - |
| CF317_000257-T1 | | **Ydc2-catalyt domain-containing protein** | | A0A3M7NIC9_9EURO | - | Wt | - | Mut |
| CF317_001911-T1 | | MFS transporter, SP family, major inositol transporter | | W9Z1V7_9EURO | Mut | Mut | - | Wt |
| CF317_005782-T1 | | Putative mfs phospholipid transporter | | A0A0G2E357_9EURO | Mut | - | - | Wt |
| CF317_006070-T1 | | 60S ribosomal protein L32 | | W9YNN4_9EURO | Mut | Mut | Wt | - |
| CF317_001879-T1 | | 60S ribosomal protein L35 | | W9WJM3_9EURO | **Mut** | **Mut** | **Wt** | **Wt** |
| CF317_001628-T1 | | SHSP domain-containing protein | | A0A3M7MQN8_9EURO | Mut | - | - | Wt |
| CF317_002664-T1 | | Plasma membrane ATPase | | A0A0D2I7E3_9EURO | Mut | Mut | - | Wt |
| CF317_005516-T1 | | 60S ribosomal protein L7 | | A0A0D1XDH6_9EURO | Mut | Mut | - | - |
| CF317_000200-T1 | | 40S ribosomal protein S23 | | W2RYK7_9EURO | Mut | Mut | - | - |
| CF317_003183-T1 | | 60S ribosomal protein L6 | | H6C818_EXODN | Mut | - | Wt | - |
| CF317_008155-T1 | | 40S ribosomal protein S7 | | A0A0D2B8I0_9EURO | - | - | - | Mut |
| CF317_002531-T1 | | Putative inorganic phosphatase transporter C8E4.01c | | A0A0N1Ny53_9EURO | - | Wt | - | Mut |
| CF317_009074-T1 | | Ribosomal protein L15 | | A0A3M7NVX6_9EURO | - | - | - | Mut |
| CF317_005595-T1 | | 60S ribosomal protein L33-A | | A0A178C7W2_9EURO | - | - | Wt | - |
| CF317_004524-T1 | | Ribosomal protein L37a | | A0A072PAA2_9EURO | - | - | Wt | - |
| CF317_001861-T1 | | 40S ribosomal protein S20 | | A0A072PSJ3_9EURO | - | - | Wt | - |
| CF317_002107-T1 | | 60S ribosomal protein L2 | | A0A0G2EU75_9EURO | - | - | Wt | - |
| CF317_002841-T1 | | 60S ribosomal protein L4-B | | H6BWQ6_EXODN) | - | - | Wt | - |
| CF317_008131-T1 | | Arabinan endo-1,5-alpha-L-arabinosidase | | A0A1L9R418_ASPWE | - | - | Wt | - |
| CF317_003042-T1 | | Ribosomal protein L14b/L23e | | A0A6G1FTI0_9PEZI | - | - | Wt | - |
| CF317_000354-T1 | | C2 domain-containing protein | | A0A4V1M521_TREME | - | - | - | Wt |
| CF317_009016-T1 | | MSF domain-containing protein | | W9VF20_9EURO | - | - | - | Wt |
| CF317_001090-T1 | | Allergen | | A0A1C1C6U8_9EURO | - | - | - | Wt |
| CF317_003063-T1 | | Uncharacterized protein | | A0A0D2AFI1_9EURO | - | - | - | Wt |
| CF317_006677-T1 | | HIT domain-containing protein | | A0A0D2CVP1_9EURO | - | - | Mut | - |
| CF317_008933-T1 | | Elongation factor EF-1 beta subunit | | H6BVG8_EXODN | - | - | Mut | - |
| CF317_007147-T1 | | Nucleoside diphosphate kinase | | A0A178C9GO_9EURO | - | - | Mut | - |
| CF317_001284-T1 | | Ornithine cyclodeaminase | | A0A443HNA0_BYSSP | - | - | Mut | - |
| CF317_000379-T1 | | Elongation factor EF-1 gamma subunit | | H6BY84_EXODN | - | - | Mut | - |
| CF317_007359-T1 | | Cytochrome c | | A0A0D2F5Y1_9EURO | - | - | Mut | - |
| CF317_002674-T1 | | Cell wall glucanase (Scw11) | | H6BUN2_EXODN | - | - | - | Mut |
| CF317_007601-T1 | | Protein SEY1 (EC) *(Ganoderma boninense)* | | A0A5K1K211_9APHY | - | - | - | Mut |
| CF317_004400-T1 | | Lipocin_cytosolic_FA-bd_dom domain-containing protein | | A0A0D1ZSU6_EXOME | - | - | - | Mut |
| CF317_003266-T1 | | Malate dehydrogenase | | A0A1C1CNC5_9EURO | - | - | - | Mut |
| CF317_009172-T1 | | Uncharacterized protein | | H6BZ20_EXODN | - | - | - | Mut |
| CF317_001805-T1 | | Uncharacterized protein | | W2RWQ2_9EURO | - | - | - | Mut |
| CF317_004551-T1 | | Carboxypeptidase | | A0A0D2GVY9_9EURO | - | - | - | Mut |

**^a^** Protein accession number in the *K. chersonesos* database of ab initio translated proteins.

**Supplementary Figure 3_**Top, most abundantly expressed and differentially regulated proteins in both *K. chersonesos* Wt and Mut at all experimental conditions. (A-F) Proteins regulated in the whole-cell proteome, (A) CF317_002712-T1, (B) CF317_007709-T1, (C) CF317_005321-T1, (D) CF317_000935-T1 (E) CF317_001622-T1, (F) CF317_006383-T1; (G-H) Proteins regulated in the secretome. (G) CF317_007965-T1, (H) CF317_001879-T1. Protein abundances are expressed as fold change. Proteins were evaluated for increased or decreased abundance using a cut-off value of ≥ ± 1.5-fold change and p value of ≤ 0.05.

**
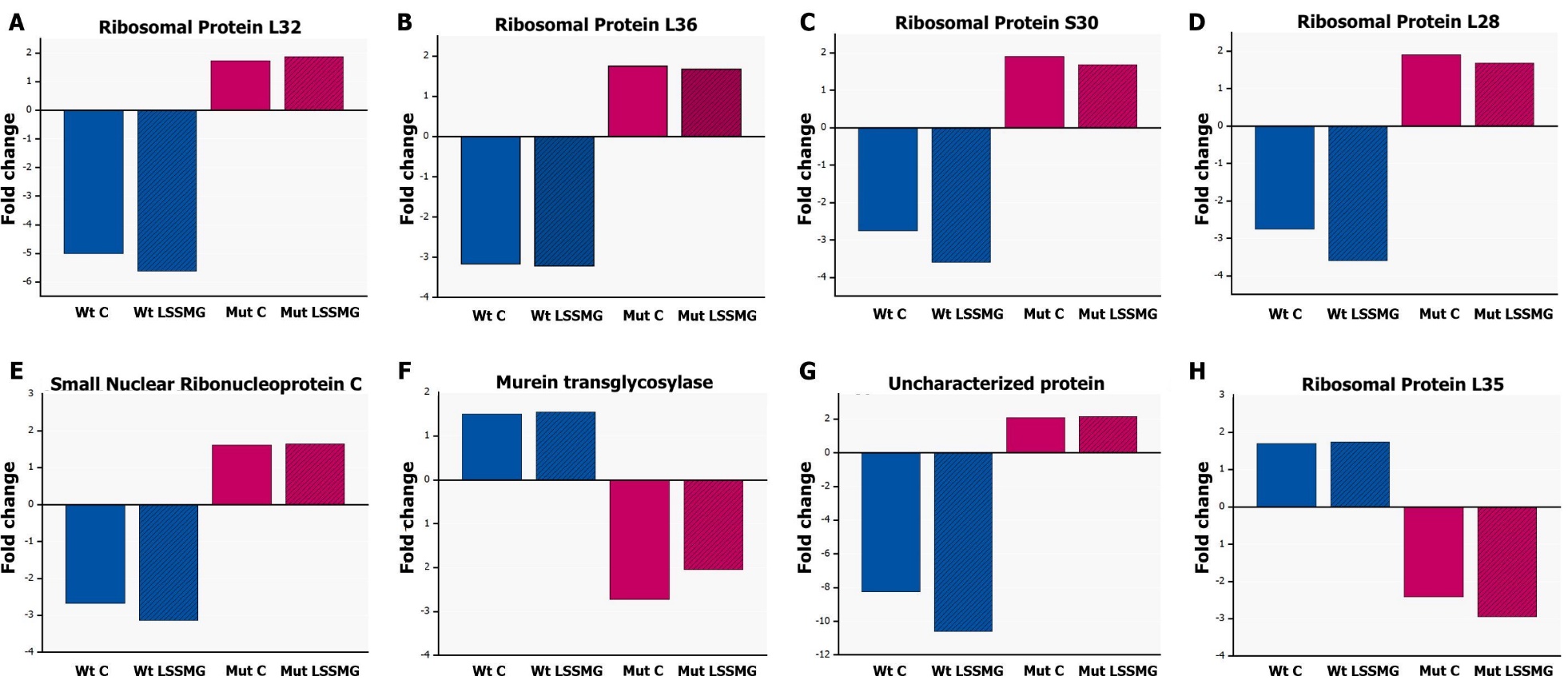
**

**Supplementary Figure 4_**Protein-protein interaction analysis of the 8 most abundant proteins common to *K. chersonesos* wild type and mutant at all experimental conditions. Due to its phylogenetic proximity to *K. chersonesos* (Tesei et al., 2020a), the proteome of the black yeast *Exophiala dermatitidis* was selected as reference database for homology-based searches. (A) Protein 6-node network including only the proteins which matched protein homologs in the STRING database. U1 small nuclear ribonucleoprotein C/CF317_001622-T1, homolog of XP_009159253.1; ribosomal protein L35/CF317_001879-T1, homolog of XP_009155966.1; ribosomal protein S30/CF317_005321-T1, homolog of XP_009156842.1; murein transglycosylase/CF317_006383-T1 homolog of XP_009152794.1; uncharacterized protein/CF317_007965-T1 homolog of XP_009158195.1; ribosomal protein L36/CF317_007709-T1, homolog of XP_009160183.1. An interaction was found between proteins CF317_001879-T1 and CF317_005321-T1. Protein 6-node network with highlighted all ribonucleoproteins (B) and (C) ribosomal proteins, based on annotated keywords (UniProt). (D) Outcome of the statistical and functional enrichment analysis. Strength: Log10 (observed/expected); FDR: p-values were corrected for multiple testing within each category using the Benjamini-Hochberg procedure. The images were obtained using STRING v. 11.0 (<https://www.string-db.org>).

**
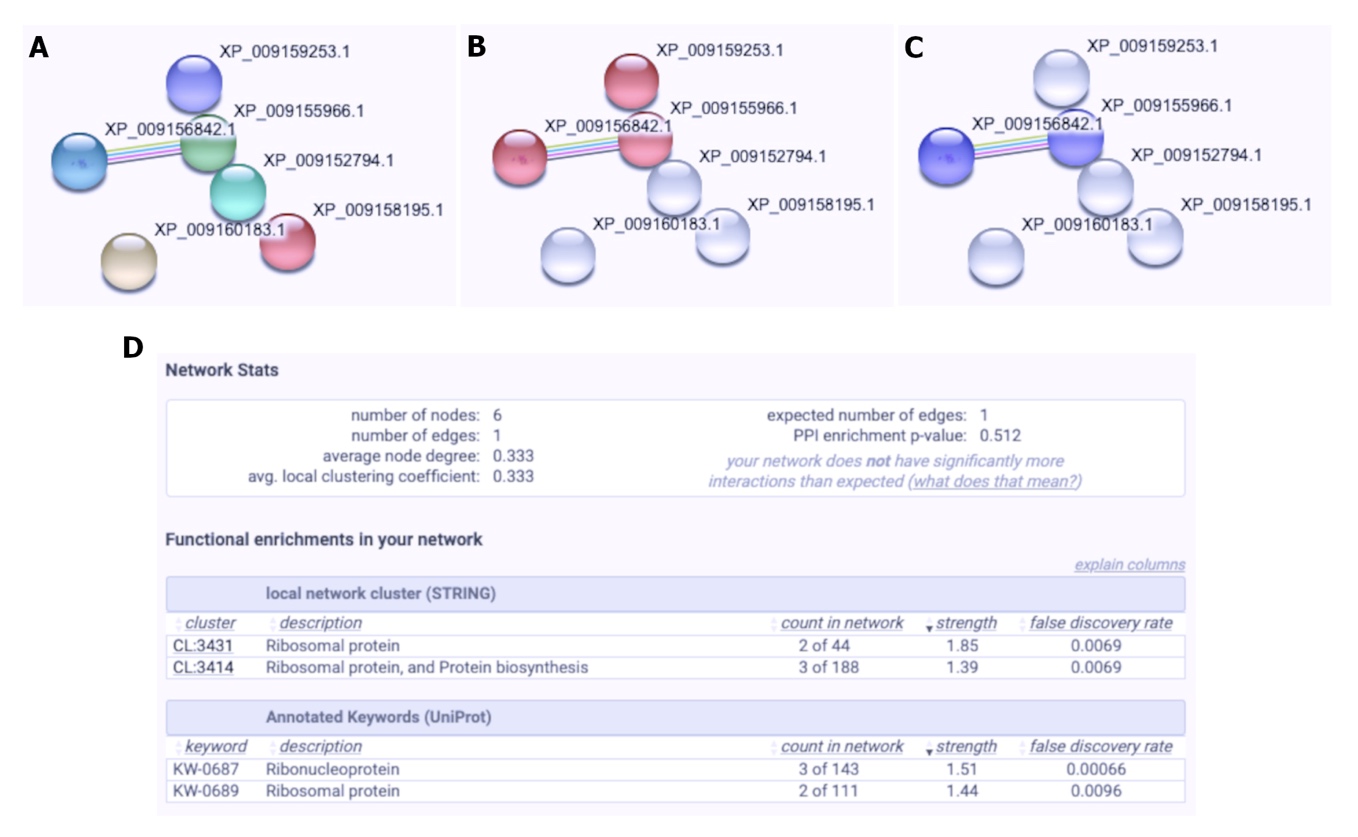
**

**Supplementary Figure 5_**Biological processes GOs categories of differentially expressed proteins in *K. chersonesos* Mut as compared to the wild type, under normal gravity conditions. (A) Whole-cell proteome (B) Secretome. Proteins with changed abundance (FC ≥ ± 1.5, p≤ 0.05) were annotated with terms representing various biological processes using OmicsBox v. 1.4.11 (<https://www.biobam.com>). GO terms were thereafter summarized using sequence distribution/GO multilevel pie charts (Filtered by sequence count, Cutoff=2). Other: BP GO terms with Cutoff <2.

**
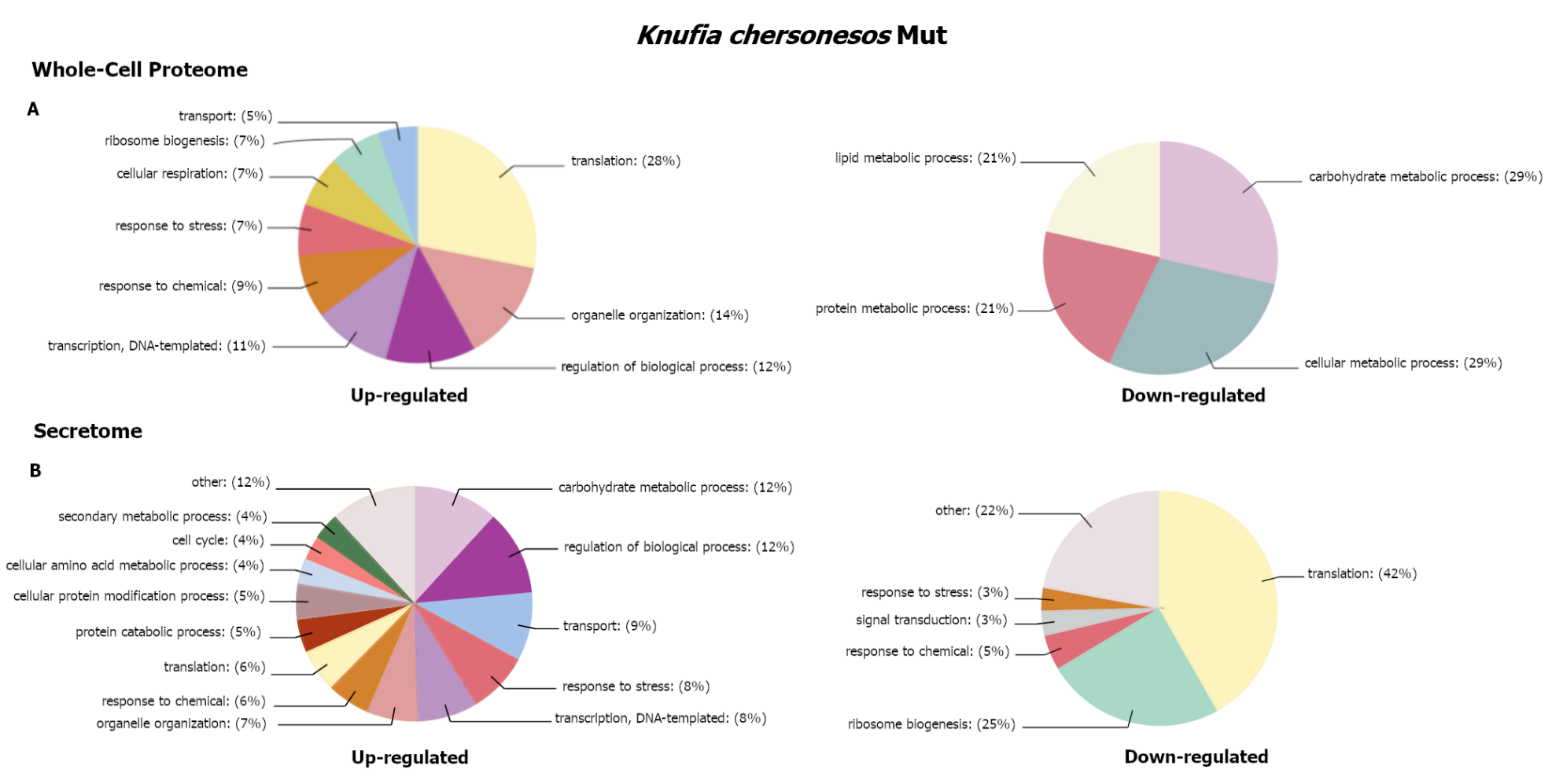
**

**Supplementary Table 10_**Homologues of differentially abundant proteins in *K. chersonesos* Mut whole-cell proteome under normal gravity (1G) as compared to the wild type (BLASTP algorithm). Only the matching proteins with known identity (when available) and the highest max score are displayed. The predicted protein subcellular localizations are also shown.

| ***Protein* accession No. ^a^** | **UniProtKb accession**  **No.** | **Protein name** | **Max**  **Score** | **Identity**  **(%)** | **Expected**  **value** | **Predicted Localization** | **BUSCA Score** | **KEGG Enzymes code** | **Mut/**  **Wt**  **FC** **^b^** | **p-value** |  |
| --- | --- | --- | --- | --- | --- | --- | --- | --- | --- | --- | --- |
| CF317_002712-T1 | A0A0D2EEJ7_9EURO | Ribosomal protein L32 (*Exophiala oligosperma*) | 372 | 63.7 | 1.2E-43 | Mitochondrion | 0.52 | **-** | 3.121 | 2.06E-03 | |
| CF317_007709-T1 | A0A1C1CK07_9EURO | 50S Ribosomal Protein L36 (*Cladophialophora carrionii)* | 531 | 58.2 | 1.1E-66 | Mitochondrion | 0.73 | **-** | 2.475 | 3.24E-04 | |
| CF317_005321-T1 | A0A6A6AL45_9PLEO | **40S ribosomal protein S30** *(Dothidotthia symphoricarpi* CBS 119687) | 324 | 93.5 | 1.3E-42 | Mitochondrion | 1 | - | 2.384 | 7.30E-04 | |
| CF317_007955-T1 | A0A0D1XL03_9PEZI | **NmrA domain-containing protein** (*Verruconis gallopava)* | 764 | 51.5 | 1.7E-98 | Cytoplasm | 1 | - | 2.210 | 3.74E-04 | |
| CF317_001622-T1 | A0A179A0T0_9EURO | **U1 small nuclear ribonucleoprotein C** *(Fonsecaea erecta)* | 557 | 61.6 | 8.9E-71 | Extracellular space | 1 | - | 2.116 | 5.39E-05 | |
| CF317_000935-T1 | A0A177EQW0_9EURO | Ribosomal_L28e domain-containing protein *(Fonsecaea monophora)* | 523 | 65.1 | 1.2E-66 | Mitochondrion | 1 | - | 1.991 | 4.66E-03 | |
| CF317_003555-T1 | W9Y725_9EURO | RRM domain-containing protein (*Capronia epimyces CBS 606.96*) | 869 | 70.3 | 2.7E-115 | Extracellular space | 1 | - | 1.922 | 1.47E-04 | |
| CF317_004524-T1 | A0A072PAA2_9EURO | Ribosomal protein L37a (*Exophiala aquamarina* CBS 119918) | 464 | 96.7 | 1.5E-59 | Cytoplasm | 0.7 | - | 1.849 | 1.27E-03 | |
| CF317_007794-T1 | A0A3M7NY82_9EURO | **Cysteine dioxygenase** (Chaetothyriales sp. CBS 134916) | 671 | 64.6 | 6E-87 | Cytoplasm | 0.7 | EC:1.13.11; EC:1.13.11.20 | 1.773 | 5.07E-04 | |
| CF317_000197-T1 | A0A178C0C7_9EURO | NADH dehydrogenase ubiquinone 1 alpha subcomplex subunit *(Fonsecaea multimorphosa)* | 616 | 76.8 | 1.2E-80 | Mitochondrion | 1 | EC:7.1.1.2; EC:1.6.5.11; EC:1.6.99.3 | 1.754 | 5.53E-04 | |
| CF317_000101-T1 | A0A0D2H943_XYLBA | 40S ribosomal protein S21 (Cladophialophora bantiana CBS 173.52**)** | 430 | 93.2 | 1.8E-54 | Nucleus | 1 | - | 1.692 | 1.92E-03 | |
| CF317_0005549-T1 | A0A0D2D8I3_9EURO | HABP4_PAI-RBP1 domain-containing protein | 942 | 53.4 | 1E-123 | Cytoplasm | 0.7 | - | 1.692 | 1.11E-03 | |
| CF317_0007844-T1 | H6BRC5_EXODN | Adenosinetriphosphatase (*Exophiala dermatitidis* CBS 525.76) | 6.958 | 71.2 | 0.0 | Cytoplasm | 0.7 | EC:3.6.1.3; EC:3.6.1.15 | 1.586 | 2.35E-04 | |
| CF317_0008166-T1 | A0A166NDY3_9PEZI | Translation machinery associated tma7 *(Colletotrichum incanum)* | 300 | 82.4 | 2.9E-38 | Cytoplasm | 0.7 | - | 1.554 | 4.74E-04 | |
| CF317_0007284-T1 | W9WGT8_9EURO | Uncharacterized protein (Cladophialophora yegresii CBS 114405**)** | 322 | 63.9 | 2.3E-37 | Mitochondrion | 1 | - | 1.454 | 4.20E-02 | |
| CF317_0002966-T1 | A0A1C1CBA7_9EURO | G2/M phase checkpoint control protein Sum2 *(Cladophialophora carrionii)* | 1.520 | 58.1 | 0.0 | Nucleus | 1 | - | 1.412 | 1.32E-04 | |
| CF317_0007362-T1 | A0A0D2B6Z7_9EURO | Uncharacterized protein (*Exophiala spinifera*) | 701 | 49.8 | 3.9E-89 | Extracellular space | 1 | - | 1.399 | 1.27E-02 | |
| CF317_0004115-T1 | H6C3H9_EXODN | **Pyrid_ox_like domain-containing protein** *(Exophiala dermatitidis CBS 525.76)* | 743 | 69.7 | 1.5E-98 | Nucleus | 1 | - | 1.334 | 2.66E-03 | |
| CF317_0007853-T1 | A0A3M0WCJ6_9EURO | **Rick_17kDa_Anti domain-containing protein** *(Chaetothyriales sp. CBS 134920)* | 649 | 56.6 | 3.1E-83 | Cytoplasm | 0.7 | - | 1.329 | 7.88E-04 | |
| CF317_0004704-T1 | A0A072P8A9_9EURO | Uncharacterized protein (*Exophiala aquamarin*a CBS 119918) | 221 | 37.8 | 5.1E-20 | Nucleus | 1 | - | 1.287 | 2.10E-03 | |
| CF317_0006724-T1 | A0A0J6FT39_COCPO | Mycocerosic acid synthase *(*Coccidioides posadasii RMSCC 3488*)* | 91 | 29.7 | 7.9E-2 | Cytoplasm | 0.7 | - | 1.217 | 3.36E-03 | |
| CF317_0007406-T1 | W2S4F5_9EURO | **NmrA domain-containing protein** *(Cyphellophora europaea CBS* 101466*)* | 1.077 | 68.6 | 4.5E-146 | Extracellular space | 1 | - | 1.198 | 1.19E-02 | |
| CF317_0008038-T1 | A0A0H1B9E7_9EURO | **COX assembly mitochondrial protein** *(Blastomyces silverae)* | 443 | 58.4 | 3E-54 | Nucleus | 1 | - | 1.159 | 3.65E-04 | |
| CF317_002997-T1 | A0A0D1XFA2_9EURO | **COX assembly mitochondrial protein** (*Exophiala sideris)* | 508 | 82 | 1.5E-65 | Nucleus | 1 | - | 1.115 | 4.66E-03 | |
| CF317_007480-T1 | W9X9U0_9EURO | NADH dehydrogenase (*Capronia epimyces CBS 606.96)* | 481 | 68.1 | 1.2E-60 | Cytoplasm | 0.7 | - | 1.101 | 2.50E-03 | |
| CF317_009364-T1 | A0A0D2DG69_9EURO | Uncharacterized protein *(Exophiala oligosperma)* | 4720 | 41.0 | 8.1E-89 | Cytoplasm | 0.7 | - | 1.100 | 7.09E-04 | |
| CF317_001207-T1 | A0A0D2CU26_9EURO | Uncharacterized protein *(Exophiala xenobiotica)* | 1.101 | 80.5 | 6.3E-151 | Extracellular space | 1 | - | 1.096 | 3.10E-04 | |
| CF317_002698-T1 | W2SCX3_9EURO | **RRM domain-containing protein** *(Cyphellophora europaea* CBS 101466) | 770 | 73.2 | 4E-102 | Nucleus | 1 | - | 1.090 | 4.30E-03 | |
| CF317_008283-T1 | H6C4E3_EXODN | Laccase TilA *(Exophiala dermatitidis* CBS 525.76) | 1.948 | 49.4 | 0.0 |  |  | EC:1.10.3; EC:1.10.3.2 | 1.087 | 7.01E-04 | |
| CF317_001843-T1 | A0A1C1CGV8_9EURO | Putative formamidase *(Cladophialophora carrionii)* | 1.910 | 84.3 | 0.0 | Cytoplasm | 1 | EC:3.5.1.49 | 1.086 | 4.74E-04 | |
| CF317_003660-T1 | W9Y5Z7_9EURO | Uncharacterized protein (*Capronia coronata CBS 617.96)* | 733 | 45.7 | 3.8E-92 | Mitochondrion | 0.93 | - | 1.083 | 2.83E-04 | |
| CF317_009589-T1 | W9YT66_9EURO | \| **C2H2-type domain-containing protein**  (Capronia epimyces CBS 606.96) \|  \| \| --- \| --- \| | 947 | 59.3 | 3.7E-125 | Cytoplasm | 0.7 | - | 1.072 | 1.37E-02 | |
| CF317_002122-T1 | A0A438MWC1_EXOME | Uncharacterized protein *(Exophiala mesophila)* | 129 | 28.2 | 2.6E-7 | Nucleus | 1 | - | 1.031 | 9.60E-03 | |
| CF317_005435-T1 | A0A0D1VRZ4_9EURO | Uncharacterized protein *(Exophiala sideris)* | 497 | 66.7 | 5.1E-63 | Mitochondrion | 1 | - | 1.020 | 1.05E-02 | |
| CF317_004981-T1 | W9Y456_9EURO | Murein transglycosylase *(Capronia epimyces CBS 606.96)* | 1.889 | 54.5 | 0.0 | Extracellular space | 0.94 | - | 1.018 | 3.00E-03 | |
| CF317_002972-T1 | W9YW17_9EURO | **H/ACA ribonucleoprotein complex subunit NOP10** *(Capronia coronata CBS 617.96)* | 324 | 86.6 | 8.5E-42 | Nucleus | 1 | - | 1.010 | 1.29E-03 | |
| CF317_003870-T1 | A0A0D2HPT5_9EURO | **NADH dehydrogenase [ubiquinone] iron-sulfur protein 4, mitochondrial** *(Fonsecaea pedrosoi CBS 271.37)* | 704 | 63.1 | 2.3E-92 | Mitochondrion | 0.7 | - | 0.992 | 3.79E-03 | |
| CF317_004682-T1 | H6C176_EXODN | **37S ribosomal protein S25, mitochondrial** *(Exophiala dermatitidis*CBS 525.76) | 795 | 62.6 | 6.7E-104 | Cytoplasm | 0.7 | - | 0.981 | 1.44E-04 | |
| CF317_005054-T1 | A0A507QZB2_MONPU | **NADH dehydrogenase [ubiquinone] 1 beta subcomplex subunit 7** *(Monascus purpureus)* | 371 | 85.7 | 1.8E-49 | Nucleus | 1 | EC:7.1.1.2; EC:1.6.5.11; EC:1.6.99.3 | 0.979 | 1.11E-04 | |
| CF317_008325-T1 | U1GFX3_ENDPU | 60S ribosomal protein L38 *(Endocarpon pusillum* HMAS-L-300199) | 312 | 71.4 | 1.8E-39 | Cytoplasm | 0.7 | - | 0.973 | 5.61E-05 | |
| CF317_006017-T1 | A0A072PVN7_9EURO | Nuclear transcription factor Y, alpha *(Exophiala aquamarina CBS 119918)* | 779 | 71.1 | 4.8E-102 | Nucleus | 1 | - | 0.971 | 5.10E-03 | |
| CF317_005164-T1 | A0A1Y2TGI7_9PEZI | ABC-2 type transporter *(Hypoxylon sp. EC38)* | 5.961 | 74.8 | 0.0 | Endomembrane system | 0.83 | EC:3.6.1.3; EC:3.6.1.15; EC:7.6.2.2 | 0.952 | 4.98E-03 | |
| CF317_001529-T1 | V9DQD0_9EURO | Uncharacterized protein *(Cladophialophora carrionii CBS 160.54)* | 651 | 46.6 | 3E-81 | Mitochondrion | 1 | - | 0.951 | 1.25E-03 | |
| CF317_005328-T1 | A0A1C1D2N6_9EURO | Methyltransferase-like protein *(Cladophialophora carrionii)* | 574 | 44.3 | 1E-70 | Cytoplasm | 0.6 | - | 0.941 | 9.01E-06 | |
| CF317_002844-T1 | A0A4U0VAR5_9PEZI | **Enhancer of polycomb-like protein** *(Friedmanniomyces endolithicus)* | 157 | 36.3 | 1.2E-10 | Mitochondrion | 1 | - | 0.936 | 1.31E-03 | |
| CF317_004940-T1 | A0A3M2TAX9_9EURO | DUF1264 domain protein *(Aspergillus sp. HF37)* | 731 | 67.7 | 2.6E-96 | Cytoplasm | 0.7 | - | 0.935 | 9.20E-04 | |
| CF317_001221-T1 | H6BRE3_EXODN | 30S ribosomal protein S14 (Exophiala dermatitidis CBS 525.76) | 495 | 81.8 | 1.3E-63 | Mitochondrion | 1 | - | 0.909 | 5.73E-03 | |
| CF317_009591-T1 | A0A1C1CRD2_9EURO | C2H2 transcription factor *(Cladophialophora carrionii)* | 1.144 | 51.9 | 1.2E-144 | Cytoplasm | 0.7 | - | 0.906 | 2.67E-02 | |
| CF317_002768-T1 | A0A178ZLT2_9EURO | **HMG box domain-containing protein** *(Fonsecaea erecta)* | 614 | 45.4 | 6.9E-75 | Mitochondrion | 0.9 | - | 0.894 | 3.44E-02 | |
| CF317_006500-T1 | A0A2B7Y5E3_9EURO | 60S ribosomal protein L28 *(Polytolypa hystricis UAMH7299)* | 741 | 90.6 | 5.7E-100 | Mitochondrion | 1 | EC:3.6.4.13 | 0.878 | 4.96E-03 | |
| CF317_000265-T1 | A0A3A2Z7A8_9EURO | **Transcription factor RfeG** (Aspergillus sclerotialis) | 291 | 34.9 | 1.4E-27 | Cytoplasm | 0.7 | - | 0.869 | 5.47E-04 | |
| CF317_004027-T1 | A0A438NAZ0_EXOME | **Tyrosinase_Cu-bd domain-containing protein** *(Exophiala mesophila)* | 1.326 | 62.4 | 0.0 | Extracellular space | 0.84 | EC:1.14.18.1 | 0.867 | 1.42E-02 | |
| CF317_000225-T1 | A0A0D2HHJ9_9EURO | **Mediator of RNA polymerase II transcription subunit 19** *(Rhinocladiella mackenziei CBS 650.93)* | 457 | 34.2 | 1.5E-50 | Nucleus | 1 | - | 0.856 | 6.03E-03 | |
| CF317_007339-T1 | A0A0D1YRY9_9EURO | Uncharacterized protein *(Exophiala sideris)* | 776 | 54.7 | 2.4E-99 | Cytoplasm | 0.7 | - | 0.856 | 8.08E-04 | |
| CF317_002353-T1 | W9WUY8_9EURO | Uncharacterized protein *(Cladophialophora psammophila* CBS 110553) | 720 | 55.2 | 4E-91 | Nucleus | 1 | - | 0.838 | 4.66E-02 | |
| CF317_002517-T1 | A0A178FM89_TRIVO | Mitochondrial 37S ribosomal protein RSM19 *(Trichophyton violaceum)* | 390 | 76.8 | 3.6E-48 | Mitochondrion | 0.54 | - | 0.836 | 5.91E-03 | |
| CF317_000645-T1 | A0A0N1H608_9EURO | Cytochrome c oxidase assembly factor 6 *(Phialophora attinorum)* | 483 | 68.5 | 2.2E-61 | Nucleus | 1 | - | 0.832 | 3.53E-04 | |
| CF317_006945-T1 | A0A5M8PTI5_9LECA | **RRM domain-containing protein** *(Lasallia pustulata)* | 424 | 35.1 | 7.4E-44 | Cytoplasm | 0.7 | - | 0.828 | 3.50E-03 | |
| CF317_000542-T1 | A0A178CNR3_9EURO | Uncharacterized protein *(Fonsecaea multimorphosa)* | 236 | 33.8 | 5.1E-20 | Mitochondrion | 0.74 | - | 0.818 | 1.06E-02 | |
| CF317_006516-T1 | H6C4H9_EXODN | Uncharacterized protein *(Exophiala dermatitidis CBS 525.76)* | 1.043 | 64.9 | 4.1E-141 | Cytoplasm | 1 | EC:2.1.1.165 | 0.816 | 2.38E-03 | |
| CF317_008346-T1 | A0A1J9RUB5_9PEZI | Conidiation-specific protein *(Diplodia corticola)* | 126 | 41.4 | 4.4E-8 | Nucleus | 1 | - | 0.772 | 2.32E-03 | |
| CF317_000002-T1 | A0A0D2AIF6_9PEZI | **CMP/dCMP-type deaminase domain-containing protein** (Verruconis gallopava) | 930 | 81 | 1.4E-126 | Nucleus | 1 | - | 0.759 | 5.39E-03 | |
| CF317_009080-T1 | A0A1C1CK24_9EURO | Putative dna-binding protein *(Cladophialophora carrionii)* | 526 | 81.9 | 3.4E-68 | Nucleus | 1 | EC:2.1.1.13 | 0.758 | 7.81E-03 | |
| CF317_002219-T1 | A0A135UUW8_9PEZI | **U6 snRNA-associated Sm-like protein LSm4** *(Colletotrichum salicis)* | 508 | 78.6 | 4E-65 | Extracellular space | 1 | - | 0.740 | 3.92E-02 | |
| CF317_008039-T1 | A0A0D2DFT3_9EURO | **U3 small nucleolar RNA-associated protein 11** *(Cladophialophora immunda)* | 698 | 53.3 | 1.7E-89 | Nucleus | 1 | - | 0.738 | 3.60E-02 | |
| CF317_000273-T1 | A0A017SP44_9EURO | Putative cytochrome c oxidase assembly protein *(Aspergillus ruber CBS 135680)* | 406 | 58.6 | 1.2E-49 | Mitochondrion | 0.91 | - | 0.735 | 7.12E-03 | |
| CF317_009482-T1 | A0A438MT58_EXOME | Uncharacterized protein *(Exophiala mesophila)* | 886 | 35.8 | 3.9E-105 | Nucleus | 1 | - | 0.730 | 1.24E-03 | |
| CF317_006448-T1 | A0A0D1Z760_9EURO | **CUE domain-containing protein** *(Cladophialophora immunda)* | 2.316 | 54.9 | 0.0 | Nucleus | 1 | - | 0.721 | 1.95E-03 | |
| CF317_006097-T1 | W9VHN3_9EURO | Pre-mRNA-splicing factor isy1 *(Cladophialophora yegresii* CBS 114405) | 1.039 | 78.0 | 1.5E-141 | Mitochondrion | 1 | - | 0.721 | 1.63E-03 | |
| CF317_008787-T1 | A0A0N1H2C3_9EURO | Protein rds1 *(Phialophora attinorum)* | 2.062 | 83.9 | 0.0 | Extracellular space | 1 | - | 0.714 | 3.05E-03 | |
| CF317_008509-T1 | H6BW63_EXODN | 3-dehydroquinate synthase *(E*xophiala dermatitidis CBS 525.76) | 2.043 | 80.0 | 0.0 | Cytoplasm | 1 | - | 0.707 | 4.56E-02 | |
| CF317_002157-T1 | A0A177D1T6_9PLEO | NAD(P)-binding protein *(Paraphaeosphaeria sporulosa)* | 848 | 69.0 | 3.9E-113 | Mitochondrion | 1 | - | 0.707 | 2.15E-04 | |
| CF317_005715-T1 | A0A0D2EWD5_9EURO | **FAD_binding_3 domain-containing protein** *(Exophiala xenobiotica)* | 1.656 | 68.7 | 0.0 | Extracellular space | 1 | EC:1.14.13.1 | 0.706 | 7.16E-03 | |
| F317_002864-T1 | A0A0N0NHK1_9EURO | Tripeptidyl aminopeptidase *(Phialophora attinorum)* | 1.121 | 45.4 | 2E-145 | Anchored component of plasma membrane | 0.99 | - | 0.705 | 1.10E-02 | |
| CF317_001633-T1 | B8MNS2_TALSN | **Histone H2A** *(Talaromyces stipitatus ATCC 10500)* | 650 | 97.7 | 1.3E-86 | Mitochondrion | 1 | - | 0.703 | 1.37E-02 | |
| CF317_001682-T1 | A0A1L7XWV9_9HELO | **Multiprotein-bridging factor 1** *(Phialocephala subalpina)* | 538 | 68.8 | 8E-69 | Nucleus | 1 | - | 0.693 | 3.71E-02 | |
| CF317_006023-T1 | W2RLN7_9EURO | **BHLH domain-containing protein** *(Cyphellophora europaea CBS 101466)* | 694 | 58.9 | 5.3E-88 | Nucleus | 1 | - | 0.683 | 9.50E-04 | |
| CF317_006924-T1 | A0A3M7NUG5_9EURO | **Peptidase A1 domain-containing protein** *(Chaetothyriales sp. CBS 134916)* | 1.169 | 57.9 | 1.2E-156 | Extracellular space | 1 | EC:3.4.99; EC:3.4.23.1; EC:3.4.23.32 | 0.682 | 1.41E-02 | |
| CF317_004824-T1 | A0A0D2B1G6_9EURO | **Dihydroorotate dehydrogenase (fumarate)** *(Cladophialophora immunda)* | 1.281 | 73.3 | 1.1E-174 | Cytoplasm | 1 | EC:1.3.5.2; EC:1.3.98.1; EC:1.3.5.2 | 0.678 | 2.86E-04 | |
| CF317_000174-T1 | W9XP19_9EURO | DnaJ like subfamily B member 4 *(Capronia coronata CBS 617.96)* | 1.347 | 70.4 | 0.0 | Cytoplasm | 0.7 | - | 0.673 | 9.88E-03 | |
| CF317_001855-T1 | A0A0D2AJX5_9EURO | Uncharacterized protein *(Exophiala oligosperma)* | 203 | 28.5 | 2.9E-15 | Nucleus | 1 | - | 0.672 | 2.48E-02 | |
| CF317_006625-T1 | A0A1C1CS86_9EURO | NADH-ubiquinone oxidoreductase *(Cladophialophora carrionii)* | 762 | 59.0 | 2.8E-100 | Mitochondrion | 1 | - | 0.668 | 8.91E-03 | |
| CF317_001222-T1 | H6BRE4_EXODN | ATP-binding cassette, sub-family F, member 3 *(E*xophiala dermatitidis CBS 525.76) | 1.709 | 51.2 | 0.0 | Cytoplasm | 0.7 | EC:3.6.1.3; EC:3.6.1.15 | 0.664 | 2.88E-03 | |
| CF317_007693-T1 | W9YWP6_9EURO | **4F5 domain-containing protein** *(Capronia coronata CBS 617.96)* | 275 | 81.2 | 2.6E-34 | Cytoplasm | 0.7 | - | 0.660 | 2.56E-03 | |
| CF317_005967-T1 | A0A0D2CML3_9EURO | Uncharacterized protein *(Exophiala xenobiotica)* | 500 | 47.3 | 1.1E-60 | Mitochondrion | 0.5 | - | 0.648 | 3.43E-02 | |
| CF317_003533-T1 | A0A072PQ20_9EURO | **GATA-type domain-containing protein** *(Exophiala aquamarina CBS 119918)* | 1.191 | 51.8 | 4.2E-158 | Nucleus | 1 | - | 0.646 | 4.44E-03 | |
| CF317_001872-T1 | A0A072NTX4_9EURO | Uncharacterized protein *(Exophiala aquamarina CBS 119918)* | 182 | 48.4 | 1.1E-15 | Cytoplasm | 0.7 | - | 0.645 | 1.49E-02 | |
| CF317_003980-T1 | A0A0D1XEJ1_9EURO | Uncharacterized protein *(Exophiala sideris)* | 868 | 56.0 | 9.9E-151 | Nucleus | 1 | - | 0.643 | 4.06E-03 | |
| CF317_008279-T1 | A0A0D2BHB3_9EURO | **Cytochrome b-c1 complex subunit 7** *(Exophiala spinifera)* | 551 | 83.6 | 7.8E-72 | Mitochondrion | 1 | - | 0.642 | 8.88E-04 | |
| CF317_005464-T1 | A0A0D2D8C1_9EURO | Uncharacterized protein *(Phialophora americana)* | 1.020 | 73.2 | 6.1E-139 | Cytoplasm | 0.7 | - | 0.631 | 2.49E-02 | |
| CF317_002731-T1 | W9XYR3_9EURO | **2'-phosphotransferase** *(Capronia coronata CBS 617.96)* | 1.839 | 41.7 | 4.2E-105 | Cytoplasm | 0.7 | EC:2.4.2.30; EC:2.4.2.30; EC:2.7.1.160 | 0.630 | 6.86E-04 | |
| CF317_002984-T1 | W9YQD4_9EURO | F-type H+-transporting ATPase subunit epsilon *(Capronia epimyces CBS 606.96)* | 374 | 90.5 | 2.9E-50 | Mitochondrion | 1 | EC:7.1.2.2 | 0.614 | 1.76E-02 | |
| CF317_006564-T1 | A0A135LYK6_PENPA | Ribosomal protein S21 *(Penicillium patulum)* | 216 | 31.8 | 6.7E-18 | Mitochondrion | 1 | - | 0.612 | 1.53E-02 | |
| CF317_009023-T1 | A0A0D2BRZ4_9EURO | **Peptidyl-prolyl cis-trans isomerase** *(Exophiala xenobiotica)* | 871 | 89.0 | 7E-118 | Cytoplasm | 1 | EC:5.2.1.8 | 0.603 | 2.24E-02 | |
| CF317_008242-T1 | A0A1C1CIJ9_9EURO | **Translation machinery-associated protein 22** *(Cladophialophora carrionii)* | 526 | 85.2 | 4.9E-64 | Nucleus | 1 | - | 0.601 | 4.88E-02 | |
| CF317_006879-T1 | A0A1C1CKI5_9EURO | Putative 7alpha-cephem-methoxylase P8 chain related protein *(Cladophialophora carrionii)* | 1.302 | 81.9 | 0.0 | Cytoplasm | 0.7 | - | 0.598 | 3.10E-02 | |
| CF317_008394-T1 | A0A1C1C9R4_9EURO | Mitochondrial 54S ribosomal protein YmL35 *(Cladophialophora carrionii)* | 1.401 | 65.7 | 0.0 | Mitochondrion | 0.63 | - | 0.597 | 3.26E-03 | |
| CF317_007359-T1 | A0A0D2F5Y1_9EURO | Cytochrome c *(Phialophora americana)* | 571 | 92.2 | 3.2E-75 | Nucleus | 1 | - | 0.594 | 9.93E-03 | |
| CF317_001420-T1 | H6BM60_EXODN | **60S ribosomal protein L36** *(Exophiala dermatitidis* CBS 525.76) | 571 | 92.2 | 9.8E-751 | Mitochondrion | 1 | - | 0.589 | 1.21E-02 | |
| CF317_007719-T1 | A0A072PSY0_9EURO | **Cupin_2 domain-containing protein** *(Exophiala aquamarina CBS 119918)* | 987 | 56.2 | 2.9E-131 | Cytoplasm | 1 | - | 0.588 | 1.51E-02 | |
| CF317_008861-T1 | A0A0D2HZ34_XYLBA | **Proteasome subunit alpha type** *(Cladophialophora bantiana CBS 173.52)* | 1.377 | 87.8 | 0.0 | Cytoplasm | 1 | Ec:3.4.99; EC:3.4.25 | -0.606 | 1.79E-02 | |
| CF317_004212-T1 | V9DIH7_9EURO | **Isocitrate lyase** *(Cladophialophora carrionii CBS 160.54)* | 2.412 | 76.6 | 0.0 | Mitochondrial membrane | 0.81 | EC:4.1.3.1; EC:4.1.3.30 | -0.607 | 3.34E-02 | |
| CF317_006579-T1 | A0A178BWA1_9EURO | **Alpha/beta-glucosidase agdC** *(Fonsecaea multimorphosa)* | 3.262 | 66.3 | 0.0 | Anchored component of plasma membrane | 1 | EC:3.2.1.20; EC:3.2.1.21 | -0.609 | 6.11E-03 | |
| CF317_008191-T1 | A0A1C1D2P1_9EURO | Putative 4-hydroxy-2-oxoglutarate aldolase, mitochondrial *(Cladophialophora carrionii)* | 1.230 | 72.8 | 1.1E-168 | Cytoplasm | 1 | - | -0.609 | 3.80E-03 | |
| CF317_005988-T1 | H6C564_EXODN | Acetyl-CoA C-acetyltransferase *(Exophiala dermatitidis CBS 525.76)* | 1.740 | 81.1 | 0.0 | Extracellular space | 0.94 | Ec:2.3.1.16; EC:2.3.1.9 | -0.624 | 6.06E-04 | |
| CF317_008635-T1 | A0A0D2J828_9EURO | **DNA helicase** *(Rhinocladiella mackenziei CBS 650.93)* | 3.282 | 72.3 | 0.0 | Cytoplasm | 0.7 | EC:3.6.4.12; EC:3.6.1.15 | -0.627 | 4.33E-02 | |
| CF317_006995-T1 | A0A1C1CKK9_9EURO | Putative 4-hydroxy-2-oxoglutarate aldolase, mitochondrial *(Cladophialophora carrionii)* | 1.227 | 73 | 2.3E-168 | Cytoplasm | 1 | - | -0.632 | 8.79E-03 | |
| CF317_006421-T1 | A0A4Q4YMA5_9PEZI | **Aldedh domain-containing protein** (Monosporascus sp. CRB-9-2) | 1.849 | 73.9 | 0.0 | Cytoplasm | 1 | EC:1.2.1.5; EC:1.2.1.3 | -0.644 | 1.49E-02 | |
| CF317_009563-T1 | A0A178C6E0_9EURO | **RNase III domain-containing *protein****(Fonsecaea multimorphosa)* | 241 | 34 | 2.4E-21 | Cytoplasm | 0.7 | - | -0.646 | 9.50E-03 | |
| CF317_004788-T1 | A0A0D2CIA0_9EURO | **Proteasome endopeptidase complex** *(Exophiala xenobiotica)* | 1.339 | 91.1 | 0.0 | Cytoplasm | 1 | EC:3.4.99; EC:3.4.25 | -0.657 | 4.24E-02 | |
| CF317_007601-T1 | A0A5K1K211_9APHY | Protein SEY1 (EC) *(Ganoderma boninense)* | 99 | 34.9 | 2.4E-2 | Extracellular space | 1 |  | -0.677 | 2.05E-02 | |
| CF317_002131-T1 | A0A0D2B4N0_9EURO | **4-nitrophenylphosphatase** (*Exophiala oligosperma)* | 1.305 | 79.9 | 1.5E-180 | Cytoplasm | 1 | EC:3.1.3.18; EC:3.1.3.1 | -0.680 | 1.46E-02 | |
| CF317_001542-T1 | A0A072P870_9EURO | POT family proton-dependent oligopeptide transporter *(Exophiala aquamarina CBS 119918)* | 2.605 | 75.3 | 0.0 | Plasma membrane | 0.92 | - | -0.687 | 8.37E-03 | |
| CF317_002004-T1 | W9WT35_9EURO | Glucan 1,3-beta-glucosidase *(Cladophialophora psammophila CBS 110553)* | 1.325 | 76.6 | 0.0 | Extracellular space | 1 | EC:3.2.1.21; EC:3.2.1.39 | -0.757 | 4.50E-03 | |
| CF317_006969-T1 | A0A177FDD4_9EURO | **HRXXH domain-containing protein** *(Fonsecaea monophora)* | 976 | 63.0 | 3.8E-131 | Extracellular space | 1 | - | -0.764 | 7.56E-03 | |
| CF317_002650-T1 | A0A0W7VBN3_9HYPO | **Tautomerase_3 domain-containing protein** *(Trichoderma gamsii)* | 374 | 45.4 | 3.1E-43 | Cytoplasm | 0.7 | - | -0.769 | 2.00E-03 | |
| CF317_005049-T1 | A0A178Z717_9EURO | **Peptidase S53 domain-containing protein** *(Fonsecaea erecta)* | 1.745 | 58.4 | 0.0 | Extracellular space | 0.93 | - | -0.769 | 1.54E-03 | |
| CF317_000578-T1 | A0A4U0XJ64_9PEZI | **PKS_ER domain-containing protein** *(Friedmanniomyces simplex)* | 1.482 | 77.1 | 0.0 | Extracellular space | 1 | - | -0.774 | 2.64E-02 | |
| CF317_003179-T1 | W9YR98_9EURO | Uncharacterized protein *(Capronia coronata CBS 617.96)* | 575 | 52.5 | 6.2E-71 | Anchored component of plasma membrane | 1 | - | -0.782 | 2.39E-02 | |
| CF317_002560-T1 | A0A5J5F901_9PEZI | Arylsulfatase *(Sphaerosporella brunnea)* | 1.330 | 53.9 | 5.3E-177 | Cytoplasm | 0.6 | - | -0.799 | 8.70E-03 | |
| CF317_007869-T1 | A0A3M7NMJ9_9EURO | **Adaptin_N domain-containing protein** *(Chaetothyriales sp. CBS 134916)* | 288 | 27.9 | 1.3E-25 | Nucleus | 1 | - | -0.821 | 3.87E-02 | |
| CF317_008822-T1 | A0A2V1C428_9HELO | HET-domain-containing protein *(Cadophora sp. DSE1049)* | 108 | 27.6 | 4.8E-4 | Extracellular space | 1 | - | -0.868 | 5.91E-04 | |
| CF317_005885-T1 | V9D746_9EURO | **Lipase_3 domain-containing protein** *(Cladophialophora carrionii CBS 160.54)* | 952 | 52.1 | 2.1E-120 | Extracellular space | 1 | - | -0.872 | 6.87E-03 | |
| CF317_004960-T1 | W9VN02_9EURO | Murein transglycosylase *(Cladophialophora yegresii CBS 114405)* | 1.419 | 62.2 | 0.0 | Extracellular space | 0.9 | - | -0.876 | 3.12E-03 | |
| CF317_009191-T1 | A0A2P8A4S9_9PEZI | Choline transport protein *(Elsinoe australis)* | 1.780 | 61.0 | 0.0 | Plasma membrane | 0.88 | - | -0.922 | 9.34E-03 | |
| CF317_004099-T1 | F9XH01_ZYMTI | Deacylase-like protein *(Zymoseptoria tritici IPO323)* | 1.287 | 66.0 | 8.4E-176 | Extracellular space | 0.99 | EC:3.5.1.83; EC:3.5.1.81 | -0.999 | 1.48E-02 | |
| CF317_001423-T1 | A0A135TBW5_9PEZI | Uncharacterized protein *(Colletotrichum simmondsii)* | 245 | 35.1 | 2.7E-23 | Extracellular space | 0.93 | - | -1.021 | 3.64E-03 | |
| CF317_003460-T1 | W9YGP2_9EURO | Murein transglycosylase *(Capronia epimyces CBS 606.96)* | 978 | 61.9 | 8E-131 | Plasma membrane | 0.89 |  | -1.032 | 6.17E-04 | |
| CF317_009777-T1 | A0A448YG28_BRENA | **Elongation factor 1-alpha** *(Brettanomyces naardenensis)* | 163 | 28.6 | 1E-10 | Mitochondrion | 1 | - | -1.036 | 1.59E-03 | |
| CF317_007265-T1 | A0A2G5HZC2_CERBT | **Inorganic diphosphatase** *(Cercospora beticola)* | 1.114 | 68.1 | 8.9E-151 | Cytoplasm | 0.7 | EC:3.6.1.1; EC:3.6.1.15 | -1.170 | 2.80E-02 | |
| CF317_008131-T1 | A0A1L9R4I8_ASPWE | **Arabinan endo-1,5-alpha-L-arabinosidase***(Aspergillus wentii DTO 134E9)* | 84 | 41.7 | 4E-1 | Extracellular space | 1 | - | -1.207 | 4.71E-02 | |
| CF317_004400-T1 | A0A0D1ZSU6_EXOME | **Lipocln_cytosolic_FA-bd_dom domain-containing protein** *(Exophiala mesophila)* | 476 | 48.2 | 8.5E-58 | Extracellular space | 1 | - | -1.367 | 6.58E-04 | |
| CF317_001497-T1 | A0A067MPR6_9AGAM | Uncharacterized protein *(Botryobasidium botryosum FD-172 SS1)* | 81 | 31.2 | 1.1E0 | Extracellular space | 1 | - | -1.423 | 1.21E-02 | |
| CF317_004176-T1 | A0A409XRE1_PSICY | **AAA domain-containing protein** *(Psilocybe cyanescens)* | 83 | 31.6 | 6.6E-1 | Cytoplasm | 0.7 | - | -1.599 | 1.46E-03 | |
| CF317_003266-T1 | A0A1C1CNC5_9EURO | Malate dehydrogenase (*Cladophialophora carrionii)* | 551 | 45.7 | 1.2E-67 | Extracellular space | 0.99 | - | -1.948 | 5.07E-03 | |
| CF317_006383-T1 | A0A072PG02_9EURO | Murein transglycosylase *(Exophiala aquamarina CBS 119918)* | 1.127 | 68.8 | 2.6E-153 | Extracellular space | 1 | - | -2.033 | 1.50E-02 | |
| CF317_003479-T1 | A0A0L0SYM3_ALLM3 | **Centrosomal protein of 19 kDa** *(Allomyces macrogynus* ATCC 38327) | 97 | 32.1 | 3.7E-2 | Extracellular space | 0.99 | - | -2.211 | 3.83E-03 | |
| CF317_007621-T1 | A0A0D2AG04_9PEZI | **Cutinase** *(Verruconis gallopava)* | 704 | 54.0 | 3.8E-91 | Extracellular space | 1 | EC:3.1.1.1 | -2.795 | 7.69E-04 | |
| CF317_004369-T1 | A0A2H3IQM1_9EURO | **Alpha-galactosidase** (*Penicillium sp. 'occitanis')* | 768 | 55.0 | 2.6E-99 | Extracellular space | 0.99 | EC:3.2.1.22 | -2.939 | 3.45E-03 | |
| CF317_002368-T1 | B6H4B7_PENRW | Pc13g08300 protein *(Penicillium rubens****ATCC 28089)*** | 214 | 39.3 | 2.6E-19 | Extracellular space | 0.93 | - | -3.281 | 1.67E-04 | |

^a^ Protein accession number in the *K. chersonesos* database of *ab initio* translated proteins

^b^Up- and down-regulation of proteins (expressed as log2fold change) detected in the whole-cell proteome of *K. chersonesos* Mut under normal gravity (1G) compared to *K. chersonesos* Wt.

**Supplementary Table 11_**Homologues of differentially abundant proteins in *K. chersonesos* Mut secretome under normal gravity (1G) as compared to the wild type (BLASTP algorithm). Only the matching proteins with known identity (when available) and the highest max score are displayed. The predicted protein subcellular localizations are also shown.

| ***Protein* accession No. ^a^** | **UniProtKb accession**  **No.** | **Protein name** | **Max**  **Score** | **Identity**  **(%)** | **Expected**  **value** | **Predicted Localization** | **BUSCA Score** | **KEGG Enzymes code** | **Mut/**  **Wt**  **FC** **^b^** | **p-value** |  |  |
| --- | --- | --- | --- | --- | --- | --- | --- | --- | --- | --- | --- | --- |
| CF317_007965-T1 | A0A438NEL9_EXOME | Uncharacterized protein *(Exophiala mesophila)* | 403 | 47.5 | 1.5E-47 | Extracellular space | 0.91 | - | 4.112 | 2.40E-03 | |  |
| CF317_009675-T1 | W2RWQ2_9EURO | Uncharacterized protein *(Cyphellophora europaea* CBS 101466) | 346 | 37.8 | 1.4E-38 | Extracellular space | 1 | **-** | 2.718 | 1.12E-02 | |  |
| CF317_001497-T1 | A0A067MPR6_9AGAM | Uncharacterized protein *(Botryobasidium botryosum* FD-172 SS1) | 81 | 31.2 | 1.1E0 | Extracellular space | 1 | - | 2.131 | 2.66E-02 | |  |
| CF317_004027-T1 | A0A438NAZ0_EXOME | **Tyrosinase_Cu-bd domain-containing protein** *(Exophiala mesophila)* | 1.326 | 62.4 | 0.0 | Extracellular space | 0.84 | - | 2.126 | 4.41E-04 | |  |
| CF317_003654-T1 | H6BW33_EXODN | Uncharacterized protein *(Exophiala dermatitidis* CBS 525.76) | 587 | 64.8 | 1.8E-75 | Extracellular space | 0.99 | - | 2.069 | 2.28E-05 | |  |
| CF317_004702-T1 | A0A179FUL4_PURLI | Quinoprotein amine dehydrogenase beta chain-like protein *(Purpureocillium lilacinum)* | 439 | 39.6 | 1.8E-48 | Extracellular space | 0.77 | - | 1.964 | 6.01E-03 | |  |
| CF317_004653-T1 | A0A1L7WLC8_9HELO | **Carboxylic ester hydrolase** *(Phialocephala subalpina)* | 2.077 | 75.0 | 0.0 | Anchored component of plasma membrane | 0.85 | - | 1.951 | 8.43E-05 | |  |
| CF317_006512-T1 | A0A072PPW0_9EURO | **NTP_transf_9 domain-containing protein** *(Exophiala aquamarina CBS* 119918*)* | 993 | 64.0 | 4.6E-134 | Mitochondrion | 1 | - | 1.927 | 1.29E-03 | |  |
| CF317_001423-T1 | A0A135TBW5_9PEZI | Uncharacterized protein *(Colletotrichum simmondsii)* | 245 | 35.1 | 2.7E-23 | Extracellular space | 0.93 | - | 1.709 | 9.63E-05 | |  |
| CF317_000257-T1 | A0A3M7NIC9_9EURO | **Ydc2-catalyt domain-containing protein** *(Chaetothyriales sp. CBS 132003)* | 295 | 35.2 | 4.2E-28 | Extracellular space | 0.99 | - | 1.656 | 4.17E-04 | |  |
| CF317_007486-T1 | A0A1C1CHP7_9EURO | Phosphatidylethanolamine-binding protein (Cladophialophora carrionii) | 463 | 36.9 | 4.4E-54 | Extracellular space | 0.99 | - | 1.656 | 1.60E-02 | |  |
| CF317_009172-T1 | H6BZ20_EXODN | Uncharacterized protein *(Exophiala dermatitidis CBS 525.76)* | 448 | 49.7 | 6.4E-54 | Extracellular space | 1 | - | 1.646 | 5.33E-03 | |  |
| CF317_000335-T1 | W9Y0V5_9EURO | Glutamine synthetase *(Capronia epimyces CBS 606.96)* | 2.190 | 81.0 | 0.0 | Cytoplasm | 1 | EC:6.1.1.11; EC:6.3.1.2 | 1.593 | 2.38E-04 | |  |
| CF317_005659-T1 | A0A0D1WSW0_EXOME | **Peptidyl-prolyl cis-trans isomerase** *(Exophiala mesophila)* | 796 | 85.1 | 1.7E-107 | Cytoplasm | 0.7 | EC: 5.2.1.8 | 1.520 | 3.58E-02 | |  |
| CF317_004119-T1 | A0A438NIP5_EXOME | **Adenylosuccinate lyase** *(Exophiala mesophila)* | 1.713 | 70.4 | 0.0 | Cytoplasm | 0.82 | EC:4.3.2.2 | 1.509 | 1.58E-02 | |  |
| CF317_009031-T1 | A0A1Q8RG26_9PEZI | **Carboxylic ester hydrolase** *(Colletotrichum chlorophyti)* | 2.111 | 69.3 | 0.0 | Extracellular space | 0.74 | - | 1.498 | 4.50E-02 | |  |
| CF317_007359-T1 | A0A0D2F5Y1_9EURO | Cytochrome c *(Phialophora americana)* | 571 | 92.9 | 3.2E-75 | Nucleus | 1 | - | 1.493 | 1.11E-03 | |  |
| CF317_007195-T1 | H6BV74_EXODN | Aminopeptidase I *(Exophiala dermatitidis CBS 525.76)* | 1.970 | 73.7 | 0.0 | Mitochondrion | 1 | EC:3.4.11; EC:3.4.11.21; EC:3.4.11.16 | 1.492 | 3.54E-02 | |  |
| CF317_003059-T1 | A0A0D2I2U9_9EURO | **Cyanate hydratase** (Rhinocladiella mackenziei CBS 650.93) | 708 | 84.4 | 1.4E-94 | Cytoplasm | 1 | EC:4.2.1.104 | 1.487 | 3.95E-04 | |  |
| CF317_003885-T1 | A0A0N1NXS1_9EURO | Putative RNA-binding protein *(Phialophora attinorum)* | 1.648 | 79.6 | 0.0 | Cytoplasm | 0.7 | - | 1.426 | 4.83E-03 | | |
| CF317_001843-T1 | A0A1C1CGV8_9EURO | Putative formamidase *(Cladophialophora carrionii)* | 1.910 | 84.3 | 0.0 | Cytoplasm | 1 | - | 1.383 | 2.05E-03 | | |
| CF317_002674-T1 | H6BUN2_EXODN | Cell wall glucanase (Scw11) *(Exophiala dermatitidis* CBS 525.76*)* | 117 | 27.0 | 2.1E-5 | Plasma membrane | 0.76 |  | 1.373 | 5.39E-03 | | |
| CF317_008862-T1 | A0A178ZQK6_9EURO | **6,7-dimethyl-8-ribityllumazine synthase** *(Fonsecaea erecta)* | 944 | 80.9 | 2.2E-128 | Cytoplasm | 0.7 | EC:2.5.1.78 | 1.340 | 6.05E-05 | | |
| CF317_003302-T1 | A0A4U7B707_9PEZI | Transferrin receptor-like dimerization domain-containing protein *(Elsinoe australis)* | 2.536 | 77.5 | 0.0 | Cytoplasm | 0.7 | EC:3.4.17.11 | 1.309 | 1.07E-02 | | |
| CF317_006677-T1 | A0A0D2CVP1_9EURO | **HIT domain-containing protein** *(Cladophialophora immunda*) | 559 | 76.7 | 1.1E-72 | Cytoplasm | 0.73 | - | 1.308 | 8.90E-03 | | |
| CF317_003425-T1 | A0A507QRN3_MONPU | Uncharacterized protein *(Monascus purpureus)* | 368 | 60.2 | 4.5E-44 | Mitochondrion | 1 | - | 1.278 | 4.74E-03 | | |
| CF317_004564-T1 | A0A3M7NGZ3_9EURO | **Peptidase S53 domain-containing protein** *(Chaetothyriales sp. CBS 132003)* | 2.073 | 63.0 | 0.0 | Extracellular space | 0.96 | EC:3.4.21.73; EC:3.4.24.7; EC:3.4.21.71 | 1.276 | 6.38E-04 | | |
| CF317_002654-T1 | A0A0N1P280_9EURO | Scytalone dehydratase *(Phialophora attinorum)* | 807 | 78.5 | 1.1E-108 | Cytoplasm | 1 | EC:4.2.1.94 | 1.266 | 3.70E-02 | | |
| CF317_006099-T1 | A0A438MU03_EXOME | Putative agmatinase 1 *(Exophiala mesophila)* | 1.546 | 74.6 | 0.0 | Extracellular space | 1 | - | 1.257 | 3.33E-03 | | |
| CF317_003229-T1 | A0A194X6D2_9HELO | Putative versicolorin reductase *(Phialocephala scopiformis)* | 788 | 61.6 | 8.9E-103 | Cytoplasm | 1 | - | 1.255 | 2.61E-03 | | |
| CF317_005766-T1 | H6BXH6_EXODN | Vanillin dehydrogenase *(Exophiala dermatitidis CBS 525.76)* | 1.728 | 66.8 | 0.0 | Cytoplasm | 1 | - | 1.241 | 8.22E-04 | | |
| CF317_008840-T1 | W9XS38_9EURO | **Carboxypeptidase** (*Capronia coronata CBS 617.96)* | 2.033 | 67.8 | 0.0 | Extracellular space | 0.98 | EC:3.4.17.11; EC:3.4.21; EC:3.4.16; EC:3.4.16.2; EC:3.4.16.5; EC:3.4.16.6 | 1.214 | 3.75E-04 | | |
| CF317_002949-T1 | A0A177BY00_9PLEO | **Feruloyl esterase** *(Paraphaeosphaeria sporulosa)* | 730 | 54.5 | 1E-93 | Extracellular space | 0.99 | EC:3.1.1.73; EC:3.1.1.1 | 1.199 | 1.16E-03 | | |
| CF317_003648-T1 | A0A0D2CPX4_9EURO | Glycerophosphoryl diester phosphodiesterase **domain-containing protein** *(Exophiala xenobiotica)* | 1.663 | 72.6 | 0.0 | Extracellular space | 1 | - | 1.195 | 2.65E-04 | | |
| CF317_007147-T1 | A0A178C9G0_9EURO | **Nucleoside diphosphate kinase** *(Fonsecaea multimorphosa)* | 743 | 90.3 | 4.1E-100 | Cytoplasm | 1 | EC:2.7.4.6 | 1.195 | 8.70E-04 | | |
| CF317_000379-T1 | H6BY84_EXODN | Elongation factor EF-1 gamma subunit *(Exophiala dermatitidis CBS 525.76)* | 1.704 | 76.7 | 0.0 | Cytoplasm | 1 | EC:3.1.4.17; EC:2.5.1.18; EC:3.1.4.53 | 1.184 | 2.56E-03 | | |
| CF317_009579-T1 | W2RKS2_9EURO | **AB hydrolase-1 domain-containing protein** *(Cyphellophora europaea* CBS 101466) | 1.236 | 67.0 | 4.6E-169 | Extracellular space | 1 | - | 1.178 | 2.96E-03 | | |
| CF317_008154-T1 | H6C3S8_EXODN | Alcohol dehydrogenase *(Exophiala dermatitidis CBS 525.76)* | 1.336 | 70.9 | 0.0 | Cytoplasm | 1 | - | 1.164 | 8.10E-03 | | |
| CF317_008933-T1 | H6BVG8_EXODN | Elongation factor EF-1 beta subunit *(Exophiala dermatitidis CBS 525.76)* | 904 | 75.0 | 2.9E-122 | Cytoplasm | 0.7 | - | 1.151 | 2.09E-02 | | |
| CF317_006154-T1 | W9Y727_9EURO | **Pyruvate decarboxylase** *(Capronia coronata CBS 617.96)* | 1.980 | 68.0 | 0.0 | Cytoplasm | 1 | EC:2.2.1.6; EC:4.1.1.1 | 1.139 | 6.87E-03 | | |
| CF317_003202-T1 | G3JMK8_CORMM | Concanavalin A-like lectin/glucanase *(Cordyceps militaris*strain CM01) | 1.038 | 56.0 | 1.6E-138 | Cytoplasm | 0.7 | - | 1.105 | 4.79E-02 | | |
| CF317_002908-T1 | A0A1C1CAZ9_9EURO | Conidial pigment biosynthesis protein Ayg1 *(Cladophialophora carrionii)* | 1.760 | 76.6 | 0.0 | Cytoplasm | 1 | EC:3.4.21.73; EC:3.4.24.7; EC:3.4.21.71 | 1.101 | 5.16E-04 | | |
| CF317_004751-T1 | W2RJE3_9EURO | **Phosphoglycerate kinase** *(Cyphellophora europaea CBS* 101466) | 1,900 | 88.5 | 0.0 | Cytoplasm | 1 | EC:2.7.2.3 | 1.089 | 8.27E-04 | | |
| CF317_009408-T1 | A0A1C1CTV9_9EURO | Cyanamide hydratase *(Cladophialophora carrionii)* | 711 | 56.7 | 3.9E-92 | Cytoplasm | 0.94 | - | 1.085 | 3.78E-03 | | |
| CF317_008283-T1 | H6C4E3_EXODN | Laccase TilA *(Exophiala dermatitidis* CBS 525.76) | 1.948 | 49.4 | 0.0 | Anchored component of plasma membrane | 1 | EC:1.10.3; EC:1.10.3.2 | 1.074 | 9.13E-04 | | |
| CF317_008040-T1 | A0A1L9SZB3_9EURO | **Carboxylic ester hydrolase** *(Aspergillus sydowii CBS 593.65)* | 1.084 | 50.8 | 2E-142 | Extracellular space | 0.99 | - | 1.065 | 1.56E-03 | | |
| CF317_002308-T1 | A0A1J9RJA8_9PEZI | **Carboxylic ester hydrolase** *(Diplodia corticola)* | 1.819 | 63.0 | 0.0 | Extracellular space | 0.99 | EC:3.1.1.1 | 1.056 | 5.16E-03 | | |
| CF317_000574-T1 | W9XSL0_9EURO | **Cytosolic nonspecific dipeptidase (**Cladophialophora psammophila CBS 110553) | 2.215 | 86.2 | 0.0 | Cytoplasm | 1 | EC:3.4.13; EC:3.4.13.18; EC:3.4.19.9; EC:3.4.19 | 1.041 | 2.26E-03 | | |
| CF317_002864-T1 | A0A0N0NHK1_9EURO | Tripeptidyl aminopeptidase *(Phialophora attinorum)* | 1.121 | 45.4 | 2E-145 | Anchored component of plasma membrane | 0.99 | - | 1.040 | 4.11E-03 | | |
| CF317_005438-T1 | A0A178ZAV1_9EURO | **FAD-binding PCMH-type domain-containing protein** *(Fonsecaea erecta)* | 1.253 | 49.8 | 2.4E-165 | Plasma membrane | 0.84 | - | 1.039 | 2.67E-04 | | |
| CF317_001805-T1 | W2RWQ2_9EURO | Uncharacterized protein *(Cyphellophora europaea CBS* 101466*)* | 332 | 46.8 | 1.5E-36 | Extracellular space | 0.91 | - | 1.034 | 4.22E-02 | | |
| CF317_007740-T1 | A0A1Y2E5Z4_9PEZI | Ubiquitin 3 binding protein But2 C-terminal domain-domain-containing protein *(*Pseudomassariella vexata*)* | 906 | 71.4 | 6.5E-123 | Extracellular space | 0.98 | - | 0.998 | 4.79E-04 | | |
| CF317_009346-T1 | A0A4U7B682_9PEZI | Serine/threonine-protein kinase-like protein 3 (*Elsinoe australis)* | 222 | 69.4 | 1E-19 | Mitochondrion | 0.58 | - | 0.983 | 1.94E-02 | | |
| CF317_007588-T1 | H6C4E3_EXODN | Laccase TilA (*Exophiala dermatitidis CBS 525.76*) | 2.081 | 61.5 | 0.0 | Extracellular space | 0.94 | EC:1.10.3; EC:1.1.3.2 | 0.969 | 1.13E-03 | | |
| CF317_004005-T1 | A0A0D2EPQ4_9EURO | **Phytase-like domain-containing protein** *(Exophiala xenobiotica)* | 1.393 | 58.6 | 0.0 | Extracellular space | 0.99 | - | 0.941 | 5.62E-04 | | |
| CF317_001276-T1 | A0A072NWJ9_9EURO | **Mannan endo-1,6-alpha-mannosidase** *(Exophiala aquamarina CBS 119918)* | 1.537 | 69.1 | 0.0 | Extracellular space | 0.97 | EC:3.2.1.101; EC:3.2.1.24 | 0.941 | 6.57E-03 | | |
| CF317_007618-T1 | A0A6A6HNE7_9PEZI | **Carboxylic ester hydrolase** *(Viridothelium virens)* | 1.873 | 60.8 | 0.0 | Extracellular space | 1 | - | 0.929 | 1.17E-03 | | |
| CF317_009669-T1 | A0A2V1DXI3_9PLEO | Multicopper oxidase *(Periconia macrospinosa)* | 2.184 | 61.9 | 0.0 | Extracellular space | 0.9 | - | 0.919 | 2.26E-02 | | |
| CF317_003513-T1 | H6BNI5_EXODN | **2-phosphoglycerate dehydratase** (Exophiala dermatitidis CBS 525.76) | 2.067 | 89.5 | 0.0 | Cytoplasm | 1 | EC:4.2.1.11; EC:2.3.2.13 | 0.915 | 1.53E-03 | | |
| CF317_008924-T1 | W2S109_9EURO | **Homogentisate 1,2-dioxygenase***(Cyphellophora europaea CBS 101466)* | 2.182 | 81.7 | 0.0 | Extracellular space | 1 | EC:1.13.11; EC:1.13.11.5 | 0.902 | 4.76E-02 | | |
| CF317_005113-T1 | A0A0D2E1K8_9EURO | **Ribonuclease T(2)** *(Exophiala xenobiotica)* | 1.203 | 69.0 | 9.9E-165 | Extracellular space | 0.97 | EC:3.1.31; EC:3.1.27; EC:4.6.1.19 | 0.876 | 1.46E-03 | | |
| CF317_009012-T1 | A0A0D2DZ16_9EURO | **Uricase** *(Exophiala oligosperma*) | 1.280 | 82.0 | 3.3E-177 | Cytoplasm | 0.55 | EC:1.7.3.3 | 0.846 | 9.78E-03 | | |
| CF317_008227-T1 | A0A0D2BGD8_9EURO | **Alkaline phosphatase** *(Exophiala spinifera)* | 2.767 | 81.7 | 0.0 | Extracellular space | 1 | EC:3.1.3.1 | 0.839 | 1.44E-04 | | |
| CF317_006992-T1 | A0A178C5N3_9EURO | **Serine/threonine-protein kinase** (Fonsecaea multimorphosa) | 1.067 | 51.4 | 8.9E-131 | Cytoplasm | 1 | - | 0.819 | 2.90E-02 | | |
| CF317_004136-T1 | A0A0D1ZBT9_9EURO | **Chloride channel protein** *(Cladophialophora immunda)* | 2.706 | 66.7 | 0.0 | Endomembrane system | 0.73 | - | 0.810 | 3.74E-02 | | |
| CF317_006109-T1 | W9YXW4_9EURO | Thioredoxin 1 *(Capronia coronata CBS 617.96)* | 470 | 59.7 | 2E-58 | Cytoplasm | 1 | - | 0.809 | 8.21E-03 | | |
| CF317_005481-T1 | A0A0N0NLG1_9EURO | **6-phosphogluconate dehydrogenase, decarboxylating** *(Phialophora attinorum)* | 2.384 | 89.2 | 0.0 | Cytoplasm | 1 | EC:1.1.1.44 | 0.795 | 7.57E-04 | | |
| CF317_000763-T1 | H6BK35_EXODN | Carboxymethylenebutenolidase *(Exophiala dermatitidis CBS 525.76)* | 989 | 73.4 | 2.8E-134 | Cytoplasm | 1 | - | 0.792 | 3.12E-02 | | |
| CF317_009511-T1 | A0A6A6UNE0_9PEZI | Alpha/beta-hydrolase *(Microthyrium microscopicum)* | 1.198 | 56.3 | 1E-161 | Extracellular space | 0.92 | - | 0.787 | 5.84E-03 | | |
| CF317_001633-T1 | B8MNS2_TALSN | **Histone H2A** *(Talaromyces stipitatus*CBS 375.48) | 650 | 97.7 | 1.3E-86 | Mitochondrion | 1 | - | 0.784 | 9.78E-03 | | |
| CF317_005306-T1 | A0A2T2N8K6_CORCC | HAD-like protein *(Corynespora cassiicola* Philippines*)* | 995 | 76.3 | 2E-135 | Cytoplasm | 0.7 | - | 0.777 | 5.21E-04 | | |
| CF317_009216-T1 | A0A0D2D0N5_9EURO | **Catalase** *(Exophiala oligosperma)* | 3.095 | 76.2 | 0.0 | Cytoplasm | 1 | EC:1.11.1; EC:1.11.1.6 | 0.772 | 5.74E-03 | | |
| CF317_005124-T1 | A0A178C1A2_9EURO | Spermidine synthase *(Fonsecaea multimorphosa)* | 1.424 | 90.8 | 0.0 | Cytoplasm | 1 | EC:2.5.1.16; EC:3.6.1.15 | 0.770 | 2.48E-03 | | |
| CF317_002004-T1 | W9WT35_9EURO | Glucan 1,3-beta-glucosidase*(Cladophialophora psammophila* CBS 110553*)* | 1.325 | 76.6 | 0.0 | Extracellular space | 1 | EC:3.2.1.21; EC:3.2.1.39 | 0.763 | 6.03E-03 | | |
| CF317_007960-T1 | A0A0D1YWP7_9PEZI | 60S acidic ribosomal protein P1 *(Verruconis gallopava)* | 479 | 86.8 | 3.5E-61 | Extracellular space | 1 | - | 0.760 | 9.18E-03 | | |
| CF317_003714-T1 | A0A0D1WRJ8_9EURO | **Mannitol-1-phosphate 5-dehydrogenase** (*Exophiala sideris)* | 1.635 | 79.4 | 0.0 | Cytoplasm | 1 | EC:1.1.1.17 | 0.753 | 9.36E-05 | | |
| CF317_002925-T1 | A0A0D1WC07_9EURO | **Proteasome subunit alpha type** *(Exophiala sideris)* | 1.314 | 96.3 | 0.0 | Cytoplasm | 1 | EC:3.4.99; EC:3.4.25 | 0.750 | 8.77E-03 | | |
| CF317_007096-T1 | A0A0U1M0E3_TALIS | 6-phosphofructo-2-kinase *(*Penicillium islandicum*)* | 463 | 37.6 | 4.1E-49 | Nucleus | 1 | - | 0.745 | 5.59E-03 | | |
| CF317_001758-T1 | A0A178ZL52_9EURO | **Citrate synthase** *(Fonsecaea erecta)* | 2.218 | 89.9 | 0.0 | Mitochondrion | 0.97 | - | 0.737 | 4.76E-03 | | |
| CF317_006383-T1 | A0A072PG02_9EURO | Murein transglycosylase *(Exophiala aquamarina CBS 119918)* | 1.127 | 68.8 | 2.6E-153 | Extracellular space | 1 | - | 0.730 | 4.39E-03 | | |
| CF317_009544-T1 | A0A6A5RG93_9PLEO | **SCP domain-containing protein** *(Didymella exigua CBS 183.55)* | 554 | 47.8 | 1.1E-68 | Extracellular space | 0.98 | - | 0.730 | 1.44E-02 | | |
| CF317_008544-T1 | R1GAU1_BOTPV | Putative fad binding domain-containing protein *(Botryosphaeria parva)* | 932 | 48.7 | 9.3E-122 | Extracellular space | 1 | - | 0.728 | 2.43E-02 | | |
| CF317_001544-T1 | A0A077CZZ3_9EURO | Metaxin11 (*Exophiala pisciphila)* | 622 | 41.5 | 2.9E-71 | Extracellular space | 1 | EC:5.2.1.8 | 0.724 | 1.22E-02 | | |
| CF317_008191-T1 | A0A438N0Y4_EXOME | Putative 4-hydroxy-2-oxoglutarate aldolase, mitochondrial *(Exophiala mesophila)* | 1.230 | 72.8 | 1.1E-168 | Cytoplasm | 1 | - | 0.721 | 6.28E-03 | | |
| CF317_000127-T1 | W9YJD7_9EURO | **GTP-binding nuclear protein** *(Capronia epimyces CBS 606.96)* | 1.071 | 93.1 | 4.8E-148 | Cytoplasm | 1 | EC:3.6.1.15 | 0.714 | 1.62E-02 | | |
| CF317_004846-T1 | A0A0D2JAB3_9EURO | **Nitrate/nitrite transporter** *(Rhinocladiella mackenziei CBS* 650.93*)* | 1,820 | 66.0 | 0.0 | Plasma membrane | 0.79 | - | 0.713 | 1.84E-03 | | |
| CF317_008998-T1 | A0A6A6HAZ9_9PEZI | Putative fumarylacetoacetate hydrolase *(Viridothelium virens)* | 3.578 | 68.0 | 0.0 | Cytoplasm | 1 | EC:3.4.13; EC:3.4.13.18; EC:3.4.13.21 | 0.708 | 2.66E-02 | | |
| CF317_000453-T1 | W9X0J4_9EURO | 5-oxoprolinase (ATP-hydrolysing) (Cladophialophora psammophila CBS 110553) | 6.028 | 83.3 | 0.0 | Cytoplasm | 1 | EC:3.5.2.9 | 0.706 | 3.46E-02 | | |
| CF317_002428-T1 | A0A0D1Z1A9_9EURO | **GST C-terminal domain-containing protein** *(Exophiala sideris)* | 1.397 | 76.0 | 0.0 | Cytoplasm | 1 | EC:2.5.1.18 | 0.700 | 8.77E-03 | | |
| CF317_003258-T1 | A0A1S8BBE8_9PEZI | Chitin deacetylase *(Diplodia seriata)* | 1.025 | 51.3 | 4.9E-134 | Extracellular space | 1 | - | 0.693 | 9.73E-03 | | |
| CF317_003956-T1 | H6BVV8_EXODN | Uncharacterized protein *(Exophiala dermatitidis CBS 525.76)* | 431 | 50.5 | 1.1E-51 | Extracellular space | 1 | - | 0.681 | 1.50E-02 | | |
| CF317_006165-T1 | H6C9K5_EXODN | Esterase/lipase *(Exophiala dermatitidis CBS 525.76)* | 976 | 72.1 | 3.2E-132 | Cytoplasm | 1 | - | 0.678 | 9.78E-03 | | |
| CF317_007621-T1 | A0A0D2AG04_9PEZI | **Cutinase** *(Verruconis gallopava)* | 704 | 54.0 | 3.8E-91 | Extracellular space | 1 | EC:3.1.1.1 | 0.659 | 1.12E-02 | | |
| CF317_008448-T1 | W2RU25_9EURO | **Phosphotransferase** *(Cyphellophora europaea CBS 101466)* | 1.902 | 72.8 | 0.0 | Cytoplasm | 1 | EC:2.7.1.7; EC:2.7.1.4; EC:2.7.1.2; EC: 2.7.1.1 | 0.642 | 4.34E-04 | | |
| CF317_005049-T1 | A0A178Z717_9EURO | **Peptidase S53 domain-containing protein** *(Fonsecaea erecta)* | 1.745 | 58.4 | 0.0 | Extracellular space | 0.93 | EC:3.4.21.73; EC:3.4.24.7; EC:3.4.21.71 | 0.640 | 3.46E-03 | | |
| CF317_005318-T1 | A0A0D1WQ50_9EURO | **6-phosphogluconolactonase** *(Exophiala sideris)* | 1.058 | 75.8 | 1.6E-144 | Cytoplasm | 0.58 | EC:3.1.1.31; EC:3.1.1.1 | 0.629 | 4.50E-02 | | |
| CF317_005799-T1 | A0A1L7XT81_9HELO | **Carboxylic ester hydrolase** *(Phialocephala subalpina)* | 1.312 | 51.6 | 5.7E-175 | Plasma membrane | 0.83 | - | 0.626 | 9.50E-03 | | |
| CF317_000247-T1 | A0A0D2FR36_9EURO | **Formate dehydrogenase** *(Phialophora americana)* | 1.876 | 89.5 | 0.0 | Mitochondrion | 0.87 | EC:1.17.1.9 | 0.622 | 7.30E-04 | | |
| CF317_004400-T1 | A0A0D1ZSU6_EXOME | **Lipocln_cytosolic_FA-bd_dom domain-containing protein** *(Exophiala mesophila)* | 476 | 48.2 | 8.5E-58 | Extracellular space | 1 | - | 0.614 | 7.45E-03 | | |
| CF317_001284-T1 | A0A443HNA0_BYSSP | Ornithine cyclodeaminase *(Byssochlamys spectabilis)* | 888 | 49.1 | 2.8E-115 | Cytoplasm | 1 | - | 0.612 | 2.62E-03 | | |
| CF317_009244-T1 | A0A0D2IVM2_9EURO | **5-hydroxyisourate hydrolase** *(Rhinocladiella mackenziei CBS 650.93)* | 454 | 68.4 | 4.3E-56 | Cytoplasm | 1 | EC:3.5.2.17 | 0.607 | 1.54E-04 | | |
| CF317_007601-T1 | A0A5K1K211_9APHY | Protein SEY1 (EC) *(Ganoderma boninense)* | 99 | 34.9 | 2.4E-2 | Extracellular space | 1 | - | 0.604 | 4.38E-03 | | |
| CF317_006000-T1 | W9VLD8_9EURO | **ATP synthase subunit 5, mitochondrial** *(Cladophialophora yegresii CBS 114405)* | 879 | 78.0 | 4.8E-119 | Mitochondrion | 0.92 | EC:7.1.2.2 | 0.601 | 2.91E-02 | | |
| CF317_003892-T1 | A0A0D2BGD8_9EURO | **Alkaline phosphatase** *(Exophiala spinifera)* | 2.244 | 66.2 | 0.0 | Plasma membrane | 0.82 | EC:3.1.3.1 | 0.599 | 2.55E-03 | | |
| CF317_006347-T1 | A0A1C1C7L8_9EURO | Inorganic phosphate transport protein PHO88*(Cladophialophora carrionii)* | 811 | 81.4 | 2.6E-109 | Organelle membrane | 0.86 | - | -0.594 | 4.10E-02 | | |
| CF317_005450-T1 | C0NXQ7_AJECG | **40S ribosomal protein S27** (Ajellomyces capsulatus ATCC MYA-2454) | 468 | 100 | 1.3E-65 | Nucleus | 1 | - | -0.617 | 3.72E-03 | | |
| CF317_000809-T1 | A0A178CAJ6_9EURO | **Serine hydroxymethyltransferase** *(Fonsecaea nubica)* | 2.165 | 85.2 | 0.0 | Cytoplasm | 0.7 | EC:2.1.2.1 | -0.628 | 7.51E-03 | | |
| CF317_000354-T1 | A0A4V1M521_TREME | **C2 domain-containing protein** *(Tremella mesenterica)* | 138 | 26.0 | 5.7E-7 | Mitochondrion | 0.87 | - | -0.647 | 2.06E-02 | | |
| CF317_003462-T1 | A0A0N1H3F7_9EURO | Mitochondrial peroxiredoxin PRX1 *(Phialophora attinorum)* | 1.090 | 91.1 | 1.5E-150 | Cytoplasm | 0.79 | EC:1.11.1; EC:1.11.1.15 | -0.648 | 3.38E-02 | | |
| CF317_007500-T1 | A0A072PWW7_9EURO | **Ammonium transporter** *(Exophiala aquamarina CBS 119918)* | 2.250 | 77.0 | 0.0 | Plasma membrane | 0.82 | - | -0.651 | 2.27E-02 | | |
| CF317_004501-T1 | A0A0D2F446_9EURO | **40S ribosomal protein S24** *(Exophiala xenobiotica)* | 632 | 92.5 | 9.6E-84 | Mitochondrion | 1 | - | -0.654 | 1.53E-02 | | |
| CF317_000112-T1 | A0A0D2EKS0_9EURO | 60S ribosomal protein L34-B *(Exophiala xenobiotica)* | 539 | 84.6 | 1.1E-68 | Mitochondrion | 1 | - | -0.695 | 1.14E-02 | | |
| CF317_005810-T1 | A0A178ZG48_9EURO | **60S ribosomal protein L20** *(Fonsecaea erecta)* | 774 | 82.2 | 8.9E-104 | Nucleus | 1 | - | -0.696 | 1.85E-02 | | |
| CF317_002791-T1 | W9XAX5_9EURO | GTP-binding protein rhoA *(Capronia epimyces CBS 606.96)* | 968 | 87.1 | 2.3E-132 | Mitochondrion | 1 | EC:3.6.1.15 | -0.701 | 2.49E-02 | | |
| CF317_004351-T1 | A0A0D2DGP3_9EURO | **Endoplasmic reticulum chaperone BiP** *(Exophiala oligosperma)* | 3.063 | 87.5 | 0.0 | Endomembrane system | 0.79 | EC:3.6.1.15 | -0.704 | 5.04E-03 | | |
| CF317_001401-T1 | A0A0D2ARD5_9EURO | **t-SNARE coiled-coil homology domain-containing protein** *(Exophiala oligosperma)* | 937 | 57.0 | 1.8E-123 | Plasma membrane | 0.75 | - | -0.714 | 4.08E-02 | | |
| CF317_006726-T1 | A0A0D2E5Q2_9EURO | **CRAL-TRIO domain-containing protein** *(Exophiala oligosperma)* | 1.395 | 73.8 | 0.0 | Cytoplasm | 1 | - | -0.725 | 2.82E-02 | | |
| CF317_004635-T1 | U1GGM2_ENDPU | **Clr5 domain-containing protein** *(Endocarpon pusillum*HMAS-L-300199) | 1.503 | 52.7 | 0.0 | Nucleus | 1 | - | -0.742 | 4.34E-02 | | |
| CF317_005687-T1 | A0A178BUN2_9EURO | 60S ribosomal protein L16 *(Fonsecaea multimorphosa)* | 960 | 90.5 | 1.2E-131 | Mitochondrion | 1 | - | -0.743 | 6.96E-03 | | |
| CF317_004896-T1 | A0A438NBG8_EXOME | 60S ribosomal protein L9-B *(Exophiala mesophila)* | 828 | 83.7 | 1E-111 | Cytoplasm | 0.7 | - | -0.748 | 4.47E-05 | | |
| CF317_001542-T1 | A0A072P870_9EURO | POT family proton-dependent oligopeptide transporter *(Exophiala aquamarina CBS 119918)* | 2.605 | 75.3 | 0.0 | Plasma membrane | 0.92 | - | -0.750 | 5.56E-03 | | |
| CF317_001113-T1 | A0A0D1XHA1_9EURO | **3-hydroxy-3-methylglutaryl coenzyme A synthase** *(Exophiala sideris)* | 2.097 | 83.0 | 0.0 | Cytoplasm | 1 | EC: 2.3.3.10 | -0.761 | 5.70E-03 | | |
| CF317_005995-T1 | A0A6A6THM3_9PLEO | 60S ribosomal protein L42 *(Lophiostoma macrostomum CBS 122681)* | 551 | 91.1 | 6.2E-72 | Mitochondrion | 1 | - | -0.765 | 8.79E-03 | | |
| CF317_000101-T1 | A0A0D2H943_XYLBA | **40S ribosomal protein S21** *(Cladophialophora bantiana CBS 173.52)* | 430 | 93.2 | 1.8E-54 | Nucleus | 1 | - | -0.768 | 4.29E-03 | | |
| CF317_009008-T1 | W2S3U9_9EURO | 40S ribosomal protein S14 *(Cyphellophora europaea CBS 101466)* | 4744 | 96.0 | 2.3E-100 | Nucleus | 1 | - | -0.842 | 4.42E-03 | | |
| CF317_000963-T1 | A0A1C1CP89_9EURO | Putative Woronin body protein HexA *(Cladophialophora carrionii)* | 1.111 | 57.8 | 2.4E-145 | Mitochondrion | 1 | - | -0.855 | 2.51E-02 | | |
| CF317_000051-T1 | A0A1C1CMJ4_9EURO | Plasma membrane phosphatase required for sodium stress response *(Cladophialophora carrionii)* | 1.406 | 59.3 | 0.0 | Nucleus | 1 | - | -0.924 | 1.73E-02 | | |
| CF317_004669-T1 | H6C1E1_EXODN | 40S ribosomal protein S22 *(Exophiala dermatitidis CBS 52576*) | 643 | 96.2 | 1.2E-85 | Mitochondrion | 1 | - | -0.932 | 7.71E-03 | | |
| CF317_005118-T1 | A0A0D2DT15_9EURO | **40S ribosomal protein S6** *(Exophiala oligosperma)* | 1.173 | 94.6 | 7.3E-163 | Nucleus | 1 | EC:2.7.6.1 | -0.940 | 1.24E-03 | | |
| CF317_003991-T1 | A0A1C1CVD4_9EURO | CipC-like antibiotic response protein *(Cladophialophora carrionii)* | 437 | 64.8 | 1.7E-54 | Cytoplasm | 0.7 | - | -0.946 | 3.63E-03 | | |
| CF317_008676-T1 | H6C3U9_EXODN | **MARVEL domain-containing protein** *(Exophiala dermatitidis CBS 52576)* | 515 | 62.7 | 6.3E-65 | Plasma membrane | 0.79 | - | -0.960 | 4.39E-02 | | |
| CF317_008131-T1 | A0A1L9R4I8_ASPWE | **Arabinan endo-1,5-alpha-L-arabinosidase***(Aspergillus wentii DTO 134E9)* | 84 | 41.7 | 4E-1 | Extracellular space | 1 |  | -0.975 | 4.32E-02 | | |
| CF317_008030-T1 | A0A0D1YYX4_9EURO | **D-amino-acid oxidase domain-containing protein** *(Exophiala sideris)* | 1.042 | 60.6 | 6.6E-140 | Extracellular space | 1 | EC:1.4.3.3 | -0.976 | 1.10E-02 | | |
| CF317_004720-T1 | A0A1Y2TDM1_9PEZI | Glutathione S-transferase *(Hypoxylon sp. EC38)* | 1.092 | 78.5 | 2.8E-150 | Cytoplasm | 0.77 | - | -0.991 | 3.34E-03 | | |
| CF317_007220-T1 | A0A0D2G5Q6_XYLBA | **60S ribosomal protein L27** *(Cladophialophora bantiana CBS 173.52)* | 671 | 94.8 | 9.5E-90 | Mitochondrion | 1 | - | -1.016 | 2.05E-04 | | |
| CF317_007376-T1 | C0NH92_AJECG | 40S ribosomal protein S10-A *(Ajellomyces capsulatus ATCC MYA-2454)* | 720 | 82.7 | 1.7E-95 | Cytoplasm | 0.7 | - | -1.027 | 4.50E-02 | | |
| CF317_000184-T1 | A0A072PMQ4_9EURO | Large subunit ribosomal protein L24e *(Exophiala aquamarina CBS 119918)* | 704 | 89.4 | 5.7E-94 | Nucleus | 1 | - | -1.034 | 2.10E-02 | | |
| CF317_001237-T1 | H6BQF3_EXODN | 60S ribosomal protein L21-A *(Exophiala dermatitidis CBS 525.76)* | 685 | 82.9 | 3.0E-91 | Mitochondrion | 1 | - | -1.053 | 4.82E-02 | | |
| CF317_000935-T1 | A0A177EQW0_9EURO | **Ribosomal_L28e domain-containing protein** *(Fonsecaea monophora)* | 523 | 65.1 | 1.2E-66 | Mitochondrion | 1 | - | -1.056 | 2.37E-02 | | |
| CF317_008624-T1 | A0A072PIE2_9EURO | 60S ribosomal protein L5 *(Exophiala aquamarina CBS 119918)* | 1.383 | 85.4 | 0.0 | Mitochondrion | 1 | - | -1.067 | 3.46E-04 | | |
| CF317_003684-T1 | A0A0D2JML2_9EURO | 60S ribosomal protein L3 *(Fonsecaea multimorphosa CBS 102226)* | 1.925 | 92.1 | 0.0 | Mitochondrion | 1 | - | -1.088 | 3.29E-02 | | |
| CF317_009016-T1 | W9VF20_9EURO | **MFS domain-containing protein** *(Cladophialophora yegresii CBS 114405)* | 2.334 | 81.2 | 0.0 | Plasma membrane | 0.98 | - | -1.088 | 7.01E-04 | | |
| CF317_005751-T1 | U1HU65_ENDPU | **PH domain-containing protein** *(Endocarpon pusillum* HMAS-L-300199) | 1.406 | 54.2 | 0.0 | Nucleus | 1 | - | -1.094 | 2.16E-02 | | |
| CF317_003063-T1 | A0A0D2AFI1_9EURO | Uncharacterized protein *(Exophiala oligosperma)* | 2.922 | 58.1 | 0.0 | Nucleus | 1 | - | -1.106 | 8.76E-04 | | |
| CF317_002531-T1 | A0A0N1NY53_9EURO | Putative inorganic phosphate transporter C8E4.01c *(Phialophora attinorum)* | 2.647 | 75.5 | 0.0 | Plasma membrane | 0.73 | - | -1.107 | 1.01E-03 | | |
| CF317_007375-T1 | A0A0D2J8W0_9EURO | **40S ribosomal protein S8** *(Rhinocladiella mackenziei CBS 650.93)* | 910 | 86.6 | 5.4E-124 | Mitochondrion | 0.96 | - | -1.108 | 1.14E-03 | | |
| CF317_003734-T1 | A0A0D2EUP4_9EURO | **Ribosomal_L23eN domain-containing protein** *(Exophiala xenobiotica)* | 680 | 86.3 | 2.1E-90 | Nucleus | 1 | - | -1.123 | 4.09E-02 | | |
| CF317_001216-T1 | W9Z0C8_9EURO | **40S ribosomal protein S0** *(Capronia coronata CBS 617.96)* | 1.335 | 86.3 | 0.0 | Extracellular space | 1 | - | -1.128 | 7.29E-03 | | |
| CF317_005707-T1 | W2SCJ9_9EURO | **PH_6 domain-containing protein** *(Cyphellophora europaea CBS 101466)* | 1.219 | 43.0 | 3E-155 | Cytoplasm | 0.7 | - | -1.147 | 1.33E-04 | | |
| CF317_009253-T1 | A0A0D1ZQL5_9EURO | 40S ribosomal protein S19 *(Exophiala spinifera)* | 708 | 88.3 | 9.7E-95 | Cytoplasm | 0.7 | - | -1.154 | 3.33E-03 | | |
| CF317_005079-T1 | H6BSZ5_EXODN | 50S ribosomal protein L22e *(Exophiala dermatitidis CBS 525.76)* | 571 | 86.2 | 1.5E-74 | Mitochondrion | 1 | - | -1.164 | 6.23E-04 | | |
| CF317_008686-T1 | A0A0D1Z881_EXOME | **40S ribosomal protein S1** *(Exophiala mesophila)* | 1.235 | 93.8 | 1.2E-171 | Nucleus | 1 | - | -1.174 | 2.95E-03 | | |
| CF317_006800-T1 | A0A0D2D5R0_9EURO | **Aa_trans domain-containing protein** *(Exophiala oligosperma)* | 2.325 | 72.0 | 0.0 | Plasma membrane | 0.86 | - | -1.176 | 3.54E-03 | | |
| CF317_008702-T1 | W9Z3M1_9EURO | Large subunit ribosomal protein L14e *(Capronia epimyces CBS 606.96)* | 590 | 78.2 | 5.8E-77 | Cytoplasm | 0.7 | - | -1.182 | 6.84E-03 | | |
| CF317_008969-T1 | W2S2C1_9EURO | 40S ribosomal protein S5 *(Cyphellophora europaea CBS 101466)* | 1.021 | 93.5 | 1.7E-140 | Cytoplasm | 0.7 | - | -1.190 | 1.23E-03 | | |
| CF317_001861-T1 | A0A072PSJ3_9EURO | 40S ribosomal protein S20 *(Exophiala aquamarina CBS 119918)* | 568 | 97.4 | 1.2E-74 | Nucleus | 1 | - | -1.221 | 4.62E-03 | | |
| CF317_007792-T1 | A0A0D2CNM0_9EURO | 40S ribosomal protein S3 *(Cladophialophora immunda)* | 1.200 | 93.2 | 3.4E-169 | Cytoplasm | 0.7 | EC:3.2.2.23 | -1.229 | 6.38E-05 | | |
| CF317_003551-T1 | W9WE21_9EURO | 40S ribosomal protein S13 *(Cladophialophora yegresii CBS 114405)* | 745 | 96.7 | 1.6E-100 | Mitochondrion | 1 | - | -1.238 | 6.16E-03 | | |
| CF317_003042-T1 | A0A6G1FTI0_9PEZI | Ribosomal protein L14b/L23e *(Eremomyces bilateralis CBS 781.70)* | 680 | 94.3 | 5.9E-91 | Mitochondrion | 1 | - | -1.241 | 4.16E-03 | | |
| CF317_006910-T1 | A0A0D2IFS5_9EURO | **60S ribosomal protein L8** *(Rhinocladiella mackenziei CBS 650.93)* | 1.196 | 89.7 | 1.3E-165 | Nucleus | 1 | - | -1.259 | 2.64E-04 | | |
| CF317_006210-T1 | A0A0D2CGC8_9EURO | 40S ribosomal protein S2 *(Cladophialophora immunda)* | 1.241 | 93.2 | 2E-172 | Cytoplasm | 0.7 | - | -1.268 | 8.83E-03 | | |
| CF317_005768-T1 | A0A0D2CYL9_9EURO | 40S ribosomal protein S18 *(Cladophialophora immunda)* | 787 | 99.4 | 8.7E-107 | Mitochondrion | 1 | - | -1.274 | 5.75E-04 | | |
| CF317_004524-T1 | A0A072PAA2_9EURO | Ribosomal protein L37ae *(Exophiala aquamarina CBS 119918)* | 464 | 96.7 | 1.5E-59 | Cytoplasm | 0.7 | - | -1.284 | 1.43E-04 | | |
| CF317_003037-T1 | H6BN96_EXODN | Acyl-CoA-dependent ceramide synthase *(Exophiala dermatitidis CBS 52576)* | 1.613 | 68.7 | 0.0 | Mitochondrial membrane | 0.81 | - | -1.285 | 2.18E-02 | | |
| CF317_008148-T1 | H6BQY8_EXODN | **40S ribosomal protein S4** *(Exophiala dermatitidis CBS 525.76)* | 1.271 | 92.3 | 5.5E-177 | Mitochondrion | 1 | - | -1.289 | 1.76E-03 | | |
| CF317_007665-T1 | A0A0D2CV56_9EURO | Guanine nucleotide-binding protein subunit beta *(Cladophialophora immunda)* | 1.810 | 95.2 | 0.0 | Nucleus | 1 | - | -1.302 | 2.98E-03 | | |
| CF317_009010-T1 | A0A0D2DXK4_9EURO | 40S ribosomal protein S16 *(Exophiala oligosperma)* | 694 | 93.0 | 5.4E-93 | Mitochondrion | 1 | - | -1.316 | 5.56E-03 | | |
| CF317_004724-T1 | A0A0D2AL59_9EURO | 60S ribosomal protein L17 *(Exophiala oligosperma)* | 754 | 78.1 | 6.3E-97 | Nucleus | 1 | - | -1.317 | 5.58E-03 | | |
| CF317_002464-T1 | A0A0D2E8A3_9EURO | Ribosomal protein L24 *(Exophiala oligosperma)* | 569 | 83.3 | 3.6E-74 | Mitochondrion | 1 | - | -1.333 | 2.18E-02 | | |
| CF317_005549-T1 | A0A0D2D8I3_9EURO | **HABP4_PAI-RBP1 domain-containing protein** *(Phialophora americana)* | 942 | 53.4 | 1E-123 | Cytoplasm | 0.7 | - | -1.339 | 9.16E-04 | | |
| CF317_002664-T1 | A0A0D2I7E3_9EURO | **Plasma membrane ATPase** *(Rhinocladiella mackenziei CBS 650.93)* | 4.514 | 86.7 | 0.0 | Plasma membrane | 0.85 | Ec:7.1.3.1; EC:7.1.2.1 | -1.340 | 3.25E-02 | | |
| CF317_002107-T1 | A0A0G2EU75_9EURO | Putative 60s ribosomal protein l2 *(Phaeomoniella chlamydospora)* | 1.257 | 92.5 | 3.6E-175 | Mitochondrion | 1 | - | -1.340 | 6.60E-04 | | |
| CF317_006500-T1 | A0A2B7Y5E3_9EURO | 60S ribosomal protein L28 *(Polytolypa hystricis UAMH7299)* | 741 | 90.6 | 5.7E-100 | Mitochondrion | 1 | - | -1.353 | 2.22E-03 | | |
| CF317_005736-T1 | A0A150VFC6_9PEZI | **SHSP domain-containing protein** *(Acidomyces richmondensis BFW)* | 456 | 55.6 | 3.6E-55 | Cytoplasm | 0.92 | - | -1.380 | 7.75E-03 | | |
| CF317_009074-T1 | A0A3M7NVX6_9EURO | **Ribosomal protein L15** *(Chaetothyriales sp. CBS 134916)* | 1.045 | 95.6 | 1.6E-144 | Mitochondrion | 1 | - | -1.389 | 2.00E-03 | | |
| CF317_001420-T1 | H6BM60_EXODN | **60S ribosomal protein L36** *(Exophiala dermatitidis CBS 525.76)* | 410 | 83.98 | 9.8E-51 | Mitochondrion | 1 |  | -1.428 | 1.09E-03 | | |
| CF317_001485-T1 | A0A1C1CC26_9EURO | **40S ribosomal protein S26** *(Cladophialophora carrionii)* | 620 | 98.3 | 1.9E-82 | Cytoplasm | 0.7 |  | -1.457 | 6.49E-03 | | |
| CF317_000200-T1 | W2RYK7_9EURO | 40S ribosomal protein S23 *(Cyphellophora* europaea CBS 101466) | 756 | 100 | 2.4E-102 | Mitochondrion | 1 | EC:3.6.1.15 | -1.463 | 6.12E-03 | | |
| CF317_008155-T1 | A0A0D2B8I0_9EURO | **40S ribosomal protein S7** *(Exophiala spinifera)* | 908 | 88.2 | 1.1E-123 | Nucleus | 1 | - | -1.468 | 5.63E-03 | | |
| CF317_002841-T1 | H6BWQ6_EXODN) | 60S ribosomal protein L4-B *(Exophiala dermatitidis CBS 525.76)* | 1.556 | 82.3 | 0.0 | Nucleus | 1 | - | -1.476 | 9.74E-07 | | |
| CF317_001489-T1 | W9ZPS7_9EURO | 60S ribosomal protein L11 *(Capronia coronata CBS 617.96)* | 848 | 92.6 | 2E-115 | Nucleus | 1 | - | -1.492 | 2.05E-03 | | |
| CF317_001628-T1 | A0A3M7MQN8_9EURO | **SHSP domain-containing protein** *(Chaetothyriales sp. CBS 135597)* | 416 | 42.0 | 1.8E-48 | Cytoplasm | 1 | - | -1.510 | 2.54E-04 | | |
| CF317_009248-T1 | W9X9W8_9EURO | Isovaleryl-CoA dehydrogenase *(Cladophialophora psammophila CBS 110553)* | 1.691 | 83.1 | 0.0 | Cytoplasm | 0.7 | - | -1.513 | 2.63E-02 | | |
| CF317_003823-T1 | A0A1C1C932_9EURO | Putative carrier protein pet8 protein *(Cladophialophora carrionii)* | 222 | 43.9 | 5.4E-22 | Mitochondrion | 0.97 | - | -1.645 | 7.17E-03 | | |
| CF317_003183-T1 | H6C8I8_EXODN | **60S ribosomal protein L6** *(Exophiala dermatitidis CBS 525.76)* | 798 | 75.1 | 7.8E-107 | Nucleus | 1 | - | -1.720 | 2.26E-05 | | |
| CF317_005516-T1 | A0A0D1XDH6_9EURO | 60S ribosomal protein L7 *(Exophiala sideris)* | 1.200 | 89.3 | 1.6E-166 | Nucleus | 1 | - | -1.734 | 1.03E-03 | | |
| CF317_005782-T1 | A0A0G2E357_9EURO | Putative mfs phospholipid transporter *(Phaeomoniella chlamydospora)* | 1.831 | 73.5 | 0.0 | Plasma membrane | 0.78 | - | -1.749 | 7.05E-03 | | |
| CF317_005595-T1 | A0A178C7W2_9EURO | 60S ribosomal protein L33-A *(Fonsecaea multimorphosa)* | 492 | 83.5 | 2.8E-63 | Mitochondrion | 1 | - | -1.828 | 2.49E-04 | | |
| CF317_001879-T1 | W9WJM3_9EURO | 60S ribosomal protein L35 *(Cladophialophora yegresii CBS 114405)* | 546 | 85.7 | 6.3E-71 | Nucleus | 1 | - | -2.038 | 4.67E-05 | | |
| CF317_006070-T1 | W9YNN4_9EURO | 60S ribosomal protein L32 (Capronia coronata CBS 617.96**)** | 636 | 90.1 | 1.5E-84 | Mitochondrion | 1 | - | -2.276 | 4.70E-04 | | |
| CF317_001911-T1 | W9Z1V7_9EURO | MFS transporter, SP family, major inositol transporter (*Capronia coronata CBS 617.96)* | 2.232 | 74.9 | 0.0 | Plasma membrane | 0.95 | - | -2.328 | 2.92E-02 | | |

^a^ Protein accession number in the *K. chersonesos* database of *ab initio* translated proteins

^b^Up- and down-regulation of proteins (expressed as log2fold change) detected in secretome of *K. chersonesos* Mut under normal gravity (1G) compared to *K. chersonesos* Wt.
