# Supplementary material for: Effects of Simulated Microgravity on the Proteome and Secretome of the Polyextremotolerant Black Fungus Knufia chersonesos
Source: Front Genet. 2021 Mar 18;12:638708. doi: 10.3389/fgene.2021.638708 (PMC8012687; doi:10.3389/fgene.2021.638708)
Supplement: Supplementary file 2 [file Data_Sheet_2.docx]

>CF317_000001-T1 CF317_000001

MSEPDVIGPDHHPKNIGYYTKAIKRQFFTREGLIGDYNYGYLFKPNLPFTGAQNTSPPFFGLNDKMPLLLAALLGLQHTLAMLAGVITPALILSGAGGVNLTADLQQYLITRFHIRGTPYHIGTGLISVVGTSFAIIPVASGAFTQMYANGYCPVDANGNNEPCPKAYGALIGTAALCSLVEIAISFLPPKLLQRIFPPIVTGPTVLLIGVDLIGSSGFLNWAGGSGLCSDANPIEFYAKCPDITAPHALPWGSAEYIGLGFSVFVTILILERFVSNLEVDGPMFESRVQGGILADGLNGLLAAMMTITPMSTFAQNNGVIALTRCANRKAAFLFCAVAVAGMAIIARAPFNRRNRFILTASLSVGFGAILVPNWFSNVFTYSGGNQALLGFFDAIVLVMETGFAVTALLALLLNLTIAEELELDTRMLDGRAQADDRRHSMGTDGGSSGKGQAEMIKPVPEAKTE

>CF317_000002-T1 CF317_000002

MTESRAPEDLVSKLLSTTESSIIPLTRSQVSSGNKLFGAAILSKSTLQPITVATNNESASPLLHGEINCIQTFFASPKDTRPETKDCIFFATHEPCSLCLSGITWSGFNEFYYLFTYEDSRDLFAIPYDIDILQSVYQVPSPGDSPETLAAKPLYNRNNKFFTARSVADVVDSIQDGTEKAKWTKEIARIKAVYNELSDTYQKGKASGQETSSFFK

>CF317_000003-T1 CF317_000003

MSQTFKGHCHCGACEWEVELPSDSQGHILCHCDACKLQSGGEYTLNQIAPKDAFKLTKGDLKVYTYKGDSGNPVDCYFCGNCTSSPYHHQHVLGDKIVVRTGMLEGSKQWGKPMLEIFGKDKLSWQPQTADNIAPGPPGS

>CF317_000004-T1 CF317_000004

MSLTRVASVEGIERNNSDSHVTSTRRLSFNPMSSPSDAKAHAVQQLQSQEVSKSRRVLQVGIAIVYCLFAAGIVFGYAAIKPVLIDEGVYKNYCTKEELRQGTTPCYGQEIRLNIVFTVSAVSTNVAALPIGTILDRYGPRVCGIIGSVLLTIGALLFAFAGQAPVDAYIPGYLFLALGGPFVFISSFQLSNTFPDHSGLILALLTGAFDSSSALFLLFRLLYESTSGRLGTKRLFLIYLVVPAFILAVQLFVMPKESYKSISEVVEEAEEEAIAPTPPEYDENERQLYRERRDSTATEIQSLLDKSTNKKRADREEKKNDRSGVWGALHGYTAWEQIKTWWFVLITLFTVVQMTRINYFVATVRSQERWLLGSEDRAKTVNDFFDVALPLGGVLSIPFIGTILDRTSTPISLGVLVFFATVIGVLGCLPYMWAAIGNVVLFVIYRPFYYTAVSDYSAKVFGFQTFGKVYGLIICLAGILNFVQSPLDALTHTVFHGNPIPVNLVLLGCAVTVGTLLVAYTAIKSRNMKRHRLEMEAEGATDNAMPANGTSYGTN

>CF317_000005-T1 CF317_000005

MSSKNALFPRPNGSLKRKLNDPTTSFARSSSKKATKHNEHDSPRGMADSHVTSGSKEAYVEDEPEAHENDQEAGPDPPPEDDEEAEDNAGSDEEGRFFGGGVTKEERNVLDFIEQNEDGAPDEEINLAWLKRTALSFERKINKNAELRAKYEDDPMKFVASEADLDSEIKGLSLLSDHHELYRDFVRSGCSDSLIGLLAHDNTDIAIAACEVIVELIDEDSGVPAEEWSLLVKSMLKAELVDLLVSNLGRFDETVESDTAGVYHVLNVIENLLSDSANAEKLGSLSRLLDWLLLRIKRADETSSHKVGQNRQYAAEILVILAQNHDQNVSRLVKLGAVDTILQLLSTWRRRDPEKDSDEEEFAENLFDCLATMVKGAAGCEKFIEDEGVELCLIMLKEGKFSRKGSLRVLDYAAGGLNGSALCEHLVESGGLKQLFTTFMKSKKLEREATEHVIGILASLLRFLPAGSAARIRTLAKFVEKEYEKCNRLIELWQEYTSRLKTVDTGIDRERMDSTKEEADVNEEEWMSRRMDAGLFPLQTLNVILAWLVAEDDGARKAIAGIMEKQGHGGLEAIRKSLQEQLDGIEVEVNDEAVSTRDMLNALIRCLS

>CF317_000006-T1 CF317_000006

MAEYNVDEPYTNGDYGNEAEQVELAEDDCWEVVTSFFNHKNLCSLQIDSFNDFVDYQIPDLFREKGQVMIDQNEVAVDEDDPNPVVIKRHVIEFHNTTVARPTHVEGDGEVTELMPHEARLRSLVYSGNLTVNMSRKTHYAKARPWRLDQEGREDELGRMLYWEEGTGPDDYEEEQEIFAGKVPCMVKSKLCHLVDPARFPTENELYAWGECPYDQGGYFIINGSEKVLIAQERSAGNIVQVFQKAGPAPFTHLAEIRSVIDKGNRIMSQCTVKLYRRGEGPDGTKLDNPIRVQLPYVKQDIPLTIVFRALDVVSDMDILEKICFDMKDKEMIDLMMGSIQEGQVIQSAEVARDYIARRSNNTQLRSADRQKQALDILHKEFLPHIGQTPDATVKKAHFLGYMVARMLKCALGRTEPDDRDHLGKKRIDLPGPLMTTLFRQQLDKACKDLARYMEKRIQAGQPLSIRLGWKPNIISNGLKYSLATGNWGDQKKLDKAKAGVSQVLSRYTFMATLSHMRRTNAPIGREGKIAKPRQLHNTHWGYVCPAETPEGQACGLVKNISLLSLVSPARDSIPIERYVMEAGGNVLALEEWEPRLNPDVTKVFANGVWFCVLPGDPVFLVDAIRRLRRNGTLASDWSVVWDIREKELRISGDVGRLLRPLFVVETDRSSANFGNLVITRDDVDRIRADNWYNENTQEDKDADHEPFHWYSLVARGAIEYLDAEEEEQAMIIMTPENLADNRARAAGYEIEEDDDPLRRNQVPLAPGAHTFTHCEIHPSLILGVCASIIPFPDHNQSPRNTYQSAMGKQAMGIFLTDFNQRMETMANILYYPQKPLARTQSMNYLKFRELPAGQNAIVAIAVYSGYNQEDSVVMNQSSIDRGLFRSLFYRVYQDKENLVGQNVVEKFEKPSRADTLRLKHGTYDKLDDDGLVAPGVRVVGEDIIMGKTAPMAPDAEELGQRQKTHVKRDASVPLRSTEAGVVDSVMLTQGGDSQKVVKVRVRNTKVPQIGDKFASRHGQKGTIGITYRQEDMPFTREGVTPDIIINPHAIPSRMTIAHLIECLLSKVASIRGEEGDATPFTSVTVTQVSELLRSMGYHSRGFEVMYNGHTGRKMVAQVFFGPTYYQRLRHMVDDKIHSRARGPTQILTRQPVEGRARDGGLRFGEMERDCMIAHGASSFLKERLFDVSDPFRVHVCDICGLMTVIAKLKKNTFECKNCNNKNKISQIYLPYAAKLLFQELWSMNIAARLYTKKGS

>CF317_000007-T1 CF317_000007

MLGKQGALGATFILSRYAQAASMIAIIGMTSNFIAEMIQASMVPSEVLVGTLSVVCIAALYCAITVILFLDGLLPYLVNAIIDSLFLIALVVVSVVVGKPLSYLQCTIIGDLNSSASSAMSFASALSNSLTNEGGNINYANWIGTNKSTCLQMKSIWGLSVALCILFFLSAVSTICLWKRAKSGAAPEKGEA

>CF317_000008-T1 CF317_000008

MSSYGSYVPPDDDDDATDLSSAFSLASMSADYMIEHGRHFPNRDGSSPFPRGDDQGRANEIALHNLMFQLYDGRLYAAPIQRPRNVLDVRCGKQGLWAKNMADVYPEAQITGTDVFPVDTEGRDNLEFIYQSYNDQWILDEILQQYGKFDFIYARHLFATSQDYPDFYKQCLDHLEPGGYFEQCEMVPYCRCDDDTYREDSIVKGLCDLIPGIESAYQTHFDLALRMEQLIQEAGFVDIQRRIDKVPWSSWNPVGTKEHRTGELMERFYQTGIQGWLIQPLVNHLQFDQETVNNMVSAAIIELSQRDIHWWSPSITITARKPFNSD

>CF317_000009-T1 CF317_000009

MAFNSDVGYTAFANAKPSARFTLPPASFFKFPPSEIPQTLPPPPDHPTLAHPFTIRTDIYNATLSKFVPTTTALLYITAIVYLNRVNESRNHKPWAFSRTRLFKLMVLAHNILLAVYSAWTFVGMLNTLKVSVYPPWEELGLVGTVDSLCKVNGPRGLGSAARYNETSEAWSIANRFFHLGADGLTPDVTDVGRIWNEGLAFYGWLFYLSKFYEVIDTLIIIAKGKKSSFLQTFHHAGAMLCMWAGVRYMAAPIWLFVLANSFIHSIMYTFYFFSALGVKVPIWLKRTLTTMQISQFVIGAPFAFAHLFIEYQSPISVPYTYQFSSLASTVASSVASAATSADYGAYAKKLLLRAAGREGLAENVLNNQGHTFGIDALHPAQELVKRYETRYREELDWTHCLDTSGQTLAIYLNVLYLLPLTWLFGQFFVKSYMQRLERRRSSTVSQKAQLAIQSTRDASKGVGRRVSEAFEDSQGGINDLDEIAIIDEEEVKSALNDVKQAAKVAKAEIGKAASGSKKGMSRALDEMKGEADRVKEKARNEFQVDLDKVKEKYESVKSEVNLDTVQETYENVKSGIVSTAQEVMDTINTAQDESAVERQENEKSSISNIGTVTDRLQESAGSAVESVKNTVSDVLGLDLKHEDTNSQDEPANDKSQGHDNQSTADKSHRQEHPLTKAQKNDDTSIAENADDGAATDDKADDDVVVNKKKPEDGKQPETAEEDKSKQTQRPDQEVRRKDPEGQSNAEPRTGDARAAKSPADERTSRDDATQESPGPEIGRGGHSTSKPNKEESSVEDSSKQGSPAQNKSRQEKQMKQPESISPKDQGASWIHVDHNDDDTHGEKSSEQAKGARTHATKEGKDSGNHNVTSDNSKQVEGTQEGANRKLTEQPKKNDKTTNKGVGKSREEAARGASHREPIAKSKSSNQQPPKEGGSKIHHTPNDSAHLSYADVVAEHIEDDHEQKDDAEGQTPSDAVTPARGVPNLDTNKGDLSGAVANTKHRPEQTSKNTSATGTKQTQSSSTKTTSEDKGNSASRSSSPQWFENLTTGIDSPSKQKQDKHDRDIPKQDQDAIIADSEPVRDEAKDTATSPSKKKDQDSVKDTKGKAATKKTPNVPLGA

>CF317_000010-T1 CF317_000010

MGSLTATLPPMAHEFSDTYQAPKHYGDWPNAQGFDTTYEERTPVELQVEGHIPSYVAGTLFRTGLGPRSVDCGDKGVFRTNHWFDEFGQVHRFQIHAPTKEGDKARVTYNSRLTSDGLIAKVRQTGRLDGLTFGAKYEPCKTFFQKLQSFFRSAASQVPQALPQPNEANIGVTMSINFPGLSPTGQAISGPVDTSKISSVCTKTDASVVQMLDSETLEPIGLARQVVLHPELKGPLSGAHAKSDPETGDVFNFNLQLGSQGTYRVFRVSASTGETSILATVHHSSAYLHSMWLTENFVVLCIWNSHFKAGGLHILLKQNLADAMEYTTSQPAKWVVIDKRPAKEGGQGIVATYESPPFFGFHTVNAYEETSEDGVKHIIADIPAYESLEVLKAYYLDNAMSDSPSAGQVQGQWGNLLAPKYRRYRFPDIPDAPRAEPREAVLEFELDRQQTLELPQINGSVYTKKHRYVYGIFDSGKSTFADSLMKLDLEDKSVKRWSVHGHTAGEPIFIADPDSNAEDGGVLLTVVLDGIAGRSYLLVLDARDLSEIGRANVDGVVGFGFHGIHAKAHSAQKISISPEY

>CF317_000011-T1 CF317_000011

MAIEEPNPEGVDDEYDEEDDSDFDGQADEAGSISSATEDEPAVDKEVRQGNKATSSRPVVEELDSGDEVTIKERQKSKKKKQKHRKDAQLSSGDEQNAEWRAKTRSMRAHEQAERQRKTLISIKSSTIDVDKVWEEMNKPGPLPPVRVEGQSEERSAKEVHSAPRDDRPKVDTSALPSEEMVTIKRQYKFAGEVHLEEKTVPRSSAEAQLWLAQQENRKTTSQDSTGSKQRPLRKISRFDPNYSDLEAFRNHSIKAQPGTFNGPKLNVVEKSKMDWAVHIDQEGLRDELEVHAKAKDAYMNRMDFLTKVGQRQDDEARAARQKG

>CF317_000012-T1 CF317_000012

MDFLQSDEREYERYGSFPHSESCSSLSSSFLTVDTSSLSFDDGRRGSTSPDNFTASSGSVTSPITPLGPIPMHSFYAPEAWSKTQSHDLWQAQNPDPESFWMASEESAMANCVMNSFPQHTPATLFQPGYFCPTARPKSEVDPPLSRAVFNNTLSSSKINNPDEMVAWSNMGMITYPETVEPSATFQTMLPSSSPLSKYEPITPLRHKRGSSSLFASSPMSGGSSILASQWEMIDTKYMPDVDVYGHSEKSFLRRTLKSSQGSSPIKLEDESARKPFNSKSGVDCEAVIKQNTFACSRSGCHRRFKRQEHKKRHEKTVHAPQAGVIQQKYPCWVVTGDKVCGRIFTRRDNLKSHETKTHGRRSNNQRNAYVATLDPGSKYYDADWTGPLTAEGLPIGHPSFPEIC

>CF317_000013-T1 CF317_000013

MKTSTLLSILGGISSTIAHGKGDKGQKPITPSPPKHEPISSIPPLGLGLWNSKDKDATKATLSAFHNNYTHLDGAAAYGNEDYVGHALSDKKAPPRSSYWLTSKLWNTMHRPKNVPLAFNKTIAEYNTTYLDLYLMHWPVAFLSDKGSRSVIDQDTSITDTWAAMEMLASAYKASNGAYGARHIGISNFSPRQIDEIMKMCTICPYAHEFETHPYLQQQEFVNWHLEHDIKVIAYSPLANINPTYKDVMPDLKPILEDPTWEAVAKKKNCTVAQAILGWGVARGTIVIPKSVHEKRIKENIASLNVRFSEEELEKISKEDKRIRFNNPSKAWGVKLFEGLDDGTERFIMADGEL

>CF317_000014-T1 CF317_000014

MGGSEVTTSTTNGHSQTNHEPSSLVSESALDVKTESAATAKRKLESVENGDSEHQDKRRRGTAPIKEEFLLFNEPSEPAAPPSERENNDDDAAEAFHHKDRSASNNSKNQRKKQKGQNKGRHLKVGGDEVGLCSTRSHTPEFSPRECPYGDKCTFEHDLRKYLSQGRKSDLTTFNGICPVWQERGKCRVGWPCRFVQSHSKEIEHPDGRKELVLIENPELMAKVVDSSTSENAEVAVVNNIDIKDRQLLTRKKFPTPKSDKYLPYLDASQQNPEDQGKHRQKRQDRANSSSPEPDTKDNRANFVDPPLLPSEKRRLYFGPDTPVLAPLTTQGNLPFRRLCASLGASFTYSEMAMSLPLLSGQRSEWALLKAHESEHTPPTFISNDTVSSLKSKTAPNVVYDYNHATDLRFGAQVSANKPWLAIKTTEVLSALLPQGLRVIDMNCGCPIDLVFKEGAGSALMDAPNKLDKMLRGMNTVSGPIPVSCKIRMGTRNNAPTAQKLVERLVLGARRDRDRETPCGVAAITLHGRSRQQRYTKEADWEYISETASLIHTLNETAALETDTIREADPRDKPASSGKSHGTFFLGNGDVYSHTDYYNHLDRAGVDSVMLARGALIKPWIFEEISQNQYLDKSATERLSYIETFVRHGLAAWGSDTHGISTTRRFLLEYLSFTHRYVPIGLLEYLPPKINERPPRYKGRNELETLLASDSYKDWIRISEMYLGKISDAEGKSQEEYGWVPKHKSNAWDGEAQG

>CF317_000015-T1 CF317_000015

MWRDLHALLTHYEETHAQQEPVQDQGTGQSTMPDQRAALASNTRAQIQHEAQRRAQQIEPQGGAVQPALNRSGFSNTLQTIPDMDAVEDMEMDDIDGNNETTPPSNMFAPISQHNSPTTQFSPTAQPQVPQLNTNLMQSHQAYRNSTPNTPIASRNPAAMQGANISSTLNLNPMQQFQDLHNPYRDTPDSSAPGTPGELDESFMAELGDMNMQPDGMFNGMNGFQNNFGFGASNDMIDLCIDEPAKRLFQTNQAPNTQQAIHQRLGSGQYGPNSEIARRIREQQIKAGLPDTMNNLLPNEEPKPYRCPVIGCDKSYRNANGLKYHKTHGHQNQRLQENGDGTFSILDPDNNQPYPGTIGMEKEKPYKCDVCHKRYKNLNGLKYHKTHSPPCNPELQQQRLQQQQQQLQQTQRTPVASPNQQQLPASSSMSNILPNNNLGMNMNMNNMNFGGVMGNDRNIS

>CF317_000016-T1 CF317_000016

MLIKVTTVISTGLLTLEPRAFVDVPQAMRATTAFDGSDGLPLDKIDARAATGLYGHREYNMLLPNGTTTQLAFQLVEPVTSVPRDTYAYNASVDVFVPNGWDCESGKLTYENATDHVPTGDSTGYSEISPFMMYYNTSISLSDCQIHNGHLDAPQWYYDTPSNATIEPRYGYWGIFQSVNCSNLEPSHPKFHRYVISVAYSKGVGQDDNILLNSSNVVCIPSYTIQQGFVSTFTNGTLNGEIEFRDNMRQLEGITADDITLAAYKSVQQTNIPSNFNENTVTSDMFLNTMMALTTGFQIEMLMDISWLATKSKEAYQQYAAQIAGLYLLKQQTGSAQLDGHLARNEQRLVAKAGGFLSLSAPAARALVDCTLIPQSQYWNLTYEESGHSVSYNLSTPEGCGNMGWMDSNMIYYQSDVTAPTSDTGYFGDLVSGFGTVTRSTDKVKLQCPDYYITYGKIEDRKLADFSMYACNTGLETVQTEVTISLDLATVVGVSQVMENSSKPFQDHWDPEFAIYFWDVDHSHPDESFDAFVDVMVYGKDGIPREQLLDPDTVVEQFTHTYRQWVAQWMDAYMRNPVGELARNESNIAGNLPGSLSGVYNDPNRQRLFVTEVSTRILQGIIAILLVCGVVVFLLVDMSKVLPKPVGTIAAVASLLAGSRLVDEKSGLIPPGSEWLSDTEMKKRGMWDGERFRMGWWNDESAEIDLDELDTSYDGGATAKEADPGFFGIDARPDDTGDFKNDTR

>CF317_000017-T1 CF317_000017

MSSENEIVSNGANSNGVEQGDKSHDQGHSHTEEAPSTTGIRSSSGWDGKLRMQPRSAVLANPEALTDPDYSDPDAPPVEQIPADDDLLDDEDPDTEDIDLVHCRIVSLSSLRLDRFSKLKRLCLRQNAIQDIQLPDELGHTLEELDLYDNLIKHLEPGQLDKLTSLRTLDLSFNKLKHIKNLDSLTSLTELYFVQNRITKIENLDTLTNLTMIELAANRIREIEGLDHLAQLKELWLGKNKITEITNIGHLQSLRLLDLKSNRLTSISGLEELPNLEELYVSHNAITAISPTSLKENSKLRVLDISNNRIAKLENIGHLKDLEEFWASANGFSDFRDVERELADKENLETVYFEANPLQLNGPAVYRNKVRLALPQIKQIDATFVRV

>CF317_000018-T1 CF317_000018

MTTALHGQDGGHNQAVMAQALALVLVIIFQIWRSSSSLSNDDTAASHVANIIRRLNTILDEESRKAAPHSCLHYTASNQTYIIVTKLAVSSTSNEIIDQTVRFFHSLINGEVEDVLDHSLFARSLIELVKRTTAPATKLIDDKEETALIELLFEICTKIRLDPDILPAWFYPERVPGKTLSPLEARRSQFPLFYILIDYVHYDGPIGDFARTALLYLTETASKSRPLEKWMMESDLSHQMASGLSALYGRLSRHAPGISKKALPILSYSDTSDPKDGQDEFYDDAGMDMRAFLSYLAFWQDTLSHCKSVEVRDTLLDSYQVLFVEQLLYPSLLESSDVEGGSTAAVILHLCRLLDALDHPQLVSGMLSYLFATKSQPALAKDQRARSRPSMSRRHSMEQLANLVSKTDNPSPDLFNLLDLMVFSLRSSNSQTVTSALKLITVLLRRHHPHVKHHLFHVDAAKPASRQNLNGLNDTMMTLFDQAGAISPIHTMDSSYQAALKDAQTMMEHHSCLLEDDTSRQAEVSQMAIAKDCKVLEQMLNLLESWFANDTLVNLELTGTLGALASCEHVLLRSWLAPEQADQPSVISILEELIQQVKTWRKQFVEWDAFYSIQRVDLMSEEELLSLDHSTSSVFNEKMSPAPKMGSNSPPSSPQTLVGQVAGMKIESIDGRIASDGSTTVERGSTRQTSGEQAASDISTRSVSSGNLSATLLETRIPIEPRSRKQAASTNTEGGGSVDVEGAEGGPDDSTISDETGGLDAEVKVTASLRHVLTQAIILQEFILEVGAALQIRATLFDEVDLS

>CF317_000019-T1 CF317_000019

MAQEVEERYISPVARVRSQLSAKSDNEEGEEEAPTPKRQPTKTSTGIASEGGHELYKIKPLTKHEGKSVVTFFNGLFQTIIAISTLGTSVTFSFILSTDSALSRPEAYYDQSQVATFLAISWLLFLLALAFGSLCSTLLTFFKAHWIRDWDGEHGRISQREVQLYAVLASGLLGGLVIGAFIFLCLVVVAYSPVVGWIALAFTGWFGLVILLSILWQVPWPWRQNAPDARCGKPP

>CF317_000020-T1 CF317_000020

MAGPPLSQASGYGIVLGLGFAFALGMIGTTWALKRYHNEVQTSEAFSTAGRTVKSGLVASAVVSSWTWAATLLQSSSVAYSYGVSGPFWYASGATVQIILFATLAIELKRRAPNAHTFLETVRARYGKFTHIVFLVFGLMTNILVTAMLLTGGSAVVESLTGVPTAAACFLLPVGVVLYTMFGGIKATFLTDYVHTVIILVIIFLFAFSAYATNDIIGSPGRVYDLLVQAAKAHPVEGNTDGSYLTMHSKEGAIFFVINIVGNFGTVFLDNGYYNKAIAASPIHALPGYVMGGLSWFAIPWLCATTMGLAALALESNPVFPTYPNRIPASEVSAGLVLPHAATALLGKGGASATLLLIFMAVTSASSAELIAVSTIFTYDIYQTYINPKASGKFLIRMSHTTCVVYSIFMAAFSTGLYYGGVSMGYLYLLMGTIISSAVIPAALTLTWKDQNWIAAAASPVLGLTCSLIAWLVTAETHCGVLNVECTGSNYPMLAGNVTALLSPIIFVPILTFAFGRQNYDWVSMKMISRVDDSEIIRRTSVASGTADPELVPGSEAARSQATEDAEVAHLNKVAKIARGLTVFMTVALLVLWPMPLFGSKYVFSKKFFTGWVVVGILWLFCSTFMVGIFPVWQGRKTAAHTIKSMFLDLTGKRKPTLQGRVQEVSDEDDSGRVTPTEKADAPSSPSPPDSPSTATSGKSSSLLRASSPPGGPRSAIRRRAAADHKESVKNARPSSTRAAGAGGSSGTMLRLYTDESPGLKVDPMVILFLSLGFIFSVVALHLIAKLTKRFS

>CF317_000021-T1 CF317_000021

MFKRTAGYTTDDIPGQLPPDSIPEDVDVGKVSELATDCLKDLNPTILTHDVLWRDWMSLTGQHRTFYGTAEVSETWTKAVLQKRLTDIEVKSKRVLRPCPGSSWVSVTVTYNTQQENGLKGFHTANLGLVPDSGSWKIWMLVTILESFEGYNHPDEPQMAPAPERSILSSGCDYSVVVVGGGQGGLSLAGRLRALNIPTLVIENGPAVGNAWTSKYDSVKQHTLREYNNLPFGRTWNKDDPDLLPGKVVADGFAKYTEKYGITVWVNGEIVSSTRDDSTGTWTFEVVIKDPSSGSAKTRAIRTKHLAVATGAGVGAPSMPSIDRADRFRGTYIHQSTFRNAKNFSGKRAVIIGTGTTGHDIAQDCFNNDMRVTIVQRGPTAIYPIEWVIETQKHLWNLDMDTGLADRMGGTAPVKVGCEIIKRNMQAAAKQPHGMLDQIWQRYGGYYIDVGASKHITNGDINVKSAVPIAELTADGILFEDGEEMKADLIVAATGYDQDYRKQVAQIVGNDIASQLPEFWGLTKDGDVRGFMQENRPGLWMIGGTAPQARWSSRFVAMAIMLDLLGIENVVT

>CF317_000022-T1 CF317_000022

MDSDGVEQVPSNVTRIVRAIPAVGVDQTGPGPKPISQVTFYQNGIGTGSSGWYNKHIGKYISGATGEGLAANIREAYSFICCNYHRGDEITLLGFSRGAFTARSISTLIRQVGLLTPKGLSHLVEITDDWEHQNDKDYTTPYPDEPFSDRPGFSTEEYHKTLHDLHMTRQNINVKCVAVWDTVGALGLPRIALFPQPAAKEFAFVDTRVEDNIEYAFQALGLDEKRRSYAPSVWFKPLEQKFPKVLKQTWFPGVHSDIGGSYSNDDIANITLAWMVGQLEEHKLVTFNRDYIIRLVHSTIKHHAQETKKLEEDHSPSLERAKTFGPLRKWGLGKIHDSYTLFFRLGGERTRTPMQHPEVSRETMKPTGQLLQHTHEKMHACVRVRMALDGRGYDDKGRYASEALKDWKYTYGRRAEPDTPFQVQQPGEVGKLKDVEWVKVVTKDGEKEEVARMAEDIMTDFERLVLRHWTVAEDDWEREADEREYEVDTARSTARESSKVWGTPSSAREDAQGVRGETANGTIYGRVNGFQAVREVEENGAGRTRHLHVETDVD

>CF317_000023-T1 CF317_000023

MLLQDSVAPHDTASPIASQFLLTISLEVLEALEAAVMATRIYSNNKYLLERLLSACDSILELCLCRRQQPPQHPQSPPPQPSSSLLLVFPPWSQGSEEPWDPLVRQWSLDSTPEPFPEPIAEPDEPQHAVSGKEILVLLLALGLWLCVLILRDMMEAGPLPSTKIHPQSHPQVETQYTRPTIRSLDFPLAHCTSHIQGAQRNAGARVSMQLTNIFRTAQGLPAEVIFDTGASMSIADKSWLLKHCPGCQPKTLHRPLQAKGVSGASSWLTEFVDVDLVFNAQTRDGPVDLRLAATVVLFENSNANMLLGMDLAYLHKIDVDQRRQKLIFREAKDAEVDFRLYVW

>CF317_000024-T1 CF317_000024

MLEDSSLSAMAAIPTPLPPVSAGEVFTSDQWTTLMSICEVFMPSISISSLRETTYAEARDKVSAVLPQDADANLADIYLAETLVASQDFKEALRQRFAGYIPQAQVQGFAFLLSALNSKIGCLAMTSSTTLLHTQDLATRTKIVMKWADSYIGPCRALYQATEFLTKATWLAQSSVLHQVLDYPKVPKNVERHPGYDFQFLDFSNESADTPVEVAADVVIIGSGCGAGVVAEHLSSSLSSLDPKPRILVLEKGYHFPSTHFPMDQSAAGVNLQEGGGGILADDGSIAVLAGSTWGGGGTVNWSASLQPQHYVRQEWATKNKLPFMLSQDFQACLDEVCDKMGVCKPTDPAGLAQIEHSFGNSALLEGARRLGLTAKVVPQNSAGKHHYCGRCSMGCASSTKQGPATFWLPAAAERGVEFIEGCFVEKIIWDENRTSNRMATGVKAVWTSRSRDATRQLRISAKRIIISSGTLHTPLLLHRSGMTPEVNKNIGSNLHLHPVVPVHATYSQRTDPWDGAILTTVMSSLENLDGQGHGAKIEIMLGTPDMAGTMLPFRPHLSLVKGSDVLESALDYKVRVARHGHAFSFIAMGRDHAEDSNLKKSYVYGDRDDMRIVKVSYTPSPKDREHVLEGAMVAARMHYVMGAETIEICSPSISVFERPSVRLSNRTSSQVQSPTDDEAFEHWLLNIRTKGISLLDPRTTRMGSAHQMSTCRMSASPADGAVDPDGKVWGTENVYVADASILPSASGVNPMVSNMGLSLHVARGIAKTIAQARG

>CF317_000025-T1 CF317_000025

MTRSDSRRHISGHESTLERTISHPPPAIATAAPLAPISSGPEQGHVDHGHRQTLGDDEKHPPWNHKDRNFNEKAIHPSRRLSSSSSTLTRPQNDNDIDNTISRVSTDPAGNVYPEGGRKAYSVVFGSFCGCLISLGLMNTLGTYQAYVTTNQLSDMSHSRIGWIFGTYAFLSFFLGLQFGPLFDAYGPRWLVFSGSICILLTYILLAECTQYWHFMLVFGVLGGTGTSLVFTPCIAAVSHWFYLRRATFTGLACVGGALGGIVYPLTLQALFEIEAVGWPWAQRIVALINLPLAICANLFVRSRLPPRRPVERSQIMPDPRIFRDPTFSLVTAAVFFVEWGLFVPVGYLTNFALDIGLEEKFSYQLIAIFNTGSCLGRWLPGLIADRIGRFNAMIFTTVICWVGAFAFWLPSELLGVESESGRKALLIVFSVVFGFGSGSGISLIPVCVGQLCRTEEYGRYYATCYTIVSFATLTGIPLAGGIVSATAGNRYWGLILFTGVSYVISTAIFMVAKGGKVGYGKALWANNF

>CF317_000026-T1 CF317_000026

MKSTRNTSTRERAPESHAAAEHAGHVVEKVVKLKIGAESAKPNLHFRLPGPAQAHTTREVTVQAQLPRDSEQSDIGHHHPTSWQYFELDHKLASHTSPIHGSDGGLTSPATATSQSRKRDASVAGFDASPGSIESPDQHDADDHHSRKRPVKRACNECRQQKLRCDVVSEPAYQICSRCKRLNLECKIEENFRRVGKRSRNAEMEREIVQLRKQVAAQNVNTNVNINTTASANSQLPQPPPPSMNQAAVGTSEAAAGLLDLRDGGRGGSYKRLGDVVLQVDRVQDLFQRFFKFSHPFLPFLNPERPPEEYYAKSQLLFWTIILVGSRHYSAEPHLFASLARQVQTLTWSTLAEVPQNYHHVKALVLLCAWPLPCSSTSTDPTFMLAGMMMQVALQIGLHRPDHAQDFSKFRIEMRQAELQDRVVTWCVCNMVAQRIATAYGQPAQTVYDWTLGPKPIESNPSYELPAEIYTRLQIERFVDKVSRTIYRNPNDPVGLPDDHTRYQLVQILTQDLKELEQKMEGDQSETSAITRIYLNAAALHLHLSAFFSHRDLPSYRTDMLALYHATVNLLEACLNLESDYSINLPSNYSHGLSLSHGTNYIFHMMLAAGFSLLSLMKHFLPQHNLDTQGASGLLTRTLWALRGSSVLENDLAERLSEVLAQVWKTGAKLPEQISADASEEPSDELQLKVRCRMSMSLVFDSVWRWRKNFRDQRPPPARFFRANGEGRPTDPTLAPASSANSNETLAATAAAAAVSTPAIPGMAGMPTGIADDFITDPYAASNYEVFDPMNWLLDGTVDFPFNFQTNNGADQMKYLSTDI

>CF317_000027-T1 CF317_000027

MSSSSATTTGGAGQAVLLPREVRYKQHMAEVLVTDRKCRDNNARAKTTVESRIAYEDFIGIPRAERLNQIVAIFMAPEHPQRALDSVMARSVAETRLALEEAVYDFDGPSGDSKDAKVYLESHNAELRQRLF

>CF317_000028-T1 CF317_000028

MAATHTSSSTTSTARSSEVDWTRGSQVSTPHESPKKLPKKLPKKLRVPQRLRPNNTQPTQNSVSSSATYFPGTTSLVEWAQDKQTRDFYSSMHKESLRKLRIPQRLGQNHTETTQDALSSSAKFFKQTSSPVQRAQDQQTRDFHHAMRERSFKLRVPQRLRGNNTTEIPSNSSSLVQWAHDKQTRDFHDAMRAQMSLLKLFKRNS

>CF317_000029-T1 CF317_000029

MHDTSAIDPGSTILVTGANGLIASHVADQLLTAGYNVRGTVRNLEKCAWMPSFFASRYARKDVVFELVEVADMSLDGCFDNAVQGCTGVIHTTSSIEMQASSPEPTITNNAKTVMTCLESAAKETSVQGFVLTSSAWSISAPRPDTNFTVSPNDWNEQAIKDAYATNTPASNGMSIFMAGKTMAERECWRFMRERHPAFVFNSVLPDTVFGAVLSPEHQGTPSTAGLIRMLFDGQGLDILQWIQPQYFVDTIDCARLHVAALIHPDAKGERLLGYAEPWNWNDMLAMFRKWFPDKQFPKDMQLGRDISTVENGKSLELLKDVYGQERWATLEESVDANIASFLHSNSQAPMTFFERKP

>CF317_000030-T1 CF317_000030

MVHAEFFSSNGAHRIAIISEEPELVRIKYIDTVQSSGTCKGTILLLHGWPQTSYQFRKVIRPLADKGFRVITPDYRGSGDSSKPMDLDGYRKVTMAADLHSLIVDHLGINDKVHVVGHDIGGMIAHAYAASFPCDTATITWGECPLPGTTFYHDTKGSVQLFHFNFHRVLDLPETLLKGNERAYLKHFYDKLAYNAAAITPDDIDYYTEQYAQPGGIRASINVYRMFEKDAEDNVTMREKNGKCTVRCLTLNGNKSFLATAAADEAAEFYEHFETAEVADAMHYIAEENPEGFVQELLKFIEG

>CF317_000031-T1 CF317_000031

MLHIDPLQGERQTVEQSIFALLVLYKGPRRLLRLFGKQLSERPFGATLAKKDMRQFVKHDDVFLQAVDRLRNINKTQYAKHFLNFHKANDFALVELSECSTRLINGSHLALVGWLMRLDDDFIELLQQRQNLAILILMHWAVLLDRPTDTWWVIRSGRTLVYELSESLSDLTKEEDDARAWCHLHVASGNSPATNGHAYYAREGGVPLKHAA

>CF317_000032-T1 CF317_000032

MIIPIRCFSCGKVVADLWEPYIDLLTQKVPDGEALDKVGCKRYCCRRMIMTHVDLIEKLLKYDPSERKVRAAQ

>CF317_000033-T1 CF317_000033

MPSFYRSAAALAFAGAAFANPIQQSGDACLAAVTGSAALGDANLRKEHCTSFAKTIVTPSAVTVTTTITGAPSASVTEWSNYKRDVTVCPNEVPNYASACNEAGYKSACSAFGVSGSDTTVTIPATTSTKTVYVAKGSNGGNNNNNNNNGVCTSTSTVFATQTAGNGGKNGNGGDNNGKGGNGGVSTVTSTVTKTVAQATVTVTQTAGNSNKGQSNNKGDNDDNDSDSDSNHSNGQNCLTDAKAKEFTDAFADLLEHTSYNGTQGAPGSGYHYNVSAKYLAEDFQDYSDSINFMAGIPLGAVTFGSRAAFDYGQGVMQPELSVETMLVSHSCSEITWRWQATTQTHANVMGINHMILSDDLSQIKVNYAEFDNANWLYSFGLDCKVSSNSSAAH

>CF317_000034-T1 CF317_000034

MVHNNKDEEEDEVSSNLLHVHDLPQIYTRPPAQALLGALALLRKPTSTFGTGPSGSKDRTTRTHIDPSGVPHYLTSIIGASLEWLTEDDQERIWELASVRLSERSGRSAAPAMTRSFKISEDLTLSLHEPSLTEDNLGLKTWTSSLLLSRRLALLSRHVPAGRPRVLELGSGTGLVGLSAASTWQGSVSEVMLTDLPEIVPNLQKNVALNQHLLQRGTPQVRSRVLDWSDKSDAPKTADEAYAVILAADPIYSPEHPAMLVDTVRRWLRSAVSSRFIVELPLREAYAKERQHLRQGLEAFMDMVEEDEEVGFDDWETADGRQAEVTCWWSVWQLRNIA

>CF317_000035-T1 CF317_000035

MPVVIRGAALEGATTSTSHDHDHDHGRGHTSAPTSPSAHTESRPPLDSSQSEENDRVAALRSWRSHSSRNAHANADELAVTSPTSPSPHSRHVVFRADSNEPPTASPHPAARDRRRSSLSLFRHNSEDRPEADAYYDSRAATKREWRRRATTLQHYYDENPELLPQLPFTWHRGWKRWKLIFLVFLMWVDACAVPIALYYGFTYAGEIEGWITFAVVTTLWGGPTYVEFGVRTIKLMKKERFYRPLGTNSRWCFDYLNWTSTIAIFVVTALFIVGSAPHEVWLRVLCMPMPAMLYALAILTFTPTLYCRQGWKAPFRLSSTGKGEPVKPAVYYFIEDTVAVNANAGRPYREALAARYDASPRFREMLYTQSMFWSIPAFILAIPLTIIAVIPPVPPQVAYAFCWAIPFIWAAIWGLISLWWCKKQMIRERVEWEHTNPPLAHKIKAASRGLDTETSSATPPPPEDRREIERDQEEIARLNEATGRSRPTSEQMV

>CF317_000036-T1 CF317_000036

MDGRDLSTGTRAEDAITVEDNQDAIDEVDSAFGDSDNESAYSTSIASSVYRHVYENGRRYHSFREGSYMIPNDEDEQDRLDLQHHIWKSAIGGALYTAPLKEPQRMLDVGTGTGIWAIEMADEFPAAIVHGTDISPIQPEWVPPNCQFYIDDVESPWIFKQPFDYIHGRSLGGVIGDWKKFHAEAFKHIKPGGWFEIQDFEGFIFSDDGTLSDDSWIVHWLSAMDRGSRQFGKSFNAILEQKQNMVDAGFVNVRSQMFKLPIGIWAKDKKMKEVGKYFEIMMLKGVDSYTPALYTRVLGYTMEETQVLMEHVKKEMVDKKIHKYGKYHFLIGQKPEA

>CF317_000037-T1 CF317_000037

MFPFPNDLLSAILVAGLAALACLFSLAVYRLYFHKLARFPGPKLAALSQWYDFYYDVYLKGQFVFKLQELHDQYGPIVRIAPDELHISDPDFYVEFFTKQGPQSKYAWAANRFNNGTSVFATVPPEHHKLRRRALEPMFARAQIRQLLPMLQEKTAKMLTKLRKYQAEGKAVRLDRACMALSEDMIFEYGFGICRDALDKPDFRDPLHEVFVAAGSSGVVSLHFPILPKLVNALPDSVVGKLQPDFLPLIKVRQDFQAELDTLQNEENLKSRSAKTHRTVLLDLLNNTELPPSEKTAQRHLDEAQAVIGAGLSTTGWAATVGCYHILSNRAIVERLRNELFTVIPHGEKRQDCTDLDWAALEAFPYLQACLKESLRLSYGMSARNSRVTHKEVIYTESAQAVALPAKDSDSDTNAKKFSPGRAWHIPAYTPVSMSIPLMNHNEHVFADSRTFNPDRWLGANKVPDRYFAAFGKGTRMCLGMQLAWAELSLMIAGLVRWFDFELYETDERSVRMGCDVTIPVPQSDDGVRAKVKAEFD

>CF317_000038-T1 CF317_000038

MIGYNRLGSRAVMLSSLLLSTFLPTTFAENTTIPSSSVTFGDNGVKLAVNVPEGSAKTDLYFTFEAPAKDHDWAGFGMGTAMHDSLIFVVYRSADNQGEPTLSPRLGEQHSMPTFTNQVNTSILSGSTVTDDKFVVNFHCVNCRSWDGGSVDVASTDAPFFYALGPKGTLQSDDKEARINRHEIHSDIFTLDMQAATGANGVPVIGSAGDVDMNEVDEISGSTYGISRGVAIHGFAMCFAFALVFPAGYLFLRIFERLWLHWGIQSFGVLLVFLGVASGIAVSIREQLSPRLTHPHQIIGLLTFIVVLAAWTLGLIGHMMFKRKGIPSPLMKVHRIVGPSSILLGFVNACIGLAWVGIPRAIIGYTVFNLLVVILVGSLVMMKKKRKMRRDAMNSNAAQNFREGTTAYSHVRGGSTQQHAPANFAAPPPAYGQAVPLQTFQQQPSTHGPTEYYSVQPNK

>CF317_000039-T1 CF317_000039

MSGSRNSSDGLLGDMFVRAFNVGNYSADNQVRGFPNNAPQGARPDTVVSTPEETADSQQDETVSASDSRSTLNPRSCVTCRKRKVRCDKREPCSNCVKANIDCIFPPPGRAPRKPRRPQDAELLKRLRRLESVVDSLGAQVDEDGNPVPADPGAEERDSSGLLDREELSAGRPSRTTSLTHGLGRLVIKEGRSRYVSNDIWSSLGEELADIRNVLDPSSSDEEEDPLTPEHANDPASRDAHHQGFVFGYSSLIYDMSSLHPGPSQIWIMWEIFKENVDPLLKILHAPTVKHIIMKAAVSNTTLSRASEALFYSICFASIISMTDDQCRQLLGDNKERLMQKYRFAVEQGLARASFLNSSNLVVLQAFVLFLTVVKYIDKSRSIWSLSGLAMQQAKAQGLHRDPTIFNVHPFESEMRRRLWWHLSLQDERSAEDHGAEPNFHEHLYDTKMPLNVNDEDIWPEMTEAPREHQGATDMTFCLIRFELGDRHRRLRLMTRVENDAHKARTSQENKTLIDLTHQYMEEKYFSHCDPSIPILCVASTVGKLILAKMWLVVHHPHLYKGGKQATGDPKDQVFITSVEMVEFGTQLTRRKETAQWGWMAKTYIQWHSVAYVLSELLHMPPGPEFDRAWNAIDSIGDKRLAGAQRSQRVVMWRPLKQLYDRAKLRRDQLGGQSTTSPHSHTDSQHTPSSDSLGFQTPGNMTMTENLLGANPYTHALTASDGGFVWDSSGSMFNGAPFTSMSISNDMSIPNNMSSSATQRVGMMPEPMGAMPFGFLPPDEYFHGYGMQNQQPVWQPQMQ

>CF317_000040-T1 CF317_000040

MSPKIIVGLMGSSVSSGSTLLSTPAQLTSFLTVLKSHNIHELDTARVYANGRSEELLAQTTANADFLISTKAPGFTPQSLSHANILLNSAKSHAALNQAKVDIYYIHGPDSATPLSEQCHAFGELYNAGRFARFGVCNLSAAAVQEIHNICVREGYPLPSVYQGAYNPLHRSAEHELIPLLRRLNIAFYAWGPLAGGLLAKPIDELLKPKQGTRYHEMPVFGNMYLKESNIVALKKMDETCGGAGMSMMEATMRWFMHHSPLESRDGVILGASSKEQIEGTLNACEKGPLPEEVIEGWEQLWKNVVESGTALPASF

>CF317_000041-T1 CF317_000041

MSSTDIFKLCLVATCLVADSVLVHQSKTSFLCYMWKPLLQHPKRILLTTAGINCLIMICSASPSLLLGAPAVTCLVLWVYVIWVHLICKLVQRAGEVTAFREEHIAYINGAIVYYRDMTPDEISPYLDDDGYTADNSSDLQDDEGNESEDEDEDEGPEPDSSEDQADHKNK

>CF317_000042-T1 CF317_000042

MAALVQTLPQSTSTITMLQPRPNSADRYNAIPSHHHHGSRSSPQKYGSTSITGYRGVPSSGPVAPYAFTSTPQLGNATHRSQPSSPIIAASMAPQLPSLNLSGSSLSNAFSTKSMPPSPSATGSGFSVTPKPGEDMPRSTSSQSINSVGGAKSTPDRYRKNVRRTDIGGSVTPKSHGSAVPSGSGMAAVGQLYNHPTQSSSTPSLNSNSSYRGTPRSPHDDMQPGRQQSPEVASRYHRRSVGVETAGQHHGSGDSFDQSSPHSNAFIQSAHNEHSSVQHPHRPSSGHKHTSSSDSISSVRSGHSSRPASARNDSSNSMQHGQPAQMSHIAKQDIRMVNVPSRHGDNNRRSIAGLNNPSPLSRPMSPHAEDEAYARQGPKLSRPVQAPSPAAEHLAALNLKDTKKSKSKLRRAFSFGSAAELRKVTAQANLEKTNSTPMTNKRTSRREEELDPESARVAAKQEASGLGESIYSGQGHFFTGSTDNLSISSTASSASIMLRKMGKGVKKGGRSFVGLFRPKSVVGVPAADGPVNEASAAQVSMVTVEAEREKVNVNVDPHTHQGGGTGFPKLERNSIDNKMSLDSGTASTDTRSRRSIVGGERERAEVLAAVKKGILKRTDSNQSSPSLRPVDARASDLHLPNISHANDSPRSSAPSTPKDEPLGGHRRADSVTIHDHHDDYFGGAGSSRFTSLDSKTGLPVTPNRNISFSPRLQFHDTWPSGEYDRRGEIATCNRLTPMLAQQIKEELNSFKMEMEVHESSKVYTHFF

>CF317_000043-T1 CF317_000043

MSDFKGIMKGGWHPKGKNGGKESWRGDFKGVDQVAGWMGKGKTSDTEAAREHISRPLTTLKDPAAFGPPPKNVNYHGGAALPNQITPHRGGLGAPLSPSEISRANSSTQLARQQEEEQPERPPPPAVPYRADRTGLKTDNLPPPPVHRNLRQAGVLPPPSSSPVAAAAQPPLPPRLPSRQNTLSPPADDLGPPSYDAVTTQEPATTPYINQSAVNRLGNAGVSVPGLGIGKQADSNPWQNERSPSQSSGASHMSQIQSRFARMNTQNPPAYEPAVVSPPPTQVQSPSQSPMPSWKQSQSAFQTASNLRTNPQNVSFADAQSAAQTAGQAQRSASAFREKHADSITNAQTKAKAWDQKYKVTSKLNKFLDEQTESTPAQQQQVQQPHMQQYQSGQASSVYSPVSPTPHYSAATPLPVQSPSPDVSASISRKPPPPPAPRKPANLQAPPPVPTGTKPSFG

>CF317_000044-T1 CF317_000044

MASRSGFNGPFTPTKSIDSASFASMASDMSADMVAFATATGSLSPQQSSLQASLASRLGKVNSAASASPTTSQPTTLDTVTSSAISSAPTESVAASSASASQPAASASSTPAASTALSSAIDNAASSTAPTASSAANNSANDLHAESVTMSSPGMQAAVAVPIVVVVLAVLGLIFFCVRRRRKRREATDGTVEKTPGVVAAMKKRNWKRHFRLFSFDTELLMGGHYSSTNSIRSRETGSLRSANRSQHSGATSLHSVDEVAPPYRDAINSAQPPSIAQIIGGGAAGAAAAGAGAGAGTQVSRTTSNATAPPPYGAAGSLLAVPSPVTPASQRSGRNPFADSTPVSSIEGSPFNDPPGDSPPLSRQNSSVYASTIGAISDAASIREATIARNASVMSRGRVINNVANPTDAS

>CF317_000045-T1 CF317_000045

MSWTYTPQSNHPYVDSCYPKATVPTTPAQNSNPDWSPPSTEPWLSSTCNTSAQGRGKRKSRASPAAQARGLGLSVHIHRQPAQYPADGRPAITTTTYHQPQIHYTPIDPSLRLASSPAFFDPPHSLPAVTSARAQIAGLYTPPLSAEPLRPSQQQQQQHPIYRTATMEQQRRMSSQGHRPLMGPPETPSRGMIPQSPNLFTSMQFSPDLFSQPMLDPQSVPVFPQQRLFWDPATATPLQSTPQQFHTPTAFPDDFSASFNSNSTIMPLDFGATPQDMPYDLPIVSQSMSSSFMHGSVFPAPFQTSPRLAPPPAENPTQFLSSPARRFGGDPKMDNLNARRTVPELPAYYHQMQESKREKELLARERRKSRTLKRSQDEDMVMKSVTRALSPRKTSRPGLGRSATFAGVHKSTSLDTISLDSGTSSRSNRAGRSSPIRQIRDFHRRSSASASLSKRSSSVSLAIDQNGVARTVMNTLPETDDEDMDDDMTDQLTSEDENDQSMLYSFSGDPHAITDSFMSRGDARDALRSSAYLSTGADRVARVSIGGPSLLDPRLGQDATSTIKKPRGETLTSTNTDTANGGNAQQALRIMMQDRSTSASSHASTSSMQFHSSPPMQPSHLPALNRSPTTTTDPDLGTPSTDAGSTSSGTRCVCKRAPPDGKIMIQCDSCSKWQHAKCVGLNNRNIPDVYVCVFCEQTPVRAGTIRPNLRQASFQKSPLAHKSNRLR

>CF317_000046-T1 CF317_000046

MAAVLQPTIFSPFDTALGTSSRRIEGSSNIPEHAPDLDKLSVNTVIHDNSRRPPPALASSITPAAPQRNDENLSPRARLRDGGPQLRSASSHSSKSPEASVFTAQLRPRNFTFQPLMHDTDIVKRPEERADTARTKLDRQDDMVDLSLHDKWDTEQPTRKQRPVTPAVTSKREQSHDKSSPITSMQPLEPNRNAMITPETGLLPRIRELRQKTTSSETDTSIRSPTPGRIPVHYQRLSDGSSGCVHSMRTASFTNASFSVFPRSSRVGMSTDSAVFYGSQPRYSIDSDRPLTSHSFDDIALKRGFKRQQIIDELISTEESYVADLKALVYLYSTLLASASSIPGRLRSAIEQNVEAILHLHEQVVEKLHSARLHAAARRWADTTVPDKLGHHRRFYANRSVRGGRARTPTFHRHTRSDESADAFGLHPQQAGSAEPIDVYEITAVVKEAVKEFYVYEEYCANFEIIRHELQKHMPQLWSTYESGMESLAKVIMAVDRQRANDHRGLTVGDLLIKPIQRLMKYPLLLGQLLHSTPVADAPGTHAELDLVLQSVREMVQMVNLARDNQYARAQIQRRWLLQDRLDLSRLSITPEQFRYFGSVELCGVLHIAYQTTTAVSGGYALCALLDEHFLVAFPVANTGYFQAIALIQLSDVKIESATDGKGKSLSMSRLQCASTFFTWKISFACAGYYHELILSACSKTEEDHWTAGLRGDFGDPVHRPLDTELAAATCLAIDLKSVGVVFSSQKSLSRESSVQRAATVGGRSAISQVIVRNTHNAQDLHEYRGPTSSFAPINRSQSHLNTKRIPVLEPKRSERTRLESSIGDIWTKERLPFPGMVGSRSGQMLRASAGSLARKLSLASIHTPFSKARTSSLSMASRRSYDLFNDSKTTFEVRTREPTHAQASRSKPKEIPEVDDMRSVVGRMIGSSMPGPKKDQIVPELHVERSREKKSRKVSIRTVGPDDPAAVFYEGSDSFECSGGTGAVVDAKKISVVGAGSRGRRRKRRWSRQLSRLKGFGSEAKSILYSSSSGA

>CF317_000047-T1 CF317_000047

MGKLIKTHWSRLILLTASAYQLAAAIEGYFWPKIFWDVLTKNLDAAVKPYPVLQSLNLVFAIIGLCWEWPLPLVSRFLPSLHRSIEARMVVYPLAALAALLLYQGTNAGLYYLIGVGVLFWGFCEGEGIAPVPWTLPQIPEQEASRV

>CF317_000048-T1 CF317_000048

MATMKSHFAPAKIAFYILWWTFHWGLFALGWWKQAADPRLAALNTLTFSVWLSRGAGLVLSVDVALILLPMCRNLMRMIRPKFRWLPLDETQWFHRQCAYSLLLFTCIHVLAHYVNFFNVEKDQLRPQTALQIHYTEAGGITGHIMLLCMLLMYTAAHHKIRQQSFETFWYSHHLFIPFLLAMYTHATGCFVRDTAAPYSPFAGANFWNHCIGYEGWRWELWGGGIYLLERIYREIRARRETKIYKVIRHPYDAMEIQFKKPSMKYKAGQWLFLKMPCVSSAQWHPFTITSCPYDPYISVHIRQVGDFTRAMGDALGCGPAQAKEYDGLDPNGIYEIALQQGQEMPAIQIDGPYGAPAEDIFDNEVAVLIGTGIGVTPWASVLKQIWHIRAGPNPPRKLRRVVFIWVTRSIESFEWFQTLLSSLEAQSVDAAEASGGAEFLRIHTYLTQKVDSDTAANIYLNTAGYDLDPLTELRTKTQFGRPDFKRIFGAMRDGLMDQSYLDFGQSKLHVNQMRAQVGVYFCGPSAAAREIKRACKDTGNEFVKFKFWKEHF

>CF317_000049-T1 CF317_000049

MPTGQAGVTKVHYKGSDEDFIVFVENPEELKKWKGDRSIPLAQVVDSFKIFVTHKHGAQGQMDGASKATLENEFGTKNEDEAIIQILEKGDIQNVEVGATILSATAEEDKETTLKVVADKLTRGNARSPLDIVGPVVRNTA

>CF317_000050-T1 CF317_000050

MHPILLVYSALALGVARALYAILAAALSPTRHIPGPFLARFTRLWYFYSVWKGQAEKDNIRLHRKYAKHGQFYAPIVRLGPNMFSIIEPDKQIYGVGSKMRKSDWYEGWKHPSPERWTMFPDRDIKRHNDTRKKFQNLYALSTLKSYEQYVDDCAIIFQDRLGELATSGRYIDMAHWFQVYAFDVIGAITYSKRFGFLDRGEDVDGIMQALDSSMPYSTLVGIYAWMHPYLYPLLQRIPGSGAAGRQKLMDFTQTNKETREAQRRTWDLEGKGVEERPQGTPEDFLDKLLDMKKDQQKGVTDHHCFILGLSNIIAGSDTTAVSLSSVLYHLIKTPRAMQKLREEIRSLTEQGQCETDRVSFKDSQEMKYLQACIKEGLRMHAAVGLPLWRVVNEGGAEICGEFLPAGSEVGINGWVAHYNQDVWGPDANEFRPERWIEAEEEGGGKLRYLETYYLPFGLGSRTCIGRHISYLEMTKLVPQIVRNFDFELQRPDVEWSCKNYWFVKPADFDVRVKRLEKQG

>CF317_000051-T1 CF317_000051

MSGTATAQPGTGSDITPATDNNTVDIPPRSTSQQPGSSALQPPANPENQTEPPSPRRGSKRSLLSRSRQGSRSSRRSARHASKALDEGDNPPAVPQNDSARSERKQKSGGFLAFLNCCRADDGVDGLKSEEPDVPAKQRKIEPTPQRTVIPDEPVAPVVPVAPVASEKMTTQEQPYNEKRAESVAVEDEKPTPIDNPRANARPRVDRNEISHEKPLPVIDPVSVPSTCAPEAAQPAPSTQAQSPRQAEPTVHDLITLPPSHAADTVVTDQKPQLEFPPPPKDVSRESDVDMTDAPPVPPVEEQNVVDEPVQPHDVVPVALPPPPPLQKRIAQTQAPVAATDVPPVPDPEPESKQTWLLPPVQPHHKGRKCLILDLDETLVHSSFKILNQADFTIPVEIEGQYHNVYVIKRPGVDQFMKRVGELYEVVVFTASVSKYGDPLLDQLDIHHVVHHRLFRESCYNHQGNYVKDLSQVGRDLKETIIIDNSPTSYIFHPQHAVPISSWFSDAHDNELLDLIPVLEDLASSQVQDVSLVLDVAL

>CF317_000052-T1 CF317_000052

MSTKEPQEEPQQEPQPQAKTKAQKRREREREKARRDAEEREKSKPPGAARVNVSQSPKPGTKGLPTTPVSQSKGESSRSPKQAAASNVSAKGIVETGESSSSSTYPQANLERISSNNSKEALTNSKTPWSPITLLPATASINVPEPIVGQQNESTHESNAGETPSKALNQRDQKSKTPSQSLASIDVPLVQSEKSSMRVFQNGLETNHFSINIDEAPILYRYDCSEEIKRIETANSSGNASTGSKPQEKKMSGRVRRRVFVLLLENLRERGFEVATDYATHLVSALKLSDHRDEWTEHVLYFDEDKYKSDPNLTTFVITIKPAVEINLQKAVNYLTRGKSAHAAGYADGSDAETELARATDALNLVFSHIPNQDTVRHAYNSQDGKATFGREPGVANIGADKYYSYAFTRRALPLSKSPKYLHLQPSTQGDALWSLDRDVEPKGLLALLGFFRSVRAPVGPGFLLNINAVTGCFYQWINLHDLIMAWWGNNAGSETAFRDLEGFISGLRVRTTHMTKYDAWIKLAQSQKIVPRERIFTISGFPGTANLANDRDYPWVWKNDVLEEPTGGKVEFKPKNDQGEELAITTVKNWWEKRGYKFQQYEYIVRVGDKKPLYYPAGVLEVLPGQAYKRSIQMVRQACRVPDSNFNYITKQALNLFGVSGQLQDQEVYKKFNIGLEPNLLKVNAFQTRLPALQYGNGTVTVQSHEKAKWNLTQSTLLRTPTAPFRYAVVQIQDTNTTLDTTAFQNFNLLFQNHVNKTFGHGKAVFVNGERDWQLACTVNNARTYSTVKRAGDLEVGIPTICLQAQNLQKDRHMDDFVANVLLKVNMKISNKNANHTLASLGGKFQRPKLLGNRTMIVGLDVTHRGNGGMKESPSVAAMVASRDDLFTQWPVSWRLNPVDLESGKKSNERVELIGEMLIERLVHWHHANDRQWPDQIVVYRDGLSEGQFEMCKTQEIQWMSEALEKHSVENSITRPRLLVVCTVKRNNCRFRGQGKGSAGLTDKNHNACSGTAVFDKVTYGTGKDFFLISHKTLQGTARPTHYVVLHNEIGGLSLHDIAETTYHLCFTWARSTCAVGLVPASYYADKACDRARNYLYKLYNDFGHLPTVRFDWNNPNHLELARIPQCNDRMVYI

>CF317_000053-T1 CF317_000053

MNDHAPSPDSYSSSRQDTTRNATSNNNSKQADHHEAQTPTQITRGFDAPPAYDQLSIAPPSDEEPSAQLSSTDTNTASKDNSVKDRSRGSTMKQKWKATKEDDERRRAGRYQTVSATEADRITRLDRHREEQAKKSSEQKRGGASLLGVLFLT

>CF317_000054-T1 CF317_000054

MSAKGKGKPWNKVGNTSNAPTATEQRHRGLLPSPVTIHHDGHLKSNHFKVNVGNDYKLYRYPITAILEPKKPKQDGKDVAADTPSDESTNSQPVPAGFQKAKLSARQQRRLIFLLLNHLGKDDKDHVIASNYVDTLVSAKPIEKPELPNDILITFYDEDEVSSSARATQYTISLGKVKIIEVGRLSQHLKNPSVGYGSFSSVDAGRNEVAEVLNIALSHHVHNKTTRWARSTGGFDEPTVAPVAPNKFFRQDLPFRIGDPPHDVRDDTGNQQGLCATPGFFRSSRSVYHQDLLLNVNTTTSAFYKWINLANLIHEREPSAPALSHDAWRKLESFVSGLRVRTTYMVASGLSSHERILTVKGFADTGEYYAYQKLKAEAKKNNKPLLATDPGYPWNHQRGPFANRVTFSKNVLEKDGQEDKRVDCTVQEHFKDANKKSIQAQDLIVRCGFKGPLFPVSVLEVLPGQFWKQKTDLANIASHDPAVNYRTIIQEGARIFGFQASGPSSQGTTLAPFGLSIATDPNGDAALIDVPVRRIPMTQLRYLNANGGNAFVSQNPWFLTNRRAIQPAGGSKTALNPKQVYNWSLIEIRNSGYGGQQPGDLQHFADVFQKQMANYGMVNTKILCNNAGWNSHKLVDDVHVGDGVRRILKGMMNQPSKLLFVVIILPKRDLDLYARVKRIAELELGCTVVCCVPKGGKLPSDTTQNSQYNIRANMISKINLKSASTAAGHAWSQTPALLQGQTMVVGMDVTHPGSGALRGVPSVAAMVASHDRNFAQWPASLRANPRPDRPEKGQPDEKYKQSQEKILDLEGMLFERLDCYARMNQDRLPAKIIFFRDGLSEAQFISCRDEEIPQLRRAIEKKYRPKDLPNKWRALPQIMVVCAVKRHHTRFYAPAPQKVEQLLLPSKKGPRKHPLPGVTVFDGVTNGKYDDFFMISQVTQLGTARPTHYVVLESDFGNEFNIIDVAQATFDLSFMFGRCTSSVGLTTPAYFADKACDRARCYVRHEYVGNPPNGPAWSPVQNPLQLTVHEALKDRMWYI

>CF317_000055-T1 CF317_000055

MADQNKYGAPTGPPPNYPMSPPPAQHYDVGPYNQGQWQQQGQWGPPQPGYDQYGQNYGPPNQGYYGPPQGERGETWRNVQRLAVIYVALEMVGSDEGTTGTAFGSLDKSDGGDNG

>CF317_000056-T1 CF317_000056

MAQPGSTTWQAPQPTFWERTKNYSTTAYSKALKPGFDKAYAVVDKLGAPVNKLSNKVGSEAFWPTTLDKESEKCARILRTFCKDGFYDKIEEEAAQQALEARKDPTLNVPAGTQRNIVKIPAKAIQNAVGLAIFTTMRTGWLFGGSGGAGLVVARHPDTREWSPPSGIITQNMSFGFLAGVDIYDTVLVINNYRALEAFTRLRCTLGSEVGVVAGPVGVGGELDMEIHKKPAPVWAYVKSRGLYAGIALAGNAVIERSDENAKFYGYQVSAADILAGKVRHPPERDYKILSDTLKAAQGDNVDASLLPSGESPSDFDVDFTGSTFGVPALEDDDPYGVKALEAEGLHIREAGTHLRPSAEVFDFNPAPTSPIYKTFRKSIDSSSLRRKTDSWRNSTTSLNSVTRATQTGDEEGPSISDRRASRPVSVRNHSSNFSGRSTTTSPVKEEVIDESNPIHQASLREERREGKQKDEDEEDEDDDDIADLSDEIDSDVEIGTAVSTIARPRMVQVSKPVPPALPARNPGRGTPTLQLNSSAITAPDSNHFTVKLKTNQDLPQDRLILRTSLDNWTVDRRGEMKDGEFHFRLDTSQFSDDFECKFVILPDRWMHDPNLVVANPAPGEDITFTDSRVIFDPPSGEGATDFVEALEAVKLAHRKSSRTESDADDDAFVSVPPTPDESENKMEKANGV

>CF317_000057-T1 CF317_000057

MRSPTGASVYEDEPAYNDAIPAVINRCTVPRTLALSFDDGPSNYTNDVLNVLSAHNATATFFLGGILNGRGQMDVDWVPVVRRMVTEGHQIGSHTWSHPNLDLLTSEQRADEMHKMERAIANVIGKIPTFMRPPMSRCNTSCEEDMRALGYHVVQWEYDSRDSEDPRPSLDAMTDDNLEAAMNRTDVNGSMFIIQHDTHGQAAQVAHILLTYMHANRSGWEAVPVAECIGRDLSDAYRFPKYLEYNGVAPGGCLVSGPNMCVQPIAFKSWDGCLDARDALDRDWRQCVTREQPNTPHCVQAQKLAADMDRFCDECDEEGMPACDSDAFKSVQY

>CF317_000058-T1 CF317_000058

MAWILVTPASRGIGHAMTRHLLSTTPKSTPIVATTRASDPKETKDSILADLNLDHDASSRLDIQRCDLLSESSISDLAGYCKSRYNSDPKDQQHHLRLGVFLPGLLYPEKSPSQIDHDNALATLKLNLLSPMMLLKHFHPFLAKKSTTLPPIPNLPSSSLLAFLSARVGSITDNALGGWYSYRASKAGLNQLVKTGDLFQKITSKDNAAIVGLHPGTVKTGLSKEFWANVKKDKLFSPEYSAERLCDVLVKGGSGELAGALEGFRGRCWDWAGKEVPP

>CF317_000059-T1 CF317_000059

MSSKHEIPKQHRVQRPGLWLPEDHRTHQQWLERTVKHVDNNPKDFHPVVKEFQELIESNTRLYQLVTGMYDEIPKKKPYNKDPSGKMTIRGYKHMLEVLNHLLSTAPSWSDHSHEVGLVGLPIYALFDWPMGTTSGFAFFLDPQVNATLKKVLNVWGEFLQSSDSASCLDESTTGWFSETGREDLTQVANLNAPKEERTPFEELFQCDPKAKYHGYKSWDDFFTREFHWEHRPVASPDDKNVVVNACESTPYRITHNASARDKFWIKGMPYSVLDILQFDDTAEQFVGGTIYQAFLSALSYHRWHVPAAGKVAKVVHVDGTYYSEPLFETFEERGEADEEGQSDAQAYLSASAARALIFVEADNPAIGLYCVVQIGMSEVSSCDVTVKEGEHVEKGQQMGMFHFGGSTHCVMFRKGVKVSEFPEPGKVTTNWPVRNQLCVVEG

>CF317_000060-T1 CF317_000060

MADTTTQLKADDHLLLDQPLLRLPLELQRRTFKQSQIKIDHEQKKTTELLRNAAKTANSASPDDTIKSLDAMIQRMENLKRDLETLNEEEQSLHEQSAKRIRHLQELYEIPSLTDVKYDNWSRTRLDRLVVDYLLRAGYSDTAAALAESKNIQDLIDLDTFKQCHKIADSVRGGSTTEALRWVARNKDSLKKLLEKTASDKNALVPAVAPKVSQLEFELRFQDYIEILRRHRTQDTARFEAIMHVQKHLAPHSGAFPEQYRVTAGLLAADPSDPSECYREYFSPLRWTYLSDLFIDTHHQIFNLPTRPLLHVALSAGLSALKTPACHSSLNPSSAATDGHEHAKPMFAPTSSSNHIGNSLCPICSTELNELAKNVPYAHHTKSSVEDDPLMLPNGRVYGRERLEELERKNMRIAGTSRGMDDERDKDDQRGRTIKDPVTGDKYPWSKLKKVYIT

>CF317_000061-T1 CF317_000061

MIFSRLSTTATLLSLLLVPSYAHSVPRQSTNLTHWPAGAAEALDIMIAANADQGNYACFDMDQTSYRYDLTESLLPFLENRGVLTRDNLDPSLKLIPFEDTANYTETLFSYYYRLCDIDDKVCYPWIAQAFSGLSLSTLKTHVDALMESAQLINTTYLDDGVVTPISVNPPVVYPGQVELYNELTRNNITVYVITAASEEIVRMVASDPKYGYNVAPENVIGVATLLRNASSGALTSSRLQIEHGAYDEQANRALVYTPTLWSPLTWYSGKWAAILEYIDQWKKPVLVAGDTPTSDGYMLFHGVDVAKGGIHLWVNRREAYFEVLQDMIVENTAAQVEYGLPVTADKNWVVVTPPDIL

>CF317_000062-T1 CF317_000062

MAVQRHAFDTNGHAQDDDDDVFSSPPDAFSPSYHHRFSSRFDIEPLDLSSTSSPSQLKRTIEAHLQETDRRLEDTQQLGTSLLKQREDLSSKLEEVEQYQDEAHIPPDLQRRLADIEKEHADVSREVARALIAPKPKHDDSSLGDSGVFTSQATGSPTKLSAPSRRHRNQPSTRAGDLQFAADISTSLLAQVRQLQATVAERDDALRRLNAEREAIEHDLHNHTQKIRALDESEQKYKDENWNLETQTHELLTAAKEAQDKEKRLNAGLASTTAERNRLQTELEDLRMNHAKLAEEHTAARKAHDTEIHALKRNADVADTDRQSLQDKIDDLVAQNQELAKAFSSRSRTTQFSPGPGLTTMPDDSADQLDTPDDSPPASPTKATPRHGALESETLKSSLHHSHRMIQNLKAQNHREKTEKIELRRMLQEARDELESRREGGSAAKRQKTKPDVFKKPLRPDMLGGTRRPRTDVELTDDDWEDQNVDSPTHARSINVLQPHQGNGQHWTDLSDAYQTANDTEGTFDTADERNTTESEAFMTGAESLAGDSTDELTETEDTTSTAGRTAPGRVGRVFGARPAADRAGYVSSASPSADGGDHMQTPMLSSFTKFKLRNNKASFPRQTQLAEEITPESFRSKSVTYDSPASTVTGRQSPATGEQSLFAELGGMDEINSEASSTPARSSTMSSARSTPGYVYTPSRASLVVPESATKTMMVDSSTMTEPWTPETRRADSAQEKKLLPSAFPLPPSAPPSPLKRGDSNRQYTPQRAMSESPSRPTSSHITPPKTVWDEVHEPSTPGNVEEKSLPQHHYSGVLSQDSAPVHSPGHDSAPTRAQLEQLQADLAAKETELSDLRGSNAAALAAAAASLESLRDTHATALTEKKSQLDVSRSSHAADVARLESQVRELKDTHTSDLSTKDTELADLRASHSAALAASAAELEDLRASHSAALAASAAELDNLRSAHTSELANRQSELEDLRSAHASALEKKEAEVEELRQGHATELARKDSELNDHRNEHATALETKEAEVVELRNSHAAEVDMLREQLSSHREEIGSKKMELDGLRDSHTAALASSQAELERLQATHAAESDKLKAQIAGHREEVSRKRSELEVLRNAHSEQLDNLRGQISGYSQEMGTQQTEFEKLAAVHNAELQGLRRQVSGHNEEMSNKEAELHTLRSTHNTELEGLQGLVAAHTQQLSSKQSELDALRLTHNQELERLRGQIDDSAKDVGRLQTDHDQELTKLQSQISSYTQQLDSKQSELGTLQLTYNQELERMQRQIDDNAEGVERLQTGHGEELAKLQSQTSSYSQQLDKKQSELEALQLSYREELDRLRNKVGIKHNLIQRQHADHSQELGRLHGEIYTYRLEVEQLQNALHASNARHAALRGSGYSSILSQETRPVASQLAEPVRSGRFRQSPERPRTAEKTVGGGLFTSPAGASDFAAAKRDEPMAVREDGTSGTIENVGQSTSSALKDISGNAVPTQHGHGADDLGGSLIMPPSSHDHGSQTLLRGEQLDQGSKPRKAPLAPVIIPARNHSPATRAPVSPTRHPRRSLSRTREFSPIVEDPTVIPSTPPPRPSSATSNRSSSMVAHSHPPLPSDHKDIIAKANQNVSPTRQGSIVKQGGLMGPPIMPASAMRRTKTPTESVRTASRVGQSGKRAAGSQMSRRSSVSSFASEIDERFNIRPDQQAAGPGFVAGPGTDPRMIQAITQTMIGEYLWKYTRKTGSKEMSNTRHRRYFWVHPYTKTLYWSDQDPQTAGRAELKAKSVAIESVRVVSDDNPMPPGLHRKSLEVTTPGRKVKFTASTGQRHETWFNALSYLLHRAEQDVSGNAVGSPDDITNEDIAEFSVNGYGAHLVPNDSRMSMSSYNSRTTAGTSRRVGRQSVPVGGAAGYSSSQLPQPQGMQSSTMTSNDDTLSRSRLDAPQDKDRTRDLDSRMLEPVVMGSIMSEV

>CF317_000063-T1 CF317_000063

MAFSTLYARQFDSSDYDGQNGLLFGLFVLFMFGVIMSVALIYFRRRRLARQAALLPRYTRRGHQRSVTITTAPRYAGQDKVFVYEEKMNLIANSHGRPDSPVPEIRVTFPDEVDQLGQKLSGRVVVVRIAESGSVGLEPVAQEQLPAYQQADSGRFQSLDLNRIGGLKEGSAAGAAARCR

>CF317_000064-T1 CF317_000064

MTSTLPANNLFDNNPFSSTAFYYGRPAAPSRTYTGDSFAGTGFGNNIWQTDSRNDLVTRDYVRQNTARRPLELSLSTTAVKNNVLSFGAPGEKRSPVTSDAGTARLPDPFSAQSRSHNESAVATPTSTTFTSQSENASVVGNSKQDPKGKRNSGYGSHVYQGRPNPWVTAKEFQPSLSSDASRTPTSALDSTRRAVPNFNTIHLGDNAVNGLETIWSQDDRSQSLHQQIHQNDFRPVLPASENDRNAQNNGYGNGPRFDGTTDVLSEPSLDSQPTPRVDLRSNTYQQVVYPNTSRIRQHKVQQPQGAELYLSGAWPQGSYSQQNTAINSLRTSLDRPYIPTSGHVSNGVYQNDFMPKYGVQQSAHAINTSVELGFHSRQQYNEYLSRAVRPFTTQTYNDNHYSTSFNGMVGVRNGGYGPDPALATYALNDPALNRQNESSQICRSPLLEEFRMNGKTKKYELKDIYDHIVEFSGDQLGSRFIQHKLEVANSDEKERVFSEIARDSRQLMTDVFGNYVIQKLFEHGSQSQKKVLASHMKGHVFALSTQTYGCRVVQKALEHILTDQQASLIKELDGRVLKVVEDQNGNHVIQKAIERVPGEHIQFIVDAHRGHVHYLSKHPYGCRVVQRMLEHCQSRAKRQILDELHLNISDLIQDAFGNYVVQHVIVSGEPQDRKPVIDKVQSKLMENAMHKFASNVVEKALDYAEEQQCRAMLHKLTARDENGQSQIIKLLSHQYGNYVIQKCAARLSGHALEDLIEQMEQYLSTLRRVSNGKQVTALDGSIRNARQRLHSQQYETGMHIAPTQPRGSVGRR

>CF317_000065-T1 CF317_000065

MAIELPTNRQSRNLDGPVPDIYDPNYIPVLNGADIKADFSPSSFDALPGMPAPHQNPLQSSLKRSKSGSAPERVVHTPTSSPRRAQRRAANRPTPPLSPQEIGSTDDPLRNNDKPTGSSEDNRTESSEASTVDVFPALEEAVFMVPGNATPRVEEIQFPMVPHSARSSISSGKKSFVPTSKYNKKQPSLPSLPSGVLPQHRYTQSPLASRAVELELNLIQLQQLQSQQQQQQQQQQAPHETARATPSPTTSPRKGASSAVPPTSTPRMQSMKSTASERRKRALHSHPADSSPRSKRTSIDEEPADVPEMPYNIITRSRASSVRSRSQSICHSVPSTPAPDAPLPELPANSPPRTTSNSPSTFPSPLPPVPQPDHTEMADFMKSKKTTVFRRFDEVHVRLLLHLQDETSALEKELLEIEEAGPARPDTTLRKANLMRELRKVLAEYVFIVVWLLGDIFNILGAVLQGVLPTMIILAIYYTLADIVLLLQLFYYRGFTLKDDVKKPADTDDESRPLLSDPTSRPDGQRRSSFGEDFRRSGHHLSPATPLHDAHRDPPAVVNNKPTTTIQKVLFNLAAVLVVCAAGLLGWYISVHHSGKHHHHHHHYNKHDSEPPEPLEFDFLGQVFGYLCAVFYLASRIPQILLNYRRKSTEGVSLLFFLFACIGNLTYDMSIFAYNPENSCQRGPGRCEPEEARAIYARYIAVNASWIAGSLGTLLLDGAIFVQFFLYKKDDDDVSSTSTDANGNGHVVPEQAVSD

>CF317_000066-T1 CF317_000066

MAPTPKVNDFVDFSDIDPLSQNHCIWHLPDKDRRCILPLNPENRSNAAALKHRIVHFTDTDVSLATLERYALLNCCRRWHYTRIEEVGIIQPLARRWQEELRHHQISSITRSPQHATSSIGPLQSSQVLPALPASITPRSESPVFPRYNLRSQGPVESQSSAPLAAESSSHTPEFRPHKVYPPQNVSSVLLKPLSLTDKKPGSIYLFSRTTSPGFIKIGYTSRRVQTRLAEWKRDCGYVPVLVGSFSDVPNVKRVETLIHFALYKLWRKEVWCSRCARQHVEWFEIATDEAVGTVRAWTEWMREAQPYDSNGMLSDFWRTEVQRLEALGQAVTAEALLDIYHADRKRTSAEANEDGPLSMGISTPAADSTVANAWQRRAEHSSTSPVHQTQTHSVHLRSPGRYRL

>CF317_000067-T1 CF317_000067

MASLSKKQKGGVPLSGSTPIQTDDAVWQDKSNALWLTDIHTAVLYGKPRGLTLLIHSGADVKSSDQSGHAALHTAPSLSLPQKEYSLDVWEVEEQMVGCVWDHEEE

>CF317_000068-T1 CF317_000068

MALTQNDLARRLRSLHVSGDPLLLANVWDGASAAAIAGHPSTKAIATASYAIAATQGLDDGDMTLEQNLAVVRNVVAGLRKVGKAEELPLTADLEDGYEDPANTVRRVVELGVVGCNIEDVDNRKKELRNIKDAVARIKVMADAAKEAGVPDFVINARTDVLGYGGDISTVIERGKQYLEADATTVFVWGVSKWDIKSDEVAEMVKALNGRLAVHPGSIGIEKLRGLGVSRISVGPALWRGSMSVLEKEGVRLKVLR

>CF317_000069-T1 CF317_000069

MASITRSDFGATTEGLTVAAAFPTAIKDRTIVITGINKLGIGYTTAEALASQAPRCLILAGRSTTKVQECISSLHAQYPNVDYRLLHLDLSSKKSVHIAATEVLSWIDTPTIDIVINNAGVMHIPTRTLSEDGIELHLATNHIGHFLFTNLVMSKLIAAAKASNTPGTTRIINISSVGTAISALRASDPNFTKPASQLPEREKPNFAMMRMAKLQVDEDMVYFPFAAYGQSKTANVLYSVGLNARLFEKHGILSVALHPGEIKSELQRNTDPQWLEGVTKYKEKNNMPWKTLRQGASTTLVAALDPMLAGASKSGPGGNEGCQTLFLSDCQIGKTQAYALDKRDAERLWGISEAWVGEKFECREFLANGWTATVYRLKNTQKVIKVLDKESYPREELFQRERRAYERMAQQTRPSSILEFYGIDDPLGLILELAENGNMHGYLWDCRHANAAPDDSTLLRWARQAAEALSFVHSCGIIHCDIHVANFFLDRDLNLKLGDFGACALDGEKPLMAYRRTHQLWVKEDEKWRKAFSISAEIFALGSAMFNMETMRDPLEELDNERDREEIARRIREKELPDTESLVALGEYVKKCWNLEYGSMSDMLRDTHESGEEYVHRKLEL

>CF317_000070-T1 CF317_000070

MVVTALPGASPDEYKVAVDKSSFDGQYTAASVIVTLSIGLALYNSLEMILLISATFKRWRGLYFWSLCICNYGVISYTLGMMLMYFGLGNQLASHIVLDTGWICMVVCQSLVLYSRLGLILDNVKIERAVMWMIVGVSVLLIPTVLTFDFGNTYSNLATFPEGYFYIEHVQMTVFTLQEVIISSLYVWKTINMLKIISKANTRSMVWQLLVINVVIIGMDIVLIYLQFKREQLYQESIKGFVYSVKLKLELNILSKLVDLVHGGSANRSMTLDVIDSRALPGQAQKNVQRELSGQEYFGSLFTSDTKDHVQHVENSHVIERSHEDTIAAV

>CF317_000071-T1 CF317_000071

MAQTDDLTPYSKSFSDLLRSLRLNPYVRPSETDPERLLPSPIGRHTVSKTQDIMVALLGDGFGFGSSELGLGLEVAAGNWFDGRTSQPRRVGLPATNLAKEVGSGAPKYSQERSVSNSAARGEEVQLNRHRLRRSIFSRLPARVRAKILKYLSQNFDLGSELIEALADVTSAARAVADITRGCTFVNEFGWAEWDEELGTFCYGLRPTWHYNSDMRVPRWVFNGVGVRVEPKEY

>CF317_000072-T1 CF317_000072

MSAVTKRILLALLCAQTCTALYVAKGSPCSAACNSTGGSWWTNLVCSTDDYTSTTTGQTLRSCLECTVDSTYVNSSYTDGNSDQFWTLFHMKYVQQYCLIDTTTPHIAAVNACTSACSPLGDVLETLWISSHPFLGQYDYCSMNSSAYTNHAGDCARCLQGQEESVIIGNFVDTMNQACVSRPLAANGELLSPTRALFETSLPSSTGASSTAASTSGSSAAATQTSSASVSGSSAAQSTDETTEDDHTAKSSDSGGLSTGAVAGIGVGIAVLAIGVITGLVFLFVRRRRTTKLAQSSTAAELPVNNGYNGMAQDVSHEKAAWGSEEQLHEAPSDTKGHFVELPAADR

>CF317_000073-T1 CF317_000073

MSGNYDQPLALVVISSISIGLGAIAAGCIALDIIVRRGWKSMMWIMIPVYVINALYLWPITLWVYFKYGRPSKPGAGGDQVSHCHDGGAGGQDMQQDGTEQNDDDSDRTQTASADQQQHQHSSNPEQKDEESGHEMRMEEHHHGDSSRPFFATVTVGVCHCGAGCVLGDIVGEWLVYGTNAYIGNPPRLLWAEMLVDFGFAFLFGLFFQYFSIAPMSGDYSIKTVWRALKADALSLISFEIGLFGWMAAFQLGIFNDELGMPTVTYWWMMQIGMFIGHWTGVPVNWWLIKKGVKEPCA

>CF317_000074-T1 CF317_000074

MLFSLLLVASSLVSAETGIDGWLRYARLPHAASLHQSVPGRIFALNDSESSPVRTAGIELRQGIAAIFGKDCHVSHANWHGAMPTETTAVVGTVSQFESSGGDAQQLPELEEDGFWLHINDSATYILGQNERGALYGAFEYLSMLAQGNMTQVSYASNPDAPIRWINQWDNLQAGGTHGSVERGYGGPSIFFANGSVKEDLSRAAQYARLLASIGVNAVVVNNVNANDLASRSTLPSPLALGDLDTFDPLNASVIEWWHNKTDELYRRIPDFAGYLVKANSEGQPGPLTYNRTLAEGANLFAKAIQPHGGVVMFRAFVYDSTNLNESHWREDRANAAVDFFDGLDGKFEDNVIIQIKYGPIDFQVREPTSPLFAHLKQTNSAIELQISQEYLGQQCHLVYTPPLWKTVLDFDLRVHNQPSLVRNIISGDRFKRPLGGYAGVANVGTNQTWLGSHLAMSNLYAFGRLAWDPATDPESMLQDWTRLTFGLDPLVLQTIVDMSMESWPNYENYTGNLGIQTLTDILYAHYGPNPASQDGNPWGQWTRADADTIGMDRTIDNGTRNAGQYPPEVASMYDNIETTPDNLLLWFHHVPYTQRLKSGTTVIQHFYDAHYNGSARAQTYAPRWQALEGKIDEQRFNEQLFRLVYQAGHSLVWRDAINNFYFNKSQIPDEAGRVENHPYRIEAESMELDGYESYAVSPWNVASNKTCIVTSSNSTQGTASTTLSVDSGKYDLAVNYFDMAIGRSQWELYLNDNLVGQWTGDSDLTLGHAPSNYIDGHSATRITFRGIQIEKGDMLKIVGRPDGIEPAPIDYVSVLPEGVVD

>CF317_000075-T1 CF317_000075

MHYFDPTVDWPKELLEQPMPDLVVIRSTSLIDTKAPWSDADKAWTTDWIQYASTDSNQHFINIIFFDKTGASDRDRQEVVAYLDGLVCAGASYRRYRSDSSGSHISTLPLAPVIECGPGSASSYLVGTADTLPPDWMLRYSLVDHAGSARQAPHQPSLTMIVQPAGDVRAQTVTQQGVYATPPVNEFRNNSNQHEVGNERDSIVLYVVACSASLEADNSNGSTFEWATTSGEIDIS

>CF317_000076-T1 CF317_000076

MVFIYVCPEGRTFTPVDRVNAKVISINLELARKVVGLEELITQEREHKTSLQSANRILEPHITALRMQTEAGRPLTVDGLDLGKVSIAAEAIPTTVTRSENSAELNASTEDDVIDGAEAVKTRALTD

>CF317_000077-T1 CF317_000077

MASSSYSYDEESTHAPFFILTLAGIFAVPITYSIFKKTEDLETTATRIESDYKPKDEDIIQAQRRKQKRKERKTKRILSACVLWGVIVYMIYLIAVTTRTAPQIWDPYDVLGVSRTASEKEIDRFYKKASIKYHPDKARPDPAKNETLETINDRWVEMTKAYKALTDEEIRQNYLMYGNPDGKQSTNVGIAIPQWVIAEGNRWAVIAFYATLLGILLPYLVGKWWYGTQALTKEKVLHSSASKLFKEYKEDITEGGVLTALSAGDEYEYILRGGKADRGAAKIESAVLASGLLDADDVQKLKNIEDPIRRKTLCLLWAYQARIDLGSEDLNRQKYEVAPAALQLNNSFSNITLPFLQTGPLINSYRTSQNLIQALTPTASPALQLPHFTSELTRSISGSNSKSPLTVQKLMSLPASIRRQVCSTLSDEQYSQAMSIASSIPAVKTEHAFFKVIGDRVVTPGSLVQLVVKLRVIPPGTPAHTIPPVDPEDLKDIDPEEGDIDAIKGRKKTITRKRRNSEGKVIGTITEKEENTQPPLAYAPFFAADHAPRWHIFLADSRSGRIAVPPFTFTSFDRPIFTSDGKPTFNVVTLKAQFQAPPQVHAFPFTMHLVCDSYVGFDSSVDVVLDVRDVSEAAKVESDDEISEPDEDSLAGQLHAMKTGELPKQMAKKLMEEESDDESDTEGEEDDTSETDTETEDEES

>CF317_000078-T1 CF317_000078

MPLSQLDFINYPSPKESITARKREEAASNPVLSGLLLALLSRALAASTFVQKLLWRLNGFHKIKDLPELEGYTARFDPTVIPATADSSEPLSLLDLPRPPNRKNEKAYYTTADYHHAYTSGVLTPVDVAEYLLPLISRQGKTPTKYSVAYLDVQPDIVRAAARASAERYKSGKPLSVLDGVPVAVKDEINLKGHKKCLGSSLDFRSKLDTTDWCVRKWEDAGAVVIGKTNMHELGLDTTNNNPTWGTPLNPHNEAYYTGGSSGGSACTAAQGLCPVVLGVDGGGSIRVPSSFCGLYGLKTSQNRVSVFPADPAANTVAVSGPMAPSIDDLALAYRIMAQPCPEDPVSKAWPSTTTLCAVQATGEGKRYLGIDREWIDRSDPVVLNMFYAAVQYYTEAHGYEVVDIRIPLQAENQKAFSLTILAETMTALNMDKVKQLQHPNQILLNASGIHATAQDLLASNRLRQRAMRHLAWLWKQHPGMLVLTPTTPFAGWKIRKPSDVADGHGASDADMTLRSMEYTSFSNWSGTPAITCPLGYAEGSVPVGIMAMGEWGSEEQLIQFGQRHESFLGEDGVRRPTQTGNWLDVLSQAKGQRSS

>CF317_000079-T1 CF317_000079

MSQSFLVAAHDRAVSVMARSSEPVSFETIAGAIIPAAVIAGAYIIAFIAIRSKYRSQYAPRTYSQIIPEKDRTPSTSKSGSKWFQDYRRLDDKFVLRHHSFDAFLFLRFFRMLIIIAAVGCALTWPILFPLNATGGGNGRELDRIAFGNVAKPAHCWGHAVVAVVFLSFIMLIVNRERIFLINMRQAYITAKPNALRLSSRVVLFLNAPQEERVRQLFGNHVKRCWQVYFLDDLKKLVDERSQKVYDLESAEIAAAQNVLAEQRKRKQNGIADGSSLERGDASVDYPSNRPSHRSPPLVGEKLDSINHILKTVPDLNKQIADLREKYKSDKTRPASAIFVEFNSQLHAHQAYQQINHHHPLALQPRFIGIPPSNIKWKNLSMPPAVRISKSYTGIALIIAITIFWAIPVGIVGTISNIKYLADNYSWLSWINKLPPSIIGLIQGYLPPFALSFITSYVPFWFRDIAELSGEPTAQSAENMTQKWYFIFQFLQVFLVTTLSSGAATIIKQVTEEPGNIPSLLAENLPKSSNFYLTYFTLQGLSKAADQVVKYSDTLQYLFFKRIAKTPREKYELEVKMKGLFYGSNYPKFTNMAVIAIVYSCIAPLVLGFALVGFTLFYATYRYLLLYSNNAKLEMKGECHGRAMQQMLAGVYVSELVLIGLFSARGAKGPSILMTIFFILTIVHHVTVNKYLGPLEEYVPLDVLLDAEAEDSDNGGVEANNNANNQNENDDADAEEPLLSSSQSRDTRHATRHQEPNLTESLPVYILDPLKIFLESYNIMPSIESFRPYLLPESESDLNLRDSSLTTVPSYSDSQIANAYQNPAVTSKAPLVWLARDSAGVSEEFKSRNKDRAELETTDEDAELDEKGHLVWDESDVSKAPIFKLPTRF

>CF317_000080-T1 CF317_000080

MLLPILSVALLGLTSTVSALGVPKESMKATGVTKRQASSTFPTAAGYSALSKPMVVTGTFDGKMYRFDRGVSCTGQAEGGDSDAVFEVQEGGTLKNVIIGPDQIEGVHCMGACTIENVWWEAVCEDALTVKQKSGTSHVIGGGAKGAEDKVLQHNGGGTLAVSDFFVSDFGKLYRSCGNCKTQYQRTSSFDGILAQDGSVGAGINSNYGDTATFSNSCFTGVKSICTEYEGNDDGDEPEKISTGASDACIFTNADVSNC

>CF317_000081-T1 CF317_000081

MGSRRGPSRRSDIEEGDWGGTSEVADDLQLLPAVNTRGRRHGTEDYPGHRKWQTRRVLAVHPALTDYGPQTMQIYLENELAKMRDRAMRAVIHIQQDQDLQLRQFARDLVNAQTNIYPAKEDGKRTFEISKMKGQQQRPSSDKGKAVVRDIPQNDGLGMLDVLTASSTSRNVNECVFSPSAVRPSDPHAASDRSHNRIPRSKPLRLGGFDRPKHNANHKSNTASPLRRSPRSKAATKGVADQKLLPNDSLHRDTDMLEPAAIGQGDEADAALYSATMADASSESQVLLARSTSFENHASSSRTSRRPKDTSLNFGSQSHYDSHRRRKTPAIVHVDQNTRDASARLRRSSVLSQQMHPLDVMSSSPTLYLSAGAAINYHANTEPPRADPPASEHPFDSQTSASEYNHPHQAATADKMVTDEGDCYELENGSQMVEDMPAQQLKEQHAIWASIVGDKTRPGHSRPDSFSQCPIHFDYAQIVPEQMPPDIRQAEVNRCKIWEAHGLDDRGKVIAAKSNSGHFGPFTDVQLDPERENFLCCPNDLGDYPRKFLQPASFFNPRQPPFCPSTHPKISLPTEFWEVGVLHYLSSAEVRSLRLVCKILAQELEPYIFRSVVAKFGPSLFSINPSYNADGKLNLTSGDSMLKRHGPTMNKFGLTFDVDLHGLMHAPFKNTEKTVDAWFGRYTWPITDYPYFPALRAIDKLLDDERRLMTQAMSNLGKCSELAISVDSGHGWLEGPDMSDLAIYRRCTSGGDRIFGKAFGAVDRGHEDGMKQLFTWAQMNTINESIKYLDDDLSKVREELDVLRRIIIREYDSYRQEKAQPDYARDIHTGGLVTSSTGAQGQNAVAGNAAHPVAQQINQLTQQMGAMPPAQNANHQVFQQRFASVMNLVRSHLPSSDTQSKTIEQKRERVLPQWPIIFNGYNLSAESRGHRSCVQGRVATPADFPLLPAALTEAQAQWLMETSWIQRTFLSAYTDAVRVNSSVLKNVHSLHVAKLSSGLLPALSQKAFWAALPGLRKVTLLIKPDWRVEHIPGDKYFQTTMSTDPVNAAYKFSELLRDFVVPLENLNSLHVGYVGGGEHQTGMFGRNQHVLPAPVTSNPRDWLVAQNLSQAKPNANTLFTFPHIRTLTFENCWFSPHMLETFMRKSRDNSLHNLVLSSVSLTAQVNSTRVDGPLRTVENDLKCQHTPSAWLNETMPANASWVSTLDKITPGLTILDKKYDAGMINEKVSPRPERQFRGNVRQITLSSCGYVKILGIPSNDFNQNELVFQNSSWSAMDEGLKVRSCKFTKIIPHDVPSRPADDGQQNDSFTAHPRRASACVATSGSSEAAASRPERVQQKESQTTSPTSSRFIMLAGRQGHHVRGDSVSLVEGRSLTALTGFLTQCIHPVEKRILERMWGMEFGWGDDMKRWEAVEDGWFIGGTGRFSGHVFRDPEQQEAVALPLEEQDDQTSSGQSGSLSTHFDTYNAHTPYDLTNAASSAAAHGAGPLGIPAQDTDGDQYVGEDAECIHESEGHEVDDFHDENNAVYGTEYFARTTED

>CF317_000082-T1 CF317_000082

MNKQKHIIVVGGSLAGLMNAIPLKRLGHRVTILERSPVPLLHDQGAGVVAGGETLEWVHRHGRSRRTEDDISVLSQWRCYLDHKGNIIDRQNSHQRMTSWGVLYNIGRECFDGMQYARKGPDANAVGGADNEERKATYEYGRTATAIKDLGDQVQVSFKQARQGETGEEDGTLQGDFLVVADGQSSHLRKLLLGESTSERTYAGYVAFRGTVSESALSNAAEDVFREKFSFFHGSNPNTQILAYTIPDAEGVLERGKRLVNWVWYHNVTDDSPEYKELMTDIEGKRHRFTLPAGGMRPAVWEQQKKTAESRLPPQFAELILKTEKPFVQAITDLEPPRQQKDVGPLMNGKVVLVGDALAGFRPHTAASTSQAAFDALRLEEVFSGKSSWDDYQEMVFAFAKNLQKHGVRLGDRSQFGQHPLAD

>CF317_000083-T1 CF317_000083

MPSPDVQALRTPDSRFADLPGFPYEPKYLQYGNLRMAYIDEHKGKQDGQTETFLCLHGQPTWSYLYRRMIPVFLDYTTSGSEPSRRVIAPDLLGFGRSDKPARDETYTYNFHRDSLLHLIRTLDLSNVTLVVQDWGGLLGLTLPIAELSRFKRLIVMNTSLATGQSAGKGFDDWRDYNNRSPDLNIGDLIGRGTKHLSAAEKAAYNAPYPNQDYKGGVRRFPNLVMTDPNMEGVDISKQSLEMYKTSDHFKANDIFMAIGMKDPVLGPPVMRNMAKMWKNGCFWTEVAEGGHFVQEWGADVAKKAIEAFEKQAAPEGVTRVDPQSSKL

>CF317_000084-T1 CF317_000084

MAQEFKLKNITTLSDLKNCDKQEAEVEGIEGGKVVVVKVQDQVHALNANCTHFGAPLAKGVVSPEGRIVCPWHGACFNVKTGDVEDSPAPDHLHRFDVFEKDGAVYIKGSQENIKAGRRQPKFKVKSSGQEKVVVIGGGSGTFGAVQKLREHGFNGSITVISNEGLPIDRTKLSKALITDPSKVWLRDEQWYKDADIDFVKDTASSVDFKGKSVSTKSGSSYKYTKLILASGGTPNKLPLPGMDLDNIFLLRHINNTQDIMNAVGDKGKKVVIVGSSFIGMEVANALAKENDVTVVGMEEAPMDKIMGSKVGKVFQGLLEKNGAKFQLNASVDSAVPKSDGSNAVGGIKLKSGTVLEADLVILGVGVKPGTEYAQDNSEVQLEKDGSFSVDENFAVKGLKDVYAIGDIATYPYYGPGGNGKPVRIEHWNVAQNMGRSVGQRIAKPNEQQHPFIPIFWSALGSQLRYCGHTPDGYDDVITDGDLSADSPSFVAYYTKGDEVVAVASMMKDPAMSKAAELMRVGKMLSKKDLQGGKDLLQANL

>CF317_000085-T1 CF317_000085

MSSTLVEVEKTSKKRKHEGDSKSAKKKRKQTDEVLETNGVDELGANSSRKERKQKEEQEPEAAGMPQSEVPSTKLNGTSESSSSARKEKKKKKRKEADDPQPEATVEEEALEPDAPATDQGTADALDGTAVQDDPVEAIDTNQLQSDKPSSFHTTRMSLYLPIPAIASSGRMSAMLALHLSPLLLTYFPPASGVILSYQDPVLSARPEAGLGRPLLQPSAVVEPADEQGETLARVGEEAGVSWAWLTVTFLVFRPEMGYELAGWTNAMSEGFIGLVSYNYFQTSIAKNRIPGSWTWSGPSRDRTKQRKTPRKAKLNDSDGPSQESYMDSQETVVAREDDAIDGAAGCFLDAQGNNVSENLTFKVVDLEMIPAQERGQRFALQLEGTLLGAEEEQAVRQEERLKWETKQGKRLSRSRASTPGTPMMSGGLGAFGRGASVVSTP

>CF317_000086-T1 CF317_000086

MDVSPSRPQARDGTAVKMAVTAAAAAAAVSAYLNARYHISQDLTHYYYVRRGLRHHERMRKQRRINIWRTFSENAAKFAHRECIWYAEPSTAPPTIYSYSWREAHQWACRYAAWFLESGVKPGDCVGFYLQNSPDFMFAWMGLLAVGCYPAMINYNLVGGALVHCTKLADCALVLVDPEFQGRVVGNEELRMMGVRLQVVNQTFRSELLGVVPKVPDEKYTEHADEKTRYALRYTSGTTGYPKAVMATTGRIKKFSASRFWDEIRTSRSTAFTYVGEIARYLLALPPSPLDRTHSIRVAYGNGMRPDVWGRFQDRFGVPRICEFYGSTEGGLSHLTLQEGEYLRDAVGHDGLLRRLLLRNEWVPVLVDAETNEIARDSDTGLAIRMPYEIGGELLFRLESEAAFVGYYKNPEATEKKFARDVLCKGDLYYRSGDSLKRTADGHWSFLDRLGDTFRWKGENVSTAEVSECLGRYPGVVDANVYGVQVPNHDGRAGCAALLLDPEVARSGFDFDGLLR

>CF317_000087-T1 CF317_000087

MADSAIVPLLSYLGWSFLPNLGTQLLQNIYYRITISAGAPYPQPGTPIYARDHRRIRVLVLTLYLLYTLAQSLYDVKLAGDFYTLLSVDPHHTTDKEIKSRLRRLAARFHPDKISQSTDGTTNDAPSGMFLQLRLAGETLTNPSHRYAYTHFGPSILTHLPKPEENTTTTVKQLSLQTAQLVTMALKQKIPSYISTLVFVFVLNTFFLPGKQGGKFWRYLIISASFVLECYLLTHDVPALPGALDTVVGWLRGYTKLGDLLPPHLLPFQLLDVSGRLALSLNIFISQISVLFPGASGAVRAAGDGSAQMKGWVQQLNSNLANLAGISNRIDQEAGGLLQLQFAPFKGQPAQVRDLRRGMKEGMVMGSVRSHPEVREVVRKVADRRKAAVRRGNVGSAPTRDGADVVDLLNSEEQFT

>CF317_000088-T1 CF317_000088

MVRSKMGRSTTPIMLVQLLLLLTSLALGAPRPYVQTADTTIALNKRQSSSYWMETITRQGQAAYNSDQSYKIFRNVKDYGAVGDGTTDDSAAINMAISEGNRCGNGDCDSSTITPAIVYFPAGTYKISNPLIMYYYTQMIGDAHSMPRIQGASDWNFTGLALLDADPYIPGAYGAGWYTNQNNFYRQIRNLVIDMTQMPEAGPQGQEVNGIHWQVSQACSMQNVVFDMKATTPTSKQRGIMMENGSGMFMSDIVFNGGNVGAFLGSQQFTTRNFTFNGCNTAIEVPWNFMWTFKSLNIQNCQLGVNITSTNGVNETAGATIIMDSKIQNTAVGIATSYSAVSFPEAGSTFMIHNVDFSGTSVAVQDTKSKNTVLAGNVKIASWGQGNAYMPSGSNQPAKRDPQGVNLGSSMAAAETCSDVVSTTTVFVSPLPATASTTTPIGTISGRPTPSSNSTLPRPNNDTATIAAAQCTSSALIPTKSRVQQQNMNQAAMPSSLLTADGSVFERSKPQYIDVPKEQFISVKSFGATGDGVTDDSDSIQQAMDSVTTDQILYFDHGAYVITKTINVPSNIRIVGEIWPMIMISGAFFSDQNNPQPGFRVGNAGDSGNVEISDMMFETKGPAPGAIMMQWNVQETSQGSCGMWDTHFRVGGSAGTDQQMADCDARTSTEFRPQCAASFMMMHVSQQASAYFENMWFWVADHDLDMIRQYPRPAANETQISIFNGRGVLIESQGPVWLWGTSSEHSALYNYQIANAKNVFMAGIQTETAYYQSAPDSLNSGFTPNAAFSDPDFADCTVGNCKKTWGMRVLESTDVYMFGGGLYSFFDGYSQVCTETESCQTNMVELRCSSNVFLYGLVTKASTNQVVVDGKPAVLESDNPNIFGATVLLYQQQ

>CF317_000089-T1 CF317_000089

MSKRLSSFFGKKDKSEESSPGSATRSRSRHDSLAFTGSPGASPTRPQQRRLVSTSELSQKQRLTSTSELSLNSQSTPSLQPPPTFRETASGISSKPPSRPVSRAGSYEYASASRESSRSRPQTPTPNLLALPGQVLSPPPRSPASPGSAKLSKKKGYFPGAKNDKHPFEDGESQQKAWIAGLKEHIPYDLTALLCGGRVTELWDDNGDVIVHLYPEASGRGPSFRVHSALFRDSRSFGYLCMPAIDSSLQSMHLRGNNEFGNDFGSEFGGPLGDANLGHSRDPSIPTIVPPMDQAKVLYLPLELDGDYSAPETTPHGDDLELVILYRNFFAFLGGGALISTPRQVSLFSIFMGIASILRRLAYSNSDGSTWGEVPDSSFARYCEELRMGDVRTSREKTIEAIVLGENMRYWPLYNEGFVHAAGRLEDVKSIRSPKFAKISPITANRLERASLDVESRLALLHTRLDEFDYPSIFSGIANSQTATESKLVRFKAWRLAFIDMRKFVLSQYKKRYGAWPPKARSKKNNFVEDGLNRLLVKEVYGDMCNLYDMLVNPREMTTRTIDLKPMTEEKGTNETIQHALRSMESEFDRSTPPVIPPIPFDTPLIPSLDQSFKGGNTFASTTGASKLKANEINGLLLGSYNREYIKPSLFVQEFMAYERRLNSGATLDQIVDNRCGQWLFIYVLLQTLPMTAMDARGVNFNEGCEYFLFAAPRGGKPWMREDTLTSKAWYNVTSAGQTISLSADMLDHQPEGVYRRSHCWLMANEWLAAAGMLPEVSLTHSHDSYEGQTPPQAALDEQGISSPQHQTPAQSPLLRPITPSGLQSPLNRSSSYNTLQVNLEQVAAPQKAPRPASQYNPGITFDSILGAAAAQQKPDKKDKKKKK

>CF317_000090-T1 CF317_000090

MSTIKRPIDLQRGWPNPGLLPPARLESAAHTVLSDPDLTWESLKYGPDEGYHPLREAIAAWLTRFYAPQDAISHDRICITGGASQNIACVLQTFTDPVYTRNVWMVAPTYLRCCPIIDDAGFGNRLKGIPEDEEGLDVGYLAKHIETSEQNAIAEGNLEPKLKTMYPWRKIYKHIIYAVPTFANPSGKIMSLERREQLVRIARKYDALIITDDVYDMLQWPGSATANKSVVGKAVLPRVIDIDRYLDGGPISEWGNAMSNASFSKIIAPGCRTGFAEATPKLAWGLSQCGSSRSGGAPSHLVASMIHQMIATGGLDDHITNVLLPAYAKRYRRMMQAIQARLFPLGVTVPQPDKEVAGGYFIWLTLPGGLDGEEIARRAKEEEELIVCPGTLCNVQGDEGNETMRFTHNIRLCFSYEDFDVLDDGVERLARVIKASMQE

>CF317_000091-T1 CF317_000091

MGRSTYAGGSGINLAAPDRPSVVTEHYPWQRTLYDVGIFSDCIYPSIALHGGLSAIAYGIGRMNNRVQTKDYVWAAAPVINAWWGAVGRRVVQRGIPISQAIGVLSRPERLILGGATLWGGRLLYRLVTRAQKRGKDDPRYDEMKSEPGFWNKALFTTYLPESIFQVLITLPMTAPFYHQGAVLSGYHPFLQSVAVGLFFTGFTLETLADRQLEEHKETSADEHSLLKEGVCYLGDSLIHFSFPLLLYASDMLAPIELLGSIANYAFLRYLGGDKATESSQERRYSVSSQQKHSDLQKYRQEKNSFWPNLAQEINNSWLWTVLGAGAAGALIEQAVASFL

>CF317_000092-T1 CF317_000092

MQAPSRQLLRALRAAVDTPPSLTAPSRTSAQRHLCSPRHAPCPSRSLTTTSPLRDELHARARNRYAPKHSNRGPKSTEDTQTDFGAMDVLGHVPAPATSVDACTSDGFHLDNGAKIEGGAGVCLVGGEAFRWVPWRAALSDDAAAAAVPKDATLGSLLDPRRGVLSIPRQSLGLLELVHPKPDLLIVGTGGRLWMLSKEVREYIGTVLGCRLDVMDTANAAAAYNLLAKERGVEGGAGVGALLLPAGWVGVKAPGRR

>CF317_000093-T1 CF317_000093

MNCEGSGPIFKDVASHQEFKYGECGTGAYCLGGCDPINSYSLDACVPMPVCESKSYDFSSLDGVEANTKYLGDASEADWSSSGEPVEYDGTVLLTMAQGTVGTLLASTRYVWYGKIGARLKTSAGAGVVTAFILLSDTKDEIDFEFVGTELLTAQSNFYSLGITNYDNGGNLTISSDSMANYHDYEIDWSPDELTWSIDGTVLRTLKKSDTWNETSNRYSYPQSPARVQLSLWPAGLPTNPEGTIDWAGGEISWDSPYMTNGYYYAQFDSVTIDCYSTPADAQVKGSQSYVLTNKNANESDFAMTNDPTVLGSFLATGLGMDEGKDESSASASADATKTANTVPGMTGAGNGVDNHAGDSTASSSQVQQSATTATDSSGSAVTGFVQGGSSGASNIQSETVLSGSLFAVIVAIVGLCIL

>CF317_000094-T1 CF317_000094

MGHAASKPDPNAKFRVIGAGLSRTATTSFGTALTELLDGPCYHGGTQLLCSDESVIKRWIDVFRKTPIKNEEDKKKVHAEIKSLMDGFVGCTDLPGNAVVEELLEIYPDAVVICTVRDPEKWWQSIRPIVENANFTVLSWILAPVPRFRYFRTYHDALDEGNFGQAHFKEGEKKMPSRVTYERHIEYLKRVVPKDRLFFFDVRDGWEPLCQILGVPVPKDKPFPRLNDAAAVEGIMKSGIKQGMATWAGIGVTAAAGLYTGLRMARIV

>CF317_000095-T1 CF317_000095

MSVSSQHARVPKRRVNSLQNGNKRSYSIGRPRSKSIDGNDAYLYALRVAYLSYLLQPRAKRTRSIQRPQVARAATSTSLHDMMKDFQVVRDSKSEKYPHGFVKALEKRLQNIIKSEDRRPEYQDPHVRRTFAIFLTHYMDPTFHRQIEESKRAEDLVLMFFSKATQELSKGKPPHDDGVKRMVDRHLALFVRLMSSTLKEKGWADERRDLAARLTNLERKLLKHEEDLSSPTAETKGENVEEEVPLTYDVKDMPHVQIVGRIFGFRNTMLQSDIDKHKNEWTEEAALRDLKTYQAHLNMGTRRTLSKVDFDTKAAYEEWKKHEAPDLSQMVLAIMQINPTLAKTSGATSLPQFNPRASMIADGQYAELAKTLSPSGAGQHAEREEMLSPGGGSSYSFEMPDLNGLQIDDRASAGDDEENIYTFIPADSRGTFRYIMNMALTTDLSDQTQDTTNGLTPQILSKQSTELLNELALRWRIPKFSRIVLFLDCIREKFTEQMLTLNQLDAAFNYLKEPVHDDGKNKRSSLVMTSLLYDRFKWPVADFSLMQQTLSGLYDGLLRELYGAMMTCYDNKQSPLLGPILAVIDEHIKSDPNFSYSRDDFNSFKQQVSAGLEDKARDTYAGFVEKEIPGEQDTWEFYHVMQLGKRVLEKAQKIQKRYRRNPEILGIEPLNILLQCMLPAFAEDSRAMIDSVMETSKATGQDVPIEDGFELYKELADFRRIYADALPGKAFPYKLEDVLQEFVWKWINATDAQVMGWVEQAIKHDSFQVRNEDQSRSPSEEERHSNSVLDVFRSFNQVIEQVTKLEWDDDLTYAKFMTALSKSLGNGISHYCEILDQMFHREMDRLTPEQEMAAKQTTQQKYLQYAKDAWNNETRVEPFQFYPESFVKLNDVSFAIQQWDKLESEMNVDACADVIKRHNPPSLQQRRKVTNYVFTIKLVEAEGLKALDVNGFSDPYVVLTDEYQKRLFKTKTIYRNLNPRWDESVDITTQGPLNLIATVWDWDSMKDHDYVGRTSLKLDPNHFGDFLPREYWLNLDTQGHVLVRVSMEGERDDIQFYFGKAFRALQRTQRDMTRNITDKLSAYISHCLSLKTLRQLTNKTISVAKVSSYISSYRNRNSAPTGPTGPTEVDIANALTPLFKYFDDNFFIMNQTLTPGAMIAVMTRLWKEVLMTIESLLVPALSDLPSQQRQLSQPEMDIVFKWLQSLFDFFNAVDEDTGQANGVPTSVLKSPKYHEIQTLNFFYNETTENLVRTSERMASATAQSQQAQRSRLSAPAHLGAPSSGGLLAAGAAPGARRAKSIMLARNLGTMRKAKEEKWKAAQAEPSDDMILRILRMRPEAAGYLRDRSRQKERLAAAAAAEMIVRQSLMSSGGGRMTGPSVIRR

>CF317_000096-T1 CF317_000096

MAISDGASSISVTVRVRPFTIREAAQLTKCDDATIFLGDGSLAAAPTPKLNKKGLRPIIKVVDDKCLVFDPPEDNPVQKFSRSMVPSMGKRVKDQTFGFDRIFDDSAQQADVYEATTKPLLDNVLDGYNATVFAYGATGCGKTHTITGTVQQPGIIFLTMQELFERVGELSTDKVTEISLSYLEIYNETIRDLLTDGTTPRGGLMLREDSNQAVSVSGLSSHHPRNVEEVMDMIMRGNQMRTMSPTEANATSSRSHAVLQINVAQKDKNAGVEEPHTMATLSIIDLAGSERASATKNRGERLLEGANINKSLLALGGCINALCDPRKRNHIPYRNSKLTRLLKFSLGGNCKTVMIVCVSPSSEHFDETQNTLRYANRAKNIQTKVTRNVYNVNRHVKDFLVKIDEQMALINELKAQAKDQENIAFGKFKKQNEKKETAAREGIIRIRNAYEHSAQERQERVTNMVKLRQIGRRISMLSSWIAAFDAVVDMMQEEDPLQNLQAIRKTAQGMLIELESGRHHCQQKVNKNNWERALNSALEFGHTSLKDFDTGDTLDASTLSREVELLKANAEREALLTVAEQDRMGDAVTAQVLLQAHFETIAAIHQMNRMSVDEAIEKATGILTKMLSSATGAVSHVVKPDGSLAPVEAFAPRRAGTPKRRKQPTTAIGANPSRPLDLTTNSQHLASTPVRASPRKRAFIGSAKKGVQLNFTPKKRKSPVKTTKRAVRWKDDTEQGALAEYEKTPQKFTPSPVADETSAELPTVPQVSEMSSFAQRMEEAASSPQSSPIPAAPEPTLNVQPKNDRFKTGFLSRKSEGSPNGSVLPPPPRVTVLSSSDSEQSPLRKLHGNRRSRSPSDRNEESTPANSSDGENQDPSNSQSKSDAMKIRTAVKRSSSVRNPGISKAHRRRSPTAATAASPPGTGDMFGAGHTRRMVMAPPAKSDGWKTSVLSPRADGVTGHHRVVSAGARRTTMGGGSEGGHSRGMSVGERAAMRLSSVNHHGMREKENVSVAPGGVRSSLMPSGKSTWR

>CF317_000097-T1 CF317_000097

MAYLAHSKNRWLNPDHNRLLHVLLKKTFYAQFCAGEDAKEVRRTISQLKVLGYRGVILGHAREAELSKTESDSVDTVQESTEADLQEVKQWRDDTLNTIMLAEKGDYVALKLTGSGKQALQYLKAKMPCFPALRDAVHEACKLAEEKGVSLLFDAEQAVLQDGINNWTLYYMKHYNKNRAVVYNTYQAYAKKTPGELAKHLAIAQKEGYVLGLKLVRGAYMGSDPRELFWDTIEGTHECYDNITKCVVERQYGGLVQPAEGAPKEFSRVELVLASHNMDSVKKAQALRDEQAQRGEPRIRMAYGQLMGMADHLSCELVQQANSRKDIASETVEVPEAFKYLTWGTMGQCMKYLLRRARENQDAVARTADARKALAKEIAIRLRLTKA

>CF317_000098-T1 CF317_000098

MDARQEDINDVLDDVIVKINRITDHLRTAGENEAANQFTRDLTVTFQGLMNIIAPWDLEQCLMNAHNQPKLVPNKVGPPSRDFSATRGNTWASVAATEPVADKHGIIKFQPGDTVKQATWVPRTEENGHDSRDRRVVWVSPWSALRPLSDISNEMREVGAIYSIAFAPEAQSVCIIFQHAYCAVQFVHSCAEYSGRHGISPFGKEQDISPGLPYPINDSLRRMDPPHNERRRLTFARSQLFSNGISETRFRKDIEDIVGPSNVERLWLFNTGNATVVFSAVPLAKMVREAFLKHSRTKRHPYEGVMVSFSHDPCERDLHLVSQIPGHANYIGGNSSANGNDFYRMRTNTVSTDRSSPSSRRMSATSSDYQTAPKRRDTGSTKSAADEEGWQTVKKRR

>CF317_000099-T1 CF317_000099

MAFQMPNFVTKLFRPFTTAMSRPYVQDSLAAANYPPGTERAIFAGGCFWGLEELYRKDWEGKGMLDCRVGYTGGQTEAPDYRSVCSGRSGHAESLLIAFDPQKVTFRQLTEYFFKMHDPTTLNRQGADTGTQYRSAIFYENDEQKKIAEEIKEKVGKEWYKGKPISTEIRAATKWYDAEDYHQDYLKKEPFGYHCPAHYVRPLPPLSS

>CF317_000100-T1 CF317_000100

MAEDLTAQVLSVLNNADGTVLTSEAFPDKDFVTVKSAVDKLRSREFLEAKQVDRIELVPTEEGSTYVEHGSPEARVYGVVKEKGKVVIKELGALIGDANVAKFGQGNAFKNKWIKKDGDSLVPVADSIQDVTGNQLKEVSETKTLADSKAVADLKKRKLITERKVVTFEIKKGPNFSMEFVKEQTDLTAEMLADGSWKSVKLKPYNFKAKGAQTPSGALHPLNKVRQEFRDIFFEMGFEEMPTNRYVETGFWNFDALFVPQQHPARDMQDTFFISDPPKADPPREDPPFNKLADLTLSDPPSQEKSKSDVKESDDYKQYWENTRAVHENGKFGSIGYRYPWSEDESLKLVLRTHTTAISTYMLHKLAKNPRPARYFSIDRVFRNETVDATHLAEFHQVEGVIADWGLTLGGLIGFMEVFFGKMGITDLRFKPAYNPYTEPSMEIFSYHKGLKKWVEIGNSGMFRPEMLEPMGLPKDMRIYGWGLSLERPTMIKYGVSNIRELLGHKVDLNFIETNPAVRLDKD

>CF317_000101-T1 CF317_000101

MENEKGEIVDLYVPRKCSATNRIIKAKDHASVQISVGKVDENGRYTGENQTYALCGFVRSRAEGDDCINRLAQRDGFLKNVWSAQQTR

>CF317_000102-T1 CF317_000102

MAAISTEPKGIHKIPNEILVNILALVPFVPPSPSYPYEPGSLGSHDRLKQVDHRFRDVVDSAALRLQTARRQFPEQAALRGLVTVSATELVDISLLDSNIRLATDKVLKKEARESTQHAIFTSLHLLSYMSSLVDDERHDLTNACLFIWARKDLFDPRWLQVLRHALHHVFDHFFPTIDDFANMVPKDPMNVIKDSQPIYQDGILPMPDLPGMLRRRCFESTILLQNHAGPIYAKIVAGDLSDLAGNIGALHSRISSILVHRLVIAARARPEVADVEFEDDLDFVRSMQWRMLSEEREDEIVDAQTSRLLQVLNPYIYEAVNDSPFHDTFETCNAGETPSISIGEMNRAGSFLRKAYAAIAREGVPDHLKRYAEAGGLFLARHMSDDSDESEDAEDVDSTDGDDGSDGEDEAESDVDEDDDGMDEDGEV

>CF317_000103-T1 CF317_000103

MQLLLASSVLLAVAASIAVPKSDFISYDGYKVFRVKTRGQLESMREKLSSFSYEEWGGKELTHLDVAIPPEELAAFESLGLDSHCMHQDLGTSIKQESAQQTVWKRQINDMSWFDSYHPYADHQQYFEDLHAAFPDNSEMVSTGTSYEGRDMFGIHMWGADGPGKPAVLWHGQVHAREWITSMVLEYLTHQLVTGYSSDNYTQSFVDNYDFYVFPFVNPDGFVYTQTNDRLWRKNRQPPPPPPANQTCIGRDVNRQWPFMWDANPLGASTDPCAQTYKGEEPSDSPENKGMVAFVDHLRDTQGIKLYIDFHSYGQYILSSFGAVCTMYDESLGRHTKMAALTSEAIYEGGNGTQFTFGPICATLYATTGGSNDYVLASGKAEWSYAFELRDKGDYGFVLPPAQIRGTAEEMWAGMQVMLYLLDEEFFDGEGPVLGA

>CF317_000104-T1 CF317_000104

MSTFMQVNRKGIALGAKSGTPALLLALPDHIASVGNDNYGSYDTHRKGNTWPWIEEEQSVMRQFTIKWKLDELYPEDFCHGFRRTDRALTDGHKQILGRHHESYYSFLKEVAAEFDALPLDEQSLEMAGSLLEEKIAENSASVTDDEGPEMNVKKRPRKKIARTVRCTRVVGRVAKSGRYGGKPVVVDSDDDL

>CF317_000105-T1 CF317_000105

MAEANQLQGSNVSQPPPAPPLPPQRPGVLQRFIGPLAFNRPSRAQSAQAAQSTSASPKDASHNRPYRRRPQRAAIYRTGIPIAAIDINESATHAILAGRDILRTVKVEDRSIFEDLNIRGAVSNYISTQPLKPDDIHKRREFLPAKDVRWSHKHYSHVVATAAQNGRVALYDVSRGSSRVELHHLYQHVGQVNKLDFDPHSGYMLLSGSQDKTCKIWDIRDPKKPRGYAQFHARAPVRVVRWSPTDAMEFALCTEDGAVQKWDIRAPQQAVLGIKAHEKSCYTVSWHPDGKHLASGGVDKNLKVWDLKSQKGRQKPVFSVRCPAGIMNLAWRPPCWSAEFAERGTWQSTQIATSYTDDDPRVHVWDLRRPHIPFRELDHYETRPTDLLWADKDRLWSAGGSGVFAQSDVSYAPQPEDSLPPGATSWAADGSFYAVTEDRRVKGRTPATDPAAMFLNIPEGRLSGAEDGMVSRSLTDDEATDTSLSEYASRRQVTAASTRSAKSQANTPPSHEDLPKITPLDRAVMAKKDMFVNGQLSAMTRIPGIHLPHKVLEEIVNGYALPMTEEERQAEPDKILPRLERAFQHNAMVAQTANLHQTAENWRLMGAVIVPELRTWADNNRKKRLDAEKAERHFKADEKDKLAVQNTLPSFTKIWPQNKDIKSPSQSEKVKSNLFRGVVESQGGNSDQASHHGSNMTTPRQQPLLSPAQSTRQAGSTWYTLEDSIDPMQPLPPSLTNAHTTAAKASRALLDNSSDAPDSPKSSPEKPRSSPEISKGHRRSVTESATQHLSSLAARGSPTSTFGEMATERRRSPLPSRTQEERRAALKDYKAPVRQPLNLDPQTTSPRYARGHRHDSVDSFALFPASTSSSARLRDVDQSFDVAESVLDVPAKATQDSDVWVQGDSYYESVGRSEQEYEESPAGSGRGDTQTADFAMDDSPAPLPMGLDGTTDSKPVRASESVEAKFRKVLARSPRSRDEVVAKHPVAQAGAEREGNNPSTSGPQFDSLVEEAVATQSALLGPPMHHFNPLLRGLSRDTESLSRIAEGDVSRKSSYQASDFRPIDITKYEPLVPWALSAYPQICQAIEADVTEGGYGSTSSQFSAHLLCHVHPFFFHQSFRRRLSLQGLTNMPRKVADKLQHPAFASQLIMSILANHIERLTNHGLNAAANTIRKLAVEDFDYPLLAGREERTRKDESAGETLKTDPRKLQEDRIRQQIAAREEDHAIRGAAASSSIARKDPKMAAPSPAADKARDTLKKGGSGDTVTQSSDERTTPSSGGTAWSRSAGGTSFGRRVRVVKPGEDE

>CF317_000106-T1 CF317_000106

MTAWSISLPLELIVLGSVLNIYTQPHHEPTVGDQIGGRLRTDITGWEVFEVIVYFVRVIIIVGLIILYTVLKIVARRYEKPSDSTSEATQPLLGASNTSPQSNGHTNGHAYGTANGSTPHSKEQPDAWAKPTETPIVTWYSYLKGFAVLLPYLWPRKSLKLQALALSCFCLMAAQRVINVFVPIYSGKITDALSGADHHGIRAPWIIITIYILFRWLQGGQGVLGAARSILWVPVEQYSYRAISTAAFEHVHSLSAEFHTGKRTGELISALNKGSSINSFLEMVTFNVGPMVFDLVVAIAYLTYKFDIYLGLVVAIVTFLYIYVTIRLAAWRVTLRRNYTTADREMEAVKNDSLHSWDTVKYFNAEEYEFDRYRKSIKAMQGFEFFVEITLALMNTVQGTLFMVALLSVCFIEAFQVSKGYQSVGNFVTLLIYMAQLQAPLNFFGTFYRAIQNNLINAERMLELFKEQPNVVDKPTAEDLKDCEGDIVFDHVNFSYDKRKSALNNFSFHCPPGTTTALVGESGGGKTTVMRLLFRYYNPEAGQIRIDGRDVNDITIDSVRKHIGVVPQDCNMFNESILYNLRYANQNATDEQIFDACRAASIHERILQFTDGYDTKVGERGVKLSGGERQRVAIARTILKDPKITLLDEATAALDTETEERIQDALRTLAKGRTMLIIAHRLSTIVEADQILVLSGGTVVERGTHADLIEAGGKYASMWRKQSRAQKAAAEADMLRKQAQRTEEAAGIDSTTVSEDEAGGGGKVKRKGHKRVNFSASSSGLGRMPAWSMDAGRGVPEGHP

>CF317_000107-T1 CF317_000107

MAQNDRPLLARDNSRDSIREANEEDALLTGQARPTSRSERSRWQKYREAGLSVYALLATITIIALAIVVNHESKQAQTKTPSHHWGEDGKPTGKRNLIFMVSDGMGPASLSLTRSFRQYTEVLPKEDILVLDEHLVGSSRTRSSNSLITDSAAGATAFSCGMKSYNGAISVLPDHSPCGTVLEAAKRAGYMTGLVVTTRLTDATPACFAAHVNNRAYEDIIAEQMVGNYPLGQTVDLLIGGGRCFFLPNTTEGSCRHDDTDVVKMAQEKFGYSYVDNRKNFDNLRGKGGVKLPLLALMAETDIPYEIDRVHVDHIYPSESEMAQLALDALEAATKDSDKGFFLMIEGSRIDHAGHGNDPAAQVREVLAHDKAFDTVLDFLEDSDTPAVVIGTSDHETGGLTLGRQRPDETYPVYAWYPSVLANASHSASYLGHKWAAYLDSHAGQKASRKQKSKFIKHELFEKDFGIYDAQDEEIDSIIDATIFRPPSWIFADLLSLRARVGWTSHGHTAVDVNIYASNPKHARALIGNHENTEVGKFLADFLDVDVEAVTKELVKKGVRTHGQGEGTGVSKVNGPSLILPDMATPQAVPPSAAAVYAEKSYITHVDDASASSNTSKHEADTANEAVSQYSPKEARKLRRTIDLRLIPALGLMYGVSLMDRKNVSNAYIAGMGDDLSLKISYRYSLITLVFFITYVIFQPPMTYLCRKIGPPIFLPGLCMIWGIVILVFGFAKNWTTLVALRLLLGLLEAGYFPGCVYLLSTWYTRYEVAKRYSVFYLIGSLASALSGILAYGIMQMDGAAGYEGWRWIFIIEGVITCALAVLGWALIVRFPDQERDRPSFKFLKREECLYIIDTIERDRSDVATEPFTMARFFKPAADLEVWGFGLIFFCQTTCSYSFAYFLPIILRTMGFSVAASQCLVAPPYILAAIMMYSTSWVGDRYKMRAAIIVFQSIVAIIGLPMMGYGGSTTVRYLGAMIAVAGITSNIPATMAYQANNIRGQWKRAFCSATLVGMGGIGGIAGSLIFRSQDAPEYKWGFVGTIVSVVVTIVTCAVLSLVFWRRNKKADKGELLIEGLQGFRYTI

>CF317_000109-T1 CF317_000109

MDRISGWQELKTDRKKQCPKLFYNYVADEHGYTLQFTDLIALWTATATADDVRNRARENRTSIDASQSSQLLVLLRKLKESLAQGSNVLKKDLPSNSETVLLETTLNLPRPLEPLKWQFVLERQGSGEMAEHILRPCLYEASESMKKIDTLISTIEAKDRVILRLVQKIEASSMDLSLIFPGITGPRARKGQVTVQDAERHVPGLRKFHKREWQDFFKSDSDYANFETAGLFTLAVKKCPKHTPQQHEKWLDKLPKATATTSQVVQAAWYRSSSPVRPSDSESDEFETQKKRPTEIVQRRDGHMSSDTESDDGGLADRDDPSPRGQRLGLDGSGTRSPIGSSPPPLPPLPSKHSRSGSGSSPPTEVPLSQYTRNKPKTSFGKLGAKPRATRAGSPRTIQPSSSSPIPSIPITKSQLESSSPAPNPPLESDKPPHFPSSVATASNPSTPRKLGRLRGSQSVAEAITPKTTKADTGDLPTTQTTPIHNRLGRLRRGSSQVVSQSQHPTQKTGADLDIEMQDVTANESDASTASTASTSTPPARKKSQSKHSSPPQPQDGIDEVTLTSQSQQLATQKQEEQEPEEELTKEEKAKRRREELKRKIGDTNATTTGTGPAGAAGLRKKRKF

>CF317_000110-T1 CF317_000110

MIEGHHPHAIDLNELDMTIFPIMDKEMDRKVGLTVTRHESTNYKRPNDIESSWRKKASLSYDRRERLPRYVIIDPIEDLINDDTREEELDFVDPETVLEGLSASQLNEVRKAIDEYLDIEYKSTTKDYWRTLRTICRDRQRGVEGRAVRLVSADIDNLLRPKSYEELEKLEKQVNRKLDSDEPIDTDYWQQLLDNLLVYKAKAKLKKLYAHVLDNKLAELRRNNAEAAQQAVGELPSTSAPLIRHSKAVDPDALLAVDPMDKSVPILDEQQFLDDITLQRRKVVKAGYMPASTKALATHAGPVLSYGRPADSDRTASSMFDREAAKGVEEDEEVFAGEEHVETKSAELWRGKFRPRKPRYFNRVQMGYEWNKYNQTHYDHDNPPPKVVQGYKFHVFYPDLIDPTRAPTYKITREGGRKKGETVAPAGEEDTCIIRFIAGPPYEDIAFRIVDRDWDYSAKHDRGFKSTFEHGILTLHFSFKKVYYRK

>CF317_000111-T1 CF317_000111

MTQERPNPFNDSDSVEDEDHQTYLAPRTSVSNEHAMLACSHLSNHDQYSLPLLPGNSFNPSFVPGSNYTSLSYSGAGIPTSYQQLAYHPQHGFSNIHQNMAYLPSNRPSVQLNPFSSTMEPQMMLRHSEFAPVAQPYDFNTSFAISTSACSPQTMSATTVSPPLSFSCGNDEYYPEIAWQHNLPTFQMPDGEGSTASVSDGFEEEEGGVYDKPYAQLIYDALKEAPGHRMLLRDIYDWFLHNTRKPQESGTNGWQNSIRHNLSMNQAFENDKNDPASSRGARKANSVWVLTEHAIKHGVQSTTRYRKTGTAKKGVSNRAPAIQRQRSGAKGGRAARRAARLKRQGEDQRRAKTILPESETPISLGYSRSPLSIAQDQWSSYSCSPTTPAEDQFLNTTYSLPHRSYYDYDQELGSKQEAETIPYEDEQLRQMLSQPMSDDAFEDAAQA

>CF317_000112-T1 CF317_000112

MVNQRLQYRRRNPYNTRSNKVRIIKTPGGELRYLHTKKRGTAPKCGDCGTKLPGIPALRPRQYATTSKTQKSVSRAYGGSRCASCVRDRIVRAFLIEEQKIVKKVLKETQEKKGGKK

>CF317_000113-T1 CF317_000113

MASIPPPPPPGWSSGPPPPPGMSSAAAPPPPPPPGYRPAHDPQKAKFDQRKKEWLRTQKNRFGEKRKGGFVETQKADMPPEHLRKIVKDIGDVSQKKYSSDKRSYLGALKYMPHAVLKLLENMPMPWEAAREVKVLYHVNGCLTLVNESPRVIEPVFHAQWAAMWVAMRREKSDRRHFKRMRFPPFDDEEPPLSWSENIEDVEPSEPIQLDLDEEEDSAVYEWFYDHRPLLDTSHVNGPSYRKWNMTLPQMATLYRLSHQLLSDVVDKNYFHMFDRESFFTAKALNVAIPGGPRFEPLYKDIDPNDEDFGEFNAIDRIIFRAPIRTEYRVAFPYLYNSLPRSVKLSWYSHPQAVYVKSEDPNLPAFYFDPVINPISSRSVAPKNITISHEDEIFGVGNNEDDDFEMPMGAEPWLADEELYTNETSSAIALWWAPFPFNKRSGKMVRAQDVPLVKQWYLEHAPSGQPVKVRVSYQKLLKSYVLNELHKKKPKAQNKQDLLKTLKSTKFFQQTSIDWVEAGLQVCRQGFNMLNLLIHRKNLTYLHLDYNFNLKPVKTLTTKERKKSRFGNAFHLMREILRLTKLIVDAQVQYRLGNIDAFQLADGILYAFNHVGQLTGMYRYKYKLMHQIRSCKDLKHLIYYRFNSGPVGKGPGCGFWGPAWRVWLFFLRGIIPLLERWLGNLLSRQFEGRHSKGVAKTVTKQRVESHFDLELRASVMADLLDMMPEGIKQNKVNTILQHLSEAWRCWKSNIPWKVPGLPAPIENIILRYVKSKADWWISVAHYNRERIRRGATVDKTVAKKNLGRLTRLWLKSEQERQHNYMKDGPYVSSEEAVAIYTTTVHWLESRKFSPIPFPSVSYKHDTKILILALERLREAYSVKGRLNQSQREELALIEQAYDSPGTTLARIKRFLLTQRAFKEVGIDMNDNYSSINPVYDIEPIEKITDAYLDQYLWYQADTRNLFPSWIKPSDSEVPPLLVYKWAQGINNLTDVWQSENGECNVMMETQLSKVYEKIDLTLLNRLLRLIMDHNLADYITSKNNVQLNYKDMNHINSYGMIRGLQFSGFVFQYYGLVIDLLLLGLQRASELAGPPSSPNDFLQFKDRETETRHPIRLYTRYIDKIWVFFRFSQEESKDLIQRFLTEQPDPNFENVIGYKNKKCWPRDSRMRLMRHDVHLGRAVFWDLKNRLPRSITTIDWEDTFASVYSRDNPNLLFSMCGFEVRILPKIRNLSDEFPVKDSVWALANNETKERTAYAFLQVTEEDIAKFNNRIRQILMSSGSTTFTKIANKWNTTLIALFTYYREAAVSTVNLLDTIVKCETKIQTRVKIGLNSKMPSRFPPAVFYTPKELGGLGMISGSHILIPTSDKRWSKQTDTGVTHYRAGMSHDEETLIPNIFRYIIPWEAEFIDSQRVWTEYSQKRLEANQQNRRLTLEDLEDSWDRGLPRINTLFQKDRSTLSFDKGFRARTEFKQYQLMKSNPFWWTSQRHDGKLWNLNAYRTDVIQALGGVETILEHTLFKATAFPSWEGLFWERASGFEESMKFKKLTNAQRSGLNQIPNRRFTLWWSPTINRANVYVGFQVQLDLTGIFLHGKIPTLKISLIQIFRAHLWQKIHESVVMDLCQVMDQELEQLGIETVQKETIHPRKSYKMNSSCADILLFASHKWNVTRPSLLFDTKDQIESTTTNKFWLDVQLRYGDYDSHDIERYVRAKYLDYTTDSMSIYPSATGLMVGVDLAYNLYSAYGQYFPGLKALVQQAMAKIMKANPALYVLRERIRKGLQLYASESNQEFLNSQNYSELFSNQTQFFIDDTNVYRVTIHKTFEGNLTTKPINGAIFIFNPRTGQLFLKIIHTSVWAGQKRLGQLAKWKTAEEVAALIRSLPVEEQPKQLIVTRKGLLDPLEVHLLDFPNISIRASELQLPFQAAMKVEKLADMILQAKEPQMVLFNLYDDWLKSISSYTAFSRLVLILRALHVNTDKTKLLLRPDKGVITQPHHIWPSLSDEDWIKVEVQLRDLILNDYGKKNNVNTQSLTSSEVRDIILGMEISAPSLQRQQAAEIEKQQQEQQQLTAVTTKTQNVRGEDIIVTTTSQYEQQAFASKTEWRTRAIATSNLRTRANNIYISSDDVHESEDSYTYILPKNILKKFITIADLRVQIAGYLYGSSPPDNSAVKEIHTIVIVPQVGSTRDVQLPQQLPQHEYLKNMEPLGIIHTASGNETPYMTAQDVTQHAKLMQAHPTWDKKTVTLAVSFTPGSVSLSSWNLTPAGYEWGAQNRDTQSDNPSGFSTSFGEKSQLLLSDKIRGYFLVPENDVWNYSFMGASFSGRVEKGGVYVKVDQPKRFYEGLHRPVHFASFNELEDTWADREDVFA

>CF317_000114-T1 CF317_000114

MLPVKLAYAQAAAAPPPPPPAPPKPKFFAFLKLPQELRDRIYHYYYTPLSITVSQQRRQLYYPRRRGGPPELKISERPSRALLLICEEITQSAAPIRARAPVDLAVGYDQAKGLSTLQAICSRGAQYNALRKQVKDLIIRGFIGSSSLGRLGHAYNLITLHFPNLQHVSLDYSAMRQVYEDYEGTFETYEWKAPDFNAFHAGNNDRELRYPDRLMGVKDLAAVLENAGKEDVKVDLEMSVSWYAKGRGYYLSQACSSLTPLRRRACVHA

>CF317_000115-T1 CF317_000115

MVESVDEPPLRGLHSWAKKFRSPRALSEKDTPPSSTSQNILPQHADGTDQVHGRTQPAERRPGNETDTQQQGAHAVHNAGNGTEATAIADGQDKSPKAPIHQRFVRDAKRIITASWINWLLITVPVAIAIGIAHDWAHVGGISPSVVFAVNAVAIIPLASLLAYATESVANKLGDSWGALLNVTFGNAVEIIIFVLALVAGEVRIVQAAAVGSILSNLLLILGMAFVLGGLRFREQLFNRSVSQLSACLLSLSVISLLLPTAFHATFEDQDLANTVVLKVSRSTSVVLLLVYILYLLFQLKSHAFMYESTPQHVIDEESHPGVLAQMLDSSSSSSDDSSSTDSDSTAGSHKTANRIKRALRRRRRKSSSSTKEGNLGSFSRSETNTGVLSGSTRNNDNAVCSGDEADSERRPRGRIADPEVQTRDFEQQQDREKPQKRSKKSKKSKKSKAIEPQPEKIEMKEHFAGRTPVPSIQVGFAEDVCQIPDNTSGRPYNPRNLSSAIKPVLNSTNFPHHSQSVRTLAPPGLRAIDRASSGSRPRASSMPDFVREGSHPRAASPRPTSTHSNPYPNQQVIETVDPEIHLSITSAICLLLISTALVALCAEFLVGSIDYLVQNSGVSQAFIGLIILPMVGNAAEHVTAVTVAYRNKMDLAIGIALGSSIQIALFILPITVVLGWAISTEMSLYFSLFETMSLFASAFIVNYLMIDGRSNWLEGVLLLAAYVIIAISAFYIPTCGLSDANGIATSGAC

>CF317_000116-T1 CF317_000116

MAINKDLPLTSLQGVSLTTVLFLLIWVCLKRHVDKRGKFVWAETVLETHDFLVATVSLVLAAYVLDIGHDVLVASTGYSFDPYTLGYAYHLLKIYEYLDIIIAILSGNTLISKNTAFAHLALPYWSYFRILNRPHDSLNWRLQVIADCFVRFLSRAVPWLMEDVKMEETILQMFGEGRWYADLAVTGIWLLFTVTDQREDEQAVKIFGKPYEDESTAYFLSAIIMFYAGYTYRQVEVEKTEQENAKGQTKQLQVEAEKGVGTEPTSRSTQKAIRQSSRRKP

>CF317_000117-T1 CF317_000117

MSQTKSLKDLKRPDLFRQKGCIAEEWVDAASGKTFDVYDPATLDKLATIPEMGRRDTDQAIQAAHDAFQSYKKTTARQRARWLRKWSDLCHEHIDDLALILCLENGKTLTEAKGEVIYSASFLEWFSGEAERIHGEVVPTSNLNQRILTFKQPLGVAACLAPWNFPIAMITRKVGAALAAGCTTVWKPAGETPLSALAQAVLAQEAGFPKGSINVVTTLNTVAEVGQALCESKLVRKLSFTGSTRVGQILASQCSHSLKKLSLELGGNSPFIVFDDAKLETAIEAAIAGKFRNSGQTCVTANRIFVQEGIYDKFSEALSKKIKTLKVGLGTEEGVVIGPLTHERAVEKAMNHINDAKKHGAEVILGGSSYKGEHKGYFLEPTIISGMSKEMITTREETFAPVVALYKFKTEEEVIDLANDCEVGLGSFVITESMPRMWRVAEALEVGMVGINVGVLSASESPFGGVKGSGYGREGGRQGIEEYLTVKSMIINVTN

>CF317_000118-T1 CF317_000118

MSSISDLNSPVDRHKFYFQFVTTPTADTPGTTLLLHFDNKRYLFGRVAEGTQRACIERGVSLKKARNIFLTGETKWETNSGLLGMILTMADVASESNEEDKKALGDMVIHGGPKIWHSIACARRFIFRTGMPLRVFEADPSAWQASQQPDYIDENILVWALPLQRDTRRATAVAPPNGRSASPSTIQHEQSLRQKTVHDMFDSDWRKDRLTEAVFHDVKLPAVVWKRDPATKDLRATFCTKLEDAPHIHPDEKVLVRMPWPAALVTALPPADNLSSSVAMSYFVKGRPQRGVFLVSKAKALDVKPASNFSKLAAGHSVTLEDGRVIKPEDVLDTPIPGYGVAVLDIPHVHYLPDLLQKLIDMQTAKALVDVHAFIWILGPGVLHSHQFSDFRASFPSVKHVISSVDDSPNYLAMDSFAASAARLAEIRPETFSVPKHNNSAGGEEFYSGDLIQAQRALSLQVAPRFELSTKDVPSYVNLYQRVAEMSQESRSLISALPRSAPSDEKYKDVSITTLGTGSALPSKYRNVSANLLHIPSLGYFILDAGENTIGQLRRLYQPDELEDILCNLHMIWISHLHADHHLGTLSLMVAHREATRKRAEAGRPVSHKLYLVSETNMTDYLEDYKSIEATDAVMLRVLQSKVTDVNHQPVNLKDTTLPISKLDTVRVSHCQRAQAISVTFSNGFKLSYSGDCRPSAAFAKIGKDSDVLIHEATFDDGMEGDAIAKKHSTIGEALGVAHAMKAKNVILTHFSQRYQKLPTLTDVRLPDQTKFEEGDDPADSSGPVEDPALTENAVLTGDDLKQLDQAASSKARAQASSAPPALKTQTSLQEAAEQMSICIAFDLMRVTVPQIKDMYKFYPAIELMFNHEQAKSDDRRGQNRAANEASVNARKEKTQSKIRAGEQKKKDKKPGSKSDKKNSTNQKQNKPASEMRQANLSSPAGANGTPHATKRSRSPKALQKESETGDKKPKLDADRT

>CF317_000119-T1 CF317_000119

MDVNQSEYLSVLSRELANENADASIRQAAGLALKNAFSYRDVARLREVQARWLQTVDPSIKKQVKELALQTFNSPSMASSSAAQLVATIAAIELPRNEWPELMPLLVNNVGSGPERTKMSSLTTIGFICESEDADLREALVMHSNAILTAVVQGARKEETNQDVRNAALAALSDATEFIRSNFENEGERNYIMQVVCEATQSEDPRVQAGAFGCLNRIMGIYYDKMRFYMEKALFGLTIAGMKSEEEDVAKLAIEFWCTVCEEEISIEDDNTQAQQEGSNELRPYFNFARVAAREVIPHVLELMSKVEEDDADDEYNVARAAYQCLQLYAQTIGSELVPMVLQFVEANLRAEDWRKRDAAVASFGAIMDGPETKVLDPLVKQALPVLIGMMQDPSLQVRDSAAYALSRICDYCYESIDTSAHLQPLMQALFQGLMSNPKMASSCCLALLNLAERLVSEDGSDTNPLTPHFKDSVTALLQVTEKADSKEDGANQVRTAAYEVLGGFITNAANQSLGMVNDLTGVIIERLSQSLSQAKDVVSIEDKLNLEEKQISLSSVLLTIVQRLEQHIAGQSDHIMQICIETLNATGNTAVPEVIFQIVSGLCNALNGDFLKYMESFAPYLNAALRNQEAPDMCSLGIGLVSDIVRALEDKAQPFCDQFMNDLLNNLRSDKITNQLKPPILETFGDIASNIGPAFETYLTVVAQVLTQASQVTVANDVSYDMIDYIVSLRSGIADAWDGIIVAFKGTQRVNLLQGYAQPIFAFLQIVAADVNHNEGLLRACMGIVGDLGEAFPDGQLADFFRADWLTKLIKETRSTREFSNRTITAARWARETVKRQSAHQGNLMNSS

>CF317_000120-T1 CF317_000120

MQPISINLLPLPIGTQQPGAQNVPASEFGLKYCPTMDLVAVFPQVMTVESAADTATSEKTEHDDYYGEDDEEVLVEVYRLNGQKVFTISIEKEDGAVGVVDVAWRDDGVILAIVTSDNTTRLVNSFSGKIVHSFSSASSQPLTSASSINPLKSPNSKRKSTDRDTGSNKRKCIPTSIVYSTHFTEPKNASHQLETAKLERGTDLDDLLSLNADLDQLLRLKADLPRELANLDVEQYLPKLATLPSNGMGEDDVFSTRTSIDTMFHPTKQNAGSISTDVVTVSQSDACLHMRVFDSFEVGSVDLHRALDAPAGYNIGKIHRIATHPFSEKIYVVTEERQGSSTRRQTRSQDTAEDAALQLHLLSLDLRFIRQSAHTLPMLATKATQLHNLIRYLRQIESQLAREVKTAFDLPARFIRTLEEDLKEQDGEGSTFETSAYHALLTGEVHGKFKEWLVDILGDRGVKRWDKAVHECLELVRRLISENWNPAVERAGIVVSRLTGLAAASSTFDIEKEVLDRLQDTIDTMAVVGEDLLRDTNAEISGFNAFIIWLKREVEMAELEDTSEKLDEMREGSDHSEVRKVLRYISERLRDTSVKKYIKDGQAQVTEDEGELRAYEKFKKDRQTGTDDSSIPSMKSLTAKLTGQCEHLFKQVATMLRESVVAQYMCKLTDDVDTEIMACRISYQPEYAVLHVLGRHSTSKGRLCWLRKSIDSVPLRSNVSTKQVDLDNAEEVLDIKFVDDREAVVLARTTSGTRVISFSLQDEDQQAVRHTFGGRDDPYTKAGLKPWKLEINGRKLRRTMTVLDEQGRGYGVFDLDSAESGGDGEDEAMSG

>CF317_000121-T1 CF317_000121

MSPTTTLITFRVRAPRSTRSLLLYGSWDNFSIGYSMIKDLQLGSEYWSGCFNFTNIICDGRPSDVMRSRDGGLKMGGTYWYHYKVDDETDFHNVCERATTNCPMLPGQLVNVLNVPVALSGNRSRDPSTSSTSSERRTMNPEDKFMNPRPAPAKPESLRLNTSPTLSDFPGNNDASRPSSPSQETPNTSRFLRLPRKRSVDGHTSPSSGTALTGGLRAAFRLRTARSQSPESQMNNGTLNDRRAVSAERQASSPNGDTHSERLQRPQRGLLLRAKSDETVSTLSFIKHRQQRSTSKKHVYPCETVPIETSRHIGALNLSQSNHEASSGVKDVTSIPDAHVDRLAEDVPVRIELDLEKRLPTLPNTPSSAYPMSIVGDSLPCHQPLDMDQLNSHFSATTVDTQMHPTSRALNERSHFSAWTTTSDTSSIFVDTSEPVPSLDRNGYLGGGRPRLDHAVEELFLPASFSYSSMTSSTSTTPSSVCGNMDTESADHEESAPSARFSHVAALPNQTQHYSLPDYGYQSQNTLKSPSRKSPEGFSGSATISDIEHQTTINGHGNEIIHSESMQRLLDELSYLGGMIRQ

>CF317_000122-T1 CF317_000122

MNPNNRGIPTASSPSKHNAQPTYPSNNEAPPAYSAGPPVMAPQPRSALETIADSPYAFLGQFDTVFLIDDSGSMAGRSWKETASALSAITPICTEQDADGIDVYFLNHRNPYAADNIGAYRNVTSTGAVQEIFRTVRPSGGTPTGTRLYAILKQYLVDLPKELEKQARGQQSTVKPLNIIVITDGVATDDVESAIVSSAKKLDKMGAEPWQVGIQFFQVGQDRAATQDLKELDDSLAEEHGIRDMVDTVPWSGVDGAVLTAEGIMKVTMGAINRRLDRKRASQEALHGR

>CF317_000123-T1 CF317_000123

MTTDSPMFPSQFDDAKPASNSPERATEVNPRTSTGNMSSLKEPLLRRTTFQYSASAVSAHAVNPIDHALLPSLLQPRRLLLRNYFEPVRAAPGTAFRSRRNKIAVERNLPYLADDGSPNAATTQSLVYSNHAGTSTHDVRGFGDPQSVSCVDLLAHPGVGQQSLSFASIWKGADGADPRNESSSTSLDTECIACNTSPPLIFSGTSDSDSIIKMLSDAIRSVVDREARLVSDAVSCAEQTPRAQQVAILPQHAATGVTKCPGPPPLQPLPPLPRPQNVLERIELAKRYDAYVRSSMPDHTDDQIGTRPFHEPVWEGLD

>CF317_000124-T1 CF317_000124

MLAHQKCERRSAKAQVQAKAVEVDVNTDGSELFSHDDSRFMMSSDDEDPEAASNHTSTQSSHLQGQLDEQDFPQPSECNLPKSPNVDFRRLAVVHGPGAIFIKTPATPDCDGDTDRESTDSDVEDAQPVTDDEYPSQETYDHAVKRGGREWRRKREEEKAVARRTFKVPSDWNDAAKVNEFLVESKRIWQ

>CF317_000125-T1 CF317_000125

MGLKMSDADPNLSTIHVPSGPTTLSSSSATDLSSLSLPELITRRDNLEAELKALGAVLDSHGVTMQTTLTTFDGYPRADIDVAQVRVTRARIIRLRNDWKDVMAAVERGLHAWHAANKNVPTQTPTQTQTPTSQEPTVTQPASADPPAAPEAPFARVNTVEAGSPANEAGLKVGDLIRRFGGAIWSNHERLRMVGEVVQQNQGRPILVRVQRKLDGQDVRELELRVTPRVGWGGRGSLGCHILPL

>CF317_000126-T1 CF317_000126

MMKSVLSAAGLVAVASAFTQPTSQTFGSLVTPDLSSPVTTGQEYTITWTPNYVAAQTISLVLCNGPGSNCVLQSSAIVEGVSAALGSYSWNVPCSLSAGTQQTDSGYGMLIIVDGTGEFQYSTQFSVEQGASCSDVTSSATSAAPSSSSVVPSSSTSSWESSAMSSDMDSYMSSSMTTSVSAVPTSYTTSSGWYGWSSTFNTTATPAYTATAMNSSAPAFVTSAVATSAVATSKPVEASSSASSSAYVHATMASGTTAAVATDAVATSAMPKAYTGAAAMPTAFANVIGVVGVAGLAMFAL

>CF317_000127-T1 CF317_000127

MAEAAQAPPTFKLVLVGDGGTGKTTFVKRHLSGEFEKKYIATQGVEVHPLGFTTNLGQIQFDVWDTAGQEKFGGLRDGYYINGQCAIIMFDVTSRITYKNVPNWHRDVVRVCENIPIVLCGNKVDVKERKVKAKTITFHRKKNLQYYDISAKSNYNFEKPFLWLARKLVGNSTLEFVAAPALAPPEVTVDQATLDAYQKEMTEAAAMPLPDEDDQDL

>CF317_000128-T1 CF317_000128

MAASLEMIAEKKAILVALLTSEMQTLLLHVGTRRITSHELDLIHSLLRSMSPSYDLKDAIFYLETYDWNLTTTRLQYTFDDIDRQGPYLDEDGNSPALAQYNHSLDRAPATAPVPQEEDIKSNKHNEFDCSKFEITVNLNSDKPKTVRQATYTYPGINSFDWHDQSCLDNLNIWRRNVFREHIGPHENGHIAAATLTPQTQYQNGIRIPGRGTGRLLPEYATFAKEMAPIFRGRWLPGRVILKAVERGSHSITSFLHRRFIAPRSRGNEKTRWTQEMKHKAGQREALRSRQGQWNLAATLPISALGPVWPPCEPSGDGMVIVQMSDLEMEGAEQWVGADEDRPAGEAVEPGDVEDQEYFESRKRRRIREVLASDTMH

>CF317_000129-T1 CF317_000129

MSASLPGNRDLPASQYDLSTYWGRVRESADIADPRTLFVSSSGLENAKRAVTAYKKGEVKTMTPDIWQAKKIIDSTLHPDTGQPVFLPFRMSSFIFSNLVVTAGMLTPGMGTTGTLLWQITNQSLNVAINSANANKSTALSQKQLIQNYFVAVSASCTVALGLKAIVPRLKRVSPNARVILGRLVPFAAVASAGVLNVFLMRGEEIRRGIDIFPMQTDEEKAERERSGKEVQSLGKSKKAAFLAVGETAISRVLNATPIMVLPPMILVRLEKTEWLRQRPRMVLPINLGLIFTTSIFALPLALGAFPQRQAVSASSLEEEFHQRGGLNGLVEFNRGI

>CF317_000130-T1 CF317_000130

MSLTEACCTCATILADTKVPYDTENEKPICLDRRLECCGRTICATCQYKNDRFRNYCPFCQQSTGPSALPSEGLRLPPSYKDDEKRRNDLPPAYDTLTLRTQTRSSQTAIRPPEHTEDTVHFLSPEDTLHSLSLAYRVPQDVLRRHNNLFSDSLLLARKFVLIPRSYYDGPPLSSPPDPEEEERKNKVRKWMMRTKCAEYSMALLYLKGSQYDLDIAVDAFKADEQWEKEHPMDSKGKHKDRPSRRFGSSLAGQLL

>CF317_000131-T1 CF317_000131

MGFLKTTVLVGSGIYLGKEYMKGKQLAREPQVNGALGYGGNNNSGWNHSAAAQQQQPVPVNSASQWQSCTKS

>CF317_000132-T1 CF317_000132

MAPSSNIIVFGGASLGDTSQNFPELQTPEDIQKLLDLLKSKGCTTIDTAQLYGMGKSEETIGQAKAIEQGFTVDTKWIGGWLGKSWATRETMVSSAKESLEKLGAGKGKQVDVFYIHSPDLHTSFEDTLKGVDEAYQAGGFKRFGLSNFTPSQVKEVLEICKKNSYIMPSVYQGSYAAVARKAEDELFPLLRENNFSFYAYSPIAGGFLTKNRKFVEEQQGRFNKDAINGIYHKMYNKENFMSLLDDWEKIANEEGVSKAELAYRWVNYHSALDPEKGDGVIFGASRFQQAESTLQYLKNGPLKDSSAKQIDALWDRVKDDSILDNFQAVFGGSA

>CF317_000133-T1 CF317_000133

MRCSNSAVLIAGSLTWLTTAFQPATRLPDPRSLQHPIADEIRRPRYGNHEISQALQDVLDGLETMQETYFDTFAGTWADAIDWTAAVLGTHVTTSLSGIVEGLDLKFEEACTDILRWENLINQYYTQTAFFYFGENAFALRNQAFDDMLWVVLGWLESTKFADMYARRHAGGTEYKSAAWHGLQLSPMAAHRARVFYELAAVGWDDSLCGGGMTWNPSLTPYKNAITNELFIAASISMYLYFPGDNDSAPFVKGQDRFYNAHDPIYLENAVKSYKWLKDSQMRNSIGLYQDGFHITGWQRFRNGTINPGTRHCDELNTMVYTYNQGVLLTANRGLWIATGARSYLDDGHNLVSSVIRATGWPNEDKQWYGLGRGGVLEEYCDHGLHCSQDGQTFKGIFFTHLTEFCRPLRPQERESASQITPTPFDEEGYRYHLARCAAYAKWIEHNADAALATRNEDGLFGMWWSVPYKSRDRAEWERFSNGQLLPEGAVDHLNPVSYFRSAVEDGDLNDRGRGRTVETQGSALAVLRAKWMWQALYS

>CF317_000134-T1 CF317_000134

MSTTYFTKLPAYRNARFAEHWPEENEQWRDRKRNSTAGPVPTNGPSKPKFPLLMFSHGLGGTKTAYSSICGEFASHGFVVCAIEHRDGSGPRSVVNYNPTDSLRRVESEKAAEAKHKRRPKAERSYDTVDFILSDKDKYDTAPQHHLDQELREAQILLRVAEVDEAYYLMTEIQAGRGNELKKQNLRRTGRAGASSLGLEGVDFRAWIDRIHMDNVSMVGHSFGSATTVEMLRSSSQYSYIKRGIIYDIWGIPVRPCTPDHRIDVPVLGINSEAFMYWDANFDIAKGVCEEAREVGQPAWLMTVRGTVHVAQSDFCVLYPRVASGLLKMTMHPVRAIDVNIDASLDFLNRTLHFDGEDDKHQAFRRNLPKETFLDLNPVEQMPTEHKPDPKFTAMRLKLQHEGRQRLKPHAREKYWQRLRERGEEEVWVHMAPGKEVVSKHGDDNERAASVEADADNEKV

>CF317_000135-T1 CF317_000135

MPRLVNNDAQLQSLPVELFDYILIELDNAELKALRQASRFLSSLSTPRLFERFTLYPHIRSFERLLSISESESLRCCVQYLEYETGYLGLTDRFIRRLQTVWSSQISPEQKKKAIEHAHVVTSQKIRADVPLDNMAQLDYLERAFPNLMNLRAIVVQDSCEHLDEGFTREEMPHFYAQLAEETCGLYPHTRLEHGTLGVRHSSYATYAHAVMIAASKLPASSLEYLEINGFNWQHFLHIGTFSKFHNLFQRNMAGLKSLTLYAQRHGFCLGVQAVANLQTLLRAAENLEELRFSGRWCDDIRLYGPDLIDEDVGTTYRSIFQPRLSEQDLPPLPAQLIWSPKLRHLELCGITISPKEFKHVLKPCCDTLESISLANVVLVPEDFSKHPPKEVPRACWVNMLKWMQRHLKKLQQVNISARLTNGGMQHWRVASHPVVNGENCLRKRVCEFLLKGGPCPLEHVAIKPGDYDLKKKTWTGSVPEFIDNHPSEYDGDSSWRMDYNDEDDPDMMHDWDFDSDIDEEEGDEEGDDDDNEWPEPDDFPHGFPSILALAAGNAPPPSMM

>CF317_000136-T1 CF317_000136

MLRSDTPRRESLSNFGTMNAPPAGTKKHKLGRLLGGSSSSQPDDERPPPRTQQTDSAYASSDNANNEIIQVENDGSIPNTHKGQNLSLDRGTGEVYDEDTGEVVTVVTTTTTTTTTTTRSGGGRPQQDVRRDVQTTQHQQPQGQSISTTTGGPAQYLNQPSRDSTYASGGSGLTAPDIPVRSSRRSGEYDTAHYPAGSSASDLPTSPSRHNFSYPGRNPPNPPAATETGYTPQQEAQAKRSGRFADLKAAAIGLHGVGETLRGTLNSTIDQQYPSHNPNKAAAAQARNAAVLERGKNEMGRIPSRRQQPVDQVSGYGGSYTTQQQADPVSSHGRGYSNQYQPEPMSSQGGAYNPHHGTYSSQGSLPPDPNSNLKGNAVDGSHSAVFGLTPDGHRHLDTRSPTPPPQHAPQGTNDLSSVSDLANNSYSAVGQAPSRGTMGRVNSGTAPESLTGNVERNVERVPTASHPAPGTNQAAAMSGEVMSPVSPDGGTKKQGTFSKLFKRKPVAGNEERKSYY

>CF317_000137-T1 CF317_000137

MTALQPAMQLSEAEASAQSYGPKLQAPDDLGDDGWKAHTTDDDDFDTDDEVRNCGGFEHEASADNMLGVSISRYQDDMKSPTTPAATKSQILEASKSYFAAADDDRDEILEVPSEEIAPDQRGRSPHALPERNIEQEEQMQAVPLPRWVPPSTEKTERRSSLFSSILGAARPRASSRPGMLNGIKKRIPLRPPINLPKRARPSGSAKAMPGSAAPDPLRPGDDRQGTFKDVEWQSETLENGIPSAKPARLHSRSGSYDDAGKGASRRRPTQRRRATSDQSLYLRREPTGAATFDDWNQFSDFSEMVNSRKQAIHDTWQDSAFRIPKLTKVSQGRSPKPMDSNYNAETINNTNANTNSSQLPDVYQNRRESSPTTNGKASKHPILKSTLSRTKGDLVIMGGYRGSILREAQPPYRQLWVPIKAGMNLRKADLEVGLTREDELKMEEKIVADGALSHIGPVDICRRLLRKCRRSPNVQEGKLRVHDYGYDWRLSPDLLADQLIKFLEALPCNSTTLPVEERGAWIIAHSLGGLLTRYAINKRPELFAGVVYAGTPQNCVNILGPIRNGDNVMFSSRILTAQVNFTLRTSFALLPQNGVCFINKKTGERYDLDFFDANTWHKYHLSPCIRPAISRNRPEKKTSLIGSISEMVAQQSRRSSWLPSRNDDSSISLQKDDSSSARDKVNEAKEDAAGAAKGAVPDAELQPELAGSASQSSKNKPAVATQSTIPVPLAKAYLHNTLASVLEFKQQLSHIPEQQSRNAYPPAGVMYGKQTPTVYGAFVASEEAIKYDDAFDDLAFAAGDGVVLASAAQLPPGYRCVRGGRIEVQRGHIGLLGDLEGVGRCLNAVIAARNRGVGLGAYDR

>CF317_000138-T1 CF317_000138

MSTPSVATTMLPAHHSPYGYPHQSTYAPTTSRTYPTNNTLPAPPRLTTSYHSMPQHTQYQQPQPSPATLKQPSYAPSMASTQASGPGRENNKKGPNWNEFYKNGPPKEIIVIDDDSPQPQASSSNTQRHDVYSTAGRKRKVDQGYEAEYADSPVYSTRHGQHDNSSSSASIHSAARTNSIQTITAPTSLESYGSNPASNSYEDVRIGQKRKRVIPEKNTRSQAKKKQQEAVPDPFLDYIPPTKPTRKAPEVIVPVIRDSIHKHQKVDDEDGHYVIEEGLPLTDRYDIIKLLGQGTFGKVVEAFDKRRKTRCAVKIIRSVQKYRDASRIELRVLSTLSMNDKENRNKCIHLRDCFDFRNHICIVTDLLGQSVFDFLKGNGFVPFPSSQIQNFARQLFTSVAFLHDLNLIHTDLKPENILLVHNHYQTFTYNRNIPSSSHTTQRSARQRRVLLDSEIRLIDFGSATFDDEYHSSVVSTRHYRAPEIILQMGWSYACDIWSIGCIIVEFFTGDALFQTHDNLEHLAMMEAVCNGKIEPRIIRQVLQNHKSNSSNTAAKFFSKNKLDYPNSETSKASRKYVKAMKHLSDFIPANTAFNKQLLDLLRKIFVYDPKQRITAKQALKHPWFRETLIDDGTEAIRIRDERAAQTPAQQVPGPEPKRARV

>CF317_000139-T1 CF317_000139

MAQPPPPQQRPSFTQQSPSFNHAYPQSNGRPHFSPQSPTGSMSYSSPSPQPQFSPPYTGQAPPAKRPRLSPDAPSSFAQQPVHTPLAGSPVNGQLNGIPHSGAQRSGSMAPPQQPFVKREEGEMNFLRDTSFEPAGGPSPLALNHAPSPNPAPTFTSVPEPSTANGGHLTPGSSGAAMAAPQPVVNQEERQARSNQRQDWEEARHSQHELWDPFLFGATLNDKLKSISHRSNLLEPQAGVLVNTQKNQPPPMVRVNGLEGATRVINKGQSILDTKEKADRLNELVKLLSLSAKARLTGLVQAAARLALERRQHSQGRIPEDWSDIAVTSKPTGAEPHDNSSLAVSAGMKRTHAQANDETTSQHQSQGLPHPAKAEFEKLSSRELKAEQARQAKRRKRNEVITAQETEAEKQAEAAVIAAAAADSEKKTTKKERKMAETKVTELQQHASTNEAARMATANLLGRFGSSKKKTYSWMAGGGGAASRAPSSTATPTRSTSAAAPAKDKAVEVQKGPQIGQFDEGTEPGVQARDLLLVLESDGRAAQSFVRASSIIDEMAARLAT

>CF317_000140-T1 CF317_000140

MASKRKAAAISGTAENEDPIDPSDELTFTGLGGCQEVGRSCHILQYKGKTVMLDAGMHTGREGMAAMPYFDDFDLGLVDILLISHFHLDHAAALPYVLAKTNFRGRVFMTHPTKAIYKWLIQDSVRVSNTSSTSDQRTSLYTEADHISTLTQIETIDFYTTHTINGVQVTPYPAGHVLGAAMFLINIAGLNIFFTGDYSTEQDRHLVAAAVPDQKKVGKIDLLISESTFGISTAPPREEREFALLKSITNIVNRGGKVLMPVFALGRAQELLLILEDYWQKHPELQKTPIYYTGNTARKCMVVYQTYINAMNDNIKRIFRERMAEAEASGNAKGVSAGPWDFRFVRSLRSLDRFDDVGSCVMLASPEVAPDQRNGVVMTGYNVEGTMARTILSEPDQIPAIMSGSNAQSIGRRNKDDDAVLIPRRCSVEEFSFAAHVTGKQNLDFIEAVRAPHIILVHGEKSQAARLKSRLLDNNSKRTASNADVQQTKVYSPENGAEVKIPFRKDKIAKVVGRLAQTPAPTKSEDERVISGVIVQNGFKLSLMAPEDLKEYAGLTSTTVLCKQHITLSAAGVDLIKWALEGTFGAIEEVGQVKSETNGHVEQTNGTTKMEVIPKEEEADEELISEPPRTFLIMGCITLKWSGQAKEIELEWEGNTMNDGIADAVMAVLTTVESSPAAVKYSSSKAKHHHHHDHTTKPEESPEEKAVQQVNGTTNGNHKPLALRNQLAYLSPEERFSRLCMFLEEQFGDNISPIETPRISHLTQPDSKVTKSEAEADTDANSDDEEAIEQRNAALVASERARLAAQGIPVPGLEIKVDKMVARLWLDDLTLDCASKVWKERIMAVVERAVETVAPLWSVGGGSR

>CF317_000141-T1 CF317_000141

MSTPPALESSSSGGKWNQGDLIIFLIILTVLAGLGHGLNKVLTSLDQLFGSGGSKDSSNDDKKQTTPKDPKNQQSLPDLNVAIEDLHSAAKARGVSAEVTLKLE

>CF317_000142-T1 CF317_000142

MVRFKERAPHPEKAKESPNGIDHEPPREAGIRDRIAHFTWIWFACTMSTGAVAVVIAQTPNRFPGLDTIGTIFYIVDLVLFCAFLAIITTRFILVPKKFLASLHHPVEGLFFGSFWVSVSLIINGMQAYGVPHSGPWLIKALEVLYWTYCAVVLLVGVFHYFFFFREAKLSVSDALPAWIFPIYPLLVVGPLAGTLIPSQPPHTAFQIWVGGVMLQGLAWTVSLLMYAIYTQRLMVSILPAAPTRPGMYVSVGPAGYTAAGLISLGLQAPLVLSPNTFGVTDTNVAEVVKVMGILAGIFLLLFAFWFFCVSTLAVISGIKQMTFTLNWWAFIFPNGGLTLGAIQMGKAFNNSPGINGICSALTIMLVVLWLLVAVFNIKALWEGSLLWPGKDEDKDMKGIRWGSAKNAA

>CF317_000143-T1 CF317_000143

MSNPRIEEVSDSDPEIDDPSDFLGSDIIRRADAPAPSQSASPPQPQVAAVQAPNPTLTRPPPPTQNQPTPAEMAAQRAEIKPYTTLYPIYFSSARTRHSGRRVSSKLAVQNPLAFNVFKAVRHVVGPSIRVRLEPDKTHPKDWANPGRVKVQLFDNDTKEPLHPKIRNKQYLYKLVAEYMQDHPTQREDPLELKIQGLPVPENFLDSKVAVPRGWKMGDVLPVHSAAVSGGGVSDNFFKDAMEEMKQAQAAGQLPAGGGGPGGMDMSAMMQAMQGMGGMGGMGGMGGGGGGASGGKKKDKKKG

>CF317_000144-T1 CF317_000144

MALYYDAATVLSSEKHQGSLKSRIYDTSTVKSNPAQVYALISETAKRDVFLKEVLDHAQILQDEPKLTPLLALLLCHDHFFAKAGIAAAAKHPLRQSIERHKARLQSEFTRARLRRKCGSIDALKSSLDKERASHARPHPRWARVNTLITKDTEPIVRSIPGCKRKRDLDDVLDVDAGAVSFCYDAHVPKLLALSPGTDVTKTDIYKQGELILQDKASCFPAYLLLGDESKEKIGDVLDGCAAPGNKTTHLAAVMGEVGSKGKIFACERDTTRSKTLRIMVDKAGAGMVHVLARQDFLALDPQDVRFGKVTHLLLDPSCSGSGILRREDIPKLALPDDPRTSTKATTNTQPKKRKREDAEEKGSEEMVAEVKAELAPTDEEASIAVNEERLQKLSSLQTRIVEHAMKFSAARRLTYSTCSIHEQENEVVVSRLLASQVAQTRGWKVLPRSQQVDGMKRWKHRGVRSKPDDSSDENKLTEEGLDACIRCNPDDEEGTMGFFVCAFARDVDSGETPYSNGNSDRISGNDGSSDEGESWEGFD

>CF317_000145-T1 CF317_000145

MPGGNQHANQPDINLPDLDLEKGPPEDKTQGRDHDDEHKDHTGQHQQHDHQQHKGDSQEGDGPHQQHGSKQGGSHTSPDDSNSGNMKIEKVSKKEQPRQGLFDGPPVKDLDPAADRIASHDTKPLTADHFYELMGLQQPRSAEEFNTKLQKLAIHNGLYKTIWSELKWLQYKYRIFEITTYVLMTLQLLISAVFIILGSLSRVEAHTAIAVLGAISTMIGGVLALMRGHGLPNRLREARNEMQYVKFEAEELFWDFRAGKAILYKDIKKLREDYLRVVEELHASHPDNYSKAARNIMSGSSITKTKVWRK

>CF317_000146-T1 CF317_000146

MSLQRSDIRVEIQPPPGSVVAPNGFPADPHPKDAPQVFLDAMEVRIKVFCDEQKCALEPELDNDDPKSWSWIAYQQSPESKEKVVPVSTLRIVPPPHPPHPNGFHDPEEEPYVKLTRVATMSYARGQGLSRYLMNHAFEFLVSQPQKISPDWQGLVLTHAQVSVEGLYAKLGFVTDDRLGRWDEEGIEHLGMWKRLSLPATTS

>CF317_000147-T1 CF317_000147

MASETASNVPKITLHWLEVSRSHRILWLLEELKIPYELKTWKRGSDRLADPKLKEVHPLGKSPIVTVEREGKDPLVLIESAAITEYLCDYYGKWLIPARYPSGQEGNIGTETEAWTRYRTYMHYAEGSLMPLNVITLILSSIRNSSVPFFIKPITNGVADRVTSMFLKPNFETHFTFIESQLETSPDGGDYLCGKDLTAADILMSFPLDAGRSRSGMTQEQCPRLWRYVDLLHQREAYQRSVQKIQDIEGSFKTNL

>CF317_000148-T1 CF317_000148

MYLFDLTATEHNSTEKTSKQKALPLHKSNHDTAAKPQDIKRTSSAIADKHGSRDEQQALGFAENWGAKAFKSLKDIDSGKRLGEGFKATWTSARSGENMLDLVESDSDPEADDVESGDEADEYPY

>CF317_000149-T1 CF317_000149

MVNFAARCPQPQSTDRPATTITVAGNTDGSESGPVAAEEHTTGDGESVSDGSVSMDTTDDQDEGVVSEGSTAMDTTDDQDEVLARLAPTTRNRRGPLQPMTDLEYEIDQAETRTSVETDRAATSSNTTTDTFSDTLQQQLPWTLNANEYIFETDAQAVQARARDRQYRELQRHAAQFRATSHDPMIGGRELNAPLSRQSSIDQALGGGLGGMEDDGEFRNFLDGARLVRRRPGVVPGRTADVMDVVRYVDQQ

>CF317_000150-T1 CF317_000150

MFARCLARPSICTRLHPPPLARGSLLSPPATIRPRLQSTAPTPEPARKPSFWRPEKPPTSRKWHLYVVIGLISFTVGSWYRILVLNNKKDDPTSFKTFKLVSKEQVSSTASIFTITPESPRQLISTAAWKQGIWNVEFKQPQLQIVRAYTPLPPALADEVDAEIPDELRFLIRKDARGGEMSSYLHRLPVGATIELRGPNIEYPYTAIGEGVKNVIFIAGGTGIAPAMQVAHAMFDGLNERQKKEKALHILWANRSREDCAGGVSSDATPVATPKPASSSWSWASIFSSTAKIANDTVAEVENEQLQPNAIVQQLNSLKSQSGERISLSYCVDEEKTYLHTGTIEHALSSIKDVTGQEKTQIIISGPPGFITYLAGPKVWQDGKEKQGPLGGRLAQVLGKEGRADVKVWKV

>CF317_000151-T1 CF317_000151

MSRLGFSDLSKPAALQLPGRDSLELASLADSDGADDNDLDNDGTTESSSVSGLPSSRRLSLENEDPLDSLSAAQRSRMNRSYSVSSAFDFTPALFPLSTTAGGYTALGAPATPSFDRAGGIENLEKQKTLTYFNGLSLVVGLVIGSGIFSSPSQVNLHVGSPGASLIVWTISGLLAWTGAASYAELGGAIPLNGGAQVYLAKIFGELAGFLFTWTAILVLKPGSAAIIAIILGEYVVRAVIGAEVENINPWINKAPAFLAILLVTLFNCLSTRFPARISDGFMLFKFVALFAVTIIGIIVAVTHLSYRGEANTEWRDHGWFDNTSDSISEWAVALYAGLWAFDGWDNTNYVTGEFKNPSRDLPKVLHTAMPAVILCYLLANVSYIFVLPFADMDKSNAVAVQFGSKVFGPIGSLLLALVVSLSAFGALNATTFTSGRLVYVAGREGYIPDLFGRIGLTATNEQQRYTPQKLRTRNAMRKAVEKVAGDKDGTLFLTPVPALLLNLILTTIYIVIGEFRTLITFYGCAGYSFYFLTVLGLIVLRIKEPNLERPYKTWIITPVVFCCVSLFLVSRAVVNEPGQAAIVLAFVVAGVPVYFWRVAGRDERGGRGENIGWRFWRRWGRT

>CF317_000152-T1 CF317_000152

MSTNNAAAAAPTAAEGGGTSTFTAVTRSIALFLALQAGIKYFTSGGPQTGSTDAAGAGGVSGVGSFADRPDVSTVTNYSQIPYNLAPIWPADSALDIRVHVSPNLQLPPLASLPADSLVVSEDNFKMGDYKYSRDYNTLINLPTEVQNNGSFYAHIFAAIHGHELDPTSASYDTASAAHYVKQLNHYLPKKKVRKERNLLDKPGEQEVEPEDPTPQVSSYWHSNFSLAVVPGSGVVNWRSMHPGMRKDILMEPTGARDASGQNGWYYPVFFLNTFWQLRSHMTELNDTVKSVPLNINLYQLPNWQYSILASMDEGIKQQQNAAAFGTANPNPAGGDGSEMEKFKEILLDSNPWLLGTTFIVSILHMVFEALAFKNDISHWKNKKDNIGTSVRTIMSNVFMQTIIFLYLVDNSDGTSWMILATQGFGIFVEAWKITKTANVVVKPPAPGSTFSFLPYVVVLEDKHKLSDIEEKTEQYDAEAFRYMAFAAVPLLLAYAIYSLVYEKHKSVYSYVIETLVGSVYAYGFLMMVPQLYINYKLKSVSHMPGRALVYKTLGTFIDDLFAFTIRMPILHRLATFRDDIIFFIWLYQKYVYKVDYSRVNEFGQGGDEEDATKEKENEKAVQGQNTASMKPAAAVATSSGAEKKGGAGGGKKRR

>CF317_000153-T1 CF317_000153

MTTSTPESIFPISNSPRVWLISAADTPVGISLLRRILAHGDFLVAGIDHQAFESDSYKAKGFKEVLTEIGHYPARKDWQARLKVVALDVRSQAQCQAAVATAIHLFGKLDILFCCTSQAIVGAVEELGCSTGTSLVREQFEMNFFGPMNMIKAVLPTMRSQHNGHVIVLTGITGHLGTPGLSMYCSSQWALEGFCDSMAYEIAPFNVKLTIVQSSIEIGILTNRVVSAPAMAEYTTEGGHQAPLFRGILDGLLNRLPGIRAQYPNAHAGATPPGEDKERKPDEEDTSPTATSEAERASGPFLLSRDETASLIPPLSCGHAEKLVAETVHAVTAIGGHENPPARHIVGIEGVASVKEKLKTVSEELEEFVDASVSVDCERAGDKRPRRTSHASQADSRGHAKTNANGNGNGWDEPVDHVHPIHEAARVLQAL

>CF317_000154-T1 CF317_000154

MDLLATVRKEGSRGGRGEFNWTDVQNSSRREHYLGHSLMAPVGRWSKGRDLNWYAKANGDEQDGEDPAQRAARERKEEIRRVKEAEEDALARALGLPVPDRNNANMEPVSDKREVDKVVKETSGIDYEAGKGIGFGRPSQQSGQMSETIEDEDTTMTTARTEVMAKSASHDITNPTGSINEEGIVIGQAPEIEPVRANATTEDAPGQEVLTKDTIDDVEIVHARVRLINHAESIASIVKMHTRAQAAVSDNPKDTP

>CF317_000155-T1 CF317_000155

MENNRKPYEPVRVEMIGLESGLERFSKDGLVTLPPVSKWRNNLTALSHYENLFFTASDDCIAVYQPEFPFQTLRRQPALLIKPELANRNASGYLSQRGGAQDHCINHLMVGDLGSQEILLFVTDSGNIEAYYTSAVLEAIKRAPGQYCEGSSSDVLGLRPFFSHWVRQSAWGIDIHKEARMIAVSANVPNAAQPGISADSSAVITVFAFALQPPSSTASESAGDEPDSADNVEWTVWDPRYHSQMPKRTRNYKIVLAGWEGHSNNIPNICFANTSADDGDIWLLSTDITGEMKSWQVWKGSVFRGWNFGTAEGSVRRWMHHQYPGWNVAALDISCFRPARNNDEFIGSSKAPVYFGYHDQGESFNISRIVDRQSENSHYHPSNLEADDEDTPSEVDEIHSTADEDASEYDEPDVMEDVQSPTNPTSSVPESDWDFEPFDHSVGREIEPFDRPVEFQVESEDDSVLESDSSSGDEDDSMEDERQSSSPNPRIQRHIRRLVTPIQNPNAKTPEIAMIHCSDSHVRLLGSPKARFPHIFCASILRQIMPQNIQTHMQGLEFAHMDRLNMLQKIAELGIVLIATQTGRVAVCALTRRPDGLLGFRVDWVLPTKKQERRGRRPEFCSLIGMAVAPIQGRWKSRTDFANDDEPSNFEEQEVDGVSTSFDPNVVVLRHLATPQERHAWKSEKGDPPAVKMEHRPWTSAPSEVPSWQATETSRRYRVMLTYSDMSVLSYEIWRDVEKDERAQEREG

>CF317_000156-T1 CF317_000156

MTPSSEQKNIVIIGAGIIGSTTAYYLTRHPSYDPSKHKITLLEATSVASAASGKAGGLLALWAYPKSLVPLSFRLHKELAEEHGGKEKWGYRPVGVGAIELEGRRVPKDSIRAAVHDKDAKGKKDPVGTTHSGDVGPIGDDSGYEKNDVSLEKELGVDRKQLMKMGLPEDLDWVAGDSVKGYQSMAPEGQTAQVHPFQFTTSMARLAEEKGVSVVIGKCEKVVVEGGEVKGVQYREKESGEEKTIDGVTDVIVTAGPWSKKVWPGAPIGNLRAHSVCIRPSRPVSAYCLFTDIDLPKNFKEGVKSRAMNVTPEIYARPNNELYACGEGDHMVPLPETSADVEVDDARCQDIVDYCASFSDEMRDGEVLVRQACYLPQVEYGGGPLVGPTRTKGVWLAAGHTCWGIQNGPGTGKLMSEYVFDGEATSANINSLDPRKTTRR

>CF317_000157-T1 CF317_000157

MVNITEKIKEIEAEMARTQKNKATEYHLGLLKGKLARLRAQLLEPGPGAGSGGGAGFDVSKSGDARIALVGFPSVGKSTFLSKITKTKSEVAAYSFTTLTAIPGVLEYGGAEIQILDLPGIIEGASEGKGRGRQVISAAKTSDLILMVLDATKRAEQRALLEAELESVGIRLNREPPNIYLKAKKAGGMKITFQSPPKYLDEKMLYNILRDYKMLNCEVLVRDEYATVDDFIDVIMKDHRKYIQCLYVYNKIDAISLDFLNELAREPNTVVMSCEMDLGIDDVVARCWQDLRLTRIYTKRTGVEPDFSEALIVRKNSTIEDVCDSIHRTIKDTFKYALVWGASAKHIPQRVGLGHPVTDEDVVQIVSAWKA

>CF317_000158-T1 CF317_000158

MAKKKKATGALLQPSQPLPKQVVENGRPEAPAHSRDLSRGNTADIASVWDDMLGDDAEALKGFPEPQRNGFAMVKAMYGNETRLDVGRKTVWGKPKLGFEIAITARHDASVFVRLDIELPENYPKLGPTIRLIELEPDTAELRAGLKSTIDQVTKSLQDDDAMISSMLSEIETKLSDEVDNQASRAQGATLEEERTATEVAAKADASSKQQLALRQKEAEAAAKESQLAKDVEIQRRRRQLLSSNDSAPEQLTTVGEDYPSSCIVFSHDVTCHDFSIDADLTFRAVQPMSVIYQRDDKKVMLACPYRNGDIMTQQLVLKEVRLPPAMDSEADHRAALSRIETALQEAKSFDHAAVVKIHGYKLVMVSNKGAQPFWNLFILSEYAQQSLTALLDMVDSLQAARIRTYTRSILDALEFYDRRGYIHPAIHAYNILLFGSARSSYQAKLSDGYGTALQELIEKAGNTQQSEPVQGNWAAPELTNGSSARTNKTCIWELGVVLLQMALGNELTKTYTSPEDALSRAGLDQDFDHLISKMCAISARKRPTAFQLQSHQFFKSHERSIFAAEIQRKTSIQTINRSKSVFDSRWASEWEPVERLGKGGFGVVVKARNRLDGHFYAVKKLTCKSIQDTEGIWGEVRMLAQLNHPGIVRYFGSWSEEDHQDVADTETSTAPTDTRSFAIPTDSAAPRSSLFALPSTGHDFMDPALSQIPDVEEDEGEEEETDSSDDGNIFGYQSAPSGDGEDSQAIQSGDDDDESSSNPFELQAAADNLDDGPNLFESNEKEESLKRTNVPSRPQPFVVAGGTPSAIRRPPQYYNKPSTLYIQMELCETGTLHDLIKNGLPDSTDDAWRIFRLLLDGLNHIHSLGVVHRDLKPMNIFIDSQKMPKIGDFGLASPGQATVNGHKIATHVAGPMSKNVGTAFYIAPELADIKSSGQYSTKADMFALGIVFFEMCFPFQTGVERIDWMQSMNRPDWRLPDRFDTEQYKVQGRIITSLLTRDPDQRPSAKELLLDPEIPEPLEEEKEQRFIQRLMHGDAEQLQTVMKNFMSKTATKAQLLAYAHIDKDGFEPPDSYIVCSIQGKLGEVFRCHGAVEGSRQTIFPVEGFYLNPVSYVDAAGFTVQLPHDLTVPFARAIAVQKPRYNKSFCFGTVFRRRGPGVEPLCIPEVDFDLVSYSAQDLSLRDAQVISVLDDCLTKLGSLFMRSFTVVISHGDLLDLILRACEVPEARIDPVKHLLSSLNVGKVTWKQVQQDLQLPSVGLAATTVAALSHFNFSSNFDDFRQLVLANLRKLKKDDSAMKATRTLNRLQEVHEYLVLLRVNVAWLFSPLSNTSEMLYRGSVMFRCVESKALKTVVVGGRYDALIRNYQTPTQKTFARAAGFRINILDLASYAKIDAHGPGNKSSKIRTTLPPSIPARVDVIVTSFDETTLRGTCIEIVRSILDAGISVELSESFESMEALERAYSDIPKYWLVIVRPIGATQRAIKVRAPSREETDVTASELVGHLREELGERQPATVDQPVLRRTRSSHGAADRENIVILTPQHKSKKVNRAAIVDSARSAAQELAETVSKTYKVLAIDTDDDTLHRIRNTRLTDGESWRGLRHAVALTDREYVQEIQEQLVDWSKAGQDGAFLCNYKTKTCILYDFGKL

>CF317_000159-T1 CF317_000159

MPLAERNNLVLDYLTESNAESVAPLFMKSFHAYEYFQRMMPDTPQAAKAWAEATQHAIDDPNTICLVVTDTSTGAIVAHGRWVRPKAEGEKDQPGNEEDRWGDSFMEACDSKMAEDLFGAFHKNREQFMGEEKHWYMELLLTHPDYQGKGCGSMILRHGLELADRDQLPCYIDSSPMGKALYEKLGWTLDHKKDFDYGLSYYFGVRQPQITS

>CF317_000160-T1 CF317_000160

MGYLSLLLPKITSNEERSPSFMASNVDDGDDKTVHDTARVSKDRTSEESGNFTYDKDVQAGVRRVQATASVWTKSSLIAAYTFVWLVYVVTSLEEVIIRALNPYVVSDFQLHSLTAATSIMASIIGGLTQIPIAKILDTWGRPQGLALTLFIWIVGFIMMAGCNNVSTYAAAQVFSAIGARGVSYCMTVFIADTSSLRNRGLMLAFATSPYIFTTFAGGPASESVLAPGGIGWRWGFGIFTMIVPAVVLPLVFIFWYNQRKAEKKGVLEVTREKLSISSLRQYAIEIDLPGILILATGMALFLLPFSLYSYQSRGWQSPMVISMIVIGGVLLIFFGIYERFFAIKTFTPFRLVADRTVFFGGMMFVFNFAASSIWGGYFYSMLQVVWNLDVTAASYISSIFRVGQCLFAIGVGIAVRCSGRFKWLAVYVGMPLNMLAIGLMIHFRRPSQDIGFIALAQIFVAFSGGTTVICGELAMMAPSDQQHLAVILAILNLFGSIGSAIGSTIATTIWTSTFYEKLVQYMPPESDVPTIYGSLTQQLAYEEGGPERGAINRAYVETQRLMLITSICFVSVAYICVFLWRDIEVKHLKQVKGRVV

>CF317_000161-T1 CF317_000161

MSGKAVAGLFGGTPTVHVAKKQLAYPLYGADFDPLNHHFLLVGGGGGSSSTGVPNRISLLDTSNRSDIKEISDIVLAPDEDNVSSLAIAESSPQSLLAYAGINGSVKDQEAGKNEHFRSFRVPLPAKRKKEKDTSDAEPPAAHVAPTQPLARTAIFRAATGPKNDCYQRVLRLSPPTYADGTEPTIEEKPAQLAPRLIRRVATIASGLATQNEVITLPVTETPSATQAISRINLEKDEANDADLVGIDTTGTSFALAYATDHELFLQQLGDQKGTVKSEPVKVYETQSGPKGRSRIRALRFLSSRHLLLLQNRQDKSGAELIVLKISKDFAAVQQTLVKYLKNLKQAVGLDTCALSKGQDGSQQFVIAIAGQDSSVQIHTLDYIAGEGINSFKQYADALDVHSGPITRIVFSNFISPKLPVSGQTPPQYIRLATVGVDKTVVVQTLPLKPTPVVGTGKHMPRYVLISPSGTGAFIYSLFLALIALFICTTALITFLESQGALPPIIGASKYLPQRWRHEYVRPYPYAHDNPGPMIPDSMPAAQSIIDRIRLSEATPIIEKIQEIQEDIKLAEASEAIKSMQSNIATAIPSMQEVQDTIAALVARQQSSDSEPNKAIIVRDLEHAGGELSTELRREADIVREGTLKRWESLTGHEQKTWKRKLKEAGQWTEHQGEAILKGVFFGQLAGVVGHIVG

>CF317_000162-T1 CF317_000162

MPAFLCFGYNQAVAGGVLTLESFVSTFPQLDTVNTTGAQAHYNSTIQGTVIALYTLGAMFGALSCIWLGDVLGRRRTIFLATFTSMIGAILMASSFSLGQFIVARIVLGLGTGGYTATIPVWQAEISSSAKRGAHVVTEGIFIGAGICFALWIDFGFYFIKNSSISWRFPFALQIVLNLPVLAFIFTLPESPRWLIKKDRFEEARTILAILEDCPEDAETVTLDIQSVQTSLRIAGTGSMKDLFKTGNSRILNRTLLAAACQMFQQMCGVNLITFYATTIF

>CF317_000163-T1 CF317_000163

MEICQPLGGLLCFFTIDRFGRRRLMLSSATMMCICMIILAATTSQPNNTGAQVSATVFLFIFNFVFPLGFLGIPFLYATEVAPLHLRAAISGISVATTWLFNFVIAEITPVGFNTIGWRYWIIYACINAAIVPTVYFLFPETMGRSLEQMDLVFEESQNMFAPVKIAKRLPVDAVGDAEAKDRVAGEKSLDGSFNKDLEESDEVERL

>CF317_000164-T1 CF317_000164

MAATGTHGGPDDGVNGDYDPLAKYTRWHNFPIAMDIPIGDAGEEVELNLQDLDEPTELCQLLENEKVDRKYWVAVALAYARKGLLDYAIEVLRQGSHVFARSSSKDKLSLLGCMSWMYLLKSRHAPRTVGEGQDPADVKTKEHYLREATNVMNEATRINPAHPPLYLTRGVLNLLRAALATSTKTGPGAENERSDALNQALKCFDEASKLSNHRNLLALIGRARSLYLLKRYSQALTTYQQVLGKMPDLSDPDPRIGIGACFWQMGYHDQAKTAWERALTLNPKSNIAHTLLGIYNLRESSKYSSDDRQFQTLYTKAIQIHLKTAYGLEKNCALTNSVFSTYFLNSRRFDTVDPLSKKAIEQTDVNAVASDAWFVRARKEHVSGNIQLANDAYNRADQARGGQNAGYFPAKFGLIQLMIESGDIQNAKFRLEKLFERNKAIEIMTLLGCIYAEEIFAALKANPKDDKPNDVRKAVRMLETVRKNWKDEKAKVKTKPDSAVLLYLAQLYETDNPLESEKCLAEVENLQRRAIADENDFDPAAEDFNELIQEQMPPSLHNNIGCFYYRDEAFEQALQRFEIALNATLKLHEKQAEEKQRAEEAGESTTELDQADTDALVTTISYNLARTQEALGQREAAIRTYEGLLNKRHSEYSDASARLAYIALVESPQDQGPKKMKAMYDADYSHPEVRALMGWYYHSSKKKTANVAEDQEFRHYKHTLQGYDKHDLYSLVGMGNIHLTIARDMPRNSESEKEKRSKMYQKAYEFFDKALQLDPRNAFAAQGVAVALCDDKKSYSDALQILVKIKDTVRDVSVFQNLGHIYCETKQYSRSIENYETALKKQVLTRKAKADFQKSTLNADAADGEKTNGVNGTGIAEPDHASDASLLSCLSRVWLLRGKTDKSILSHTTSLDLMKRALATQPDSPHLRFNVAFIQFQVVQLVNSLPETQRTLEDLLAARAGLEQAIETFEAVAQSPQPPYPKSLLEQRAAMSKGTMMKQIERATQQQESYERANSEKLAEAKRRREEELKARDAAAARRREEEEAREADIRKKREQFMEEAAAKAEEFRQAALVREAADYTTDEDTGERVKRQKKKSAAGGRGRKRKQRDDEDGFIEHDDENDSDASHGGARDRTPLSGTDEDATKEKPSKKKRKLERKAPKKERPSAKKEKPGKFKSQAVVVDSDESEEEAVATPERGSRSASEAPGNVPTPGAGGEDGDGDVPMDEPSRPAPRQRKQARLIADEDEDDDEDAEPVSAPSGPKSAARVVDDEDDE

>CF317_000165-T1 CF317_000165

MASFKVSAVLKGHTSDVRAVLHPDPSFALTASRDGSTKVWKQTSQSPPAYEADELTSGNQFKTCLAYLPPSKEYAEGLVLTAGQDTLIEARQPSTTADQNADAMMVGHANQVCSLDVSERGDFIVSGSWDSTAKIWGVGKWEVELDLPDHTATVWAALAYDRETIVTGCADRAIRVFTTSGKQLVSFDGRDVVRALAKVEGHPTGAQIASASNDGVIRLWTLDGNLRGELHGHESFIYSLAVLPSGEIVSSGEDRSIRIWKETECVQVITLPAISVWSVAASPNGDIIAGSSDKLARIFTREQERQADAATIAEFDQSVQSSALPKQQVGDVNTTDLPGPDFLTRKSGTKEGQTAIVKDTDGQPTVYQWSMSQQEWVKVGQLVDSAASGNKVMHNGQEYDYVFDVDIEDGKPPLKLPYNATQNPYDAATKFLQDNELPMSYLEETANFVIKNSQGAQLGQQAQSGGADPWGTENRYRPGEVGQSSYKPAAPQETAARSTLPQKDYLSIVLGKPSAAMGQITKLNAASNAPLSQSELDALSQLASKLDKHNFQAKPSMQSSVVEPALTPLLKIIATWEPLKARLAALDLFRFAAAATSEFPVPGDDSLTHLFTNEIFEDAALRENYKLAMVGYRCFANLMYGSDTGRVVTQVYTDNIIQHLAAGTPMITDKKDVSFAVAHATLSLNTAVLISSEKTDQSEQQALKLIEVLVMVLDACPEVDHAGSNPLAQSTEGAYRTIVALGTLLVRFKGNEDLKKAARSMYKVDDVLGDLKAKKYNEEPRFKRAAGEISRLLK

>CF317_000166-T1 CF317_000166

MSVKNDHILTLSCPDKPGIIHAVTGLIAQHDLNILDLQQFSDPSSQKFFMRVHFGHAESTSHLTDAFATLNQDLQCEKFNIRAVSRKPRVLIMVSKIGHCLNDLLFRVKTNQLGIEVPVIVSNHPDFKELAGSYGIPFHHLPVTKDTKIEQESAILDLIKKNEVDLVVLARYMQVLSPKLCSEMSGRIINIHHSFLPSFKGAKPYHQAYDRGVKIIGATAHFVTADLDEGPIIEQRVARVDHSMSAKELVDEGSNVESQVLAAAVKWWSEDRVLMNEAKTVVFN

>CF317_000167-T1 CF317_000167

MASQQNGGYYANQFMNPGPAPRPPMNGTSTERPRPPELIRSVTTGPMIPTNFDSMNLGPASPSPAVSTPSQQFFSNASTLSLNSSSKTIAAPNVIKEGYVRCKEDKFLAGWNRRYLILREFRLDFMKDENGKLVQSIPLNTVTGVSRSEEVRSAFEIMRVANAKEGMGKAILHRDLPQRTITCEVRNDDEIYDWIDKIYERCPGMGGVSNPTNFSHRIHVGFDPQTGGFVGLPQEWEKLLTTSAITKEDYKNNPQAVIEVLEFYSEHKMREQHPEMYPPGMGMPTSVGGMSSQHKQPGFPTGSSIAPPRPAPPTEQQRYNTNQFLNKYNNGSSSTSLSPPQKPPLTRSQTDKGAYDMEADAARIKEIANQEQERVRREAEAKRQREADTRSQRELEAEQNRREQEAYNASIPRSKTPMAQQEIGGGGYSSPSQSRADPKYAPARAAPSAPGQPPRQGPTAQRPAPSAPNSVSKVPQVSSQSSSSLRQPESSQLRQPSPSSRVQDGRQESSSQRPQQPNGTSQRPPQQGSSVQQPKPLNVANKPQPQSKPAVDPRKQAEEALTSKPKPEEHKKEVRMSSMSESEVMAKLRQVVSKDNPMDSYSKQKKIGQGASGSVYVARVKESSSSAVAREIYRTHGPKGQVAIKQMDLRNQPRKELIVNEIIVMKDSKHPNIVNFLDSFLQEQTNELWVVMEFMEGGALTDIIDNNPVITEDQISTICYETCKGLAHLHSQDIIHRDIKSDNVLLDRMGNVKITDFGFCAKLTESKSKRATMVGTPYWMAPEVVKQKEYGPKVDIWSLGIMAIEMIESEPPYLNEEPLKALFLIATNGTPRLKNPNKLSRELKAFLSVCLCVDVRSRASADELLKNEFFRERCSLASLSELLRWREKGRQ

>CF317_000168-T1 CF317_000168

MARLSWHHSRSRTKSGNEKTYLGNAQDFFSRHTAEPSRQALADLVKVHRREKSDDVDGPRTPSRGSTITSPEWQRSRSRTGSGSDDARSSPDSPLCFREGSVYSPCDRSEGATRTLLAKGGMMLMRTGSRMSLSSAGSSSTIGLASPTRGSNVSAMSSASPRGEELRNKISAPFDFQHVTHTEGTQFAGLGRIEEAELFDQFTDAVTGQPAAPGLRGIEVSDLGTTAGHHALTTDTASKELNAPLSQLPITTHRPTPPPKDEVAEGCVTGTSAGKPADCSRRSGPRSPGFSPKSLLAAIDLALARADKSSPPPTARGQTRMTADEVDLNAKPLPDLPTTPTEHPIIHAVTTKDNTARAMIAAPLPTPPSGISHGIEGAHSPSRPLHQRQKSSYTLPRHMSLHPSAKASMPDLLSADRLSATPKTLPRHHSDMALSQQARIAHAPPRPCESRTSFAAIDTMDWEDAVDEAWDDVDETNGSATNTSFSSFNSTLSMASDPATSAVSTPLMMAPSRPLPAIPSQRVSPCNGAKSEKLGSVREDAQQTDLAGLGICSDPPAAPLPSLPNFSRSSSLHVPKRRSSMCYGANETLTRSSSQESIILSIASSYMDTQRSSKSSVCADDFITFSKAQEQPIFAFSTPGQDSQATEETESRKARPESGCLPSDILEQLSKVSASLNSQDVLEAQDTVPPVPPLPQHKYSKSSPKVIVPERRSSVAVTGRSRSHTTGARPRQTSGVSYSLFPAAMPTSS

>CF317_000169-T1 CF317_000169

MSKTHSESSDDFEFIETPAAPSPQPPAEDCGVRTTSYPTIKNAPLPADAAGSESFNNYVFVGILLFVPWFIARSVGGGLWLTLFFALFTSIPILMAFWAVASTISPRKNEKARYPGRPIEHYLHFRNEEDRGRYYGRHKIPMETFHEMYFDEKVDFKGDCLETMEYRHDWASFRFTLSLMKFFITGMIPEVIWHSRSQDEEQVRDHYDRGDDFYSWFLGPRMIYTSGIVSDIHKEESLEQLQDNKLAIVCEKIGLKQGETLLDIGCGWGTLARFASVNYGAHATGVTLGRNQTAWGNGGLRRDGISEEQSRILCMDYRDIPMPQPTGTYQKITCLEMAEHVGVRHFSTFLTQVYNMLDDDGVFFLQIAGLRKPWQYEDLTWGLFMNKYIFPGADASTPLDFFVSSLEGSGFEVKGVDTIGVHYSATLWRWYRNWIANSDKVKAKYGTRWYRIWEYFLAYSTIISRQGSATCYQITLVKNINSTHRIEGVESQFGLSGALAAGKARMLGNKAKDGVKELSNAVKGAFGQ

>CF317_000170-T1 CF317_000170

MDGDPTVEPELDAFSRVLPLPYRVAIILVCGIWAWALNLHYLYLVRIDVPALLRYSSRLGPTQRHVAHHRSTYRLATFLTLPLALSLVFFWIITGASKKAVAEWQILPQSYLIFFAICFIVPIQRMSHTGRSRFVHTFRRVCVGGLAQVQDGKFGDILLADVLTSYAKVLGDLFVSTCMLFSSNATSTSKPDRACGGQYLVPLVISIPSIIRLRQCLIEFVRVRRNRTPESGWGGQHLANALKYSSAFPVIVLSAIQRNYDPNKFDLSEIGLFRLWLLFVFINSFYSFYWDVAKDWDLSLFSSSRQRNDPEHPWGLRRNRYFHAKQMYYIAVVLDFFLRCTWSVKLSPHLDHFNDLEGGIFLMEFLEVIRRWVWIFFRVETEWVRNNKGPAPDDILLGEFAPKIDED

>CF317_000171-T1 CF317_000171

MMATSEPVSQSASDRFNFPWATSPDIIRTHQKDAYMTGTLSAQLSGIIRELLGARFAHKYSNATTHLSEFLYLCLTTLLGNRTLGEEYCDVVQVEDDTLRLASLVRRVGYIASVVFSPWILSKSIPALRRKLRLKLERNTARARQRLDGQRAYSNKHMSAPFALRFQEYIHDHLDVLTSLSPIYALSLAAFYFTSSYYHISKRLFGLRYIFTKKIAPSEQRVGYEVLGVLLVLQMAVQATLHIRERFFHTEPAQSSADVAMQTAQPVDVGAGVEIPIGSSDSLLLSDLDLHSHLHHPPSSSFEKNTNTPNVDPNENPHYHLEDDTTMEWIPAGQQRKCTLCLEPFKDPSDWVREKAECPLCRTTIQGNKVLPLRS

>CF317_000172-T1 CF317_000172

MVQTLKTLGPKPAADLDGDLMSDEGGFSIDQLMELAGLSVSEAVYKVHPPASGKRVLLACGPGNNGGDGLVAARHLYHFGYSPTVYYPLPTKKPIFDGLQKQLQQLHIPFANDQPFDKLAEDTDLIVDALFGFSFKPPVREPFDQAIKVMEAGKKPVLAVDTPSSWHVEDGPQDEGIGAKYMPDYLISLTAAKPNVGHFKGKKHFIGGRFLGQHIADKYGLDIPDYKGVDQIVEVPVDVKVQKL

>CF317_000173-T1 CF317_000173

MADVAVPERVSIPAAPASPSRKRSAEPEEPTSTALTTQDGESAVEGPPAKKTKLIRRKRKPAPPQIDPALLKTGQPPPQTGTTFNIWYNKWAGGDREDAYLSKTAAISRCKVREHSGWTQADKTPGSFFCLFFARGICPKGHECQYLHRLPGVYDIFNPNIDCFGRDRHSDYRDDMGGVGSFLRQNRTLYVGRIHVTDDIEEVVARHFQEWGEIDRIRVLTARGVAFVTYVNEANSQFAKEAMAHQALDNSEVLNVRWATVDPNPLSQKREVARIEEQAAEAVRRALGETTVRQIEGRETAEDRAQRRVDAGFGLEGYEAPERVWFNRQKELEAAEQQAQLEGPGERAMIEPASEQEVPTVEVDPTQEQLSNGILGSRALAALQGRSANATPTPGPSKPAASGPLVGYGSDDDED

>CF317_000174-T1 CF317_000174

MVAETKLYDSLSIQPSATQDEIKKAYRKAALKWHPDKNKDKPEAAEKFKEVSQAYEVLSDPEKRKVYDQYGLEFLLGGGRAPPPGGDEGNMPGGGFGGFPGGFQNFSSGGPGGGRSFHFSTNGGSNGFNFGNPEDIFAQFTKNSGGGDPDLFDLLGGMGGMGGGRSRRAGGAGFGASPRAQPRPPTPEVQIVEKDLPVTLEDLYTGAKKKMKINRKKFDARGQRTKEEKILEMDIKPGLKAGSKIKFAGVGDETDGGSQDLHFIVTEKPHKDFKREGDNLRTIIELDLKEALTGWERRVKTIDGREVPVRGGGPTQPGSVQTFPGQGMPVSKKPGQRGDFLVEVKVKFPTSLTAAQKTKLKEALP

>CF317_000175-T1 CF317_000175

MSGAIAAGWLGDKIGRRWMLATATVLSAASVVVFITSDTPTNLEGRRGLFVLGKVISGLGIGAITTTVQTYMSETIPTQLRGSIITAFPIFQLIGQIAGSIITQVMMERPGRSSYRITFATMWAFSIIPLVASFIIPESPAWLLRKDKVVQAVKAHGRLEANQKYPGAHLASFVRLRNTIARDGHQSTNPERRVGYLACFKGINLRRTGVVVFANMLPEMFGLSLMGSINYYLQCVGVDHSTSNLFMIIGIALGLVANISAFWSLSRIGRRRLILVTLVPAVLLWLSIGVVGTIQREGSFVHWSVDFDHPMLPASYVVASEASTLRLRAKTSGIGWFVNGGVSAGFGWALPYVYGPDALDLRAQTIYVVAGFAAVALVLSFFFVPEMKGRTALEVDRMFEMGLPAREFRHWRGGDTPLV

>CF317_000176-T1 CF317_000176

MPFTALGNGSWLRDRPEKDTFKLLVDTYRMRKADQFKFDETVVGVNVHTGSSRFAIKNDFFDFISTVKANDERRAQEQKPELLPHWWNADKTVQCVELAESDEPVGKKDIQKHYGDELMPMQLRMFGEEIDGTIPAGHPGQSLRDIIAAHEDGTGVPYMIHMSISD

>CF317_000177-T1 CF317_000177

MSQTLLFLGATGGVTNACLISALMSNQYKAIALVRTPEKLLKQLAKQPGLEENIINSNLTIIQGNALDVSDVKRALLANVNAHTDTVLPSMILSGLGGGPALNLDIWHPLQIAKLDNPTICEDAAKTLITALQEVYSEQPSLRARKPGACFVSTTGISRGPEDVPFGMQFLYHQVLALPHADKRKMEATFRDHMLQSGPVFTSVTGIRPTLLKGTGALNEGMGIEKLRVGTESKPATGFIVQRADVGAWMFENVVKEGEGGKWRGEMVSLTS

>CF317_000178-T1 CF317_000178

MSLNVVAPLMSCLGLKRKPGRHIFVEKSTFEPKYTGEEPHDAELEEAASKFVAILLHADSPSPQDPKLEETLKGTVSAYGYHDRIAPYILRKLENAIKKGAPLGKAVKQASERAIAEAVGFASEHPAYCTLIALGILVVMAPWVLEILGFAELGPVEGKSMFMLGQ

>CF317_000179-T1 CF317_000179

MKFQTTLLLVAPAATLAQNSLLSASVGTGVAQSGLSSLSAFASSINAASTETGSITSAAATASASSILSAASTVLSGTRTNSVTGSASSALVSASAEVSSVLDDESSSAAGAAGSGAAGRPMATAGALVGAAAIGFAVLL

>CF317_000180-T1 CF317_000180

MSFTSQDSAQSNETFGKDASFKLSQLIGSMSISPASRDVVLGSKEGLHIIDLDSPYSPPRLLPHRTPWEVADVQWSPFADRDYWVVSTSNQKALVWNLNLVGWRNSIEHVLHGHTRAITDINFSAHHPDKIATCSVDSFVHSWDLRCPSRPTNSFSDWFAGATQVKWSRQDEHVIASSHDKFLHIWDDRKGAYPARTIEAHDTKIYGIDWNRFEPSKIVTCSLDKTIKVWDTNNENNVPEKIIETDFPVWRARHTPFGYGLMVMPQRGSGDLYLYDGRADDSTHEGGRAQPVHIFPGHKGQVKEFLWRALGTVKEGIDHRDFQLVSWGTDRDLRLHAVPEGVFTRIGYEKGVSKPQRLRFTRRGAKYRTFRNEPTEQDAALAAPRADSFPTSNQFLRAKGRPSTNMGMGRPSVVQFKGWLQAGKSGRRTDMHGKGSARPDTNPISWMKNVKITSWDTDALADELTSVGEKFKKVDFESIDVSQRKATMSLESPWGEPESVSAYTRVDFRFPKAYPRDAGAIIRVQRTNLITAETQKKLSEDIQKIAEAHKMKGRGCVEAIVRYLLKEQSLDQIITWTMRNSVTDSKIIEAADIPQDMSDDSDDDPTDAVPKVAHTNPNINVPLAKGCAAIWAENGKLVCFFRPKDREPASFLSTLGTGQLDESDSSKLFGGFGKFQVDSLARKARDISQASPNDSESEDSDRSSVFTSSTSSSDSSVGLEERNRFVPWQRTTLDSLQRGKSADGSQKSTTHDGTKAGDVAQPTVISIHDFRDLLPAGQAAAQSYQLKENLVATCEHNRTIAAMYSLDTAAMVWGLLESVSSSASFQDDQETLADESEVFALTTPAKTREPCEVCKIGDLILVKGSSKTIGLQA

>CF317_000181-T1 CF317_000181

MALPKRIIKETERLMSEPVPGINAVPHDDNLRYFDVTIHGPASSPYEGGIFKAELFLPDDYPMTPPKIRFLTRIYHPNIDKLGRICLDVLKSNWSPALQIRTILLSIQALLGAPNPDDPLANDVAQRWKEDEAAAIQTAREWTKTHAMA

>CF317_000182-T1 CF317_000182

MTPHSGPPSSVSRERSRLPHRRERTSPVTPVKSQEGAKQTNTKPGNDAPTPPSTIRERRGRRQYLANGDFHPLSVFTKSALFPFHLDTEKAFEILKGDSPEHLEKAKNVFSPEEPQDELKEEKKISTPERCTSSPRKLASQAIMADTLGCSNLDMDLITALKMTSPEKDHTPRVHADFATLCELPIAVPSQHPAFLSSPFKAKTEAPLLFTSTSIDEIRLLNHKNTRRGSSSKLGRESIERKRSFFGLEFKTPSSARSRNAFSSVDDSPIPSSIPTPSTGNTYGSIKGSSPLHTENITPRESASSQSTANSVRKFSDLFRRKDSIERNLDALPWTPRSSQDSPHLARDDLERYLRTTTSTPTYDHEPTSPFPRHPNSARGWKSRNLKCTTCNEAPCAVCQRTCCAFRAAVLALENHPEGSPGRIGALQRIQEITKVFPYGREVPTFFSAPKEMVYQGLVGVGGWSALTAAVFVQTSFVVTRFVENASLTCGRSVNGTERILLPWRIINDFGQSGVKLILKVCLGEMEYSPLHLL

>CF317_000183-T1 CF317_000183

MTTSNLSMTRLLTLAAGLRLVEAFWRMDCGVIQLGRVDPIVSPGTVSGHVHIVAGPNNFNVSSDYDSLQSSACTSCTVQADKSAYWTPYMYYQKADSSFVNVPQGGLIIYYLGRGEGNVTSFPPGFKMVSGNPSLRAYDDTTMTYGDATNPPRPVADRASFNCINYATPAPETPGFPAIMDCPQGLRAQIQFPSCWDGVNLYKTDGSHVAYLSQIDNGICPPTHPVLLPHLFYEAYYSIDQVDASDGGSFMLANGDTTGYSYHGDFMNGWDPDVQADAVENCLASTGSGTIDDCPVLQANNDPQSGSNCPQQPALVDEQVTGVLSALPGCNTPSAGPAPVVGQVCPGKLVAASTALEGTRQVPTPGQTLATLGSNSATYKGCYVDSTSARALSGASYADANDMTTETCGTFCLSKGFSVFGTEYAGECYCGDSIATANMTQSDCSMACNGDLLSYCGGPDRLSVWTIADVASTNTAPSERTTSTSMTTSATTIAGPTVSGAEYLGCYSDTVSSRTLTGYYSNSGSQTLDTCAAAAIANNYQYFGVEYGGECFAGNTIASSASDGASCNMKCSGNGSQDCGGPNAISMFRNKAFVVASNEQLVSVSNTSATASYNYAGCYTDAAGTQRTLTDYWFADASMTVEMCLTTCFSRGFSWAGTEYAAECYCGSDGISNGATVAPGGDGDCSMRCAGQPSEWCGAGNRITVYQKAST

>CF317_000184-T1 CF317_000184

MRTYDDTFSGTKIYPGKGKLYVRGDSKIFRFQNGKTESLFLQRKNPRRIAWTTLYRRQHRKGISEEVAKKRSRRTVKHQRAIVGASLDVIKERRTMRPEARAAARQAAIKEGKEKKATAESKKKAEKAKSASKAAQGQAPKVSKMQAKGAPSKVAAKSR

>CF317_000185-T1 CF317_000185

MSASRCLRCALEAVRPAKRINIQSRSFIQSRTYANATDEKAPRAAGSETVRTNTARDTVLDEKVERIKAQPKRPAPRAPEPKSPARTPAEEHPLPTSEQSQEPASHDPKSAVPLPPNVLAAFRTPYRHKAEHGIPVASLQLRSFSVRNLEFMANFAMRVAYYLKLPASGPTPLPKRIERWTMPRSNFVHKKSQENFERITMKRMITVYDGAPEVVEVWLAALRKWQFYGVGMKCNVWHFEGLDVSSRMDKQFQDVEKELDAKLANFGWNNSVAPNKSIQEMLLRQGARVAGTPMMEVRDDIKDAKNIDLFRDVK

>CF317_000186-T1 CF317_000186

MSFLLSPMRRQAPTTFTRAFSTTRPHALARMTIVGRLGTDPEISESTRGNQVIKYVVGSSYGPKDNKQTSWFRVASFAPHGSPSRDYLMGLQKGTLVYLEGDATMRTYEDADGKKQSSLSLVQTKVEVLKRPQSSTDNAAEGGALGV

>CF317_000187-T1 CF317_000187

MSDPKNIAPAPTEGDLDTGRRSAATTNIQTAAPSPTESDADVSSTVHPNNKIALLCTLHPTTQDARSKVLSLLDRAGSYYRSPKSQCTTWTYFTPSARAKAPSQLIPKDKHDRVIGGMEIYSSKDALSTQQHEAWFQDFHAQVTAEGLYEKDEDLVVWYPAAGFVSRGDSAAPFGNGTIVMQAVFTCKDGQREKVLGVLGEYASSFVAKNEPDVLTYCFMTRPKAPNEILVFERYKDTKALAAHGSAKEFKAMFKAIAPHIEVKQTKLQEWTELDNAFVGNAVGQGEKAKL

>CF317_000188-T1 CF317_000188

MATPASNIDAKLEKLNVQETHSGDLRTKLKGVVDPRYNGEVAHFRGIPFATIAQRFAKPKLTKTFPGDATEYTKFGPRCPQIDFDIRDFMQIPKTIAKDEPQAEDELQWKPIILVTIHYRLNIFSFGNGEGEVNLALQDQQAALKWVKTHIVKFGGDPSNITVGGESAGAIYTHALLASGAEFERAILQSGCLYTSPPQSDKAGFGLAGLIDGNLQLMEFDKGNTSGSFGPEASVSGASVEALVQSLRDLQITRFWLWDEPYFKDWDNDERVFGKLKGLLIGDTQHEWVLWHGAFRKLTAKKVVQCFATKSNPAVGAKLAKIYGIDGLEEDHDTARREALHFLADVRFCAWPPAITKNVNGAGGQTKAYQYCYDEGNPFQEKHRSKAGHGVDLFGLFGGYDVDVNDETRRVGRMMRTKWIDFINGEEPWATNETYVFGPEGMTGAIDRDSNAAVKDLNPRRRQAEISAIREAGWQAVMEIWQNLGSAAASAAAEG

>CF317_000189-T1 CF317_000189

MANTLTAPPWVQETWSAPNYTDPDTKGKSLEHVAIAFSVFGTAVVLLRCYSRVFLTRAFGLDDAFIIVALLSCIALSVLLVLGIEKWYLGYHIWDLPAESVIDHRRGMWIATFINLWASCCIRISILLFYRRLSISFTRGFLIAVWLGIAYNVCMIVVFALANAFICLPTQAFWLRYDIAWRTSHHWKCGNEGISLPTAGIATVIADIYCTILPLALVMTLKIPQRQKFALFFLFGLGFLACGSGIVRVTYLCWTVWRSWDFTWTVYDMWVWQVVELFLGIFVACAPSLKPLARKCLGSRILGYTYTYGSERRSTARHGSNAGRDGRATVTSTIGSKKWSASGSEWKSIGTTATVSSVVGDQEKELESSRTSIHRPVSLLSTSGSLEIGRASSESFPQRRSRLIKTIPLGGPIIEEQSAQPAFQEVGVDPPIYPVAALLRPNPSRVSQLAVGGLGTAHDVRIMREARRPELLQTQQDSYGCVSDSSPLPRSNMYLSKDGWVELKPAKKTHDWGSAESLGGSVTAARMRAGARNESRILQPSASEPQAMSKPYYDAESSHTATFVSTPLPRLSHESAIPSDDRTRLGHGDSSESTLVTDLTQFHFDLSTLGSHSSFTALPQLKREP

>CF317_000190-T1 CF317_000190

MASIEQLNQGVNVLGNVTEAQKKILTPEATGFIALLHRSFNQTRKSLLQRREVRQQELDKTGILDFLPETKHIRDNKTWRGAPPAPGLVDRRVEITGPTDRKMVVNALNSNVWTYMADFEDSSAPTWENMINGQVNLYDAIRKQVDFKQGEKEYKLRTDRVLPTLIARARGWHLEEKHFTVDGEAISGSLFDFGLYFFHNAHELVKTGTGPYFYLPKMESHLEARLWNDVFCLAQDYIGMPRGTIRGTVLIETITAAFEMDEIIYELREHSSGLNCGRWDYIFSVIKKFRQSPAFVLPDRSAVTMTSPFMDAYVRLLIKTCHARGVHAMGGMAAQIPIKDNKEANDKAMDSVRQDKLREVRAGHDGTWVAHPVLASIASEVFNKHMPTPNQIWNRREDYQVSGNDLLNMNVPGGITEEGIRKNLNIGLGYMEGWLKGIGCVPINYLMEDAATAEVSRSQLWQWCKHGVKTNEGKVVDKDYALKLLREQTEELQKNAPKGNKYQLASRYFESQVTGEDYADFLTSLLYNEITSVGPAKPAAKL

>CF317_000191-T1 CF317_000191

MLPQASLVAGTMLFAACAAPAPQPIPVEAAADTSYTTTRTVSATFTFQVTRTVVTTMPEITTTMTTTSSSSYKPTYTATEVYAFKPESPIHLLPFQAKGSIFRLADSPGIYCPSFVQQDGGCSMDSNFTGINGCSLSATVPGGQAVYLAPDGQLRFTQAHSQALYPPNSTICPLGFDTVQDSDAIAVSVHGFDSSGFMACPMLERTLAGIKQVWQIYANVTEMRPPLLKTNSSSCVNFEAVAFVKQLGHAAAAWQYI

>CF317_000192-T1 CF317_000192

MTNEMTSKITFEKPALHQWKRVNLMGVDSLASGSETNSLGPAVNVSDCDVPDAAMTAESFEPSQMAVHAYNFAKYCASLKPTHILIERQRFRSSGHSAVQEWSLRVGMLEAMLYATIRTLTAEGLLTATTVEPMLPMRVNKYWFKDQDIPATGKQAKLAKIAIVSDMLQALNTDAAPFSAGTDVVGIVESFGVKPLQGSAANKVTRSLNKLDDMSDALLQGLAWLEWQQNRLKLIDEGPDGLGIGD

>CF317_000193-T1 CF317_000193

MSSVLANHGPDQDTYPLARAQGLVLLSNTPKGRGVFATQSISKGTVIDVSPVLILTPDEVSNHTSQTVLQHYTYYWPNPSDPKGPQTQAIALGLGSMFNHSRLRQNVVWSRDIAAETITYAAHRDIQAGEELCISYGSARLWFQDADLEDESLGVQGQDELQQSGLHALALVESNNSV

>CF317_000194-T1 CF317_000194

MQLRTLLLAAVSVTSVLAGTINLKAPKRNKVNLIITRESDDLCCTQGIEDLKDLGACHKCRPWQKLDTSTSSITSDSHAPVITPSVKARDVSMMVDPSRHPYPQLNTTGTPSNTRPGLVFPLCLEGGNVVPARPFPEPWCSNANKHFQCLFCSHASQPPSSANNSLDGDIFEFEVVSDGKSDVTNDMQAKKCCLTFRGFTWCYKCPSATSATEVSLAAATAATVAVRDSDGIKPAQCCFTNGLEVFGCHDCEETPTQCCSTNGLTVFDCHDCDKKKARSVSNEGRAVSKLVTTKVSATATKPSPTLRFPTTLTNLPQVTPSTATTLEKPNDQEECCVHLGEELICHHCPTGVADVTDKYLHHITPLLTPPPILTVYSQAPSTTLSVAKRQQTYDFHMECSWASAEQGHKCNKNKKQKINADMCKSMCQCDNLGSMHCQAPDNDCTDDETLDYCKKGSPFFGCACSHL

>CF317_000195-T1 CF317_000195

MPLAKSKNTVGLGNSLMNDRFGRGKGSDRKKVSSDKGGVVRVNKATGEEYVTNAAKEADWVKMRSITEQNDLDEFLTNAELAGTDFTAEKTNNVKIIHTDQRNPYLLSSAEERGVRRRQDKQRNRLTVPRRPKWDENTTPRELDEKERASLLEWRRGLAELQENQDLLMTPFERNIEVWRQLWRVIERSDLIVQIVDARNPLMFRSDDLDKYVKEVDSKKRNLLLVNKADMMTEDQRSAWADYFMEKNINYKFFSAHLAKELNEGRDAEEELAERELEQTVNQDKPQKSMLDNVQDLNLEEQEEELDDEDDLSADETESTDDDTSDASPTGNPYLQQPDHAQDEKTRILTVEELEALFLENIPIATDSKDGEPVRKTTIGLVGYPNVGKSSTINSLIGSKKVSVSATPGKTKHFQTIHLSDKVILCDCPGLVFPNFATTKAELVINGVMPIDQLREHTGPAGLVAHRIPQPFLENVYGMKITTRPVEEGGSGQPTGSEMLRAYAKARGFAGTGRHGQPDESRAARTVLKDYVKGKLLYCHPPPHDPEIDGKEFNKGLYDFAHLPAKRQAWLISHGDEAEAAADEAPSTLSAPKAATGQKSKKVDANFFAPDPRNAGHIKSRAFSHQYTEQGQADLQQKSLTGRKEKMMRALELGIDPKEVVKDKQHFKRRMKTRKAKAADDDD

>CF317_000196-T1 CF317_000196

MPRRKFIDKNNPTTQTFQLLYRSQNDPLVNDEDAGDRALFPVGRGKNAPTPSSSSSASSNAAQDRALHLADLEEGDDLDFDSMRENEGEAAEYGVYYDDTTYDYMQHLRHLNQGGGESYFVDALPTKVKGKKNAKGKQAMRLEDALAQVDIHDDEDSVAPSLIDASSSTFSRTTKKRMLEAQQDVPDEIAGFQPDMDPRLREVLEALEDDAYVDDKDDGHVFAELGLDGVNNGELDLDEFEAEADMFADDDGWESDATEKAEVQVMQSELKMPPPTDAPGEPRTLDAGEQLDQAAAATASAEDGDWLRDFAKYKRDAAMSKKAAPVQAGSIAASGVQHRAPSLYTLNGTPLRQKKRKGAQTNPSAYSMTSSSLARGPGLELLDRRFDQVEKMYGIDEGDEFDDAMDGGASMLSGMTGASRVSKMSALSTASFADPGAVRSDFNGMVDDFLGGWDKANPGGKRKGAKGKRGKNGNEVFGLQQLEEVRRELGPARVRRTVQKG

>CF317_000197-T1 CF317_000197

MSTILRTLRNLRKIGFKEYGHQMQYMGDTKAGVLVGKDRYGNKYFENLEQDLPLRTRWVDYKDKELDASQIDPGWHAWMSYMVDKPPSEDPLMQFGTRPWENPETVKNVTLSRGAYKPYSTVKPKLQAWTPVAKPRDPSVPFDLPKAVEGPPSGLKQQQAESSL

>CF317_000198-T1 CF317_000198

MKDRQIQFPGLANPARAPATAHDIAAAWAARSTRQLSVSDILATCLSLARGAIRNEFSDEFRYVVKEAIEADISTRCKKLCMAEGSATILKKLPVPRYFKNFEALVEYVSDVFICIENNKSYHVLRKSTLSNTQMPLLRSSRRHARVALIRDELTRQLAAAYQTHTLLHDFDAYLKIYGPPVLNFSVPDLSESVAEPQEEKMISTFSEQWRYQSQTTSEILHDINAVYQDLEAKGEHIRRAIFFTAFVALINIERYRSVLALLHELYISRWSNQVFNRQIYTLWVRTAMTVSNPLALREALWAIVDSPSSIELPARFLVLVRLAQEDLIYYHGLGWRNITQEQHDEIEYLKKRIYRRKWYQMGCPDAVDIEEPHLREWAQGKTHATFYPMSNEVPANTEGRISKVQTGGDAIEPHTLGEDTVSASEHDMQLCIKSETKSTNNVSAESRAVAGSFHGIVDQQSQPIGDAYLVGVPTSVAGPEEPLPEKGTEARSSIALTARRRFDQPSAALEDNALVKGNEWPSSTDTRFETALDSAPGSHDRKRRMVRRVRPSDRDA

>CF317_000199-T1 CF317_000199

MGSDKSNHSPSESHCPPASKPNTTKPKHLDLPTTTPITSGASRTTPGQLLPAPALFHGPHSRNASNLSLTQRSRDRDDKPTGTGTGTPAGEPDQHLPRARPPTSSFSGGSRSSLIADHALPRSSHKKPPNSLGAGIAIRSTQQQRRHDDEFRADAVWAEMQKTLADVELSAMNSSHVFSQEHVAALEDLRVAQLGLARAWAREEAEEQADEEFGRDVEGGAAADNNAEVKGGMFSRGHEAAGADAGSPKNEKRRRRHSRRESSVSVMGETLEEETERDIKSGRTRREANDRYFKQVNSGVVDVVKRLDEVVEAMRRIEKESREIWNSSDSGGGSGDDSEDLGAHTETDESLRHKTHGGGGGGGRKRAGTADTGTATIESDVFSDSPGTVATRER

>CF317_000200-T1 CF317_000200

MTNRGKPRGLNAARKLVQARKDGRWADLHYKKRLLGTAYKSSPFGGSSHAKGIVLEKVGVEAKQPNSAIRKCVRVQLIKNGKKVTAFVPNDGCLNFVDENDEVLLAGFGRKGKAKGDIPGVRFKVVKVSGVGLSALWKEKKEKPRS

>CF317_000201-T1 CF317_000201

MDDELLYPKGLREIITSGSIVRDPYPSYSDFVGSFVPSLPDMSNSSFQNEYEEEFHTLVQHMLDYDFDAYRMQQKYLMKQAIAWNQLWAETRCNQTREQEDRENRKIIAALERDDILW

>CF317_000202-T1 CF317_000202

MSSPASNNKRKRGTASDRVKAGSSGELQQASSRDASGEDMAASSPAANTRHRKQASQDQTAAPPSKRARTRSSASMKLPKVVAQDVDDETEDQDAEEPSRATKDELDAKSKSKRRGSHASSTDSSQMDTGDAEGERELDDTEKKMDAPPRAGQRDPIGGYKTNPPPTGRPVRIYADGVFDLFHLGHMRQLQQAKSAFPDVHLMVGVTGDAETHRRKGLTVLTGQERAETVRHCKWVDEVIPNCPWIVTAEFLHEHNIDYVAHDDEPYGATEGDDIYAAIKKEGKFLVTERTEGVSTTGIITKIVKDYEKYISRQLKRGTSRQELNVSWLKKNELDIKRHVGELRDSIKQNWTTAGGEIGKDLKTFWQHAGINISRPSSPAPSLRRTLSSGGDNGKALDVSGAKSPTTLEHMKHLEIPRAQSPGFSLRGRSDDFAAGYSLGLLGSVRSWMSRSRRSLRDPEHNSPEGTDEEDMRSPVSEHGDPVSPVVSRGRQGKSGVQKGDSDAMDVMH

>CF317_000203-T1 CF317_000203

MPLLTFPINAQLPAVAVADEDFSFTFSADTFASDLSINYTLVNGPSWLQLDDTSRTLTGKPSSKDVGETGFQIVATDTTGAANSDSRLVVLEQNSLRMRQDVFIEGLSQAGKYSAPSTLLFYPQSAFHVIFGPDVFEGDHASIQYYASSGNNTPLPAWVAFDSAAVAFIGTTPPLLTPQSSPQSFAFNLAASEIAGFSQSTLTFQISVTNHVLAFLAPTQQVAVTPGQPVTIPPLLGQLQLDGSPLDKSKITNVTANQPLWLQLDSQDLSFSGLSPENLENTSFKLAVADDQNNLASAEIRLLVASQEDNSTTEVFLGTVNATIGEHFEYKFVDAKTDSSVRKVDVDLWAEPWLSFSQTNLTLQGVVPSDTVEGDFNVTLTISEDGQVTVNNHLTIKLVSKNVMTAPLIPNQSATRSSVMPTSTTAPPNGKAAQGFDDRTRKLALIIVLPILALLALCLLAFILWKRRRPRDGAHQAASVPPPMQQSSRPPSMGSTMELTELEARSLSEASFQQLPSSPPPRVDLPWHLRDKPRIKLLSAVDEYDRDSPETRSSWDEMLMEVDRPVSDKGEDDCVLWGLSAMQGWRISMEDAHAAVLDLQPEVEGKDQTAAAPDKRMAYFGVYDGHGGEKVAQFAGENIHKIVAKQDAFAKGDIEQALKDGFLATDRAILNDSRYEEEVSGCTASVGIVSKEKIWVANAGDSRTVLGVKGRAKPLSFDHKPQNEGEKARISAAGGFVDFGRVNGNLALSRAIGDFEFKKSADLAPEQQIVTAFPDVVTHDISEDDEFLVIACDGIWDCQSSQAVVEFVRRGIVAKQELHSICENMMDNCLASNSETGGVGCDNMTMIVIGLRQGKTKEEWYDMIAKRVADGDGPCAPPEYGKSDEDKSDHPDDYELDVPRYQSGRNDDAEMFDHSMEDEQDEESQMKKGASATGEDDSARKDREGTPGPQSEAEKQHMKHSSVSQGPGTSIQGIADDPDNNK

>CF317_000204-T1 CF317_000204

MPSKENKREKAKEVVDILEEISILLDTDLDRAQLSLCVSLIENGVNPEALANAIKELRKEVRNNEKLLHETGPESSMSD

>CF317_000205-T1 CF317_000205

MSSPPGSSSDDYARHEKDIENNKTGIHVDEQAASRPANVTPAREIIRARQIQSTVAPFRFLSKGEQWLDRKLGIETQGIDRIPEEEKRPPSIWNVFLLWWGATCHIGTVPIGALGPTFGLPLGESIAAIVVGTWLGAICPAFCGTLGPKLGMRSIATSRYSFGFYGAKLCSVLSVIVAGGFGVVNIVITGQLLSAVSDYTMSVSVGIVIVTVVSYLISVFGFRYVHTFLKYCWVISFILLLVLVAQAAPHAEAGLPGFATGKAHAGLWLSMFSIAFSSTAGWASFSADYYCNYPAKTRSWKIFCLTQFGATTPILFASIVGAVIANAAELGQVEPWYSTDNTYGLGGLIRETYHPLGWSKFCLVMLTFSVLGNNILIFYSSGLDLQLLGHYFHAVPRFIWSFLVAVVIAILAIAGKANLSTIISNFVSMLGYWAICFTLILFIEDQFFRKRIGYNLEAWDTPSKLPWGFAAVFSLLAGYLGGGLQGMAQVWYMGVVARKFGPYGGDVGIYLTAAVTLVVYPITRYLELKFTGR

>CF317_000206-T1 CF317_000206

MVGVSRHDMNDLSGSRGEANFVWRCKNCKRESSATIKAAPVSYEGGDVPKAKNIIEIDTRGLEFTEFRADGEWEAVGSDSGTKFTGIDLSEGEWFDYDEKAGDEVSIKDIQWQIRRA

>CF317_000207-T1 CF317_000207

MSFTPVNPRPLLQSLINEEVIVRLKWGQTEYKGVLVSVDSYMNIQLNNTEEYIDRKQTGTLGQVLIRCNNVLWVSAAKGVEMNGAAGDVKMEG

>CF317_000209-T1 CF317_000209

MTVCFYLGREFNFYTQIGYWIKGETPVVISAKEIGSLNHFNSLKPLLLGQPTMPKAKEVDNKDTSFGKKGTR

>CF317_000210-T1 CF317_000210

MESLNNETFDVVIAGTSLSQSLLALALSKCDVKVLHVDKNDYYGGIDAGLSLSEAQKWADAREDAAIIEVNDTNSGLGPSRSYTVSLKPQIIYAKSRFLPTLVSSQIHTQLEFQAVGSFWVLADGNLKKIPSNREDVFADDTIPVRDKRRLMGLLRYVVEEQDEGADTSGLPSTLEAKLIDQFKISPSLMPPVQALTLSTNRLRNTSFDHAMTCMRRHLLSMGYFGPGLAAVMAKYGSNSEIAQVACRAGAVGGFVYLLGHSMGSINLPAETDSLVEVELFDGTAVKTKHVVGMQGDLPPTIISSPNQFPDHAATSRVVHRISIVSNPLRHLFAAPENASVPAVTIVLVDAGDLDLPPVYLQVHSSDTGECPEGQCIIYASTQHCGGDGQERLASAINNILTTLEESQPPAQILWTMGFTLDEAAGPTGWNHYIRDTANRVIVLPERTHDLAFDDTILDNVKEAWEAILGERRTEYDFLRFDEHRGMNEDD

>CF317_000211-T1 CF317_000211

MKLLSLLTTSLLTVLTSAQLSGSVGPTTDLASKQAKKTCNITSYGAKSGTDVGSALLAAWKDCKAGGVVVVPSGTWTIATWVLLNGGSGWALQLDGTIIRDTSATAGGNMIFIEHASDVEVFSSTGAGAILGNGYEMHKSGSISGPRLLRFYDVDGFSVHDIKLVDSPSFHFSMDTCKNGEVYNMLIRGGDHGGLDGIDVWSDNIYIHDIMVTNKDECVTVKSPAHNILVENIYCNWSGGSALGSLGASVDISSIHYRNVYTVSSNQMLMIKSNGGSGHATDLLFENFIGHSNAYSLDIDQYWSSMSTVAGAGVALNNVTFRNWKGTESNGMSRGPIKILCADGAPCDDVVLEDFAMWTESGSKQKLRCKVLGFEPTSLGKTTTAKTDLPKASTLELTTADRVSDEGLKDK

>CF317_000212-T1 CF317_000212

MEAIGFEVDCIDQSNHTDIAIPAITAYNADGDTSAWTDLPIFNSTFALEYASESVNYSKIVLDLHYFQSDDPYNSKSDTCSGTVFKKPCSLRPAIAAYPLKVSNFTNEHIINGVSLATKTSTNDDTDMNKSTPPPKYNATLKQAEGYAVIKYLYPQDTHTIRSLTALGGIANALSQFLSSSAAITYQADGSWSLKQVGTLAQTMMYGPPNMGSCDCSFRNEALDSIIASINQLTFLVATGMIDTAGFKGLPRGKQAFPSPLPEASVVAVDSNTTTSFRVLSDHAVQLTDVVHFRTHYIYAGLAFSITIVCIILVIPSFWQYGEFGRKVTLGPVEIASAFGAPILVDDARPEARKENIETLIKHTGDPKIVYGFVDVDADRQLDDRQDMHLQDTDQSGLPSSPDLTSPGFQSPMLSPALSQTDGQTSGVQKRRSVRLAMGAPERVRPASEVFPPRSPRLGEVRE

>CF317_000213-T1 CF317_000213

MEKPNANTNVAVSVVDSEREYHDVQPVSKWLPGYWKRFPFVGAFALACIAALACIALGVLIGSDGVSTSTWPQRIAPNVVLSMINAMSSLALTAAVREGVAIAWWRHTMQGSTVAQLHHQRELSSGLFASITRPKSLFTSSIALAVLATQVTLLNSVLYQRATSTFAAPDRPKGLQSVGIGVEEFPMTGYVVSNTSFGAQTSCSCFMIGDSFTPVVNT

>CF317_000214-T1 CF317_000214

MSAPHTIVVIGASYGGLPVAHALLKDVLPTSGKDYKLVLVNPSEEFYWKVGAPRAITRPEKLSMEKALLNFLPTFEKYGDKFQFIKGKVTAIEPTSKTVDVDTGDKIHYDQVVIASGTYFENDLWSTSNGTDALRQEVRELHNRLPTAQTIMIGGGGPCGVETAGELGEAYGGKKEIILLSGADRLLHKPRNQSPSKLSQQMLEKMGVTVTHKVRVQSMKKEGEKTVITLDNGEVKAVDIYIGAVGDKPNNSFVPKDWLTEKGQIKTNGNTLRVDVPGVTGVYCVGSVASYSDGSILDTKLAYTAAVESVKLDLNGENAGARTKKIYKKIQSEMMFVPVGSQQGVGLAFGWKLPSFAITMFKSKDYMIGNAPKLIEGQA

>CF317_000215-T1 CF317_000215

MGGTRTETDAFGPLEVDSTRYWGAQTQRSLGNFKINQPQDRMPDGVVRAFGILKGAAAKVNMKYGLDPKIGDAIVKAAEEVASLKLIDHFPLVVWQTGSGTQSNMNANEVISNRAIEILGGEMGSKKPVHPNDHVNMSASSNDSFPTVMHIAAVLDIEQMLLPALKNLRDALDQKRQAFDKIIKIGRTHLQDATPLTLGQEFSGYVAQLDRNIERVKSTLPHLRQLAQGGTAVGTGLNTFKGFAEGIAEEVTKMTGTEFITAPNKFEVLAAHDSVVEASGTMNTLACSLFKIAQDIRYLGSGPRCGLGELVLPENEPGSSIMPGKVNPTQCEAMTMVAAQVMGNHVAATVGGLSGQFELNVFKPLMIRNLLHSIRILSDSMNGFVEHLVDGLQADEERIGKLLHESLMLVTCLNPVIGYDMASKVAKNAHKKKLTLKESAMELKALSEEDFDKHVRPELMLAPKEKK

>CF317_000216-T1 CF317_000216

MPLLEDLDSSSLHLLYAAHTLVARNETSNSTSTASNANRPPVYKVIGLSLAIASGVFIGCSFVLKKMGLLKANVKYNEEAGEGYGYLKNAYWWGGMTLMIIGEICNFVAYAFTDAILVTPLGALSVVVTTILSAIFLKERLSFVGKVGCFNCIIGSVVIVLNAPEQSAVADIQEMKSFVIAPGFLSYAGLVILGSAFIALWIGPRYGKKSMFVYLSVCSLIGGLSVVATQGLGAAVVAAADRGNQFNQWFLYVLLVFVVATLLTEIIYLNKALNIFNAALVTPTYYVFFTSTTIITSAILFRGFHGTAVTITTVVMGFTQICSGVVLLQLSKSAKDVPDAAIFKGDLDQVREVAEQEHPESEPKADAIRGAASIIRRLSTPRREMEHQEVKRLREEKQAEHLEPLKENEVAEWDGLRRRKTVIDAGPTASSIVRRGTLHPPLGMTHFPDEDVHDSSQSDHKFLGNVRERAHTIFHIRGRHADDEDLDPAGLQSPDKPVALTNINFKSADADSPALPYGPGSFEEAQEHIYGLPASLKPRPLPSPRSKPLPKSPAPSSAGLKAPDSAKRQFSFSNMFRHNRHSSHSTDEPVRPPTAASAAEKKAKKDATEEERLGLVKGDSSFPLMADSSPERRPAPPLHVTRSDSPEALETASLYGNHPYTSTHKHHRSASPDAMSDNDIQSDDDSRQAPNPVYQRWPAQPFSFSSSDPPAEAHQSPQRPQSRNRPKPPAVYTPPPAMPMTHTQDFAQSSERLPQPPSLAPPSSLGPRRITIGSVSPHSQSQEQVPQLPHVRLPPSLEPPVQFNPTNRRTPRDLSPVPSGHSQVDPTLSETSLPSSVMARARYTSALTGDDSSSSTSRDDSNTASRERFVEQSARERAERRDRAKHRSFEPGHNVDLS

>CF317_000217-T1 CF317_000217

MDSHNVKRKDTTKGPPLRVLSLDGGGVRGYSMLIILQELMYRAYVETEGKPPKRDEIPKPCEYFDMIAGTGTGGLIAIMLGRLRLDIETCMDVYVRMTKKVFETDKTIAGIPYKQTLFKASKLEEAIKQCVAEHTVYEDEGNDGTQRGGKPFGSSVYSPTSPMARSVSVRSNGSLAPTSPAGTNRTSMYSPGPGFGASPTIWGNPEASLYDDRENRTKTAVTAVYKGTNTKTGSAILLRSYDSRKEPPPEFNCTIWQAGRATSATGLAFKPIQIGQSVFIDEGAGRYNPSPQILEEAVVNEYPGRDLGVFVSIGTGKRPPGTNSSQQDWWEGFIGGSVGSFAEARRRLIAKIEACEDTHIQMLNQELPRFQVARENYARLNVEVGVGEFGMNEWDRLSEMTIGTRRYLKKEDTQEIIQNAAYKLAKIHLMHRRMTQHMRPVSWQEPQQNLDYQVNQPQQQNDWPAAVELPADEPTGHSPSFRPQNTQPQIHFRTPSNPPYPDENSHSSRRDSSDKFAVINPGTHNPSSRLSYDSGRYSNTNEKFSITSSDYQTAPSSDAPPRPPKTPINEPQSMVSPMSPVRTSPMFRPSGGTRLPYPVDDEGPPPVNKLRKPQYMPS

>CF317_000218-T1 CF317_000218

MEDSSDPHFKRERPAAYGPPPPLHMPPTDAPHLHNYHHPAPQSAQMPQPWNPAPSPYHDGSDHRPPPTDVPPQHHPSYPPPQNYPPPPPPSHTPTYTPDTAYSRQNSASGPPRSPPSAPPHYPQAVNGGPAPDGYYPRQDYGNRPPYAPSESPVNASQPSLHVQTTSHEMMPAQAQAQGPPPGYPPPSHSAGPPPTPYWGPPPGDWYQQQRRKPVRAAQACDSCRQRKAKCDEGRPECTHCKENGLRCTYRDVPPQKSEKQMLQMGEKVDALGDKLESHIKSTDDKLNSLLSTVQSLVDKRSLDSRNSRSVPMTRTTSRPQTKEEPRSDPSLNFWGHQTINAPFMQKSTNHTMQQNNTMDDTTQEEKKKKKRTDASALLEWTAISELVPKDIPVSYVLDGEAERGLLRLYGCGEGEDKGDGHEGAVASPAASSSSGRMEEDVSTSSPSGVWGTGQLPHREDSLHSAHDHAGGVTPNGELLLDKKAVDSYVKSYLDRMYILHPFLDKKVLRKMVSAFKKKYSWDYGNNINHLAHNVGGGTKRKRESSQSPHSTTDDLHTIPQYDSVRPSLRNHPPIEHSVSNAIILLVMALGKICGYEYALPGPPKSSSIATNAKTTMQQPHLGYGDLPHLGRPPSSAPPSPYTSHMKGMPAAPVISNLHGKNIDVIPGLAYFAKAADILGELPGGVDVSHVQANLLAGLYMGQLARVIPSHWYIANACRACMILIESPDYQEKKMTRERRNLINFAFWSCLQLESDILAEVDLPPSNIVAKEGDMLFEVPHDLTVADPEQEGYVPGTKDPTADHYSNQIQLRRTINDAVSHLYNSSRESEKLSSTIIPLLTENLEAWRVWVGGTFGWDWPEENHESPDINQARMRGKYYGAKYIINRPALHYALNVAGPSTPMSRPSESPVGSGAVSEYTSPALTHHGDPAASRRVNEMRPPPRTTDQHLEPWILEACTKCVDAAIKSTTAFDKIPGRLVITNILGTAHAQFGNVLVLAATFNSPNPQLRSLVPEEKLRGLLVRTINFLDDKASISPALGKDAQILRHVQNKLFPSSGHTRFYPASANSSFSSNH

>CF317_000219-T1 CF317_000219

MTEQVIPPSLRAIAAEVAQLLKERKETISVAETAAGGLISAALLSTPGASGIYKGGLTLYTLQSRIQFAGWTQASIDGYKGPTPEIVAGLAENIRAKLESTYCVSESGTAGPTGGSTPNRTPGYVALAVASEHGTKKTELDTGKGTDREQNMLAFAIHALRLVKEVVAGQSKM

>CF317_000220-T1 CF317_000220

MMGGVVAAVQFTGHITSMDTQKKERGRAKISLLIYLVHPICIEPPRQIRYSGFDSVVPEERQIRAFNGGDMAPIGFKAAADKLFSVLRKAERFLDTFLRSFKQETAAAYMDRTTEWKRKVDRYNTDLVLPDLKVDGSNSDYRGDDETALASDRDTFDELQAQIQKHVQQLSWSRLEKPASSNHHASHDRQAGTGHDFHLPDDASIKLEVQYEIAQALLRSVETVQPELIRHVKRMDQDYVAAWEALSTMKRLRRVLDQYKRGWQDGEDDSLEG

>CF317_000221-T1 CF317_000221

MAQIGMYYYVLSRLTAAIHERSQLSASEKHIVGETLIALDNTKGCDLQGDDIVQEEGGDHDHGSWQQAAPLPKEADLIIWWNNTSQVLNARWQRTVVLRR

>CF317_000222-T1 CF317_000222

MHQAHQSSFGRRVSSWICDSCQSRLARGQKATATASATASAARAFSTSSKRVASASSSHASNSNASPTRRLKVPDAPARTRFAPSPTGNLHLGSIRTALFNYLLARRTGGQFLLRIEDTDQKRTVPGAEEGLFRDLRWAGLHWDEGPEVGGAYGPYRQSDRLLLYQSHIQTLLDNRKAYRCFCSADRIDKLNRLRHEKGLPLGYDRKCIDIPTHQAEDRAANGESHVVRFHVPKDYPKYNDLVYGTSGHGAGKSKQHLADEPVFDDPVLIKSDKFPTYHFANVVDDKLMRVTHVIRGSEWMSSTPLHVALYNAFDWSPPQYGHVPLLVDANKQKLSKRNFDSDISSFRDKQGIFPETLTNFAALLGWSHQRKNDVMDLAELEKTFDLKITKGNTMVAFEKLNFLQEAHARRRIEAGGEPFEHMVRHVALQILNTYGAALVTRFIGKRKLRDIVADFLHVRSLPYRNSQQFVESLVIFLSKDGIKKRQPLHLAVPDVALYPRLRVAATTLFYIPEAHWNRETLRYHLSQLHLTDDLLETPEAKVQAKAGTKALYAFLRWALLGVESSPDAPTTMEILGREVCQSRIQEAVLVAKEVENQKTTPKVQARRVPKGEGNSKWQAHTLPSAAASNS

>CF317_000223-T1 CF317_000223

MNGMPGMNGGSAADEIDLYDILKVDKSASKAEIKKAYHKAALANHPDKVPEDQREEAEARFKQSSQAYEILSDDQKRGLYDAHGMAAFEGGGPGMGAGVNMEDILGGLFGMNMGGGMGGMGGRPQRPKRSPDENQKYEVSLEDLYKGKTVRFSSTKNVLCSKCSGSGAKEGVQPRECSTCKGNGVRQVVQQVGPGMLTQRLVECSACEATGKVVNPKDKCKKCKGKRTTEEKKQLELYIPRGAKEGDKIVLEGEADQIPGAEQTGDIIFHLVEQPHDIFNRAGPDLQATLEVTLAEALTGFERTVITHLDGRGIQLTHPQVEGEIMRPGQVIKVKGEGMPYKKSDAKGDLYLIVDIQFPEDGFFNQDSATALRKLLPQPEPAIKTDVVDEATWEDADPEEFGKGDPRGGGQWEDEDEGEGPQCATQ

>CF317_000224-T1 CF317_000224

MPSTDRTATTSLPHPDPCTSYWQQPLDEIADLRTTPELPQHAEVVIVGSGITGSSIAYNLLSAKPDLNIVLLEARQAASGASGRNGGHTKTASYRSFLDNIKAVGEEEAIKVSKLEYDCMAAVHDLVKQKGIPCDAKRCDTVDIYYDQGHLDQARESIALMEKLIPHHPTSKHTFYSPEETKKNFFAENSLGSLRYQAGSLSAYKLTIGILKLALQLRLNLQTNTPATSISKSSLAPDQTTWTVTTPRGNITTPTLIIATNGYTAHLLPQLQQVIVPFRGVVTAQRPGQSLPHGGDLPTTYSFIYKEGYEYMITRPSIRGPSDHTAHQHPSPTPPQDSTHDIIIGGGLTKTPSRGTSEFHTTDDSHPSIPASITSYLATSTRTFFGPSWDPPHALGTTRTVWSGIMGYSADGHPLVGAYPGAPGLFLAASFQGHGMVLCWLCARALSSMVLGREEEDRLADWFPRCFRVSAERLGKRFDGRVVGREVGEAESVNGFDGGAEV

>CF317_000225-T1 CF317_000225

MSVATKRYVSAYEDGRHTNEMTNPSPQHYSSHSHSQSQRPATNSATPQAQAQNQNQNPLKRQASQDEEDRPTSKRQKKEDAPEAEFMRLDSSFTSTNHDRQKEQRPNEQSLTQAEPGKAEAHLDPIFTNVGKSFRIGRTTHKRGPVDINQNLLGKYGLHDLLARCARQDPDTGEKINKLRSSYAGQIKDAQLPGRNDHPRVVREEDQPSKLRTMATIPDDEFTPRLADRKIGDLNGLQGLIKSAMHLEPGNMNKHTMHEWDIILGHEPQKAVKPQQQQPAYAQLQHSRLSNGLRQSQPQPAPQADKMRTRGKKRSYGDTAYVGYGDGLSEVEDEQDRSDDYVDNRNKKRR

>CF317_000226-T1 CF317_000226

MTTICHPTEDVAKGTRPPEERTLLTSTDYYPISPTTAPPQSLNHTPSPQCHPRPPHAHPHSPQQTLSSFDIYGTLIDR

>CF317_000227-T1 CF317_000227

MINATQVVVQLLYAAVSFAAVRWLWEAFFSPLRAFDGPSLAKVTDIWRAVATARYNVDVTHRRLHQQYGSAVRIGPNCISISDPNLIRTIYATKNPWMKSDMYRPNDVLINGQLLSNVFNTQDENWHSKYMRPIRGFWTMTKVLDYEPLIDETLVKFLGRLGSDFAEGNNTGKSCPGDEWLSYFAWDVTANISFGRHYGFIDQGKDVNNLIVDSTKGLYYFAPVSQIPWIDRLLDKNPIVRIGPKPTLTGVMYAFQVVAQYQAELAEGKDRPPVANSLDRYLRLKEEYPDIVNDAQVVNWLMLNVLAGGDTTSATMRAVVYYLSKNPKAYEKLTQELDSAKLSFPAQWKEIKDLPYLDAVMREALRINPGIAMIFERVVPEGGFSLPDGRFIPAGTKVGINPAVTNRDSEVFGDDANEFNPDRWLPREGELPQAFDIRLKRMREVADFVFGGGGRICMGRYLATLEIYKLFATLYSTFDMRLADPKHEWTYRNAWFVYQYDMPCIVKRRQKSVVA

>CF317_000228-T1 CF317_000228

MATTSATPLSAEYLARDDSGKLIAVMWTATILPLIFVCMRVYARVFIRKTFGWDDGIAIAGLACLMAYASVVTAAARQGLGQHIQVVLANDPQHLIDIALLAQVAQVLAIMACTLGKTAFAVTLLRIVVQRRLIYILWFIIVSMNLVNVLCAIFVFTQCEDPRHLWNQAIPSKCWPTYVFTNISLFVGAYSGAQDFVLALLPWFVIMKLQMKTREKVAVALAMSLGIFAGVTAVVKTTFLVNLSKRTDFTIALPPLLMWAAAEDGLALVAGSIPVLRPLYKAIFPGSSAGDSYKNVDSYQLRQPPSNPAIFGVTKGQSHVSAPQRGEEDDESDKSILDKSYSGLSFHNIKKTTDVNVSYAGSQ

>CF317_000229-T1 CF317_000229

MSTLLDSMLNSDAIAGATPSSQPAPTPRDLPSSPRRTPGPPPSISNGFPSEAGLFPDDEVVGAAQGRPRNPLDRNVPKVEDQAASRIQTAFEDFLESHTEEPASSALPPSSELKTDKYYVNQIHGLKEFQLSTLYVDYKHLSAYMEGFLAEAIATQYYRFMPYLTKALHSLLAKYEPAYFREHRQMGSASSQAQGTSLGAHISAPEEGSQSASILNAQTDKVFTLAFYNLPLVSRIRQLRTDAIGKLVSISGTVTRTSEVRPELSAGTFVCLNCNTTVPNVEQIFRYTEPTICPNQTCNNKVGWRLDIANSTFVDWQKCRIQENSSEIPTGSMPRTMDVILRGEQVERTKPGEKCIFTGTLIVVPDVSQLGIPGVRPEASKDNRSFRGAEEGGSGVTGLKALGVRDLTYRMAFLACFSQPDNTTPGVAASQLTGQSTNILNSLHQVDLHDTFESGERAQEAYLETLTMPEIDELRAMVHGDRIYSRLVNSLAPMVYGHEIVKKGLLLQLLGGVSKQTPEGMALRGDINICIVGDPSTSKSQFLKYIASFLPRAVYTSGKASSAAGLTAAVVKDEETGEHTIEAGALMLSDSGTCCIDEFDKMDIADQVAIHEAMEQQTISIAKAGIHATLNARTSILAAANPVGGRYNRKATLRANINMSAPIMSRFDLFFIVLDECNENVDRHLASHIVNLHMFKDDFVQPEFSTEQLQRYIRFARTFKPIFTPTAKRLLVQKYKDLRANDSGGLGRNSYRITVRQLESLIRLSEAIAKANCVEEVTEQMVVEAYNLLRQSIISVERDDVGFEESDDEEGGEDDDADGEAAAVDGEDRDSPMGDTDAARQSSAQPDQAAAGTRARTKISADKYNKMRNIFIKKLADDEERVAKADSERQQRRAERQQAQRDGAGAEADADAEDDDDDADQDQDQDPTAAVGGVERGDLVLHYLESIEDELAGPEDMQREQKVARKVLKRMEKEMEIMVVRANADGTAAPEPGAGVAEDGEGDAHEQGTVVRYVLHPNISLDES

>CF317_000230-T1 CF317_000230

MTTHRVRASSPSAPRIYLDAPRSSTGTILSSTYDTRYDQRPPRASFSSVPSTSAQSSSRHNSVYETAQPVSKRTYHDPAHSGATISRTEYAIRPRMDSNTPENRRPANVISKNASPTRDKFDKLDSPRDLVPYAKSSRDDTTRLRPTYPQASAHHQRRNSATRAESTRHSLGLDAHRSDREYHKRGPYVEKVADSRAPPPAARYAPDPTYEYTGPREQFDRDYPPPRPRRESLTRRERPNSAIGTKFDQVPSRRETNPPPSASRQLQRIERDERKSGFESDPERSREDRPRERPSRHPTKTAVVHQRDDGYSSARDEHDSRRPPRRLHDDDASIASSKSRYHEVDRDAERERERPRRDRDREREEERPREPARDRDRDRRERRSDKPENRDYERERERPRPRDYEVIEEDDSQRRPRRREPRDTRDESPDRGSGLKTLATAALGGLAATGVANFKSRKDDEGSDSDGRKDRKHRRRRSKERKEDDGPVDEDLEERKHRRRRSKERKEDDGPVDEDPEERRRRRRERRAQKDDSSGSDTPDDRKRPRSRTRRRRDTQDGYGSDREKAQAALAAPSADRRPSRDADPHAARGESAREPGPSRALQESPTNNEGRTLSPGEGEDDRPRKVSIVEPNKKEDVKPKGILKPPRSDPFPEDPNPTREGVAPLKQAGKDGIPSGARWTKVSRILVNPEALERYQERFEERDDYVIVLRVLSREEIEKLAEKTREIREAREREWQKQVEERKRRRAERGDRDTEDEDGYDEGSEPRRAEPLALEPPPSEPPVDLRQFAAQNSAPQQPQMAYAPTQQQQSYPPQQGYPPQQGYQSQQMPYEPQRQPQPLPQMQQQQQAQPHYPPGQWSNPTSARNSGEAIANTAPGQPLPSRQDQETYDGSYSQNV

>CF317_000231-T1 CF317_000231

MPKRTEANRKTNIGVARSVPVLILAIIGYASWVLTKLICVDYLISPPLSITARPRRSSAIAILVIYYLLLILLLVSFGRLLDTVVRWPGLIPRGPQWYVERTRQKKNHNGRRSRSTSRSDGEKSAGSDLNDPKMPRETQHLARETLPFKVEDFWLRDVFVCNADGRPSFCSTCYNWKPDRSHHCSEVNRCVLKFDHFCPWVGGVVSETSFKFFVQFTFYAALLTLHILVVTAYFFARRRQDSSFVNGHWIALLAIAGLFFLFSGGMCMSSCQFALINSTTVENLSRKTKVWFLAVYTSPQVLEEAQEKNIGLRLISYPRPPEEQLHMLQQQGAGDVSGADVQRPSSSSAMPSTGHRTFAILESAPGANPFDIGPLSNFQEIMGMTIWEWLSPIKPSPCLKHDSTTSLYKLGPVVDQMKADTGLKSWSSQQSKRSRRRKGRGRSENTAHNDA

>CF317_000232-T1 CF317_000232

MQPHQNQPYQGQPPARQVQQSPNLSNPPSTQQSFAAQNPYAHHTSIQSPYLNQSGHFPPPSQQHQNAQEHPYYSSDPSPRSSNGTSYYHPEKQEHMAAAATMQRPPYPPMYHTPQSNSPSSVTSPQTHDHGRPMFSQQSTQMTQPMYPYTYSPLAQMQTPYGSHSQSSMAPTQMLPASYNPPQISHSQVSSHTPNLNTSPRIKSDPSQSFSQTPQQRPSLMNQQHPTPGPTPGSTGVGSSQNNVPASGSNAAPGPIPATTPLVVRQDNNGVQWIAFEYSRDRVKMEYTIRCDVESVDTASLAQEFKSENCVYPRACVPRDQYKGNRLNYESECNQVGWALAELNPCLRGKRGLIQRAVDSWRNSNQDPRLRSRRVRRQAKIINKTRASTAGPPQSAGPVSTGLPGPQSMPAPSSRAHTTLPSAGSQVHHHHGQHDMSPTAQDPSSAASYSTSQSMHRPSSTSQHLSSPNELRHAQTFSNHQAYPNSTGSLGPSMAPPMPGNMYQFGRPNDNSTMVPREQQDRDEEETALFGDLPEGKKRKFILVDDTQKNARVRVKVTLDQVNVQDIPDSYRKQNSVYPRSYYPIQMRAEPSAGRFSTDDTEADDGAPTIGRMTVPCTTTDGDSQVEVSQLTRGKREREQKINELGYRMAWGQGRVFSSRPIFLARALDAYRTKQRSALVGAGQDASTIPSHLEIRPGKRRWIERTRPASYAESSPPKLTDEAP

>CF317_000233-T1 CF317_000233

MDRSFSFTANDRNAVENQREYQDSPERSIPSSPPPEYSSPPRAPEPVSPITAYRSRSAHSGHSSKHDQSHSLPHRSMVITNGEDTPPPPPPHRSSPTRASAIRDSTVTTGIDNFGPYSQGGGLEPVAQEASARNQRQSGSEAARGRPPSLPPSAATGYFPQNSDPVAHNNRYSISNRPLTRLHEAGSSQSGFPLAAGAATPGSATPYTFSHASSSRHSMRSLPQSYYAMGPIEVYDDSPYQRPATSSKTAVDDAHVYAHDIVDDGDDGFMPEPKRKSMLPLNRERSRKVPSRGGAAVGGVAAAGAGAGMLGGLLGRKEKNISSSGSYDPVSNPDLTAAEKSEWLAKQSKRSNRMRWLVILAIGAVVVLAVIAGIVGGVVSSGSHSSSGGSGSDSTNNAATDASANGDLDLNSAEIKALMNNKDLHKVFPGMDYTPWGVQYPDCMIYPPSQNNVTRDMAVLSQLTNAVRLYGTDCNATEMVLHAIDRLQLSDMKVWLGVWIDPNQTTTDRQVEHMYKLLADTEDRSIFKGVIIGNEALFRADQDKAEAHAQLVTELNNARANFSSLGYDLSVSTSDLGDNWDASLAAASDFVMSNVHPFFAGVNVEDAAAWTWDFWQSHNVVLTGKESNNVIAETGWPSGGGTDCGNEANVCAAGQTGSVASVENMNIFMADWVCQALENGTNYFWFEAFDEAWKVSYNEPGKEWEDKWGLMDTARNLKPGIQIPDCGGKTVS

>CF317_000234-T1 CF317_000234

MDDDYERSIGSPDAASQDAGSDNDMDETMRDADAEEDDRQEDEMDDGDEDGEGEGEGEGDGDGDEDTGNQSEDNADQQQDMNTDSNDQMQAQSTGTTKPIDLSHPRPEVLTAATYDIVPTIAAPQSTSINAICGTADSRWIFTGGADGYIRQYNWVESVNGKTQLTVAQRHPFVDSVVKAGVLVTYWENYDARKQTPESTTLTTTQTLSPVYSLACQSQALWMVAGTASGAIRLQTVRHDEGHTIALLQKHTSAISVLNLASDEKSLLSGSWDKTVVDWDLNTGQVRTSFAPSGSQISCIESRPISTVPVPYESGEPVKPTTTFASNNAAESTNGAWNGTQSPVAQEKEGAATPDSLFGGDDGDDDLFGDSAEAGLSNGNAFGFDESEMNQTVEDAPAPENGIATDLEPTSEAPPVQPPGSPPKTDTSVPDNATSNGVPNIAPQNLTNGLAHAEDLEPKEPTQDHSKLDDQPITSDSTFMATSIDGSIRVWDRRQPLPVARITPYDTPPWCLSACWSPDGNNIYAGRRNNTVEEYDLRKGLRQPERVFRFPQGSGAVTSVKAMPNGRHLVCASYDILRLYDLKHSETNKSGVPFLIVPGHRTGTISHLYLSADCRYMLSTGGNRGWEGSPTEVLLGYEVGVPKGV

>CF317_000235-T1 CF317_000235

MDFSELTAYFEGNGRYQGHGGGSYTNGDTNGELNAHLSERFRGSARRNDGDRGPTASHATKILVNPDVATYVQAQKAIYEKAEPGDWLAMPEIPPSSEVCLPEGTSIVLPRNRIDQPWSKPERYLKAHYKLLREDAVAPLREAVDRFRKDPQRMDDNETRIYEQVRVVGMTFTFQGIASRVRFSTARSGRRIQWSSSKRLTSGTLVALTPKNDNFKTKCILAVVAARPLRNLESDNAPEVDLLFGDPDDLEVDSQNSYIMIEATQGYYEAYRHTLRALQKQSQEAFPLAEHICKLNPDVDAPDYVKQKPERDISPAADDVTRDSFRNVDILKHWPPAPSSGLDPTQWAALRDILTRKLAIVQGPPGTGKTHVSKIAVDILLRNRCQDDAPVIVACQTNHALDQLLKYIMDFAPNLIRLGGRSTEPIIKQRALHEVRRTENIEDLQGSCFLKARRDNEIESMKLKSLLLPVTGEDNAPTPPKPITADTLCDLKIITEQQRMSLAAGADQWVRSDASTDDPLQMWLGNAVSPFIPKYAQDSFGFEEAEEDLEAEQLKELEAEMGVADEEDSEMLQGHWVPLAMGHTCPPPTSQTLDQAKRMLDTATDLYRVPEYLKGAMYLIMQEKAKKTIKYRVKEVAKAYMKTITQIQIGRWERDAAYLSKAPIIGMTTTGLSKYRALVSALKPKVILIEEAAEVLEAPVGVACVDSVEHLILVGDHQQLQAHCSVRELEGEPYHLNISMFERLVRNKTPHKTLLRQRRMEPEFRELIAPIYPQLQDHQDVVTRKRTQWGLGHNYSWFFNHDHHELQDESMSTFNEYEARMVARFYRHLTRNTVPPAAITVLTFYNGQRKKILRYLKDDPETASTYNIVKTVDSYQGEENMIVLLSLVRSNISGKIGFLDINNRIVVALSRAKYGFYLFGNGGFLAQRNEVWDFVETRMYESGRSGDYIPLVCAKHGDEVRVKHVEDFDKLDGGLKPLQPRTNMPAKETSRETSSGRIRYEQHWPDGLLAKPSDEAPVQPNISQKQQESRHSIREGKKREVPERQPRAPPVPGSTGFDGANDLVDVCQVKRDSEIIHGAEQASLVSWDQEPIARNDALDEWIDPELLQWQREQDQQVKEMGTAFW

>CF317_000236-T1 CF317_000236

MPTRFSLSICLLAVTTFLRAIQAHTVLTYPGWRGDNLHSNGTVEDTGGLSSFAVDNGTLHPFGMQWEYPCGGMPMSQNRTKWPVQGGAIAFQPGWFAGHSSAFMYFNLGIGNNPQNYSFVMQNGLNIVGPSREPWPGTFCLPQVPLPAGLQVDVGDNATIQIIETALHGAALYNCVDITFAEPEDVAEVNEQNCFNSTQLSSNLIFTTEALASDSTTVNVPLVAMITIAAAALFALI

>CF317_000237-T1 CF317_000237

MSAPSSFRQLIGVQPSKASPSDSTLVIIDAQNEYADGKLATVDVSRTRKNISVLLNRYREDSKHSGKNIVHVVHKTSPEAPVFTTNTDLAAEFSELKPTQSEKVIEKLYPSAFASTDLQDYLQGLPDGVGNKVVLTGYMAHVCVSTTARVAAEKGIDVVLARDAIGDRDIPGIGGNEVTETVLKELGDAFGTVVGTDEIK

>CF317_000238-T1 CF317_000238

MRLTNSLVLAYASFAAFAAATSTITATATISATAGTSTMGANAAANTATVGSGGGSTDAGIGAGPNAVESVSNDAGAGSNDTVSGSGAAAAAGNNTAAGLSPSSGAGTGAVGGTGDASAGAEDRADGEGPPWAAGRGKGRWQGKHPHTPRSWTDWFGKRSVGRQLRRDLGLSHPRRSEGGSRQGMSADGKPQGAAQGQSQDHGHAQGAGKSQKRGIASSGPSWNDVVNNMAGQKQQKRDVSSQGGTYSGPSWSQLVGSPQ

>CF317_000239-T1 CF317_000239

MYNCYSPSETAEIVSRAGAAKANTRLDKIFMSAVMAGMMLSFACATLLSTSASPWYQDNAPGLIRTIAALVFPYGLCMIVLTGSDLCTGSFMFTTVAVLHRRLSIPKMLMHWTLTFIGNLAGSLFIVALITGYGGVFDSGAYLDEVQAFVTAKQMKPEWHQIFLRGIGANWLVCLACFLGMSGREYVSKIVGIWWPTFAFVSLGFDHVVANMFFIPTGLWHETPGVTVGLYIWKGIIPALLGNIVGGGLFVGTYFWYFYLQGADAAAIDGNVFDASPVGRLDVRSGNVDFGLRRKTVDEETLHGTPPAEGSLPGTPPNEGTGKKE

>CF317_000240-T1 CF317_000240

MADANPHTTLPFPPITAQHILNCSFHTWHPKLRTKTPKARLIPLSQAFVDYLRADGIMLPPEATRNDDDSGYSDDEDEEDPSAAWPEVHEKVKSTIAELGGKVVPKLNWSAPKDATWMSSTNDMECRTANEVYLLLKSSDFVTHDLEQAFDGCDDTADVPYHLVLRKSFNLNPSLEFRCFVRDRKLIAITQRDMNHFEFLFDLRSDFSLEIEEFFDETFKYFPDPNFTFDVYIPPPHKRVWLIDINPWAPRTDPLLFSWLELLTMDEPEDEDFVPEFRLVQRDDPEAYQFSASKYSAHKLPKDVVDASMTPGGMTDMMKEWKRVMDREDQEDSETDEAG

>CF317_000242-T1 CF317_000242

MRRSSVVSRASSARAGSTGAQTADANLAKMGYKAELPRSLSMLSVLGLSFAIMAVPYGLSTTFYVSLPNGQSVTILWGWVLLSLLSTAIAASLAEICSVYPTAGGVYYWAALLSTPKWAPIASWVTGWLTLVGNWTVTLSINFGGAQLILSAISLWDESYVANTWQTVLMFWFVMLLCLAINVWGAKYLDFINKLCIYWTGASIVILMIVLLTMARAGRRSGEFVFTHYESDSGWPNGWAFFVGLLQPAYVLTGYGMVAAMCEEVQTPEREVPKAIVLSVVAAGITGIIYLVPILFTLPDIDMLLAVASGQPIATLFKTVTGSAGGGFGLLFLILGIWLFAGVGALTAASRCTYAFARDGAIPGSRLWSKVDKRYDVPLWGLILSTAVDCLLGMIYFGSSAAFNSFTGVATICLSTSYGLPILVSVLRGRNNVKHASFSLGKFGFAINITTLVWISLAIFLFCMPVSVDGLTPTGMNYASVVFAAFATISIVWYFVWGRKNFSGPAVLKTLMTDDGVGVIKGQALTEAIAKEESGVTDQEAYVAEHSLEKKAS

>CF317_000243-T1 CF317_000243

MDFMLLPGEMRNMIYEEYLKPKALRFEVKRTDQGRRRWTTQNVDRSNILSVDRRIRSEALYLLHEAFNGRLEIGDSDTYTTLKAIRTQTGVPIPRCIITCLRIGGSMLMRSTNIFDQFPMLQTLEVRCDKHMHLDSEHVATPPSPRQEYHVRRFMELMGTSGVGLGWYLDMQMGAVSRGHMDPRWECCCFVLDLTRDVRKIAKEVDWKAISPGPST

>CF317_000244-T1 CF317_000244

MPLQSMLCSGAFRATRAFSWNSRLHCRAFSQSTRFHQDQIVSLKVGGNGFINLRITRPVRSTPSSKILVNFPPGPLLGGHEPGKALLEDAKQLSESFHDATVVDVRYRLGPRPQDAQPELDHRFPTPIHDIFTAWDYITGQLALQNTTFKRSKICLCGSYIGGALALMLALTNPSVVHAVAVENPLVDWVMLDELAAYSTENGKGNTSTRKTTKHDVNEATAQTAKALIQLRTSLFRTPSGYFDQFASPTLFLRAPGRDTPWTKTAALTDPELEIVEGMSMRYGEEDDEVGMVESDRPDDFGPYDDDYWHAVETRRIREQEYRHDTASSDTASTSTEFQRSASSKDQDSASGASSTKSSPQDIAPRRRKVLRRWPPNAQPEEVTLPHVNIFLTKAAPSRVGDQAVAQMADITPVTWPQGMELADLLKRACFWGRDKSFADERVTVSEHDPTTPHLERQEQVTRWLRAKFDERS

>CF317_000245-T1 CF317_000245

MFSRLLRPTTASKLASPCFRRGLATVQGNTERAMPIPGRQQYKATPVTHERATLTIRNGPIFHGTSFGARSNISGEAVFTTSLVGYPESMTDPSYRGQILVFTQPLIGNYGVPSSVRDEFGLLKYFESPHIQPVGVVVADAAEKYSHWTAVESLGEWCAREGVPAISGVDTRAIVTYLREQGSSLARLAIGEEYDQDEDEAFIDPEQINLVRRVSTKAPFHVSAPDPVAHVAVIDCGVKENILRSLVSRGASATVFPFDYPIHKVAHHFDGIFISNGPGDPTHCQDAVYHLRKIMETSQIPIFGICLGHQLLALAAGAKTIKLKYGNRAHNIPALDLTTGQCHITSQNHGYAVDAATLPKEWKPFFINLNDNSNEGLIHKSRPIFSTQFHPEAKGGPMDSAYLFDLYLDNVKQYKESQAAISPSRQSLPNPLLVDLLSKERVGVEMPDGIKNMIAQQNAGKQQGQQVFAAGAA

>CF317_000246-T1 CF317_000246

MPQEVRDHIYRWVHTPQIDGHPQVVLNLLLVNKQIYHEVKPLIDTIEHTITIGDLERFKAGTIEYMGTPQTIRVEGRLEWHMASLKHLVLNLKICGIAGMTPDCFDVWVGHNSKEQWRNLRRLIGIWPDIRAVPLESVRLDVAISEGATTERKKYRADLIRVIRNFKRTKVWAETADGDCSTHRGNRSLLLPLVKAFNQGRRYWDTESDIDNNLIVRYDTHILSTRAIVVDKDDDEKSEEEKIEEVRLKWSVQPVEDTSRSDNSVWPEWTGKEEAYICEKMIRREREHDREWVCGECLAVFDKPRELKAHIARGKGRA

>CF317_000247-T1 CF317_000247

MVFARSLARPASSLLSGAASPHFTRRAVPKAFGTNFSRSLTATASRQGKVLLVLYDGFEHAKQQPRLLGTTENELGLRKWLEDQGHELVTTSDKEGEGSEFDKHLVDAEVIITTPFHPGYLTAERLAKAKNLKIAVTAGIGSDHVDLNAANKTNGGITVAEVTGSNVVSVAEHVVMTILNLVRNFVPAHEMVERGDWNVAAVAKNEFDLENKVVGTVAVGRIGERVLRRLKPFDCKELLYFDYQPLDPQKEKEIGCRRVEDLEEMLGQCDVVTINCPLHEKTKGLFNKELISKMKKGAWLVNTARGAIVVKEDVAEALASGQLNGYGGDVWFPQPAPKDHPLRTAKNPWGGGNAMVPHMSGTSIDAQERYAAGTKAILDSYFSGRHDYRPEDLIVKDGDYATKAYGQREEKKHANA

>CF317_000248-T1 CF317_000248

MAAFRDIEVVVQRLDPTSGTWHPLEEADSQQTLKAEKNSAAIRTVILETQEGRFRFIIRVSAHFKWGTANGLIAKLTFDQGWRINEYPVAIFKPEVDVLRLFGKVPAGTGTLQVSEVDSKLEYILETIALPENMAKPEGPWCNTRFVFKKLEYIGSGAKSRPYVSTTDLARLRVVVQPRRIFRDPKPRLISLPSRPDEKARASSRDLAAKKGITLGTAMKFKAQDQPVPQVCQIPSIRIAPHEPRTFVFLYREPGHYDKINDVELIDHTRTKAAALFSGCNSAQLSDSLYGSDHTWDVENQTPIDTGRSTNRSSPLTWDCVESLPTPFPSVNDSRSRADLMNVSTRQEVTHASSANFVNEQNREAGTTCFAMRDRLREISNNTSLPSDRTLDTEEDTIVVNTDNWRSQQEDEPTSTLAPNLTPLTPPDQPAQAWSPPESQERAERLRYLLRLEDNIQAERLKAASQDLQRREEIRDETSFANNKIDELERQMQELCLRKTVWVQHVEVLSKEDADIANADIASRLAVLSRKSQGRADVLDQEESEAFQKIEKHQLEELAVVKNVSESAKKARKRRRSQCSAGSTPNRHRATKRHASEKSVDGHSNRVDSDEGRRADNGDLVEGED

>CF317_000249-T1 CF317_000249

MPPPSKPSAPFLPSTEQTSRDASELDKKKAMRESQENREDKNAQQAESKQLRDEDELEEEEEEDVEPVRDEPVPVKAEPVEPEPSKDAAEAQPKESKEPKDKDKVSKERELSPLGNPELDMVLSIPSPSEIKGSDKFSSQSSSTHPHLEASPYEHHFDTYTLVQELCKQQAPPPTATGEVSDEQVVPFSEAQSVTLMKAIRLMLAQNLDLAKEGLVSKSDTENETYLFRAACSELKTTMQTSRFMEAQKQRSQRAQLQHEFDILNQKVSQDIMTMREELKGMFNDRKLGLQEEKRQVDGKIQNLGYRISVDLNSEARSEVEGLRWVLTRRAALAIGAAAFMVLSFLQYYSVKAREQAEIDKKRKAAQKQAEERLRKEEERYGASGATIGRSTGTQTDGIVLGGTAGSGNYEESLG

>CF317_000250-T1 CF317_000250

MDETRIDAAPTPAHVFAYRAFRSVFVTSPESSPLQPRDYDHQQDHDKENTRRSPARPSRFTTSPVKIKRKDGEINAPRLEDELRLTPKRQKTVPVSPTKSILKNPHAPTPKRAGLRDVTVTFKDVRKSISPELLRQGQGSPVRVRSQPAMRTLFEDLAKQKPVTTVQVSASPPIPMQASGFDLDAYKAQTEKEMRRLIKYGQKWKEQAKRQDDENAKLRVLLKETRKDNQRLQKKISQMQQLNATKPTNTVSAVPTAENLTAHRTRARVCKLPEDYSVDCKPQRETTSESRLASTRQSSLQQKLDELQHLQPSPPAAPNEQAPPAKGQARVQHPRQQQPQTSLEKKIDLLKQATSELERSSLHEAQADREREMKQKTDALELEPTTGDQVQQVSLPVRTASLSIADDKKAAARERLRLKREARAASSQAALSVVRESKRETDSSKLDVDESQVDWLAMA

>CF317_000251-T1 CF317_000251

MLTLSALLLSSLCAATPLNKRQITNDAVSGTPVPPVGASGQQYPTGTLLGPAQSIASISAAGPAATLPADSWSLVANQDASADEGLILNFEDVGNPQPIRGENGNTDPGPRTYEYDRLNPDLLARPGTDMGDIPNSKWPMGLSSNRAGTGQNSGWARQQNTNELPVATAMAGVDMRLAPNAYRELHWHSANEWSYILSGSVRVSAVNQNGETFVDDLQAGDLWFFPSGVPHSIQATEEGVEFLLVFDQGSFSEDDTDLVTELFLRNPREVLAKNFQTDISTFDNLPQQKYIFNGTPLNQSLEDALAAVNGPAGHIPRDQYYSYHASQQAPLEVPGGSVKIVDPTVFPIASKFSMGLFSIEPGAMREIHWHLTSDEWNFFIAGHARLTSFEGPTASRTFDFQAGDVGYVPAANSHYVENVGNETVVYIEVLQAPKYVDLSAGQWLGLTPRQVVKDTPGLSDDFVDTLPRDKRYIVPGNADYTTTNFTVGSYPNTDLGGRGGQKRRMRRGTRQSEGMASTGYTRNKAVAYN

>CF317_000252-T1 CF317_000252

MPIVVSNQGWVKLPPPPKKRKPAKPVDPVSAFRGPLAYITGRPDFKEPVKKQSIKQLLIEAAPPPPPIVEARGSRQRSRVSSEPLASKNRHRAPTDIVEVEEEIYSPHLRPDDLQSRRSASPAASRHTRAGPSVRSRRQEPPRSLYEDGLEGHRHNRSKRHVSAQQAYYDDDSRQHRSHRERSRERYRPDPYMHNYSTSHLPQIQPIVIYSTPPQAQMGCGGHSCHGNAHYQQQYYTSPTPMLQAAPRQAALPAPRPPPSEVSSRSSESKASRAMSYKWYTATQPLAL

>CF317_000253-T1 CF317_000253

MFLLLVVFAIALPWALPHPVQPAAELLKRQDLDVTVLQFALTLEHLENVFYQQGLKNFSQEEFLDFGLDAEDVQNFQLIAQDEAQHVQFLTEAIAAAGSQPVAECTYNFPSTDVASFLTLSTLLEGVGTSAYLGGVPLISNKDTLTAAGSILVIEALHTSLQRAVIGVVPAANAFGTPLSATAVLSLAASFIVSCPDSNEPLPFTSFASLSMSEQQGCNSSPSQNSQMNTTAIASSPSSSTPAVTASTATANNPQCSSHKNATASTTQSSQSTAPFTNSTIAATASCGALSAGASVAFTAGSSIPPGSFVTFVSGLSVVSMMGEVNDVAISAMIPPGISGQSYVFVTSSDTQGKIDDAAVLFGPAVLEVSLGSAAPNE

>CF317_000254-T1 CF317_000254

MSCQRNDDVVKIEEHGSDTDVVRDLLAWPSDTTCGAGDQGLGCSQVVSEHSVTIDIYVTGEVKETVHYQTGTTELKHSDPDSPSMSEPDSKNNLLKYQGSIKQQERRGRPQLPHMIEQEAVRSTETGDSVGVFDLSPCRVVFEILSLKSTWTCKRIKIWDDVSSPRAFLVGVDGTCRVTAAHDMSQLHRC

>CF317_000255-T1 CF317_000255

MHVPAYWPVVVGVHCIEKSVVLVEVKKAIAIESIPITMLLSDDIDILSPVEVAIAMLDIVVVGYIDIDTDADVMVLLELTIDIAEVSMIVNASISLNKSLLSVNFG

>CF317_000256-T1 CF317_000256

MATEIVIFPIKDGDLPNDLNTTTGKVFQEILQEVLQQPGAQRAYWGIEVENPTNARLFIDWDSVDAHKNFINSDIYKPFGEKFGKVTDLSRIQMFHANLKPHPPSQVLSKSTSPATEILLLYFPTDYSQADQDKLETDLQKLMGIVEKNSDKYTANAGGWIVEELTVPGTEEKAKAYSAQIGWKSVQDHLDFRSHEAFKENIYLLRGAKDLKKLAVVHYHGNEVSSS

>CF317_000257-T1 CF317_000257

MQTKNLAMLLMAGSALAAPTARSDSAVDPALQNIKYTVMAIRSGSPVQYLPLNAIAGRFYLGGKTTSFCPDSDSCGNTCPPGNVTVFDGSCALDASNPEYYDVSEQTVWTTDNGVIGYDFSQVRPENAYNCPWYLGTSEGAVFAATIEPTYGADGFLACPTATDGIWQVLRDVTDESELAPPQGDASKCLSIDLVALKYNATEYGAYVYS

>CF317_000258-T1 CF317_000258

MHVLRLWEALTFLLLQAAAARQVTDLSTYSWTLQSLPLNISVPATIPSQAHLDLLASDVVVELEYGLADFGLRWIWMQNWTYTTTLSDLNSSISRTYLLFQGLDTFTSIELCGQHVAATNNRFRQYYFDVTDVLSDCTLDPDLSINFGSAPQIAENIANEPGQETWPYGTEITFEIPNRQYIRKEQNDFGWDWGPAYAPAGPWKPAYVVQLDQDQVYSRNVLIDIYRQGQKNNFIPDQNQPWVINASIDVLGDLPDGVSLSVTLKDALNATVMDGNLESINQTNNVVTGMVTIADDMVELWWPAGMGSQNLYYLTIDIKSVSNSTLASVIRRVGFRTIVLNEMPVTEKQIALGIAPGNNWHFEINGCPFFAKGSNFIPPDVFWPRVNASRIRTLLNAVVAGNQNMLRVWSSGAYSPDFMYDIADELGILLWSEFEFGDALYPVDEEFLSNAYEEAVYQVRRVNHHPSLAYWAGGNELENLELPTANQSDPERFPELLAQYEQLFLTTLFPAVFENTRSISYAPSSTSNGFISLNFTDPPYFEERYENTSGGIYGETDYYNYDSAVAFDTSAYPVGRFSNEFGYHSMPSIQTWRQYIPEDQLFFNSTFVIYRNRHYPPGSLNTSNYANTSKGMGEMTLAAERWYPTPNKTDRVANFSAWCWTTQVFQAEYYGSQIQFYRRGSGLPQRTLGSLYWQLEDQWAAPTWAGIEVGGRWKVLHYRAKDLYKNVIVSPFYNETTKELGIWVTSDLWSTVDGTIDVVWYAYNGTEVGSALGLKSHTLSVGALNSTRVYSANLEVGLAGYDMRNIVMRTNISAAGQLPNSNDSTTFAHTNWFHASRLSEANLTDPGLALEHDSANQAFTVTATSGVAVFAWLDYPSGVIVTFDDNGFWLGRGESKTVKYEATGDTTGGSWARGVRVQSLWDNNVAE

>CF317_000259-T1 CF317_000259

MADIMDKFWSAPPVSRTLVAAIGAVSLLVHGGLLGFHRVLFYLPYIWKFPFPELWRLVTPFLLTGGGFSALWDMYMFWTYATQLELNSPRFSQPGDFAAYVGFVAMSILITAGLILRSFVFTQALLLAFIYTYAQDNRGRKVHFIFFQIPAEFLPWAMLAMTLLMGGTTATLQQGTGLLAAHMYDFLTRLYPTMQGGRNYLQTPGFVRRYFTGNTTATTNRAYGTSFRPAPQNPQPAASSNRGWTSGFSNSWSGRGSGHRLGGD

>CF317_000260-T1 CF317_000260

MATDAMDYSIKPENVTPTISTADWPLLLKNYDKLLVRTGHFTPIPAGCTPYKRDLKQYVSSGVINLDKPSNPSSHEVVAWVKRMLRVEKTGHSGTLDPKVTGCLIVCIDRATRLVKSQQGAGKEYVAVVRFHDKLPGGEAQFARALETLTGALFQRPPLISAVKRQLRVRTIHESKLIEFDNDRHLGVFWVSCEAGTYIRTLCVHLGLLLGVGAHMQELRRVRSGAMEESDSLVTLHDVLDAQWMYDNNRDESYLRKVIAPLESLLTTYKRIVVKDSAVNAVCYGAKLMIPGLLRFESGIEVHEEVILMTTKGEAIALGIAQMSTVELSTCDHGVVAKVKRCIMERDLYPRRWGMGPVALEKKKMKVDGKLDKYGRPNENTPAKWNAEYQDYNAENDTTMNDGPGAPLTSGPAGTSETTSKSDVLSAPPVAPTGEVVEQDAGVDANPQTNGEKKRKSKHEDETPEEKAERKRRKAEKKEKKEKKRKSQGGGDSDDSD

>CF317_000261-T1 CF317_000261

MPPKSTNPASTPSLQGCAIAFASNSIPADCLGGASLANTKSLISNNGGSYVTKVEECTHLVVSDNQYNKSLAKVETAKALSTVHILSFKWLEEALRATYPVGEVPYIIHDRNQTWGSSAKPTTKAITRNAAKASRSTDKTNDDEDVDDTKLPPSKKRKLDIKDDYSAQAEPRAHGGKPMNVTKNANLKIPLDEVLASQWGQDYTVHISDDSTIYDVTLNQSDSGANANKFYRMQLLKDPNGSWWTWTRWGRVGEDGQSKMLGGGNYDLAINEFEKKFKDKTGNKWEDRATGPIKPKKYAFIERSYEDSDAEGEEDLPGGEKQKTKKGEGAKEEELVESKLPEPVQRLLKLIFNEEYFNNTFNSFNYDAKKMPLGKLSKSSLMRGYEVLKTLSSMISGTGMAEEIEALSSQYLSLIPHVVSRSSRPPVLDNMNMIKSEIELLEALTDMQLANDLMKDAKKGKDKAEAMNLLDRQYQGLGMQEMTPLNPKSTEFTDLESYLTGSVGHTHGTQYKVQDIFRIERNGEHHRFDTSYYAKIPNKNRKLLWHGSRATNFGGILSQGLRIAPPEAPATGYMFGKGVYLADMSSKSAGYCCSFNTGGTGLLLLCEAELGEPPLKLTHADYDAGKRAKEANCLSTWGVGRTAPLVWKDAGTVSKTLAGTSMPDVVSKPPGATNEGGGLQYNEYIVYDVAQIKLRYLFRVSM

>CF317_000262-T1 CF317_000262

MKFSTLTLLSIAATSNAFSLSHSSTELTLPIAVSLSIFHASQAAAFVLPSHITARGDTTIAKRQRGHGGNSDIKADADADDDDENDDDDTADDNGTGRQRNQQGQAPGQSRSSNSNSIANGDGNATFQRNGRGRARGRNRDHNAQANANNANANGNGRGQNNGQGNNNGDDDDDNDNDAGNRNDNGNANASNQNGNSNGNNNDNDAGNAGNVNSTSISSQQNSNSTSMDSSNTAPASTSSAGNSTSTADPDSDTNSSSSSDDSGTGTLPAGFPPATPDAGFVAGADAETQVKRSKAAIARREFREKRIAENKKRGLKVPVAPKRDVNREVYDV

>CF317_000263-T1 CF317_000263

MAKRPEDTSPYTKRPLYIYDLPPELIETLTLHATTAIRPNTTEEEPEQQPSQRGLTEDGVSSSTACALCKATFRDVSDQRQHVRSDFHRYNVKLQVKQLPPIDEATFVKKIGELDESISGSDSSDTEDDEDNTHPNDTTLSALLKRQARIAQHDEEDGPLPLRRRGPGNAPMYWLTSPKLPEGMGLGIYRAILSIEEQESAQKNLVDILKRKQIQPIQAKHSSSKQDAAPRPQDPHFFLCMIGGGHFAAAIIGLAPEVRKGPGGVEERHAVVKAHKTFHRYTTRRKQGGSQSANDNAKGNAHSVGSSIRRANEAALEQDIRNLLAEWKSMIDSAELVFVRATGSQNRRTLLGPYDGQVLTSKDKRLRGFPFSTKRATQNELIRAFQEVTRAKVDKLIEEQPEKPTEQQKPVQKPKIQPAKPTPEQEELLLHTSQLQNLIRRSKAPGVLLYLKKNNLSAKFTFYPPEQGTNHHAPTPLHLAASQNAAAVVTALLTKTDADPTLKNSDGKVSYDIAGDGRTHDAFRVARHTLGEKAHDWSAAHVPSPLSPEEAEAKSKRETQEASAAEAERRKADLERIKQEEQDRKVGKIEKKAGAGKTLGAVPEKTWSEKNDEDLRGLTPEMRQRLERERRARAAEARMKALSGR

>CF317_000264-T1 CF317_000264

MTEQQPSPTDSPAQPHRLLKLTLLAYRHPSMSEAEFHTHWSQIHSAKAAAHLAKCGIVSYRQYHTPSALRAQLAAALPSLNLSDDQIVDYDGFVELVMPGLDCFEKVRDDPYFKRVIAPDEAVFADMARTKVTVGWVEAHVEGGQVVEVGRREAYEGA

>CF317_000265-T1 CF317_000265

MPARHPDEYEYAREARGVRRERDGRDVREREVVREAPRARRDMDVEPQRPSPRMMDPDMMGREPAARVANAGVPRQDPRAMRNMPAPRDPRDRDDEMMYDNRPTQYAPIREQPGARRFPHEGEFDDIPQAVRPIIDPGRSRDEPRPSYNEYFLPGEGIDREVIQSEICRYLGQDATCKPGAHTDGRRGYIIRAYRALTTEMIRSLKEDSVKYSRERDSAARRNRQPASFAAFRDQARYGGDEMMIDEPEERYQQRYEEPRLRAAPAVSASYLPDPGYSSAYYPVTQQPPPPGVDARTMDPRYIPGSNTPPAGRNPSYQPGGYQPSGYQPATTRPPVSSIPASGAFTDARGNVVRDPGYGPSYAEPRARHR

>CF317_000266-T1 CF317_000266

MSADNDRSIRYYHYNGSPYARKVVWYLTLRGMPWAEVKQPMVMPRPDLEAIGVGYRRIPVMAIGKDVYCDTRIILRKLEEFFPHGKIGASSPDGYAIQKLIEIWHVEGPLFSRAASSMPLSVFKDAKFAKDRGQMTGRPWNMEVMEKFRPDALAYVRSAFSFLEKLLADGRQWIISTSEPSLADIEAIFVVHWLNGMKGALPEDLVSQAKYPKVFAWIGRFDNALKTAQAKAPKPTKLDGATAAKQVFESGFAEKEGTVEVGEFQKELRQGDQVEVFPTDSGMHNKDSGRLLSLTEDEIVIDLENGLRLHTPRAGFRVKQLGSKL

>CF317_000267-T1 CF317_000267

MQIRAEKGHLVRLYHGAFRIVGQDHEYSRKISALTELEEVTIQSICGFVYRHPKQRFHLEYSITYSSVRTQRNKGSEKYSDMVRQEVQRLMDSNENFSGQKFIPRIDRDELASGEIISGIIAQESNLNLDDIQSLSSRIIREGATGLFLACVNRFLGIDDFRHLLEVCHYNDDKLPQISENRTHLRDEEYRRLVENIHIFFAKTIDGGGRHLKLRKHEIMPVLHVTQDSTEVKTQLGHGAVGDVCKVRIEPGHHNLSGHIKSHFALKTIHERNKDAFEKERAMLSRLRKQGHPHIVRHIMSWCQHETYYILYPLATSNLRSYMGDTPPPRVTWPSVRWFLRQISGLANAVHHIHEQQAQDEKIEGYSSTPPPSIDPRTAKRISVESIMKPTHKRSGYHHDLKPENILIFEQREGINPVFKISDFGAGKFTDLTGEEKSHEASHLAGTASYFGPEWDQARSRPFDIWAMACVLTELLIWFLQDAKLEEFHRGRVQSSLSTAGFSYDYYWFQDESQRRLKPVVSTYLEEIERNCKQDDHPRPLPASGLKDLPSTIKSCFEIEPSKRLLAGELYEILQRMNEGAEVAGEEVEISIHTPSSPGSRRSQDDGSVRLENGTMDPSA

>CF317_000268-T1 CF317_000268

MASGTAYAARRWLSDQVETAPSIRLLPWRVHVDLLDIAVSAWRPYIAYLSERIDIQSDLAITADLDEDNLDHATLETRQFLKQLEDMLAEVTLNLNHARKTASALQRQYQSKRRCRAQGDVHEMNRHSVSEHCSDTFIRIEDTLLRYLEQSEALKHKVKSTSNLVSNILELNNSNTLKLLAIKAADEGAIMQELTKKATQDAAAVKVLTVLTLIYLPSTVVMNFFSTSFVNMKTNASGSSVLTVANNWWVAIAATIPLTMITLYAWWFYVQKELRGDWPMTWRQIAETARNVQLSLRNSRRRSRSVPGSYS

>CF317_000269-T1 CF317_000269

MELSRRSTSTSGTPSVALNHTQTVKETYLDPNRLKAYMAQNHAPGTYSIKLKLGCPSYQRGNLNIFGGGGEETLHELNRVARKCYLFYDNQQKHNFTYEEMEARMDEVEGTLRGHIGLIEDSLKRIEERQMGKKDIKGGWGQGMSDEASENYSKRGKDDRGK

>CF317_000270-T1 CF317_000270

MSSNGNSVDFPQFEDGDVSVVVSTSRIYKLHSYVLRRVSRYFKNIFETSEPPRLTAAARREGYTTWRFQLYQDHTLPNDPGILVPLQISDSGRIAGQNFPPRGANTTGGGEDNDKCWDWLFGCFYGITPDFDSSTFGATVYDCFVLIDCADTIEAVDHVREVVDLALMRQDEVLWKSIAGSPTVWAELGRRVKSPAIFKEAVAHIVGQWKMLDAPSKKQLPKDIQRLCIRKWEELELAKRAIEIRIAGHYPPFLCRNHADRPHRTAYASDIYSWMALSYFRQYLAQSGNDSKNRQAEDGGYAWYKAFAQGGDAYLHHEDMRTFHTFFPMSSKACSVLEAHMNVLKEDVKDFVKDLMVVRTHVDPANLEGVSKPWLTCTIVEKDDLPWNVADESDDDDRGLNTVARQAMQIEETGIEADDEDES

>CF317_000271-T1 CF317_000271

MSGVPATFWSGPIRYLRWASYEKPAIFWSVVVGAMGPVSLLVVPSMRRWAGDETPKMIPLTYPVPDGPRQIPKGYDD

>CF317_000272-T1 CF317_000272

MRLFSSLCVLLWTLLTFVTAKSAVGDRVLVVLEDSAEKDLYSQFWKDLEARNFKLSFESPKADQLSLFKLGAPAYDHVLLLPPKSKGYGPNLAPKNILDYTNAGGNVLLALSSETATPAAISSLLLEFDISLPADRNSHVVDHFNYDAKSSADDHNVLLLPKAQPLRPDVMDFFGWEGKSLLAVPKAVGQTLGASSPLIYPILKAPETAYMHNPKEEAGDEQDISATGSQIALVSALQARNSARFTVLGSLEMLQDKWFDATVQPPSGERSKTANRDFARRLTEWTFKEAGVLKVGRIEHHQVMDATKPVANTTQVGFSDPEIYRIKTDVSFSIEVSHFSGGHYAPFTVPKDDSLQLEFSMLSPFHRIPLTETSRTPNSTIFSATFKTPDQHGIFAFRVNYKRPFFTYVDERRQVTVRHFAHDEWPRSWRITAAWPWIGGLWTVIGGFVLFVVLWLYCEPPKEDEKEKLKKETS

>CF317_000273-T1 CF317_000273

MPSSCRELRAALAACLQNSDCIMIQRHTPLECLSPPLKDELPVQCQQLQRGFRDCKRGMVDMRKRFRGNQPVGAAANAEMEAGSDGNVLAPGQKIDGIGKKPPQLYGGKPAFEPVRERSGDEVQVDLSQSRGL

>CF317_000274-T1 CF317_000274

MRLLASSRVPPLRTTRDDVTEDPEASAVNGSPELLTANLHKERPTEAGTDEETIVNDNDRSGYPAHPKLEPRTSRRAWYDEEIERLGIDINRLVQERAEIRRTFAEEKEQLEATIEDQQKEIEELRQDRDAIATMYEEFRRGADGVFDRPTKRRRP

>CF317_000275-T1 CF317_000275

MGAGASKPGDASKQVFTSEHPIQFSQEVIDSLQASSETNSTRAKSLELHIQQRVAAELERLTNENSSLLEKARQKITASDDKDTSKKSDRQPGLLELPSVTPKDLFKTETEEEKRKKNASSKKVQEEIEKLQKALGERKVLKDLPKEVSDAREEVVSCLRLHDRQSLNCWKEVEVFKREVRKMEEQFVGKVL

>CF317_000276-T1 CF317_000276

MSQHSFGDSLSAEQALRMLSSQTPELPLNRIERYPHQIDQAPYLCGSCGLMLFPWAPTGYQFSGICSQCDHLNDELQRQAIEAYNRRLLSIGGLQRALRLNDRQCTCPCKCEETDVEQQDTNYEDDSEADDDTDSHLAGRAVAAEQQMARRYGWQTNVVCKQNDDNERSMFYEAPGHYAMACMHDIGIPGESSLANSENTVGRIEAAMLDKVLEPWELEAVHLSTGRRYSSLSRTFPDARTMQLPTHWNPEQHRQPTPTCPHCGKSYNEEFVEWVQSGNATICESCGLVERPEMLADDPDIQVFGVDFRRAMDNCYCRCECPMDIVSPAADSDSTFQEAFYEASMELDSPPPTLEQAGPLPGWSSDHSPLSNTTPVDYASLSDEQIPQVPVNAQPFDGLVAHRESVRERFGLPELRFSDNSDFTTARHEEVDMAFEDADTATAFESFEEAHSYMRAAAYFVSLAHASADDPLHQLIRERLADYAERVDHEVEAEQTRQQGREGTF

>CF317_000277-T1 CF317_000277

MRQGSILAFLQPQIPAADHKRTSGAITITAVAPQSTPASESDKYGEGSHDAHRDEVQRLDHVTLPAKGNRNLPGPRAAIANVTTSYLDRLKSITTTLLPVRYSDKFFTECLEPEKYSAIAFVALYDSKVVGWIRCRVEPFPHAGNEVYQQIYIQALGILAPFRSLGLATELLRAVVDQGTSDYPKIRSIYAHVWESNEDALAWYDKQEFKRIMLQPQYYRRLKPSGAWIVRKELE

>CF317_000278-T1 CF317_000278

MANTLDESLTYEGHIASMSQILESVIEGIVHDLVLEVHRDEKIARMQTAVVDLNIRAEKLGKNINANSDSGVENEAVETKAAVGQAGNIQLRGNPLKQVKHIRCATCRLQRLWYPRVGFNSRPPSDPTVQYCKTEPMIIIDKHDVHGQRKKGVKVKGQPKGKGKGNKLRESSPASSIGDPSTPQSSMPDSFEFKEIDYPAAKCPNRNSHLGDHWKAVNLFATHLNGSCWLKRDRAALREANAKMAGTPKDSRANSPKPAATNGVKRKADEKDSEPAPKKKQKLSENKKLDKKAAPPPSKLREQSTAGDDGDDSPVKGDPDTIQVMPKSAPDGETTGKLKLTGSGVARKKPPKSGKKS

>CF317_000279-T1 CF317_000279

MNLNLCNSSFIDIVLSCDKSAHTTEEVSYAQAQADRQRHAEEQALSAERKRKREADFPDHLKAARVRVQKHQDENEQQSIPHEEQTGFESSSQLKLETDTPSAARDATLPHIEQTIWAKETDLTATRIGDEEVIETNVDAEKLDVIYDYMASVGCVSPYRKSVYKIDPMSGARCGYVRSIPNFGVLAFKTISGLPQVLWRVVHSGSAGSVTTSQLGAQLYSQARKVHGTSSDFASFRMEEIADQLKRHINGEKENFSSHWISTTDSFDHAWDKARSHHCSTKTNDVKIYRIDTRTLKRPTLVLPMYGAIKAWSIEDGLLEWRWKMFKYSTLTEWLVWDELDADDVEEIPYELFLRPPQSNPSRSCSSVSKRAPNLMRILQKAAHSASKGPDYKRGRRIPRTAHHKKCYYTPQQEVAIQIFDEQVHVGRFGKLPTAPKVVQDDKRSNISTNLLDDIYACVKQWRSGNLLFIWILSTLTKRFYLEDMVQTIVSRYSNVVSAVLDVDDSGYGAAVGTDRKVLKYDIPGPHGSAKVDVNHYQELMKACIEEWCKVSQKDHSEYSGLDVYYMYGDHQTARRLLLPQEKGLLGRCEIVDSLSVQRAGMASVKHPSISLVQSVTKVVSHTESHGECMARRAVQYGAYTKMLAPDVPMKRQLWYDTRKLTGQKAKEKADEMKTRTAH

>CF317_000280-T1 CF317_000280

MASYKIPMTHQLLLNRTTTTPDSKEEQNAVDTDYPASAKIQPDNVKDEDIDIDVARGRSIHRHDHSQPRPKTNGNTCTSISPYSRSRSAVIPRQSSRRPRREAGLSISPIRAVYNHAERGDVYLYDAYCHIPGFQPRARSGRSAARISPGAWYDRSAHLRYGLAHETRTERCKSPDWDDEGDEDAVAKRMVEARSKRTRRKDKRLVRSWVGENQEGVGRARESAEEAAAWDTRAEACGESRTS

>CF317_000281-T1 CF317_000281

MGESKIAVSEIQSHNKEEDCWIVVDGQVWDITQFAPEHPGGPAIIYKYAGRDATEAYSEIHAPSIIKNGLPEKAHKGTVDETTITKEWSEPLPSAAPKAASKSVAAKPPLEGIINSHDFELVAKNSATAKTWAFYSSASNDLITRDANNSLYNRIFFRPRTMRKVMDITTKTALLGKPVSFPLFVSPAAMARLIHPDGELAIGRACKTRNVIQSISNNASYAAADIVSQPEVLDHPFIFQLYVDKRREKTNELLAQILSHKNISAVMITVDAAAAGKREADERVKADESIENPMQRNKPKNDKMGGGYGRLMSSFIDPNLSWDYVSWLRGEIGNDIPMLTKGVMSADDVKMALDIGLQGCVLSNHGGRNLDTSPPPILVLLEIHARFPEIIRDHHPQSASVKAGLTKPFSILIDGGIRRGTDILKCVCLGAVGVGIGRPVLYATGYGQEGVEHLIDIYRDEFEVAMRNVGLTSIEQCGPEYVNTGDIDHMVCKSTSHPYAASWSAQVTRQSKL

>CF317_000282-T1 CF317_000282

MMITGSNLIESQNAVSISKQYPGTCFATIGVHPCSTQDFENFEGGAGKLLEDLRQLALDAKATGEVVAFGEIGLDWDRLFLSPKETQLKYFEAQLDLAVELQLPLFLHMRNCNDEFTALVRPRLEKLPKRGLVHSFTGTAEEMRSLVDLGFDIGINGCSLKTEENLAVAKDVPLDRLQIETDGPWCEIRPSHASSQYLKEAPATPKAVKKEKWQKGLMVKGRNEPATIPLVAHVISKIKGITVEEVCDAAWKNSIAMFGLGEGVTSD

>CF317_000283-T1 CF317_000283

MGSAKEHWSLADQVAKALCAMRPPAEYSSKTLMLDQSRPPTPDGTGRPTNFQTIAPGLYRSSYPVFEHFEQLESLSLKTIITLVCEELPLDYCNFISSCGIVHHQIPIPANKNPDVYTEAAVVYEVLEIMLDPSNYPLLIHCNKGKHRTGCMAACFRKACGWTDEAAIEEYVQFSAPKDRELDKVFIERFDPSPLKPLALDRGYVGGVYKQPQAGDTQNSERSVYTNNSVATYSNGETGEILHEYQEKVRKENNELMESSRLWSHR

>CF317_000284-T1 CF317_000284

MSSNVGLTTPRGSGTSGYVQRNLSTLKPRAAGYGQPYSLNDSARPLKIRKPDEDILKHDRLREIEVKVMELQDKLEDAGELNEEEIEEECEKLRKKLVEDAERGRGAKGEKGGRTKSYQVHELAEAKARESEKMRRALGLKGGVKDGEEEGDHPMARQERRKRDADLQRVKREGEDERDDGGRARRYQDD

>CF317_000285-T1 CF317_000285

MAFASIYPPSTKKSSIQDRRFEGLTILQYNFSVITWILVGASLQALAVYLFSSGRYILLVSTLLLIAKIMKTLLQAYNVTPNPYLEDVFAGRTTALLPNQETGEIETASSRKIAVLHLGAKSNHPFGYFAPQFKVVGGWLDKMNEGFDYGTTKGFLGQTTFHRLDERGAPEIVAISYWNSIEDIWAFAHGDLHREAWQWWEKTIKENGYVGINHEIFEADAQHWENIYVNFQPTMMGATTHLKKGGKTIEGGVVPDQWISPLVDARRGKLAKSSGRMGREISQYDANRVAKELYQ

>CF317_000286-T1 CF317_000286

MEHIQPPSLYRANPEWFKGLLKLTDKDFKQLLNEDPEELTQLTEGEHSTGDSALQSNSRRSLKVVLKVPDNLGKAVGKQVADQHGDYTSNTSHIHQPEAPMSTTVLDSSEDSRTRFLSPNSTTPFTSESKLSEQYGDNAGNIIQTTDKLANSSKKSTVSLSGEALTEDSEVGDTNRSLQHESVSATADDGDTIIVDVEGRKAMMLAEQSKKKSPMKRQRASSSRGNAKRRRIHDKQETDARRRLNKSTVLQKLTDNFSHTLPILRRDTHPSPAESSMNDHMMPMLADKIVTESSTIRIPDREEAESGKQTPQHKEGTKPYNPTSCPNPALYWVTRLLQRLPRPRNNTTLSIGAAEQDESLRGELEDAQAKMVELEAKVKLLEEAQKPNLVKVKKLSKRLQLALERNIVLRRGISAKDRILRTRKREAKQATDRAYKLRFELDNEHTLFKQSQALAISLQGRVECLEGKHQSAKKAFGNAEASLTTSQAEAFQQLYELVDTQQHTIQELRAETDVLQQERESHPKTLSDLKAKRTARQCQPESKKTAQRLLNLCHEFGLVRQLLQEKTRSLEQSKQSADDLRTNFSGLEESLHKAQAEIDARKQEQEEERAEKNPQMNVLRREITALRSAGFNSSTDQSLMAVNSTKQGAERLAKEITDLQSQVQEATSRAEKSKHAVQKLANLYDALVLQHRTLSLEHETSPRGGYIAGEHEQLANKVIIRWQRFFAGLVYVLFKLIDEDFNTSRLRQIMRGKGIDRDVVREIQARAAQWEPFVTIALQDRCTDDTRAYLTKMKQQLPKTSAEALEHAEETGERECEG

>CF317_000287-T1 CF317_000287

MPSSYPPHSGRRSNSGSRDRRDRRQRPFQDYEQEQQKQTKWRQLRGHLVAMASEFIGTTMFLWLAFAGTQASAQDGATSASSQGLLFVSLSFGFGLLVTVWAHYRISGGLFNPAISIALGITNNVPWLRIAFLIPAQIIGGIVAAALVKCMFPGPLAVVTTLSQGTSIAQGVFIEAFGTSVLVFVVLMVAAEKHESTPLAPVAIGLALFSIELATVRYTGGAVNPARAFGPSVAEPNFPGYHWLYWIGPLIGALMASAYYGMCRFSNYWEVNPGQDSKGHKGADKA

>CF317_000288-T1 CF317_000288

MNDTISTINILPAWVSEHITSVSTRPCSNLAYQYNISALVLVGVPLLVTLVILLRDLFTNSIAPAEIESRPPPPELEVQAQSHQSTRPIVLQCSNVTTQTTAATQDSPTPNSSQIVYDHLRVAEVENEHAQQRADWLAAREGLRSQLLQARTKIELLLKANQIEIRTRARGKFLAALEVVGSQESIAEFSARQERKIEAFKTKVQQQYDQRLEQSSRARSATANVEQEIRKQAEQDIRVLTEQRENDLQQLATTSARLQELERQFPVREQELTAELNRLRDELEMASQQRDNLLRKQDRIPEAAEPLCASTGLGTRRASSAILSPVQPVVPTVKEPAADPSTEAKESEPVPLKQPNSFNAPVTISPREIEAIQNKVPSSPPAAAPTSSLRSTSGRPPFTRPTLNQYAESSRAHARRQLKAPSRVPPAHDVQNFEPSASHSAYDEAQDSCVSGYAFAWKGPGAGQTPTTADLENAFTKPFEALQDAGRSGKAPRSNGEAPVRLSPRRSRHPLANEARQLKDDEHSMTAKDIPTKYRGPAVEEGPATPGLEGFGGSLPADGTATLEDNRRAAIPYASSNKRQKEDLRQPRKAQKQRPVVGGTSRPSTYQRPVIESLPPTPGIEGFGGTLPADGTATMEENRRTKLPYAKYATRQREDLRPPVKLQKPQPKQSAR

>CF317_000289-T1 CF317_000289

MPASRPQAIQPRDESEDEEISDNASATSGNISSSGEEEQSDGDNESQSSIESEAQPEEEDLKDISFGALAEAQARLNPNPRKRKLADREGSIESAGSTKEFTERDRELRKDIDYTNRGQVKHISRTSKHAPTAMSTRNPVSRKRAIFSPPPADKFRDPRFDASVTADSRRGNTSSTQRVGKNYAFLSDYQAAEVLDLKAQLKKAKDPDQQAQLKRQVMSLEAKLRNAQHQRREAEILQEHKRKEREAIREGKKARPYYLKQSELKKQIEQERQDAMGKRARDKSDKRKKKREKTKEARDMPRVRRFAE

>CF317_000290-T1 CF317_000290

MDRMNAQYRRSDLQYSTQTPPWRATQLPHRGVTTQGEYYGRNSVLTGVDIYALVDSWRSYKNDATNLQTQAGSTLNATAEPFEPPTKAPQQHYTPHEPLRSVHTYARYPSYRVDKPYRVSNGTKERNVVETNNQSKNKHRNHKKQAGLEPLITEASSAQAAPPVQPSAAFASRYPVRSNRGLANKPLPSIEIPDPVNTPAVRIVLTQRGKDSRSRTKSPQAPETTAAYRLQAHKEPLRLSAPRKLLVVLDLNGTLLYRSRRKSVHMRPGVTPLLDYLFSNHVVMVYTSATPDTATHLVDQFLHPKYRTQLAALWARDQLDLTKEQFAIKVQVYKRLEKIWQDESIQKTAGTGNRWDQSNTVLVDDSKLKASAQPHNLIQVTEYTAEQDPSKVQSEDKHNQRHQIQQDIMQQLQMKLEELKYQEDVSRLIRKWQTGEIAVPRLSGQEVVVEETVDQNTVKDEEDSIQAIKQVQPRPHLPTPDSIGDNSTREGDSIERPVQISDDEDEANGALLSPARSMRSTVSPIDEAVFRELLEGTGK

>CF317_000291-T1 CF317_000291

MAGMFSSFWRSKDPEAKDANEKPSVYGIGLDGKASNVSRDGDRDSDAMLVEKLSPSVATPDSIAADIENQTLGEGRMPTTNASIQNNAPSLPHSNKRPNDPGDTSMNPKKKRSSSPHSDDLNLDHDQGVDTEHSGSLEVLALSNCQEHSFDHDDDDASRPSLRLLRDLETRAITISQLIQEVKGIYAGLVMVEKKCIEIVSQQAQNPTKLSKEQWQALIALHRTLLNEHHDFFSASHHPVTEGHELRELAARYAMPARMWRHGIHAFLELLRHRLPESLEHMLSFVYLAYSMMALLMETVPDFRETWIECLGDLARYRMAIEEADLRDREIWSNTARTWYDEAADLSPNTGRIQHHLAVLARPNIVQQQFLYSKALVAAIPFKNAGDSILLLFNPLLDQTSASAARYQPAEFSFVCAGAVLFRRRSVVEYDMHRKQFASHLEGHLARSGSRFKVQGPEYAGSLFAMMTDFGNAENTLWRMVFEHQQTMKHKYCERTGSEIDKLPKDFVVQDSAIIDPVKAEYWNANIMEHHVPSPALPLEKSFTTSDDISAYVGPLLFDTISDVASHIGNKNVVPFMHMVLAHLLALTWVPGASVYIEKCIPWEQVVQFLNTVGRSGVSYESVEAKDFPKSFGGGKRQLPEDFSMRGLMVMTYYYKPDFFNLEDLVDEDERLLEQPSHQAPRVERCIWAAIQLATDTNNRWFSYDKQSKQFSTTAFSRGLIQSYGWG

>CF317_000292-T1 CF317_000292

MSSEYIKNSSRTSSDYRWEYHFGPTMDTNVTFEQKGDPFQYLVPAYRVSAVTASPYEGLDYWSPVSELGRPDHTRLTVMFIESCRILYTGPSNDYIFPASDRITDGHIDQESNPHEVEYEFVRAAMRKATTYSAIEFRRGSALLAKECVADLISQQLDDHQWILESAAPFETSLSRIQHDALDIAIGFGHEKVPGRYVDETLDWARGNMCGLYKFKIPAGFLNIAFWPNDTTFIVAGIICLLGSPTPMEYETPEKGQQLGGNMKVIECIYWLLCWIYNRNRKGYARSALGVIRHAGNLLANQLKSVFHQPKKTRPRTASANGRPPQDGGTPG

>CF317_000293-T1 CF317_000293

MEYTQTLLEYSGTVADGVDVPEQQSQPAQGQGVGWDEQEDSDEDRKKLKETDSMPRYARDQNNERQPRSYAWTEAGTRLSDPRGITSRTTALETISPLLNSQSGPTGLALARNSRAREAVTARRLTTWGPTVEYPKALKPRAREGPATQSLGASALIVPNPHQNRHPRPQALDDEQLRGRGAGRRPLSARHEDLAYSESPDTPPETQPEPLQCAPS

>CF317_000294-T1 CF317_000294

MANPNPFPPPPMFNGQYAPQPYYQPYNASAPTPSGAHFPQGMQHPPPFPAQASNTQRFEANSNTSTPPPPLPFLFPPNVNLENLRKHIEAHGLPPPPPPPPGQAFPATFAPNLHPTAASMLPQQPPLSHFPQSRPPFPPIPSPASLRAQNGYTAPVQQDTPQRAVSVQQQQPPSTFSVPNFDGTAASRVSPYRLPPQSTNGDQSAQWNYAAVPQNEMSRTAEKGQGQESSKEEKHQKARAAITDFISAGITYQNLIDEGIKPPVLNQLFAELGLDRHTTLLAPNQPGQDSPSASHPLVQTTPSEQPPDTRQATATPQPAVLDPAMERKDRIARLLAMKKGQAIPSAKSSQSASPAPVDASPRPIPRPAPVLAQTTPARTPQQLAVNTTETVDTDPQQSTAPQPLSAGAVENAEKRFNSLPTPVREEIRQHGFSIPGLFLTSADEALEPEPAATTPAPAEPKSLKRSAEPFATDAMPHAKRQVSQVVDTPQEVPSASEKVMVDPADVTVSSPLAKQISEPKDLPASSDSDKPVDTGIPASRSKINQDKLKNRMAALRADLMRKNTRKKALQDGMPELAAEVERTRERLLEQQTRLSVVRKDIETKNSELAQARDEEDRLLQEIRRLENQLADGESGQKQFTNELTQLNDQIVADTNESILSPQATARSSPQAEITEPRSEKVPQAVPMDVGIEVVSTTEALDEQLEFRNLAGQSLGGGEAHSRLPSHDQQRTETDYSPEQEDLDRQLNFQIASSAARDQDMANASEDNLSKEASLEPNLAADVLPQTQISNATTPHVDQSVDNSDDDRMSIDETSDAGSDGSASMSDGSDDYEPAAEMQDVQTVPPEENDDYDPENPVQCQQPLETENDDYEPAEEVDVLNVDSLNGLRKEPDIPAEAVQVSQADEDVDRSSRVQSIGAIALNGSTISDRAGANDQELPVTTSGPTTDDERLPLQQPTQPTSDVLQPERIDTLPEEEPPVVPRFKPYESPLSTLKSFRFHPQFNEHVKDGYRSLTYSNRIDAGKPLCPTELEGQMCTDVKCEEQHFGGMALSDDKILVQMSSANDIKEKDARDAYLLGLKKVIAGLRAQGVKEFSEVATALSKYRRDWMATQDGLVT

>CF317_000295-T1 CF317_000295

MLLHEVSPSAPSFSNAHGNGQRNHSYSRHNSPTPVPVPVPITVDMSQTTPLSSTAQQKQARFASPNAISFSRQPSQSPASNFSPIPSHVRSNSMQRTASSSTFAPQFVKSEEFRRSEDRVSAIEGENDFSGKRYVWVKDAEKAFVRGWIVEELSQQTVLVQFESGSQLELDLDDVDMVNPAKFDKADDMAELTHLNEASVIHNLQTRYQSDLIYTYSGLFLVAINPYCPLPIYGNDYIRMYKGQTREDTRPHIFAVADAAFRRLVEEGENQSILVTGESGAGKTENTKKVIQYLAAVATSDLDTPLAGRSPAKQLSNLSQQILRANPILESFGNAQTVRNNNSSRFGKFIRIQFTRSGQIAGAFIDWYLLEKSRVVKVSQQERSYHVFYQMLAGADKRLRDALLLSGLDIEDFAYLRGGNDTIGGVSDRDEWNHLIEAFHVMGFTEQEQHAIFRTIAAVLHIGNVAAKQESRATDQAMLTAEAKASLDRACRLLGVQTDPFIKGLLHPKVKAGREWVEKVQTAEQVRFALDALAKGIYEREFGDLVSRINKRLDRGGLTSDDNHFIGVLDIAGFEIFDNNSFEQLCINYTNEKLQQFFNHYMFVLEQEEYAREQIEWQFIDFGKDLQPTIDLIELPNPIGIFSCLDEDSVMPKATDKSFTDKLHSLWEKKTPKYSAARPRQGFILTHYAAEVEYSTEGWLEKNKDPLNDNLTRLLAASKDDHVSTLFSDCVDESDELYSPRSRVKKGLFRTVAQRHKEQLTSLMRQLHSTQPHFVRCILPNHKKKAKQFSAPLVLDQLRCNGVLEGIRIARTGFPNRLTFAEFRSRYEVLCPNMPKGYLGGQEAAKMILDRLKLDRSVFRVGITKVFFRAGVLADLEEQRDTLIREIMSRFQSVARGFMQRRIAYKQLYRAEATRVIQRNLNVYLDLQANPWWRLFVRLRPLLGATRTASEVKKRDERIDQLHQKMKEDQLERQRIEEERRRAELNVHQIQQTLEAERALALDKEEIFKRLQLRENELSEKLAEAIVEQEELEDQLDAAVDSKRQGEAELVTRRDQVIQAGQIITRLESEKKDLQKQIEDLNDDLVHAERDTSKVDGLDQEVRSLRSQLGVRDRKIHELESNHSQADREKDTKLSGLEAELRSLRSQLSQKDRRADELEKKLESMQRITNEEKERNTNSYESQIRSLNSQLGTKDRKLGDLEDLVVKKDQRVQDVEARLLAANKELTGLRSQVQDLEADNDDLQAQFDNMSSSKQHHETVLRQKDNEIATTRRQLQDFEAQMDKLQIENKSLKQRHEDAAKKHGNAEREMEEVRRKRAQMDRDAIEARRKLEAIIPFFNSHFGSSM

>CF317_000296-T1 CF317_000296

MPLSSGGDKSTRQSSATKSFLRQKLGRSGKDTDSRYEPSIPDASANASWNSRHSRHESVASINSDGQQGNVGLNMNAGVMTSIPYNSGTTDSRSSPSHDPYAQVPREDPQPYQLARGTDYHQYPAFAGSAMPQNSGHSVAPPRPPPHQGMNNLTMASSQPGDRGTKLQQWGPPRPGSSNTNHSTYDSFSTADSSDRPRKSLDAMSIRSTASSQTANSVFSSEISARTARPSASESRDSMSVLSPTHSRLGKIASHAGWPAQQASPFSSTTSFAPAGFNLPRPKDDKEVEKMFVELMHKRGWQNLPEAARRQMLAYAPAKKWTLVHQDKLTEWQGELKRRQQSRQTDTPLTRPDEEGSPEWYVKKIVDDTISPKQLQSLSVSLRTQPILWVRAFIDAQGQIALTNVLMKINRRQQAGPAPAHSVMSDEKALDKEYDIVKCLKALMNNRFGADDALSTKTVPIALATCLTSPRLQTRKLVSEVLTYLCHCTESEGHVKVLQAMDYVKGMVGENGRFDAWMRLCEVTVDGRGKMGSLVGASDEVRGGGVGMENQLMEYALATLILVNTIVDAPEHDVNLRHGIRTQFHSCGIKRILTKMESFQYELIDRQIEKFRENEIIDYEDLLQREGQSQVDGVQPETKDMTDPKEIADAIMTKVQGTRTQDHFVSAMQHMLLMRENPYEDRLRMFQLVDSMLSYVAMDRRLPDMDLKQSLNFTVQSLLDKLHTDAEARLAFDEATEARQIADAALAERDEMKAQVEMGADGLIKKLQKQIEEQQGIIDLQSRQNESLKSELADLQRIRGQELQRNELETRELYLMLRDAQDIAASNANKISVPGDPARAQGILDRERLMERLEKQLERTKTQFKLEGKVWEQQGPSDRLRELREQMDDATDADFQEKTRQHLTSSVIGSVHRSNASRRSIRNPRRAAADALLENEEDDLEDDMEDAVIEKPRVVELVRPKVTSQQRDDRAGMLSELQSKFRKGTSVDGTSEDGTEGDGVTTGTSHPSLDADSPKTPLDEVMHDKSKLEFTGPPPPPPPPMPPTAGAPLMPGFEGGPPPPPPPPPMAGSGASSPLMPGFATGAPPPPPPPPPPGMPSSQFGGPPPPPPPPGAPPLPGASHGHFLPRNMSPGLPQIHSSFIRPKKKLKAFHWDKVDTPEVTVWSSQADLAAKEAKYQELARKGVLDEVEKLFSAKDIKIIGRGKEAKEDKKSVISSDMRKNYHVALAKFKQDSADELVKKIIQCDREILDSAVVMDFLQRDDLCNIPDNVAKLMAPYSKDWTGPDAASSQREQDPAELTREDQIYLQTAYELHHYWKARMRALALTRNYEAEYDEISKKLKEVVDVSESIRDSTSLMSVLALILDIGNYMNDANKQASGFKLSSLARLGMVKDDKNESTFADLIERIVRNQYSQWEVFVEDISGVVPAAKLNVDQLRQDAKKYIDNIKNIQQSLDMGNLSDPKKFHPQDRVAQVVQRSMKDARRKAEQLGLHLEETTRIFDDIMTYFGEDNNDENARREFFAKLAGFVNEWKKSREKNIVQEDNRRKMEASIARKRAAVNNAAERAGATGDGTQTPTNANSGAMDSLLEKLKAAGPGKDQRDRRRRARLKDRHQDRVASGAQLPEVRVKDGLEESRGIAPLSEGELETPGGNDTPAKRGNISEGEDVADRAASLLAGLRSKASEDSSDGISVRRRRENADEERRARRARRRTQQAKGSEMGDRAMSPDPEDEAATISGQVNGKQDSSDETGSPVLYSTALPTPSIIVSPTPDDEVDYLQSRQGHPEEGSRERPVEIPE

>CF317_000297-T1 CF317_000297

MQRQPFPPPNAKSPELFHPRPQHISQVPHLRSPPLPNQPQQQPPVNSYGNPYGSPPMQQAQGGMENAYGQQYVNGFMNDPNAQMGLQIGKSAMMTGAHYMEQNLNRYVSVSALKHYFNVSNSYVVRKLLLVLLPWRHRPWTRQQTRLHAAHDPSAPQYSNMYLPPRDDLNSPDMYIPIMSLITYILLSTVLAGIRGTFRPEILGSITTTSVVVIVLELLILRTAIYLLNITNESSLLDLAAYAGYKFVGVIVTLFLSEVFTGGRGTNNWIGWAVFLYTWNANSFFLLRSLKYDTNASILLVEAGPDCKDNENIKMTGGWSKNFDAETDWNLVTPPMAGVNNRQVKLSRGKFLGGSSGVNGTLCIWGTEQDYDDWDLPGWSGKEMFTYMRKSENFHTKDWFEDDVNAHGYDGPLHVEPHDLAPISELMLKSMESQGLPLDHDMFTNGNRSHGCGHATRTVHKGLRSTGADFVTDDYHRQNIDIVVDTLVDKVNFDDDKRAVSVDLVGKSGEKSTVGAKKEIIVSGGAYCTPAVLMRSGVGPKEELQQLGVDCLVDSPGVGKNLLDHIIVFTFYEVMKDGLTNDHLVYHHPNAAMEAYMLYKEKQTGVLSTFPFGAFAFARLDDRLKDEPLWQEAQEKAQPGRDPMDLTTSQPHIEFFTTELYGGPKQYDQYPIDKKSAFAMITELFSPRSKGTVSIKSTDPFENPVVDCNYLADPLDLLVMTEGVRFGNEIVMQGAGTKDVVKGSWPADLTHHGYTKREEWIPHVKDQATTCYHAAGTCKMGKEDDAMAVLDAQLRVKGVTGLRVADCSVMPTLHGGHTQMPAYGIGEKAADLIKAAA

>CF317_000298-T1 CF317_000298

MLESFEIFSTSGVVLWSRGSSNAAASAVNSLINDVFIEEKSSKSTSFQHDKFTLRWTLVKELGVIFVAVYQSLLHLSWVDQFLDEVKTVFIKRYSEQLEAGATVAKYDFDGYYDELLRRQDQITGGEATRSPRVAIQASKQNPEERDNGGPPPPSRPDLLRAQSQTAPVNGDSQDTTPLQSPETSRPTTPLPGVAPHLLTENRPGSRGSRRARKAANATPGSNYASSGDESRRPKTPKGAAKKGRVWDADGMAAEDDGTVLDYSNVADADATNGTQPSMESIAQESWGTKNKQGQFVLKDLDDEVSKILKESGSKVTEDGTPTSGAFGMISGYFRNMVGGKTLTKEDLDKPLKAMEEHLIKKNVAREAAIRLCEGVEAEMLGKKTAAFESTDKALQPALENSLRKLLTPKTSLDLLQEIESVTKGGRSGGRPYVISVVGVNGVGKSTNLSKICYFLLQNNYRVLVVAGDTFRSGAVEQLAVHVRNLKELSARENVGRVDLYEKGYGKDAANIAKDAVAFAGVSDFNVVLIDTAGRAHNNTQLMSALEKFGDFAKPDKILQVAEALVGNDSVNQARNFNKAFGRNRSLDGFIISKCDTVGDQIGTIVSMVAATGIPVVFLGVGQHYGDLRQLSIPWAVKLLMN

>CF317_000299-T1 CF317_000299

MKTSTFLLVASLAAPSSAIPMMNTTVRFNVASEDASNVGSSDYSVLQFALTLEHLEAAFYSEVAAKFSAADFSSFNIPDLYTDVLRLARDEAVHVDVLTSTLRSLQKPAPSPCKYTFPYTSAASFLALAAVIEGVGVSAYAGASTSLRDPAVLSMAAGILSVEARHDAILRAASGIAPYASPFDAPLDFNQAWTLASQFIVPGSCPDDYAPFHLTTFPKLALTGPSTGAPLQAGQSITLTASADEPLEKKRHAQLHRRHGAGGARARRHAAHVHDRRIDGNVYAAFLTVQGAVLVEAVQNGDSVSTTVPDGVAGQVYVLLVRGDGGVTDANTLAGPAVLEVADAVGSGATEDEGEGIVERC

>CF317_000300-T1 CF317_000300

MSELLCPNRIDEILSQRLRDDSIDEPDIFTAPATYLQNLNELAPFAHRHKLFFDRFSKVTKELLVAVSEYNYEDFESQKDLPFAFSAYVVVEEAKNMFSQEKITVEDLEDKLPDVSEADRNVRGVFCCVVYHSGYVYVYIGSSGNLLARIVLHDRRIRHGDTSITLYRTLSGLGTWNYRWKTLARFPYGAHQSFSYLLEAICMVVFQSIDNSMNRRFHNGPSRLMYSSLMGDQDDKDCDTAFEFETQSINHQSPLLAGCKNDLTPKQWYFAPGRPFEAYICVACYLWNDNKGTSNKRKRDETDQQSVEAKHDQKRVRGVIKQGSNPPDCDWCGKNVHLPAGINSNGNKRDKRATILNDQVALCLCKKCHDQTFRNHALPNPAIVKGCKCAQCSAEDDLEFVPYPVKKFMCGGCRGGAETVYNQLVALTKTDHPSRTKHYHKLIAKIQQDWSLIHVELRDETLLSGSHDAFTPIKLLRKGGKTVHKLDDREEY

>CF317_000302-T1 CF317_000302

MELAYTITGVALYLSIAVSRSLDVLVVRLFDSPHHAAACPVGDFPLNGKIVLVTGGGSGIGLAFTQLCCEAGARVVIADLKLTDEGQEFVNSAKQDQVFFQKCDVSSWPALHDSISASVKAFGEVPDVYAPVAGIFEPKWSNYWDDLDEKSYKTMAINVEHPIKLTRLAMRALAGAQKKGVVCLVASTAGIRGNYLASLYSASKHAIVGFTKSMGQADPEEGVRIVCICPGLVDSPLWRDREDDMLDSAKFNERKAIFPNDIAKVMIKMVESKEYSGGTCVLKTPLEERVCEQGWPASAEGYDPSPRPEADNRRIHAAMDKERGVAWQ

>CF317_000303-T1 CF317_000303

MENELPTTSTGSTSYGSGYRSGSVHVTPGSNSASSKSSDSNTAAVSGSPASAHSLESSEEQAADAGEHQRGATGHGRLSNVSIHDLAESVSESTAALKDSPLSDSPPLSPRSIGSLELVEARDGQKEQCIRVKCKQLPSRYSVLSVQQTANELLYQDKDYLPDGPQNYLRIPTSLIENANGTDYVRTMTTIVDFVKEVESRRARQIAELERAASRRAQLDAMRWLKEKVFLTFLALFCLSISMLGRPGKDNMFILLVSAIAFTSMVAAISGTVRDSCQRYFDDVATCFADIWHAIERLTVALCAMTFVQWHTARVSRAQGLLLAFFRTFNWLDKTAKKMSSLIYNFRYGFLVTLALTNLIYWFGSLTRCTLAASLRLSCSKLYNAGTTMQLRQNYLSGVCEVMNQLTIDSIPPMAGYLEQGRMIILGSVGALVALSAVGYRAVQRWQRRTNPNTSGFMYNCIVKLVSTVEEVQRTLVATEHRLKSMLFATARYFRKHWFAISVLIMINLSTVDYAFNSGVQAKMAEITTSMPVRLYDPKTLYPDVQFCILAIIASPLLRRIVFPLVDLICQGLLDTLRAIFVVAWRWPWKMIVRCVVGLVLGTVWLAGTITLGIWGFHAFIGPMPTNGPVPVLDMLVQYPNGGEAQPVRIIR

>CF317_000304-T1 CF317_000304

MHAMLALSSAHQEVVFDPNCRPLRDTSHQELLTIRQYSKAIEYLQPHFSDRSRASICLTVITCMVFVTLEFLRGNYTIGMKHLQHGLNLLQEAARIVGLESGGSAQSTVCFDDQIVTLFTRILVQARLLGQSITSPHDPFLYNRSSLELRVFGSPDHARRTLEPLLLRIFDLEERRICRDEITTASRNSQQLVQNELLEWTQIYQTTMASLPDTTSPLEAFAYRLLELYQTIATIMADVCVDTNPEMAYARHTSDFVSIISHCIQSYSIVLQSKGLESTHAYDGGNPNANSISDIGWIPPLYYTGVKCRNHRVRLHAVRLLEAAPHKEGIWDAKLAAAVARKIIAVEEQRLYINCADDFDLLAAPTAQELLLLPVLPEAHMLHELKVALPDTPAGRLVLRCNRRESGLWEAMVWVYDLPSELWSHASGWSKCWSTCA

>CF317_000305-T1 CF317_000305

MEVNRYRTRGEAEDDRDLVMPYRIIDDQLYDEVDTKQRQQNREKYLVKRSIRHHRHATTKWGAALESFRPMWFAICISSSGIGLVLNGPFVYRAHWQITIATIMYIVSLITFFIFLGLQITRWVVFPHVAVRRAMSDPDELGAYAIPPIALMTLASLTIMQVSTSWGGYAFMMLGYVLWWIGVVWVFITAVVVLCTLIYTGSQSGRNMSPVLFMAPVGLATAGAEAGFITIFGNGEGQMSPRLAVPMIVVGYFALGIAFFMALLLYTIYFHRLLSAGWSAPAKRAGLFILIGPAGQLSTALQLLGESANWFTNLADYKPPAAFQPPTRGTFFTAGTARGIDGAGILLALLLLGFDYLMFCIATVGVVDIFIRRQASYSLTWWSIVFPTVTLTTAWLELSFSMDSPAFRVLTCIMTVFLTIVYCVNLCFTLRGIFNGSLIFAQTELQMEDGVMKKVNEGKDKEEAV

>CF317_000306-T1 CF317_000306

MYAKMAAVFLGCSIGGPLIMYYVTPSEGELFKRFSPDLQKRNLEMRPEREKNYKDFAAKMIEYSKSDKPIWAAEEDARQKVREEIMSRESAERKVREERAAEMRAEMQAAKAR

>CF317_000307-T1 CF317_000307

MRLQICSLGALALSTQHAYAALTVDLTNPESIKQAASQAAAGMLTHYTGDNPGDVPGNLPEPYYWWEAGAMFGALIDYWYFTGDDQYNNITMQAMTHQLGPDNVYMPPNQTKSLGNDDQAFWTIAALSAAENKFPNPPADTQASWLGIAQGVFNLQAARWNTETCGGGLKWQIFSFNGGYNYKNSISNGCFFNIGARLAVYTGNDTYADWAERVWDWSERVLMDEHYNIFDGSDDTLNCTELDHLQWSYNVGAYLAGAANMYNYTDGSEIWRERIMGIIGNLGLFLKDNVMYEQACEPIGTCNIDQRSFKAYFARWMAATMVRAPFTYDLLKPILEASAKAAVSTCTGGPDGNQCGLQWTQGVYDGSTGAGEQMAVLEVLQSNLVEYVAPPATLENGISEQDPSAGTSSPVGPGDLNKQTVTDTDKAGAAILTIVVCVMVMGTMLWLML

>CF317_000308-T1 CF317_000308

MADGRQEPYDPYLPSTGNAQGGNARTAALQAQIDDTVGVMRENINKVSQRGERLDSLQDKTDNLAVSAQGFRRGANRVRKQMWWKDMKMRVCIIVGIIILLVIIIVPAVIATKNG

>CF317_000309-T1 CF317_000309

MNSVTMSTFSAAAVRAASRSLAITRPRTASALRAARPAIPACSRLTSLSQPQRSYATSSDRASTRSTVVQLLSNIGSKREVQQYLSHFSSVSSQQFAVIKVGGAILTEHLQTLSSALAFLNHVGLYPVVVHGAGPQLNKILEDSGVEPQFEEGIRVTDPKTLGIARNLFLEENLKLVEELERLGVRARPITSGVFTADYLNKEKYDLVGKITSVDKKPIEAAIQAGCLPILTSMAESTSGQVLNVNADVAAGELARALQPLKIVYLSEKGGLFNGDTKEKISAINLDEEYDHLMTQWWVRHGTRLKIREMKDLLMDLPKTSSVAIIHPADLQKELFTDSGAGTLIRRGNKVHTASKISDFSDLAKLKDVLVREREGLDAKAVVDRYVKELQDQEFKAYFDEPMEAFSIVLPPGRGSSMAHLSTLTISKAGWLTNVADNIFAAIKKDYPKLMWTVKEDDENLTWFFDKADGSLSKDGEVLFWYGLRTGEEVQEVMMQFSEHGRSMFGDTNLESKLQRAARAAASAMSNVAKSAGIQQARGYSTSANPLRAARKTFASAQPALKIRTYATTTNPNPPLGSKNKSNDGPSRVALIGARGYTGKALIDLLSRHPNMDLRHVSSRELAGQKLQGYKKRDITYENLSVEDVRQMEEDGKIDCWVMALPNGVCKPFVDAIDQVGGNSVVIDLSADYRFDDKWTYGLPELVDRSAIAQATRISNPGCYATAAQIGIAPIVPYLGGQPTVFGVSGYSGAGTKPSPKNDVNNLTDNILAYSMTDHIHEKEISTQLGTPVGFVPHVAVWFQGIHHTINIPLKEEMTSRDIRNIYQERYAGERLLKVIGEAPVVKNISGKHGVEVGGFAVHSSGKRVVVCATIDNLLKGAATQCLQNMNLALGYNEFEGIPLE

>CF317_000310-T1 CF317_000310

MRGISTLLKELGTDPEVVAIRPEQLNLPTDLAFNMDLDLGFDLSSFDTTFSSSEPSSLLSPRTLASTQTSFLEDEQMLEPRPPLELSSSHDAGEGFGFDAGEEFGFDALQPASGAAGQQPMSKVAGELAIGEQPSVIEEAAFEVDEEGNVTFRDEEDRPPAIDQSASNGLMGEYQDIFELNFDADHTEARDTQTPLPVVDDEHQIYFDDDDEPQFGQELPPQTPAARSSPSTHQAQRSIEGDGDVEDGPEETNAISGAAQQRATKTKTVPVDNPPELQNRVLNGWNNNYLDFMEEAHTENASKISMAQAKRNAAYWVLDQGIGGVDSDFRGDNREHPLAVFSGQALLDALLGSQAQSPSRKRARSANGDDGDDGDGMTSRRIRSSPPRQEGIGLGVEDDGLQTMADDDRGLTLGDDEFEPEVGRHEQPPMSEHQDSFPWNAYASSKQGSRHGSIRPAMSIAGATSSAGGRAGFDLVPSSMGSKRVSRLIAESPLEQRRRLLRQSSVLSGSRQAQEHTGLGSADDDFGLDLDISDTALDRELAGNLANADDFELYGPGAAVSTQQAADSQWLAATLEQEAFNFLGFLQTTIEGKQADLDSNDDKENVTVTFEELLPPENNSEVVAAQGLLHVLSLATKGLITVKQEKDFGVIDMRIVPGLAAAEPEEVVEE

>CF317_000311-T1 CF317_000311

MASHKVDILESLGLTSQSAELERRLTAIEYTVQVQRQLRSTLTRLQVPRQGFTDWRFSLRKFDGDTKQTRQAQQQGEEALVLLAKIDKNICNLEEELMSADCAQIDSNELLADVVELDVAVRDLIRLFPGARRAGLFSIDQSGPTSSCARAQPPTRDRRRPLPPLTPTEEAPLEEVGDTVPVLQPRQSTLASSPSPRRTSSGNGHVYRHVTSDNQAQAVLGTFYAASVLAHSGPLPQIPANEYNDILAKGNSKMLMGDAVGGSPFVETAEPRNTTPTQGLIRRFTDKMKRKESVR

>CF317_000312-T1 CF317_000312

MQRPPGPPQPASPPRKTLVTIQELNDLRIAFRDNPKDDKLGLKFAKRLVEAATVLANEGGKADAKTAASNRERYINDAHKIVKKLVASGSPDAMFYLADCYGQGALGLEVNPKEAFQLYSSAAKTGHAQSAYRVAVCCELGQDGGGGTRRDHVRAVQFYKRAATLGDGPAMFKMGMIMLKGLLGQQPNRREGVSWLKRAAERADEDNPHALHELAMLHESAQAADIVVRDEQYALQLFTQSANLGYKYSQHRLGSAYEYGTLGVQIDPRQSIAWYSRAAQQGEHQSEFALSGWYLTGSEPLLAQSDTEAYLWARKAAASGLAKAEYAMGYYTEVGIGCQPNLEEARKWYFRAAGKSNPM

>CF317_000313-T1 CF317_000313

MAYNGQRGGYQEVQHRSTPQGFNTQYNGYDQARYDQGDQGHGAYQQDAYDSNQQFTQTYDGNQEQYGNRPQNNHDMNNYQYAQAGQQQQQQQQQQQQQQQRQQPRQYQYDNRYRTNGRAVQEPGNAGRMMKQERILDTARRESLNAMAWDNPFGVFPGTKKDVEKKKKEADQATSHNKVDTPAERPTTSSSSRKPTFDQFGPFNTPARTSLEEPRRPHTSHARHPQSPPEQSPYVNQPLGPLDDMPLRKRFSPPYQGQRSHAGQYPQPYSPPVMFNGRKGPPARPGPQTSAIEPLVNHQHTSFRSGPTPLDNGQMALQRQDGYGNVHSQSHEFPDHNGPQEHLYELPDSQSQQANIDGNEPDARQRGSRTEPLYQQEQPYELNGRKGSVPQEHAELAYRGVEPTGPLPSTSEPAMPNFAAIAPGVTENVEREVTLQQVPRAPAYRKPTYGDSASRPPPVRTGLVAPPVQQDPSGITVQSPIADFDFGLPPGNLVQATMIRPHGNIDNCLIPFRMEELNLRNKNFLLEVPVDQVCGLNNPLTAIHILTTSPTHVSRNPNPDSRDRDSLRSKIQAFIRLVGPHAKTMASISADTSRTTPTTGLLEAVATRKPRLRTNLISNHDL

>CF317_000314-T1 CF317_000314

MFDALLRALIARSIATTLGLLVAVWNLVSLWHLVQWRLRIRHLRAQGLPVPPHSFFLGHLPIIKQVMGQLPKDAHGVYLANEIMRTYPELGPNCYLDLMPFAPSMLVTGTPETHHQIAQEHLLHKLPNLKNFVRPIDDGLGMLTILWRVPNGRNGEAYSIPVSVQLI

>CF317_000315-T1 CF317_000315

MKSLTDYLTLDIIGRIVLGVHFDCQRQLNAVMESFRTQICWFTFGAEPNLADRYNPIRPLVQWYHSKIVYDYISEQFDQRLAMQKAGGSADLRNTRTIIDLATTAYSSDESESTVGAASDPFFKRVCMAQIKLFLFSGFETTSTATCYHLYLLAQYPEVMARVRKEHDAVFGSDTSATASMISSSPHLLNQLPLTTAVIKESLRLYSTVTPSRQGERGFCIVDKEGRSFPTADFMVFPCTLSIHNDPLFWPQPGAFIPDRFLVEAGSPSPPSQGCIARL

>CF317_000316-T1 CF317_000316

MTSSKGEPDNPELREKVKEEVKQMSKGGGPGSWSAWKAGELARRYEAQGGGYKDKGDHKNKSQQGAPEPKDPKDKKESAAEEAHREKEEREKKESHDGKKEEGKKDEGKKEDSKGSSGKKGGNKKK

>CF317_000317-T1 CF317_000317

MSARAGLRFFTQRAQFQARSQFRQTAQRRSQTTARNPATEPGAAAQQSLFQRLWTSEVGIKTVHFWAPVMKWGVVAAGASDFLRPAEKLSLSQNLALMATGSIWTRWCFVIRPKNMLLAGVNFCLFLVGTIQTSRILAYQSAQKGSLEGGAKELEQVVKDDAKRVEQQAETAGKELKQKA

>CF317_000318-T1 CF317_000318

MATKYAFTQGLRELRFHLSPSGSGSDAIRTFLRRAYPTMKHHNPNIPILIREANGIEPKLWARYAGAKEKQQSLAGMSDKEIEEKVTGIVKSEFS

>CF317_000319-T1 CF317_000319

MRRTPVYFVSHGGPTTMYEKDHPVYSKLEEIGREITHKVKPSAIVVFSAHWQAERPNTIEVNVSENEPLLYDFYGFPKHYYMEKFPNKGSPQLARRVMELLGEAGIQALPEERGLDHGVFVPFKIMFSPEKNPLSIPIVQVSLFDDDTDAAAHIRLGRAVQKLREENVQVIVSGMAVHNLRDLWMTRSTGQTMPYSVSFDAAMKEAVETVPGADRDKAMESLLRRPDSRKAHPSFEHLLPIHVGVGAAGNDTGRQLWTLPEGSMSWGQYRFGEVEA

>CF317_000320-T1 CF317_000320

MNMSNGDDISPGSGKNHHNGLTSPPGQQKLGRDPSHLRALSVGKPPNLLSTTHPTLLSLKDNSHSSPEQSPAHTDFSWKEGNAPVCSVTSLESDHKTRAQAEETVRNPDRRVSIAEPTLEAQFPNPIVSRQSIAERQTEHARVRKAEVKILLENYLPGYLFTVNPGLLKNPKGKPHPTLLDQEVSKAIQSAEINLYLSHITDIGKKIQFLQEYQAIKDKYEGKNSRVAEDMKKYAHTKPMLKEVIVDGKPVKQQVTHTEMKTYKNKDGKERIKHKAGDIKWNAKELVPLGEELLKEKDDKIEELKSKLNRVKASCEDVNV

>CF317_000321-T1 CF317_000321

MAPLSVFLRDTGPVINTSGEADTWNWRKVAGDEDLILESWSQGFIVGALMLMSCITLANMRHHVILHKLIFLEQIMALSHGTFCFMDFKGYGWYLSTTAALLYLSYILHNVVAWLKVKPFFHGKSTIFKPRFVKWTTRIYTFSLVCTVPPILFQISDNFRFFNGYGGWYTKVRPYEPLTRDPWWVFCSFILFYVISKSYGMGVIQVIKRSPRFGILFVSIILALLFTALDIVASIHSFIGSTDGINPFWKLSLVFKCLTDAILLDDFKTELKRLNLKRMKRDEKRRESTALVLGDEYALDSDDEADPHYSNPAANRYMNGHAYRLSVSNHSQEKSNGNSNIEEPEEVEEVNFLQALDTHPSQLSSDRNSRRSSGQRHQVCRGGTPATRLPKLFAAIKPGRKSKKSADEDTSPNEGLFAPLGDGLGNKKQKKRSRDDGDIAPDEVTCGDDALSQAKREQERTIAELTAGKNNTTTPETTGHGPSNAPREESLLSDSSSMLSSLDTQNNGNDAQPASSESLPADSMLKGLSTQLPRNSSATVDMGSVSTSSVMERRSNATPHRGSISTAKNTADLIRQHVQQQKKSSPGGDFWNDLHDTNEGRSSKQHQS

>CF317_000322-T1 CF317_000322

MTQRVLIYLLRRDLRVADNPILHEISRLANQSHTPFTHILPVYIFSSQQIEVGGFISDPSKQSPYPEARSEVGKFWRCGPHRVRFLAQSLWDTKSDLQSKGSDLVLRAGMLGDVLEDLLSSFKTKENDVQIAGIWMTGEEGVEEKQEEQRARKIAEKRGIEFRLWIDEKYFVDDRDVPFDNPRNYPDVFTSYRKAVEPLRDAPRKTLSTPKSLPALPDFVPPQKEPFSMPEDYDGVVAALLKPLEQKPMLDNIAPFPKGTKSAMPFEGGSKAGHKRIQHLIGSGSMTKYKDTRNGLLGHDFSTKLSAWLALGCITARQVHFQLLDFEEGRNDQYKGTEGYGKGENKGTAAVRFELLWRDYMRLCTRKFGPRLFRIEGFRNDDSYPWKTPGKDNPEVQEALQRFLNGTTGMGFIDASQRELYHTGYTSNRARQNVASYLAKHLGINWKLGAEWYECQLSDYDLSNNWGNWQYVAGVGNDPRGEARVFNPVKQAFDYDNQGDYVKAWVEELRELSEPQEIFQVHKASPEKVKELGLEGNVMVDKPLKKIDFRVGAKGGRGGGKYGGSSRGGARGRGRGGNHNQVNGKHERKGTQDKATQVNGYQPIPVR

>CF317_000323-T1 CF317_000323

MANNILANTSLSGILAAAAVLLLIKRVYWELTIGAQRWRLIKENGCKPVRKYPHKGVSGKLFGYDTLKQNFQAAKEGRFHEMARLRNFGPQQHTIQIRNLNRDFVVTIEPENVKAVLSTKFNDFSLGTLRTLTMEPVFGNGIFTSDGKAWEHSRALIRPSFTRQQVGDLSTYEHHFQNMLTHIPKDGQTVDLQELFFRLTMDSATEYLFGKSTNTLVKGHENPRAERFADAFTYVTERMSRDFRTARLSRFLPDQKRKDDSEFIRNFAREIIDDALANQKDIERGAEQEKRSYTFLYELLKVTNDPYTLQSETLNVLLAGRDTTASLLAHTFHELARRPDVWAKLQAEVEELGGQAPEYETLKSMKYVKWVINESLRLRPVVPGNTRMAIRDTVLPLGGGPDQKSPIFVPKGTPVTYSVWSMHRRKDFFGEDALEFKPERWEKLRPGWEYLPFNGGPRICIGKLNFLTETGSCTRRSHAYSYMLFDFA

>CF317_000324-T1 CF317_000324

MSWFHVKPMQPFFSKMFRQKTLDVITLFHKPSLSSSNRVLTLLKQGSSQAQETATVDQAADHAPQNDAVQREPFELDVTESAPTEDQLRSIFEYVGQHKISSVVEGATSVSDAVKKLAADEGAFKRPLVVDWNQGRAVVGDNESEIQKLLKDAPNQ

>CF317_000325-T1 CF317_000325

MAGLARHVDPEEIIQKLKISDDKAHHHRLRAGDSNDGSSLTPYSSRYNARDSISKFKIPQDGAPAEAVHQMIKDELDLDGRPNLNLASFVGTYMEREAEALMIENISKNMSDADEYPAMMDIHARCISIIGNMWGAQKGEHPIGSATTGSSEAIHLGGLAMKRRWQEKREKEGKDKSKPNIIMGANAQVALEKFARYFEVEARILPVSKKSHYRLDPDLVKENIDENTIGVFVILGSTYTGHYEPVEEVSKVLDDYEAKTGNHIPIHVDAASGGFIAPFTHAKAGFKWNFENPRVCSINTSGHKFGLVYAGVGWILWRDEAYLPQYLKFELHYLGGTEESYTLNFSRPGAQIIAQYYNLIHLGFNGFRAVMENALANARLLSKSLEATGWYVCISDIHRRKGQFDFDPTQDVWHKEGETSADYNAGLPVVAFSFSDQFKKDYPHIKQESVSNLLRAKQYIIPNYPLPPNEEQIEILRVVVRESMSLDLLDRLITDICAVTQQMMDTDLTDLHVWQPGQTSLEKQHQSLGHNAHHRKKHGSQRPMSEGVHRSVC

>CF317_000326-T1 CF317_000326

MPSSLTQRRQAASNLPAFELPPPSHFAPPHYKFPPLSSIHAQPLPAAINIGNLPTPPPQSANDTPNGGSHLPQHNMPVLPYTPTFWQNAGSTSGFNTGLTPQPWQNGQGFGGRQMYSPMSGNVARNDPNSPASGNGSAHPPPYETNGVQSYSHAPPLASPSSSVNQSTQHPSMSNSMYANQGRSHHQASPISPADGASRASSTPGLYAAMTSSAPPQPPYGYQNQPPVTQSPHSASAPPLTSSPSFHQGPIPQIPTHSSPFVKPPYPSYSLPAMPGPVLSNVNNPGSQMSLVGNMHAQMMPMHFNSGYAANPQLAYAQARANSPQQAANQDRPFKCDECPQSFNRNHDLKRHKRIHLAVKPFPCAHCDKSFSRKDALKRHILVKGCGKAGEQDMHAADGVKSESGSDNQDNSSMAMAA

>CF317_000327-T1 CF317_000327

MSDPILSPSQSHPLPPRPYSGNPLTNAAAMDSMSQSSTPVQSASKLRGGFELDDEDDHDVTIEDAQDDDVYDTIPTEVNGSASNGDQGALDRPSKSPTQEQENGMTPVPAQAIDSLTNVPSSAVSNIPQSHSVVTNGQESADQSSPADVLSVLPKTRLAHDVVGMLEDRIKDDPRGDSAAWLELIEEYKSRNKDDQVRRTYERYLEVFPLAAEQWCTFLRWEEAADRKWQMEQIFKKALISVPSVELFSIYVNYVRRRHSMQTGDTSQAYKNIHQTFEFALKSVGADKDSGNLWQEYINFLRAGPGTVGGSGWQDSQKMDTLRDAYGKALSVPTSAIQSLWKEYDAFETGINKINGRKMMQERSPDYMTARTAYTQLQNITKRIDRTTRPRLPPALGYAGDLEYQQQVSLWRDWLQWEKNDPLELKEDKLDAYLDRVIFTYKQALMALQFWPEMWYEAAEFCILNGRDETGLGLLNQGFTANPESALLAFKLADHLETTTSNDSTNDPGAKIRMKTVREPYDKVLDALYDVQKKTEGRMQAEIQRLELSISNGQADASGDEVLTASNVSEKKAAIDEQINAVRKAASSHIEVLSNLISHIWVALMRATRRIQGKGLPNEKGPSGFRTIFNEARKRGQLTSEFYVETAHIEWNCYRDPTGTRILERGVKLYPNDSLLPLEYIKHLIGKDDITNARAVFETTVNRFTSSNDPALVAKSKDLFLFFHDYESKYGELSQIVRLESRMRELFPEDPALKQFSSRYRTPGFDPMSAQPVISARQMVPKTALQPSIEVNTMVNSPMQKAIDQITSTNSPKRPLPIDDDDEDGRPQKIARGESPSMATQIRKIPQASVSARPPAPAPLPPAILHLLSILPKASAYTDIKFDANAIHKLIKEKHLPAPTTVGAARPPPTPQAGAAPAWPPQFSQAPPPPIIPPPQGYVPQGTPTSQYGANTMSSAKRRGQARGQAPDVGGSAPRRGPNPARGRVAPFDGPASRGSGSAAGTQSQVTGSAAGSRRGSNAGTQAPSQAGSVAGSQSQAAAQVAPIARDPAREGPVPRTTDPLRNVDMPASFYNIDNLYALPTEFTKRPGFNQTGKAIQLPVNSYEVTKLPQVTIYQYDVIIGNGAEKRIVQQKAWQSKARKDKVGPDCIYDGNKLAWSMKPYGQIKFMVDLDEEAGKGPSKEGKNAFRVHITPTKKLDLSVIMAYLQGRIQQNAAILEAITFLDHLLREGPSNNARLVPVRRSFFARDGQRADLGGAIEVFRGVYQSIRMAQGPKLVVNLDVANSCFWKPQQLMHTILQKNGWRDPSQIAQQFQRDDQVRATQKYLKKISVKAHYKGNKQPNTVWKIDSIAKVNANTHRIMWRDPQTGKETGEKISVAAYFARRYNLPLQFPNLPLIQMTKKMKGEPVYFPIELLVIIENQRYGAKLDETQTANMIKFAVSPPNQRLASINDGKSWLQWDTDKYLKEYGLQISKQQVVTAARVLPPPGVKFKNKTEQPGTKGRWDLRAKQFITPNTKELSSWGIGFFGGRVALNPGAIEKFAMDFVKAYRGHGGAVSNNKPFYMKLNDDPGQAVEQLFQATGNHFKARPQILIFLVQNRNAQHYLRIKKSCDKRFGVVSQVMQAAQVQKGNPQYYSNVLMKFNAKLGGATSQVVPARDSGFSGQFAVPTLFIGADVSHASPGSEQASMAAITVSYDRFGGRYAAGCQTNGHRVEMITESNMKSILGPLISNWMSEIGAGRVPGQVYYMRDGVSEGQFTKVIQEEVPHIRAVLDKLSNGKWGGKLTVVIASKRHHLRAFPKQGDGDQKGNPLPGTLIEKDITMPNEFDFFLYSHIALQGTSRPVHYTVLLDEANHTPAALQNMIYEHCYQYMRSTTSVSLHPAVYYAHLASNRAKAHENVAASQGPQGGAGFKQNQSASSDTPRSSEAAPLLTWPAGQRVEYSMWYI

>CF317_000328-T1 CF317_000328

MSIVSLLGVKIQNNPAKFTDTYQFEITFECLDQLQKDLEWKLTYVGSATSAEHDQELDSLLVGPVPMGINKFVFEADPPNLSRLPSSELLGVTVILLTCSYDGREFVRVGYYVNNEYDNEEMQNEPPTKPVVERIRRNILAEKPRVTRFAIKWDSEESAPAEYPPEQPEADTLDDDGVNYGAEEAEMQAALEKELAEEEARNKAEEDGDKAMVGTDEKEDDAVSETGSEDLEAESSSDDEEDEEEEGGEGDEDVEMGDGEEKAVVESNGQSRAVPPKQEVMAH

>CF317_000329-T1 CF317_000329

MAARGACSTTVRSVARTARPTTNPSPSLRLGPQTRAYHGARRQLQTRQAWAKPSTAVLAAAATVALIYTASNTNTHAETSKDDAAPVPIVEKTKKKKGLSKEQNRDLISSQHLQVKRSWENPGVYAWGSNTGKVAAPDSNEAYIKTPRRISWFDDVLLRDIKLDRQFGAAVLENGDLVQWGKGYSEETAEPTTTLRGKNLQQIAISRDRIIALSKNGTVYSVPASKAEQENGVKLSESSWLPGWSSTSDISYRILKPQGLSSKEKVSKLSGGLEHVLLLTNNGRLFSAASGSEDFPSRGQLGIPGLTWTTRPQGSFDQCHEISTLKGFEIEKIASGDHHSLALDKEGRVFSFGDNSSGQLGFEYSSEAPYVDMPSLLPISRMYQGTNQTPKVTSIAAGGLNSFFTVDATRVLGHNEDPSEVRTLGRITADTWSCGQGIKGALGNGRWTHIQGMPTRIPSLSGLFEYDEKSNSVVPIRLASISAGSTHASAVMSNITFLETSEKSSDDTTNWGADVVWWGGNEFFQLGTGKRNNVATPLYIRPLDTAAEVEAGRKDMHRFHVTPRHTVKVKGGRQVSMEQRIECGRNVTAVYSGV

>CF317_000330-T1 CF317_000330

MVSPDGLRDQSQSLPEVESELDLLQLDEDEILEASLNEYQRYLDELDAAKSHLDQLLASTTATLDQLADISKAFRSVDQQTSAFQAKSASILEEQRQNDRLATDLAENLKYYEPLERITRRMNAPGAGNFVRTQDFRDMLLTLDECIDYMQTHPKQKEAELYRSRYKQLLTRALTLVRNTFMAGARDVTDEVGKRIADKQANVNETLLSSLLYAKFRVDAPLMKDLGLEIQKRAVPPADAEPGTEGEYQSLMNELHQAFAACRIAAAPTAKGLVAFARSSITYVRGVCLDEFELWEEWFHGQRGVYDFLETLCEPLFDQLRPRIIHETKLSKLCSLVTLLQTRYLNADDEDDAPDPMQLDFASLIQPAIEDAQTRLVFRTLALLRDEIQYYKPAPEDLDYPHRVSEAPTPKSAAPLSGRKTSNIAAATEDDSGDEGTPNMWSRNRILDLSYPTLPRAIRLLSRIYRLVNSSVFDDLAHQIVHSTTHSLVNASNQISTKSSAVDAQLFLLRHLLLLKSQIVAFDIEYVSQDVQFDFSGMTSTFYELRDRGGLFNPRSWVRLFTSGGLIPRVVENMLDAKVELDGQLRTVINDFTAGFAKTMTNSLRTTSKPEDATAAVRDKVQKEVPILRAKLEQYLDDLRTRETLVAAVEDQVVQAYEAFFEEYAGKAAAGVKGKSNGSALGKTRISRKGKGAADDVWDADTFAEWGEAVFNVLLPQSYDENASPDTRSVSRSGSS

>CF317_000331-T1 CF317_000331

MSNADDTPDPVLPLTASDGGAWQVACSRTDVQAGLEQFLEVTNLLLEQVQLNSTHLFRADILADSASKLETLAEKEARCQAGAESDNGDASVQVAKDEVSSAVSARPPVIRGFELHRTVVRRLIPRKPQLDKPLEQSCFIYHDTASERESKKKQALVVYYPHCTTSDAMPWYHPKVQGLAYLYTDPSAVEASADTTPASALSVHYLPFPDTPSPLPDRLTRTFQSLLKTMIRLLKLPSKCPSQVLDRQLAKSFTASTTTTITTMPLTPSSLKDTILPQHTVQNTYTRLKQTYAADLISRWIEKTEPSKHVFEDLSIAAFLIELWTSMYSSKSTFNSTAGFVDIACGNGVLVYVLIKEGWKGWGFDARRRKTWDVLGDDVSACLHEKLCVPRPFSESMRTGEFEGVDTHDGVFAAGTFIVSNHADELTCWTPVLAALPDPGRPLPFLAIPCCSHALDGSRKRYTLKEVAESLEGADRVGELDNGEEGSIQPVVGDLKAMRAAKQQANAHGDDKSMYACLTRKTAALAQEVGMDVELTLMRIPSTRNIGVVGNRKTIYKEGNGKKALNADMDALSLAADAGTTAKKVRELLDREFASLGGANNAGRTWIEKAKKLHGGQGRGKVNLNGPVK

>CF317_000333-T1 CF317_000333

MGSEGQDTSASEPDGLRPRGRILRKIRSEDGTDVEQTIEKKPWKPTSQEGLRDLQSLVQSLKEMSAQFKQVDQEGAARGTDPSSQQPDDYLELPTSPLEKHMAEKKKWHPKRRPTPEEKERLAHNPWAAMLAGPLRLDAASKARSPVALFMDLGHITNPKDGVTYLMPDDLADLEAFDRRLKNGHRSAALKRQAPDDAGEKIHILPYKLLLEELTDAFLVWDNVNQVGRTKKGVVAKRLFPAKWQEKAQKLEAYRSASKDYWDIRAKQGETDDGLHKRPKQAYDSNTLQWQPEIVERVPDIMRQRVLLAFNHIAQMQNQPKDKQRKLLYGSEWLEGRSMPLTDKVLRSMGIPVEYWNDMGDIESAQAITGTGERQGDSHDPRPPIAVQPSEIPEESVETPYDQLNMQRESSTVNKSGLKEKSSQLDKDDPNVWLPGSFVLHIGPPSVCLANLPYVDFSSLPLSYLPGTVDPALEHSKYIPPMITVAEAHRLPVFNIPAMLGPRFDSILRKILRRHQQHIDIPDRLKRDQ

>CF317_000334-T1 CF317_000334

MVNRLANKVAAITGAAGGIGLETTILFVREGASVLMLDISEPALEKASAKVHELCSELKDSKQFLKTMKCDVSKEADLKAAVESLDDAGGVDIMFNNAGIMHSEDDDAISTSEKIWDLTQAINVKGVWFGCKHAVLSMRRHKKARGSIVNTASMVALVGAAAPQLAYTASKGAVLALTRELAMVHARENIRFNSLCPGPLNTPLLQDWLGDDQHKRYRREVHFPMGRFGEAIEQANAVVFLASDESSFVNAADFVVDGGMTKAYVTAEGPPTAAPKNNVV

>CF317_000335-T1 CF317_000335

MQNVLPALDKSEETKDIGPQSLTLDNIETALAGDTKVKLAGIDVDGILRGKIISKKKFFSVAKDGFGFCSVIFGWDMHDMTYFRELKISNAENGYRDLIARIDLKSYRRIPWEDNVPFFLVQFHDPDTNEPVFADPRSILRSAVDKLHNAGYDAMAGAEYEFYTFRAPRDPTLQGEGRTSATTMPYLQQNNTNSLPHLTQGMFGYSLTDPVHNQDWFYKVFEACEQFRCNIEGWHTESGPGVFEAALEFGQVNEMADRASLYKYVVKAISSKHGITPCFMAKPKEGLPGNSGHMHCSIVDQSGKNMFARAEKDPNPPFKDLEYLSDMGRQFLAGLLDGFSDVMPIIAPTVNSYKRLVENFWAPVTVSWGLEHRAASIRLIAPPTASPKATRFEIRTCGADANPSLVLATIVALGWRGIEKKLELKLPPLGKGQEVGSKEDKGERLPKSLREATTKFMAKDSVAREIFGDDFVEHFGGTREHELRLWDEAVTDWEIKRYIETV

>CF317_000336-T1 CF317_000336

MALKVFVTGATGYIGGTALASIVEAHPEWEITASVRNSDKGAQVAALFPRIRLVYGDLDAVAMIEEEASKADIVYHWANADHVPSAEAIVRGLAKRQASTPAFFIHTSGTLILGWASIREKHFGESRGKVYDDWNDIDQLINLPDDAAHRHVDKIVLAASQQHPDKIKTAIVCPPIIYGKGKGPGNQRSVQVYKSAEAFLRSGQAFMVGKGENHWHHVHVADLARLYLLIGEAAAAGGPPATWDDEGYYLAENGFIAWGDIMKALGKEAHKQGFLPSAEVKQFSVEEANNVPPFPTISTGTDSRGVAMRGKELLGWKPREQSLLDEIPMIVTDEAKALGLAPKGHAEMVTKE

>CF317_000337-T1 CF317_000337

MGPEDLQRNTYGQDVRHLECPFYFGPRGCKWTTAQCKYAHHQTGERAEKPRYIRGVGCVAGENRRRARRSQMRGSWRAGTQTPSPEPEVREHTSEDGPSSSETDRDSSPEKEEAPPVITRILCAKCLVGAPERGFCLAPGCGRPRRG

>CF317_000338-T1 CF317_000338

MATQANWELDPETRTKLAALQKKEGSGNSRCADCGAPSPQWASPKFGIFICLQCAGTHRGLGVHISFVRSISMDAFKATEIKRMQLSGNKTWQDFFNANSTTSFDDLSIKERYDSEAGEEYKERLNAKCDDREFDPVAFKKERAAILEKQAAKNASRSQTPMGGSRTQSPAPPKFGNDPTQKAKNEEYFARMGNANATRPDGLPPSQGGKYGGFGSSVPEPQQQQAGLADEFQKDPVAALTKGFGSFWGAVSKQAKVVNESYIAPTAKNLAQTDFAAQAQKGLATVSSGVASGAKGATEQFQKFVEGQDNAAAASAARAGGRAEPERKDFWDSFGASDESANTAPSKPSSIGTAAMKKTTTSPNETGAKKKKDEGWGDDW

>CF317_000339-T1 CF317_000339

MSTRQAIYASAAPKPNGNYSHVIRSGDKLYLCGWMGDDPKTGEIVDGDIGAQTKQAIANIKACLEAAGSSLDKVISRRLFMTDRTEFRTVDALWAEAVGEPYPVSTLIGCSWLAKDGARVEIEVVAEA

>CF317_000340-T1 CF317_000340

MLAKHARLGRHHVALRSSKLTCFQHPLSKTFSSSRTLRDGHSFSDTDRTRNIGIIAHIDAGKTTTTERMLYYSGHTRRIGDVDDGSTVTDFLPAERARGITIQSAAITFNWPPLADPAEDSKILQAVQKQELPRSALSHRINLIDTPGHADFTFEVRRSLRILDGAVCVLDGVAGVEAQTEQVWNQAADWRIPRIAYVNKLDREGAAFGQTVREIGVRLNGWPAVCQIPWFEGGRGKFCGIVDVVNLCGMKYEAGGDGKVVERFNLPQLEIQEPALADELRKARAALVELLSEHDEVMVDAFLEAEEDHLAITPAQLLASLRRCLMSGDAKVIPVFAGASFRNIGVQPVLDAINDLLPSPQERPHAEVSINESTGRLDELLGGKMVAQTSPTTKSGKRVEAPVPAMDKMLKGCALAFKVVNDARKGMLVYIRVYSGTVDRNALLYNTNLKQSERATNLLRMYANESVPVQSLEAGQIGVIAGSKFARTGDTLIACNTNKATPPAPLDKLQLRPIHVPPPVFFASIEPNSLKDEKDMHDKLNLLLREDPSLQVTQDEDTGQTLISGMGELHLEIARDRLVGDLKANATMGKIAIGYRETLTQTSSPSTRIFDSSARGDVKGKAGCTATVEPNSPGVPDEDYHDNASRFEQDQNVIIVQAPSLDKRGRPLHDDAMSLPGHLTLSEVQSAFVNGALAALARGPAFSYPLRNIKVVLTIDPAKHIFGNETSYASLTAAARLATISAFREIHSAGGSALMEPVMNVDIAVDDSSMGSIIQDISSSRGGQIVSLGDNEEAEAEEQSSARDIDVSRIYAPKDPFEFGTSASQGQQINKPRTVKAKVPLKEMVGYLKHLRSMTGGRGTFTMSVDRFEKMTGQREKALIMELQGGA

>CF317_000341-T1 CF317_000341

MDYSAAIADDPAAASPWGSSSPRAQRAPFTGTIPDSPRTPGGHVRSESQSSLPESAMHNSQTPTDDGSFSEQQVPHPASAQSYPAEAQQPPPPPPQAQGQGQSQAQHPHAQHAQQQQQQQQRPGAARYHSARQQRHIPQYKLQAKVTALERTGRKDPVLRFDVYTNLPKFRTTQFRDVRRTHSEFIKLQEHLISSNPEAMVPAVPPALTSAGAGTEEDEVRVKASIQRWLNTICTNEVLMRDEEMVFFVEADFGYSPVVRMRQPATGVRRKVIKQFAPPPDDTPELHFARPVVKAFYLATLDSAQRLDRVVKARRGLGLAESSLGEKLSTMHVQETHPGLSHAYRKLGRIIQQCGDYHAAQGTGEATTLGDALSYHSADAFIVKETLTNRHILLRELVQAEQTRKSKESAANRLKQSSSIKRDKVEDAITQLDEAAQAEAYLRGKCQRVTQNLLVEKRRWVDRTSNDTLNAIRDYVLRQIETERRTLAILETVRPDIRNIDASGGLSRLGREHHPNVRRVSMASSQTAKGDAWSGVQRSREALSRSVSGSFVAGIPETEETSTNGSATDPATGRPRSGTGSASLASVKEDEDNDRVDARNAASRLAQSTF

>CF317_000342-T1 CF317_000342

MRRSVLVFLLITIVLLTFLLHEVSTLIALLLEDASTDAIQKSELPAPNSTLLNNRPQLIPKIIHQTYINESIPAHWLPAQQACLDLHKDYEYKLWTDAKSLEFIETEYPWFAATFRNYQHPIQRADSIRYFVLAHYGGVYIDLDDGCKRRLDPLLSYNAFVRRTVPTGISNDVMGAIPQHPFFLRVVESLQGANRKWFLPYITIMASTGPLFLSVIWKKWMGEHADLQTQSQNLVDGASIAADWKGRVRVLMPDEYSGHTWSFFEEYKGNSWHGSDARLIFWMGKNWMLLTAAGTVIVLSAGLAMWWVYGRILLLGQKRRGYVAVGPTGSPKLTASSGSSRSPRFSPRKGPSLWRKLGMEGKKERYELVESHDA

>CF317_000343-T1 CF317_000343

MADKGTLRRRSLRIFHPKSQASDEYSAPSFTPSPVEYSPTESSIADGASPRIRPKLLSRANRNSVFGSLRSSHSLEEDDKTLTKSDSKSSSLQGDADSLGRGLFGDQVKRAVEVQVTGTSMFRKRTQYVVLTESHLIRFKSQAKASEMFPTIPHAAKNSLPRNAMNSIGSYTDMQTGPYSDITQGVPLDDVIGVYKVEDGRPYFTVELSYMDEQGKRASSLQLNFNGPREAEAWVVAIRENAVIRRAQCTRKYAQRTLDYLARALEKEEDYDPLHFRVFKVVQRSPVRPAGTRGPNDELSKSNSSVSYLVIGINKVHLIALPKASARSSSTSLSELDTPPSSFGIVTLTSIKLRSGDDGFDLYFRTPLRQPYLASLASYESRQIALWLRYASEYLRPEWTVQPFVFDVPQGLEDEMAPPTFPPEDNDCFDRTLIAFCAAFNVDTSRIYYSVDYECEDAPCFRLLPPEVGPSYDSMELLAVFRALRYNESFTSISFANINLCTLRNVYDPFGESEDGLCTRSGVFVEVPEHANLSALQQEVRALALKSRRLRRFDFSHSLPAIKAGQSSGIPEALTTLCKKSATNVDWFTLNGIRLSEHDIDFLVDAASERQCHLRALEIGECGLSVHDVDVLLSTIGIHDNTMEVIDISGTQGRFSPELFQRAIGALSRLRRLNLTRVQKTAGPEPLIAPEILLSWRLESLQLNGTTLNEQSVDTISAYLASPKSDLLRELSINQCGLTGKDLGVFFQSMTREPGVARNMHVSASENRLGVGSSLLCKCIAEDYGPASITMRMVDFDKEYQFRELVAALAKNTTIRSLDISQASLPYDASMETCEALKDMFAKNHTLEELDISGDVAHLDVARFGIGLNVALLGLEKNNALKLLRIEHQNLGLQGANTLAGVIEKNSCLAEIHCEHNDINLQSFTVLVGSLKKNKTLLFMPAQDADRAKSMEKVREEFEALNQCDEPKSPRTGTLMKSFHAVAQKAPSVKRHRRQSSTLSAQSNSSFTQSDYNETLVALEEQWNTQVARLQQYLLRNYQLVSGLSWEELDSKSEKDSRTRPSTADSLARMLAKVNFDSNPTEFGLEDDKNISPTHYDMFEDNGKTPTMEKRGFVFSLPDD

>CF317_000344-T1 CF317_000344

MRPSTSSQTYVCLPCLLRTAPTARTGRTNVSTSYKQDLLTQSRRITTAPVGVSPVSQPRRKHGAEQPSATTIQSRTNTSSTAEPVKLSINQISKLGQLHGLRTRLQNGLSAVSDAESDPKDVPIEKILAALKTEGDAAASPLLQVIGQVYTGRPYNEVSEELSMISKHRRPAHATVTDDATISVLNKLGILTQGKDSGAAKPSQVTDVAKEDAEEVSATSRLNRERRLRRVRKVEEKKKAPRVAKVASTLVRKELVKPGPVIDAPRTRKTSSDAAPARARRASSDEAVPIPLRAPSDAKSVEPARIPQRAVETENTPSVPRLSFDLSRVLFNPGVYHLQDPRSRVYNFDPYLEKIMPVTEFNFDALNPYITSSQDQVLQDISQKHRKRYVGSTSSMSAAMSHFHFLLSAWRPIDPSILSQKITGLKSFTQITRTPSSIYLRWKNGTYAIDADKEYDTPNILMMQGKSMEKLLTLEKDDFEKYRKPKAGEQAPAIDADPEAFHYSTVENFLLRSQLDAYDPRLPGTGMFDLKTRAVAGIRMIMKEHEVGMGYQIKDRFGMWESFDREYYDMIRSAFLKYSLQVRMGRMDGIFVAYHNIARIFGFQYVSLEEMDMALHGQPDRVLGDREFRATLKLMNEVFDEATKEYPEQSLRIMFETREPSKTEPNGYMRLFAEPMTDEEIDMVQKSGKDKVEEFEKKISAGIDPKASEPAGKTIKEKLSSSTLESNAADTAFLDDVLSETTVEEDLKTPAETQERPVISWLLHIYNEVNGTSVARPNGISTEDRWVIRYKFDRVADSKSRPLYNASRNRRAADLQFNLEAKAKNFFIDKLRVMSESGRVWREEQDALDAQREKVMLYNLEQ

>CF317_000345-T1 CF317_000345

MEGHLRILVVGSGGREHAFAWRLASSPSVDVVHVTPGNGGTDGDKIVNAAIGVSKDNFPELVSYARKVGINLVVVGPEAPLVDGIEGFFRSVGIPVFGPSKKAARMEGSKTFSKDFMQRHEIPTAAYRNFTDHAEARKYVEHVKHDVVIKASGLAAGKGVIIPQTKQEAYDALESIMVKKEFKEAGDEVVVEEFLQGDELSVLTFCDGHVIRSLPPAQDHKRALDGDLGPNTGGMGAYAPTHLATPEMIVEIDRDIVQPTIDAMRIEGMPFRGILFTGLMITKNGPKVLEYNVRGGDPETQTLLPLLSDDTDLAKVMLACAEGWLDGVSLKIKSGASATVVACAEGYPGSYAKNRQIHLSKPSADTYIFHAGTIRDGKGLWSVGGRVIAATATGANLEEAVAKAYKGIDTIKFEGMQFRKDIAHRAFGAKNRQRRESDAPLTYAAAGVSIDAGNSLVQRIKQHVKRTVRSGADGEIGGFGGGFDMSSLKAGFPTIITCCDGIGTKLKVAFATNVHDTVGIDLVAMSVNDLIVQGAEPLVFVDTYTTSILDVDTAADFIKGVADGCVQSGCTLVGGETAEMPGLLAGTEYDAVGTAVGWVDTGAGKKVLPDLESMQKGDILIGLGSNGLHSNGFSLVRKIIERKHLGWDEIAPWDRDSNVGTSILTPTRIYVKPLLEVVKRDYVQGMSHITGGGLSENVPRMLPKHLSAEIDVKTWKRPAVFKWLQDAGKVSVEEMSRTFNNGIGMVLVVSEIAHGEVMRILEAQGEQVYKIGKLVARGEDQGCVLSNLKAWSA

>CF317_000346-T1 CF317_000346

MHIIKVPCSSANIGPGFDVIGLALSMYLELTVTTKPNEQSTSSNPLNCTVTYSGVGADGIDLNPSQNLLTRTALYVLRCHNQRAFPPGTHVHINNPIPLGRGLGSSGAAVVAGVTLANVCGNLQLPKSRLLDFALMVERHPDNVAAALYGGFVGTYLNELNPEDMARVEVPLAEVLPQPAGGEDTGLTPPIPPMDIGHFRRFDWSKELKAVAIIPHFEVSTAKAREVLPQQYTRKDMIFNLQRLALLTTVLSDSPPNPDLIYSAMQDKIHQPYRSTLIPGLTEILQSVTPSSHKGLCGICLSGAGPTILALATEHFDKIAETIVDRFKQEGITCEWKVLEPAEDGTTVVEVPDS

>CF317_000347-T1 CF317_000347

MEEEVAALVIDNGSGMCKAGFAGDDAPRAVFPSIVGRPRHHGIMIGMGQKDSYVGDEAQSKRGILTLRYPIEHGVVTNWDDMEKIWHHTFYNELRVAPEEHPVLLTEAPINPKSNREKMTQIVFETFNAPAFYVSIQAVLSLYASGRTTGIVLDSGDGVTHVVPIYEGFALPHAISRVDMAGRDLTDYLMKILAERGYTFSTTAEREIVRDIKEKLCYVALDFEQEIQTAAQSSSLEKSYELPDGQVITIGNERFRAPEALFQPSVLGLESGGIHVTTFNSIMKCDVDVRKDLYGNIVMSGGTTMYPGISDRMQKEITALAPSSMKVKIIAPPERKYSVWIGGSILASLSTFQQMWISKQEYDESGPSIVHRKCF

>CF317_000348-T1 CF317_000348

MDKGMNNLLAWSIENSQNAQSQNPDGTAAPTQPGYKGQEPRSIDADALRSLMGMGGPSDADLMKQSMHVIVNPEATLDAKMVAFDNFEQLIENLDNANNLEPLGLWTPLLNQLDNTETSLRKMAAWCIGTAVQNNEKCQAHLLSIGGAAKVAKMAVEDDDSAARRKAVYALSSTVRNFQPGMDEALKILPKNVTGPDHVNASDMDVIDAIMEKLREG

>CF317_000349-T1 CF317_000349

MESNRLFHKFPTPAWVNNANVRTGGVYLSGAMFSLGFFLFLDAAVFSHSALNGSTIHITFVDWIPLICSTLGMTVINSIEKSRLNSDSFSYSGNGVAWKARLVLFLGFALIAGGLAGSVCVLVLNYIVEEARPPTLWMGVSNVLGNALVMLSCIVLWVSQNIEDDYAYNLSL

>CF317_000350-T1 CF317_000350

MVAGLKTIIALSFVLAIGFLLVILSAALFHNYLTLLVVATYVIAPLPNFICAKCANPDDFMGDAGYAVVDFGRFVTGFLVVMGIALPVVLAHCALIQVPAMAMSIIGGLLIYGTIISFSQFFKEEQDF

>CF317_000351-T1 CF317_000351

MFRNNYDNDSVTFSPQGRIFQVEYAQEAVKQGSVVVGLVSKTHVVLTAVKRNAEELSSYQKKIVGVDDHLGVALAGLASDARVLSNFMKQQSLASKLSYGRAIPVERIVNMIGDRAQSSTQHYGKRPYGVGLLVAGVDDLGPHLFEFSPSGLTQEMMACAIGARSQMARTYLERHLDKFADCGRDELIKHGLLALKESLAQDKELTVDNTSLAVIGVGSKDGKRRVENFKMYDGQEVGPLLEANVETSGTGEGEGDAMETDS

>CF317_000352-T1 CF317_000352

MLSGSKMASTNGSDTPQPVVCVFCASSPGKNPAHLESARQLSTSLSNAGYKLVYGGGTMGIMGEVAKTFVSHAGPDSVDGIIPRALVHTEKDPSTRNDSPAPPQDSSVKQPERTVTRDQLKRANTSEIIPQSEFGNTVVVADMHTRKRMMAERVVGGGPGSGFVALAGGYGTIEEVMEMVTWNQLGIHKMPIVVVNIDGIWDGLLDWVKRSIREGFVGEGNANIIMEVKDVNQVEEALRNYKVAEGRFKLDWTKE

>CF317_000353-T1 CF317_000353

MLKSNVSFLTVDIQLALSELEVLGTGVVVVSGSEDAAGVTDVLDSVDSIEEIDDAGVVVVLAEEITDDIDDEMDETTDEALVATEDSEEATDDAEVAIDDNDDAADELSTAEADDAAADAELAIELADDADAAADETASEAEADIEETLEVAVLS

>CF317_000354-T1 CF317_000354

MRRRRRHNAAIADDDRYTPSLGNSMSNFGYAGAAVGKHSSRYTDSEINSNSPPMREANPLDRYATPGVSSTRTPAPTNSQSRTSDISSVSGSEPATYRPGPGMPAVPEQHARSNFIEELPDQTNEYLNPRSQWPAGVGAAAASAGPTHYHSLSGDTASVYSDDGGYPGPTQQSSPMHPTNRQPNINPAALELSGENANNGPGNGGGAGQGYRGLDPDYQSRAQLTKPEGRRFNPSQNF

>CF317_000355-T1 CF317_000355

MPSSRPTTRNSERSYVSEFASSDNVILNPLYTNPDQSALPSQTNLRGPRGRRQANATDHGHPGGNGRREQVFALGSREDVQQEDYVSPIFGMFNRAWTRYRDAEEERRIVAARSLVASQYADDWDNDVYQRTDHDDIDIQAIDFQAVQQENLPPTDEQRRVVARQRLLDSSPLTVDTMLREGHAQMDRNVAQAIVINGVQQAIYLRRANSSSNVASSQQDPPLREVANVDPVILEQTSSDQPIPRAARTARHNFHRQFYSNPFDEQRPLGSHNDGQSVESIDENIFAGVPSVARMNPIDTQQLRRPAPTKSEDLKRCVVGAVSSGGPIAEPPMVEWTTVSGGASYVVSR

>CF317_000356-T1 CF317_000356

MQFPKLSQLQSRFAVSFGGTLLLVLVYLSISRPSSAYAAEIDSTILLDYDRLLWPVVLEDGSFSQEDNIPKDNTQAAILGQDDTLDEQRDAAQPSAFEEYEEGQRGDYGLDSIELRKRAPAGVSSLANNAPQNMNIEIGETENWMFPLDEIEGPHGEEGVGLPSGASTTGNTDDSAPSRRELKKRQSSTTIWITINVCLQPASNTSEDKTPPQLQLFYSDSTDIQKPGSTSEGTKGIQVHVEGGYGIIKLNATDDIYVGVSAPNTTDFTGSWNYEIAASNDAPYHFYSNSTDLLFVDGDNHAALLVTQDLPGSGNSTIYEEWMTMQPPYGMFAHDQKDKSFLGVSRSYCGLRNNANIAANVPGITNNNVGTMTNRGLGGAPKEQFYISALNASSDYWGFLAMTGNSTATGTGVVGGGGRVWSNMTFSTKASDNCALMYNLSFCSEVAYAVPSNPDKYSPLDGLADLAAVYDSYAAQMYQYFNYSLQQIPCNTTASAQYSLARNCDDCARAYKQWLCAVSIPRCEDWSNDALYLARRNMAQRFPNGTVPSWISNPDPAQRVLLNSVQSNSSRNSEIIDDVIQPGPYKEVLPCIDLCYDLVQSCPAALGFGCPRGKYQNMSYGVRDPNPGILSCSYLGAAYYLSDAVKDTTLPGTMMWASMIITVALLFF

>CF317_000357-T1 CF317_000357

MACVGSTQAIIKRRENDLALMPPPPVKRIKRPTEVLDEDDYTKALSDIVARDYYPGLREAKAQEEYLNALDSKNIEWIREAERDLRQARDGKVEVKKVKRKTRDTRYDTPRSYKQAEDTPQGQYGADTPVTTVDTEVADGGAEGLLTQDVDTSGLSLSAYQAKYTSEDNASFNTLLDKQNDKRRQKHAHLWTADQRMPSQRLIAHRAQQQALLKDKADYESTNGKELVPVTTGSTDIRSAKPASWKITNPANTFMFTPSTSIDEQGLLTIQDQKEALSKAGPKQIIHENTRFPPSGPRWEADDEDDATSIHTSFIARRNARGDAATDLGTITGAETPRVNGYSFVDEDEEPPVPALKQEPTYRDLLAGQAGDGLPNPFRLKDTRSREDLHHRLVERDAEKKRAKQKETMKGGKEVGNMTPATRRMMERLGGRTPTAGSSGSKEQRREDWTPVATPRRHRAAV

>CF317_000358-T1 CF317_000358

MDLLPEWASYRIDRNGTRLPKTTPPPQGTYKASDGIGKTDYSDPRDFYAPTPVEQQLVWQALKPTRESYHVLTTTTLRIEEMRCLPLGYNASWDEIRRKFVNWCFLEHGPDSLDMSDHVAEGRIVRVWGPSVIPGYSQISTDARREPTLNNRILGGKHSTYLFRNGQLAIDSHRHLPKLRRWYGLIEDFYFSPSWKPRVKNSTGVYLQNPDAFACRRCLLSRVLCYGHREHPNRTCGPCQVLKLKCSKEDDYGDEEVQVWNDRVPDATRNPTTLPQSDMMMVSDPRRQYASPSGCGKYPPGGLFGVFVKPKSSTMRFR

>CF317_000359-T1 CF317_000359

MANKMSKYSPADAKVLSVETMPPNESKWIQLKKIKWQDPSGKERIWETAERMTRPKNDTLDAVGVVAILNDPSSSTGPSLLLQKQFRPPVDKITIEVPSGLVDEGEDPGTSALRELKEETGYVATLPKDAKAAEGFVMFNDPGFCNTNTKMIFVEVDMNDPRNQNPEPELEENEYIETFTIPLDDMWNELEKLEQQGFAIDARVGTLAQGMEIAKKWKGVLGKQA

>CF317_000360-T1 CF317_000360

MGVDWGKSKALGGYFTAMIDNGDKEWHWSLIPFILIWLIFVGLLTLWLFTVFVVPYPRKPFPSEKKYRTLDKDGQLTAPKDLPCWMDAAKRKWLEAKEKNQEFNVNKYVERAEVFMTLVVPAYNEEKRLIGMLEESVNLLERHYSNVPVPASLKNQQANGSTARKRKSQQPDIYEPIPDTIYGDFTPKGWEILIVDDGSSDKTVEIAEHFTRTHMLPAHPRPASGPWTPNPKTAVNIPPDSIRLISLESNRGKGGAVTHGMRHARGAYILFADADGASNITDLPKLVTAAEGVADSESRAVAVGSRAHMVNSAAVVQRSKLRNFLMHGFHLFLWFMTPPATAQIKDTQCGFKLFSRATLPYIVPHMHMEGWIFDVEMLMLAEFNRIPVVEVPIGWKEVAGSKLDVVKDSIGMAWNLFLLRFCYGLGLYS

>CF317_000361-T1 CF317_000361

MRQNGNIGLQDPAASPTFALSRPVNTDNPDVPPSLRRNLFGSHLSRRPAGPLPVASTDRMPDPFHPVTSTSSHPHPEIYQSSIQHSQRAANVNHQNAYDPPPRVSPTRSQSPAKSLAYANSNTSIVALDPTTGRPQIPVMPRLPARMREGSDEEDSNEDSDDLDDSQDPSQDHSTAWRRMRASEYANPSQHDLDLHSSSMLGMHPQSQSQPGRLHELIDPGPNGTDYTDYTKIEDILSEMQRQQKARAKTAHIPESSSSFSLDTPTDGGGVSARTRGKSRANPRGQASPTKQPQDETKPRIGGVGATIKPADKDQLLGLIMTS

>CF317_000362-T1 CF317_000362

MPLRPTHLGAAAIFACLLLWYTLSSGSRSEPVPVSAFVQPSGVKPDPSDELPFYKIPANSHFRGHNGIINFTNPQIQMAKVHISVSQSDIQSLPLMQPLDIKLPASTQITVTDELSDQNVKQPEVARTTVDIPRLPQTRPNASELIFGVATTYERLEASLDTIGHWLSGTSSRLIGNMQPTLDPDAEVRLLTKSRGLKLGLGVVSSRYGFLDRYFALLKLLHENKGPNTKWYVFIDDDTFFLDMSTVLATLAKYDPAEPWYIGALTEDFQQMATWGYMAYGGGGIFLSAALVEQLAPHWEQCFAKRDTGDKMLARCIYQYTTTKFTWEPNLHQVDLHGDQSGFYEALRPQPVSLHHWKSDAFRSSIDMYNLSKVASVCGNSCLLQKFKFKNNWVLTNGFSLVKYSGKDLKYRELETDVSMELTWDFRYGGASSAHFEHSLGPIRRPDAGGKISFRLESAIAESDGRVRQLYVKRHKTDPEVEPYGAVEAVVEIEWSPV

>CF317_000363-T1 CF317_000363

MAKIKASAIEDLKSQIDDVTSKPEENAAGIVYVAVNKQGEVIFEHASGKAGLGLDEDMTMDHTFWIASCTKMITGIACMQLVEQGKLSLDDSDLVEKLAPELKEVQVFDGKQLHKKDRPITLRMLLSHTAGFAYSFFDQRIQDYYGAAGINEFSGLPYDYLSQPLVNQPGTVWEYGINIDWAGILVERVSGLKLNDYFLEHILKPMNITHINMFPTDDMKSKLAWMHSRDLHTGKLKRSIDGHLNRAALVAKTQAEKDAVFQQGGAGCFARPNQYAQVISMLLNDGVHAPTGNRILKKETVDAMFTNQIPDFPNFGRTAITAPKPEYTNSLPELYPEPHDIPQGWGLTFFLHLKPSAIHSEGTGWWAGLPNLFWWADRQKGVGGMIASQILPFGDPKILGLWAQVEGGIYQNLE

>CF317_000364-T1 CF317_000364

MQLKALVLTLLQSFALVSAQDAQQPLGGAPSNDSLLWGPYRPNLYFGVRPRVPKSLLTGLMWTNADDFKYAQENFRHTCEQHAGMAGYGWEEYDTRKGGRQVIHDAGNKIDLTVDFVKIPGGSNGGSWAARINGALRADAPPNHVTTLLFYAGMEGIGELGFAHEPQEQGIAGPVTIHGNTGELGDFDIEIVDGPNNKMPYRLHPSWDEKHLDVTLTQSFMAPVDQVWRGREIAFSAFKQTVDKLMKVYTKDNMAPPWQAFTINNNIMPGNVHMIQKVFVGNFQFDILFKSASGPDVTADALDKAISATSDSFKSRFPKVFKPAKPFDDKDHQACAESMLSNLAGGIGYFYGDSLVDRSYAPEYDEEDEGFWEAAAEARARNQPTTEPASGLFTSVPSRPFFPRGFLWDEGFHLLPIVEWDADLALTIVESWFDLMDSDGWIAREQILGPEARSKVPAEFQVQYPHYANPTTLFLAVEALVTKLESNDLSSSEKANIRSRLINLYPLLQKHYQWYRRTQAGDIKSYDREAFSTKEGYRWRGRTPRHILPSGLDDYPRAQPPHPGELHLDLISWIGLMSRSLITISNLLDAKDDVETYTKHLTAITRNLDDLHWSDKHNVYCDATIDDYEEHQLVCHKGYLSLFPFMLDLLPHNHKSIPATLDLIADPEQLWSSYGIRSLSQQDEFYGTDENYWRSPIWMNINYLIVKSLHTLATDTTSATAAPSKTREQARKIYSDLRKNLVNNVVKQWKDNGFAWEQYNPNTGLGQRTQHFTGWTSLVVVIMSMEDLPPAGKLHEEL

>CF317_000365-T1 CF317_000365

MATKTAGGNAAFHNFHNDFSHIQDPNERRRLALAEIDKAPFGWYHVRAIAVAGIGFFTDSNKGKIPPNADTAIKVATSGGTVIGQVGFGWLADVVGRKKMYGLELILIIFATLAQALSGDSSAISIVAALIFWRVLMGVGIGGDYPLSSIITSEFATTKWRGAMMGAVFAMQGIGQFTAAIIALIVASGFKESLQTAESAATCSGVCGLAVDKMWRIIIGFGAVPGCIALYFRLTIPETPRYTFDVARDAEKAVEDVKAYQAGKAEGKPDEVSRAAVLQDSRVRLDPPKASWADFRRHYGQWKYGKILLGTAGSWFFLDVAFYGLGLNNTIILQAIGYTGGSTMYEVFYNAAVGNLIIVCAGSIPGYWVTVATVDTIGRKPIQLMGFTILTVLFIVIGFAYHHLSHSALLGLYVLCQFFFNFGPNSTTFIVPGECFPTRYRSTSHGLSAASGKIGAIVAQTVFGPLRVKGHPTTANPSPWLNHVMQIFALFMFCGIFTTLCIPETKRKTLEELAGEVPGTANYDPESAGHDTASYVLPDNSSGEREVAKETV

>CF317_000366-T1 CF317_000366

MIILLSDPPHTSKILKAEAFAEEVRGLLYTVSASELHYSADCVKPALEKTFDLAARWKALILLDEDGAFLEKRSDNELYGNNSRPLPQTRRNI

>CF317_000367-T1 CF317_000367

MSLLITTASSSPNESLEEPLSTITTTSTTTETVTSWTRNHSVPVPKMLTTITRLLPDPYGATFSEEWMTATGARLAELNAQRMQSMSANSTVTPMVSMASMTITTGSDEVMTPSTTTALGLDTTSEDTATAGAQSTTAFSSPAANIPPSSTKYTSAGVVSRLSDEATVGAAVSTDTLYEDAPTTVYITVDASSSTPFSNTTPQASASEIATKSAVVSTDFVTAPASLDTVLTTTKPPTSTIFALNTTTTTTMPDQASAVTLDTSANDPITSAGSPTASASTALASTPYSSSSSSALDPELTQGADPPSFPAPSTATPLASLSVQPPPTSTEHPTPPTIATATPTRLALHPISLNPGPITWGPLSSAPATALKHEDPHRNLARWATIETPVARTPSHRLSPTSYHTNAKARCTATSLQSRHRHLASPLPLWLAASVFAALLVLVCWRVLVGLLARRRARRGRSVRSCGGDGSWWWWRTPRSGHGLKRRWSSREYGVGVESGTGYGNENENENERVRASTLECGYVPGQHAGERDPMYWPHERVGREVERRGSMP

>CF317_000368-T1 CF317_000368

MEALLRQSKGVCPFLKKATPGALRTLSTTSYQSPAGGTISKLQHYARRCPVMNKGLAVQSAKINARSYSKAATSTSIVKSLLEKKLHTTAEKKASVDTQMYRAEQRPVPPAKAKQFVKDPQVARAGPKPAVPEPPKFDYEKFYTDELDKKHQDKSYRYFNNINRMAKQFPRAHMATPEEQVTVWCSNDYLGMGRNQHVLKTMHETLDMYGAGAGGTRNISGHNQHAVALEKSLADLHGKEAALVFSSCYVANDATLATLGSKLPNCVILSDSLNHASMIQGIRHSGAKKMVYKHNDLEDLENKLKSLDPAQPKIIAFESVYSMCGSVAPIEAVCDLADKYGAITFLDEVHAVGMYGPRGAGVAEHLDYDVWADANRNADTQKGTVMDRIDIITGTLGKAYGCVGGYIAGSAMFVDAIRSLAPGFIFTTSLPPATMAGARTAIEYQARYQGDRRMQQVHTRELKDDLTSRGIPVIPNPSHIVPLLVGDAELAKAASDMLLSDYQIYVQSINYPTVPRGEERLRITPTPGHHGELRADLVQALEGVWNRLNLKRISDWKAQGGFVGVGVENADLVEPLWTDAQLEHAAELPKAAVDDVQDARIMPVAAAAA

>CF317_000369-T1 CF317_000369

MAEAPSPAEVKHLRPSELGTKEYWDQSYQNDLENGFGQEEGAELTQLESWFDEVDAPQKVLEYLTDEDFPLSPNYPERTNAAPTVLDLGCGNGSSLFELKLEGEYHGSMVGVDYSQQSVNLAKTLWSKHLEQQDEAERPEGSISFEQFDLLKDTPGEQSWWPEGGFDLILDKGTFDAISLSAETTEHGGQEVRIVEAYPGKVAQMIKPGGYFLITSCNWTEQEVVRWFTETAEVQNVLKEYGRVKYPVYEFGGRKGQGVASVCFQRALVS

>CF317_000370-T1 CF317_000370

MLLSRVWVRPPPNPEQEDIFSSSLASLFTDDTQNSHGTPGQSVVYNSPRHGDINLEIPRHPDVEEGRKLFAHYLWNAAVVAADAIESASTDEQEELDASKVQWNKKYWDVRSKNVLELGAGTALPSLISSLSAAHSATITDHPSSPALVADTIAHNVHHNLKINLLPTGSASESEPKTNTTISIHGLTWGDPLFTSPTTYGKPSSPQPAKHSFDKIIVADCLWMPSQHANLVKTIDSCLAAQAQQDEAQANAASMPCALVIAGFHTGRSTLARFFKIATGSSTLSLTASVPDRTQTADLDSPSDEDEDEDEDEDEELKALSTHAPLRAAELFEIDVDRNVRPWQPSRAGEDKHAAKRWCVVGVLVRR

>CF317_000371-T1 CF317_000371

MASTNGAHTVQGDLVRGVLAEVNTLTVDRLKRVLKAENLTVSGLKNELQIRLRAHVHDCERNNDQNGLKRIKDAIRGYPASQASAAYNPNPYPSPYSNHTPASSASPQNYYTSPTPRLPPPPGVMPHLAYNSSRVTFKNSPFYTIQKQLSNVVELKPREQTRDTARLTINLDDALATKFQSDSSCRAMVFCAADAFDSAWKPVDIAFPHHAELRCNQDEVKVNLKGLKNKPGSTRPVDITPFLRKKAGFPNNVELVYALTNKISPMPSACLQGANQQKYLLLVNMVQKKSIDSLVEELKRGKYLSKARVVQEMMSRANDDDIVVESSNMSLRDPVQMTRIDTPCRSIGCKHNECFDAAVFLALQEQAPTWTCPICNKPAQWENLVYDQFVQEILQNTKRDIEQVTVEPDGRWHLIKTDEDNPQPESKNPFSHHQHDSDDDNDDDDDDLVEITDIDAPKTTPSIRTRPSADAIRTPSLNGRESFPPPPAPSSISRAQSVQHPPAPNSQSRKRPREEVIDLTLSDDDDEPPVSRVKRPSLSSQISDSNRSGVPGPYRFNLPPPIPSRTSTSSPANRYSPGNFYDYDVFKTRL

>CF317_000372-T1 CF317_000372

MAKSARASTRKRNNATLRTKVFGPAYDARTERLSNKLQELANAPKPDQKKKMHVDDDVDEDKKEADAHVPAVDEDQVEAMQWTGLGPSLKKQKRAKSAGRLNKISKRKPRNRMVFQSEIARKKRQSKGKKA

>CF317_000373-T1 CF317_000373

MDFRGFQEYKPGASRYNGRISEDEWNKHKPKLRELHEAKLTRQQILEIVSKDFEDTIPTYGQLCTKFDKWGFHVYRQNERKDTTPGAKKGQRKNTKNQQPEPELQQAFSPESSNGSTIVPDVGVLHISDKSSPMTFGTPQTQYLEPATRSASTPWAVHSRNVSAEHASTPARGPICEPRSNYQNTEYGHQEASDSGQAQEWVSSTSLSTATASHGNRSSMTDTASSRTSVHHTISDHLNDIEPEDPAMIAARTRKADDALDAVQCYLRSIGHKQSHGEHVLAMTSLAAYLFATRSYAPSFRIYHKIYDIMSQDMECDDVSRIGALMKCYQSAVEPDDLDFMQRELHQAMRWNFSESSLPATVANMLQTVEAQISEGFNLAQGSVLSTTSITRNLNQLMNKGSNSTYPGTKRLYWRLRLLTHSPFHSGCKRLLRLMSELLSWAWAAMTILKFPDPALKDSGCLCKDGLYFDFVRMVTTVVMEAAMGFEIAQLIKLNSIKAQTKCECVMCADLETTFEICSIETFTAIALLTVDRCRLDWTSCHPGGRHQELTPNTILREQLLPTVESLLNQVAADIQAAATIHTSDLYDDFLSCVWSTFETHDLPLKNARFDASTEKVEDIMTTLITEELNKSHPARNSTLDDGKMPIFTYGENNDDELANKITAKRTRSNASALTKSSSNASERSMIRIRKKLKYRTSADYLNSMSSYSSLSSRNSMNRFSYVTGLPELETTLEDDEFDPRDLPPDLTPDEFRSMQRHDSWSRRSGTGHFSWVPSRPGSTVSRGISWRESNVPSSRPGSTTQNGSFNRKYSQKDVVPNQQAIQNQRGSVNHATNGSQSQTQPSVAQHAAQLPPTGLYTIRSKQAQPQPPIQNGSFQQRDPQQQIQSQPYAKAAAGSQPQMGPPIQTGTQVPVRQDSTKTTSSKESQKGRWRNEQKRVEGILKQDAVMGMR

>CF317_000374-T1 CF317_000374

MLSQLLRVGVFALILAATATNAQDASTSGYVGYNLTLEGDQDSVIYSTENTRPNASVAYPDPDVYLNASVHVNEIDIKVENLTAKINLDLQVLSLLQFNAGVEVSIDQVQLLIENVTAKVLLEARLENLVAMVNTTLNSIDLNSTIAALGQTASGVIDDTVGGLTGSGSSNSSTPSDALRKRTFELANNILYSVNDYSGNKHTNRVLAQNGDLVDESLNNDGDPQGQRVVGNYEDDMTFAGHEQTVVRDGEEITVLEYKYSPYDGISAISAIYLNSSGSVIGTQVLSESFAGGSSTIGDL

>CF317_000375-T1 CF317_000375

MSTALWRTLLAKLIFVALALSTNAQTTSSASTSPSTAAPTSVSASQTQTSSTAAASASSSSQPPDVLLNVPQLSVGRIELTVDDLQADINLNAQVAGLVTLNAGVAVSIQKVNLTIVDVDAQLELIVRLGHLVDIVDRVFESLDLNPMLLSIVSNATSAASGATDALGNVTSALDKRTDDVDGLLGSITQAGTTLSFLVDDLGNIVQQISGGASSIVGSYSQNMTDTGGSQGLGGGLTQKTYSYAPLGAMVDIVFNKAGRVVQATVKRADSGDDGGNTSDSSASGTGS

>CF317_000376-T1 CF317_000376

MPQISASTGDSSRSSGNSTQDDTEISSSFGSDTMSDDSGSDIQSVDDFDDNTKQQVSTRRRRTKKNHNEYIARKYHHLTARRVDLVGDYAGDEPFFIEGDSLLLQAFDDSMLDFTEGFQLLHAVYNVEKLLHGLTQRKCNYKIVFFESNQALCVPRNADHANASKYHLARAVIIRHLRANLPVSQPQITINTFQSWNSPEFLQVLQEMPPYFLMAHDGAQSARPSSSGAAAEPSPVDHSVQSTALRQMILFMVGRNFNVALINEIQFQDTKVMTVVLESRKRGDMIRVEEDNCQLQKEPDVEVSKYADEIMAINPTLTERQLLAVAVVSNLLSRPDTTVSGPIWSQLCSRFLMHQALLMQLPLTSRRLQVDSSSDFVQCFLGHVASVATCLLSSAEWEELQSDNQTYCDVADFVDGRLFSQLVHGEDVPRADVNVTALYARLSSVVTTMTGISLPTITNPEMPAPQPREVPDASTRPEKEQSSLGVLPFSNSVFDNHLESVRLEVDRHPVVQSMASRKVFKEASHWHNAKKPLMAKSGHVAVPSGKTSREARREQWFMAEMRTYAASLTNAVGKSLEPETIVVGAPVKPGIASSGEVDMRSGQTQDAVPASEKKVTDSNKGGGGKAAAKNKAKQERLDKIVASQAKKADDAETKTSKAWQMVCKDFDADGDPLSRYRKAQVYMGNQSSTWKETMGAECELYMVNCLVLCWKEACHKQLQAQSLHIPALIWSHVRSISQSSNLSKAIVSAIVSVSESLRLPGVTQSLQSTTERRLAFSFEPLNKSSELSVNLAPKLFQLLHCGPYLDRSFDSRPDSRVSFQPDDWQRRVLDSIDSNKSAFVVAPTSAGKTFISFYAMRKVLEADDNGVMVYVAPTKALVNQIAAEIQARYSKKFKYGGKSVWAIHTRDYRVNNATGCQVLVTVPHILQIMLMSPANARWSSRVKCIIFDEIHSIGAAEDGVVWEQLLLMAPCPVIGLSATVGNPDEFSQWLACTQESMGHELVTIQHPHRYSDLRKYFYVPPKKFVFNGLSDGRAIGNLDLDSLPGFNYIHPVAALVDKSRGIPADLALEPRDCLLLWQAMGIVCTEAYPVPLSLSPEHALPAVPRKADILEWEQGLKGLLREWMSNPASPFDRLLRELEHTFREGKRESQYITLPSDADPSEDKLSEEDDYIRASLALLCRLHERNALPAILFNYDRHLCEEACKTIIDQLQHAETTQQKSGPKWQRKLERWEEWKEAKVKTSAQSAKEKKSKTTQKKRQDGDDDKMSKADMLREATSAEVDKWESFDPTAPIDGYHFADHTKVQASELEDYVKQLRYREIPSYLIDALGRGIGVHHAGMNRKYRQVVEILFRKRFLRVVIATGTLALGINMPCKTVVFAGDSVYLTALNYRQCAGRSGRRGFDLLGNVVFHGISRQKVCRLISSRLPDLTGHFPITTSLVLRMFTLLHDSGSSKFAVDAVNSLLSQPRLYMGGDSFKHQTMHHLRFSIEYLRRQHLLGVDGAPLNFAGLVSHLYYTENSSFAFHALLKEGYFTDLCASIDENEKDTLQTLMLVMSHLFNRVVCRRADAEFREKTVKHSSSMVFLPPLPAKAASILQEHNKQTLQVYQTYVRTFVDQHITHDDDSLPLTDTKFGGNDDSAASDLHTLPAASVRSTFVALSGLGDDFDSIHDLCSTTRSGVFLEEAVIPYMQVYPVDMEVPLNAWLLDFLKHGDVITLEKDNGIRRSDIWFFLNDFSLVLATIVASLLNFMKLGDVDMMNVRGGMDVQEEAKDDEAVQTEESTDASTTDVGSSNTADKTAGAPAKKTKRVVKDSWEDEDSEDDRQPRSGHLADGIDGDIESMTDDLDDWDIEEGGLRNVLKAFQKLQVEFNEKFRAMWA

>CF317_000377-T1 CF317_000377

MSGGERRQSQYGAPYQSSGSGRPAPGQPLGPPSQDQYSHTAPSPSRGGMAGQPSARPYLSDYQYSYQPPQYQSQIQYPSSYLQDPSRPHHLQQSPGQQQYAAYGHGSVLPPVGQQSMYENMPQYQQQRQSAAIEVMASQFGGGELPSYMQQNESNTPVQPPSQYLSSQPEQHYGSVSYSRPQLQPPFTPSPADYSMLEQPIPQQAQEETNARQVTEEGRRQYEQQLRATFDAITAGRVTEASSKLIGVTEWLVGSVRALGLHHDDEESHEQRIQIWRELNHAWEALGYKQKSITENALRTHQAPPDLLSATKIQELIEQLIQLCDQIEKYGLVDYEMGIWEEQVVAIFTQCLDILPRAPARAESGSAEQ

>CF317_000378-T1 CF317_000378

MAQKALSSFFGRATWTCRRCRTNQWIQRPRFSTSTSRRQELEINAAPEIDFERGQRSDAPARILPASPSYFTGAPVFNDDLLLLQSLMNRFQKVPQVPADKAPRMAWLRLSQYRSASGERVGAAKYASILRLLTRLNRIHPQLRSEEVQEVLEKFLRPGAQEVQKAKPGTIDVYGRSKGVGRRKESSAKVYLVEGTGEILVNGKSIVQAFPRVHDRESAMWPLKVSERMDKYNVFALVAGGGVTGQAEAVTLGLARALLVHEPALKPVLRRAGCVTRDPRAVERKKPGRLKARKKPTWVKR

>CF317_000379-T1 CF317_000379

MSFGKLYGFKGNARTMVLLAVAKENNLDIEFVETNPGEGLSAEYQKLNPLKRVPTFEAPNGWVLTEVIAIAIYFTSQNEKTTLLGKTKQDYAQIVRWMSFTNSEVLVNLGTWFRPLIGRDPYNKKNVEDAKARTLEAIAVIENHLTVCTYFVGERITLADLFAASLLSRGFQFVFDKEWRSQYPATTRWFETITNQPVWKAVVPETTMIEEAIKYTPPKKEAKPKAEPTPKAEKKAAPKEEEEDEEPRQEAPKQKHPLESLGKPELILDDWKRKYSNEETREVALPWFWEHYKPEEYSLWRVDYKYNDELTMTFMSSNLIGGFFARLEASRKYLFGACSVYGVTNDSVIQGVFMVRGQEALPAFDVAPDYESYEFTKLDPSKSEDKTYVEDQWSWDKPVEVNGKKFDWADGKVFK

>CF317_000380-T1 CF317_000380

MSTSTSTSRPIPQATRIRNRRKRYLEAHPEYFSPDLESADPLLFDRLVRRFFTPADREIERRQKGYAGSLEADLLRSEAKLDEAQHPDPDTTYARTETGEIVANVGDEEELSKEEAYERWYATMEQRFLDGNDADFDYGVVDESEEYDDKRAEERDAEEEYFDREEPDFGPREGEVKAGLEGETGVQDF

>CF317_000381-T1 CF317_000381

MPTYCITGANRGLGLEFVRQLAQSPDNTIIATTRSLSNDLSDLKSVASQTTHILECDTADQDSIQSFASSTKTALNGAQIDVLLNNAGANSVPAQTSLSLGAADLHREIDVNVLGPAKTTEFLIAQGLLSQDARIVNMTSGLGSMSVGLSITPRKCATYSISKAGVNMLTVHQSGDLREKLRGVVVICMDPGWVKTRMGGEGAMLEPEVSIGGMLKVVHGLGDGDNGKFYTYTGEEVKW

>CF317_000382-T1 CF317_000382

MPDKDDCPSLVTDNGTEDSGFAEDLRTLPDNEDIDLAYVQTDNEPQEVDDRDELDIVGERMHDFVRGLPGGRN

>CF317_000383-T1 CF317_000383

MSSAEENLSNVHVTEYAAISYTWGPASVRATIVVDGLPLDVPKSSEEALRFALEAAELPVWIDAICINQLDDDERNHQVSQMKEVYSKAEEVLVWLGHDTAKTTLLARSSVETIVSQARQCTNNLEDLDTKLWAGTGSNRYYVYSDEPLPPSINWPALYGFFSVAWFTRLWPIQESVNGLFELLQLCIYFDATDPRDLIYGLRGLIPSTSAGSRGQSLAIKPDYAQASLVDVYGLATRVAIAEGSLTVLKRAAYIRPPAVPKTYSNPTLQLGKFPSWVPRFDWTWDPNQGSPSAISAMTILGAHNSVEPEMRPPLKSHSDELWMAGVVDGEVSVCADTIFSPCLLANEDDLASSLRAAWKLAQGCAVPSYVDTGQDDACLDSFIETLVAGKNADAELIEGSTAFKASFKAFMALHGGLSGAHNMPQATCQAQSEQQQSFLQALKENAMNRKFFVTSKGRLGLGPQDTKPGDTVSVLLGGDAPFILKSKSGRMDMYRLVVEAYIDGIMKGQLIASIHQDGELARRRRWFGLC

>CF317_000384-T1 CF317_000384

MASPDRFTYPIDTTHLPQNESKTILVTGVNGYIASHIALLLLRKGYVVRGTSRSDSTPSRAFDEAVRGTHAIIHAASPVDFRLGSVDEFFRPAIEGVQSLLQSAYLENRDHKGQISSFVQLSSIAAIVDKWRFPPVSEGGVENRAYTEDDWNLSGEAVARKSESDGSFLPMVAYGASKAAAERSMWTFIETAAQSDGGWVGPACTAINPGVVTGPPVNWPNQPDALNTTLLPVWNIWSGKCKAEGRLPPQIGGATYVDVRDVASLHVWAIEHPQASRGQRYFATNGKAPPQGIADTIRKLYPHDDKMKERIILGESGRGYVTAFGWPGDEPTCKATKAYQALGVERFRGFEESIADTVEAFRMQWPELGL

>CF317_000385-T1 CF317_000385

MAPATGSKGAGDEQVSINPLPATGHDNTQSSVTTSQEAYDKVGGGSEKDEKVSVNPIPATGHDNTQSSVTTSKEDYEKTG

>CF317_000386-T1 CF317_000386

MAIENPFAPPQAQDQKLTMPMPPPLLHTRSGNQLYDRERPSTPTSNHIVSPSQTPQGSPSKNHLPPGAHDLPNVFENALKLQPPSHPRSQPGSPTKETQDPFSDKPARPQSPTRQSNKENAPGSPTRFGFTQNAAALARDAPYRTESRRGPQQRGLTAEELEKLQLPKVKRLANVTQLYFLDHYFDLLSYVHQRQTRHDAFKEAVPEPPTTPQADYDAALHKYLGRERANLRKRRTRLRHGDFQILTQVGQGGYGQVYLAQKKDTREVCALKVMRKKLLFKLDEVRHILTERDILTAAKSEWLVRLLYSFQDEEQIYLAMEYVPGGDFRTLLNNTGVLHNRHARFYIAEMFSCVDALHNLGYIHRDLKPENFLIDATGHVKLTDFGLAAGMLSPIKIESMRIRLEEVGETKVPFGMPAIEERSVDQRREGYRSLRKKDINYAKSIVGSPDYMAPEVLRGEQYDFTVDYWSLGCMLFEALAGYPPFAGATVDETWQNLKRWQRVLRKPQYDDPNYFLSRRTWDLITRLVAGKDNRFKNINEIHKHDYFAEVDFNALREQKAPFVPELDSETDAGYFDDFGSEADMAKYKEVHDKQRALEEMADRDDKMNKGVFVGFTFRHRKPAVDNDDNKPASPRKQIDENTFGTIF

>CF317_000387-T1 CF317_000387

MSHPTVISQAPWKKTFEEHLQHIGGPSAEFYLATVTPDGQPRVRCCIHRGFWAQLPENKHNELPKNPAIFQSDCPVFTDDARMEKTYQIFATGKGKGSREQSKSGTGGGGPVEAVYWVKDTMTQWRIRGKAWVVAADDVEGGDEAQNSGTVTVKAEVGRYMRPVGDHETKNSEWSWKREVENNYENLSPGMRGSFMNPPPGHPVSEGKDTQAGEDLGQKGGHLADDDLARKNFRVVIITPEEVERVDLSDPAKARRWIYTLAEEFGGPGGNEGSKSNGTEWNETETWP

>CF317_000388-T1 CF317_000388

MDLSRKRPDQQNPQTSNRATATMGEAIKMTHYPQAQSSFKPPLIAHDNHFLGDVSTSGDDKMPITAGFYHFKPGKPLEYTYTYEEMKIILKGSGTLKDASGQEVHAKAGDVFYFPKGSVITFSTEEPEGVQAFFCGQRAKGAA

>CF317_000389-T1 CF317_000389

MEIEIPSPSVFLKRSPVLSAAVPTPAKRATKPRGTAPTRPVPKFNSATTGTTVQKGGVTKPKQSKSRNGCMTCKKKRLKCDETKPTCVQCEKRSVECEGYKKDYKWRSFEETNKQSRVGKAKKVQVASFQASSEPQEVSKPAPVSETAHSHSKDQASSSPGLQHAFSSAKHAFNGPPPSRQTNALTSSPRRFSPSQFEPLPILHPGFSPSDFDLPSPFPSSSNRQQGRQEHGSSSPSLSTGSPNLADLLLPGTKLRQPPDPSEMRPPMSPLPYQPGSMVLNGSPGMAVSNDEEFDEEVVRDQMPSAPSNTSESQWTFRASSPAVSEASSTSSKSTNMSLIRAPPLDLSSPEMLMLRFDRETCGILSVKDGPTENPWRTLVWPLAKDSQALYHAISSMAALHGTADNPQLRLVGMAHMTKSISRLSAEMGQMSLDQALATALALALGEGWDDKISTGVQHLKGARTLLNSALVQRSRNLQTRQRNQEEARRLRFLCNTYVYLDVIARLSSSDEQESLDLETILDIVDQPFGTMEVEVDPLMGCATTLFPLIGRVASLIQQVRKTSTNSLIVVSEANELREQLLRWQPPDINFVEQPEDPSSDVRHAVQTAEAYRRAILLHLHQAVPEISSESAHAQAKNILTTLAGTPLSSRTLIVQIFPLLVGSCEMVALEDRQWVTQRWQAMLRRLSIVNVSSCWKLVQEVWRRRDSHIQDQARKLAARPAGRNVSPGLFIPPNLKRKMPTADAVTDDDFFDTFGQENVLLRDCIGRPLKRRMTFGASTNLSSGHISDGHMTPLHRRHTDISISNLEPQYTVRGNLHWLGVMAGWDWEVI

>CF317_000390-T1 CF317_000390

MSLFRDLFDLPKIVSPYIFRPLTGVEIFLPHARPVSWFSGSTAFQPERDIPSLQGKIIMVTGGNAGLGQETIYQLAQHEPTKIYMAARSAEKAKTAINEIRQWLREKERATVPDIEYLQLDLADLNSVAEAARQVLACEKRLDILVLNAGIMATPPSKTSSGHDLQLGTNHIGHFLLVKLLMPLLLSTATSIELSDVRVVTVSSEAYNIAPSDFMDLIADHELLCSSSNYTRYGISKAANIVFAAELARRYGPQGVTSVSLHPGVILTQLYDSTRNANFIVRLGLPSFARLMFDDVPRGALGQLFLAAGAKKETLMNGGYYTPVGRRQDVALTNNEGQAGRLWQWSDEQVKDYTC

>CF317_000391-T1 CF317_000391

MDMSSSSSSSSSSSDSSMSMAMTFTNTHTTALYSTAWTPHTTGQYAGTCIFLVCLASLLRALLASKHILEQRWIAEARNRRYIVVAGQTPESERIANDPDAKIGTYISPQGVEERVKIVQAAKQPVVPFRLSVDVPRALLTLAIVGVGYLLMLAVMTMNVGYFMSVLAGAFIGELAIGRYAQYEEHEH

>CF317_000392-T1 CF317_000392

MLLLLLTLWINTIHAVRILGSPPRYHASEIEGFNWSTVVPSKQLRWHECYDSCQCARLQVPLDWSNSSNTNTVAIALTKVPATVSQEDPTFAGSILINPGGPGGSGTDEVIWGGYGMRDNIIDSEDKHYEVIGFDPRGVHHTTPSVSCFGSEWDREVWQYRNWAVGQLDSSDNAFNVKRASYESFTDLCAQSEVGKFDDGTNMRQFVSTALTAQDMVAIIDALQEETVISAGSHEDHPASQTVLASVKRPALLNYWGFSYGTYLGNTFASLFPDRVGRMVLDGNVDPQDYTATGWLSNLYDNDKNLHWFYYACFHAGPKCALFDAETESLFDLEKKMIKLLERLREDPLPVVHDGAADLVTYYDMTNLIHGAAYAPLYFWPGVAQVAHDLLNGNATSITKYLKNLQVPQGPEPTNPDSPRDGDDARFTLKLSNDSLPYPPDYPGGLDGATSILCGDGEPLDSLTKHDWQLRLSQLKNQSLIAGPFWAALPFACQRWPASLRPFERNRFTGPFESKLVDYDERGSPLLFIGSTADPVTPLRNAIENSKRHEGSRVLTQDTPGHCAGPVNPSQCTYEVIRRFFADGVLPEAGKVCLGDRSAWDDS

>CF317_000393-T1 CF317_000393

MSSPQPYASSPVTAGRRGKSRFTFKHLNLLSQASISCPLRVIALIDYDAFYAQCEMVRLGVADDQPLAVQQWQGLIAINYPAREFGLNRHVTITEAKQKCPQIICQHVATWREGDDKWAYREDSFEHIHTDKVSLDPYRLESRKSLALVKEILPAPPVQRVEKASIDEVFLDLSAQIHGILLERYPELRHAPYDDPSEFLPVPPTTALNWEADGLIDLDEGQAEDDDPDWDDVVMNIGSEIVRDVRKTIKERLKYTCSAGIARNKMMAKLGAGYKKPNQQTIVRNRAVQHFLSGFKFTKIRNLGGKLGDQVVQAFNTEEVGELLKISLQTMKSKFGDDTGTWIHNIVRGEDSSEVNSRTQIKSMLSAKSFRPSINSFEQAVRWLRIFVSDIYSRLVEEGVTENKRRPKTMNLHHRQGGQMRSKQIPIPGGKKIDIDMLFDLAKNLLQQVIVDGRAWPCANLSLSVGGFEDGITNTRGIGTFFVRGDPAKALEPPSRSTSATPDQEEGRSGKRRRLDNDSSIGRFFKSESTAGDEDSEPEDEGAVESTEDATTSVSAAHILQGDMATYPCMDCGKEIPEAEKEEHQDWHFARSLQAADRCSIAGPTNGRPPGKTVVSQLKRGTNGRGRGRGGKAERGQSRLAFG

>CF317_000394-T1 CF317_000394

MSNAQSLIRGVLRPLVRPSTRPACTYTPRTTLRLASTSQSKHPPGFTPPTTEDLYELRERVQEFTRREIPEEVAQRTDHTNEFPNDMWAKFGEAGLLGVTAAEEYGGLAMGYQAHCVILEEISRASGSIGLSYAAHSQLCVNQICLNGNKDQKDRFLPGLVSGEKIGALAMSEHSAGSDVVSMKTTAKKVDGGYLINGTKMWITNGPDAHYIVLYAKTDPEAKSKGMTAFILETEPAKGFSVAKKLDKLGMRGSNTGELVFEDVFVPDENILGQVNRGVKVLMEGLDVERLVLSAGSLGIMQASLDLVLPYTHQRKQFGAPIAHNQLVQGKLADMYTKLQASRSYTYATAQKVDEEGQIRTQDCAGAILYAAERATECALDAIQLMGGTGYVNEIPAGRLLRDAKLYEIGAGTSEVRRMVIGRYFNKEYADQAR

>CF317_000395-T1 CF317_000395

MASLRSAPSVAVLRSLRTALQQPFLVTTKHCSRSQSRCIASFTDPRHAKAISVLPSLVDKSSQEYRENVTNLESIMQKYTDLHQKISQGGPEKARQKHLDRGKMLARDRITALIDPGAPFLELSPLAAHEVYGEDVPAAGIITGVGTVEGVTCMIVANDATVKGGTYYPHTIKKHLRAQEIAQQNRLPCIYLVDSGGANLPRQADVFPDRDHFGRIFFNQARMSSMGIPQIAVVMGPCTAGGAYVPAMCDESIIVEKQGTIFLAGPPLVKAATGEVISAEDLGGGQLHSSVSGVTDYLAVNDEHALVLARRSVANLNFPKEQPAPYEVKEPLHDPNELSGIVGTNLKKQIPMHEIIARIVDGSEFSEFKKDYGTTLITGFAKIYGYPVGIIANNGILFSESSLKGAHFIELCCQRNIPLVFLQNITGFMVGSDAEKGGIAKNGAKLVTAVACADVPKFTVVVGSSAGAGNYGMCGRAYSPRFLWMWPNAKIGVMGAEQLTAVMETVGKSVDPELRDRIERESDAAFSSARLWDDGVIPPAQTRHMLGMGLKAALGGSAPKQDTRFGVFRM

>CF317_000396-T1 CF317_000396

MSYQTRREHNIVVLGAGGVGKSCLTAQFVQNVWIESYDPTIEDSYRKVVNVDGRQVVLEILDTAGTEQFTAMRELYMKQGQGFLLVFSITNMNSFYELAELREAISRIKEDDDIPLVVVGNKSDMEDDRAVPRARAFQLAQSWGQKPYFETSARRRTNVDEVFLNLCRQILQKDNIDKTS

>CF317_000397-T1 CF317_000397

MSPRRSSRARSSQQPTAPSQANSSSSSTTTLKDSRNPRNDIPRSNSEGRGSIQRSESVDDADAMSRSEQSAPRRSRRGAEHEKEPLPKQPSVDDDDNEAIEDDVTRCICGHADYPGPSQGIREQYGAAALTEDMGNFFVQCDNCHVWQHGGCMGLHDESIIPDEYYCERCRPDFHKIFKPNNMYSQPDPLLPAMANVPHSSTRSSRYLPVLDGSSRPAPPSAADHARMKDLKNKALQATKRRATMNSRTAYDEDEALRKAIEESKADGTLGKRGREDSEDRRPTTKRQRTATSNSDTSSKRSQSPDTDKTLTGKNGSKLRGAAARNNREKEIRERAKQDAAAQRAEAASKRNARSERRRVEDSPPPTPTISPSKPAEPKSSKAKADTPQPARAKARGGARPGTRGKRVGRNQYTKDLYDATDTPHRFDSTDRNGHHSPHGINGDSGRSSKARTHPARMSLNEMKKRVAAIMEFTSQMQTQYASSSNASKNTSSNPNGSGNHSRSSDGRKGNSTPNGTQIGGTPTSKLIEAVTAGLQDNDNVKMSLVDEAEFTKLGSTQMMEALMKELVGWQSQYGVYSR

>CF317_000398-T1 CF317_000398

MAPDINALRNFQTDGYANNGVPRDPKYEIPDIIMHAPTVRRVRVLSIGAGVSGIMNAYHIQKELENVEHVVYEKNADIGGTWLENRYPGCACDIPSHAYTLPFALNPDWPRFFSYSPDIWKYLNKVCEVFDLRKYMHFNHEIVGCYWQQESGEWLVKIKETKTDGTTRLFEDRCHVLLYGTGILNNFKWPSIEGIEKFKGRILHTARWDTNYQAEQWKGDRVAVIGSGASSIQTVPTMQPHVKHMDVFVRTGVWFVQIANNYGQNHEYTDEERKEFHDDPTKLVEHAKSIEDQVNGLWGGFYKNSTGQVEGQKALKERMAEHIKDERLLKGFTPKFGFGCRRITPGDPYMAAIQKENVDVHFTAVEKITEKGVVGADGVERECDAIVCATGFDVTYRPRFPIVGQNGIELADKWKVNPESYLGLAIPGFPQLLMMIGSSWPVENGSVMGPLYHVSQYALQIIKKVQSEYICSIAPKQSVTDSFNAHVQEWVRHTVWTDDCRSWYKDNETGRVNAVWPGSSLHYIEAIRTPRYEDFEITYLGPAKQNPWAFLGMGYVRDLVEQSDVSPYLSVDNIDPKWMQANNINMDKVLESKVERTKKEWEGKTAEEGDTQEHLKEADIA

>CF317_000399-T1 CF317_000399

MAANLRYESVRTADDDGDEHNALELAYADDASSVDGDDGKFRLDEAPEIQYHPTRRSRALCACQRLSKTCIALCAATGVIIVLFLTSGGYLAYKSGLIDGQSPPWYPSPRGGTLSTWADSYDKAHKLISRMTLVEKVNITTGTGWAMDLCVGVTGEAAHAGFPALCLQDGPLGIRFADHADSFPAGITVGSTWNRELMRKRGEEHGREAKLKGVNVLLGPSMGPLGRNPAGGRNWEGFGSDPVLQAIAAAETIKGIQSQGVQATAKHYIGNEQEHFRKSFEWGLPEGLSSNIDDRTLHEVYAWPFAEAVRAGVASVMCSYQMVNNSYACQNSWLLNGILKDELGFQGYVQSDWLAQRSGVASALAGLDMSMPGDGLFWQDGNPLFGDQLTLAVLNGSVPMDRINDMAKRIVAAWYQVGQDSWESEGPNFSSWTNETTGRLHDAAADSDETGVVNHYVETSTRKSRRVARMVAQEGTVLLKNEGSLLPLSYNLEELRLGRRAKVIVVGEDAGPGKGRNHCEDRACNQGTLASGWGSGATEFPYLVDPFLALHKSFNVTGVELTKALDNTLSKADKAKIKQQDICLVFANADAGEGYLAWEGIRADRNDLELQKDGTKLIREVASLCGGPVIVVLHSVGPVIMEEFADLPAVKAILLANLPGQESGNALADVLFGRMDASGRLAYTIGRSLAEYGETAPVLYYPNHIVPQVDFKEGLYIDYRHFDKNDIEPRYEFGFGLSYTDFEYSNLTVEALKVKSSLPAPPPKGLSAPQFQNTPPDPASCVYPEGFRRLKKYIYPWIKDTSQVKQGTYPYPQGYDIARKPSQAGGGEGGNPSLFENHVRVTMNIKNIGSRSGKEVIQLYLSYPENISDPVSGEAVDMPVKVLRNFDKIELHAGETKSVEMFLTRKDLSYWSVVQQNWVMPENDEFTIAVGRSSRDIRLRGTY

>CF317_000400-T1 CF317_000400

MLASRALKPAPLRRLAAGSFAAARPTPVLRPQAQSIARPSQILRPTMASAYSTTAPKGSAMPSKAAEVDYDPEIKDIASYIHNYEIKSDVAMDTARYILLDTIGCGLEALRFPQCTAVLGPIVPGTVVPNGTRVPGTPYELDPVNGAFNIGAMIRWLDYNDCWLAAEWGHPSDNLGAILAVADWISRTNRAGGNLGNGKILTVKDVLEAMIKAHEIQGCLALENSYNKVGLDHVVLVKVASAAVVSKMLGLNERQTADVVTQAWVDGQSLRTYRHSPNTMSRKSWAAGDACQRAVNLAMKVSQGQSGVPTVLSAPTWGFYDVLFKGNKFEFQRPYGSYVMENVLFKVSYPAEFHSQTAIECAQKVNKILKDMGKTAEDIKEITCRTHEACIRIIDKQFKAMDNFADRDHCVQYMVANMLVFNRLEATDYLDGSEAATSPLLEDLRQRIKCVEDPQFTKDYHNPDLRTISNALTVTLNDGTVLDEVVVEAPLGHRLRRDEAKPEILAKYKRHLGHHFPESHIKQLVELGTDASKIDSMEVDKYMDLYVKEKMEW

>CF317_000401-T1 CF317_000401

MCRKNVILFDCGHTRKLNITPCNDAGVECAVPERILIPEGGGIGIEAPKHKTQWTCAFKCVPGRASHEPLIDLAKENADAVKAEIDHKIEVLSVLSTPFGTPASEKGGFSFVKSPAIERGGFNFDQRFVKSVTRRLQSATPSPGARETTTFNFKLTMPGDKVKDNTTNKLEAQAPAFAQIQSESPVPLPSASASDME

>CF317_000402-T1 CF317_000402

MEIHSVLPEERARTEKGEMLPWGYRYADSSKNARQPEESGPFGRNRSIRYTSSVSGGRRASTRAGTTPSRQKENSAVADFSRLFAKEQATRPDHNSLPVAVSTEGGGPESAAVEKVPTECLLYGYRAKNSEWKVLSRYERIVTPGIICEDYPREDPMLFTSSNSPMGFNRSSITVHQHLSREALQKSRVYKGGNHWIKVTFDSYEAAERACFYSPVEIDGCMVTCEMWNGRGPLSDAPVPTSAGVDGALLSTRPARTVAVQGGRASAVAGFEQAMTGTLPRSHTVGDVQFGQPFAGSSAGGARDDMDVDVLGSDTASSATVTSPPQASTGLQVQQTTGSSLRSRSVPNLPSQNASADPALHNSSRIPGVRKIALRPVAEALPPQQSFLERLLRTVPVVNWVLGFTTPVKKDGVEKAGEKEKVGLIGEGPAVKDDGTFDQADNGWYWKLWHTLDSVAGTDFCGLKED

>CF317_000403-T1 CF317_000403

MDAEKLKRMQQSVRIGIGKGTPRRKQKKVHKSSVGDDKKLQSTLKKLNTQPIQAIEEVNMFKEDGNVIHFAAPKVHASVPSNTFAIYGNGEDKELTELVPGILNQLGPDSLASLRRLAESYQNMQKKEGGDKKEGEDDDDEIPDLVEGENFEGKVE

>CF317_000404-T1 CF317_000404

MSLRPLIVNALSKHTATVIMLHGLGDSGAGWVSLAENWRRRNKFDHVKFIFPNAPSIPITINGGFVMPGCPRATSFSDLNAVHDESGIMKSRTYLNSLVESEMREHKIPSERIIIGGFSQGGAISLFTGLTIKQKLAGIFGLSCYLVLHDRVPEYVKEANMVNQATPFFIAHGDADQVCQYEWGVETARVIEKDLGHKVEFKTYPGLPHSAAMEEIDDLEKWISKCLDAKPAAQGETSSGTTSV

>CF317_000405-T1 CF317_000405

MPVSALTENFKNNNGDQTTETSSPSRDIPISGGQSLTREVSRSAHTRESSFSLGTSPDQHRHRPTFAHPSQDSGSPLLTRTMTGAPPKKLQPFREQDVKILLLENVNKTGQDLLREQGYQVEALKSSLPEDQLIEKIKDVHVLGIRSKTKLNSRVLAAAKNLIVVGCFCIGTNQVDLKYAADHGICVFNSPFSNSRSVAELVIAEIITLARQLGDRSNEMHAGTWNKVSSKCWEVRGKTLGIVGYGHIGSQLSVMTEAMGMKVIYYDVVNLMSLGTAHQVGTLDELLKSADFITLHVPEIPETKNMISTAQFEVMKTGSYLINASRGSVVDIPALIEASRSGKIAGAALDVYPSEPAGNGDYFNNELNSWCEDLRRLKNIILTPHIGGSTEEAQSAIGIEVAESLVRYVNEGVTVGAVNMPEVTLRSLTMDQEDHARVIFIHQNVPGVLGRVNKVLSDHNVDKQMSDSRGDMAYMMADISNVRQAEIDTLYKDLEGLRERIITRILY

>CF317_000406-T1 CF317_000406

MPTKHERISAPTVETVAGMTAGLASTITVHPLDIIKARMQLDTSPNPLMNSTRSVFADILRNEGPTRIRALYRGLSPNLLGNTAGWGLYFLWYKQGQDLVRKAKGYSKDQKFTNVDYLTASSGAGALSAILTNPIWVVKTRMISTSSATQGAYPSMVYGFKSIWQTEGIRGYFHGLLPALFGVTHGALYFLVYENIKIWQRDRKGNQDLSNTDTSIAAAVSKVFAGTITYPSQVVRSRLQTYDPALKVQRKSVAAIAWVLNGVLRKGATQTASVKCEMVGNDLVRVKARVGGKHLYPYVPGVHVYLNQGELGPRTPFTVVDARALEKDETEVKLVAKNSDGPMTGALAEAAKNGSELTLDLEGPYGEAQVYVPELLPRVKGKHEKFLLVAGGVGATYALPIYKALLDAGADPKDVRVIWVVSKEEDVKWAAPFMASGKESPIVIHITQPATSDTGPHQQVESRKDGRPDFDNYVADFVSERLVHSTRDPNSLERDPTKTDQKLYGTVTVMVCGPRGLSSAVRRAVGRHVWDYGRDVRWYEEQFGFGSS

>CF317_000407-T1 CF317_000407

MDVILEAADTYFFDKFWATVLPAQNNIAQNATATFSSMREGATLIPQKQWVWEQATQYFSFPPSEYAWQSAWPRDRTERQFIDLFLIVWLFGVVLYFICATLSYLFVFDKTTFQHPKYLKNQLWMEIKQAQTSMPGMALLTAACFVLEVKGYAKFYDRLDEAPFPLYNYLQFPFFILFTDFLIYWIHRGLHHPMVYKTLHKPHHKWIMPTPYASHAFHPLDGFAQSFPYHLFPFIFPLQKFAYIALFGFINIWTVFIHDGEYVANSPIINGAACHTMHHLYFNYNYGQFTTLWDRLGGSYRKPNEELFRRETKFAADEWNRQAQEMEKIQKQVEGEDDRQYTAQSRGECKKVQ

>CF317_000408-T1 CF317_000408

MVPHPYKNQNFVLNHIAVAVDDVDKAADWYGKHFGFRRIRSDRVNDQNVDGTNAPLYRIYGESLRKVKIAWLSTGNSVGFEIFQFIDPPTDSAETILKDWTLQNQYQRGGVFHVAVTAPDPEAVARRACEDGAVQIGETVTVGDDERAVYLRDPWGIVMEVLSCSYDQLMANQG

>CF317_000409-T1 CF317_000409

MSQDLQSWAVPRLQRLLPIDDESLKEIIVYTTTLSKEAGAEHLKNMLGDSAQAFEFISAFNSRRPDVKPPLAQSETSSRQAVEQGGVPRHVRKQKQKAPLHSAGPVRRPEGYGNVSGAYTKQQDHDRNVRGHKSGNSATSLSDALSLSQQPEALQLPKVSQGGLQTPTPGQSREPSPGNRGSPSLLPPSASGKLISDLPNVRTKQSKKPAHTTQPHSQPQSGTSTPHGHGKGASTTTSSIVDLTAAIAALELSTNPTLSTERRRCDCNASIHPLFTTAPNCLECGKIICALEGLQPCSSCDAPILSREQVNGMIRALKEERGVERTATHNAGVAGGRSGGGTSMFGSSPDVSGDEGSAASRARAHRDRLLAFQRENAQRTKVHDEASDYDMTLTPGATQWMTPTQRAAALKKQQQYIREMEEANRPEWEKKRTMMSLSVKNGKLVKTFERVKADMPKTESEEEIVVEEDTRDEERQKLGGSGAFSNNPLLKGGALIRPMWKAKEGEETMDAGGKGRERKSVWRRVQDDHEDNEQWILDGGLRGYGVESRLMEDGSQQERG

>CF317_000410-T1 CF317_000410

MDTDTTARGMGNSLADDRPRPPQPFRRDLYDTNYASDQGPGPATFEITPPKLSRLPRPRFVPHVMTKSTPERSDCLTAEQTIDLFTKKIEEARNETQHAFSGPDEVADALKPTLTVDLGRARIERLPDAIIDLIITDVERLSLSHNMLRYMPSRIADCKQLRYLNIRSNDFRELPPSIYRMPLLEILDVSKNKITVIPPEIKKLESLRVFSIVHNRLEDLPIELTELPRLKVLKVAENPFRKSIKSVIREKEQEVAFSEMLENERPTALTTEILKYLKAIKPSLAATPILETPEHNEPQSALEQSRPIRRALSGRFPVVPAAFSSDPTSESGETTPQQGKPPSLPIKSHLRGFSGQSNSGALLKRPGIAPVVNGNERNRSNSESVIQASAAARQKRMGIMRRDRTELDSIDETKVVRQSHLRGLSYGSGFRRNGSISSPGAASSSPSSPRDVRKDRAQFVRRLSSLPEHKTDYIRRPIVEGAKGMLYALYQVHPHISGLISASKGRDPRRSGLEMSFYNASVHVDKLNKSLEYTENIDPEDEESWERAEETINNDCAICIKSFVHVTTQMQDSTRKIIAGADPRYVRSLMLLLYGSVVEIKNAIKGFGIEVKHHRRQMSSGATHPITTIPEETTPETPEQQRSLNSDIDDMTQRPLRFRSDTVLKHPSQNGHTLMPVQPPPLQIPHLPASTVNGVYGRSQSALGTSSAKSFATRSRSNSRSMTLTMNSDSSSVASTPRLIEGYHPSPVASLNGSASSETRMIDEQEERRFEQIFIALTKAYETALRAVPITMQHLGRCLDAAHQQRQTKETIEGWTGLLQRSRTCLDLTQALQIRLTNMKVKDSGVGLSIAEGGRHDPSFWVLCKSFLSTFVDLVTEMKAVRKARLIVLPQEAVGVLRPVQKACQEAGHAITTSPWNHMLQGVHPMPAPTPLAPRGGGGEHFTRPQFNRAVTVANGNVSLYSAAVVHQQNQQAQYAHRPTSQRLQIQPTPSLSASSTPALQPTGGMNGAASPLSATSSMPATPLSAALGPAAQATVPSTPASLYGDSFFKGDNPAQAGKASTSVSKDVGQNMGQETSGADAGGGLDARLDQADMARPGEFAGAKAEDNSVKVGTEGGVDAHEAAAGKQP

>CF317_000411-T1 CF317_000411

MSLDPARSRVASRTSTSSPPEDEITTTLDALKNLKLSTWQTYREDLAQAGLLQQLRFQLKDLEAPTNPRDSFRRAHGFQIISSNIHSLVEVWSRSPVPPETKDMIQTLAQFLLVLEAALIHHAGNNRYFRTRVEGGGWIQLKKSLSTLLAALTKDALDKNVIELFYGVLFAVAVGDEALSSIYTNTAKACPPPDRSWDLKSAATIEAQVGKYLNDVAEIAVPDVLPIVVQTWIQYIQTTGKSIETINLALPISLKALLQLSRQNIVEAHTSGLLSAVIPLLFDGQHTLVEKSLYRELALLLFQQGVTNLDDAHHFFSKAATSAAAANFLLEAIRVSRHPASIQFDLSRYGSASVELPTLRKVFPSTQADGYTLTLWARFDNLDPEAHTTLFGACDKTQTCFVLAYLEKDSHHLILQTAIKGSRPSVRFKSVKFQPDKWYHICIVHKKPKAIASSKASLFVDGLFVEQVKANFPTTPPADRNGPPRVQAFFGTPQDLAPNTDGTCMTLWSLASAILFEGTLNEDLIAVMYHLGPGYHGNMQDCLGSFQTYAASATLNFRNETLNAGNENSSHLSIATRQKASNVLPESTILFNISPAAVLDHDDRNNIDESQLIRSLSKTAAKNLFSYTRSGGNAIVINGAVPAINDALVQSNGVAMLVGEPVVTVPQSLDDVSWRLGGCAAIGLSLVSKARTTEEVATAVNILLETIKDSWRNSEVMERNSGYGVLSSLIREKLLPPPGSPEQNKTALAIATTNLDRSGLALKLLKSTLAFLGYDSDNPSKSMINNPMAYKILLVDTSVWRLHNFETQELYFKQFVVFGTDSVHAKFNLKRLSRMRALRRLLEALKSENVSRSTMPAYSTALTALLPTATSAEMLRSMALFITYSLHKRSVTGRFGKAARSRGSSGAGSTAPTNNETANLSQFEIGVEILRLYSNMLCKKGDLSMIRKFATTVTNKWLLYLLSEASPEVIVLSMRILARLLVVHGDAYVRKFKDKSGGFTIIAHRLKRWWHLPALWPACFAILFDIDVAELELDRSFDLFSFVDLFNSKKEYGIVYPEMFEVITGMLQSGLKTMVSSKQHQAGLQVHAAPTPNVPERMSMSTMAPANPLLTVVTSQNVETFETIIRFLSDLQTRSVRFREFASSSSYVQDLLSVLFPVVVGSDAVEATVELNARDSTLSFDGTDVVVQPLSARPPIIRAIDKHSAEQPRRGKVLRRGSSFVLVSREAARSEDISGHSAAEEQAAQTRQIAENNIKHGIVQSVLELVVSIFVDQVLARKDFGGLGLFLKTPPGFVEHQTFFESWILRNTISQLSNAIALDQTVLCEPKVLTNLARFFGHLGNAIFEGWFIGGAESVVDAASAVLEYLQRPDVSQLKSVRLCAQPISILRGVVFRIELLSLSHLDIAETLAFLKKLSYWQNVLLSSQEAQSGYLQLLCYLLYGSLISDDHTVRTAAADLWRTILVQKPAETAAILGQAITTDQKQLASSFEKLVELDNETFLVWIDEHREELDDLFFGTLSKVWDAFVSQENEKTDQSSKARVSRRREKLKGWARDESEREELLRRHDVTFEHWTANIYSSETLKHQRLMQDQSDDLVFTNATFSRMQRELARSTELFTEGKPRRWRVDQTEGRNRMRMRIVEDFTSIDDTQQPKRKVSGSDAPALRLQTQSLRKSTAETIGVTPGGAELSQVSSPLPNNADSASEPFPPVEKSQTLEDMEAEETFELVEDPNAGQEDFEDRNRKVMRSLHRGDQVKHVSNISRVAGLEAVEGLLILGKDYIYLIDNFFQRADGEIVHVWQAPAEERDPYVGMISGRDISTHKPVLKAEEHETRSWKWSDVISTSKRRFLFRDVAIEIFFGDGRSYLLTVLNPKARNDLYSLINAKAPQYGNPDSPHSEVAWRYETLKSADDDPQTLGARFASVFNQLPSLAATKKWQKGEMSNFHYLMLINTLAGRTYNDLTQYPVFPWVIADYTSKELDLDNPRSYRDLSKPMGCQTPDREFEFRERYEQFAEMGDSNAPPFHYGTHYSSAMIVTSYLIRLQPFVKSYLLLQGGSFDHPDRMFFSIEGTWRSASQTNMTDVRELTPEFFYLPEFLTNPNDYDFGMRQTSKKSIGNVSLPPWAKGDPKIFIAKQREALESPHVSRNLHKWVDLVFGCKQKGEAALEAVNVFHHLSYQGAKDLDTIDDPVERLATIGIIHNFGQTPFQVFQRAHPPRENARHKYKRLDSAAESLTRIPGTILDTEERVASLTYAVKTDKLLCSGAFRLNMPPNYDKYMEWGFSDNSIRFYAADSRKQIGLFEHLHIGQLSCARFGDSKTLITAGTDCTIAVWSIHSPSTKAVDVQPRATLFGHRSTVTNLAIARSFNALLSCSAGGELILWDLNRCEFVRRITTSKKLMVECATINDVTGNVIICHDSWITIFTLNGDLLVSQNTSERSSADDNIVSVACYEGAGNEWLERDIMFTGHKRGIVKVWALTIEAGAFTLELLRQLNHLDSSREDGGNVNAAITCILPQPQVVYTGDEDGRVVEWNCVQRH

>CF317_000412-T1 CF317_000412

MKSVISLALAAVAASSPMVIDTIHNDAAPIHSSSNSEPIANNYMIVFKDHVTDAGAKAHQTWVQDIHERQVMKTELKKRGQTSLQEEIYQGLKHTYSMPGLMGYSGHFDEDTIERVRRHPDVEYIELDSEVHTMKEEEPDVEKNAPWGLARISHRKGLSFSTFNKYLHTAEGGEGVTAYVIDTGTNVDHVDFEGRAKWGKTIPSGDPDEDGNGHGTHCSGTVAGKKFGVAKKAHVKAVKVLRSNGSGSMSDVVKGVEWAATDHKKNEGKKGNKGSVANMSLGGGKSPTLDLAVNAAVDAGIHFAVAAGNDNANSCNYSPAAADKAVTVGASTLGDERAYFSNFGKCTDIFAPGLNIQSTWIGSKYATNTISGTSMASPHICGLLAYYLSLQPSSDSAYAVADISPKKLKAQMIGVATKNILSEIPSDTVNLLAWNGGGKSNFSEIVNDGGYNAMSERTPDFFEVAKAGLKSELKDIVAELESLIESA

>CF317_000413-T1 CF317_000413

MRLPGKLAFVALATSAVHVSAQVGIGNIKLCYNKNLTGKCTYPMDVGLNWAKCLGVEDNNKRFGDKGSSIGIVGDISCRFFLAGASDNCKDPQALHSTSPQNVGQTGWDGRRATGWIDAPGIMEWPYEWTFKSYYCVQANNKTTDFVVAECPHKEAGDWCTRDGDWAEWPI

>CF317_000415-T1 CF317_000415

MKLSAPRIFSGRFRESVNISLLFAVLLPASPETKHLKLTMRRRSTFIPSPGSDYDPSELKIDGRHLSFAGLKAAREERLTFALKELPDEIVGILESSHELHIRWVSEEPYDKAGAYLSSLTPGLHVHYTPSKNNGDRLCPLLQRLYSPSMRCSTTNATFSRPDILSERFSSSASLQYYSLLPSLQQFVAWIRRDICAASDVVCKHNAAILNLASYVDFDYDSIAHALTMTAYWSKPPAVLTDPMGRWTTYDHWNMAIDASPNDKTELGLLQLSMANEPSELQMEGILVVVGEDSEPKPVMFSFPSRHHALSSQEAHAQKYLVTFDQPTGLHPTMRISFPSTHKLQEPRDKPAGSTCVLHSYLTLPSVLFADEYAFPTSDLDPLFTEAHNIISLRSFSGEGDLEAPDYAIQQWGSALLLELATPQSASADASEHPWEIKIPLHLRYLAPAAGGKQAVEVPWPIVFWACTAEEGTKFTVNPFDRVNLGYDGLFGPRTMFYHLEPRLQQGGRLLEKIDVPVLDTDIVSHGMAEVLTMALVLGGFLWVSWKVLKEVRWEMKWWKTPGAQRRLEKKAQ

>CF317_000416-T1 CF317_000416

MLTEWLKQKQTQVVLFSSTAIALYGYDQGMMSLINTNYHYLSTMGISENDAMVGIIVSVYYLGCAVGAVMASQFADMVGRKPGVLACVAMAALGNLIMFFAGMVLPQHALAVMLVGRTVMGLGVGGLDAVVPVYTSELSEDDARGTALAQEFQANILGLNMAFIINILVTNTLGKYDEWAWRTPIIAMQIYPILLISGTTLLPETPRWLVLHQQTDRAKRSISAVFGKDQVDDRIKELQEAHEQEEKDGMVTYADMLIPGRDQWHPTVVTIMGQVNQALTGYGAVSVYGPQIFELLGFDTTDAEFLTMGNYLFYLGMMTFAWILIDRKGRRWLMVTGAFWLAVSFAILCVLGGLATHQSKLNIPLLATGIPGIIVLYLATAVFGIGWLVPPWLIPTEIFPSSARANGSAISVVVWGLANFAVTLLTPIMFTNLGYWLFLVFAGSNAFSGCWTWLYCPESGNRTFEENQEFFKEAADAGTWSVRVVANGGYRSLPDEKDNESGDGGDQDAAKKNGRDGEREPLLNQSN

>CF317_000417-T1 CF317_000417

MATRQPPWSHPQSSTHEPELKIWNSLTRSKKPFVPRERQKITWYSCGPTVYDEAHLGHARNYVTIDVLRRILRDYFHFDINFVMNITDVDDKIILAGRKRYLYKQYQDKHRYIDDTVLADVLKAWRMFIARRLPRLSPDTAEPRTFPTAVSEAYGDILSGLSLDGPTAKAGDKEAKVKMYIRDAESCAKALTGDIRNLTPIAFYELAKDPMCDMLDAELGKAIKGDDHHIFKELTEDYERRFFRDMEDLNVLYPDRLVRVTEYGKQIADFVDTIVNNKFAYATEDGSVYFDIRAFEAAGNPYARLEPWNRNDKSLQADGEGALSQTTSGKRSDADFALWKASKPGEPSWPSRWGPGRPGWHIECSAMASDTLGKQMDIHSGGIDLAFPHHDNELAQSEAYWHCGKSTQWVNYFLHMGHLSIAGSKMSKSLKNFTTIRSALERGDWTARSLRIVFLLGNWRDGIEITDELVKQGQSWEDRVDNFFINAVEAVKMSPEQEDTRQASLADALVTANNRVREALLDSFNTPDAMAAISTLITDYNSQPKTTLTLQDHVDIGVFITRMVNIFGLNGNGKPDTNEIGWEGIDIPEAAKRYVEPISQMRDQLRQAAIARSITKEKIEKIVSSVDVPEAEASNGTRPRPSYARAYTEFKDNVLKATTYDNNAMNNEILGLCDQVRDVALWNLNVYLEDRENQPALVRPVTEGLRVARREKEDRARQKEEAKRKRDEEARQKLAKGKLRPEDMFRPPNSDEFSAWDPSGMPTTLKDGTPVAPSRAKKLKKEWERQKKAHEIFLQQGAPEAARNGSARQDVGGRA

>CF317_000418-T1 CF317_000418

MASKLTATSTLQLPNSPSAIPRLGFGVYQSPEKVCENSCSTALQAGYRHIDTAQFYANEAQVGAAVRNSGLKRSDVYITTKILSAAGSVEKSYQKCVESISKLDEGSGGQDGYVDLFLIHSPNAGKAAREEMWNALLKLHQEGKAKAIGVSNFGIAHIEGLKGLGEVFPPHVNQIELHPWCQQRDIVDYCHKNGIIVEAYSPLVRNQKAYDDTLVSIAKRYNKETAQILIRYSLQKNWVTLPKSDTPARIKSNADVFDFEISEEDMKKLDDLDEGEGGAIVQAVKNS

>CF317_000419-T1 CF317_000419

MPVDPTSDTRTNGSPVEDAHTDTMTTKPTTPPPSKPAYPTTLAELTIHSPNDDHTTSSHRSSIYKTLGHLRKSTLSIPNRLTSVLQDAEFTESLWRHLNEHSSLTLCHASAKDGTWPLIPNERSGSWPLDPRVKMTSQESYLAKSPSPSQSSSQPTSPPRTPTVESSSDQPAADLQSQLQLPRPPLPRHHSSQPPHSAPASAYFKSTDGHITQWSFSLRRLNLPLLHILGAAHGAILIDTTRRGKTYPDALRRTVPIWTTVWNRVLFPELAEAESGVCAFQAFGLDESEKAQIEDRLSRFVSECKGLGLDLAELRRRVARPVRCVWVNQPGSMDEWEGTAQTLAEQVSAEREKFAGVVDVNVLVCCSASKVVAGAEMSEEGYIQGAGDDGEGWARNLTARVFWLNRDELMSAVQNGEDVEGLVDRLVTEDKYRSSYGPGHSVLISPTRNLYVGFGEGILADHDFNLVIDVNALNDEPQLKLLGMRCKEGKIGSKMMRERLPQIAARAEKQLRTWPEKKILVKCSTGKDLSVGVVLVILCCFYADDGQVSFPQKHTIDKQFIKQRLAWITSSKPEANPSRATLQAVNSFLIGRPD

>CF317_000420-T1 CF317_000420

MEHNPNGQAAAVAEFPVLPAQVQAQAQAQSTTVMAQPMTDPSATTMAPMAPMQSISADEIALYDRQIRLWGVKAQELIRNANILLIGMKALGNEIAKNLVLAGIGSLTILDHENVVDEDLGSQFLVTEEDLGKNRAEAAAVELRKMNPRVNLSVDQESIMAKMPEYFAAFQIVIATGQPFEMASAVNMSCRMFNVKFYAADVHGLYGYIFSDLIMHQFIVERDIQGNIPTRPGIAETSTRMVMGVETKKENNKTKEIVTKQEMYCPLLLANSSPLPPEATKSRRSKMRVPPLLSCLRGLFEFQKQTAGRSPDASRKEDIALFAKVTNEKHLELQLPHETLSSACLRSFLQNLSTEIPPTAAFLGGQVAQDVINVLGQREQPLQNLLLFDGEEFKAPIYSMQPTFDPTLAMSLDDMTADGVPPQVTSNGNAVATNGGAPDTATHNPQAQPQQQA

>CF317_000421-T1 CF317_000421

MDRLARQDLFDRDVVESTGSNLVPVNTSSARQRSASAELELEPRSSREGGRSKRRKLDDGSSNHHLPPAYGLEGTLLPGNLKMRVLDYPQPEDPLPPADDLARSRIWEADNNDIFRTKKHKCTILMKHQGGWPFTLSNLVIKTPKHDYTHDTSPLQGMVFVSMDQDQLLERTSYYDTLFPLLDLSDSQWCDPSQSVDEPINLPRSQGISTTVERLLPDPNLSAESHPRSPRPWHSEDYDRSDAFMPRRTLYTDTYRPSYTSTSTLTPHQAARARQWQAAAAAAEAETDILDPEPEPDPAISESPSPSDSSTDDHEQDQDQEDETPHYRDNPSFAEVEARQSAFHEQTRARREPGRGERSDNQNHNITYAPSSSYPSLPLYTANTTVAAVVGPGAAATASDDPDDYWTRGPPTAHAYGRPPAGTSTKRLPGCNEMLRGRRASDTTSDGNANGDPNGPLAPHATFQATRDAGGAVVVRFEPEV

>CF317_000422-T1 CF317_000422

MANPTNKHPPSRQDSLDRPPALSRSASAASSHGHLRSKSRGSSGSLQSVGVGNAFQPVQPAHIQHAPLDQAAYFVGDAGQQTHYPPPPNMDDTNIMAYQHNIHQMRPQQPQQPHHMLPQHDMRPMSQHGYSSMQPMPMQYPDGMTGYGMPTQHIHHMRHASEHYEGSPAPDDSNNENGPSKRRKGTASSLANDQELRRLLAQYNGRTLKEVANEVQKNEGSGGKSEKAKQVFAMLWLQETCQRSTNSVRRDRVFARYTERCGNERVPTLNPASFGKLVRIIFPNVQTRRLGVRGESKYHYVDLSLVPDDNDPGFDTFDRLGPSSAPVPERPASSASTTRPLQLPTKQLSLNVIPPRMTMETADFPAPAPALAAPGAQDQTRLRPGPTVHKLDCRYLNTPMIRVTRGNLSTALLNALPSLRPNMPGTLATYLSMPSKTTLCQPVPSSQESPIELPDIHLYLSDENYDHHIAKLLHDLYRSYCIDVIDAFRKCKDKSFFNHHSAFNGKMTVPVSKLFNLECLAPWIQECDMRMYKQILRYMTPLVLQDVPEAVWNAFDRISNRLVGFLINSFEEKCPPHVVAAKTVPAARFTNLLRKLRSAQTSTLQLNRMLEVPQTRTQMWLDMCSVIDPDLTLQECNPPSECFSAAQGILKHDLRALLGPEPDPLVDAAEQDPTSDFARMLQEQTQSEGVLSKEDVSADLIVRLVSWLECLPDAFEGHHPQCLIDWKTRFFRSLMNQFGSGGAISYQSWWYFENFTHQMLAFMTELEGLLLPADDQKTLDIREKEKSEEHVRLYGSTDSAVDEKKRKRASTEVEEHQRAKSPRISRPNTAGGDTEPEPDSAHTASFPPPNAHDSITNPPLLTPTTELPPPPLPTQGAHEENDNDEDDDMAEISRGGPLDLPSFKTGFTSPIKAALAEDARRASGLHEDSGIGMGIDEHHDLDADAIEAEKEAKKFNKRDWFLSSDPVEPGSGVSAAMVS

>CF317_000423-T1 CF317_000423

MSFQDKAQQQVSQLDKELSKYQMLNDFERQSSIPKVYAVLGLAGLYFFLVFFNIGGKFLVDFAGFLLPGYYSLDALFSASKVDDTQWLTYWVVYAFLNVLENLVNAVYWFPFYYVFKFVLILWMALPMTNGAQVVFRSVIQPVFARFFSQSGSTAANLRAKADSATKDQ

>CF317_000424-T1 CF317_000424

MANTHKVYVLEVFSDQNFVRDIVKGVLHTIFFHRYFPSIRPSLYTPTSAVSYSSNRSQESLPVPLPAIHEPVEIAAAIDAHAAALVGQLTSSSSNAGANGSRGEIVVQLFDRKRRKAGMAGGWLGRLGGTHQTEEEVCWEEWVVQVVVAKPRTEADRIKVRRAMETSLQKAAMKIVAIVNRDKDHIPPILTSDQNPFPYKILVNPRQQQTLDQGFGGWT

>CF317_000425-T1 CF317_000425

MFAATRKWLRRNRTGIAVGAAIVGGTYMVGQYVLTKINDARERSTLDKIAKDNIRRRFEQNQIDCSITVLALLPTLTENIVDALPAEQLTQELQQKKQERQQRAAEGGAPSEASSLRDGDSASLSSFQTGSFMHTSQMDQGAMPRPRRTKAQLWNELKITSIVRAFTMIYSISLLILLTRIQLNLLGRLNYLSSVISLSHPPPPGRESTISLENNEDQGAANFGNDFETNRRFLTFSWYLLHRGYAQIMNKVRTAVEEVFGNISPNEGITQQRLSDLVLEVRKKVEGQTEQDRYSTRWLQFVLPPREEEESLLVESGVITPPSTSSASGSDKPSPSPEEQRRTSYVDTSSGPLRKLLDEAADLVDSPSFSRLHMLILNSMFTHLIDNRVIAQLYPQPNVQSPPPSDTSSQIQGPRIQELDSSVTVVPGEPRVKLASILAVLTRQAHAIGNGSNPPNEYLSRVDAEVRELDSFAAVIYTSNLEAGIEQQQGARTEGALKSSHGVGVSEVGDMPVEELVESKLESAWERVTGSTISSR

>CF317_000426-T1 CF317_000426

MDRIVEDRSALFHDVLTHLDNVRANPTTTTLDEELLRRASRNADSHTPRTTLWQILAQGESTLQTLQQDPRPLTRLLEQVVLSLPFNELKETITSEKLEEGLRSPVVPIQLLILAYLLKAADSPSGAAFVACSPSLTSTLITTWLSSESTEVADKSLDAITALLDVDSPSTSTFVVAQAASGEAHGQGLLWRRIFSDAQVYALLFEWTSLTNSKHDVSTKKGLQQATISQGRLFDCIARLAQIEWSQITTSSLPDIERPFMKGNGTTQPFGGILRYAATDMIDPNDYLMEVLRQDFFVKLLGVVGEVNDRGVNSRMLQAIQAGAGAQAGGEMNGNGMHL

>CF317_000427-T1 CF317_000427

MATSSTAAHASESGQRVLLHPLVLLTASDLIVRHRLRQLEGPVAGILLGQQQGTQVTAEHAFTAKLKDGLLDQTDDWTDKEVHQKPVLEVVGWFTLCPETGPLPEHAALHNQLASLYAENAIMLAIHSTEFSGIDGTKGKVPVSVYESTTEGEAPAAENAMQVDDKEAASAQFRAVPFVIETDETEMIAINYVAKGAGSAAAVAQTSQSNARKAEETAQADTKTRSTDQKETPTQRGPSLTSEEEDQIAGITTRLNSVKMLQERLQLVSRLVGSTPSSYLSDQTVMLSPTSPSPDHLAQLRNVQALLSRLSLLTPAGQNESQALQQAGQAQSNDVSISSILSALGQDIQGLSELGRKFTTIEQAKTSKSKTKGTYGQGTFGNMDDFNARSSRLDMDSGLMM

>CF317_000428-T1 CF317_000428

MALRTRGFSARIASLKQSQKYLGDARDPHFIPKSPIIERQKISEKDEAELRRMCALALADVPHSDDMTDDPFRYLAKHITAKAPRASAPKEEPLAPANEAQEPVVDRPETAAPAAGDDTATPSETSRRETLLSTTTTPMTTPGVTPGETGKRFSEAGRRSSTATPSSLRTEVQGLKANAVYSFLTDPLPTTRPQTAATKSLTHLPSFGKTKSRQSTSAIPPQTEGAVIGRPDFNKSLPSIAKSMDRAEPATERAPKGKPGAITRMLKTVRRQRTQTPPTVQARTTSADALRQMQHLGTTSGSTPAKKQRFNFPSIFHKRNAAHMS

>CF317_000429-T1 CF317_000429

MYHTCASEYRQPNPYYHNDSIPLMGLPLSAEQIDLHMQQLLQWAPTSAHYSNDLNTMTTTYPMACYQPQVPLPSSCTTNAYRYPVAPVPSTYVPSSGSSLQIPPNGPRFQDIPPQSYCRSPNTPVSCYQDDLGAFRDVQLAHSDMRPSMMPDSYEYLSDEQGHVLHQVHYGTSTSSSPHSFSSEDESLGSFNFGSSDQNRATEVDALMQALEEPSTRAPRGEMPQLVRNGKVRKHQCPLPDCQKAFSQPTHLKIHLRSHTGEKPYCCQVPGCGAAFSQLGNLRTHERRHRGEKPRRRSELHSDSSSGASQKRYECKLDGCKGSSDSTGKIFSQLGNLKAHMNKFHKDTLTRLSMHFANQNYEDEYGVSKEEAELREYFRSLYKNCNKGIKGRGKGRRVAVVVGDDKL

>CF317_000430-T1 CF317_000430

MFSNTQHRGTVGQPNLGPGQPAQGLQRLNDIFDSLRAEFESQARGSEQTETQVSQQIQEMELIRNKVYQLEQNQLKLKQDYEAEIRMLRHELELRGGAPVQTHLPGPPQHGGPAQAPPPALGHGQGTLFSGIMANQGPGGPGLAPPPDQQPPPQQHPLAPPPPSGQPQGPAPPTSFPGYQQGPTLNGYGPQAQASTNSPGPSKRNIANRGPGPATPQQHPGQPQPMPYQANLAASPQIPRPTPPGSDMQVGPRHQMPQYSHQIGNVGNQLADLEHEHLPDNLKRVAPDWFAVFNPNVPRTLDVSLVHNLVHESVVCCVRFSADGKYVATGCNRSAQIFDSQHGKKVCELLDDTVVDKDDDLYIRSVCFSPDGKLLATGAEDRRIRVWDIETRKIKCSFDGHEQDIYSLDFSRNGRLIASGSGDKTVRLWDIETRGQIMVLSIEDGVTTVAMSPNGQYVAAGSLDKSVRVWDCSSGYLIERLEGGQGHKDSVYSVAFSPSGRELVSGSLDKTIKMWELTPQRGLLPNTNASSTGKCIRTFEGHKDYVLSVCLTPDGQWVMSGSKDRGVQFWDPNTGNAQMMLQGHKNSVIITD

>CF317_000431-T1 CF317_000431

MLQLSEESKERISKILDITREVVHYGYLPLILYLGKRDGMRQSVMALTWCQGYSRSQPKPALISLISPFAN

>CF317_000432-T1 CF317_000432

MSGAKHWEQDKDSTVYVGNLDERVSDALVWELFLQAGRIVNVHLPKDRVTQTHQGYGFVEFISEEDAEYAARIMSQIRLYGKQIRVNKASADKQRSVEVGAELFVGNLDPMVDERTLYDTFSRFGTLVAPPKIARDESNLSKGYGFISFSNFDASDDAIANMNGQYLMNKDVSVQYAYKKDGKGERHGDQAERMLAAQAKAHGVEPTPATIPTAIAPPAGFPAMGAPQAMPQGMPHMPAGFGQPNGNYQNIPPPAQARPPPPPTTPLAAPPSGLPARPPPQNAGYGGPPQGFMPPGFNVPPAGFQQPPQQYPQPPNFAGPPPAMSQAAGRPPPLPPGFQNFGMRK

>CF317_000433-T1 CF317_000433

MADSGFRPRRGGGGGGFNNRKRRFRDDDDANDGGGRYQRRRYEEPPAIKLRKQVVSIAEAPHRRVDEDIMNSAKTIAENYFDTDLTNTFLDIAVSLTLEQPLKIPFIAAIVLQTNTQKPEFTEEVLKKLGDALQQNINQGCWREVKLLLRFLACLQALFMGDGVFPFLEELFTRAAELQTASNEDALGLELVKIILFTIAYTMASSATECEAQAASLLDQTSIIASGPPHVLENLVDPYPSFEQGAPPSHESVLSLLQKHMEEEKKNNWELPCLPRPWKSSQPAGEEDLLASAQKHPFPTLDLPATLQIGPKQIFPEVYFSVYADQDINTVPAASDSSALLIRDALIDAINVLHINRFIAGKVLIDMDCYFAQDTFVKRATAFDKIRDLAGEKNTWKPEDVIVDATFSQLLQLPTPEHKPVFYHSVLTEACKVAPAAVAPSLGRAIRYLYRNVDRMDLEISQRFLDWFSHHLSNFGFTWKWTEWVDDVDLQDVQPKKAFILDALDKEIRLSFAQRIKGVLPEPYQALIAPEKEKDSPDFKYDDPSTPFAEQAKELMSKIRQKAPDDEVALVLSAIEQEAATSGLSNPKVISTDAYMTSICFIGSKSLSHVLAVIERSRDRLTSLANESPELRKQIVTSVLDFWKHQVGTGVCLIDKLLNYGIVTPHSVVEWALVDNVDRGTLLTRNWCYELITKTTSKVVYRVKQVVTKIRDPRLTEEERTTLRAALDKEMADMKNLFSAIEDAVASVAAAQEDGMMESSDALRAEQEPVLRMWGAKWLRVFRRRSAVEESWVKEELGKPLPELPAEPGPKADDAKMEDVKHTNGAGDGEERREGARGEDVNDDVADGIE

>CF317_000434-T1 CF317_000434

MSLQRDLHGSANTSTTSNRCRPIAIRNADTYLSLVARLPSEQATSGLVDAHFSAENWSFPVLDENHFRNLQRRFQKHVENKTSSDNAIHLPADLTIFPALLFSVLSVTLQFITPDNEQALALDLCTLKERDRTSQWYLDTGQEIIELFRWQKPTTVSIEFHLMKMAWLKNCGRGDASWQSLGTALRQAQEIDLHQIQDDVIQHPSVDVGLTLERLWELEHRKRLWARIFIMDSHMAVALGRPRGIHREDCNTPAPLDCAYPREPSQTVPMSTKHDQEPPNAFSHVMFSIALSHKYHDLMSMRASKLDLKDYNRVRNLHNDIESLLAELPPALRPAFPDITWDCQKSQLSAVRLRLLTTANIFLLALHRPYVSTHVASRHAAIEAALTILQAQQELFTIVPQAQHKLYGYSFYTIDAGVFLASNVLKYSSLDIETTTKALQALQQASMRLCCMKHTSPIAKTGEMVLRQCCSMIEAHVPLPASTPSHNYEEITPQAMDLEVMNFLQGFGTQMPDQANPELMSLLAEPSTAMDLAAAAALPSPPSYHETDMLTFDASITEDFLPSNFTTESMGQWRI

>CF317_000435-T1 CF317_000435

MAIHQVLRVLLTCFIAVLPHATAQDQSPLTPPTDASPQPYRVAIIGAGAAGSSTAFHLTQFANTSSHSLPPFDITIFEATDHIGGRTTTVNALNDPRYPVELGASIFVEINAILFNATRDFNLPANNRIYESTGAEFDLGIWDGESFVFTIANRDEEAGRLKGTLGNWWDVAKLLWKYGLSPIRLRSLQQRTIGKFLKMYDEAFPFRSLSTAAEGVDLLSATGQSGTEMLAAAGVSEKFSREIIQASSRVNYAQNLNDFHGLETMVCMSTEGAMSIDGGNWQIFDQMVKKSGAKVKYNTSVTEIASNTSPHEPSSAVHHFDHNTSQQSTTDFDTIILAAPLNQSAIKLSPPPTNPPKHVDYVPLH

>CF317_000436-T1 CF317_000436

MSELRRRMGRILSDSPSTSRESTPDPNSGEEVKLITTKRLEKLVNRKSSSRRKSTLWFLLGGLLGLVAALYFAQAQDVIKLEGLLDVNLDSLYDVLPAGVMKDVKDLGKAERDAVNYDSFSVGLNLQSQGITAHHPVIIIPGVISTGLESWSTTADARAYFRKRLWGSWTMMRALVLDKAQWKRHVMLDKETGLDPPGVKLRAAQGFDAADFFITGYWIWSKVLENLATIGYDPTNAFTAAFDWRLTYMNLEVRDQYFTRLKSYLEIAKKTSGKKAVLVSHSMGGQVVLYFMKWVEHEDHGGGGPNWVEDYIDSWINVSGSMLGAAKDVAAVLSGEMKDTAQLNAFAVYGLEKFLSKEDRAEIFRAMPGLSSMLPKGGDAVWGNITWAPDDIAANNFSYGPFLSFKPPQNTSMTPSNNLTVVQSIDYLLEHSGDWYSKMIRNSFSHGVAHTKAEVEANEDRPTTWLNPLEARLPLAPSLKIYCFYGIGKPTERAYFYKEMNHASHQPIDAVTNITIDTSFTTGPPPFGTPQVDHGVIFGEGDGTVNLLSTGYMCSKGWRDITRYNPAGVKITTYEMPHEPDRFSPRGGPNTGDHVDILGRSSLNEMILRVAGGRGHEIEDNYQSRIWEIAERVQIFNEEPNV

>CF317_000437-T1 CF317_000437

MSNDEAKPLPAVLVSYSRPPSDVGKLIQTNPPNIVYVQYDISLEPKYLPQMRALIAKDLSEPYSIYVYRYFLYQWPELCFFAVDTSTEDHDLVAVVICKLERHRGGPMRGYIAMLATKDAYRGKGLAKTLVSKAIDLMILKDADEIALETEETNLAAMKLYESLGFLRSKKLHRYYLNGSSAYRLLLYLKPGVASIPLDEFYDPYGYGDLPPSAQDDPRYLQGHEDKDELFGQGII

>CF317_000438-T1 CF317_000438

MPANSRRRNADNASNSESWRAGSVQVQQPELGYTYLAANGRRSKRPTIIRSLEVAPASVIELVRRLYNQYLGEPETREQKQKRNQDEAKVSKYLQGGPGDYLDAVLRVTTTMTDAEEAAMIQRARQHIPHEFAHKNLLHDALNADEETKEYQRLARIGDLVQNLLLSELIYEGTDLSSGFVETRLKQVVGKNVYQAKQMLESSLWGCVFQRQDNSRDLHKAKKLPADVLEAVLGAVYLDTRTGPHWRTECLKVMAAFKLDATAAEGAWRELRAAGLLRQHSLGLPGGSDMWLEGYEAMSDQLGDAQKHLQRMGLAV

>CF317_000439-T1 CF317_000439

MAGRPRVYFDIKIGDTDKGRIVFELYNDVVPKTAENFRALCTGEKGTGKSGKPLSYKNSIFHRVIKSFMIQGGDFTAFNGTGGESIYGEKFDDENFETKHEKPFLLSMANSGPGTNGSQFFVTTVPTPHLDGKHVVFGQVLNGKNIVREIENLKTQSDKPVEDVVVADCGQLEGSDYDSATQKAPDSTGDPYEDFPDDQGSDLKAEDYFKIAAEIKDFGNKAFKAGDVETGIEKYQKGLRYLNEYSTTSSGSDDLKDQVDALRFTLNNNSAMLANKAKRYQEAQKWATAAIESSNKNAKDTDMAKAYFRRAQARVGLKDFEDAVKDYEEASKLAPQDPAIKSELTKTKNAVKESEKKERAAFKKAFSS

>CF317_000440-T1 CF317_000440

MSRPNHGQPRPVGNSMHDGLQLKLSLLSPLDLNSLHRHHRREPSDPSPLTAGTDYSDASRGSRTSMDSYRTDGTVMTPTPFRSRANTTTDLWQPEQQIIAPNYVPCKPPIRVILGTASVGSKKSPLAKITTVDSVSNFTSRFRARGYNDIDTARAYPVGRGGTCEQLLGEKELKLQTWANISTKVSSFMPGSHRTKNINQSIDKSLEALGAEDGVDIMYLHAPDRATPFSETCEAMHEAWLEGKFQRFGLSNYTVDQVEEIVKICEECGFNPPTVYQGQYNPICRGAEERLFDVLREHNIAFYAYSPSACGFFSNKVTRASTKDQDSRWNIASPLGAKYAGDYFHDPLFNAAETVRSQARKYGISGHAAALRWTTWHSNLDASCGDAVIIGASRIEQLEENLDILEQGPLPEALVQIMDKIWDDVKQLEKGPRFSFV

>CF317_000441-T1 CF317_000441

MDRIRRSQGNDAYRSLASVPPKPHRSTSYNHESGLVYNEVEVSGSSSQSNWVYDYRTPQIGRVRPRPHENTGARTLFHMALVKLGLESQNLTVDALQMLPWSIGERIWQQITQKYVAMFLQSSFHALTSFLSHAESFYIWRTFAIAFGTEFADNSNRYMLDIKAPALSLPDYYKGLSSRKREWLTCLRISPKQVTVPDLVSLSAITNLSVLDLSDGRLYMENRESTFDIRIMRSWSELASTGRAFQSLEVFMLGWQEKVDTWIFDLLDSFPRLDILVMTDCRCIHHKNHKEWQDDAWKHGWTFMPSKRGAKHLRTLLDDKSFYAASASNLHYEHMRMAAEVAKRPMNSSISDRPLVECWLGTPKPWRHVIDEFPGTRTVFFQKLPTARVEPNASKRPLNDTTDAKRDPSRSDASHVELGQNTISSKRRTAKHSASSLLAEMS

>CF317_000442-T1 CF317_000442

MAPHRTLATLESDGSTILKEKPSWLRQSKDFPDPGPPPDGGWEAWRSVLGGFIVVFLCWGYINSFGLFQTYYTSQPHINASPSDISWIGSIQIFGLMFIGAFSGSASDTGYFRLTSVAGIFFFVLGVFTASMSRSYWQLVLSHGICTALGNGLIFIPTMSVVSTYWSPNRKSLAIGCMLCGAAAGGMILPVLFNNLLPQIGFGWSLRVFGFIAIALFGCSQALLKKRLPPKDGVKVLEFAVLKDGVFDLFVLGSLVNFLGLYLAFFFVVSYGRDVVGMSFSKSNNLLLVINGTGIPGRLIPMWLADQRQWKGIRPVTVQVPVTLITSVLLFSWIAVDDERSLYIFAAFYGFFANAVQSLFPATLADMTLDPRNTGSQLGWGFTIGSFSCLTGNPIGGLLVQTGGGNYTHAQIYAGCCTMCGCAFYAVVAGLRIRQQQRIDNRS

>CF317_000443-T1 CF317_000443

MAGSRFEDDIEQNSANMASDQKPKDGRKRVLVVGAGAAGMSTAEHLANHPDKFDVTLIDAVDYCGGQAFAVPLNKDRHGASWFNQGVQGGSFIFHHTVTMFARQGYHADPVKLQVSFGKDDTFWTNVFPTQVLEKHQKEIRRMVFMFKFMRWFEIFFALIPLKLLFKLFLFSEDFINIIALPMTALFLGTGNATPVTPAMMLERLTTSPTYGMWYPPDKRSVASNMPPMIVFPNFSDFYETWRKDLVSRGVKVRLSTEMTEVVSRSDKGVVVRMKPRTPVEDGHNPTGGDPDAPQGEETYDELVMCTLADTAKNTLGKVATWRERKVLGGAKFSDDITITHTDSDYMKKHYENFYREDLAVKHLSGENQTQRCEFGKEKFRPMYYIKMYEKNKERLEMCFDTTNYQSQFPEKVPFEDHVFQTIFLNKERDGHLWTDHEIDKSKIIRADWWHQLCHTFSHYLKVVPWIMFLQAKNHTRFAAAWTLVNAHEIAIMSGIAAAVDLGAEYPEDLEHDKFALLCFRLYYLLTYGKWYRRKYKGSTSQKAKAGRSWANGTYGEDYEGPGVTQEEKVTWREDMKLGKSLPNMAEANAPGRSQP

>CF317_000444-T1 CF317_000444

MGCFLVLQKRSDKATKVIPPDFDALRLSAEFIQERIGHPIPLVSADDFRNGPYQFERGLGAGKEGSVDLYTNRISGENVVLKRYGDSPEVSWAPVPSFINQTDDIIPRQWPAEISSGLVVEAICNTEPVRDKFVPLREFFFGTLDGRQKSPAWHLVMPMMSRGSVGKLASDLRSERSSDVRDLDLQFRPAYEGILAVLARIHAKDICHDDIHKGNILVHDDITQWSLGDYGQVRGVAHPYHRTQFWTEGRQWTKCKLNDVRRSLKLYMEFLRNACDKPEQFDTEFFAGRTPWSRLYWEYFQSPTSAQFLIDVSRSHRPESVPVVPGPSGAPPGLVPLRAFTDFSNVRRTAIDLELSTKFMGSKMRAWSNLALFGWDIETVAV

>CF317_000445-T1 CF317_000445

MATNGTEVKLIGKEQVIDDTPKVIKGLKFGVLSNQDIVNQSQVELADRRMYNLDEGRHVAVHGPLDPRMGISDKRGKCESCGQQLANCNGHFGHLKLVLPCFHVGYFKKTISILQSICKSCSCVLLTEQDRRKFIASLKRAGSDTLRVASIVRKATDQAKKTKECPVCGAVNGLVKKAGASSLKIIHDKFKSFNSSTSAKRIAPPEKVAFDASLEAARKANGEVTRNMNKAMDDITPLRAFKLFRKVPDADCALLGLSTGRPESFLWRYIPAPPVAIRPSVGQEGATTEDDITAKLGDIVFANQQLKEALKKGAPVQSVIEHWDYLSLQLAMYINSDVPGLNKNDLAKSIRGFVQRLKGKQGRFRGNLSGKRVDFSGRTVISPDPNLSIDEVAVPILVAKNMTYPEVVNRNNKEKLQQRVRNGTKKWPGANYVKKKKDDITMFLKFGNRDHIAKNLQEGDIVERHLEDGDIVLFNRQPSLHKLSILSHHVKVRPHRTFRLNECVCNPYNADFDGDEMNLHVPQTEEARTEALLLMGVKHNLVTPKNGEPIISAIQDFITAAYLLSSLDVFYDRKTFTTICMGMLDPDTKFELPPPSIIKPEALWTGKQVFSVMIRPNKKTNVLLNLDATCRDHKPYHKDLHRDLQDDSFLCIRNSEVMCGRMDKSTVGSGKKNSVFYILVRDFGVYAATSAMNRLSKLCARWLGNQGFSVGISDVTPPGMLQQVKDDTITKIFGQCIDFEEQSKAGKLKRKPGLDHAQTLEAEQVRALSTVRTDIASVLTSKLSKRNSPMVMAISGSKGSNVNVAQMAALLGQQDIEGKRVQNGFQDRTLPHFPKHEASPPAKGFVENSFFSGLTPYEFIFHGMGGRVGLVDTAVKTAETGYMSRRLMKSLEDLSTQYDRTVRNSASNVVQFRFGDDELDPVDMEASAKPVDFERTYVHVEATTFSTTDKGLSDVDIFSNTEKLLEVQRKGLNRVDFHGNPLPYDTENDALADQFESARMFVKSIQDFVMGKSEKFNEARANKKIWATAGEDTADVATGNKRTRRSSGVAPASKKQKTQGGVKPSNQVVRASPSTTDRFELTSKLSAKTLDSFVKLCLEKYERSKVEPGHAVGAVGAQSIGEPGTQMTLKTFHFAGVAGMSLTAGVPRIKEIINASKTISTPVITCRLERRSDGSIPEELARIVKGRIECLYLEDVTNYIQISHPTGRSSCIYLKLDMDTIVDFGLDITARDIMTAIQKHRRFAKADLNCVLVRNDEIKITSEGAIKSGKRGAAAKAEESAPNDRLLRLQAVRRILPSVHILGHPQAQRAVVMAEDDPKSDEILARRKALKDTKPDMTTLPLARAASPDHMDTDPPIKQEGGVQPQLGEPPIKQEVTAPTEIPKPAAPAKPLQMHKLMISGYGLRKCLSTPGIDPSKTQTNSIIETKEVLGIEAARSKIITEIAEVMKELNIDPRHMALLADVMTYKGEVLGITRFGLAKMRDSVLQLASFEKTADHVFDAGVIGKRDDIEGVSECVIVGKTMGVGTGSVEVIRDLGVSEGDMKIRRCAFNDAWERS

>CF317_000446-T1 CF317_000446

MAVVDKSKTDTHKIALKGSAKTVAEFFEYSVNTILFQRGVYPAEDFTPVKKYGLNMLVSSDDQVKAYIKKIISQLNKWMLKSKISKFVVVVTSKETGEHVERWQFDVQVFGKGASKKSPSRTTDTENADPNPPVADSEPEKTESQIQEEIQAIFRQITASVTFLPMLDGNCTFNVLVYADADSEVPLEWGDSDAKEIKNGEKVQLRSFSTTNHKVDTLVSYRLLD

>CF317_000447-T1 CF317_000447

MRQWLQLFSRRRAVQQALAVAPRLAERPSITRAFHTSNHFRAQAHSHHSPSDIERRIAQIPIERYRNFCIVAHVDHGKSTLSDRLLELTGTIQPGGNKQVLDKLDVERERGITVKAQTCSMIYNHKDHDYLLHLVDTPGHVDFRAEVSRSYVSSGGALLLVDASQGVQAQTVANFYLAFSQGLELIPVLNKVDLPHADTERSLEQMKDSFELEPESAILVSAKTGLNVEAILPRVIENIPAPTGDPSKPLKLFLIDSWYSSFKGVVLLVRIFEGQIKAGDQVVSFATGMKYTVGEVGIQYPDQVPQSVLRAGQVGYVYFNPSMRKTKEAKLGDTYTKVGFERQVQPLPGFEEPKPMVFVAAFPVDQGDFEHLEDSINQLLLNDRSVTIQKEASHALGAGFRLGFLGTLHCSVFEDRLRQEHGASIIITPPTVPFQIKYRDGREVIITNPAEFPEGDHTKRDVAELREPYVTATLTFPEEYLGRVIEICEANRGEQKSLEYFTATQVILKYEMPLAQLVDDFFGKLKSSTKGYATLDYEEADWRESSIVKLQLLVNKEPVDAVSRVLHQSQVRHVGKRWVEKFKEHVDRQMFEVVIQAAVGRQVIARESIKPFRKDVLAKLHASDITRRRKLLEKQKEGRKKLKAVGNVVIDHSAFQAFLAK

>CF317_000448-T1 CF317_000448

MPRRKQTARQGPESDNGAPDEEDLQEINGRLTEEEKQRRAELEKLPMQAELKNLEKRWTKKGRAYITEPKDDEDVPDDNNNWYDKFALCITRLYDQQNEFVQQTSLQVNSPSLKELLKDVIDAYPGISFHTTNISILSPYHCLYHYRVEFSDKLEKTSPDSELGRHLPILLKFLEEEFVDTLTETNNLNPQGLCSYDTLWTLFRPGIKIFSRLGGQNRAFKLNSYQYGVDRCGEYLALKVDYIDYDGDDFGTRQTSLRIPKFSGTAPVTALNIFPLARHPNALEISKQLIARGRKWEEINAAGQSFMDYKGIAVRSGCPPTKHNIDCRVMIDVATSHRIEADDAFSVNPLPLTEAQKAKATLLQNDPLVISMTNGNKDLLLVDREPLTDEQRLMATPMMLGFSFNEKLFLEFFVDALTPIEWNTSCFEQLVLPTSQKELVQALVAEHTQRQAHPSGKGFDDIVKNKGLGLILVLHGPPGVGKTLTAECVAEFSKRPLYIVSSGDLGTSAATLDDKLSRILDLASTWKAVLLIDEADVFLERRSLNDMERNSLVSIFLRTLEYYSGILFMTTNRVRTFDDAFKSRIHVPLKYDDLPHSSRVTIWKNFLDKVRQTDPAAEGGIEVDVDASGYQKLADSPLNGRQIKNVVRTAKSLAGYRGGKLDVEALLRVVEIQMAFEEELGKAKSNGMDIDDIES

>CF317_000449-T1 CF317_000449

MKIHADFDLPWCKALLHAPDTTITETPTHPAAGTPARDGRVSNSMFVQTLYTPAAIRAQLNFTRPAAEPDALPAHTESCYLLSVGAGLDGLTGRAHGGFSALVLDQITGTLASRVSASEAPATATMTVDYRAPVDTPGVLLCRAWAVERSGRKTWVRGRLEDGRGRVLAEARALFVSPRAGGRL

>CF317_000450-T1 CF317_000450

MSVQRADQILNKTDENTNPEAPNYNDGGMFHNDYQWYTFGGFSDKNLTSEMNSVNKGVLFENNPIQTPGSGVGVVPYNHGEPIGLGVSENIAAGAYASSANPEQRLGWYFGGVAHPDYSALSWTYFNDNIVASGTFYRIDMSDSEHAHFTSLKWPEAQAPRAEGGLVWLPFGEEGILVALGGVITPPDLYVVAPKALPNNNTFMTDLAIYDIHGDKWYSQSTLESSPGPPQLAKFCTAVVPTEDGCSQEIFVYGGYDGTGESSTPENDDVWVLSVPAFEWTLVKEAEKGSTHGRSGNICFAPNPTTMITVGGSLWAGGSLRSNTLVDIFNLSSLEWDGNYDASSSASLSAPPAVVQQLNWPSPRGPGGITVSNLADPDLNDLFNTPYPTKIKSNYPYATETPKIDTEDGHHNTNKWKIPVIATLCSVLPALMIAAIAICCFRRYRKNKQGDERTQQSRRNVFSWLGKPSHVDPEPEKSNASDNTAVESNPDYFNQPGYKTAKEVVYEAPSNVTSPGWNEYGHGSPGLTGVTATPQHERHEIMDQPQRESMSIRNHPYYPRSIAGNHIRSVRSESLSQPSELVSSPRTTAVQTSPSELPQDKSNENLPQAPNDMKPDQNEGGEAFPSTGMAPGSAIPRKPVGERRVSSPDPWSASPRPGHKRNQSSISSDTPILPSPEPKEDWRRSRQIEALPDVPTSAHRQTTTNESGRVSAYRETFDEATDRQ

>CF317_000451-T1 CF317_000451

MKWSWLLSLAVGLKSAAAGNIPNSAPAGTEWVDWSTTTVTSTIHDTTTSYVTVMGPTSISIDYQTSTETSLVSVTGPTSVSIDYQTSTETSLVSVTGPTTTVTQVHVITQNLTTTTTSTTTSIVSVPGSCSSSSLSGLVLCPTRIINPTYTPATPLPSNYLWGCPPGKLCHPKREGCNFERDLPADTYVCAPEECLPTPELPSYEAFVAANLPQSNDSCAWLTPIDGYFNLNPEDFDLDYSIFNVYGRTTCTSSSAAPSSNKWGAWSGPAPTSATGHGNVAPPQVTKRAELAEIDLMKRQGRPIIAVKSCYPDCDYAAAVWASIGPNPVLCNAGGPWSAAFDRIINCNARNAGVGPSRVASKLDEARAFCVGL

>CF317_000452-T1 CF317_000452

MSIRQLESGLKHLNVNDENEAPQAPTGLKSKQVLTHNVSRSNLVKYALQASSESRQNEGNRVATITKTSQIPTLPPSPVRRQLSGSSEESNVISERTLASQSQASTNSSFSDMPPHPARPEKDLSLKDFEIGKPLGKGKFGRVYLAREKSTGFICALKCLHKSELVQGKVEKQVRREVEIQSNLRHPNVLRLFGYFHDKKRIFLVLEYAGHGELYKHLRKAGKFPEWKAAQYVAQMAAALKYLHKKHIMHRDIKPENILVGIHGEIKISDFGWSVHAPNNRRQTMCGTLDYLPPEMIKPGGKDNFYDEKIDLWSLGVLMYEFLVGEAPFEDTPVMTQRRIARGEMTVPSFVSPEARDLIKKLLVLDPSKRLPLKDVEQHPWILKHCLAKAGERGTERRS

>CF317_000453-T1 CF317_000453

MGDAGGSERGIRIAIDRGGTFTDCVGNPGTGKMEDDVVIKLLSEDPQNYEDAPLEGIRRLMEKFTGTEIKRGQALDTSKIESIRMGTTVATNALLERKGEKMAMVVTKGFKDCLEIGNQSRPKIFDLAIRRPDVLYQKVVEVDERVTLEDYAEDPYRNQTKADAKHEESDKNELVQGLSGEAVRILQRPDESNIKEDLQKLYDEGIRSISVCLMHGYTFPAHEALVGKVAKEIGFEHVSLSHELMPMIKLIPRATSACADAYLTPAIRKYISGFEAGFEGGLGTEATKKTSGEKAARCEFMQSDGGLVDVDQFSGLKAILSGPAGGVVGYALTSYDAETKTPIIGFDMGGTSTDVSRYGAGRYEHVFETTTAGVTIQSPQLDINTVAAGGGSRLFFRNGLFVVGPESAGAHPGPACYRKGGPLAITDANLFLGRLLPDFFPKIFGKNEDEGLDPEASKKLFEELTQDINSKTGKNMSADEVAFGFIKIANETMTRPIRSLTEAKGFDTSKHKLATFGGAGGQHAVAIAESLGVRQVLVHRYSSVLSAYGMALADVVDESQVPESQVWNEDDSSVQKALKGKIDELKERSTKRLADQGFSDDSVEFEEYLNMRYRGTESALMIVKPSQDESKEDFGGDDFAFGKAFVKQHEQEFGFTLPDRDIIIDDVRVRGIGKSFRGMSKTADQQLKELKSQLKDIDTSKAYSTADVYFEGGRQKTQIYKLEDVDVGNRIPGPAIIADGTQTIVIPPGCTAIVIETHVVINIGEEQASSNNQISTSKVDPILLSIFAHRFMAIAEQMGRALQKTSVSTNVKERLDYSCALFDATGGLVANAPHLPVHLGSMSTCVRTQAQIWKSKLKPGDVIVSNHPEYGGTHLPDITVITPAFADTNDPNSEILFYVASRAHHADIGGILPGSMPPHSRELYQEGAAIKSELLVSQGTFNEKRITEILYEEPAQYADCSGTRCLADNLNDLKAQIAANKKGINLISALIEDYGREVVTFYMHQIQDNAEHSVRELLKDVHKRFAGQDLHAVDFMDDGSPIELKITIDGDKGEAVFDFEGTGPEVYGNINAPEAVTYSAIIYCLRCLIDQDIPLNQGCLKPVKVKIPKGSFLSPSEKAAVVGGNVLTSQRVTDVVFRCFEACAASQGDCNNLTFGFGGNVAGQDENGKKKEQVKGFGYYETIAGGSGAGKDWEGTSGVHTHMTNTRITDAEVFERRYPVILREFGLRAGSGGKGQHRGGDGVIRDIEFRIPVQVSILSERRVYRPYGLHGGEDAQCGKNIWVRKVKKVHSQGAGGEHEGQRREGKNGEKEEETEERWISMGAKNTANMQPGERIIVMTPGGGGYGKAGQESKIDLKKDYEQNWKKGSLANRLAEWESSS

>CF317_000454-T1 CF317_000454

MAKRKSGHSANGDFVVADDASLNGSAAPPSKRSKKSAGETTSSHFSTPSATTPAKRDSEDNLYWEISKARRVVLNDFKGKKMISIREYYEKDGNWLPGKKGITMTIEQYGALMGVLPAVDRELRKGGVDDLPRPDYSASEDADVVEDEPEDETEESEEEVNQGGGKANHEATSDEEE

>CF317_000455-T1 CF317_000455

MSLSTEMALVGNTFSSVAPAVGALLISATQSILVLRPTTNVRQTIYGFILVLTVGLWIRHDPRLAAEMALTSHSHFDRGHPIENLIVSNRHRFDALLSRQSQTLEQAVTEYQRRYQRRPPLGFDKWFQLAQDNQFVLIDEFDTLMHSLEPFHGIHPDVLQQRLEKVLEKASGQMVTYNITDGKLTMSENLKDICERLTNTKWLDIIPYNMTLAINGFDEAMVSAPWEEVRKAVDDSKTKDKFAEHQVQANLSPLLRIGGQSAWSATSQACSVYSPSRQIECPAASRKLTDPLTFVQNATFSKDVCQNCELLQQEGFLMRPDTLNVATQLTPVWSASKPSHFHDILYPSSYYIQVRDDYNAEQDIPWEQKDNAFYWVGSATGGQVTKSNWKMMQRQRLVLKTQKGSNEAIQLFEETKNNSGIWQPRYTTMAEISELFATRISAVVQCSDAACQLEKEAFGIGVGHKDEQGAAYAHKFVLDVDGNTFSGRFYRLLQSRSMVIKQTIFKEWHDDRLIPWVHYAPVSTSFDELPEIARFFATTVQGQALAKRMAEESTAWHDKALRDVDIRLVWLRMLLEYGRLMNPGLEVDS

>CF317_000456-T1 CF317_000456

MLCRSFQFRACAQRFWSRPHTVGLQTHQSLQSRHIFRTFRWNAFQRQRQENEQAQVKVPDDAPVKAEITQISRPESAEKPSPVGRNAKAAQDANLQSKTELTKKEQRQIDFAIMKEMVRYLWPKGEIGTKVRVASALSLLVGAKILNVNVPFYFKSIVDSMNIDFMSVGGTAWTVAGSMIVAYGVTRFGATIFTELRNAVFASVAQKAIRNVARNVFEHLLRLDLNFHLSRQTGGLTRALDRGTKGISFLLTSMLFHIFPTVLEISLVCGILTYNYGASFAAITAGTMVAYSAFTITTTSWRTKFRRQANAADNQGATVAVDTLINYEAVKYFNNEKYEVARYDQALKKYEDASIKVTTSLAFLNSGQNIIFSSALAAMMYMAANGVATGALTVGDLVMVNQLVFQLSVPLNFLGSVYRELRQSLLDMETLFNLQKVNVTVTEVANPRPLALTRGGEIRFENVQFGYNSNRPILKNLTMTIPAGKKVAIVGPSGCGKSTILRLLFRSYDVQGGKIFIDGQDITEVSLDSLRRSIGVVPQDTPLFNATIEHNIRYGRIDASSEDVKKAAQRARIHDHIEKLPEGYNTKVGERGMMISGGEKQRLALSRMLLKDPPLLFFDEATSALDTYTEQNVMQNINSILKEKGRTSLFVAHRLRTIFDSDIIFVLRDGVVAEQGTHSELLEQKGLYAELWSAQEVSLGQDIDLERNLEEERRT

>CF317_000457-T1 CF317_000457

METYNGHVRTPNDAIILFEACRLGLLPRVQRRLSEKERQSIRSGSVFVWDEREAGMRRWTDGKSWSASRVSGSFLTYREMEGKRGGNSFSTPANNQQQNNGDRTPDSGPTGMDDGVDAEEGPDGYRYKPDGLMKQSFSLTTSQGQHLHLISYFSRSTPTAMGLRQPSNDHNLQTIQIPKGMYPESTVADASNTPAVTRSPMPGAPNYVMTAGGPFGRAPPPPPGYPPGFMAHPGYMYPISPMHTPPGPYGYPYMHPMHYYHPAAFAPPPPPPPPPHLPVNSAPPSSYDQRPLRGSMQAPKSPGRTSTMTNNENIPFGQDKPQNPPTNTSTYPITPGSDRPPPVQIDPRLTDSAAQATTPTTGAPPQPYSQSHPQSYSQSYPQSHLQPYSQSQPQHEMQRQNSQQYHNMSSGANAPSITSLVNAAAQASPLPSKRTTESEKERQGSVSPGGTRYGAPPQDIPSEKLAFREDKMALSKLDRVFARV

>CF317_000458-T1 CF317_000458

MIEFGSSSSSWSFEPAITLLKATANGDHTLEVTEIAQPEFSPLPTPAPQYAARGLGDFTQIWKHLGVDYNSPAAHTVDDAYDEAKLGSSIGSDASQLVKAVKWRDEQDGADLEDNVEPEQVTASNLRTRKRAARRARARERELQLAQATFDPLSDTASDGESGEELESVRRSPDRRSLIADLVGRPRPRPTENISPPTSPSPPKERAVLRTPTKKEWPTANPFLWSTPTLQSSTSKNTILPTDGLTPYLRKVSLISQLSLRFPDEQKYLKNEGLRSPAFSPLNTSPIGIHVFIDFSNIAIGFHDCLKVARGIPVSNRVRRVPLNFHNLSLVLERGRPAAKRVLCGSERTSTVDQAEACGYEANVLDRVHKVKTPTPRKKYYPGTNGGGYNSASGISYGETSGSETPAATYKWSEQAVDEILHLKMSHSILDAEKPSTIVLATGDAAEAEYSDGFLRMVERALRVGWSVELISFRQNTSSLYKRKEFRQKWGPMFKWIQLDDFVESLIDEGS

>CF317_000459-T1 CF317_000459

MALGSMNQTTNSTSDADPDRNYNLFDKPNYASLEVHSSSILAHIAFEVLAWFFVLPIGVMFSISRSKLALPVQLAFLVLNGLGVVCGTVYNIGTPDLYEGNAHHKIGWIATWVFTAHVVMSLLFLYSGRNKQETPTSSERAAFLPPSLHSMNRPYHKPSWSGDSGHGTEPPSPTSTSRTRSLDRDYNFEKPEQEEPEDMEDIPIEPVRRRPWWFKNTKIDKYLSRCLPQIASQQAIKIAELAYEMIDRTILILGFIALTSGIVTYAGIFRALNVFNGLAHFVKGGIFFWYGLLTLGRWLGSFADLGWAWNLKPTRSEVGWKARVPTAEFVESFVIFLYGCVDVFMEHLAAWGKPWTAEDFEHVSIALLFFGGGLAGMLIESKRLRTWLNTNVDLMPSRTETDYEEAHAVRQHPRSYSTSLNVMPALVILLLGLMMSSHHQASMVSQMVHKQWGTLLAGFSLCRACTYLLNYLAPPKTVYPSRPPTELAASFCLISGGLIFMLSTKDVIHYMEVYNLMPMFILTVALGFSAFMMAYTMFVVGLKGWAIRRGQKVMAQRPF

>CF317_000460-T1 CF317_000460

MYRLNTYPLVHRGLIHMLMDTICLIPLLERFESEWGTLNCLALWLGPLSTLPAGVYIVIEKFVLRGNTAVLGSSVWVFLLLGIEAIKTFRANPYLEIGSAKIPTWVTPLALIVVTSVLIPNTSFLGHLCAVTVGYLFGLGYLKFLAPPEKISRWIEAKLNLLGRLPHYVSVDQKTYGRYGVLPQSEGPSPSSHSVPIGMNWLGGGGQRLGS

>CF317_000461-T1 CF317_000461

MGLLRSSLANIRTSPRLLKVPPYISLLCFLGGVIWILLLPLDEYSRRTYISENALLPGQVHTYFEGTEQNIFRAYRHEVADIVDIHEQINQDGSKGYVRATEQHRNQRIRDLFGNSGIKSAVQSYKYTVGGQDFQGENVYGIVHAPRGDGTESIVIVAPVENFEKQLNTNGVTLLLTLARYFSRWSLWSKDIILLVTPDTSTGPQAWIDAYLSQHDPEKVASLSLKSGAIQGVICIDFPFRHSFHALHISYDGINGQLPNLDLINTATQISAGQLGIPVLMQSQHRYVKPEAQHHYLNRLKVLGHGMRNQAVGHSTGAHSVFMPYHIDAITLTAVGHGQQDEMAFGRVVESLTRSLNNLLEKLHQSFFFYLLMQNIRFVSIGTYLPSAMAVGAGFSVMAIYLWIKSGYEQREEEDQQHTEATEHDSGTRDRTEAALADPSITNQQLFNTESADGIDLSRRKVWKSIDRPLVLPLTLLAGLYLTSLIPLAILVTAQKGALLTRTGSLCSVLIMMPIGLAFVLNSNPKLLDGTFFDGAPLEKQENGAPREAGLVYSDLSPPSDPKTRTWQFHTILKGLSLMVLGLELTVLATLNFSLSMFLGLLCTPLCFAGFYKASSLRATASLIAVVLFNPFVMANAAVMATKLAGYYDGSMAMKDVLRVWLELVSFGWQVNGAYGVPVGVFCVWLPAWTVATLGTVSSFVVDEEGKTEKKVGKVKEIAKDNLTQVKQAAQRRSSPSPARKRKK

>CF317_000462-T1 CF317_000462

MTWRYLRQLCLQRGQIALSLRHEQGATRQRLGRLSSLPAVRYTSTSSNNSSSLDLPIPEHSDTPSSAGLTQPLSVSSTFSKNANLVEQPPPPGSKVKIAGLVRSIRKQKRVAFAHISDGSTFQPIQAILSPDNAKDLHNGAYVELEGLWKESPGTAQSHEMDVEKVLRVGESDVESSPIQKKSMTVDHLRMYPHLRLRLPLYSLLTRVRSQVISSVHEFYAGTNTHTDEAVYVQPPIITSSDCEGAGEVFTVSPKLHVTPQSGKEDKEQKHYFREQKYLTVSSQLHLEAFAAELGNVWTITSAFRAEESDTGRHLAELSLLEFEGRGITDLQELTNGVQRLIQHIVRRLLNHRASEELVSYYESDKRHRLEDYVDPNLRARWKALSEPNWLHITYADAMEALEAAAHRQPDTFKHAPTLDDGLSLEHERWIVQNLGRDRPVFVTHYPKKQKPFYMLPGPGGANETETSACFDLLLPYGICEVVGGSLREHRLENLLQNMREKGLLSNHAGSSEADTDYPNLRDGESLGSMSWYADLRRYGSSPHGGWGLGVERLLMYLTGVTNVRDIVTFPRTYKNCIA

>CF317_000463-T1 CF317_000463

MPKHAFAINSKNVYCMGCRDLVYDPSLVFTSRKRKSAQLNEEDEAYVNANTIPKPCGREGVRGLFNLGETCYMNATLQMMVHNNLLGQYFLGMGHPLHTCPISKEPEKKNDSDDSEDELGDDRDQKTCVACAMTEVFSDANTVDQPAPAHAVNLLFASWKHIPHMSGKQQQDAHEWFTIIIDKLHESAVNPTQNPNYKPNHRKHCQCFFHKVFYGRFSSEVTCDVCHKATTMEAEYSSIDLDFQKQKKRKKKLLKEAAVAANPSAAANTTNKKSQAALQTAAPIPSLIECLNAYTAPEALAQESQQIECQSCKKKTTAHKRTCVKKLPAILTMHVKRFGIKKPPGVTNGSNGALSSFIGIPEKYEGKLDFPLVLDMQPYTVDAEEGWYGDVPKHTYDLDCVVVHQGEHAQTGHYYAFCRVPMGEGKEMRWFRFDDEIVSATTVEEVLRQQAYLLFYGLRDLPRLCGPDTNGANGDVKMENGD

>CF317_000464-T1 CF317_000464

MPHAIADMEDATPVSTATPTTSQADDDIELPDAPQPSTSADTDKVALEDIFNDDVSDSEFLTSTADVPSSPPLQNGHASSSPTSSAPAVTAPTESYSDPTIMLQYYARLFPFRPLFLWLNASPSPSPRFANREFAFTLSNDAYLRYQSYPSHDLLRKDILRLNPSRFEIGPEYSTNPRDRKTLRKASTFRPLTKEMVFDIDLTDYDDIRTCCVKANICAKCWKFATMTIKVLDAGLREDFGFEHILWVYSGRRGVHAWISDKEARELDDDKRKALVGYFEVLKGGVQSGKRVNIKRPLHPHIRRSLDILQPYFFEILAEQEPFLRKEGEERLLQLLPDKDLNDALRKKWASSPERPSRKKWDDIDELAKTGVSKSLDTKALAAAKQDIRLEYTYPRVDVEVGKKKIHLLKSPFVVHPGTGRVCVPITCGGDMKRAEQFDPLNVPKVTELLREIDAFKTEDMGSDEDDGTIPDKTKTMKDWEKTSLRPYYNMFNEYVAKLVKSENVSLKREREEDGHTDEQSQETGQVKSGTTAQQDTAQHKLEPNQPLRDPTPNERPTTPEQSRPEQSRAEQSRAEQTRADQSRRNHTHPLPLKKAAPPPPQQHQSMSTSHPPRPHHRSHTPPPRPTTPLRPRSRTSFRASTSTSTPHTAQPLDALEPQFGELADSLADLESNLVHLQLMHESLARFSENFAAFLYGLEMNAFVVDWPEQPLAESVARWKAGQRAAGGAEGDTTVGVGASANAARTPTKRRSYSHSHGQSGSMGNDIDSTMLSETTFASMKATPARRRTLAPEERKTGGTVSRGRGGSTIGRGGNAEVQEVPGGAACRPGVGVEAQNEQEESTKAIGLAIIGLSADHGDVTADACELLLYGRIVKDA

>CF317_000465-T1 CF317_000465

MSSEGGQIVLYDLPSKPPCKCWSLNPWKTRFLLNYKGLDYRTEWTEYPDIKPKLEKHIQPNNQGFAYTIPAIMFPDGTYMQDSRPIADEIEKRHPSPSAHLDSPAIAKLEQIMPSAFPSLMGVFVPKVPKRLLNEASHPYWYETREARVGMKLDQLEREKGGQEAWDSFKPALDQVTAMLKEDPSGPYFMGKQVSYADFIWGGFLIFMQRIGTDLYEKTLETAGADAEVHNKLLLGLDQWAERSDR

>CF317_000466-T1 CF317_000466

MVLQYGNMAGGARPELKFDDEGGFIKSFRALPPKPDPSTVRVFERNDFYSAHGEDAEFIARTIYKTTAVIRNLGRGDNQLPSVTMTVTVYRNFLRDALFKQGRRIEIWQTSGKGNFKMIKQASPGNLQGVEEELGSGMDSQGDGAPIMLAVKISAKAGEARSVGVCFADASVRELGVSEFLDSDIYSNFESLLIQLGVKECLMVKDVKKEKEGAAKDPELAKIRQIIDNCGIAISERPMADFGTKDIEDDLGRLLKGESTTGTLQAAELKLAMGSANALVKYLGLMHDPSNFGQFQLYQHDLSQYMKLDAAALRALNLMPGPRDGSRTMSIFGLLNNCKTPIGSRLLAQWLKQPLMSLPEIEKRQQLVEAFVNDTELRQSLQEEHLRSIPDLYRLAKKFQRKMANLEDVVRAYQVVIRLPGFIEAFESVMDESYKDALDEAYTNTFKDFSGKLGKLAEMVETTVDLDALDHHEFIIKPEFDEGLRMIRTALDKNNHDMQVEHRKVGKDLNQDTEKKLFLENHRVHGYCFRLTRNEASAIRNKAKYQEISTQKNGVYFTTRELADMRREYDQLSANYNSKQSGLVNEVVTVAASYVPVLEALAAIISHLDVIVSFAHTSVHAPTSYVRPKMHARGTGDTVLTEARHPCMECQDDITFITNNVELRRDSSRFLIITGPNMGGKSTYIRTIGCIALMAQIGCFVPCTTAELTIFDCILARVGASDSQLKGVSTFMAEMLETANILKSATRDSLIIIDELGRGTSTYDGFGLAWAISEEIVSRIGAFGCFATHFHELTSLAEKFPAAVKNLHVVAFVGDEQKTVDGEDGSIEQRKKKQEVTLLYRVEPGVSDQSFGIHVAELVRFPQKVVNMARRKAEELEDFSHSGGGGDGDGDGVKMEGVERKQDYTKEEVDEGSRLLKAVLKEWKARVEERGKEMSNDEKVKLMREMVKGNEELMKNRFFVDSMQAL

>CF317_000467-T1 CF317_000467

MATTDVLLSPEKARRPPQSLRSAGVKSDNPPSSYHLPQNLLPRSESLLTQFEEFSSIGDVFATPTRGLSKSQTAPVLLSPSKTITSHRLFRPLDPPLPRSNTYGDVAFTSRRVPPQTPVGQASLHNMMAHNRFEEQRANAISPYPTRAQSATDGHGEIGQRPWSLPGRDSASSSPKGSEETEVDPRFVFLPQPLTYWLGRYTTLVDQLRTKVLIPQHKLTNNGRGGVNATLPKHKDSVEFERADRDTKKEAMQTLRSFCQTGSAIGSLQGFEMYMSQNDSSFSKQLSTARHEHRPRQSSGQYPPVNPAKPKARKVSDSLPRMPPNIFQKGSNPNKRGTTFTKSFTLASFRSTDSFSSPGSNHPSKERLGTDQHARELSYHSNNPETGFRGAQERSARPDFTNKSSHFEDVLQRAPKGPRMPLSEKVASIKLNSEMPQHIDSVTDNHKVSAVSVGFPQPLDAEVTTKPSSDMDAEDAPTAHSRRFRKSDSMKKLFDASIKGFRNMGQRVSNTGGHDGTHES

>CF317_000469-T1 CF317_000469

MASSSHLKRKSEFSDDQYLNSPWITSKGGDAIFSPTKKRKLRLKTLLSLPKLSFAHRDQYIGIRGDNDTKSSESTLSTAPTHPETLVVSRQQAPVLAVYLNTTFQPFILVKPSFAAGGFARCSIWLGLDDRKLYVRKLQGCSKGCPRDITHYVEHSGVPKLVAFIQHGMNPIKPELVKLKYGAVDKAVHFRDKSLSRLSYWSTMTEYINGPTLLAVFENLAGTGYVFPEPAIWQCGKILLEMIRKMRFPDPFAGDKHIQPMSHNDIYPRNILLVNDDKDEGATTNFHLIDFGMATFHSECHYRNGDLLQVIDILCGMMLSQNLGSRAKAWVRCAKDQPGWPYSNELGMRMAEMEHVNSNSIPYGFPPKLWFDKWITSCNHMAMRTSARCKKAGKSSKIPLQKPLTNERPLGFRGDNAIGELKHFMTKQTGCSPYQQAYLEDETLKVIEVDKLFYWNKPETELPLRPGAACMKKQ

>CF317_000470-T1 CF317_000470

MSGGAPLPQPTTESILSIADLKEAADKKLPRSAREFFNSGSTDQRTLQANETALARYHLRSRVLVNVKNLDPSTTCLGRRIKFPLCCAPAGIQGMAHPDGEVATSRAVRSMGVNQAISSFASYPVNEIVSAGGGGDDAPSYAIQMYPMSNRGLQERIIQRAEAAGCKAIFLTGDSPVLGVRYNEWKNDFRAPEGIGFPILERDTEVIRETTHDDGFAGLNDDGASWERDIKWLRERTKMRIFVKGVMCGEDVANAVRWGVDGVVVSNHGGRQLDGVPGTLDVLEECVEAAKGSSVEVHVDGGFRRGSDTNIMG

>CF317_000471-T1 CF317_000471

MAHDREPEVRSNVSRHALPTRRVRQLCGSTGPNMWIHHDGSGCIVRVRPGHDRSQCHVELEGSERAREITLKHLKRFEADVVPQPELEWMECVRTFSPYSDIRSLPHPPRWTLRTFKLYINALTSDHDYRSAMRYVHQGTEHRHQVVGDLLEQIFNDQSITRFASTRALQTALSFCRQHPELSNTAKNVWHSALSFGLQPDISCFNTDLSRCLVVRDFDRYRNLLVMMRAKNVQPDSTTFSLILRYAKNPNLRRRILHVVEQANLQHQCAHPSITSATIKQEVPHYLRRPDGLALLDERMTKTFGENWLTTRNVERLLRACRIRRPNSAWASDVLAIVQKAQSSNVSLDVHCMTELFRIAKKAGNLEDALEILKSRPMQQVQGLNQELLESFFLLAWRKQHFNLCRLIWFHAATQGRITRKMQTMVQRSLQSNICAGDRPDDRAWLLLAGKVIVNASLNPEQARTMFPHLAQHDIKSSPVEWLLEWTADSGARHEQMQLCQLLLEQDLHAWRFNEPLQHHDFMLLLDKAIALDNEWITTDKVRMSSPVDLLCSSLGLPRRQRAEPITIPSTNTYPLERRHDFVVNDHKLSYDTFVPLDLKDDVDIVDHSAAFGNQPAECSPSAVDNTPKSDLQLNQLVATDNHHRLEKAREDADEPQRFDPLVVADEEASKVFHHAVYS

>CF317_000472-T1 CF317_000472

MAKSTIGAVLPLIITLILLVVVAVVGFIAYQIACDVADKTSKKMEKKNVSFSKDGMKVGVKEVSAEQVGDSTQNVLMKAWNASEWPAYQSKLGWGASPATSPTTGADKRKPFARSSSGK

>CF317_000473-T1 CF317_000473

MAEVPTKPLLSFGDPAHHRGDPPCSLCLHVHPEKELLFCDQCKLKVHSICWVFLRKVFSDLDEWPCAACHLPLDKAIELALRGSVPSADEVTADMLTRRVFFARRAMNQARWAVAIGTPKNEAIEIALRAMYQDIPLEHAEQQQLQPSQALMPEQKDQAMVPFADITPSETKTIRTRMRPSQTDQKHEDHRGRREGTQQVYSYGSSAQPQPAQLPLSDSTTHYHLPADNVS

>CF317_000474-T1 CF317_000474

MKCYCPSLRTVFLAISTVLGLSLVLLVIHQVILPILGWLIPSLVPGLYDLAVYGPYPTHEFVTSDLHPPSPSIIKWDDSCDYGHVLVAPFGNSVPDAGPMILDSKGNLVWMSTAFGTVMNFKIQNYLNEPHLTFWAGNKVGGIGQGYYLVLNSSYDVVHKVLAKSPPAHPDDIRSGDIHEFMLTDSGTALLTVYNTTTHDLSAMGRPTEGWMADSLFQEVDVATNELLFEWRASDHLDPATSSKYINPFGGYKESNPYDFFHINSVQKDKNGDYLVSSRHLHAVMTVSGKTGEIVWVLGGQFNVFDDLSGGKATSFSWQHTARWVDEEKGILSLFDNGSAGPIMTDVYESKGLLIQVDLEKKTARLVQDYRSRDGILSASQGSVQVLDSDDMFEQEKHVFVGWGSSGAYSEHSMSGDLLCETHISASMTYWWERVKSYRATKTFKWIGRPQQPPAAVMSSDKIYVSWNGATEVAAWQLEGRKEGVVDSEWQTIDIKEKEGFEDSFTLPDETFAAYRVAALDNSGDLLRHSDVVDYADTKTTSVLLWLIGIAAAIGAIAGVWLFARRVPRAQRWSWTVWDRHRYRKVPGIEMT

>CF317_000475-T1 CF317_000475

MSIKAIIYSKFDPQEGPKIVQQVPDGSISTSATPEPDAILGFSTVSRFLIPRQSLCGNLVCLTPPSPTSEIPPSIIVSYPVCIKGEHYPRNEFIFNFALVLSPPDTVDVPSYKSVVTKLAHLMRGLEEQTRFLSDDTSPPGAGKIYSLCENLIEDLNNYAECMIPIDEVNTLNIKLFPTLPNPAPVRAWHVPLFTVKIESLMDENWDLTMQRVIPFIDGVNSVKKIALLADADLSLTRKCVRHLLYYGCILLLDIFSFNAIYAPSAEFTSMVATDLEMQVECARYVNTAFAPARPENVVGDADISSSFKSGSVAATAATAADDDFWPLTGRGDVVDGVGIVQLFACLRQGLTVREWYVQNSNMLANIDIRRFITFGIIKGFLYRIHRYAIQTTNLKDENTHAKAKPSKQDLQSGVSGAENIRRKRRHDTLPLSHFLDGTHSFDEICTYFGIPEKDLLQRLSHPQLGEVVVFCR

>CF317_000477-T1 CF317_000477

MSNTSPPVEQQRFARTQTPESPRPVHVPEPANIPVLLNQMDQASNDLATYNIPTDQNFAHYGDSLENPAYKTAASKDENDPFRDFLRNADSVVANPPEPTVPESTNPAAQAQLHGQVEPNHAIAHGTDTHLANDGQPLDANANDVPGSKFENGEETGAQEISLPSNPETRMAGQEQQSTEGDLKMQDGPTEIKSESGVDYQSLLDTIAQSTSTAPANGTITTTTTAASVPDSQTTSLPSIPGLPKKPPPLDAQNAFSAYVEHAQAQQQPISASNQGLSSSQAAANGYTANTNVNSTGLAHAGHPIPPNPFDTGMTNEQAIALHMQYSQQGTQQRQPTPPSRIATEPLDRPWSPTTQAAYDSFLESERRYVTEGIWDRFPIGSRLFVGNLPSEKVTKRDLFHRFHRFGRLAQISIKQAYGFVQYHDAESCKAALDAEQGVEIRGRKVHLEVSKPQKGSKASQQQPVKNQNQIPHQNQPQNRNRDRRRSRSPQRRSYSDFRDEPTRRREDFRDRRSPSPPRSRRGDRAGSRDDRRSPMYGTNFSPPQPAAYDDEASLPYPRRDPRYAPDVQIVVLDAGVPHAFVSWIEDGFKRKGLQAATTWLNQRTPLPAVVKRQIMEGVQAVVKLASHHQARSKIPLQVFDRSHGTSNVTFDEYVDLDVPVAADVVLQARQRERGSIQPPRPQFPPAFQPPQSPYQQQPPHIPWQPPHQGMPYLPPQHQPPTPQQYQYPPQPPQPFRPPQPSQPQYPTPNSAASQPSNLQELLANLKGGAHGPPTPQSAHSTGPPHTPYGQPPPPQYPQFPPQQQPQYGAYSQQPYQQLPPQHNGSGGGGQQQNMQYLLDQMSRNR

>CF317_000478-T1 CF317_000478

MFRGGVNLPHRAGYLHLKRVGTRLNQILKLRENGMHTVASNLCRAISVRSRMLDLAGREVKTVNSKAQLRVSLDQLLPMLPTVLHDGIRKSTYYAGKTNSLLIQSDDSRKQAANKDTCYRKLNELIMDVYNHSVPGETSEEQKDKVKKLQRAENEARLQMKKKQSSKKAARQKGRGDD

>CF317_000479-T1 CF317_000479

MPGLSPPQIKTQASTPNIANVPSPLSASEHALQSTESSSTYPDPSIFTFLPDLYLLISRLSEIRNAPQAAVNGSTHRDSGSGTNHNLTQTISRETQHSNRSGSGSGSGGQDVSIEVRDLPAHIYKIRQRIAEAKEYVQGIPDVDRSVEEQEAEMRELRIRCAGLRGRLGELGSIAREGNRNDTAMGGDGLG

>CF317_000480-T1 CF317_000480

MDAFVDDAAVLGDEEDEEELDEETGEPRPRANGADRDLGDSSEEEEEDDEEEAAKIAAGFIDDEDEEDEDARRERRRQKKKRRREEREEEGLDEEDLELIGIRTQREEAEPKFKRLKRGPREVEDRVRDVNDIFAEEDEDDDIDRRPGRLDRRGVQDEFEGFIEDDVFSDEEGQQAREDEEVARPRRRGIADLGLAADAGLDEQAMEDFREAFGDGTDYDFALAAEDEADEEEQEREKHLDLKDVFEPSQLAEKLLTDEDNAIRNTDEPERHQLARKPYKHIELSAADFREESAWIAKVLRAKKSIQPDLQEPFQRAVAQVLEFLVRDDYEVPFIFQNRKDHLIHANRRQVGRNEDGSPRYEMDANKLLNQNDLWAIFDQDLKFRAFVEKRQQITKTWDDIKSTGSDISDAVFEDMLPQANTIEELQDLQDYLYFQYSAQIKDVASTATDGETNGSTLVRKRANASTIYEEIRSSKVYHVVRAFGITADAFAKNAQQDGVKTYAEDPSEAPDNLSDQCLDENFSTGARALRAARSMFAEEMVTSPRLRKFMRMNIYMGAVVDCHRTEKGLRRITEDHPYYEFKYLRNQDLRAIASRPDMFLKMLKAEEEGLVEVRVRLREPERLRKELYKHIVTDAYSDLAEAWNNERKSVVSAAFDKLISIMSRNVKENLKNECEDSIALDLREEFSRKLDQAPYQPRGMKKGTIPRVLALSLGGGQSGRDQVSWAYVSDDGRVLENGKFTELSPGNEERNIADGKDVAAFVEIVRRREPEIIGVSGWTPETRKLIANLEEIVRNHNLRGAPYPDEDDRDRSDPLEVIVVNDEVARQYKSSTRAREQHPGLPELTLYCVALAKYLQDPLKEYAALGSDLVSVTFVPAQTLLPTEKLTKAFETSLVDYVNMVGVDLDDAAADAATSKLLDYVAGLGPRKATNLLKAYNAYGSEFTTREALLIGDHTPLGPKVWANAASTLYLRFESTDDAAEFLDNTRIHPEDYDIARKMAADALELDEEDIAAETRDNGPGAIVKKLIKDDAADRVNDLVLDEYADQLEKDYNARKRSTLENIRAELNDPYEEIRSPFLLKLSEGEVFTMFTSETRDSLQKGMNVPMQVKRVTEASVEGKLDSGVDAYAMEGETTDPGVNPKNVYAPHQTVLAHIMSIDRKAFNCVVSLREEKTKRPYRRIDANEYAHDQWDDRQEAADRKMLEQKTDAQGRVMRVIKHPLFKPMSSKAAEEYLGSMNRGDLVIRPSSKGNDHLAITWKVADGVFQHIDVLELGKENEFSLGKTLRVGGKYNYSDLDELIVLHVKAMSKKVDEMVNNEKFQDKPKAALDEWLTTYTNANPKRSMYQFCINRERPGNFHLCFKAGQNAKLMDWSVKVIPQGFQLMNQPYPDMRNLCNGFKLIFGNMQNASMMRR

>CF317_000481-T1 CF317_000481

MSDPFKARTLKRKNVKGLALSAPQPKQPNPSDGDAQIPGALGNTESNRADTLEIGLEFKLDLRSEDLIVLKELGAGNGGTVSKVMHATTKVTMARKVIRVDAKENVRKQIVRELQVGHECNSPFVITFYGAFQNEARDIVLCMEYMDCGSLDRISRDFGPVRVDVLGKITESILGGLVYLYEAHRIMHRDIKPSNVLVNSRGMIKLCDFGVATETVNSVADTFVGTSTYMAPERIQGGAYTVRSDVWSVGLTIMELAIGRFPFDAQDSGPGDRASSGPMGILDLLQQIVHEPAPKLPQSEAFPAILDEFVAKCLLKNPDERPSPRQLYDHDHFVQAAKRTPVDLQEWAVSLMDRHNRKSYLAPPAPKSLNSDGSTADSWNTSSTPSTSSTGTAGTVPTPGSARTPVTARKLMSPTGEITVVHPQSPPRQIPRSAGMPAMSIPTGIAERSPPPPLSLQHLSLETDDVPKSTGRAVRGFDPRDNIPEPASAIEPRRPMFPPRVSSSNNIPGSRLVGPFNATLPSRYPPPAGSLPTPPHKDLPTPPMYGSPGLVSPRRNR

>CF317_000482-T1 CF317_000482

MPPKASNNSDDLLAQLDDLSTQNQPRPRQSRPTTARAPKSNQGPGQGNKSQTEQDLLAELGNLAQRPSSRPGTPSLRSSTRPNDNASPRRAATPTITEDKLTSSDRKSADSARSSGNGAQAYTPATTTADDSPAEEATPAPTAQQSAGGWGGWFTSIASSAVNTAQAQVKNLQHQAQHQAEQRGINVDAKTFTDQLRSNPSGLLKGLGVSTDTIGNLRSMAMPTFQNILQTIAPPISSHERLAVHITHDLRGYPSLDPTIYRVFERVMAQVEGGDLLVVQRGEEAGPKRSSRDVGSRIGISSIGGAWQDGPWWRTQGERSTNPVNGVAEGTKLARANAEGYATDFFANKGGVEEAAKKATETLSESNPTRDSQIFLAVQAVRQERAGGSTSGGSDGAEEKDKEGNERDADATNRDEFLFAVYLHDPIHGIAFHAVSQSVPMQWVEWLDAPNDVEGALPESIQEIIGGGGVDPREWVSEWLEETLSLVAGVVAQRYVARRMGVGEAVKEKGRLRADMNTAQMAESGGGEAARAGL

>CF317_000483-T1 CF317_000483

METPGPLVKTAVVTLLLTSTLLPITRQFFLPFIPIAVYLIWFYACRFVPSGWRPPIWVKVLPALENILYGANLSNILSAHQNTALDLLAWFPYGLGHFGAPFVCAGFMFLFGPPTTIRYFGYAFGWMNIIGVTTQVLFPCSPPWYENMYGLAPANYSMKGSPAGLGRIDKLLGIDLYTSGFTASPMVFGAFPSLHAADATLEALFMSHCFPKLTPLFATYVLWLWWATMYLSHHYAVDLVAGSLLAAIIFYFTKARFLPRIQPGKKFRWDYDYIECGEETVARHGYGLAGIDTSNPDSDEWTVGSSSSISSGSLSPVDDNHQVWADVYHTER

>CF317_000484-T1 CF317_000484

MPPKKNVPGDQPTRPSQRTGRLAQENPIPYKHRDSDIDANTQSEGHDSSQPSSSSLASEADGWEEIDTSELEQPQHPTASNTAGSEDLSLDQQSSSIHQQLEPQPQSHTQPATHPTPQPDQTTSPPQPASSSQQTPPQAPNTPAYTFSPTNSSLWQTRHREAALARLAQPFRSPPPPPPPPRPLSAWRPTRRCTNCIMNLVTCDGAQPCGRCEWQQEWIDSTEYPCRYE

>CF317_000485-T1 CF317_000485

MTITTPNPRRTSPRDVNRTQDGRKRQKLSHDPQNNSHVEDNHKLGDEVPRVLQGEESSSSDQSAGKWFSQVNKNGLTGQKQSAELDDEPPFYMSDRMNYIPNVFAASNDGRFGVVSRFDSENEDLRGVIDDLTVENRRLKQMIRTGNRQRRDSPNASNQEQDKLFEVRMHGLPSEKRRELEFLLKNFATSVHSSVPSLTTATSNASSGMNSGVTKSRAPQTDSGYASASGTTSGGTSNLASLGPAPVKHSTSKTVKTYLKDIPDTLLPKQNPLMTERAKQALVVRRLEQLFTGRTASPGDHDQPMQQQEISDSAARADRLQDARENKKKKAEGRREARVMPPDSRVNLDAMEASDTKGTSPLKERKDVSAPGSPDQRPTRPLDLDIHRAQFAADNLQYLRHLGLSTPQVAGSIRDGEESPWMFLTLLGGLAQLHTLNVTPEFVRRAIKKLSKRFELSRDGLKVRWTGGSDGTRFAADDEKAIEHAASNPQDFAEDTGTGSSSKRSKTNSTSNVAGTLETPSEDKTSGLQTSSDSKQHQLTLSGTSNVQSSSNLFNPSATRSAFDYKPIVLREKQLQRDDLQSIDESFDTSSGDSSGLVSALNKSNLNQGAKDEGVITFYHNPHFCSDFSSEMEPSNMRSARPVVAGETLGMPAAEIVDSPLRYHDATYFTTQFAPRPFDLGKLSDEQRDKFDASLPKLPDLPAMRAAGESETMPLEFDVCGLGGIIPEDNFALDVKVSVKPAPTRGPGIVVKQLPFSHRKHLQKFTAKVESCEKLELLPSKLPPPSYVFFTSSSSSDAGDFDDDSDDESDGSSSPLADEVCPAPPAFLQRSSGDSGEDDGDDEDNDVESEIDMLAIARQANPEQIAEQERVYMLHQPGGALRGVAGSLAATVGETSSASSMHRVGAVTPTENWSQEGEDDEDEAEDDDDDEMDDA

>CF317_000486-T1 CF317_000486

MASLLESSSRLTSQTTRCLRSARAPRNARVREILLTRHFSSTQHRREINKIVSSPSEAIKDMKPDSVLLCGGFGLCGVPDTLIDEVSKTPRLTGLTAVSNNAGIPGSGLGKLLDSRQVKRMIASYVGENKVLESMYLNGELEMELTPQGTLAERCAAGGKGIPAFYTPAAVGTVVQTGELPLKHNKDGSVKEYGQPRDVKIFNGKAYVMEEAIPGDVAFVKAYKADKLGNCQFRLAAQNFNGAMARNAKMTIVEAEHIVEVGSMDPVAVHLPGIYVTKVIQSTAEKRIEKVTNRKEEGDVKESLGKGDTASKRERIVKRAAKEFKNGMYANLGIGMPMLAPSFVDPSVSVVLQSENGLLGLGPYPKKGEEDPDLINAGKETVTLLPGASCFGSEESFGMVRSGRINMTMLGAMQVSAHGDLANWMLPGKVKGFGGAMDLVSNPSKTKVVALLEHTDKKGGPKILKQCEFPLTGKACVSRIITELAVFDVDFTEGLTLIEVAEGVTVDEVKSKTAASFKVADNLTTMEQ

>CF317_000487-T1 CF317_000487

MALTVGSITSSKGVPKSVAQITKDAAEYEFDAIVPLKYYLRTANAMVRQAQVYMKEGDDEMTFFLLFRHAHLCLSHLATHPQYKQQDPEIIRALEKEVSQNLKAMELLKARINRRYEEYTKVSQTRQTRREEDVAKRRASQGPLYLSHDQYRPDSQPAQIRSQEHSNLAARLAGQEFARRAPSRRSYPTALSQPDEREDLAARMQAIRTRVEPKDRDGAIEANQQRQTNLLDNAGYSYPAVPAQRPLKAQSHPPEAIAPRPPYIAEREQPPVLPPKPVQSSRASTLPPRPGKVASPAVPSNTPSEPAYTFAPGAQLENGTPLRTIFLPPTLRTTFLRLAHKNTMKNLETCGFLAGTLRANALFVSALIVPKQTATSDTCEMTDESELFDYVDQHELMVLGWIHTHPTQTCFMSSRDLHTHSGYQMMLTESIAIVCAPSKGDTSRGGDWGVYRLTDPPGKMAILQCDKPGIFHPHDVDNVYTDAMRPGHVMEIAQMDFEIVDLR

>CF317_000488-T1 CF317_000488

MKTWCSYRAFQATVLGLLVARTLAQTSVETTAATATVELGSITTLGCYSQPDPLTQDNTDTFQTSGSCQVACGNAGYPVMAITGGSTCYCGNMLPALDLQVDNTSCNSPCNGYDQQNCGGIGYWQVYLDGLSGNVETAPNATTSSSSSAPTSTTASATRAPETVVVTASATARVSSIPTVSAAATDAGPNKIGIAVGVVVGVLVVAAITGVAIFFLRQRRRKQIEEEHKAQAINSFPGSRSSDTKSDARLDPSVASSYRRESIGSIADERDFSRRILQVRNPDNRGSLASVA

>CF317_000489-T1 CF317_000489

MSPIIQALVVDLGDVLFCWHPPEDGKISPKVLKRAMSTRAWYDFERGMLSEEDCYSQIGALLLIPRDDLGDTIKQAKMSLKPDQKMLRTLRDARDTSYGQTKIYLMSNISQPHWKYVQSIYKDWDIFDQIFTSAGANMRKPDLCFYEYVLESIGLTESPSSALFIDDKLENVCSARAVGMQGLIFDNTDNVIRQVRNMLGNPIARGTGYLSSNAKRLRTTCNTPMRCNIDMKENFAQLLILENTHDRSLVAFQDYRMLWNFFQDQPILTTTEFPNDYDTTSLALTILEGHHLPNVINQILDASLSNQSPDHIPLVYDDPSRPRFDPIASVHIYTLFHLNHRSDELAPTWNYICDFLSSGAYEQGTYYYAPPEYFLYALARMISYSRHGNMPLPTLPERLASNRKSNAKNFHHLLKTRCRERINTGPSNAIGLALRIIACRTAGITSADLQSDTERLKALQEVDGGWPWCEIYRAPCARASIGSRGVVTSFAVRALKMMDEHELDDGAATRNSASILKEAWKRVLGGWWPWVWRAPRSLRGTVSATLSILLGTAQYAFDATAHVFLRSGSIRSTSCKVLALGKRRHESH

>CF317_000490-T1 CF317_000490

MQLKALLSLISVFAPQMSKHEIDTPFAADSKADLQHVGERSHVGHSNAPAWSDQMEVDRSSQGHTKSSDNEVDMADARRSEDGPQADSERQNTIGGLLAALATNATTDADEPPAPAPAPAPTPGPATATPASATATAPATATEAAPQCTSASPEAPADSPISASPNDSQQMDEEGMTDNSNARLLSVEPNSGFHPSTIIMPADPSLMLQNMQPLPLYDEDDMQMPFPPFDLTMDQFPLEPIHDPTPVNIRRQYDAFARLEFADGIFYLNTYLCELGRDQHAYKDALRREREAQEAAQLEKHQPNSSSGKASQRLHSIKAAESQIQGSVVSERGGFAGVDDQPFETYDGKRNGQHGEKHSSQASNESVVRPAEVLHNPSLAPFDYHKDVTYHPAQPVPVPENNIVEDEQPAPVTADHMPDPSSCPLIPIHPTLDSGEQSEADCMRAISRRHVRIYWNWDESSFFMKVLGKNGAFFEDKHFEEGKCVKLHSGARIQISAVEFTFRLPITKVESPAEDDASSLPSDAALDVSPNGLTETTENGQAVKLKLKLHADGAASSATAADAEKKARRGPGRPPKNGYMSQREMKEIEKAEKEKQARALHGGPSPPILQPRKSSKSQLPKPEIMPEAPKPEKRKYTKRKREDGEEEEVMPSIEGQEEVPVVENPPPQPTTKRARTKSYSPPYKPLAECSQEDLARPPHNYAVLLYMVLSDTGEITLRQIYKQMQSRWPYFKHVVDSDGWTSSVRHNLNQEVGKLFERGRKEGKGFTWLPKPNAMEEYQAQKNKRSNAPPAPKPRPPPQRPNFPPNQGQQLTWQNSGTLPQSNRPGDSFVHQGPWPPQQPNGARPAQPGAQMNGSMPPQVNGQRSMSGQPPNDVLNNNPNYIPPVNMLPHYFGRQAPKFIPVTFEGLAVINRFQQSMMSNVGKDEATQNMWTAIFDSAKKRCLHGATASSLEGGENDDERTIIGHIKTFVGRYQNPAFEGFAARTASPATAGAAVRTGGPSTATTTPGPSVSAPGQQSHVPLTPQPAQVPPVPRQPPPGSQIAQSAQSAPPSSQASQSAQPVQSAQATSHPATGGTPVNGRQSVPLQTPTTTVPPSIDPGVASHSHPPPTSVTVPQPRTQVQSLPNGLPDPIMSRPDAAPPVPAANASPALNPGKGSVVAAQASSAPPPGPASLATVADAAHIKAETTVASEKPAMVGEAESKLQQQLDKDNGNGEPGISGTSEKSPEDQPTTAMSSEAMNQHLPPSSKVDATDVPSHTGGSSNLQESGPQLGS

>CF317_000491-T1 CF317_000491

MPPKQKTKAKGNNPPAAAKPASPAPPNWPQLKPVLAASDLSVEETLKDQILVVPNLFTANLCKTYVTFLSTLPLTTTPGKPKRGEATRVNDRFQIEDPAFAQRLWEDTAIKELVASYGDKDIWGGDVLGLNSNIRIYRYRPGQFFDQHYDESNRVFGQGGVPAKTTWTLLVYLSACGGGETAFYPEASSRRDQQPDLVLVDPQAGSALLHRHFPECLLHEGKEVVSGEKWVLRSDLIVRR

>CF317_000492-T1 CF317_000492

MSSGLPADFKSYQDRVTSSLVNVTRSAGGIASHDLSFHRSLSDKVSKSLDTQNTHLLRLTNKLLKAATKETNLKSLTFRDRDDLEDNWKNVVDVIDDLLEKADANLDEFNGLIKRQSPSQAATPEPPSTKPRYPAYHSTAYGGVTKPQTLFSTQVNNYDNSRWRPLLRTKPHAQVPLEDSIGSEETGFKHPYAYEIEKLEYPPALYKESLPIPFSRPENSEPVWVDTMEGVREMLEELKQAKEIAIDLEHNDRNSYVGLVCLMQISTRSKDWVVDTLRPWREDLQVLNEAFADPSIVKVFHGSASDMVWLQRDLGLYVVGLFDTYHAANALQYPHRGLAYLLQKFANFFAQKQYQLADWRVRPLPQELLDYARSDTHYLLYIYDNMRNELIRQSTPEKNLVDYVLMQSKKESLQVYERRVYDPENGLGPNGWIQLLLTRNVKSLDKQQFAIFRKLHEWRDQKARKFDEGISSIMTNAYLWSCAEVQPETRAQLFNTRAMSGRASYFVGQYSQEVLEKIREAKREGLEGPAVQEVLDRNADKLASFRQFRHPNPHAKPQEVQQSVAATMQQLQQSGELQNGTTTHEASTGEPLASRSASSSLWGMMQPDTTQSLLDPSTAHVALMSIMPLLGFAKESDVPVITDQATTVQSDLAPLPPRPANGEARIVEEPDIDVPFTLSDRKKKRNAEALDPSEEMAQFGAKNPEDFAITDEGEVLTQRQAQKAFKKAKKEADKAERDAQAANFQPFDYANAQSVLHPEPEPLSTYKDSNPTKPFNPFAKALDTSTGARRNKFGKELAGKSHTFRS

>CF317_000493-T1 CF317_000493

MWGWFGGQAAQRRRDAPKNAILGLREQLDMLQKRERHLESQMAEADATARKNISSNKTAAKAALRRKKLHEKTLEQTSAQITQIEQQIYSIEAANINQETLNAMKNAGKAMKEIHGGMTIDQVDKTMDELREQQQLGEEIANAITSAPIGEPVDEGELEDELEGLEQEMLDERMVKTGALPVGGELERAPAVPNVPVKGSKVQEDDEEEELRKLQAEMAM

>CF317_000494-T1 CF317_000494

MAQNSSNAPVASNLVFLYQLNDQPPNTKVRFLCCVVDYNEQNGCLTVEHRYPRVVTGTPCISAVVDVSLVLETTRCSLLQTGSWVNVVGYVQSVAMVKRRISSAKANRQSNVETSLPKVQAVLIWDAGAVQLDKYEETIEQYLAITGTK

>CF317_000495-T1 CF317_000495

MSRRGLAAFGLVAAGGAGYYLYSAGGDPKAAEKLAEADAHKASAKLRGDIEPRGREAGARGEALVRQAGDKFDNATADAQKRFEQSKNDAQKALAEGRKEAHDAAVKASAEAKREIDQFDKTVTEGAQKAKSGLSSWFGGK

>CF317_000496-T1 CF317_000496

MSAEVAQTRTVDQSADTDDGQIRLVDTTVAGSDENPQLTSTGSWRRLARIPTAHTVKEELSRRKYRSWRQDRFQTEDGSADDGSTPQRPGQPSAPVIQAHPENAESQAVDFGGRTDAGDDVADRSKPKDLVARKKNEKPESHIDILYENQRGSFFCGIPLYSHSTLLQFDPAAWVNREFRNSPVNITNAQVPDPSWMWAWKTWYVDMSGDVDEEGWQYSFMFGNNFAWHGTHPWFHSFVRRRRWLRKRVRRSEATDTKDAMETGHHFNSDYFTIHSRQRGRSPSEAGNPDTQLPQPVSYHSFPPIDEHSELKEPDDINNIPSLMKALRRATIDREKVDAVKHFIDKSGDELIYLKEKMPEIMSQLVFQNTRRQILQYLKDRAEEAQQHRDKHDAEDRPEGPNEKRRIDNLLKAAEAADKEISGLEYWSDRKHVLQTADEADDGQDPKELQSKNVLGEIKGISEKAETAAQIRVDRRLAQRSDKGKQPERQESPLNDGKREMPAYGRDTLLVQDEDEETVESDENAWKGQSAAPVNL

>CF317_000497-T1 CF317_000497

MAPSSRHRISKRQARPDNTAVFRVTKSNKLNISKQKLDAIEEIIVEKEPPQQQLPEQNSSTKKRRRDAVDTETDNEEGATKRPQKSLRKARQTQIQVLTPPASSPEPENETLPDTLEELITLHKSFTQALGVHYAHNGTRNAVSLFALMPAMTRLWKRRAVSVADVERMLAMWEVSANPAKEVEHKKGPFRLISTGIGSNHQTKIEYAWTNAFGTFVENELHQKYEAAIERLWQSAQAKPDAFVFIYEPLITFPRLTCRVGTQTQARQEKITSIRDTILSKSVKTQNQPSQSEPDFSKLQITDPSDPKPAPASREDKLKSRTLSLFDRLRAKQLANSSSSPQDSASQLRRRALHRVPDLIDTLRLKQSQKLNKLFRSDLPESSSEMSVRQMKVSFSLEQLVQEIRDSGRVPIAVEEIKECINILGRDVPDTWCSFYDGDGLKCVTLQGDGWKREAVREWCEREVRKMDGN

>CF317_000498-T1 CF317_000498

MEGPTSTGLLDLLLQQPPEIRTAILDNLEPRDLLSLRATSHALHNLVHESSGALCVGLKECISKTNDMSYIHIHIPNLSAFLQVSSRYRSACQVAAIVAERIARHVSPTDLRLDKRALEAWRKKKSRSMERRLRQNLVVLELYLMFMLQNMNDNEGDLQPLDDGEYKSLHNIFLFDEQAFFRRHMPGLTETDFVDVETTLDILKMTCEARCVPFKMKSPAYPFISVRHILIRKGLAPLAELLAEDAGLAAQETILRRQSRNMRQYRRSHTTRKPHDPCSALHALEGYWQTQAVAFDVRRSSEARENFVSHQDIWDKSARAFIQHKSERAPLKQSATAWIRRVCAEDDKSMKQSVVFVGDWAKPGA

>CF317_000499-T1 CF317_000499

MASYDSDSSSDGDLDVQTNVTLGYAAQESTGDDISHVGGHPTWLDPNAKPSAALAKCKVCNSYMSLLLQLQADLQQHFPDDERRLFVCVRVFREVKKAKISSKPVPPKQEDNTPASKPQQSLGSQLFGGSPPPSSSNSNPFSAFSQPQANPFATSSPSNGNASPFADLAAKSPQPPTQSFADKLKISSSPPQASAQKQEEPAQQDPWPSEDSFPKPFTRFYLDAESEYLDPSPPSNAEDPSTSRITELDTDPSTSAAEKDLYESPHDKTFDHFTRTLAQNPEQILRYEFKGTPLLYSSTDSTATHFVLPGHHLHQKVKTAGPVKGLPSCQYCGAGRVFELQLVPYLIYELEKDDEEAMKLDGGEGMEWGTILVGGCSRNCGGEGEVVFREEWAGVQWEEGGVELKQK

>CF317_000500-T1 CF317_000500

MAFFKDLRRRSRASFRTSDSSKDSNNSVPSQKSSSTLSSAYTSSTPPSSHPATTSTPNLSTLKTNGSSPAPPVPTRPGTMSGSNRNSVIVTSPNGSTSAMRLPQPSSPFAPKITSVADNSVVHSKIIVVSGEISEPHLRPMDGNVTVHHDKDGYAFPPTNWPVSDSHFKALVYLSPGWNKLRFEFTSPKLSQSSNGFNQTHSSYLMLNYLPLNSAPPLQLVILVAKDSPCTFDATPARVQNEGNDLGVAIRKFRMAAHLWQAFTTEQMYRHKFGRRCFRYEEEWQTGTLSIRDWENGIQKNETKIHIVRCEHTVAELRDTNLAQQNEKAAKKGDLFGIAGDAIRKHFNIRPGQKHYVACLILDSHWDRQAGTITAHAALGGGSDDLKLAIFGSHALQSYPSCVEEVVPALTDCTKTDTDFVANDCNESGSSWEAANIGIGAHLHEVGHLLGSPHQESGVMLRDYVRLNRTFCIREPYSTRTKSPGLRVCKQEDECGWHRLDVLRFRYHPCFRVPGDELPITADNGVQYYGVDGGRLIILAPSGIAFVEIRIDGADLCHQWSEYLSADPRNAALPRQVTLTEADIRAKLPEDKKKSRVSLQIWSGSATNVEIKDLGEVLSPKNIIALPPAKLGPKQDPGMVMSMVGKSFGDKRQGFKGKKLGASSMQGSKPHEIILQSCIYQSKLLLSVKVWAGAALDGLEFCYEDGSTQLFGKSGGSSAEFALDTRRGETILGFDVRAGAWVDGIQILTSTGRRSGWYGNPNGGSGHTLMAPRGYYLAGLSGSCGPWVDGLQLIVTR

>CF317_000501-T1 CF317_000501

MPPTQLQHMDANHVAEKITRRIEIAKVARNLQDCLAFASFKSINGWEARTLSSIEPEITERLAKRKRPYPEDLNSSDSSITLSEDILGPSSQPLVSNSARSYAESRKRIRACSSSRLERGALHAGWEQNNGMTRSSPLLGPPETLNAPYSTLQDSPMFDAPSVSEDEDQDVGVPMMQEPPSSILSSSPPRTPPPARRNLANTKQAGADLLLYLANSPRTPAVHVHHISSTSKAHPSTPPHQHANLPSSFVQTPGNLGLFNGAVQTPGNFNLAEFCNVTPSPAQAQFSRHDTPTFGRTPQRSARRSLNFDALIPPSSSDQRKTSDGLALQLGDALERQ

>CF317_000502-T1 CF317_000502

MPSETTFVDAPARVKKKHKKTRTGCVTCKGRRVKCDEAKPICDRCKALGKHCFYRPVPQQTYRGSPEEDDLVTRSPIPLPELALSPVERTQETAQLQQFCMNTSLAVFKNLGRPFEIFWTSAVPKLSFSEPCVAHIMIALAARQRASFYVSNASFERGHLIEVYRHHYRDALKLLVRPDAGGRTEMVLLCCLMFISMENVEDTVSNSFLHLRSGLEVLREWKSSRPPSTTSANDLIEQTLEPMFARFEAAVSPSIYDSDQRPATGLRWPMPELPDRFENLATARGALYEIAQWVFSQGHHQMVFHKPHSQEFSQTLELCLKWRNILDKYVMDHPADREKYSSAVLALETNHQLLVLLVKCTTLPNELLWRGYSGMLEEILCNVEAIDEQGPSELPQRRSKMDLLPRMMPALFAVAAISRRRATQKRAVEIIKRIHAENHSDQCFVAVIAEGCIQLAETLEQGSVMELSSMDSRIRPLMAELVPGRPGKIVLTYTRPFLQPVIVDDPELGPIVAGAPKETIQLPWKSRQAPPARVVNLWPVIEFLRLSGSSGLVGPNTGHCLCKTYGALFALMR

>CF317_000503-T1 CF317_000503

MYEQCRGTTFFVTHQATQLRPRACHFVSTTTAFRRSFSISRFQPQRVPPVLDNSGNNGRTPRLPLSNLRVLDLTRVLAGPFCTQILADYGATVLKVEQPGLGDETRQWRAAGETAQMWNDDFLSNSNHGKGGDAPLMSLYYSSVNRNKRSITLNLKSDRGRALVKEMINPHQPNAYDIVVHNFLPGKMEAMGLGYTDLKEVNPGLIYASVSGYGASGPSSKRAGYDALALAEAGLLHITGPKDGGPTKPGVAIADICTGLYTHGAILAAVNARSQTGAGCKIEGSLFESSLSLLINVGLASLNLDLDKGPSQRRRGSRFGLGHPNLVPYGGYKTRDGRQIFIAANNNRQWKLFCERLKLDQAFVEKYSSNDKRVESRSTIDQVIGDRFAEKDLREWEAAFEGSGLPYGAINDVVQALEHPQADARDMVVSVEGIEAARDGMVKLIGPAVKFDGGAGVANVVRRKPPLLGEHTEDVLQEMGYRKDEIQAMRHECII

>CF317_000504-T1 CF317_000504

MPKELKKRGRRAAKQNNEPDAHDGPPTKKRRTEESQPSFAVNGDAGDDLITFGQDHENGDQDAQQTTFYGLLTEEEQEYYANVNNKIGANDFENEEDKANFIEAVHRESSGKEIKVASSQSSSRYLETIIRLSTANQLRSLFLAFLQDLDYLVQHRFGSHCCETLFLESAKHVDVKSKSTEDGNTSFENLFLEAAAKLQANIGFLLTERFASHTVRVLLLVLSGQPLQDTAAKETVGSRKKEKPGHSQHPESNSTDARKIPHSFKKARRDLMVAAVSGLDTNYLRALATSPTGNPVLQLLLRIELSEGAEEGQNSHVVLHKLVPDQNFEPESDSSKFVSSLVYDSTGAYLVQALVRNLPGKSFKKMYRNLFRDRIGKLAKNEIASYVAISIFERVGKDDLAHAVEALVPEIPGLVERNRLAVVRTLVERSAVRGVDLLPIREVLIQTYDSDPKLRLQKMLKLEGLAPSAAENSDEKGNEKLSVDLQGSLLAQSMLRTSQLCEFVQDSLLAQSDSMLLQLAESPVASRIIQVALTSEKSSGKFLRRFIPAFDNMISDLAIDVAGSHVVDALWNATNGSHFMKERIAKALQSSEAKLRDSMYGRTVWKNWHMDLYQRKHREWQAVAKGFDQEPTQEHDSRPRKSPIELARERYMQKQPKKNQQHGTGANTVKTNA

>CF317_000505-T1 CF317_000505

MPAQAYFEPNCERAIQLEELLSNAVPELVRFVNDAEQERSQHVSGGSQPSDTDHCTSLVDPLSKSWGSLESTLKSDGLLAVDEQGSGAQGLQQLIEALLKFSVNTSAPGFLDKLYAAPLPPGIVAEVLLGVLNTNLHVYQVSPVLTLVEKHVTRNMAKMFGLNGPRSGGISVQGGSASNMTSIVVARNTLFPKTKVEGNSAVKGQLLLFTSAHGHYSIEKAAQALGFGSSSVVPVPVDHFGRMVVSELERLVIEAKRQGKVPFYVNATAGTTVLGSFDQFEEVHKVTKAHKLWFHIDAAWGGGFMFSANPELKERLEGTGLADSVATNPHKMLGVPVTCSFLLANDLKTFQKANTLKAGYLFHDDNDETAQSRDLAKGDAPGSDDEASEDDWNEPYDLADLTLQCGRRGDALKFFFCWKYYGTSGYSSMVDAAYQNACHLATLVDQSPDLTPVLSQASAPSCLQVCFYFTPGGRFTHELRDDTGQIHTQQSPGDQTVGRTRLARLGKLNSKVTSSIAKGLVPKGFMIDFAPALEGQEDRGSFFRVVVNISTVRETLDRLIQELVSVGTSVVSNIPR

>CF317_000506-T1 CF317_000506

MFANRRSSQKPNEGFYAQFKQSFPEVGPGTSSAEHGQGVPVTSQLGSSSINAAIVDPSHAHRPVFDSVDDSTKETGTTPKPFSDWRLTPSLMDPNSYAFAAFANQPPGYYTPTPGGFNTLWHSSSAGDLHTPGALGLNTPLSLPQSMHALHASDPTMHFGHFNPQMLHPQQTYQDAFHQQSHHQHQHLQPQHNYAPPGLFLQHQDSGYVAVDDSSKGPTPNQDEMQSIQQKLAPPPMQQHDSGVSMPAYASSEKYVPTAEVTHDPNQVTRFRYHTTLNAPTAMVRSSDEIPVTYLNKGQAYTMSIYDTIPSSNTSNVRYRTYVRISFEDEQQRAKPGACWQLWREGRGSNEAHQRGGKLLAVEYVDPNQGGDDETRKPQIELERASFDGFVVNWYPNIRANGVADCSISVRFNFLSTDFSHSKGVKGIPVRLCAKTELLTESPDASNNEAEVCFAKVKLFRDHGAERKLSNDIAHVKKSIEKLKQQIQQAEAGIGTIGKRKRSGSIAKGAASRSKLSKHKRSWSADSDAEAGRMSAEEDLHMKLAGLQDMFSSTRPMSVLYLKGDTEDDPDLCPVKLTSGDCIKPIEHTKTWDSRQSGSESGSNPGSPTPSSLSASPKRKLSELQHPSITEGEEDIESKQVVLDRSASPERPAKMIKREPESARSDLYALDVDNNYQPPPDRPIKPVACFYVKDKDASKSYYRAVYLLRRTVHDLVNGISEKFNIDPQRVTQVTHTNPKGLNIIVDEDVVREVPEGQDMVVEFAPVGNDRVGNHSLDLQPTMVDGDLPPLDTTASDPLEMWLNF

>CF317_000507-T1 CF317_000507

MADTPPSRQDQPGAKVVSEHLPAGYSATFDGLSDANAGQASSSAPDLTRLSEESSLKLQGGDIHRSLFKIDQRAKLAKRSATFSHPRHSTQLDDDEEQLTAGEQRLPGGFRREFIQRKQPSLARRNAVTRNFVEFLDLYGSFAGEDLAETDDEAISDTEDEEEDDDANQPPERRPLLRRKSTKRSKAEGSAGTTKTFFTLLKAFIGTGIMFLPKAFDNGGIVFSSMTLLAVSAVSMLAFHLLLQCRDSLGGGGYGEIGHAIGGAKMRNIILGSITLSQLGFVCAGLVFVAQNLYSFSRAVAGRPDNSSPVSTDLLIALQLLVIIPLAFIRNISKLGPAALLADVFILIGLVYIYWYDITTLAQDGIHKTVVNFNPDYYTLTIGSCIFTFEGIGLILPIQSSMKKPSNFEWLLGTVMFIVTVIFCSVGALCYATFGSEVQIEVIDNYPQTSKLVNAVQFLYSMAVLVGNPVQIFPAIRILENKVFKRSTPGSKSSATKWKKNAFRTFLACLCGGISILGANDLDRFVALIGSFACVPLVYVYPPLLHYMGVADNKWSKAGDAIFITLGVAMMGYTTVVTIVSSFVN

>CF317_000508-T1 CF317_000508

MEAQVRNQPDSVQTSYDLTCPCSDASVYPTPTSDGATLAVIGHRNGLSIFKAHSRTCNDFEDIEDSIKHHAPRVSPLAEIPLESPVQHIAFPPIKPSVLSSQSSTVPDFVQANIIVAVACADSSILLVSCAVDSALEQTDAERDGTDVKITKISSSASHQDMISSLAVTWSADDVQGNPETTDGTQTHYSFLIASSSTTGSGLLVVHRLPFSRHIALQGHSPVIIARQFMRMPLLGGTLCFSPANYPSAHHSSLLVTSPARGIVKVLDVSQSRPTFKRRRASETEVDSEEPSAVPSANICLTLHAGYTPSSILPRRKEILDARWAMDGKIIVALLEDGDWGVWDMAGTEIGSHDGRRSVQGSRDSALQRNFAVCGNVSTGLQRAKTTKTKTSGPLAPMTPHTRKSRSADLFGASQPRADTKTATNSAGRISICTQQGAVAGADDALVISFDGGNSFIPSVRSLQQLGRRDNATIAGHTTTQIHALPSVRLGGERPIAISPLHIPTRKTHAFPGLFSIEQDLIVLTHSRLILHTKPEPRSVAKATAVNLPLRSAHANDSTFTSRLTNNEVLDLDAMQKMLDSMDNINDQQKSPAEVHTNLLSQQRNQTKVGDPETASPTMPKSAKSKLMITRDAASRRQNLFE

>CF317_000509-T1 CF317_000509

MAKLQSTGLHEPHALRYLISEGLLPENADSSLYAWSQQLYVNEQGRTGVEEILHTDRCVTWSRNKIIKRVFNLDIEGETILHAFSTSFPSASNGAYSPVDGGAFDNPLTPKEPALVVILKTQAHIFSLAAGIHVIPLSFEVARALPFAGGCILQGDSSLITKLPKAESGRSSARWENASSRPSKASSFFEHGSNTAPPQLHSSRRMPTYCITDLLSELGLVAASYTRRPDDLSVSPLLPPDEEVLYMSTDAHIPMNAFGDVCVAVTLNRSNSTVSVWQVEASNTKGQSSQRSSESSKYRKRQSSNIHERSRVPSGRGAADASRESFGGLIQSFVENEAKPQGPQLSSIENLAAELGQDFAPSGVQTRSARRVSSMLARTDLAPANDRSTFNDNAMTSRKSLSRSIRKEGSFGSFTERNSFGGRKSHLANTSIYSNTTSFLDPGGRLSVNEGIGGGGIDDHAFEMLNERLQKAVTFIRLKSFVCDEASTHAAACDIKTMLIPHPVSVKSDGSTQLQFSVCILEARPQAMTVVTIAVKRNVKPVHFKHRPELKAIQIQKGNNIADACQVSDGQINRLIVLSKTLQGGDVLHLEAPWSSSFRLDMPVQYLVSRSRGVDTQSDASWKHGAGIHRVIDGSKVKISRFGNTFNLNCLAISDTSGQEYRLRISLLPRKSMTARVIQLCKFILPAQLQDSMLLAYWEIVRWLELQGVTDGIESTALAVMLFILALPFINALQNKSATPGRKKKGVHMRSSSGPFVNATSWSAMQDAERSRCTSAWAQSAAWNWTSNDSPISNKPKSPSKQEVQTSLDVDHESTNAFLTRCTDLTREFLQSPAGESMSGPEGYLPTAMNQDRNIRRTALASIVVALYLLVEELRLEVLANEALTNDRAMLCALIGQLSAWLNWPAWKAFMEAQFVDGDSSLQMRLPDKPTIDNLDVPPEPFSPISIFELVDSSIQSRPLKYHTLVDLVQNSDAVQPESPLLLEAGKLLPHTNALVSLFSLSALQDKAGTIDRANAGDFPEVIAKACRHAIVTSGADSKLSKSPLVSTAQLTSDKSRPYHFRPRALHAATRDVRIMCNQALDAESLGRWDASSEMDRQAVTRLIFNQDRRFQEASKLLNQALPPEIEYEHQPNWTEADALEAQKLLTQYATRRTFAVVSGRGMMYFNARTPLLTEMVPRAQFSMQCILRPKGETEGGQSMTFSADRSMFTEDKEADFIDTSWILYNKPPELTNRHAGFLLALGLNGHLKSLARWVAFKYLTPKHTMTSVGLLLGLAATYRGTADTVITRLLSVHVTCLLPPGAAELNLSPLTQMTGIMGIGLLYHDSQHRRMSEVMLSEVEAQDSEEPSGDEPILRDEGYRLSAGMALGLINLGHGHHLHALHDMRVTERLLAVAVGTKNVNLVHVLDRATAGAVVAIAFMFMKTNDASIAKKVDIPDTLHQFDYVRPDIFLLRTLARHLIMWDNIKPTASFIQESLPQQHRHRTNLRSVKRLITNDLPFFHVVAGICFALALRYAGSQRQDVRDLLVSYLDNFIRLSRVSALNYDAQVTLNGIRNCLDILALSCATVMAGSGDLVVYRRLRALHGRTDKDTPFGSHLAAHMAIGALFLSGGAATFGTSNLAVAALVIAFYPLFPTEVLDNRGHLQALRHLWVLAVEHRSLVLRDNETGQVLSNVDVAVTLKNDTTTTLRTPGLLPELDTVKAINVSTDDFWPVSQDLDQVVTRQNSKVISAAEQTLRDAYRCSTTGINISLCRRALYEKQGVDAFDAELTALNNTHGIARATSTVGTTVSKGVGAGDEHSLGWVYHLAAFARLDHAERALVLSSSGPSTWGTDEQNKLERSVGRLMLETTPIDTGLDLDLGTLGPEQASTGMQKALKSDKLWQIRLLLSLADKMEADQGQAQQRDEGSRTWLRKEVMERLRWRVWKMSGSDGAEDEQAPREGRRSEESELLG

>CF317_000510-T1 CF317_000510

MILRQTIVRQGRTAISPSTKRVRSSRCRAYATDYSINNPQHRQSEQDHQPIRSIPGHPDEPSSLLDAIGHQGTPATAPLEGSVSAAAHDLDSLRGQVLSYVAAQNAGAVPSPKSYHDLQNELPPAPYENAQALYDAREAFHQLTSGGSTTADVYYPWSIIQNPPQPDQVTLPMLLANQCHLGHATALWHPGNSSYIFGIRHGIHIISLEITLSYLRRAAKVVQEVARRGGIILFVGTRKQMRDVVVNSARRAGAYHIFNRWIPGSLTNGQQILDRCAVKVVNAADDELPQYRDPLRSATRTVLRPDLVVCLNPLENDVCLHECGLYNVPTIGIVDSDVNPSWVTYPIPANDDSPRSVALISGALARAGEAGQAMRRQAADAGKTTYSTAGVQRYLQGMGEIASLNVQEKSKEGKPDKDFD

>CF317_000511-T1 CF317_000511

MHSLSRTTRTSKQLPCLSSSASSGPRDIRTPKCSALNEGVPHLDPRWKEIIIMTNSEYAKKSLSECVWTWETNGWHHRPAMAHLHTMQELHNIIGHIEATLHMAIRFWKTRPAPANAVADALAAMGI

>CF317_000512-T1 CF317_000512

MSSQLPTAIQKQVDEGAVVNHVSNVCPKGTKFHVLCVGWLEADEGFVVRGGNTSVKSKEKESFVNKRRELPMYCILIEHPHEGLILWETGCGKDYPEVWGPVVSDVFARVRYEPEHELRAAVEATGNKIEDIKKIILGHLHLDHAGGLDEFMDRKDVEIWVHDKELRSAFWSVATGADVASIWSTTSSYPCENWKTFDERTFDFCQGITLHHLPGHTDGLVGMQINMPEMGTYLFISDHCHVIENWRDGIPQGWLARDHPAWFQSTQRLKQLVRTTRGQVIPGHDKETLENLMAQAKVFT

>CF317_000513-T1 CF317_000513

MSAALPKPSNVFKPVKKRVDSNRKQRSKYSRLSRVKARSAVYPSHSTAQPALLLLFRVGQVDAELLDEELLGLLKSQIADALKYFNPHIQDDYSREVLLALRAVLFKLSIWDNDASYGAALQGLQYTDARQSIISGGLAKPTPVQKGLYGLITVVGRYAWDKYEDYLLEAESSYTGPSDLIRKLSSVTSRLSTAHSIAAFTSFLVFLVNGRYRTLTDRLLRLRLISPNAQTHREVSFEYLNRQLVWHAFTEFLLFLLPLVGISRWRRWLSRIWKNTKRALTTDPTSQGQESEAPEKKGPLAFLPERTCAICYEEQNPGGRSEAEVLGANTGAGGGVIGSVQTDIVNPYETMPCRCIYCFVCIASKIEGEEGSGWVCLRCGETVYKCRAWKGDVLVPTSKKDSAKKSVGFVEDTGEGDEEQDRDEHAEEEEEEEPADAHPSEIDAELGSSQWQFEGERERDLTG

>CF317_000514-T1 CF317_000514

MPSFAIEAEGETVPLSVQDIFQTLAQTAGAQATQQSLQVSTKQLANWERTPGYYSLLQEVYADFSLDDGIRLQAIIQLKNGIDKYWRKTSQHAIRKPEKEKIRAKAIEVGVQEPKRNLALQNALMLAKIVRLEFPHDWPDVISTLIQHLRHASASDARPEYVSNILNITLQVIKELASGKLIRTRKSLQSVAPELLHVLGQLYITLVERWTAARDLDEPSMLNSHSALKTLRRLVIFGFEHPHREESVKQLWHVLQSHCDLFFSTWKQNQENNLLPKHLFQINKLWLEMSRQHPASFVLLGCMDILKRSWEVVNGNNAKEALSAGLDWSVHSNGDAGNDESPIEKLALRALLLFRACIKMVFNPVHTFKYQYPEDKEDRKSAVAQVKSQVFTHDFVVQLMEILLTQYFVLRPADLRDWEEEPDEWERREEEIADAWEFSIRSCSEKLFLDLVINFKELLVPRLLQVFQQYAKVDNHEVLLKDSLYSAIGIAAACIEDVVDFNGFIRTTLVPEVQMNQPNYNLLRRRTSIVLGQWVPIKPEQLDRVAIYQIFAHLLSPSEQLNDHVVRVTAGRQLKLILEPFEFSYSDFQPYATPILEHLMGLIRETELSETKMALLETVRVAVTKLEGQIEPYAQGIMSILPALWAESGEEHLMKQAILTMITAIIQSLGQKGSSYHEPIYPLIHDSVQPESEASVYLLEEALELWAALMAHAPSEQPSQQLLAMSKSLLPLLELGSEHLRQCFEILESYVMLSPATVLDAQVLTPLVASQKSMLSMLNTSRARDASLSPRVVQTIIQSLSVPGNLTAEAREAALQHVVTTMIQTEYLPSLLAILHEAYSYHQDPRPNRRPPDIMGVGETSLFTLLSHLILASPSLFISALHSLPRTEAEKDNLSWLLNEWFYQYDSTPDTLRRKTQLLALTSLLSLSPPPSPLLASLQSLFSLYADTLTELAEGAAEENRGDYLYAPAAAGEMLQHWPDSDSAEDVRKRHMTNWDAVYVINAREFVGEKLRQAIEACGGQQAFEQQWITDTVDQDVLRSFASLGIL

>CF317_000515-T1 CF317_000515

MEKHLPELDPQVAEIMKKEIQRQRESIVLIASENFTSRAVFDALGSPMSNKYSEGYPGARYYGGNQHIDAIELLCQARALKAFALDKEKWGVNVQCLSGSPANLQVYQAIMRPHDRLMGLDLPHGGHLSHGYQTPQKKISAVSTYFETFPYRVNLETGIIDYDRLEENALMYRPKVLVAGTSAYCRLIDYARMRQIADKVGAYLVVDMAHISGLIAAGVIPSPFEHADIVTTTTHKSLRGPRGAMIFFRKGVRSTDLKSGKQVMYDLEGPINFSVFPGHQGGPHNHTITALAVALKQADTQEFKDYQAQVIKNAKSLENEFKKLGVKLVADGTDSHMVLLDLRPNSLDGARVEAVLEQINIACNKNSIPGDKSALTPCGIRIGAPAMTSRGFSEHDFKRVAKYIVQSIDLCKKVQSELPKEANKLKDFKAKVADDSVPEILELRKEIADWACTFPLPV

>CF317_000516-T1 CF317_000516

MHKKYGKLVRIGPNDISVSDADGHPVDATNLLNLYSFDTMGDLAFGKDFGMLEQSEVHWAIKLLNEGMDPMGLQFPRWFFRLVLAIPGAAKGYWQFIDFCTKALENRIAVHDKTHFARSDITQTLIDHYQKSDNQKQMWPMLCGDTTAAALTHLFYHLADQWLKEAPVLNGLINETLRLNPPVPSGVFRKTPPEGVMIGQTFVPGNTNIQMPQYVMSRCKSSSVSLSLSLPSPYFPTSPQLPHVCVLSNTFTAPDNYAQPTSFMPERWYSRPETIKHKDAFAPFSTGPFGCIGKNLAYMEIRTITTQIIDLFDGSFAPGEDGTLWKAAIEDVCQQNGHKSVVLIGHSMGASIAALLASATSTVPTLSISVVGMIAICPRAHQLTPKEINAAQKLKSTPDFIINILRWFDRYGGENSTSVLRVVGSSNESDLKRTQLAWNRQYRTPVLRRITLGLLPHTASDGTVSGGYPNEAVWKGLNTPLFLIAGESDTMCKPAEIDLIVHHMTGTPTTEVEKSTQPEDEMQASTNGLVPTTIQPPTSPSTIQSITLPAPAAHALLYAHTTYRLVSALIESFLAHHVSPHLDFTYQLRLLTTTGKWDVKNLRKWKDVLPVSAPIDATPSNPNGVFRALKTMREQDDEHNPANFVRHWAATIYAVIDISHDAPVYDAKTLDAGGVEYHKFPTVSKVPPTPFEVQDFCSLVDRLLAERDASASATAGRANKHKALAVHCHYGYNRTGFFICSYLVLRRGYGVQDAVEEFARAKPPGIRHAHFLDQLWLRFAGREAGGARGAKRGDGGFGAAAKGKNDERGGSFHLTVDDGTHGQAQVQLEGGADAEAGNVSEGDVM

>CF317_000517-T1 CF317_000517

MDARYGSDGLGETDDDKIEIGIKQVQLEQDTAKTQEQDEETSLIDFNRSGHALIEIISLPHLHTPEAAAAYVRKVQSILYAVDAATTGMELGGLRADVNVSIRKLDDNTSNHSYSGVGGLGQRTEIKNLSSFKSVEDAIRAERDRQIDVLESGGRIEGETRGWSLSAPGVTRRLRGKEGEVDYRYMPDPDISPLYIHPEVVDHLREHLPPVPEQLLDMLCAKYGLTVVDAQALLALDNGSRLLWYQDVVARLKVLEDDPGSRIGVQAGNWVLHELGALLTTTETQWHPGLIDAQQMADLVSLQAQNRLTGPSAKRLLRVLFDGDKRSVAEIVKEENLLFMPMTEEEYDALADTVIEANPNEADIVRQNGKQAKGKLKFLLGQTMRQGDRNRIEAQRAERALQTKLLPVGA

>CF317_000518-T1 CF317_000518

MNSLIVASLTATTCARSLYSSYTFDPLQHLAGIAPYFESEDPPKSPSPPQGCNVTKAAYLVRHAAINANDFDYESYLEPFIDKLNNHTNINWSSTPELSFLSSWIPPQLPEQEQLTRTGKLEAAQLGVSLSYKYSNLRIPQRVWSSSAERTVKSAQSLIRGLETEDNQINLVQVYEGDEAGADSLTPYSSCPAYSSSTGSKQSSQYSKLYTAPILARLNAQAGGFNFTTDDVTAMFEMCGYETVIRGSSPFCSLDLFTPDEWLSFEYANDIMYFYNTGYGNPLSGVIGFPWLNSTMNLLTSPSSSSDQDLYISFTHRELPPTVLVAMGLFNNTQFSSNAAINSTMPLTQINYNRAWISSHFLPFLANIAIEKMDCSANYYVSQTQKQSSSSSSGGDDTYYRVLVNNAPQTLPGCYDGPAESCSSGGLKSWLAGRAAMFEGFGQRCDVDYGNSTDQVSFYTDGAFGNGTAVGKR

>CF317_000519-T1 CF317_000519

MPSLEGNGHSNGHTYGSNGTSAKKPVQTESFAPTSSIAQKFLSNPSELGVTLVGFSGGQCRPGTDAGPAVLTQAGLLTQLESDLGYTLHGDTSVRNFNDYQPTVTARDGTVSNSSPKFDDPDVRGMKNPRSVSAVTEQISDLVYQQARAGRLALTLGGDHSIAIGTVSGVARAIRERFPDNANNEIAVIWVDAHADINTPETSDSGNIHGMPVSFLSGLATSTEKGVFDWIQDAHRINTKKLVYIGLRDIDAGEKKILREHKVKAFSMHDIDRHGIGKVMDQALEYIGADTPIHLSFDVDALDPMWAPSTGTPVRGGLTLREGDFICEAVAESGQCVAVDLVEVNPRLVEGGVGDTVRAGISLVRCALGDTLL

>CF317_000520-T1 CF317_000520

MRLDDNKAFLPSVHNGPHSRSAVDIFAPSSPRAFKSGTQQVAEIPHELQHKPVPFVGFWSLQDYEKAHEEVHEEHDEQSYEQDPQNALDRHPASAAGTEHQALNFSGSETFRRRWHASRPKKIDLSYYNPITLELSKQYPTKRQGESAPAFRASAESSGSRQITKYLLEHDVYSHVSPEHGLPDNLAIGLLHRIASFEFHRGPIDIDEAFSCSWILSADTVTSIKRLVALTRHRHHMNLPPLPQWIIMQVLRSSRIDAKSLTDLIVLVKDRHTSWTWMEDQPMLLAVRLLRHARRSAAPCFDAIVDLFLLLVRPRRSTPRAVKKMAHWCNRILSLIAIPVSHSPFRSMYAQQVAQLALVRYMQDSEPQLPLMREGYRAIAKLQLMHEKTPQETEWASAKARTWPPWQEQNRMASVAGPSHYPGKRTRVRRVLERMQESGYAPTNYDLAVRIMTGWDTDDSPTTQVRRTAASISIPQPWEPGPGLDTVVYSPLIWSARVEATRTLREAWMCFSSYQAAASENPPSERVYHALFRKLFARTVSYSPDGPWPGDGTELYPDPDLARDRVYIPEEIPSIRGLFTKMLAQDLRPSTRLLSDLLKHETNLLQGLHYVSYAAIEEEKKQILASPWNHPSEHVARVLMALPSNLVQSYVGFLARPHPRVSAHERRPVFDGRSGPAYAKCLLDQMRLRDISVLNVYLYSLTCHVGPQQLPFATHHKDPVHVIWTLACQAISNLTMATPVDFTTFTHAAMIAYSVESSSWDGAEHARDADEPPTLVAKRLFQTAAGAYNASHPISWQEFCRADRTMRSLPPGAAIEDMVWVLGSTHGYKVAGDVLALLHWVRYHQERILAAGHHITKHNLAVFRFFLEARWADEEDWQTLQEGRHFMNSTQRQELQGIAEELGAWPDDRYMEKYMCIQQGRANRLRRKYKK

>CF317_000521-T1 CF317_000521

MNGIVKEQHRSPTPSSHLGKRKRSASPAKVLANGSASLNPLRAHSNIDNIIREVKQYAIATSILEQPLPHPISDAPSIKKQRTDIKHDTVEGRLALEAYARSDDLLADLRQAIANMKSHSPQVNGHNEKSEASQLLSIESILDEYAIANSARTAGDGPAFGGQVMTVRSVVEGGVPKQLFTGLRIQPANHVKPELIDARTLPSGLDMVEAMPVEPSRNPSTKHSRTFGHVFRQIRNTRHLEPPPSAPATQATPLRFEMPFESPTNINKDDYRLAALTAGSWLEYATSTSEPPRPPTQPARSFKQDPHALFNASFSSFAPSEDNTNAIVPRAQRSRLWYKKHGTHAWRRIMTNNTQIEAPSASVYPDIDDTYQQLIEDFEPDTAEDANPQPPERATDETEVLDEVSELLQTLSSYHKIRDLDKTRVHSAQVKPTAPEIDIFELLRQQLSVLVATLPPFAVAKLDGDQLKDLGISTNILIRAPEIAGVGQPDEGTLHRQREALSQQAAMARPVNPPAVRNSYTSTASAPSYNSQARSYSGNASQTPSMPGYAQRNAQMYNTPRAHVPASTAAYSQTPSYQRPTQPFPGATIQQYQRMQNGYGQNMQTPYQQRPTQAPYQAQGQMQNSMQYGRSASPAKPVVNGQGYKAPTAQQQAAQTQRSQYSTPAAAASLLQGPYAQANSHATIQQIKAAQQAGQMQVQPSQSQSPQPQAMQGVQHSNAMAVQAQSQRQASGTPQPASQSQTPQQQHASVAAIPASPAASNALSAVPGQIGQAVQASQQQAQRATSTPTPVAAGAGGA

>CF317_000522-T1 CF317_000522

MQRLLEGWLGPCPQQQMPQHPIPHVGGGYLNVHGPIGDVVQAHAQAQTQHMSPDTTRIGQGGARNLSDLTGQRVTEPMPHLLAAQESSDSRDSFVTSATSPLPPAAVQDTVEADSGLHVDDNAVVVDSQGRPYALQVSDSPTQPQRQQCPKLLHADRQATAAYEASQLLAIRDVPTQTRSSPSTQRLTRSQSLFAADAPAQQQAQGPVIGHGPMGPVYLNGNSPRRRRSQFGVHEDTTSVAPGQGDSQSRSRTTRRSSRKRGRRASAEGDARQASAGDGRFVFAEITTKSQV

>CF317_000523-T1 CF317_000523

MPLVASSSLRPLVSQPGRWAIPAHLLAGQRRGGYRFISVQQTPSTSETRLDINANSISASSPDATFEVLGSPYSLLSVTLSPSQPLHTRRGSLVGLSGDPTTVVSTLRTLNPIRRALVGIPFLYQRVTSTTPVSVLITPKSTNTTLSVLQLDGTQDWKLAQRASLLAWTGSNLSVTPTISSKLSLAHWGTSNATGRGLLALSATGHTFALDLAKGESYIAHPSNILAYTTTNNPPPQPFRFRSSQARFQIPLQLGNWFPDSRFTRAWKTSNTYKFLQAISLRIKTFSRRTVWGDRLFLKFEGPATILLQSKANRVSDILTSDQINEIADAPAGVVDKTIRQARIAQEDPYFRATSGGATRADTEGRRLSSKSDADRMGVQGGTPSAQVSKAAERDAPQVVAAEGGPNSRIPAVQELEKVARESGQAEYKVKEEETVRVEAKAAA

>CF317_000524-T1 CF317_000524

MNGNIDASHPYPTPPRTPFTGSQDANNDSPISVIASSRVVRANLLTAGTIVISNETGKITAVFDSVLPPSEFPEGTPYRDYSPHVILPGLVDAHVHLNEPGRTEWEGFYTGTQAAAFGGVTTVIDMPLNAIPPTTTVANLHTKIEAAQGKCWVDVGFYGGIIPGNASELRALVREGVRGFKGFLIDSGVEEFPAVSSADIEQVFAELADEPTTVMFHAEMLPPIADSVGDDVQKSLPPLAPHGPLNAYQTFLQSRPPAFETYAIEEILSLAHLAPNLPLHIVHLSAMEGIPLLRKARAQGINITAETCPHYLSLAAEQVLDGDTRHKCCPPIRNQTNQDRLWTELLDHANEGVIKTVVSDHSPCTPDLKLLPSHVPGHCTPTAGTVKDLMLDASSGDFFSAWGGISSVGLGLPILWTEMSRRGLTGSSSVPPQPNDPSTPSTAPTSPNTDTDTAATDQALTTLVQWCCTNTAAQVGLSASKGRIATDMDADFCIFDDTAQWTVEPSTMLFRNKCSPYQGKTMRGMVRETWVRGRVVFRQTAENGGFVAAKPAGKLLLEPRVKELKKVRSWFSKWIGQAFGY

>CF317_000525-T1 CF317_000525

MSEETALKDFLRKQARSLDSLLELTPAKNMFGEDVSDQWQRKKQTPEQKKAARMAKLDPANWKSAKDVYDERSAEAAKKRKREQEGNGESQDSTTLQEQPVKKLKTADQNAQPQPKPKPTEKLTERLESDKLQNVNLKKKMKKRAHRAEKKQHKEEQPEDLASEEPVQTTQIIPDKKPSPLEQKKPSKRELKGIKEQTDSPSQVVTKAKGKAKEALTPAARVQDKPQKIEKKGEASVDEDGDVNGVEDAWSDVDEEANNDDEDEEMADADSTATSSEGVPEVLSPPHDSASSSVSSVVPLAQTQTAKAAQNPDTKVEESASAQPMDTVQTEAERQAARQRLQEQISQFRSQRKADDKPVRSRAELLAQRRQKEQERKAAKKEQRKKEKEEEAKLQDEEMARRFSPGGSGSLLASPRSPMIDDGGSNAFTFGRIAFDDGTQFDAATGSAAASKKHKGPADPATALKAAQAKQARVAALDDQKKQSIEEKDMWLNAKKRAHGERVKDDTSLLKKALKRQEKQKQRSGKDWDTRLEGVRSSQEARQKKRTENLQKRKDEKGSKKTGKKKVKRPGFEGSFKGRAGKSKKS

>CF317_000526-T1 CF317_000526

MATQPLSKKDRHNPWTAVQQSMLQPAQAAAVAQVDPGRPTRQEALLHETDLVTRDAPEDELPPPVYGDIYGEMRNDEDGLSTSARVTDDGRVNIRINQLNRRLSQIFTPALRQQVQSVQDCRPHLPPYAPPSLRGEEGVTPPPPLNIVIQVVGSRGDVQPFVALGKVLKDTYGHRVRLATHPNFKDFIQESGLEFFSIGGDPSRLMAFMVKNPSLMPGFRTLLSGEIGQRRRDVAEYIQGCWRSCYEAGDGMGHGATDDDLSEPAARPFVADCIIANPPSFAHIHCAEKLGIPLHIMFTMPYSPTQEFPHPLANIQSSNADPQLTNYISYIMIEVLSWQGLGDIINRFRAKCLGLDPVSLIWAPGMLQRLEIPHTYCWSPALIPKPKDWGPHVSISGFYFLNLASNYTPTPDLQAFLDAGPSPVYIGFGSIVLDDPDAMTELIFEVVRKTGHRVLLSKGWGGMGADRPHIPDGVFMLGNVPHDWLFEHVSCIVHHGGAGTTAAGIAAGRPTVVVPFFGDQPFWGAMVARAGAGPDPIPHKQLTADKLADAVDFCLKPESLERAKELASKIAAERGSDMGAQSFHQHLEADRLRCTLAPSRPAVWRIKRTQVRLSAFATCTLANANLLDFHDLKLYRSQEHYIDEGPWDPISGGAAACFRAFSGMAMGLAEVPSETLRSLRIPTGSSRQQAQESVPNITRKRETSQMGERSTTPTSPEQSQTSLNLQGSLTRISGQLSSKQDDSSRSRVSSRNESGFGKDHDMLRQTGVHTSKGLGRFMKAFVQTPMEISESLTKGFHNLPKLWGDDTVRPHEQVSDFKSGAMAAGKEFGLGWYDGVTGLVTQPWKGAQKEGASGFVRGIGKGIAGFATKPFAGFSGILSHTMKGVQKEVQKLFGSHVQNYIVASRIAQGYEEWLQSSDAEKQDVIVRWKLIQKYLKKKGNPDEMVQDVLEAQRKKNVEDTMARQNCGHATSSAQSASADAPALDSESAMLAVRGSQALEEASEAAEINEIRPAVQKTSRRDVEEDADMKRAIQENVPQLQRQQQEAADHQAEQENLRQATVASEVEAQRQASEALEFKKQLERVLAQSVLEQRQGGSGREWESDIASP

>CF317_000527-T1 CF317_000527

MLQTRREERVQRQQEQDAAAQAEAAEMAEAIAQIEEMERQEDIRLAEEERQRQLEEELMLARLEETRLLEEIAKREAEEETEKIFRQILLNSCEEECQALMRDLIQIINFQHAALMSEHEARQQSCIQEKAKQQATALEESSTMASWLQENISKRRQTLHTKQREEWEHILRQAEDEEDDMFMQMQMYLRDKPNREQRVKRMQDIFQQQQQEMQASLQFKHDDERRSLEVSIQYESEGLQQAQRTRLESIEEQFESSLRQLGWHIGCNRQWFQLVSTRRIEMLKECRRMVLKQLEAEQDPVGLSEEQAKHIGPTLPAIQEGRTESAGVDSINRQDPNQLIHCEPPQLPSLPPPPPETADLYGPDERDAPDNAAASVQQTRPPLAPQNLPVRDGDPQLGRSDTIVRPIPGAYPTSTTVAPATSKRSRGHYLDAFGPPAIRSAPAVTKRKPLPWRDSTGVSPVSTGEEMCTAPRKKGESTSLSTPPSTRQGSATTNDSRRTSQPWTWTSLADGQTDAASRACTMEAGLGGRVSTRADDSADKKGRGFFSKFSRRRELGEEEMRRKMSECVGDGFGV

>CF317_000528-T1 CF317_000528

MRDLVLRRTLAACQHGTRPVYLTLHLHLHQHHSTFQQRRQQSNHTTAIDPPRISAEPRYAIKGLVPNLDTTRPAALDLPEAPHPYAWPSSQSKNPTRWDFKYLFRLGKTYGAFYWQGMKNVYANYKTRQKIVRRLNGTPPDMAARNPASPQRISYNEYETLIRFKRDLKKLVPFALVLAICGEFTPLVILMLGSGVVPATCVIPKQVTQDRKKTLERESTYIDLMVDTWSKRLASIQRSDKQPESKSKTHTYLLPDAYRLHLTPFINLTGLTVDLYWRYRLAPRLRTHSNEILSTAVLVLREGGWSKRSPLDMWEWGNKYGMYRLRQYTRDAIDRGEDPVSEDMKTILLRHFDQETKAIVNAAQRNGKLDYEHHDPLVSDRPDGLAMRKDIERMVKEREREQEQEAKDKDKAKKT

>CF317_000529-T1 CF317_000529

MRGIALAGPALLGLLPSVLADAASSINVALKASFPSAPYLIELIETAALENATSYFPLLDRVADGAFSHAHTEQELYDSFIHISHQDGHFRGPGSLSSFKLALSIHAAAPRIEAHYQYYNSTLAPSMMAAQDAVCPVWVHLDGQQYCSPELQRPQQPVDNDNLELELPFDRTLGPSDAHVVSTLYADITHPLFAQFHRTVSKTAREGKTAYRVRHRPSQQHSDRPLHVSGYGVELVLKRTDYIVIDDRDAAPANSDQSENDVKETATADLKPLTSAEVSKLGISAAAYIMDTEDPFSTLLDLSANFPKYSSLVAGRNVSDHFLSEHRKNRELLLPGGYNAMWMNGLQIQHREVNAYSLLDTLRKERKLISRLRDIGLSSTEAVDLLSHQVLAQAQSDDQPQRYDWRSDGDRDDIIIWLNDIEKDARYSRWSKSLNSLLQRTYPGQLPQVRRDIHNVIVPIDFHNIKDLDLVVNTLQTLIKRAFPIRIGLVPANEEAASQDAAKLSYHILDTFGLTSLIKFYQQLVVSKKGLGAVQTIFESVTKDMPARDGKESHAYADVLKDQGMQQRLATVEAYRARLSLRGKTPPVLVNGVPIPRSDNWFEVLSQRIYNDHRALQVAVYEREVDENAWVPEVFLQKSSSRRNALVIPESSKDITVANVAELVADFASSFIHLPRIRGEESSLLSSKAHMLLVLDVDDADGRALLKEALAFHEKHPEVEVVICHSQKPGEPLSGVATELYHMAGGAAEGITRESVQSLLQSEGGSVPQTSGDQEKAEDFWLSHEDLIRRLGLNPGDNGLWLNGRIVGPISSTFVAEDFESLLSYEVTERIAPVTTAITALALEDKFSEPIDIAKVTAIVARSLKSDIPEGIYETAQLIRLDRFKQWKNESTMIHTKSDAEPTIQIVAAIDPASEVAQSWTPLLKTLSEMDEVDVKIFLNPREMLQELPVKRFYRNVISSAPAFQANGSLAVPQATFSAIPEQVLFNLGLVVPPAWLVSPEESIYDLDNIKLSSVPVGQNVDALYELEHILVEGHSRDATKGPPPRGVQLLLGTEQNPHTTDTIIMANLGYFQFKANPGHWQISLKPGRSSKIFNIDSVGSKGYAAQPGDEGNLVTLMSFQGATLFPRLSRKAGMEDEDVLESTGLADTAASYLKKGQSLLSSFGLGGKKSSASTQAEINIFSVASGHLYERMLNIMMLSVIKHTSHSVKFWFIEQFLSPSFKRSLPYLAEHYGFQYEMVTYKWPHWLRGQKEKQREIWGYKILFLDVLFPVDLDKVIFVDADQIVRTDMMELNKVDLHGAPYGFTPMCDSRTEMEGFRFWKQGYWANYLEGKPYHISALYVVDLKRFRELAAGDRLRGQYHALSADPNSLSNLDQDLPNHMQHNLPIHSLDQNWLWCETWCSDEALKSARTIDLCNNPQTKEPKLDRARRQVPEWTEYDDEIAAVLRTAMKDDEKVDLTAGSERQNDDLFEKHYVKDEL

>CF317_000530-T1 CF317_000530

MAKLVKKKSAQPNGTPSQQSQPWNPPVAALATPSSQKLMPPPARPARLSNPVATPNGQQLHSSDAPSEGSSSAKKKRRRRGGAKPGRNSFSDNATTTSDATTTSNTNRSLSNPDSDRAVPPSLTHLRPTMSQEEQIVYEKEPEMFGDYALSLAGDASSHLDTDNEETTARPDKDQTLPTLANETTKATEHAIEDDATSDTHPSEANGHPTKLDKPRPAADLAAADPIAIPAPTKLPDGRFECPFVCGKTFSESKGAKRHAALHVNADKFKCTICGKGYSRNDLYQSHIKSHPGEKAILSKQQARSAPTLKPITNAASREKVSLPRPLHMSNHGHVNPGDDSRRKKHQSKTAPEASAPVSDPATSAESETESEDESAPDDRTTDTPTSPVEAKADAPQEHASGDETTCTSESESEASPANSRAAPPPEPTLHTDGLKRKRPLPSSPSPRALPSRKRARQQDISGSPEVEESEQAESPGPDIKSSKAPVLVDDASSDASDSDVEMHERGGNRAGSSQSAEEPDAEPVKRLVLPKPKGKASEAASRKKSTKQVLDTLTPDLAGPSSTTSARQAKASPPKPAVRRQSSMDDFVSRSRSISSLSARPSSSDSAKVTVNVPPRQKVKPQVQVKEQSFPKQRLKTSKSAPSQVQPQQDDEEHSVVEDSEEQRTTRRKTKEGKGKGKGKAREDINREVADESETHSSDRPKATTTARRTSSLTSKPKPKPKPKRKSKMAARLQDEATSGDQPGVQDEAASDTDTDTVKTKKKKPRNTSAQTSTSGSGRTGKFSEEEVQTLLSWRDSFCSEHDLTPTEFNNMMTASMRRTGGYQWPYKFITRTDFLNEYREQLPNRDKRSMNRFRERHFQNVESTTWTKQDDDELRGLVNQLGPKWVEIAQMMGRTADAVTQRWKNKLNYGEEVKEGEWSEEEVKRLEEEVEAYAESKGTTARDDSLNIPWHAISLKIGNGRSGQQCSNRWRVNTTRRIKGKFTKVPLEDRIPGRATKLPKSEAKVTKAPKKAKALRTPSKLSQRLAGDDGTPKTEASQERSFVNKSFKSSAYVTNSDEEGDEETEEEEQESHESDDEEQATEAKADTNMDSAAESNEDTAPEDDQAETDSNDLEQPRARTSDTDVAKTEQDMGEDTEMRDAEDADEDQGQESGQDQVATNASNTEPVSDNISNTIEVRSPTPPPPQSSGSRTQASQPQSQQRGKKNQSSPQSSSQAQSQSQSQPQTQKSSQTQTQKSTRTPAISKNPLRAAATTKTPANDLNFGLSLSQLHAGTQANSSARRTTTGKSTGARSRVPDSAIEERPSPELSVRRRPVSSPLGRMLVGDEDEDEDEDEDEDEDESGEARGAHDDTIEVRSPQDDSFVSARSDTRARSPTPSTSSSGSDSDSDSDSDSASSSASGSSSDAASDSGAESNRASDERERRRKASRTGSGSQGSFWQSVNGIAARFVPGMSQNQSSQAQSQSQSQGQGQGQGQTGSKRRTLADALMQGRVDEESDTDDE

>CF317_000531-T1 CF317_000531

MVLLTTTPTILAAISSLPSDSLPSQSQSLPTSTDSPVSHTTLLTLSACLRKSSPSYTLNTLLRGAKPYIAPPPPKPEPSDEYKALMARLRADQERREYRAMVAKQEAESLQALSGRIGTDGDEEEGKDDISPSLVLNIVLSIVMCAGVAFHLTRWWPNDGVRVLVSLATAVVVGVAEVTVYAAYLRKVEESKRPRDDGDADVWTGNAGEVEKTEIWGKGVHGGMRRRLREKWEKEQEQEQRKAS

>CF317_000532-T1 CF317_000532

MDANVQRLLTDKLYDKRKTGALELEKLIRDSLSKQDHDRIRKIVDQLCHEYAYAVHQPHARNGGLIGLAAASIALGSDEVARYLPEIVPPVLACFTDQDARVRYYACEAMYNISKVAKGEVLVFFNLIFDALCKVMTLPAERYIEVKKLKEKQLSGDSELSVKNGAELLDRLIKDIVSESAAHYVSILNFDDRKQEKDRDGRASQQSSNDMKPAFSLADFIPLLEERIHVLNPFTRQFLVAWLSLLDTIPDLELVHYLPSFLGGLLKFLGDQNRDVYVATQGLLERLLAEIVKIAKVKKGITESRRRRQSESRRRDSESQAPTKSPDGSTEGEDAVADGSASDESGYEDSSGDWIPGQDVEIDHAAILDILLKFVDPSSNKDSSSVLRLDGLPKQKEEEIGVVALRWIATFFEVASDDILAFVPRLLGQVLPALASSSEQFRAQAVSVNRALIDYVVAYQDENEASVAQDASPRDVNASSAPTSAHAHQKSIASIAESETALPVPQETTHKQKTNEESQEVGATDSDPSKLGLDYTEAVRVLQLQLLHEREETRVATISWLRLLHRKAPSKVLALNDGTFPVLLKTLSDPAEAVVTQDLQLLSQLSKNSEDNYFSSFMVALLQLFATDRKLLEIRGNLIIRQLCINLKPERIYRTFADCIEKDEDIEFSSIMVQNLNNNLITAPELAELRKRLRNLDTKDGQAFFVALFKAWSHNAVATFSLCLLAQAYEQAYSLLQIFADLEMTVNMLIQIDKLVQLLESPVFTFLRIQLLEPDKYSYLYKCLYGLLMLLPQSSAFAALKNRLNSVSAIALLHTPPPMPTSARPSVAAGSNIQGSSTVTVGRFPGRRETGGINTTGEVKWNELLDKFKGTQEKTRRRNERLLRGEDEFETEMQEREANIADASQEQTGRTSGANGRQSFELRRPSSRLSIEQGRAGPGLRQPLSAGANAGQPRSSFRTERPFDGPGGSEPTRPGNKSKYSLTGRFGIRSSSKDKEKDRSGGSGMGSSGGSGQKR

>CF317_000533-T1 CF317_000533

MTSLLKKPALDPRSTTILVTGASGHIGGHVVNEALKLGYKVRGTARTQEKCNITQKAYNNPNYSAVVVSDFGKPSQEIEDAVKGVDSVIMVASDTTFNDDPEQVIGSVVAGVEAFLRAAAKEESVKRFTLTSSSTAALVPRPGVELTVETDTWDDEAVEVAWKRRGQSIGVNSYPFVVYAASKTEGERAMWKFLEREKPGFVCNSVLPNTNIGKVLEGGSTGATGAVMINLFETGKKTELIVPQYYIDVIDDARLHLCAAVLDDTLERERIFAFAGPFNWNMAVDAIGRVRPDCKAKLEKDPNEQEDLSKVPNELGAQLLKKWYGQEGYKSFDQSLKENLEHL

>CF317_000534-T1 CF317_000534

MSADFCASYASGAIGICIGNRLDIVKTELQAGNRIFTSPALPAQEAGFKKFSNLFRGAAAPILGYGALNSILFMTYNRSVKLMEPSVFDPTKLAGVDLDKIWLAGAVGGLACWVVSAPSELIKCRTQLKIDGGASTYATTMNVLKQQGIRGLYQGGTITSIRDSFGYAWYFWTYELSKRLLLSRQSNPFAEPRASDVLIAGGIAGVVTWASVFPLDVVKTRVQTQSQVVDELTAPLLGQEQARAQQKTSFQIARDIFRAEGIVGFYRGLGVCSLRAFIVNAVQWYLYEETMKVLSPMLDKGAR

>CF317_000535-T1 CF317_000535

MLAIRTKVIDVNANKENLPPSARAGRLTANSTAALDRLSKPFKCPGSHNARKSTGEPTRKRRKVNYSEEAGAGDDKDEGYADGNTVVAVKKYPVFQVKDRDTIFRARFAVPLADKTSGGWDPSRPAPSLGMRRGNGFVNKPLHDPSGEFAIVLYDPTIDEKPDPAREAEKPETEKPKLDEPIMHKSLAEILGIKKQVEGEAPKVPVVIDPRLAKVLRPHQVEGVKFLYRCATGLIDENARGCIMADEMGLGKTLQCITLMWTLLKQSPNAGKGTIQKCVIACPASLVKNWANELVKWLGPTAPTPFAIDGKASKEELTAQLRQWAMASGRQVTRPVLIVSYESLRLNIDELKDVKIGLLLCDEGHRLKNADSNTYVAITGLNVDRRVILSGTPIQNDLTEYYALLDFANPGYLGTKADFRKKFELPILRGRDAGGSDTDKQKGIAANSELSGLVKKFIIRRTNDILSKYLPRKYEHVVFCNLSPFQMALYNHFIQSPDIKSLLRGKGSQPLKAIGILKKLCNHPDLLDLSTDLPGSENCWPDSYVPKEARGRDRDVNPFFSGKFMVLERMLARIRQDTNDKIVLISNYTQTLDVFEKLCRARSYGCLRLDGTMNVNKRQKLVDKFNDPEGEEFVFLLSSKAGGCGINLIGANRLILFDPDWNPAADQQALARVWRDGQKKDCFVYRFMGTGTIEEKIFQRQSHKQALSSTVVDSAEDVERHFTAESLRELFQFKPSTTSDTHDTFKCKRCKPDGTQTIKAPAMLYGDTSSWNHFVNTGEKGQLGKIQDLLLRQEASEDAVSAVFQYISH

>CF317_000536-T1 CF317_000536

MLSSKLFGRVCSQGARMGSVSNSRQHITRAEIANKSKPLTSRATYASVSNPPLDQKVEMTNWEKGHYINYKKMSENLAIVRKRLNRPLTYGEKILYSHLDDPEGQDIERGASYLRLRPDRVACQDATAQMAILQFMSAGMPSVATPTTVHCDHLIEAQIGGEKDLDRAKSINKEVYDFLSSSCAKYNIGFWRPGSGIIHQIVLENYAFPGGLMIGTDSHTPNAGGGLGMVAIGVGGADAVDVMANLPWELKAPKYIGVKLTGELSGWTAPKDIILKVAGILTVKGGTGAIVEYHGPGTNTLSCTGMGTICNMGAEIGATTSLFPFNDRMHDYLAATKRRHIGDFARQYAHELHEDEGAEYDQLIEINLSELEPHINGPFTPDLATPISKFKHAVKDNKWPEDLKVGLIGSCTNSSYEDMTRAASIAEDAMAHGIKSKSMFTVTPGSEQVRATIERDGLLKTFEEFGGIVLANACGPCIGQWDRKDVKKGEPNSILSSYNRNFTGRNDANPATHSFVTSPDLVVALSIAGDLTFNPLTDKLKDKDGNEFMLKAPTGDGLPKNGFDPGQDTYQAPPEDRSSVEVQVSPSSDRLQILQPFDAWNGKDAKDLPILIKTQGKTTTDHISMAGPWLKYRGHLDNISNNMLIGAINAANGEANKVKNFKTGEVDAVPATARQYKKEGIKWVVVGDWNYGEGSSREHAALEPRHLGGLAIITRSFARIHETNLKKQGMLPLTFSNPEDYDKINPDDKVDLLCTQLEVGKPMTMVVHPKDGEAWECELSHTYNAPQIEWFQNGSALNTMAKKAGTTQ

>CF317_000537-T1 CF317_000537

MGSIRRIKTKRLTRGLDQVKADLANPRHLTKYKSTKAAEDLPGLGEFYCIECAKWFEGEHNLAGHRKGKNHKRRVKELKAEAHTQKSADAAVGLTTDTGRQAEPMVEG

>CF317_000538-T1 CF317_000538

MGLTGITTKLPHMPKTYNTYLVAFVATVGGMLFGFDISSISAIILSDQYLDFFNTPSGIEQGAIGASLAAGSVIGSLMSGPISNKIGRRDAIFFGGLWWMVGTAVQASVTSVGMLICGRILNGVCVGITSSQVPVYLAEIAKKEKRGSLIIIQQLAIEWGILIMYFIGYGCTFIPGPSSFRTAWGIQFVPCVLMLCGLPFLPRSPRWLAKVDRHEEAIDILSRIQANGDRNDPRVVAEWEDIIQTLALERQAPKSWRKFVYNGMWKRTLAGFTVQMWQQNSGANVMTYYVVYIFSMAGLSGNINLIASGVQYALFIIFTTVMFFYIDKTGRRGLLIYGALAMGFCHFVVGGLLSTGKDVPGGVDGNPNVPVQLTGGKANTVIAFSYLLIIVYALTLAPVAWIYAAEVWSLETRAVGMSIAALGNWLFNFALGLYIPPGFKNIKYGMFIVFGIMCVLAAIQFYFTYPETGGKTLEEIEILFSPDGPRPWQTKFGDSRLDGLVAEARTKRYSVDDVNAGRVGSITDIQGDGNKNVGAAQNIEKV

>CF317_000540-T1 CF317_000540

MRTPTTTTAALRASRPAFRATPQKRNLQDIAITRTGKPIIRGQGGRSSLGGHTVTVFGATGFLGRYVVNRFARAGCQVIVPYREEMAKRHLKVTGDLGRVSFLEYDLRNTQSLEESVRHSDIVFNLVGRTYPTKNFSLFDVHVEGTERIVEAVAKYDVDRYIHMSSYNADENSPAEFFRTKAQGEKVARQLFPETTIVRPAPCFGFEDKLLHKLAGQTNLFTVNNMMERFNPVHAIDIGAALEKIGYDDTTAGETFELFGPTNYSMAEIATIVDREIINRRRHINVPKRLLAPVLNVLNKLVWWPVGSPEQLDMEMLDQVVDKNAKTFADLGMEPAELQDLTFHYLQDYRASTYYELPPSTAREKRDEKKYLHVLDDQ

>CF317_000541-T1 CF317_000541

MSKQKFDAEEISNFLTEQRDEASAEVQPVFLDLEDLWERKLWHQLTEKLLAFFASKDSNGLRLPLFNKFILSFADKINQLKLVKLALAASHEIEEDEDRAEFLETLQKRVNKSEFEEAHTYATTELASVYLGLDEEQKAREQLDTAQTTLDRFDFVENIVHASFYRVNAEYYQTREDFTSYYRNSLLYLACVDASELSPKERQQRAYNLALAALVSDQIYNFGELLLHPILDSLQPPHTQSWLRDLLFAFNRGDLATFDRLSNNLSKNKILDEHKVFLYQKISLAALTELVFKRPPHDRAMTFQTISQETKVKPNEIEHLLMKALSLGLLRGKIDQVDEVARINWVQPKVLERSQIEGMRQRLKEWDSNVNDLGHWIEDQGKDVWAS

>CF317_000542-T1 CF317_000542

MNASISLSRTCVQHCSRAKAPQKRLLNHQIQTRLISSTPTQHYAQHTDLPSETSAESQAFTSHQPDLDVPSTPSTRRNLRRPVPSTRPELQNHPAPTYMYTGTITRTGTMAQTVQITRNAQTFDKFLQKHYTRPLRLKAHDPHPAGYLREGDIVEYGAYTQAEKDTKLAKDRERVERETERLSAQDSSGKAVRKFQRTESTRAKMKGKKVRGVQFVVRRVVTPFGMGLDERMERLAVQDGNVQERASEGGQDSLLRGAGGNLSRQSGRASVAG

>CF317_000543-T1 CF317_000543

MPQIPDSPNEEHQRLLTVANRLPITIKKKNGEYSYGISSGGLVTGLSGLPKSYESLWYGWPGTQVPKEDVAKVEEEMMKEQGGVPLWIDDELMDMHYNGFSNGILWPLFHYHSNDMSFEEEPWEAYVEVNRQFARRIAQDVRTNDIVWVHDYHLMLLPQMLREELAAGNATNVKIGFFLHTPFPSSQEIMKLPYRESILEGLLHADLVGFHTYDYARHFLSACGKLLDLKTSPNGVTFHEKAVHVGAFPIGINPEKELGFMKKENVIQRVTGLRERQYKDKTVIISVDRLDYIKGLPHKFHAYDMFLEHNPDQVGKTVLVQIAVPSRQDVKEYQVLRSHISELAGRINGKYGNVDYQPLIYCYTSVPPDELIALYALADICLITSTRDGMNLVSYEYVTCQKDNHGSLVMSEFAGAAQSMKGCIMINPWDTGEIMDGLRKAVDMGPQQREENWKRMNDYVMKFTSAYWGTSFIEELMRIGSNHIQEVRMRHPSDESPISTPGSRCGNGGTNGTSTPDGVPSMSPGGAQEALPPPMVDGNVPAASGADDKPLDST

>CF317_000544-T1 CF317_000544

MADMDLPSDDLQYDFDSSSHPYYQSQNWALAIDSMDPMASDPRRNISPIHTSMHGLRQTTMAEHPMMGDWQFQQIQQQQQQQAQQQMHHQHQHQEQPHLHHQQPSHAHLHHQQQYVPHHSHAVSHHSTQHPADYRQQQQQHMDPAFGGVFHSMSMPTQMAMIPINQSALPVSLPLDQPYMTMPPQMDAMHGWADLHSELIRYQTTDIGPVALSPYQQHTASPTGSHLEILSQPSTSDEHGWTFVNGRTSFESSERSFFVDPTHTLHDRSLSESSYSDLDQAPQTAFLSNLDMGAAQAVYSPTSLSDSDWESYHHHHRHSIDHGSSVNPNALVRSLPIPATSKPSSPVRSPTLQGQAGSPGRKPGKKSPIVAKGTERVTKKTSQGGKEEKRVGKRKGPLKPEQRKQASEIRKLRACLRCKFLKKTCDKGEPCAGCQPSHARLWQVPCTRIDIKEIGYFLKDKKFDYERHVSLGFSVGNIKGFADAEKTLFVTHGYGHVLPVTAREVYVRDDKCLKMDWVEASKTAAGYEVSTAKMSAGTEGISTVMLSAYLDSHIDGGNTFVKFVDDYFEGTPFLSQMLKAAYNFYTKTNSKTIKKALKLMLAYNLTLHITMVEGMPVEESFVGKIEDKSSKFYGKTMAPVMINFQIKCAMADMWRELHKDVLEELSQLYSSVYSGDKLKNWPTIFMLASVLLAVWECMQFDCHYRVPDEAAVDKFCSDMETTPVGVIVGLFSAISQKLPSILEWDTSKHHALLGSNWDVCNTMTEVREHVTQYENYLRDRPNAKFDSKDFDSLSNKFLSRLVLRDIRAN

>CF317_000545-T1 CF317_000545

MAQSDDDFEPFMAVPPHLCQWPPIQDQWSTHTSELQAQTITECLPLLTAVNSPTSNPFDFDEHGLPSLHRQAHVEFVHNGLEPLPAPFVAMDASRPWLMYWSLLSLHIMGEDVTAFRSRIVKTFLPLQNVSGGFGGGQGHLSHLAGTYAVILALAMVGGDEAYGLVDRTAMWYWLGRLKQAGGGFRVCEDGEEDVRGAYCALVAITLLDLPWQLPDDAPARAEGLTDFRDGLGAYFSRCQTYEGGISAGPSNEAHGAYAFCALACLCIMSPPRESITKHLDVKALVRWLSARQYAPEGGFAGRTNKVVDGCYSHWIGGCWPLVEAALQGSIESSLSLSDIPAERLFSGEGLTRYILGCCQAPSGGLRDKPSKRPDAYHTCYNLAGLSMSKHRHEYSDVPHLTTNLVAWTVKRNDLFPDAAEDGAPSESEVAPFHPVYVIPYAAVTQISQWSRSNHVTFAS

>CF317_000546-T1 CF317_000546

MKSLGRTGLLFCAEGQRALPRSTRVRRYHQLLLTAQPSTRPLAQPSSVLCFSSGQTRSASFEEKAKALNQQGIDNQLHDYDAKIHEQKEKQKSTPWHREGSEDPPVRRQRSAGKLLTTPSRLLKLVLPLSTVDINTDRKDVEPLALLVHPHQPLSYLTRLIQSELPSITTEKGDSRSPNVSFRALESKDDEVTPHKSAAEPKGPNDPEDQEIGEGGVESYSGGGRESTNEPNGEFIRWSASTEIGDFIRDAARAKEFEVEVEGSPDTIKVAVPSFNDRTFYLRQKLRRISSNISDMAGLKTECDVAAHRTGQRYAMGGCATLVGYWYLVYRLTFETDLGWDVMEPVTYLVGLSTLIGGYMWFLYHNREVSYRSALNLTISKRQGKLYEQRGFDLPKWESLIEEANAIRKEIKAVASEYDVEWDETKDEKDPKVVKALRDERNSRKKSKEKKEDDDDDDGDDDKRD

>CF317_000547-T1 CF317_000547

MSGGVGPRVPKDDFMRALGLESSDARHEGLYKLMRDEAISTYTQLNASRSNLVDDKQNDPSIRPPFFWHHISRDRQIWAVTTTWQNAPVGSPQRQLFDRGSTNGEHAPNWVTLWLLYSVFRSRDTRNNRSRRNGDSGSSSGSGNSEKRMYDQVRNVHRPR

>CF317_000548-T1 CF317_000548

MSSVSSSSTNAIPILLLKTRSQPDDTYEEHFSSPTPAGFEASNIHSFSPTFVPVLEHTRNAPALSHLGNLLRTGELKRKYGGIIFTSQRAVEAWADVVHAVESDRASRQPSQQQQSDPPDPFADMELLTPFPIYVVGPATERALTTLTSQSAAMQQSPFSRLNPSVYGAHTGNGANLAAYILRHYQQLQREHYFTYFEAPRLPFIPLLGMSSQNYGRKRLEKDDARLKKKPLLFLVGEVRRDVIPKTLAAVEDRIEVEEVEVYATQVMSAFERDFEGTCRRLDEEDRDCVRVVVVFSPQGGDVMLKGIGYLDENGRATEMAKNRWWHDGRGERGQARWVVVTIGPTTRDYLRDKFGVEPDASAEKPSPEGLRRVIEEFLRQKNIMV

>CF317_000549-T1 CF317_000549

MGLLDKLQSKLELYRLEQKYAKRKNRQTLSTGATYVDGEYHYDGALSQPISASSTGSSTRGKRMSMFPGAKR

>CF317_000550-T1 CF317_000550

MVPRPLPYPNTHPNKGTQGANTLPGHFFAAYPAQNYEILRRIYWKRRGYFMAVNIPTLPEVERISSSVTRILGGNPSKFTLQGTNTYLLGRGKERILVDTAQGTAEWRKSLYGVLEKEGGVKVETCILTHWHHDHVMGVPDVRAHNADVKVYKHLGNRYDPDQTLQKEEILDIEDGQRFSVGSDADGDLLEIETLHTPGHAKDHMCLLIIKSPDPEEVGCIFTADNVLGHGTAVFENLAHYVSSLKLMKGRTGENRRAFPGHGAVINDARQKLDEYITHRQMREDEAMNVLCYGTTVAPGKTPLKTAEGFPQSTPPAESDAGKELVLGKEWESMEMVKVIYRMYPENLWGPAEGGLLQVLHKLQGDGKVEKTEDGKWKASEAAMKDVIAEAGVSRSSTPSKL

>CF317_000551-T1 CF317_000551

MKEINAAFEHINGSKLWLSGKSDTAPQPKGKPEEGTNSNSGDRAGPSAEQKQRPQSEHKPDRSEQKSSRARPYAKSSKGTYYHPKYGSYVPEASSQKYQNSEGPRFSNAKNQQRTDSYQAKDFSGFGPCPFNGPSQQYRNSNKPRASTSTKPRQSDANRQTSFSGCGAHAFTQPGQQSPNVNERQQPSNNAEPADFDFDFPFRFTDSPFTQEPPDHLWNIFCTDLDTGQRYRQAWTCPSPRGSDPTEEWGHEQFRRAMRRFETHLYFNEPNKINGCFAPDIPSVQQCLLVHEKELNARMKISWIEWRKRFAFTGPLMQSWYRIGELHLLLRLLKMRVAYCYDSELYEQIRQCGLPEMVLGTGVAEMMGPDELNAAKHGFDTSKLKNMDTVCASTSRSILLATTAAWNQIWK

>CF317_000552-T1 CF317_000552

MDQPNEKSFPSTAYDILGIPQNSPEAVVKQAYRRLALLHHPDKAGQAATAKFQEIDAAYRTVMNSERYDEEDQTSRGHAKEETTDPKSPKPEPQPRKPASCTETEFLSLHWLLKDYLATTRRTRSRNASPWVLSKEEKVNQWLQEMTGLYHKRDRGFEAELCSRIGHVLKALRAIQIATAPTLAEATRLCLWLSFQQMLSVTGISTSLNLADELRVEDSFETAEEVVLVSELWRLGDASPEASRTPGQSSTFSSFHDSKLRAYFQRGYGKS

>CF317_000553-T1 CF317_000553

MISQWFVEDIVLPRENAPVTHKIQCIGGRDMSRCEDFANKYCPGESPKLYDSYESVYNDPDVDVVYIGTPHAFHKQNMLDAIAAGKNILCEKAFTINAAEAKEVLEAAKAKNVYVHEAMWLRHRPLVHQLRKLLFDEKVIGDIYRTFADFALEVDIPSLPPTSRYKDISLGAGTLLDTGIYSLTWAILTLDAGSPTNSEKPLALGRQGIITSTTQSNHNARPFCTIWGTNGFIEVEGPAASMPLSFTVYPKLEGDPNAGSLERKEGKKYDFPRIGKGFVYEADDTALDVLAGRKESSIMPWSETIHVMEIMDSIRKQGDTFYPGHDEELDAPQESTIPVLDMFKHLGPLG

>CF317_000554-T1 CF317_000554

MSNAPLCIPAVSCALQSIPNLDGCCINDPSGHFLQTQFWDTSPPLGGNETWTIHGLWPDLCTGGFDQYCNGTRSHSPEKITQILSDASSPQVQGGSHPGLMDFMTKHWLSLDSRNANLWAHEWNKHGTCISTLESQCYDRDSSASGSSQELADGDVLDYFTHAALLYSTLPTFDFFARHGIVPSYENSYELDHLRQAIRDSQHGHEATIRCRNHNELSEIWYSFNVRADLRHAMDLWWDGNQQWNTWVPADPQGQTSNCPTTGIRYLPKGGHKPAPSPTATTTSHTHTVTATATETLGPTARPFTGKGRLMVKVVSDSDTTGAGPATSADSTERDQDTLVSTPIPSQFTGCLIRNGRWYATNALTSCATFTAHDDAKSALAMNDDDDDTNFHLFTLASRLAPCSFVPSSSETSDNRHPSADAHRQAVLHDRAPPASDWQSPHPMSFACDANLPFQSILSNNATIDDQHSESAKRLTLGEEHQYVFWAESVPKGHEQVRLWTEGGEDGRKVKVEVYWEGV

>CF317_000555-T1 CF317_000555

MRFSRTTFLSSLGLLASSQYASAGRIIESNSLNTCMDNSLFTADLFDFSLTPDNSSASVEINGFSSISGNVVAIVSVVAYGFTAINETVDPCSMDLAGFCPMTKGALSLPQSTIPLSSDVLKQIPGIAYSVPDIDGLVRLKVYQVSDSGGIGELVACLEADLSNTKSVYQKAVGWATAIIAGGGLVAAGITSGLGNTNTAAHVAANAVSLFGVFQAQAIIGLTAVEMPPIVQSWTQNFQWSMGIIRVSFIQNLATWYQRATGGTPTTYLSTLSTVSVQVEKKKRAIKRGIEMLAPAIEWTGLRTLSKRQSSSSSSSSSTLENVIVRGIERVGFRARIEQTNIFMTGLIFFIGILMIASICVALAKALIEGVAKAGWMKSDKFRDFRNGWRIVIKGILFRLVLLGFVQMSILCLWELVERDSIAEVVLALIVFISMASGLAWASYKVMSLAKRSVAMHKNPAYILYSDPTCLNKWGFLYVSYKATSYYFVIVILGYIIAKAVFIAFAQSSPVAQAVGLVVVEAAFLISVSVLRPWMDKKTNAFNISIAVINLLNAIFLLIFSNAFGQPPMVAGVMGVIFAVYNIIFIFVLLIMVLVSSIYAIASKNPETRYQPMRDDRGSFIKSQSALTTELDALGATARGDGKDVYANEAKEIDDGSSSSDSLSRPATQRSRFSNQNLQNPNTYTPSHAPSSPGQAPSMQHAPPMPTGYGQHRPNTAPGQQGSTQSFSRPGAGPYGPPRSAHSNYPPQNFNNISQDRNASPWQRGAGYD

>CF317_000556-T1 CF317_000556

MGPSLPQTYTTLTLSSRPKSSIVPGQTFATNTNNTTPPPSTLSNGQVLLQTLYLSLDPAMRGWLNDTRSYIKPVQIGEVMRGSSVGVVLASKSSKLQTGDIVVTNSGGWAEYAVLDDTTCEKVPDTGEGKRRSAIDALSVLGLTGLTAYFGILRVGEVKKGDFVVVSGAAGATGSVVGQIAKIKGATVVGIAGTDEKCRWLTEQLGFDKALNYKAKDFAKQFREVTKPKLIDVYFDNVGGEILDLALSRAAPHARFVMCGGISQYNEAVQKGPKNYLMVVSMRIRMQGFIVFDYEKEYTAAREELAGWVKEGKLKRGETIVEGGLKEAEKALVGLYEGLNTGKLLIEVSRLEDKAKL

>CF317_000557-T1 CF317_000557

MAPSGSSDPRVTPVMSLIAGGTAGGIESFLTYPFEFAKTRVQLRAEKGIPTPRNPFLVISQVIKNEGARALYLGCSTLVVGTIAKDAIRFMSFDTIKRAFADPETGSLSPLKSLLAGMSAGVVSSTFAVTPTERIKTALIDDARNTAAAGKSPAERQFRGAYHATKEILRTHGMYGVYRGYVTTTMKQAGTTSVRMGTYNILKELTTEYEVKQNTITSFGNGAIAGIVTTYATQPIDTIKTRAQSAKGAGTVEAFKGIIEDYGLKGLWRGSTMRLGRTVFAGGILFTAYEQVVAVLIPIMGRKDVDLQNKGAVQ

>CF317_000558-T1 CF317_000558

MSGWDTGSGKSEPSPHHALIIPSQEMPWDVWKRPQAGTKVHYWQRPSLETIDESPATFTHLATPPNPDVNMANNWKGDNWGKGDTTTSFTTNDEWNSSSAGADGWNDSGANSNFDSGAAASIDDFGGSGEGNGEGNGEGAGPSGPGACFNCGQEGHMKSECSNPRVERPFQGTCRTCDQEGHMSKDCPNKPKFCKQCLMENDHDTLECKNPKKIDNSSVPDMSEQEAWALIKEASDDLDVGDFKEAIKILSKAVPEMTYPQLEKELRKRCLNIYLIGLEKEAAPAYTNTNLQGQVGKPYQLGIFTKSSKCPRPILMPIWPKDDADNLERLENTGVPLERGVMICSNCGELGHTRKGCQQESTAPEPMKITCVLCDEVGHRARDERPQPRAARTCKICDSPDHIARECPDKPVEKCHCTNERVMVCRNCEQPGHVARECTEAKDWNKVKCRHCGEMGHASEKRCDPEKKAAYAAAQASAGGEDNGDTNDFDNGTAVGGDDGWNQSGTDIGGSGQASWETDVAPSAPAITVGGGW

>CF317_000559-T1 CF317_000559

MNIKEHYSPAPTPAPLPPPAYPTGPSILSIASALYAYTPTDAGDLALQQNDRIQVSEHMNNDWWRGRNERTGLEGIFPRSYVTVLEEKRPPMPTPNGTIDYNNMPLMVASSGSLNQRPPSKLEEHGKKFGKKMGNAAIFGAGATIGSNIVNGIF

>CF317_000560-T1 CF317_000560

MSTAFPFPRLPPELQAMVCEFYFDQPFYVRVTSEHWMKGDRMGCTLDDRSIRSRSLPGCLRCVPHARSLLQASPDLYKQAHTALLKAFDGHIDCNLELDFGEYCTVNHYLEKLFTYPLPISQIKHITLEVCNYTYCGSEPLESELGTWKKFVELVGQIQLFRLELITIELGIHFDRGPLRNKDDAELFLRGGMDDALVAYMQSYLPKMEIGDPEKIERADG

>CF317_000561-T1 CF317_000561

MASFLQDAVVCDPFIILGLPRASSKAEVKKAFNKLALQYHPDKAGQQATAKFQEIQAAYQAITFGNLCSESSYGDVCNADEPCFESSDRAIWEGPPIPSSLSEKDKFNLLKGRIKRFFESKRGRLLGGTGESTTRMSFETPIQTQIDVIDTLVHTMSKELGAAVGQLLHHLKRMWVDYTMGVRSELIASFRCETMASAIGVLGLLPFETLRFVIDQFIVSERAWIKVTPVLVPQTQSGLGSGGMEGRQILNMVCTSQRTDEDVEMIDV

>CF317_000562-T1 CF317_000562

MDNTKATNPRRYGQCLYLKPECLDEYKKIHAAVWPEVLAQIDDSNIQDYSIHLTMKPRPMLFATFKYVGNDFDGDMARMAANSKVREWWRVTDAMQESPVEGAVGSAEGLGWWEQMEEVFYTP

>CF317_000563-T1 CF317_000563

MSSSTASRRLAAESARMGSQSQQRNASSSSKIKVKNPVVELDGDEMTRIIWKDIKDKFIHPYLDIDLKYYDLGLEYRDETNDQVTIDAAEAIKKYSVGVKCATITPDEQRVEEFKLKKMWLSPNGTIRNILGGTVFREPIVIPSIPRLVPGWKEPIVIGRHAFGDQYRAKDRTINEEGKLEMVFTPKGGKPETVEVYNFNAAGGVAQTQYNTTDSIKGFAHASFKYAIDRGYPLYMSTKNTILKAYDGRFKDIFQEIYESEYKSQFEAKKIWYEHRLIDDMVAQMLKSEGGFCIALKNYDGDVQSDIIAQGFGSLGLMTSQLITPDGLTYESEAAHGTVTRHYREHQKGRETSTNPIASIFAWTRGLVKRGQLDDTPELVTWAENLEKAVVQTVDQDQIMTKDLALTQGKKNREAYVTTGQFMEAIEKRFKKNLAADGLEKVAGQ

>CF317_000564-T1 CF317_000564

MADKLRTLQQLESLQAKYVGTGHADTTRHEFTNTIVRDSYASYIGHPALLGYMSLGMGEPKEAVRGQFIEKMIRGVGNPPVKEDD

>CF317_000565-T1 CF317_000565

MVAIRSLAAVAVFAGTLGNAMVHDNDVLDRLQARDVTVTVTETVTNCGTSSAAVPPPATETEISSLPGTIETTTTPVTIVTTTPITSTVVESTPASNPPVESTAIPPASETTAPATPATSEIVPPTGTDIPPTDTNTLPPASTQTGSTVTSVETTTTPPSSDTTPPASASGTNPAESTTATPPAPFNGVGALNANTGLMVAIAALAAFY

>CF317_000566-T1 CF317_000566

MHPFSSLTIGIFVAGYITARWDLVTRLYELAIFAWDHGVVTRAAKAFSVLSLIYIALILPLERLAAHEAILHPRSPKHGISAREQLKRRGSL

>CF317_000567-T1 CF317_000567

MMDLCGRKTMSVLGTFNHGDTLGSFDLGQIQVFRASDQALPQVFCPIDGALSAQVVLYTPPDPTQLQYISLDPINLTLGHGKPRLHNASAGDDAEAEDQDEKTRVAALVQKLKFHTVMRHKPTLKELALPIIIRQINCSFELATLLQRNTPFVGPRRKREISVSERVVESAQSLYTVAVWSIWTIFMNSIYPALSYAFKVAIVLHRALAEGLLQCLEFRLPLRRMNFPDSTGTSSNLLSLKDVSAACQQVHLRLQQFSYYPIQYTLLRRRYGTWSSIAGTNSDYIRFYNSLWLVANDVILGIAVGSFIHENADATAHFVVDVLTQYTINGLRDTIRWLMSYPGGLKLNTELAAFLGDLFLWVIEYWSASSRLLVLPYLSKIVYLIGFSSFAGASMPIAIFSDFLSLMTVHIYAFYIASARIFNWQLSILVSLFQLFRGKKRNVLRNRIDNCDYDLDQLLLGTILFTLLFFLLPTVVVFYLTFATARMAIITFKAILDTCLACLNHFPLFALMLKVKDSQRLPGGVHFELLESKQAPSAMQSPQPIVEVDTEDEDQGGLPPQSTIEGGQTTTAYIKLSSTPLSIRQIFEQYFQLGGRIRKHYFSPKVVFRLLTGRFVPPLGRGEMYGMQYSALPRERADISEVWKGLFEKQKEQSTNAFDPNGNLFGDELQGLANIRGGARRW

>CF317_000568-T1 CF317_000568

MVHSYVYESCSIQLIPKHISRIVLTLYIHYILGARPFQPAILSVTPTFSPFTPLKWSNVPNAAPRDLTPDKQYAHCATCNNIFQPGKEGSGEKGSGSGSGDKSETDADANANAKAKESGSAAQTREGQ

>CF317_000569-T1 CF317_000569

MGACAKAKARKPSIEWLWVDTFCIDKRNSQELTESLNSMFEWYKKAKVCYAYLYDVDLSTPGRRGFKTKEGEESEWFERGWTLQELLAPQDMEFYDQNWNYMGTKEELASELERVTGIKEEYLETSYNIQSASMATHMSWMAGRTTTRVEDIAYGVLGIFGISMAVQYGEGKQAFMRLQRGLMETSTNESIFAWTKPADGLKCFQIEKLDTKPPPFPPRGESTWGLLAPSPDCFKNSGNVVVTKSKIVPRLGGGYRWTQQGVQFQMSLESGTEATNWFGVQRSDIKLPLNCWEEDTEKTIVLQLAKKNSIYTRERITSLSTTIGAKPSTNNVLGIDQVITRPLTIAQETYDHPQIFYVLTRIYRKHHIQRPAAHPPLTSKEERNDLFDRCLDTTPDIEQYVSKWFLDAPLSEIRRENIKEFFRWAFLNTDAADPDYDDEVELYVEKMEGRMGMKFQPGKSDVKSLRLTLDKVNALHRSLIWYMVRTLPSLRKRKH

>CF317_000570-T1 CF317_000570

MNTLAPLPASNTASARFYKLCATVLNTSTKRHQSSYRRARSRLNVKPDPNFLPSKTVPHDHIIHNPPPSMPNVHHTPSIFLPKDDVRRLLQTPETKSLLANTPAFTPSGKERLPPPVRKPYEKKYHLTEADFDEMRKLRGADPLTWSVKKLSKKFDCSGIVVSLATEGLARNKQVMQKAVTDVIKSRWGAKRRVAREDRQLRREQWYKDT

>CF317_000571-T1 CF317_000571

MDTSRTSQIPPISNTTQQQPGQASEVEERSRRRDFFRFGRKEDDKKKAKGTPSTTSPGGLRPASPLAGLDASRSPGSPSANHPYGATSSPRGLRSASPRTHSPASSMIFERNVQEDVAVAQASPQIPSHIITENHIPPALDATSEAITDRRLNPDAVEIVTHAAHHPAAITITEGHHDAALSPVMSEEQIPSPMPRADTDNASSYANLDSVDYCGPEVPFALEIASIVIGASRDFASYQLVAVNSRSIPTSRNR

>CF317_000572-T1 CF317_000572

MSNPHPGPVGPDTLVTVKVLIDGQNRRFKLALRDLGAHVLPQKLRFLLQVPTGAEVKFDRFSDSAGTYVTLDASNPAIYKQLYRAAKAKLKLRLKATVITADKKDDISTPGQKAMPESSKRNSYLETVLSNPPAEHAYSFKPYLPTGVNIPTATVQPMIEKLAQPPLSRPTLPTLNDFCGTTFSIDCNHCGESVLNEHYHCSKCEMGDFDLCPTCIGRGITCDGDDHWLIKRSIKNGKVYASNTETLPPKDKVQAPAVSVEHPAEEDQRTCNNCIIQLGASAFVTCQQCADFDLCFFCLEQGEHGHHPAHTFAPVDASSASVSPYIKTLCQQGRGMKHDAICDGCDAQIMGVRHKCLTCPDFDYCSTCIHVAVEVHPGHRFVPIYEQLGMVSSKKEQHKGIYCDGPICTARARKSYIRGDRYKCAICHDTDFCANCEALPTNPHNSTHPLIKLRTAVRQLSVAAHNDLDNGSSATLGDRRAPVHASTETTRPSSANAATQVQTVAETTPTEPKQDVVKQTPVSSPADLQAWFESESTPDGTIFLPNRLVHQSWTLRNPGPNAWPAGCAVHFIGGDEMRNLDDKHPSPVSSMTIANRSNTLTMPLEPGKTQTFSVILRSPARQGRAISYWRLKTADGMPFGHKLWCDINITQNNPKPVEAPAVVPVEAPAERDEATVEAEQSQTSSQMIFPKLEKESPESSVDDIKQEAIRFEPSVASEEQDLLDDLDSMTLDDEDTEDGFLTDEEYDILDAEDEEYLVNAQRAAQK

>CF317_000573-T1 CF317_000573

MDALKQDLNSTIDEMLERKGSSIESREEALKRYNRILTSHHFGDVLYARVDDILASLSKSIKAESSTTETVRALKAVALTAVSFENGALYDTMSALVKRTIEDSQDNATKAAALHCLGICLTFGGASEGEFADTCTWLLEIVQSDGAFVGADDNAEVVAAAIQTYGYLVTELHDVEAESEDAVEAFMEQLDSGDADVQIAAGEAIALLFEKSCTPREDDEDGDGEEADEADSDDSNMVDKNLVKRYNAYHNPAEVLDKVQDLANLSSKGLNKTDKQKLHRSFASIALTVEEPRGGLRSNNASKLVVRIAKEGEIKVDKWWKLMRLNALRRLLAGGFIQHYFEGNKQVLNSLPLLVRSTRTGGTLSPGRGSRRTPRRGDKYRNDRRFVAADEG

>CF317_000574-T1 CF317_000574

MAPNLEPYFKQVDASADAFIERLRAAVAIPSVSADDAHRPDVFRMGDFLASELEKLGAEVQKRPLGKQPGKEHLELPPVVIARYGNDKSKRTILVYGHYDVQPALMEDGWATKPFDLTVDEKGRMFGRGSTDDKGPVLGWINAIEAHQKAGVEFPVNLLCCFEGMEEYGSEGLDDFINAEAKQFFKDADAVCISDNYWLGTEKPCLTYGLRGCNYYSVEVKGPAQDLHSGVFGGSAQEPMTDLMHILSRLVDQKGKIQIPGINEMVAPVTEEEKKLYPDISYTMDNLYESIGSKTSIFETKDETLMARWRFPSLSVHGVEGAFSAPGAKTVIPAKVIGKFSIRTVPDMESNEVNELVFKYIRDEFKKLGSKNTLDVSCQHDGKWWVASPFHWNFKAAAKAVKEVFGVQPDMTREGGSIPVTITFEQATGKNVLLLPMGSSTDGAHSINEKLDRRNYIEGTKLLGAYLHYVAEEPAKE

>CF317_000575-T1 CF317_000575

MSVTKPGRPNGHPVKRRKLSHESIEIKDGLDSTAEPHNDGPEVKTAGAHLSDDDVDDSESSEVPAAHVARTKSRKSNAPASAITPSTGSSSATLTLQTQTLIAELKPDYASRVRKMRPVADKVIEIIKAIPEQQSMSLTEAQTFSRKTLGVAIPWASAPPSDVKYKFSFSKPSSTTVQGALMHSLGPQTSSGMVIVPEMPLSTFEGKDYLNFRALHKRAFYLACIASPLQKELQDDFSISFAFADGNELIPVIRLVAKSKDKKSSEAVFEINPSLPSSLGPVDKMTPTHNCIRKGDLTKEQHTSPDNASAFYNSSLRSLAATNSLQPLLKHAASKADHFRDACLLGTVWLQQRDFSSAQADGGFGLDEWALVCALLLESGGHQGRPLFSPRYSAIQFFKAMLQVFSGRDMYDPLVVRGVTKLPRSEQPVLYDARTGVNLLYKMTPWSYARMKHHATISLAAVNSKILSGFEPTFILKASDPIMQFDESYEIDMSRSSASELHLLYDTIKKGLGDRASLVDLHHPQRLSWTVSSPHPSVEVAKVTIGLLINSEAALRLVDHGPAVEEKQESKQFRDFWGDKAELRRFKDGRISESLVWSADTPVAQQIIRYLCAKHLKLSPNAIKPFAGLEKLQLQGSVSSDEAFQAVNTKFQSLSSTLHHLDGLPLPVRSVSAASEHLRSSSLELPLEPGAAHPIDVIIQFDSSGRWPDDLRAIQYTKIAFLNQVADKLTQSDRTLQTRIGLENTFASSLGTHNTSYLDIIYASPTPAIPPIIFRLRIYHERELHLLQQAVAARTTLSPPVREIHQLALHSQRQTATAVAHTTAIRTLITAFPPLSVTIRLLKSFIASHNLSLHVPDPILEILAAHVFLSPAPWSTPGTATTAFARCIHLLARWDWSVEPLIVDLSLSQDMNIEQRRELETRFAAWRKMDPNMNTVSWFVGTNIDATGVVWIQGIAGQDPKPPRVIAGRLTALARAAMGLIKSKSDTESNIMTKADWDSIFSSSLEDFDFVLRLKGQNKTKSKNGQYKNLEIAAGLDVDTTGVNIVASYVEDLQRCFGSSAIFFYGGKKGGSNAIGGLWRPHVRGKEIRPWRLRLGYSTVPVPMPTDAEVDDDEGDDGAAEKAMCKVNVDGMLAEMGLMGEGLVDRIPTKDA

>CF317_000576-T1 CF317_000576

MKTGYPRFFIHLTIQELEKEILSQYGNPGERGMLFPSRRTAQMCQAFFNDKRPDISKKVRLLHLEPTSSQLQNTRYVLSRLSCVLFPAADFPTAKQVWQHSGAGISSRRAEFCLKALKEGYLFPVDGPNTVSSAHHDVFSKGPRRYQRGASQNNIPFGVNGHSHYHMNGDQANGGDNQDHDQFIEERFGRNLNAKNAAKVKLAIRRRIAGSLTENTDLDDALDASESCRQKERQNEIVEGDRLRDVSEDDVYLYPSGMNAIFSAHQLVMGEAQKRGKPPLKTICFGFPYIDTLKVLEKWGPGAKFYGNGTDEDLDDLEQRLESGERFAALFTEFPSNPLLRSPNMARIRQLADKYDFVVVVDETVGNYININVLPHADIVVSSLTKIFTGECNVMGGTLVFNNSQRLFRNLKDALRQQYEDNYWPEDAVFMERNSRDFVSRIERINVNAETMAHTLKTSPFVKEVYYPSMVPSKKYYDVCKTSNGGYGGLLSVTFHNPEHAPIFFDNLAIQKGPSLGTNFSLSCPFVILAHYTELDWVQQYGVDPYLVRISVGLEHPEELTQVCEKALGAIDRP

>CF317_000577-T1 CF317_000577

MAWRCSGRTNAELIANLYKSQLITSDRVRASMSSVDRAHYAPAAPYEDSPQSIGFAATISAPHMHASAAESLLPFMPENKGAKVLDVGSGSGYLTHVLANLVCGADGKGDGKVVGVDHIAGLTEMSTRNMGKSEEGRRLLDSGKVEFVTGDGRKGYVEGGPYDAIHVGAAASELHPVLIEQLNSPGRMFIPVEDEEDRGMFGSQYIWVVDKDEKGEIKRRRDMGVRYVMLTDAPK

>CF317_000578-T1 CF317_000578

MKAVAISKYGEIDNLVAIDLPDPSQPKTHDILVEVKACSVNPVDTKVRAGTYDDYPDYYDNTPALPQVIGFDGAGIVKAVGNKVQAFKPGDEVYYAGSPIRHGSNAQYQLVDSRAVAHKPKSLNMVQAAAMPLTWITAYEALVERMEIQKGEKAGILIVNGSGGVGSVASQIARTVLDLPVVITTTSRAETTDFSKSMGATHTVNHREDIPEQIAKLKLDVPLKYVFITHTPTSKYIVDAAKICAPFGKVCSIVQDKEIPMYGTEFLAKSLTFVWELLGTKPYYGVDVDSHGKMLKDLSAWLDEGKVKCHLSQTLPLTAEGLRNAHEKIEGGGAMGKVGLSVSAEGMDEAKAFT

>CF317_000579-T1 CF317_000579

MRRLFERATSSSLVTCYSASLHAYHHVVSVPPVWHRHASLTGAINGGVRRAAAALRSAEKRRLQDGQLGFWELKDPDKRAQAQENMRRQRQQEYERKKDLEWLSSDTTVAAEPVPPHTPEAGYQDKRGRVKDWSRGQLASSTAQSNGPTEGLRKARSVRATALAKGMLVEAPKSMPYSEAASEFIYGTFAVLAALQAQKRKFFKLYIWCGEDGNLSDSDDKTTEVVREARAAKVPIVRVAGNWDKLLDKMSDKRPHNGLVLEAAAIPRLSARYLEKVESIKSPLVVKLNHMTGPEKEDLGLSLTAESMNLPLDLRKGRRFPFLLWLDKVTDTGNMGAIFRSAYFLGVDAIILPRHGTAPLTAVTIKNSAGAAEHVPILNIDNELNFMQRSQENGWLFVASAAADSESTLARSMKTATVEQRPKQVLKHHPLVLMMGNEGEGLRPFLQKQADFSVSIAGARSDGLIDSLNVSVAAALLTQRFLAPYINGASADRFQGKTTIMSALFDGIEMPAAADFHVHLRDDRMMEAVTPTIRQGGVDTVFVMPNLVPPITTVSAALDYKKRLQAIEPNVNYLMSLYLHPSITPDTIREAKRAGIAGVKSYPAGVTTNSSSGVVDYESFYPVFQAMEEVDMVLNLHGEAPPSPSTTTEPAKQITVMNAEEAFLPTLKSLHDRFPKLRIILEHCTTAAAVAAVQQCGPNVAGTITAHHLFLTVDDWAGDPINFCKPVAKLPSDRVALLKAATSGDPKIFFGSDSAPHLLSAKKGLSDGGLGAGKCAAGVFTQPNVLGFVLEAFDNAVQAGIVGESDLSQEKAQGFLGGYGRKFYGVPQATGNVRVKARGMNVRNVTVAGKSGNEASMNDTVVTFRSGKPTYAVEWV

>CF317_000580-T1 CF317_000580

MEEEDSLDVNAWLGKPPEQATSKATSKATSEAHDVEGAHSAATSPSPSVSSHGSDLIEIPHRPAPAQEVVSDEETVVEVDQDALQDEAQEEVDGFVVNRQENLTINIPELPEAERDEFDYLPDHFTAKRILYALPNRQYIVKLGSGEIDLVDGPDLTTAYRNGRPALNAHRGAVMQRPGRRPAQPPMLPSGMVDWSKIHASDTEDEDEDEDEQPRRKKRKTYAEAELSDLDKASSSADNFADSEDELPPGRRSTRLAPQRRSNYFKSDLTELKDFIDSDDATPRWRRGRSKREAAKQRTTSHTTRAMRAQRAASSEDRATLHNNARRSSRARAHPKRSMRERQEDELSSQSEKDLGPKIVATKEIFHKLPANDPFRKRHQQECATCYYTGDHEAKGPLVFCQGCANSYHKTCLGNRGSREHLVTKAGDDLFVLQCRRCIGVAREKDSTAPHLGRCTGCDKMGPVSKPFRRRLATRQEQIQREENGGSDPITIIQPGIINNPENVMFRCATCNRAWHMHHLPNRKTAHSGDSDEEDLDEKQLAQKRFDLYHRSWICKDCVENQHAVDTLVAWRPNDQDSYIPGTTSDQLQESQKEYLVKWKNQSYLRCTWMAGSWLWGIASASSRAAFGRRVENRLPRMTTKDAIPEEYHRIDIVFDVRYTSVVRNSSEGIDMARVKEVDTAYVKFKGLGYEDAIWEKPPVYSDTERFKDFREAYEDFVRKRYMSIPPQSTLRRTLANVRLQDFESNLIRKAQPTNMTGGKVMQYQLEGLNWLYYQWYRQHNAILADEMGLGKTIQLIALIATLVQEHKCWPFLIVVPNSTCPNWRREIKKWVPSLRAVCYYGSSVARKLTQEYELFPKDPDADVDKKKRAEVKDIKAHIVIASYESIIDKGVQLSLSRVPWQGLIVDEGQRLKSDQNLVYDALSKLRFPFKVLMTGTPLQNNARELFNLLQFLDRDKHNAAQLDERYAELTPENVPELHDMLKPYFLRRTKVQVLTFLPPMSQIIVPVTMSGLQRKVSKSILSRNPALMKSIFSRDGNAPVKERTNLNNILMQLRKTLCHPFVYSREIEDRSLDSDASFRTLVEASSKLQLLSIMLPKLRERGHRVLMFSQFLDNLDIVEDFLDGMGMLHRRLDGTISALEKQKRIDDFNAPDSPYFAFLLSTRAGGVGINLATADTVIILDPDFNPHQDIQALSRAHRIGQQNKVLVFQLMTRSSAEEKIMQIGKKKMALDHVLIEAMDKEDEAGMDLESILRHGAAALFNDDTEDDIIYNEASVDKLLDRSQIESTKTSENQSAESQFSFARIWANDKATLEEGLEESADSGPHTPNPDLWDKILKERQKAYEEEAERKAQELGRGKRRRGNVDYGGKPTAAAEGLDSETSPVKNGAEVGYVSDDDFQEVSQETDVDITTAAEDTEPEQVQMVKAHPFKRVKVPMGPAPHFNGDAPADYSRVPIPPSHTCAACRELHPMGWCRLKIAGIEYCGLCGLAHVGHGRTCPHLNDEKQVETLLLTLKESTESRELIDEAIKYLRVIRSDLVSQRRQRERRAQQERESTTIQQKQIQVNGVGNGGATDSLGGGRAAPMPGGVSLGANAGDYRNPHQPVRNEI

>CF317_000581-T1 CF317_000581

MPKFNIVVFAGDYAGPEVTAEAVKVMKVIEKCVPDVEFNFQDHLLGGCSIDAHGTPLTDDALSAAQNAHAILLGAIGGPKYGTGAVRPEQGLLKLRKSLGTFGNLRPCFFASPVLAAQSPLKTEICKDTNFTIVRELTGGIYFGERREADPADGENESAEDREPYSRKEIERITRLAAHLALAKNPPSKVWSLDKANVLATSRLWRRVVTEVMTKEFPQLEFEHQLIDSAAMIMVKSPTKLNGIIVTSNLFGDIISDEASVIPGSLGLLPSASLSGIPDGKGMCNGIYEPIHGSAPDIAGKGMVNPVAAILSVGMMLLYSLNMAKESVLVDQAVRLVIEEGVSTSDIGGKQSTSEVGDAVAAKLESLFKST

>CF317_000582-T1 CF317_000582

MVWSILISIIFSGLAVVAAIDHCPCGYTVQDTGEVYTHRLIEDFSKYPDITNLLRDPKAKHFTEDWMLYDFGKKSDNHDIRLDTKFEASNIQIKQGELVMKQKGYSRRDREGFNHVSIAGIQTRATDILHGTFRIEMKLKGYDGGSDDTPPQPASDAFDKPTDVAALREARLKSMETPATGRQKMKVEYQYSRPVKLQASTSTTTATKAKSVAASSGLFGRRTSILSAKPKKSKDDGYQSVYDTGDPSMADRRDLQKELEQQTKATQAVREGRTKRKSSASASTADKPKMERRKTEPASATTRRRASTGATPRPTVRKSTTSTGASDIKPPDASAQNHKDHHHPSGSTTGVSRPAKPQRASSVFTSLFAKPPPPPEKLVSCLTCGDDSIPVSKSAKLPCAHRMCHACLKRIFKMSIKDPAHMPPRCCTEKHISLKHVESLFDDEFKKNWNRKFKEWTCKNRIYCPKKGCGEWIQPKHMHIQSGRKVGTCPKCKFTICAICNQRAHRSRECPLDPSIKQLTEIAEQKGWRRCYNCRAMVELKEGCNHMSCRCLAEFCMCCGAKWKSCDCPWFNYDQTVNVIDLGGDPVRYQQELDRRREQMRRDEEMARQMAGLGMQDRAQRPGRIRGGAGDAAGVEAENVDDHQLNANFLQQAREALAANYQNAEVAARGLLGGWLNGRENVPAGLPGDLNQQVEQLLQEQQPDPLQARLGRRRTYRVRHGGANT

>CF317_000583-T1 CF317_000583

MAMVYETHAGIAPSKDANAYILELVPTVANGLAAITSANELFVVDRRNLASAQVRLSDGAPRGISCLVPGDPEGQTLICSGTDGTVATFDLRSHSRVSDFKIDRAVTVLSSSGPTIAVGTEFKNQQAIVSLWDARQNKQIWQNAENNDEITTLSFHPSHRNILLAGGDDGLVSLFNTDIVEEDDSLIQVINHGPIHKAGFLGDDRIFALSSDQNFAVHHVSTPGDEQDPEPVLIGDLRPLIPCQSSRIDMVKLEDAQRLDTQPRLDTASKHSIMGAHTEEVVRAIFIDDVAGAIFTAGEDGYIRASSSSSQAGDDAAKASKPSKHKKSSQARYKPY

>CF317_000584-T1 CF317_000584

MADPEGSGLWIDNGRGYCNFHRGQYLLPVDQKEQERLDIMHTMIQAARPKPLRLHHAPFNPQPDPISGRRHGRVLDIGSGTAIWLLDMAEKYADTEFYGLDMANMAPDGLYHNIDIRPVDYESPWALGEGSFDFIHLQMGLGSVGNWPLLYEKIYRHLKPGGWFEHVEVDFTPQCDPQDATLPQDGILRKWWHTYVAPPYAVVGRPIVYDPTTGDLLRQTGFLPAKHVEYRLPLNGWSSDQAEHVSGTWWEYAMSYGEGRGHGLEALSLAVLTRVQGWPADHARRLCEDALRQATNPTVHAYNKLHIWWAQKPPDAPT

>CF317_000585-T1 CF317_000585

MHSIAYELNRPDLEEVQKLLQPAIAANYSHSSVEVIQCPDLRAPPFNLAAEGLSGTPVIADVGGQSNLFPRPQFDKKYSLVDIAKDCMQMSSKQGMLIGAGAAPFHVVGQNAELAPNIAWMDSYDNLMNLAYVTKVDRSSGRDSITCQKSPSTDCALMMNLFGSSGLPGPVIKITARKRIGKDSCFTDVIRFALHKHFGDDRPVSMGGVFVIRKGKANFHVMPDFPPESELPFTDREQLNDWLTYHNFSVQGNGSGDEIVCLSVLHSADPDEKMGLRMEHTHCFGRNGRGGHYHYDVEDEEIEYEGYFNLAETIYRVDRPES

>CF317_000586-T1 CF317_000586

MPPWISSRLLSPIRKTYIQQHPSNGTTQRVFRSDSDAAQRAKAIDDYKEAELSFKVWLLEKVIEHHDSRTSHFTLSELQGMVENSEEKRTTLKAGATLNMEKIQQLVAEAVNADVKLGWVAKKNLMATISGRRHVSDTWFVGHVDKHTHDEWTTSLEVELQKLERKTAPAPLSPVAQPVKRKESREEILAKTSWR

>CF317_000587-T1 CF317_000587

MLALRSFARVAPRTTLRAAPVRASQRYIAISRPTILSRTTQCKAFSTSRTWRSPAGDADVELSAKLEHERQLELDSKPDNAALPRELEDFLTDSPWTVQDQPGTENIVMTRKFGNESIKVECSIADMNAEREADELDEDSALEDEPDFGSDPTSGGKRTINQSRGGKIDVAPEDSIAPADRDADPEGGDVAAYPLNLSITIDKGAIGATNIIARSEDGNVEIEYVHFYPRAELIDPKSSEAVKEAQNVYGGPPFPYLDAELQQMYETYIQERGIDTQLSMFLLRYVDYKEQREYVQWLDNMKKFVDA

>CF317_000588-T1 CF317_000588

MSQTCIKRTTLFKIPKEEDIPKALEAYKELERTAVKDGKPYILSCDAGKIINTSETRSQGYTVCGQTTFSTIEDVNYYDNECAAHARLKSIMTPIRTGAAVMLFESERLPPPSS

>CF317_000589-T1 CF317_000589

MARALDKVQKKISKKRGGKPTALHENSRDARRLRQAGAREDKLAKISSLAQKSNQHYVDRVAWFQDATADSTFPSSDAAMQELIEQFIGREDEELAELQAAQRPGRPRSKAEDRILDRKDVEQKEHKSGFWMPELRDTDSLEKLQRWGGQWAGMSTLKFVRVHKDADIKASSFPPKGLS

>CF317_000590-T1 CF317_000590

MGVSGLLPLLKSIHKHTTLKNYGGQTLGVDAFGWLHRGAVACSYQLGFDIPTTQYVAFVVNRVRMLLDFGITPYLVFDGDSVPSKAGTNAKRRKEREESKVRGLALARSGKKDLAQQEFQKAVVVTPQMTFEVIEAMRRMDVQVLVAPYEADAQLVYLEKEGIVDGILSEDSDLLVFGAKKLITKLDQHGTCIEINRGDLILTKEVSFAGWSDSMFRRMAILSGCDYLPNVNGVGLRTAHGFVKKHKEIAKITRVMALTGKHMVPPDYLAKFADAERAFLYHRVFCPRAGRLVHLNTVPPDVKVEDMPYLGADVDAELAVGVACGELNPRTKEPFTRTTQRNFFAVQRPALQENRRQTTGGSNDLKPKRSLDNFFKPQRRPLAELDPNTLTPSPSQQRLYERHRNSSWEPQLANSQPQSAPHLRRVASDVPYLSPAPGSAQGARASFLVKAAAQSTYKPVKRQRLCSESDEVSPSKEIKQSKFFSPSMVENSPLASKKTQGRRLKKPVFDVFSDDAVDGIMLELEKQVTQASEQTIEYPELPAPLTTEEVTEVTDVVPQSSPVKSQTVLADSQSSHGTVELSDMSLHRQDDSQETVRVEVNDVANAAAFGSLLDYHVKKQNEAAKARTAHGTFLNQAPHKQAAALASLKSLQAVAAQSDDETVKEDNEPEAEDFASPLRPSNSKFQVLARTFACQSPELQSRALRSLGETPSISKISHALTPVGAVGSEDVLPPSSPVRSLDEVSEAEEPAVRKLDLKAFRYIAS

>CF317_000591-T1 CF317_000591

MPAFATGWKRFVPVIGYHHVLMIIIAIAIVLLALLLAGCSSTSVLIPDIYLISLYYQQYPAIFSTIQVDPAVTVAIANIVGNANLNVRVGYFGICIQEGGGAWMCNQNATALADLVTVDDDPLNLIWVADTFKDAIVFPYLIIIAIIVAFLTFLLLATFPGWHTTRTEDGSEHEVKPFPSRAVSQVALATIFIASVFVLVSVMWQHTAAVAASQVAQDLGNGSIKAGVGTVAMVLGWFGFGLLVIVTLGLLVMILSIQLLNRLTDED

>CF317_000592-T1 CF317_000592

MFAKARPTRVQISQITRISAHPLPRTPANLVLNSQARLLSSSPTLSSEDVIKGVTMDQTKHDNHPSNGKKPEHEHTASSARTEAGRNHPAKQPDPQQSPQRSTGFETDGPGSSEAGKGKDTGNVHQEKGEQPGKHQTWGEDK

>CF317_000593-T1 CF317_000593

MLHLIAFVFALPPLYILYAAALSYYNAWQYDRKAAARGCKPAALRPYKYPAGIDMIMRIMDADKRHQIPNEFETLVFDDMAGKPTFRQYLFGKLNIFTVEPENVKAILATQFEDFELGHVRRGNFFPMLGNGIFTADGQDWKHGRALLRPQFARDQVADLDLEERHVSDMLRHMPINNSTGWTDDVNLQPIFFRLTLDSATEFLFGESVHSQISALPSSATEKDGLRNPTGLDLIEVGKAFDRATHMLGKRARFADNYWMYNPREFRQDCALIHKFADFFVNRALNSNVEKQDGEKYVFLDELAKATRDPVEIRSQLLNIFLAGRDTTAGLLGWVFWVLSRHPDIFDKLRAQVVEAFGTYEKPRNITFATLKSCTYLQQVMNETLRLYPSVPLNGRRATRDTTIPLGGGPDGKSPVFVPKGTGVDYSVHIMQRRKDIWGEDAWKFKPERWVGRKAGWEFLPFNGGPRICLGQQFALTEAGYVIVRLLQKFDKIENLSAVTEQRDSTQYQYSVTTAPWEVMLRMREAKN

>CF317_000594-T1 CF317_000594

MGVLTQPAETLRQVNESRDMLKKAKEDMELSHEAKKMPRKHTFSKLPGFYGRKVEQAQLRKVLSNNPKLTVLFGATSVGKTALLREVLATDEFYVIEFDLRISGFADIRTLYVALCEHFERFFEEMHDDEMQKQKLTFKHLAHGLTEKEKADGGHEVTVADVTGLMESLQSALIRYWEYDPKANPKNKKASEGQDQSVRHRTSRKREAETSETWTGGSSMDTAGGTHNNGEEKTEGTFKKRPIVFLIDEAHKLPALVDDQLGLKVFLDTLLVLTKQDRLCHVLLSTSDPLFHHFLRKMNFGYHAQLLTIGNCTREETHSYFVERIMPSVSASLAPRLDFDPIYDAFGGRLAHINDYVDSWVNVDGKMTPYTSPIFIQAYTLLQFHLTRSDFETYAPLSTATAGRYAGGDDTKFSSEALMYVMRKVVEEPYSLVYFDLCRKIGTGQVDSMINARILELRWTKSMTPEENWVERKWSEDGVERPIVLPSTRIVRRAMEVILNEEGATKSEDQDATSEPRGDDEAFSSHQGA

>CF317_000595-T1 CF317_000595

MDMAGQNPNNSSDFVRKLYKMLEDPSYSSVVRWGDDGDSFVVLENEKFTKHILPKHFKHSNFASFVRQLNKYDFHKVRQNNEDGQQSQYGPNAWEFKHPEFKANSKDTLDNIRRKAPAPRKQGQVPEGDLPIQQIDLMNQQMVAQAQQIQLLEASNHELRTNHQTMVQELMRLHRTVLNHDKVMQDVMKYLNSVDAQQRRNSKTLFPNAHENAAGTNLTPTSQNQNIAAADEELPASPLQHAQQLMQEYNVDTQLNFAGFDPNYQQHPQQGNGLRRGPGSVHSSGSMGYSKLSDGQLEQMVYPTGGNNGIDPMYSEHLPNIPYGMPGKEADAAELRTKYAETRKKSNYNDPGWVRNPRILLVEDDPTCRQIGAKFLHSFSCTIDTALDGLEAVNKIQEGAKYDMILMDIIMPNLDGVSACHIVRQFDRTPIVAMTSNIRQDDIQMYFQHGMDDVLPKPFTRKSLLDMLEKHLIGLKKLPAGMEPPPPQAVNPSMTTHSSGPSIRDDMSAAASPAGSTGTWNSPSQYSGVSPVAPGMPMQYPNYIDNNSAAYHHQQTSPMNPNRVMPPAPMPPQGMPQQLMRGPSDMLPQDMNPAKRQRVSGAYGGPPMTARQQ

>CF317_000596-T1 CF317_000596

MVKVGDPIPDVELFEDSPGNKVNLSKELASGKGLIIGVPAAFSPGCSDSHIPGYLADKRTKEAGKAFVVSVNDAFVMKAWGKQMDSDKTSGLRFLGDAAGELTRAWDVEFDATPLMGNKRSKRYAVMTENGKVTKVAIEPDNIGISESAADKFFS

>CF317_000597-T1 CF317_000597

MSRARGSSMNLEIPQSSGHDLEDPGAHELSSDDEFQDASDERKKGSRPVTPSSPVPITRVERVDDKPAHGEVPGTSAYDIRRQDAVPDELEVVPEGSLSKRNSRLLSDTSLTPGGTPVPRTVVEKVDPSSPSYGDEPGTPAYRNRMADAAPDVVLKSPDASRSTSQDEGESRHGRSSSNMSVPETVVTRADDELAHGEVPGTVAAEVRKRDATPDRVEVVRDEPGEAQNASEDNENDDDDHDAQGQNEGFGDDFDDFEEGAQAGADDDDDFGDFDDFEQGVEDDSQDLAPQPQFTEPQTPAASFPLIDFEALSSLPDLLETTKLHLDAMFPSTTQAQEASVSSDDIPADSPIFPTERSRSLWKQLITPPPLQPPNWTQSRIRRLFLISLGVPVYLNEILPPSKQKKLILPDITLPSRNSTPTDEQDSVRPENDSIAKLKSVRTNDSTTSVDSTQSAPGAGSSKRGRERPKSTKPGPPPAPALDLNAVKRICATTDEKLQGFTDEELQDHIKELEALTEKTSQLLEWWLKRRDGLRKEKEAFEGVIENLVRHAKRVRTTR

>CF317_000598-T1 CF317_000598

MAFKFTPSTSGQPSGSLFGTSGTSGQSNGGLFGTSGANNSSSTGGGLFGSTGAAAGTGRTSTGFSFGQAANTQNTGSSSSTPGSLFGQASNTQNAASTGNISSGTFGKPSNTQDAASAPSASGGLFGGGATGGSSSQAPGTPGQSATSKPPLFGSTTPAPSGGSNFFSNPSTTPAGQPPANSVFGASTGGATGQSQTAATNTFSFGNKQPAATTSNNSTTPAQTLGLFGGQQSSAGGLFGQKSDSTPAPSGGGLFSTKPANETSGSASAGGLFSAKPTATSNTPAATSSNPFGNFSLGGGGSKSGQSDASKAPDNTQSSNSAGGGFSFFTKKDDKPAESTPKPDAPKAFNFPSSAASAVPEQPTSSQPATSSIFTSTNTSAPSSGGFQFAKPTAPASQTNEKAKTGGFSLPSSTSTSQASTAATTSAGLFSMGGGASQSQGSSAATTTAPAPASAPTSGLFSNLGGGATSAAPANAPSTASAPKEANTFAASTAGPPATAQSRLRNKTMDEILTRWASDLTRYTKEFKAHAETIAHWDQIIVDNSAKIDKLYVKTRTCEKQTMSVEMQLTAVENQQNELEAWLNKYENDVDEMLAKDGPAQNELGGPDQERERTYKLAERLGERLDDMGRDLQSMIEEVNAANASLGKSSKADEPITQIVKILNSHLSQLQAIDQGTSALQSKVAAAQKAASGMNYMNGGISGDNRAAVDDFYRSYVGRR

>CF317_000599-T1 CF317_000599

MFRTQIVKLTRAPIRSFSVAAVRPAEGDTGGVRAGGSASSDSFSRREAASENKFIREREMETLRQLKEKLKAQRKHLDELDAHISDLESTKGGEQN

>CF317_000600-T1 CF317_000600

MATNNVTEERGDVVGATGGPGPIPPKEKSSTTRGSVMLNNPILNRKMGVEESVYLAKLAEQAERYEEMVENMKVVASADQELTVEERNLLSVAYKNVIGARRASWRIVTSIEQKEESKGNESQVKLIKEYRQKIEEELGKICEDILEVLDKHLIASAQTGESKVFYHKMKGDYHRYLAEFAVSEKRKGSADKSLEAYKNATEVASTDLAPTHPIRLGLALNFSVFYYEILNSPDQACHLAKQAFDDAIAELDTLSEESYKDSTLIMQLLRDNLTLWTSSEAEPQEQAGQSAEAPAKETEGASAQESSAEKTES

>CF317_000601-T1 CF317_000601

MSVRVVARIRPLLKAEAEKDQILSIHEGNNGKAQIVKIPNPKIQSEEYSFNFAAAYTASCNQQELFDAEVAPTIKHLFQGFDVTLFAYGVTGSGKTHTMRGGKSLADRGMIPRMLSNIYRKSKALEKASNGETTVDVTMSYYEIYTDKVFDLLEPPEKRTPTGLPIREAEGGKTIVAGLSEVVCSNLKDFEVLYDKANANRSVGATKLNAESSRSHAILCVKVTISTASETRSSTISAIDLAGSEDNRRTGNVKERMVESASINKSLFVLAQCVEAISKKQSRIPYRESKMTRILSLGQNNGLTVMILNLPPTKAFHLDTLSSLNFANRTKKIEVREIENEPIFKGPPRPPTAATTNGGIARQPLRPLNNAANANILANREAKTDKEKPAKLFSVYADKSKPTATTRAIPQKSSPLKRSAADALASSRPTKQSRPTPSSNSFLRKGPPETTTITKASLDSLVEKKVAEILAAREAKAAPPSAAPQQAETEREQAISAEVQARLDSIEKRLEGQEGERAEGLSYLFMAKQHQARGEDGNALKMYELAQPFFPGNEKLERKIEKLRAKILSSRKRERQTTFEDDTAEQSRDTVQEDPDSQRTRGGRKVPIEDADESYQADENEDPYHHGGEREADDSDEESPRPRKKTSKKTLNRAKSRAAPRADSPDPLVDRDGQEPDQSLSAPTPRTQYLIDILNSRSVSKLKALNGLGVKKAEAIVQHLQDDEDMQLADWHDVLSLDVRGIGKRSWEVMREGVLV

>CF317_000602-T1 CF317_000602

MASPSTRNLSQIYRAYIRAILDHDVDAMYRFVSVHVVHNGTQLGLEGYQELLRRNIIDNGMHIEIKRLITDENHVAAVLVFTTGPSTRRLAGIDLNSQPFSYVENVIYDFEDGKIAEVHSLFDIDTVRSHAREL

>CF317_000603-T1 CF317_000603

MPTEVVYRETRRKYRWPEVQLNLWIFVILAAASTVLGIFAWFMVVQQQLGIGIPWLFPFGVVTAGLTLIFLLIILILAGRRLLIPGVILLGSFILFVLWLTTLIETAIQLFGPSGNVNANCNTYVQGQEYRGISIETLAWLTQSNICSCWKAVFAWALIATILFLWMMVLAWQVQNDDDE

>CF317_000604-T1 CF317_000604

MPSLHPRIDNGLTKGKSDFAGGKLHCRCSSSPVEVKLSSNVAHNHACGCSKCWKPSGALFSIVGVVPRDSLSVTSNASKLKIVDNTAAIQRYACKECGVHMYGRIEADHPFKGLDFVHAELSDEPGWQEPQFAAFVSSIIEQGFDPEKMGDVRGKFKEVGLEPYDCLSPALMDLIAAYTAKQAGIKSKL

>CF317_000605-T1 CF317_000605

MAAISALMINDGDSSHDKPNGGQISTAPKPSVRVRSPIPLTVATYADSAPSGAVQEPVGSQGTSTQSAQKPDHKRSNSEAMEINDSSDEKDGSDVDEDQDEEGSASRKKKGQRFFCTGYPPCNLSFTRSEHLARHIRKHTGERPFQCHCNRRFSRLDNLRQHAQTVHVNEEIPTDSLAATGTRYQRQIRTDRVRPAPRPRTSTMSSASGHSRGHSRNLSTSSVGSNSSVYSNAADVRRRPPPLLMAGDNRPTTPPTYSHYAANSPADLTTPTSSTFPATPGSPSFASVLHSPTSVSSRPSGEMRTPARRLSVPASYNMQFQYASPYGPAYGPGPTVPSTGSSSIVTSPTSSAFPQNSIQISATDDWRRRTWHPSTYSNLNMSYNRPATSGLTYSQTPDAPQPAYAQNAMSAAGQAPRLPGIESFDHVQHRPTTPPRGNLTPNTTITFPPQPLLAAPDMDGPRRGHASHMSWDGSRPSQYPEIDDNGRPTTSWGQQTMARIDQLREAQARHQASGIPIMGPPSQGLSAPQHMAKEALQLESASKRVKRLGHYSGPGPTYRTSPDSSSSDGIPTPGISTAEIHPAIMGSQGYMEPQLLAGDTQHIPCLQPPMTASPPYRGHYAPQYRTIPPPERERRSSDLDRLDALVAVATGERTR

>CF317_000606-T1 CF317_000606

MLSLMLLLAAGVPLNAAVITPGIAAQPSSQSICDCTATVTVTAALPAVTVVAGNPSSIASSFSYAAGIDSIVYQNSKEDRPTGNPPETTSIPFTHTANGPAIIATDDPNDNTDLYHFAGTTTGPVATTTIWFPPADPVATESEIDGETAAASTSTDYASLDYDHDGTVTIITYLSTTTVPYVPSTTSAPSNLSITARDLQQRQTCSMIYARISGEWASWCNNWDGSTVLSYSTYETTTLITDVYGAPPVPESVWNPSHTTDSDNVVTIQTSPPEEPTSAPQSSTASGVTSIVVTTSIVVTYPLTTITETYPASSPTSTQGCGQTGQFVISFDDLPTYSTNNPNDTAVPPIFAPYDHFYWSPGYGYGSPPKTPYTSQVNRTNQIAIYDPSDETATVGPVEEGRQLPGSFGAGPRFGNSVYWFDAKSTYVGCNDTTTPCDIFVTGYRWYPGDSSDASKAEGHEVLAFTEQHTIPPPTCGELSCNMTQISFDAAKFSGLSTINFLADQGGANAGFYLDSFTATWTNSTCEAGLERVSSRK

>CF317_000607-T1 CF317_000607

MTTSNPPTNPRGIPSFPFLPSVTDYVKSPTEVESTLTRFQEMISKYQFMSQNVERRAAGLREKLPEMRKSLDTVRFLRMRKGEGEGEQDTDGLDESGVIETTYALQDTLYAHATVNTQELDSVYLWLGANVMVAYPLDEAESMLKEKFDKAKESLKAAEEDLEFIRVQTTTLEVATARVHNWDVSEKRKKKESD

>CF317_000608-T1 CF317_000608

MAVQETQPTSPVEGAGFTWLLDHILVYPGTYEIPLRTMYTLNSASHIQQGPSPKNVVGNAFPCTNGQHPSNMSTATAAAQLRANLMSHMTQQPAQPQSLPPSFITSFVRRCFPTDLAQVDFPQALTALDYLKDLEMRRRNEILAALTHLGVERADVENREKIARHYPNAYRWIEAMEEKERKLDYLYTHVYLGLRRWALVNEMSLKPFNKPNCLAMLNTLYPPMLNSSQFVPPTRQLTQQIVAEQRRHFFRYITAVEKSGAVVLKNVMSQHARAGEETGWPKVREDLDNYLRMTNSIIDECLETTGRSISPKSATFNHEEIAGEQKRKVDSGISFGSASGYTSNRSSSQSHLTRPSTSSSLSNHSRKYSDKSSSAAIEDEETVTLKPAGSALERIARELRKIGSRNDLKPRQIVNTSFEEVPATEVDSPPATPSRGLRLKRSLKNMRSVSRNRSGSASRPASRNGSYGRTEDMPDFDPEAMRRQREAWEAQQQHA

>CF317_000609-T1 CF317_000609

MPEAILPRDGSIIKPPGSPSHTRQASLKILSRNIDKVVLGNLLFDTWYFSPYPETVIFGLDHHSSKALSNGEQRNGHHERVVSPVLPKLHVCPYCFKYGTSPVDYVSHLQAHVKGIEANPDDWWPVPRTALKVYEWKGYTVWDVDGEQEKLYCQNLSLFAKLFLEQKSVFFDTAGFHYFVLTYSPVNEPLTPSAAKNRGRRRPLSGTDRDLGLRTQVMGFFSKENLSWDANNLACILVFPPYQHRNLGQLLMAVSYKLSGWEWEDSVIGGPEKPLSTLGRKSYLRFWSERIARFCMGQTTDAEGQRVFDKASKRRATFAKEDLTIREIGDRTGMLPEDVIAALNEMGLCEGVSSKRKKKSDDQQVLEPADETMPTMMLIKRSRVLEWAEKNKVDLAGPVKEEGFLGEWALSDSDDSGSTEPKVCS

>CF317_000610-T1 CF317_000610

MRVDFQNLALLAAILVAPTLTAADPSASDTSISGLIAQAKAARLQGNNAEALSLFDTAVKRDPSDYMNVFQRGATYLSLGRNSQAKADFDSVLKIRPGFEGALRQRAKIYARNAEWTSAKEDYVQLGKKGEQDLETVEEAEGAATLANVAEQRGDYEECVTQAGTAIITAGTALSLRQLRARCRFARGEVQEGVSDLQHVLQIHPGDLEPHLHISSMLFYSLGDTGRGLTQIKKCLQSDPDHKACKALHKEEKTITKTMDKLSNLLEKKQYVSASKLLVGNAAEEDPGLLADVQANVATAREAGYIHAKAGSELYSHLLEKTCECYVGMNNHQKAAPYCKEALELNPTSLYGLLHQAQLHIDAENYDAAVGTLNTAKENHQGERKVQEKLQEAQVLLKRSKTKDYYKVLGVSRDADEATIKKAYRKATKEFHPDKAHTRGVTKEVAEKKMASINEAYEVLSDPELKERHDRGEDPNDPSAQQGGNPFQGGFGGQQFVFRQGGGQQFHFKGNPFGGSGGGGGGGNPFGGFPFG

>CF317_000611-T1 CF317_000611

MDDAWWAPGKPGVNTGDDDSRAITYYSRATQLRRKTPIAAMSDDELPELATIILRPTERSPRKQKAASTSVNPRILADVPFLGVSSQQRNSPTKQLSPKRAPLKSTQARARKTVEDVPALAPYSRKAGSRSIASDVLLNEQGSDDSFTDLLQPLSKLSLQIDLENAPAAEKENAESKGRRRRAEPTSAMSRITQTARGKTNAARRDKPAPKRSNLYVSREAHCEDLNESSGTEGEDEDTDLSGFVVDDNAELSMYGSTEEVSSDGEDEAKRRRQRKSKPAASARRRLVRGRRKQLSDSEEDADNDQGLIEALDGMSLGRPALEKQKNAEKRKTLEVIDLTESSPVQRPKTPASDAESESEQEPEVPRRLLADPLSSFNTALKLQPSSKGQTQMLLPSKMGALPASPRRPERAMKQKEAPTTPPATPPRSPSKLKSPSKLLSPSKRNAEAPHTHHRQSMDAFWDHNVINEWHDTFSPKKAPTLSPRKNPLTRFNLYADDEELSKLEDSQPATNSSQDPFDESTDSLPSPCESPAKSRSPSKVSALKTEQNRIREEKKAKLAAKKKFDSEKGAIALDLLLALDTHITNSKISTMAASTGGIKVIWSKTLRSTAGRANWRRTVTKPSGSPVKGNPDSQTIERQPGVTVQHFASIELAEKVIDRPERLVNTLAHEFCHLANFMVSNIRDQPHGDSFKRWGSRVTSWLRGASAKTHASYRAAWKSCEVTTKHSYVIETKYLWVCAGRPADKQKQTLTQKMLNIELEDEEGCGAEYGRHSRSIDVEKQRCGRCKGFLAQIRPAPRAQASPRKSPVKRMLRTVRENEASGSENSSGAESLERLMQVVDLSD

>CF317_000612-T1 CF317_000612

MTSQVLPRELYGGAIVVALPSGAVDASDLRQIPDHQEVFLREKTLTSIIFEINEFQASETVGSAAPSIGHTAATNLPANSHVPPVDEAAATYHLQDLIAEPDYISPDGVETVTVKLPQTSVTDFPAYLSAATIITPEIDHSAKSTLPVGWQSDPVQKEYQTKTQQLLVRMKEHDTDFCVRINVPMKEFPHAQSEAALTEVAVADRVTKNIIESLDIKDFGLFGGGE

>CF317_000613-T1 CF317_000613

MKAFGAVVAAATLFAQAALADVPSIVIKGSKFFYENNGTQFYIKGVAYQQDYAPNGASTGNTSTSASSSSDYTDPIADGSECLRDIPYLKELGTNTIRVYAIDPTLGHDTCMNALADAGIYVVADLSAPGESIDRSDPSWNIELYNRYTTVIDSLAGYTNTLGFFAGNEVSNNVTNTDASAFVKAAVRDMKAYIAAQNYRTIGVGYATSDDAEIREDIGEYFDCGDSSMAIDFLGYNIYSWCGNSSYTLSGYDQRTAQFEDYNVPVFFAEYGCNEVEPRTFTDVPVLYGPEMDDVWSGGIVYMYFQETNDYGLVTVDGDTVSTLADFNYLSSQIAAVTVTGVNSASYTPSHTAAASCPTVGGSWAASDDLPPTPNRQLCECMYSALTCVSNDVSEDDVGDLFSTVCGLGADADVCAAITANGTTGVYGAYSMCNSTEQLGWALNAYYLAQDSSNKASACDFSGSASTQASTSPTGTCKSLINEAGAQGTGTVTSQPTGTGEAGSSGGSGSGSSSSSGTGNAAPRFGSTAVFGTGLAVAAYVSLAFVSGMCMIFL

>CF317_000614-T1 CF317_000614

MSGHPQQGGGYDGYGQDQPVQGGHPNDAYYQDGQYDDQYYDDRNGGHGAQQGYYDEAGYYNADASNPYQQDGGYYEGHQGYQDGYYDDQYYEQGAAGQQQHPGQGRRRGHDSEEDSETFSDFTMRSDMARAAEMDYYGRGDERYHSGYGGNYRPPSSQISYGGNRSSGASTPVYGMDYNNALPAGQRSREPYPAWASDAQIPLSKEEVEDIFLDLTAKFGFQRDSMRNMYDHMMTLLDSRASRMTPNQALLSLHADYIGGDNANYRKWYFAAHLDLDDAVGFANMKLGKANRRTRKARKAAQKKAKEAQNEEATLEALEGDNSLEAAEYRWKTRMNKMSQHDRARQIALYLLCWGEANQVRFMPEALCFIFKCADDYLHSPACQNRVEPVEEFTYLNDVVTPLYAYCRDQGYEIVDGKYVRRERDHHQIIGYDDMNQLFWYPEGIERIVMEDKSRIVDFPPAERYLKLREVNWKKVFFKTYKESRSWFHMVCNFNRIWVIHITTFWFYTSFNSPTLYTTNYQQQLNNKPKPAIQWSVVALGGAVACLIQIFATLCEWLYVPRRWAGAQHLTKRLLFLIGMLVVNIGPSAYIFFVAKGDGKIALILGVVQFLIALGTFFFFSILPLGGLFGSYLTRNSRQYVASQTFTASYPRLKGNDMWMSYGLWTLVFAAKLSESYFFLTLSFRDPIRILSYTEIRNCVGDGILKDYLCHYQPSILLGVMFFTDLTLFFLDTYLWYIIWNCVFSVARSFYLGVSIWTPWRNIFSRLPKRIYSKILATTDMEIKYKPKVLISQVWNAIVISMYREHLLAIDHVQKLLYHQVPSEQEGKRTLRAPTFFVSQEDHSFKTEFFPAQSEAERRISFFAQSLSTPIPEPVPVDNMPTFTVLIPHYSEKILLSLREIIREDEPYSRVTLLEYLKQLHPHEWDCFVKDTKILADETSQFNGDYEKSEKDTAKSKIDDLPFYCIGFKSAAPEYTLRTRIWASLRGQTLYRTISGFMNYSRAIKLLYRVENPEVVQMFGGNSDKLERELERMARRKFKIVVSMQRYAKFKKDERENTEFLLRAYPDLQIAYLDEEPPANEGEEPKLYSALIDGHSEMLENGMRRPKFRVQLSGNPILGDGKSDNQNHAIIFYRGEYIQLIDANQDNYLEECLKIRSVLAEFEEMTTENMSPYTPGIQQPKTNPVAILGAREYIFSENIGVLGDVAAGKEQTFGTLFARTLAQIGGKLHYGHPDFLNGIFMTTRGGVSKAQKGLHLNEDIYAGMNALIRGGRIKHCEYYQCGKGRDLGFGSILNFTTKIGTGMGEQMLSREYYYLGTQLPLDRFLSFYYAHPGFHINNMFIMLSVQMFMICLINLGALKHETILCHYDRDVPITDPLHPTGCANLVPIEEWIQRCIVSIFIVFFISFVPLVVQELTERGFWRAATRLAKHFASASPVFEVFVCQIYASSLYQDLAYGGARYIGTGRGFATARIPFGVLYSRFAGPSIYLGARSLMMLLFGTMTVWSGWLIWFWISCLGLCISPFIFNPHQFAWTDFFIDYRDYLRWLSRGNSRSHASSWIAFCRLSRTRITGFKRKALGSPSEKLSGDAPRARIGSIFFSEIVGPIVLVAVTLIPYLFVNAQVGVTGDDAVPATNSLIRVAAVAFGPIAVNAGVLAAFFGMACCMGPLLSMCCKKFGSVLAAIAHAIAVFMLLAFWEVMFFLEGWNFARALLGMIAVVALQRFVYKLIISLALTREFKGDQSNIAWWTGKWYSLGWHTISQPGREFLCKITELGFFAADFILGHMILFVCLPALCMPYIDKFHSVMLFWLRPSRQIRPPIYSMKQSRLRKRRVIRFATLYFFLLIVFILLFFGPLIVARLNLDLSSISLPLDLMQPTGQNNNDTFSSITGSALVNFGDGAAATGGSDDSDSGSGSGGDSAAATSDSGDVFSFGGRMAVRWATMA

>CF317_000615-T1 CF317_000615

MTTSTSTPSASNLTSAADYFSAQTLTLLNAFSEQFHKIPGSAIFLRYVKSSYQNDPIRSAVELFLVLFAIRYLLAREYSTKPGKVRLTEEEIDDLIDEWQPEPLVIKTTPFEDAELEKRAIIVGPTGPKSRLANGRTVTNLASYNFYNLVSNEQLKERAVQTLRTYGVGPCGPPGFYGTQDVHMKIEADIAAFLGTTACIIYAQAFSTASSVIPAFSKRGDIIVADKAVNYSIRKGLQISRSQVRWFEHGDMADLERVLSRVQKEQAAKKQLTRRFIVTEGLFENTGDMVDLPKIIELKLKYKFRLLLDETWSFGVLGRTGRGVTEHQHVDAAEVDMIIGSMAGPLSAAGGFCAGSDEVVEHQRLSAAAYTFSAALPAMSAVTASETLMMLQTQPDLIASLRENVKAMWQQLDPRGDWMVCTSAVENPIMMLVFKQEVIASKRLGLEDQAGLLQEIVDECLAQGVLVTRAKALSAAASGAKSDVYTHRPALKVCMTNGLSRKEVEKAGTTIRHAITKIMTRKR

>CF317_000616-T1 CF317_000616

MASKLSINSLSSKQHQPTDLKSGLAANPHNPSSAAYLAYPVKHVVSSLYRRMTEPPDQPVNRLLEAPAVPQKQTTMFTPQRRPSPFQPPPLTPLNLSHDLSESQADSLLLTRSLAEEIRLLVPPRLQLVDEWRLAYNLQLHGSSLGTLYDHCDKVLGALGSTKRGGFVLVVQDGSEAADVGSVFGAYLTDPPKPAQYYFGTGECFLWRASILPSLGSISAQSNVTRPNAPPSEDLLELAGLPPPPSADTTNAQRTTTVRGERRRSSAATSPTSPRKSFSSLASAQNGLRPASPIPAGSSTPDRIRFKAFPYSGVNDFLVYCQRDYLSVGGGDGHYGLWLDNDLEHGISETCPTFGNEPLSDEGKKFDILGVEIWYVGGS

>CF317_000617-T1 CF317_000617

MWAIKSLSKAIFGDTSKETILEIPKGQLYIVRPLSPKGYSELIYRDAVASIRRTTRDFEYHLVVQRAYEEGEQELEDSDDEAENLDKDEKTFLLDQKLRFRSAQREGGEWVLAWRDLDGDRGDLYEFVCDTSTSETDVNTFIFAAQECQYERKYRKSAQKATEQDMDEFVFGDEDPIPRASSLSGSLELPPTSKQAAEHMAKEVRDSKKSVKTTTPKTPKAIDAAHSQTEAPAAQKAPEPREILTQKAAELHLFDFESGTFIEQDPAIIATVSEIGQWQYWLQITGGHGKEWLGQPVVADISPVFNFEYLSFIFNHYSQDGSAYSWLLRFKDQAALEGFQEGLMRALWEQLNQMKWSKVQEQEKDYVIEAFNDLTMDEANAAEEEEEESEEEPDSPGRQSEHYDEDESDDDVELVDKDGNANSQLAVGSRFDRSFVVRGSKIGVFRHMPNKNLEFSTNISKIETPKGKLFSPTKVMLHSEDQNMVMQDSNNPNSLYRMDLEYGKIVDEWKVHDDIPVTNFAPETKFAQQTANQPFVGHSRNALFRIDPRVAGNKLVEQQLKQYMSKNDFSAAATTEKGHIAVASDKGDVRLFDRIGVNAKTHIPALGEAIIGLDVSADGRWLLATCRTYLLLIDAMQKEGKNEGKLGFEKPFAKDSKPQPRRLAMKPEHVAQFQHETGAPLTFTPAKFNAAPDGQETSIITSTGPFLITWSLKKVVQGQRDPYQIKRYSDQVIADNFEFGSDKNVILALPNEVDMVSRKAFKRPTRESIAGNASSSRLSAGAFSTPRKSTRGRQSHLRSEIVNSPY

>CF317_000618-T1 CF317_000618

MHISLAGLLLLAALSSAGPIDSSRRGNIPRRANDEHRRWVVNDHSISMQKRQEGTGAVAAPSSTSDPISTQVMPIPAFAAASASSAAVSELQTAQPDVVTVYSQTTNMVTVATTVRVTAASTPLDIASSSTSDLETSLANATASSSSELSSEPSSSTASIDTSASVETTESNASSPSSITIIFVTASPTFSGPTGGYTTLSETAQQNQSNGTGTVQTASESIVNGLSSIALSTSPSSTPTEGGTTTSVLSASSSTVTDDYTVLPGLTSIPISTDAGVSTTLATTVTEAATTQSTFTGADIFNGLSSIQALPFPPSASASDTSSSSAPALASASASASAVPSSQASETIVIPPQPSTDSISSESPASDTGVLTVTGSAAPSTIPAQASAEALSSAPAEASASTFTTDLSTGGLTVIPISLTGTLVFINTETASSTPSSAPSSSQSVFNSIFISTLGSEPSVTAAAASAEPEANSVVPTSLQTSTRIAAQSPPNESTTVQTRNHRHHHDSDSSSNSEATSTTFSTSTIMVTVTVDPATAVASATVSAQAAQDTDSSTSTDTEQRDQDSSSATTVDAEPQTPTTRTLVQTVTATESVNVPIIETVSSVVTETETATATITEAVTISQTVAVGATTVTESVVLTAA

>CF317_000619-T1 CF317_000619

MRHRIASQAVRQSSEVCLFCSLRWTSETSALREGPRLATRRAYSSSVTRRTPAGAAAARKEEDDEDEFPLLKQNAAAAASVWNKPKPVSRLGGAPPPPKAEPEPEPEPEQRFSIRGNQQDRPPFAEFAPRRDNRLGSRDGQQGRYQDQSSQPDGLRPRRDHRFGSRDHQQDASGPRLEDDTYRFRITPRSGARNSQQDAQGQRDTEGFRPRRGPRFGARDDLKPPSEQAPVRRMDRAFNNGSQNHATNEDGAPPIRKTGGGDSQYGAARNRVMEHDMQRGREQERSPFGRQQPSSERAETESWRPQVEAELSEQRQKQDSQKQRRDPLLDEIKKVEIPVAEEAEERERRTERSRSRFSNRFELEEDVEAELPAQKAAVVAKGNSYMGSGRKSSTDFDFDLFEAEQALNGEKRRKKQPKKRVVAEKQKPSLVIPEFMTVQQLAQSMKLKVEDLVKKLEEEGFEGARSDHMLDAETSAMFADIYGFEPVAGAEEMQDLVARSPAEDISLLPPRPPIVTIMGHVDHGKTTILDWLRKSSVAAGEHGGITQHIGAFSVTMPGSERQITFLDTPGHEAFLDMRRRGANVTDIVVLVVAADDSVKPQTIEAIKHALEARVQLIVAINKVDKHDANIDQVKQDLTRHDIVVEDYGGDYQAIAVSGKTGQGMEELEEAIITLADVLDLRAEQEGPAEGQIIESKVTNAGRVATVLVRRGVLRVGDFIVAGPTWGRVRTLRNDSGQLVQAAYPGDPVQLDGWRGSDPTAGLEVLQAENEQHAKDVVDLRHERAELAKSATDVVALNAVRSQEAEARAKVLAWEAENKVHRKKRSHHEGWIDAQASSGPKPVHFVVKADVAGSVEAIVAAVSAIGNHEVQAKVIHSGTGPVTESDIRMLATTGEVGYAISFNQPVEGAVHSLAYAAGLEILDQNIIYRVTEAVKDRVSAALPPLVTQRVLGEAEVGKIFEVTVKKEKIKIAGCKISNGTISRANQVRVIRNGETVYTGTLNSLKNVKKDVTEMRKGTECGLGFKDWDDAQEGDQVQCFEEKEEKRTLY

>CF317_000620-T1 CF317_000620

MVYSLSALKHAQYTSSWPNICPVTVHAVAEKLDTAVEPDNSIPHAYTNFGIFFCKNSHLADAHIYRALVSSGTALITSWHLLGYSKPVRKTHQALYPEEPAIVMSINDQWEDPSYVKGLLVKAGSDEEKCSFHSRTPSWDTQA

>CF317_000621-T1 CF317_000621

MDPPGCQDSSSNSVAIPELCDKDEVYRADMSQSSKATRQDLTQPTEQLPPPKRQRLTDDNLSSLPIRTPTTSRPPPAAGPSSSLFQVHTTTTNMSDGKNESRALRVLEIMNDFRTLQVHITSYVSRADANPPDQASYYLDGYVVLRQCCAEAQAILATHYNPGNLGVEPGNISDTEVQKATLQRIILDSSTRRFQAHKIYLRAAAGMRWILSRQQVSSGSLSPEKKARGLQALDARLRQELSNITDESVVNDLRVADRRKNYWLDEDPSLERMLGWIRRQR

>CF317_000622-T1 CF317_000622

MSGSAGYDRHITIFSDQGRLYQVEYAFKAITAANITSVGVRGKKCAVDKLIDPSASVDRARGEAAEFRYKYGYEMPCDVLAKRLANINQVYTQRAYMRPLGVATTLISLDDEVGPSLYKCDPAGYYVGYKATASGPKQQEALNHLEKKLKNKDCAPGDWEEVVELAITTLSTVLSVDFKKNELEIGIVGGPRSEDSDETSPEFRTLTEEEIDERLQAIAEKD

>CF317_000623-T1 CF317_000623

MASHVPAWKRLGLKLKYANEQPENAADSQNDGTLAKPKGHDASLDLSERPAKRQRLDKSPKINGSLTPDRAHAKSETYASGLGDAGQSAKADGTLKGSKQAIKRRKSVTFADDTKVEDGDSRVTIDFPAGSPGQTPKKARIPEADTDQADATGSPSPAIDGADEATPVRKKKKAKTGKRAQGNQRQSKDKSNPALEYMHQHRYDHGSWKFNKNREVWIVNRALNTEAIPNTHALALAGYVRGLPASAGARARLVKECTEALADARLEADSNDQGRTQILSILKAGSDEAEVQPFLQAHSRPAILLWALGENIESVTKSTPSSAVNGQATSEAPRKRKSRTSGPIDVSSSSEDESSDSSSDSDTDKGMESTTNGVKSKTNGAPAVVDDTSSSGSSSSEGEDQDDSSSSETSSDSD

>CF317_000624-T1 CF317_000624

MNTNIGPIHGQQILREAQQQRIARPGQQYTQPQVGASGMMQHSQYQNMPMPLSAGPQSPFAFDENSQHYFQAMHQLQEAAQMMPQTDRRMSQPDLRIQTAMRPYTPTHQLQTAHFPLTPVQTPMHQQTFQQTRSLQTSPRRQKQDDSIVSPRPMSMRKSRSLQGIAEHQEMQSFDVFGAPSLSATQGDVAEPEPVKQRRPAHIKTNSACSVQNGTGPLFGEVCTSTPDLEEQPQPILSSSNIPSSPAKSALSPRRMEISDLNLEPGISASIEETNVTLDDIAQFIEGPDLENKWTCKFEDCDKKFGRKENIKSHVQTHLGDRQFRCDHCKKCFVRGHDLKRHAKIHTGTKAYACLCGNAFARHDALTRHRQRGMCIGAFEGIVRKEIKRGRPRKHRPEMDERVDKASRTRSRKAQETSSHHMGSDPYASSASNCSISSWGSPPTETMDHLSIHGDPRSPMPTYDDSMNLFGFSSQGMDMSSHNQVEPANQVALPPDMFSFTPPASPVYSTGNKPSPHDYRELTPAELASFSDEINLTQKSEQSMSHATLLPPQHLSFPHQTLDESSQSSQAAFHPVDTFDLPSNTQVNHLSATHPTSLPALSHSSSPPPQEGPTLVFDYPEDMNPLRMSTMSNISNMTFGLSADTKAMISDGQSNVQDTQRNEFDSFLDYNDDSQLGMGMSDGDSFFASL

>CF317_000625-T1 CF317_000625

MAPEGWLDVHAHFYFPASDDEAKKLVKLFRETHFMVSEPSEVQWDPEKIIAYNDSAGVQMQLLSYLPSQHEKLRAANDYGHEVVKNHPSRFGLLAALPTDDANACLAEIERVGQFDEPKPDGYCTTTVYNDVPLSDPRLNPVWEHLNNEEAVIHVHPNAYKAGDYGKPGPLIDVAFDTAKVATDMLYKGVFRRYPNIKWIFAHCGGALPVLSGRLSLLGTESWVPNPEGLTREEIETQLANLYVDTAATAKTGLAPAIKMVGAGHCLYGADCGVPCSNARTMNENMRDVTAVEKELDIEAGTIARNTWKLFPAAAARA

>CF317_000626-T1 CF317_000626

MAEELRVRGAPKGAEYGGMDIEKLTEVQIKSVDAFMENRDGLIKLHNRKIPFQALYLSSPVVTAAISEHNKKHGFCMIQRVVFDGGPLAGYQDLFGWLESVIRHGKLVDTNVANENGPAALLRYWQFAQVAKALRVDHITKVVGPQILSLLIHRRTDLHLNVDVNTVRKVYEKDNEVIFREMRLCVVEYLAIAVPFEKLDWESELTEDTANLPGFKADLEEEIKRVDKDKDGYLADYNRYVRVIDVDAFVEERWWAKNWDLYPDDERARPYSGGGMFDPWG

>CF317_000627-T1 CF317_000627

MESTNILYVPAQADLYDCAFGAHNRAMGVYELNQSDVRGLQCGRRVDVGVDMGSGEVHKLRGRPTLRGLTMFFPAILGALHEHNKNYGGSIKLIVLPNHNISAYKKVMGWYYASITTGKVARFHRIYHDALTMYREVQFIATDLDAGYLVKAMTARTHAMAAW

>CF317_000628-T1 CF317_000628

MTTHVIVPVQTVLCQYLDTNDKLQHWKGPNPAQNGLSFNINATLSTEQSAVLRLQERGHQMPHQIITIPSAHSSYEVDLYPETLTVLIRQRDVNNKDRCTKVIMVTFSGDPDPINSNMAGNYGQSDFLRVSGARDEQSFHPARINQLDQNEVQALIQSNVVFAFVAGRPDQVPQLVSTTPVPLKALAIISPVINRALSAHNRNNPGTVNNVILPAGGTRAYREFLLWVQDVVGAGKLIPLRAVTYRPLTCYSELVFIANRLRISYLAAGLRRRVQGLLFCSVEKRLSIDPGDVRDAAATLPQGHWLIRMMVNAIAVAESNGWLHTKFAASLQELYGVVPGLKASVEGRKAEVEQRVNAA

>CF317_000629-T1 CF317_000629

MSGPSSKRPGDELPEQPSKVPKTNTAISKVAEIKPILDITDVPVRDIDKYITTDGSPSGVCSIEMLKHMEAHTARTTPPFVEYQYRLLAAQARYSGQLAQHACLNQLKFASMKPKQNEATPLAKRVLKFAQRDSIQARPEWRDALYYRVTIIMSYGSYYATPDIANNKYKELAELLEVPMFDLYEELTSASERHLARLADRSSIWESTYTAGPEPTARPPPISSSTATPMSSGPVDTVSDAWLDTQVNRLMRSWKDLAVDKSLVPIEEFDGISEEDKMTLLETHERRQLEDVNWKTETTNVRTLACYYTVLKVQERPTLVKDWCRHVKGTLITINITAQGPLRGHSERLIDMLFQKAAEIGKYEPVVIILDEAEQMLGSAAAGQDRNMQSLLRECISNIQEENVEIYFFAATVKPEEIDLANGWRRRLENRIHVQPLGETGRQALARRAFGENKVTLSTQEVESVAAKLAGFSAHDVNELARRSSSKHFGRLSQNTSWKEQACDGRLVLTPAQAGDKVFFAGSLEQLSQEDKRRVRIGSPTFQDVLELIEDGRNRGRWPSMKQVELDKHQRYADSQKC

>CF317_000630-T1 CF317_000630

MMMAFSRTSYAARLSSPAPPQSAYQVDGTSSEDIPTTPDGLHYLPTPNARFQISPAIADRVYAKPTVVGMSPPVRRKLYEGHMVKVYIDKPGESAKALPVPHHSIECICAHSPVVAAAIEVSGMSRAHIGNLVLPDGDEEAYIILSNWFNTVSKQGNLIPFPLVTKTPLSTYYEALEISRKLHLPVLNNLEGRFNHMLKSPWSFQSDDIQKAYTDYGQKHPLRTILVNGIIDAWSQLSERNYRRMKALGKAYPSFWCDLVARGKAIGEEIY

>CF317_000631-T1 CF317_000631

MAAAAPSSEYPSATTTPHGSNPSSRPSTPNLSTPAPGSADTQIKGSDDSSRLKLFISILRKFIGVADIANVRFSLPAQLIEPIPNLEYWHYIDRPETFVSIGKSDDELGRMLELLRFWFTKDLKFVKGKPCKPYNSALGEFFRCNWEIKEDAPSIASPMKAPPQPPPDPLKADKGNPIRISYVTEQTSHHPPVSAYWVECPARGIVARGYDQISAKFSGTYVKVVPGNHNKGLYITLKNRGNEEYHMTHPESQLNGFLRGNLYISVADVVSITCPKTKLKALLNYVEESYFGKSQNKVIGVIYRYDPASDKYTKPKDVPDNDVLIKIEGDWKDKVYYTIPSSKAAKSYPNLDPTKDKQLLIDIAPLMPVPKLVPPPDEQLDNESRKLWSEVSNAIHAKKFADATNIKQDVEQRQRDRATEREKEKKVFKPRFFQNATDPMGRPELTEEGRRVIEDMQRLNFHLEPRLDDSVPA

>CF317_000632-T1 CF317_000632

MDHDNNAGHYLDHVNNVYNVYHVYKLYHVYKLYHVYKLYHVYKLYHVYKLYHVYKLYHVYKLYNAYSILPSRDLCRLF

>CF317_000633-T1 CF317_000633

MTLRYDPPSDALQMSSINKLPVNALMGPPVPGFDLLPETGTWSFLLEHDSGRNLLFDLGIPTDWRDLAPQVADRLKNNGWEISVEKPTVDILKEQSIDATAIEAIVWSHWHWDHIGNPSTFPSSTKLIVGPGFKEEKLPAYPENPKADVREIDFKGRELQEITFTEQNALQIEPFRAYDYFGDGSFYLLDTPGHAVGHLAGLARTTTNPDTFMFMGGDLTHHSGELRPSPLMPLPSSIPSSYIPALCQHLSMTTACPGSIFENINSTRERTPPMTTPFFEPTMGKDIQVAIETIKKTQQADADENVWYVFAHDRTLRGGNVDLFPASANDWKQKGWREKLLWEFLADFEAAAKQMKEKAESEAVDTYYDEAELQKRLLGSKQRSISTYTKQATSSALDPGFQPRRRGSHDEYTQADQELLINVDQTVIDLLEREDKDNNCQITIEDNGPKNISVGTLASAGYRSAELRGNYVISNLLQELTLAQDLGKKVIKIHKSRLHENPVDRLSRRIRDEFWKNLERSLDAHSIQKAGTDPKDWTADPRPRIYIPHGCPEQHEYYTQVAKDRPEMRLDVRWLPEGGATPEFVRDLNEAPGILALAMRKEVVDGKETLTGEPFVVPGGRFNELYGWDSYMMAIGLLEHGRIAMAKSITINFAFCIQHYGKILNANRSYYLCRSQPPFLTDLALKIYDHIKHEPDALDFLKFATQAAIKEYYNIWTSEPRYDPVSGLSRYRPSGMGVPPETEASHFVHVIEPYAAKLNMSFEDYVNGYNAGTIKEPELDEYFLHDRAVRESGHDTSYRLEGIAANLATCDLNSLLYKYEVDIAEIIRLHFKDHLSMGADFQTEANRIDGFETSSVWDRRAKMRKARMDKYMWDEAAGMYFDYDTVKKQRTDYESATTFWTMWAGLASPRQAASLVLKGLPKFEEFGGLVSGTEKSRGEIHIARPNRQWDYPFGWAPQQMLAWGGLLRYGYTEEAHRLTYKWLYMVIKAFVDFNGVVVEKYDVTRELDPHKVEAEYGNQGSDFKGVPREGFGWVNASFVYGLKFLPMHMKRALGTITPWKIFEEKTAIRLDGFDDLTIQEASEESELACAEPFPLQSPQTRVTLELLHKAKGLLNMGALDQEEQAYQQEVAALKSWWQDSRWRYTKRPYTAESIAQKRGNLKVQYPSAVMAKKLWNLMEERFSNKQASPTYGCLDPTMVTQMAPFLETVYVSGWQTSSTASSTDEPSPDLADYPMDSVPKKVNHLFMAQLFHDRKQREERLSTPKESRSKCPEVDYLRPLIADADTGHGGLTAVMKLTKLFIERGAAGIHIEDQSPGTKKCGHMAGKVLVPISEHINRLVAIRAQADIMGSDLLAIARTDAEAATLISSTIDHRDHAFILGSTNPNLQPLNDLMIEAEQAGTFGEKLAAIEDSWTAQAHLKLFDDAVVDAINAGVHVNKQSLVDQYKQASKGKSNNECRAIAKGITGADIYWDWEAPRTREGYYRLQGGCQCAINRAIAYAPYADMIWMESKLPEFKQAVEFADGVHAVWPEQKLAYNLSPSFNWKAAMPAAEQETYIKRLGELGYCWQFITLAGLHSTALISAQFSRDFAKRGMRAYGETIQEPEMEQKIDVVTHQKWSGANLIDKTLMLVTGGFSSTAAMGKGVTEDQFK

>CF317_000634-T1 CF317_000634

MAAPPTDSFQSLQTNITESKAQSTTTNGNPQSRGPSPNRGASVPDSEAAAGNPVSQEAWLSEEGVDKSKRIMLKKLSHMRYQHEDLDQITVFLRDFGMKVVKKTDTERWYGGYGPDQYVYYVRQGPRGFLGGTFEVESHGELEKIVTTFKDRVQTNGKEEMTSAPGGGNIVTILDPEGFPVNFIHGQAETAPKQMPGKLLVNDESTKPRQRQFQRFQPGPAAVHKLGHFGLVSNDFPTLKTWYVQNFNIVPSDMLYVDEPNGERKNVAMFAHLDRGQDLVDHHTLFIATLTPESSIPHPHVHHCSFEVHDFDTQALGHQWLEEKGYKNVWGLGRHILGSQIFDYWWDTSKFMVEHYADGDLVNEDTPIGFGPAEDEGLAVWGPDVPTEFLH

>CF317_000635-T1 CF317_000635

MPLTAQDVYPGNEPVFLRHASRRSAVHSTGGMVSCTQPLAAEAGQRILRMGGNAADAAVAVAAGLNMTEPSQTGIGGDMFCLYWDAKTKKVHALNGSGRAAKNTTLEQCLAVTVPGAAAGWCDVIEKFGSGKLTMEEILTPAIELGEKGFPVSQLSAALWLAGDQQLRDASPNFREMLRKDPNAENFVRAPRAGEIMQNPMLAKTFRTLAAEGKKGFYSGRIGKAIVDVVKLTGGFLELEDLEDHMQRGSEEPEPISLTFTGQNIGNKRVKETDGSSSGRTVELWEHPPNGQGIVALMALGILEELEKERKIRTFSQTDHNSADYLHAVIESLRIAFSDANWWVTDPDHSSVKPAEMISRPYLADRAKLFDKTKAQPFSHGQPGQSPAQNHSDTVYFCVTDKDGNGMSFINSNYSGFGTGIIPQGCGFTLQNRGANFELSPEDHPNVYKGGKRPYHTIIPGLLTHGEGDKRELHSVYGVMGGFMQPQGHVQVLMNMEVFGMNPQEALDAPRVCIGAGMPSEGDVMDMTVYIEEGMDDDVVRALQGLGHKVQMLHGMQRSMFGRGQIIRRHVDDVTGHVVYSAGSDPRGDGSPQHVGKKLPDLEKRQPVSPAKRSGDFPRRQASKYDTEATQQYVVNGTGLPYVDFDIGESYAGLMDISNNADDGQLYFWFFPSVNPAAENEILIWLNGGPGCSSLEGFFQENGPVLWQYGTYRPVQNPWTWVNLTNVVWVEQPIGTGFSQGNVTATDEEDIAAQFMGFFKNFVDTFMMQGYTVYIAGESYAGYYVPYFADAFLNANDTQYYNLSSILVYDGVYSYNSIGEDIPTAAFVDFWGPTLDLNESFVEHLHNVSDSCGYTSFMENSLVFPPTGPLPTPPNPNSDDESCDTWNMVYEAASLVNPCFDVYQVLTTCPLLWDVLGFPGSFGYVPDGAFVYFNMTDVQTAINAPVQEWEECSSIDVFINGIDTSPPSALSVLPGVIERTERTILGHALADYILLYNGTLLAIQNMTWNGKQGFQQGPDQFDDFYVPYHEEYQLGTLAGAGVMGQYHTERGLTLVTVDLSGHMVPQYAPSAAYRHVEFLLGRIPDLGSMTPFTTMPDTSY

>CF317_000636-T1 CF317_000636

MEATSARMAVRHQFEGFPPPRGHLQRYIYNACDPISNLEPNLALNLEIVDLINQKKGNAPREAAVQIVQLINHRNPNVSLLALALLDNCVKNCGYPFHLQISTKEFLNELVRRFPERPPLRASRVQNKILEAIEEWRATICQTSRYKDDLGFIRDMHRLLVYKGYMFPEVRRDDAAVLNPSDNLKSAEEMEEEEKAAQSAKLQELIRRGTPHDLQEANKLMKVMAGYDTRNKTNYRAKAAEEVSKVQQKAKILEEMLEGFKEGDQIQEGDVFEELANALQSAHPKIQKMCEEESDDAEAVNKLLEINDSIHRTIERYKLMKAGNVTGANSIPKGTLGTTTGVGRNAANELSLIDFDSEPMQEQTPASTQAGQNGSGSLLEDSAPSASGKQTTVEDDLLGLSLGDAPASGAISLGGSNLSDLFNSAPQPAASPQPPQNVQAPAAAPIPAATQTARPNYDAFSSLSSALPRSKPATPAPGQQRPSHQHPAPSDPFASLLSSGSRPSTPSMSMQQNGGRPASGSFQPSGMAMAPATSSAAPATTADDDWNFVSSPPPQEPGLPQKSTLTVHDQGKLKVSLECMRQTNQGPPASAPIFVKASFTNQTNMPVSGLHSQIAVEKAYSLQLQPQSGKDLPPNQPGAVSQNMLISNVPPGKGTAVKIRFRLSYTLGGSQQEEQGMVTSLGIA

>CF317_000637-T1 CF317_000637

MDYTLQPGDRWPTEGPDPADEGEEHDGEEGDEYGEEYATSSATPIPTVTVTPTPAATSSAVSATDSHSGLAPGAIAGIAIGAAAILLIAGIAIWICGRRSRGRRNTEHDGGSWSLGFLGGKKDPSPPLAGTPATMPPAYNPHGIPVMTHASKSYPGASTSIVEHYGSPSGSPPPQMSPNYMQQNFPPHLLDPAAFNYNPYVATTGLTQYPPEQHYQFYRPEMGSGTPAPRSPPPPSGMSTATNIATGDRNSVSISGGTHSRGGSPDIVNEMNAISRSPGSINQGGGAAPNMQEPMPRRTGLGMDIKRIDLSG

>CF317_000638-T1 CF317_000638

MSSAGLASLGIPASLEQKAGTPRPPRQRLKSPASWASDGAMQHATDPVRRPPRPPLGHASAINATNDIASTNSTSNRFLGTNQRSWMTSTKPPSATPPVGPPPLPPTRPLPPYRFLSAVPETAPGHAARNNPDAGGQRGGPSASSLEQLPQEPGNAPRPATTGPSTTPQDDLEVVDLTLPQPDEHHHQHGPMQPEHALQPLANTNTTIASDLSASAAPQTRPQLLTPAASPLVQYNDQLHRKRPSLDASSLQHPQSRLRTESPIVSPLTRRLPPTTTWLDSTPQARGARQQPPAFIPSPQPVHPQSIPQQPLSASPTPGSQQVHSIWSPGNGSGPIPLQPYSASTIGMNAPLLGRGQSYLATFDSTIAEMSTRIDLSSRPRLRLPWIRDACENNDLFFLLLNQLLCMWHLSKELLFTLGIDTTCDAGFKILELLFASNSDLPKELLAFLAGWPNSTYSLKANAELNQWVMNTGQFLPQLGRHWDKFRQLCIQRRCPPTAREITDALQCPPSAVLPRAIFTSLARQTAPDYPPEFHNAAYQLFRVNQNDYFAAIFSRRLDTSLAQDYTAFATQYGHLLQQYWRKVSGAGVPSPRLPSLPALGVAQSRNPQPSFTSSSQRNFPGLATAPQNVQISNQGLINSVATGGAAPSPVNPLPADAAAQYARASVSSGSGVLNNSNQESSAAQSLSNSMRPLQNNSARTNVQSLSTAGSLTFSASHRTNQTQPGRTQIFRPNLPSPPAQLLLPDLRHLPPLLTNPLPEQEALHLAHLRQPQTEVKDVRPGETPRLYQSVQSLVLGPHPFTPGMAFFEMSFELLPDVVGKKAEIIQPPTRSFKLPKRILSTDSLLFNLRCIQLNTSQELPDESDWAALPTYYPQHIFISINDEYLEIRRKRQFRRDLPIDVTPFVRAGENKVTVSVHGGKNEEGKTFALGIEVIGLQDHSQAVKNATKIVFEEALKSIISSMQPATGEDDDELEVVTDSITLSVTDPFLSSMFTIPVRGRKCKHRECFDLETFLRSRASDNKDALTSVYEWRCPICGKDARPKSLVIDGFMQQVRDRLVEAGNTGARAIVIKRDGSWKLKQEADREASHGASRGSSKQPGKDHQKGEGRSSVASGQSAVMPPAMVSAAITSENAPPTASRVIEIIELDDD

>CF317_000639-T1 CF317_000639

MALHMPPHGQDSRNIQPGDVLLVVHDFDARSGDELTLRRSERVELIELDDDFGDGWYLGRHLTRGGTGLFPGVYTTKAPPATRSPFHSPSNSASAAATTHHAVTSPTTNPTNHVSRLSTNNTAPSLQPVQPVQPPRAELPADPQPENARSSLPAPHHSVYVQRTIGEALGAHDKDQDSPVMNETLNVIDEHITDLSTPRQSLAQQRLRRDSGSEYSSHVDRRSFVAGPETDEEDNAGADESTVRSWDHLQTAQHLRDIGVDPKHCDIFAEQEISGDVLLDMDQNFIYMKDFDFGVMGRRLKTWHKIRDFQRDVKSTSTRTSERGQNSSLEDINRPSQSQSQSRSVPPLHLTPSITEEPSDIPHPLQPSQNSKRTSWAGAASAASWRAVSGPDSLQRTNTATSRHSQRPSSIDFGAQPDLELSSVSAASHKKQPSTDRTWSITGSTQATTPASSIKPTAKPDDRLILPSPTPTPYDESSFDFDRGYFSGNELDNRKLRDRLSKTSHAGHSRQTSLVEQPSKRTSMMKRHNRLSSVDSVHEPANLNGTSNSAARPKLGRVRSTSLRATSFLPLAIGMAPSVTNLENDLDSNIGSPSAEKMRLQDRAKKIMGFRSTSDAVTSAEKTDSIKATPYTSTAKSSELLASPATGGSIPSTTPSIDIDTPEAEAAVALTKNNNRPKPSSKQRTSAYTHGLLKMPPSEARNNCDHYGWMKKKSSSIMATWKPRLFILRGRRLSYYYSESDTEERGIIDISGHKVLAANDDPTVTIHASVTGANKAGSTPKLESEASSLTESTVPSSLFYFKLVPPKGGLSRAVQFTKPAIHYFQVDSLSEGRKWMGEIMKATIEHDLSNLEMTNKQATISLAKARANRERPPSFNDTKKADKEKKEAEKTEKARTLQESGLGIEFADSAVNLGEDKVPHPAPPMTKPPSTLGMIPRASASQARLTEEAIKRLSRVPEENKENASSPVTKSPKSPNQRKSRGSTDAWGRKIFDFE

>CF317_000640-T1 CF317_000640

MAANMSTNLSTALQSPVSASVSASASVVSAVNDSNSNNNESILAAIGHVLYLSISFLPGFLVWLAAFTTITLPTWLFTTFNRTLTFTMNATTLLFLSLFLVSTVSWFIRYRFLNVYTRLPQEAHRKEPQIDLFPDGADNDSKPGLHQYFDDIFNAIKVFGYLERPVLHELTRTMQTKKLIAGETLLLEEEKGFCLVVDGLMQIFVKSARETTTASGEKLDLFDEDSGYHEGNQGYQLLTEVQNGASLSSLFSILSLFTEDITLKFDEQPSRPASAYDSANSQTPNMMQSTTSPSTPAHINNLAGYVSPRGTRNAKLATVPPLNLEAAFEEDYFHQPGVQFKRKERKPKSVHPDIVARAMVDTTIAVIPDSAFRRLTRVYPRATAHIIQVILTRLHRVTLSTAHQYLGLTTEVLQIEKLMNKYASYDLPNHLRAGALERLRDKFAKEIERLGSEDITKGIALHNPVTNRRRSSSGLRKEAALAARLASARKGSNSGANSTASDVNAGDLATNASTTRHLGRSGSMSFRNPPWNLTADLNHARAGGVQSPLSIREKSMIRPSFENGPLRRQDSGNEDNALRESILDCMVKALGLDVNTKDVNRKAGGVSVEQSPHLVSYDAKRQTAVFNNTFGFMDAYAGSADGDSESQVSTSVASFNGANARSLHDELIDDIEIVFFPKDSVLVEQGERNPGVYYVIDGFLDVSIPVEDKERQRSANGVPIRPHQEKRMPALRRTKTGQSRTASVPGFSHEQKNQVQKSLFLVKPGGVAGYIGTLSSYRSLTDVTAKTDVYVGFLPRASLERVSERYPILLLTMAKRLTTLLPRLILHIDFALEWVQVDAGHVIHHQGDDSDAIYIVLSGRLRTIVENADGSMRATGEYGQGESIGELEVMTEGRRPATLHAIRDTELAKFPRSLFNSLAQEHPSITIQISKLIAQRMRALTENPAGEDARLRRRAALSDTSTSSLNLRTIAVLPVTAGVPVVEFGNRLVTALTQIGVPNGVTSLNQAIVLNHLGRHAFNRMGKLKLSQYLTDLEEKYGMVLYIADTNVNSPWTQTCVKQADCILLIGLAEGSPSMGEYERFLLSVKTTARKDLVLLHTERYSKPGLTSKWLRNRTWINGGHHHIHMAFRLHSEALPAQPRRLGTAIKQRVQVLQAEIQKYTSRRIRQQPLFSAETPFKGDFHRLARRLCGRSVGLVLGGGGARGISHIGAIRALEEAGIPVDIVGGTSIGAFIGGLYARDAEVVSMYGRAKRFAGRMGSMWRFALDLTYPSVSYTTGHEFNRGIFKTFGNSQIEDFWLTFYCNTTNISKSRAEIHTSGYAWRYIRASMTLAGLIPPICDDGSMLLDGGYIDNLTVAHMKGLGADLVFAIDVGSLDDDAPQAFGDSLSGVWAVLQRWNPFSTLPNPPSLSEIQGRLAYVSSVDALERAKTTPGVLYMRPPIDAYATLDFAKFEEIYEVGYAYAKEYLSGLRAKGELEGLVTPWDRAGEEGEEAKLRRTMAPRRASI

>CF317_000641-T1 CF317_000641

MVLGVDDVSAIVGLASISFTTLQGCFKGFIVLSHARTCDREIANLGLRLLLTQIHLHLWAKEAGLLAAQPTLHVQPPLDRCVPKILKQISASLCDLRTLKQKYNLVLEETSEAITQVFSDDSALAKLKLRQLPFVQDLETNTTGLLRTSPKPWQKVRWVTVDGQQFRTMLDDVQSLINMLYQFLRTDGRTAFESRLDIPLRAAIMNNRFPDDLNLIIEASKESFVDALSAPARLRKKSLLSGLFGRRKSTTVDRLCSPQSARPAARDTVTNPSADPMSRQLKRLSAARLTLPEDIGPATARQLGYYSTTPVLVEWRDVQGIDWKKLEHRAAKFSTLLNEMAQYASFHSLPCLGFVKNGRTGQYGYVFDVSTSSSTAHGVPSSNAPSQSRPTLPMLQTLQEMLSKPALRPSLNMRVSYAVTLLETVLQLHTAGWLHKELRSENVLFIAHDFDPYSDHELLRSPLYVAGYVYARADDQLDFTEPLKSETDADLYRHPATCQGSLREPYCKSFDIFSIGCLLLELGLWRTIPDIIHRDMVKAPAASGNGMMKRETSVASLLTPPKTPCSSAGTIWSKNGEQELIARGKRELLTQFREDVSDQRGIVTLLQAQTGEAYTKVVTDCLNVRNKDADINLNKPNDDDDVNTGPDHLYAFDLEKRALDTLRGLLERL

>CF317_000642-T1 CF317_000642

MATKKVISVIGYIDVDLCMTTLRLPDPGESLQAKSYTSSPGGKGANAAVAAFRSSHVKEHHGDASSFVFSSPFPEVETEVRMVGAVGDDAHGAVAKDNLAQNGVNIRGVRTFPGEVTGVCFAMIDAETGENRLLYTPGATDRLLPEDFATPEQLGCGVRPDLVVSQLEVRVEAVERLLRTAHEAGIDVLLNAAPARQLMLEVYQWVTHLIVNETEAAILTGVEVEKVNEKTWHSIAQEFLEDGVKNVVITLASRGAYYANAQDHGHVQAEKVTVVDSTGAGDTFVGAYASEYLRQKCTREWRSATNWDIKKAVVQANKASAHTICKIGAQSTIPWADEVHIFARESTIDTLSRLSTDDAS

>CF317_000643-T1 CF317_000643

MAERENTWHAVLADKTVALARTNTTALSVTTDARFRAAYERLCDEHFQGRAQQYLAHFLRQHRTVVAFTGGIDSAGGLRAPDNLTSLLWASAYAVVETALRHKIELRELADILKPYRSSVPNFGADIARHFREPTVQRPLQSIYTEYLNCHETMIVHFATSSTPHNFDPIIERFNTATAIFQNAKAQWDKALRNANKALRNANQLEEDRRQMSTPSLPNLMNGLSHVPDSTNAHFNFIKELGNGTHGQVAEVQEATSKAFYARKTIRVRQGSQSARAAIEERVKREVNIMQRLQHSHIASVLFYVKGIDSFSILMLPVAEYDLRRYLEEVCSRSNRATLRQMDSWFGCLVVALIFAHNAQIKHHDIKPSNILIKNEQPYLADFGSAKDFSDSDNSIEPNDLIAGTPVYYAPEQLPWGRAADVFALGCVFSEMLTVRSGRTLQDYRDNRRDENKEYGYAFRFSLPKVYAWLDGLEDGLNDSLKIVLEQTHRMLDKDPARRRRLIDVKKTFRSDEAGLLYCNNCA

>CF317_000644-T1 CF317_000644

MPPRRRRPPRAGALSELPPLKILRSILLLQISYYAFAFILLLFTVLVFGQKFSIGLVFDWNNVKRDSTLGWLIAVVWLLVAFFTVIPLLLLVSRSKLVPDFALTIHLLHLIITSVYTRAIPTNLLWWGLQGTSAALLVSLGVWACRYREMQPISFPTLPTPNKTKDGKKTEANAGAGGPAYEMVVQRDVENQT

>CF317_000645-T1 CF317_000645

MAYGLPDWLTGSSKPEQPAAPKARDGGRVAPDRNSRELCYESRDIFFECLDKNNILDAIREDDKARKSCPQEVADYERDCARSWIKYFKEKRVQDWKRDQTIAQIQKEDAESARKAKERRKGFF

>CF317_000646-T1 CF317_000646

MAPSPLPPTSTYINIDKLTAANFTPYSYANDLVKSVNNASTTTSQQHIPSDALVDLTTPLQKTLFDLQEIDTSIHTLTSRSALDILQYTREQNVTAQRILERVEEERSRLVADFERLKGEVLGRYERAERARVGAERSLRVVGLTRGVQRVVGLARSFEAAVGDSGLGVVVAGREVHGELVRAANVVLEFRAAVSGKDASDLGKMTLVRQVRGRIFEDGEARVLDWARRVVREFSVSSLVSASGMGSTYKEAEEARGRFTSAVHVLYLLSPAPRLEGGRKMRKEEFEAEYLLRVLQGYLHSAVTSSAGGIGKGLAQLPLLERALLEASARCQNIIAFEVLLGGIAPPEHSLLQRKEKTKKTSKKLEDDELDELEDEFEELEVEEEDDDDDQGEENLLEPLLSSLDTSSLASYFWRSLASSLTSKVQEIINRGGVSVRTLRSNKEAIRTEIRGCVLRGSRMPATLMGHGSGGKEEVVGNWEREAAVMVGSVLGPLNK

>CF317_000647-T1 CF317_000647

MAKRSHQHSNSISISINAQQSQCVLPPRMSSLPKSFAAPQQPPPPVPLSQMTMEELMQILPRLDAAVIALDWVPCMQKRQQELKTWLQQGSHDPNSESSPSYLEKDLRKVIDESRDVARTYYKLFDVAKSLQNEDLQKKANELDEAHAQRSEATIALARYVSVHSDKCEQYERELAEFKDKAAQSVDILSHFIHQNLGEAMQSATQEQITEVLQTLFEMRSDTHYSSSHVSSDLYRDLQRTLARSNDERDGLRDVCSQQMATIQEQSKDLDQYIARMAKVINIVQEKERQNQSLREELTAAKEQCRVEKEGTAMRTQDDREQKSSPVGESGSDALHRSLVGEVWKRDAEITNLRRKLEKAYTRETGLQTQIRQLLQSSQGDHPDKPSRLKRFLTGHQKSSPNIPTLNSMQNLSHSVFTPFQKEKPPIQRSPSPSKSGKSSPMLGAFCEPGSNLDNLELPAAHQQEDSSPNVPPRMREAVRYPQDEIDSAVSNRFYQLPANVRSMASTPRPITPKSSSTSSNDEISDYRRGRPRYNSDPGVHESAMNDRFAHDRKPLLDHSRVLSGITEVTEDGGSFQRKNSSPDSMDKKMYLDNMHAAKALGGLQIG

>CF317_000648-T1 CF317_000648

MATPGRDNSSSASPTMQTPQLSSSSGSMDASKSPRLGQSTLHIQNPSSAAQHRQSYNDLRYPPSPRSTRQPSISSIAVQDLIDNPPHRTTPDSRFANRDWRTIHVAELSSPDDLKFIDINASVEEATNLLVESGAPVLLLRDPQSTESVVGTFDYNALNAYLLTAVGIAQPSEENAEKFAKMAQSAGQGKPIPVRDVRDIGRKDPLTFLPESASLPKAIEMFGRGVHRVLVAREGPEGSVEVTGLLSQTRLMRFLWENGRNFPVIDQLYSQYLRDLKLGSNNPISINGDRPLADALALLLDEGISSLAVLDNASNVVGNISTTDTKLLTKSSSLPLLRNTCIHFISVILSTRGMYEGKDSFPVFHVTPMNTLVTTVAKLVATKSHRMFAYGDDGSVVDP

>CF317_000649-T1 CF317_000649

MSISSAPKAPHFNITSPAQYVVHIEINRPNKLNAFYDPMWRELKGLVDFFSHDPETRAIVISGAGDRAFTAGLDVQAASNPETSALSSKGPSDPSRRATVLRRHILELQDVISSVARCEKPTIACMHGYAYGLAVDLSSACDIRICAPDTKFCIKEVDIGLAADVGTLSRLPKVVGLTSWCKEVTLSARPFGPEEALHNNFVSRVVPGSKKDLLEAGVELAKYIASKSPVAVQGTKNILDAAYARSVEDNLNYTAVWNAAMLQSGDMERAMLSGLKKRTPTFEKL

>CF317_000650-T1 CF317_000650

MDENQERLLDSPKRAHLQAQSSELRAALKTFEWTFAEQNGRKPKQNDIKNDPSIAAKYKQYHKVQDVLVGRLAYEKLYGTKPQRKSKSKVHSRQDSGVGSSPRAPQSSLQETPRKSSHPFELDPYDVPKTASPKPVLMNAIGPTPHRDGKVLGLFDLLQGSGSGTNSAATPSSSARKRKIDELYQDTPARRSPLKAIQTPSHRSGKKQGDLLQFLGETPRKSSGEDIGKHSRTPQSESKRFELSQFFATPSTQRFLFSSQDDGAAKRTPQRNTVLGRTPQKQLDVAGLDTTPTYLKRSASFKDRLLSATQTPTAPDFAKPNPATKRIGPPTLKHFRSSTSNIFTMSDIQPKSLQPRQQLGEPDDDEVHDDDLEALRELEGEDQSPHVLVEDSQLNMELPASGDEAVLHPTRPYKKKGQKRTTKKSTIRPVAHVKPSAQPKFVAADGFDEEEHRTDQDEQVAKTRYISDDEQASDFDDSADEENVAKSKASTKKGSAPKESTKAAKAAGKPKKQAGMINPNAQSHTNYRSLKIKGKSSNVKSAGRGKFGRGRR

>CF317_000651-T1 CF317_000651

MSLRLLLVWLRRIITISAILSTVYLYLYPVVYGCAFPSTSGSRRHGFDNTLAQHRGQIDDHDTSIAPFRLLVLADPQLEGDSSLPDPDDALWARIGFHWTRLRQSHQHEWKDLLAEVLKELALDDIPRAFWAVRKTLDLFGNDYYLAHIYRTLHWWTKPTHVTVLGDLIGSQWVSEDEFAWRGWRYWNRVFAGTQKVDDEIMSMYQQDTDKQTKFALGKDASEDWSRKAINIAGNHDIGYAGDISKARIERFEQTFGKANWDVKFSYPAERIPEARNTVGLPAPEIHLIVLNSMLLDTPALDQSLQDDTYAYINSLISQRLDPVESRNSTFTLLLTHVPLHKPEGVCTDAPFFDYWDFEEADGAFKNGGLKEQNHLGEFATRNGILQAIYGMSGDRHAPIGGKGRNGLILNGHDHEGCDTVHYIEANRQDATTEEEDLVLEPSGTPGKKRTFDDKDLSSWAWKAVRATEFASRKSGGVSSQPERDIVTSLREITLRSMMGEYSGNAGLLSLSYDFDAGNWDYQITMCPLGVQHTWWAVHVLDIIAVAWMMARLATWLYGRTIGYTDKTKTKPTTRTETVSEEPAKQAKKRASNSMVLTEAQSASKRRRKT

>CF317_000652-T1 CF317_000652

MAVMADGQVIFLGRFISAPGPDKLLIRQGAVLVSSTNGHGRIEKTSWTVKSSDEASKAFNVENASVVYAPINGFFFPGFIDTHIHAPQYPNVGIFGKSTLLDWLTTYTFPLEASIGDQGPAAEPTATHNGASHDETNGHPSKKRKVEPLSRAKTVYNQVISRTLSHGTTAASYFATISVPATNLLADLAHTKGQRAYIGRVCMDHAETCPDFYRDETPDQTIASTTASIEHIQRLDPQGDLLAPIITPRFAPSCQAESLARLGQLAQDRHLRIQTHISENTSEVELVKSLFPERKSYTDVYDHAGLLSDRTILAHAIHLSDDEISTICARNSKVSHCPASNSALGSGFCPVRKLLDAGIDVSLGTDVSGGYSVSMLDSVRHACLVSRQLGYVNGGDKKWNIGVTEGLWLATVGGAKCVGMEGRLGAFEEGMLWDVQEIELHSVDESEHAAAGHGPVDIFGWENWEERVDKWVWNGDDRNVNRVWVGGRLVHQRRQ

>CF317_000653-T1 CF317_000653

MARFGSLAVGTATLLATQVVAQAQITNDTAFYGQSPPVYPSPQIQGLGDWAAAYSQAQAFVAQLTIEEKTNLTGGYAEDNGCSGNIYAIERLGFPGLCVTDAGNGVRGTDFVNGYASGIHVGASWNKGLTYRRGVDMGGEFRAKGVNLQLGPVVGPLGRVAEGGRNWEGFSNDPYLGGKLVAQHIRGVQSNGVGTCTKHYIGNEQETDRNPTINSDNETVASVSSNIDDTTLHELYLWPFVDAVHAGTVSIMCSYNRLNNSYACQNSKALNGILKTELGFQGYVVSDWFAQHSGVASAEAGMDMVMPSGLAYWGPNLTEAVQNGTIPEARLDDMATRVMATWYYTNQDNGYPARGVGMPASLNAPHEVVDARSPAARPGLLQAAVEGHVLVKNVNNTLPLQKPRLLTVYGYDAVNPQFNTPTPGLSSWALGLTSSDVYSVLCGFSSAFAPCTPFLGYAANGTLYTGGGSGATSPAYISAPLDAIQARAMQDGDTVVYWDTQNINATGAVEGATDACLVFINAEASEGVDRPALYDSYSDSLVTNIADQCSNTIVTIHNAGIRLVDNWIDHPNVTAVIYAHLPGQDSGRALVSILYGDVSPSGKLPYTVARNESDYGAILSPVETPEEGSEYALFPQDPFNEGVYIDYRAFDAKNITPRYEFGYGLTYTTFGYSDISISKVSNARNMSAYAIGEIIPGGHADLFADVVTVSATITNTGSVGAAEIAQLYVGIPASGQPVRQLRGFEKIFLAPGASQTVHFYLQRRDLSVWDVVAQKWRMIIGGQYTFSVGASSRDLPLTGTLTL

>CF317_000654-T1 CF317_000654

MAPIARLASSRLMGSSRSSTASQRLSSISRQFSSTPMTAAEIRKLGVVGAGQMGLGIALVAAQRAEIPVVVVDNSQKSLDKGMAFADKLLSKDVSKQRISQEIADATKKRLTPSTNMQDLSDVDMVIEAVPEIPKLKEDIFSQLAQICPKHAILATNTSSISITKIAAATTKDPTDTSASSRVVSTHFMNPVPVQKGVEIISGLQTSEDTVQTAVAFCEKMGKVPSVSADKPGFLANRILMPYINEAVICLETGVGKKEDIDNIMKNGTNVPMGPLALADFIGLDTCLAIMETLHQGLGDSKYRPSPLLRQYVDAGWLGKKSGKGFYDYKS

>CF317_000655-T1 CF317_000655

MEDDNPFTKSKTIDPEVRAYVYSLVNALGGTGIDESGSYVLGDDALAVLRDLKRWLKLYDEKTNRLDVARCLAEANLVRGDLLPILSTWKENGKNGKVKMRTALACLELLVPLTWPVEHDEMTANHHRHTPYILQSQVKYKAAVLHWESTAVLRQSVRIALPALAMTRDERTPRDEGIIKLMLYFIRNIAAIRQVPNLPSQGLETEVSRSVTIEAFRAQDVFALLLTMCSNMGDDYNLMDVILLEIIFNLVKGVDAEKLWMNQQQRTKTGICDLRATLAAEQHMHMEAKKKEGSRHGRFGTMIWVKGDDKQMKTVSGQDNLKNHKTAMFNMDNNKKWNKPMQRRKDLDHNNYDFDQKETLSGEASEQLRNFAEEFLDSGFNPLISHLRKAIEREAERLLPVNYRQFFYVVAWFLKAERVRRERKHKDAKASKVVTEFDPESYGLVAAVLNQETFIALNRYMQMSWDEKAWQDLNACMRCFTEILLTVQDMAQSTLEEDQEIAENIQNRIFYEETTHDRIRMVLQNYKDQGLGYLDACTELSQVFLKMLERYSKENVDLQIRSKRKARRKRKDAQLKEQPAAQQSGEQEDNEDSEQEDILDTVQVSKERKFDFKRFAAKFVTQSSVDTFISLLKFYKDLSTEQLKRIHRFLYRVAFKQEQAVLLYRVDITALLYKIVEGPEGLSNKHALYGEWQEFTKQLFRKMFKKIDQRPELIVEMLFSKITNTLYYLEYGHDKQSSTSTRPPAELEVKASADRSRKEQIGIVVTVLLRDHKDSLVDWVKRIVNRAYDERFAWEAEADARKTEAEAAAVPAENENINQQEPVLDLPAAQPSYISISPENDETRIAMFKNARLKLLMRLVGLTEESNDKSGTPDATWILPSHIAASHLEESKAGIEQYSATQWQPENEDDNPEDFLRRVRKDQRASHHDEEIVGEDGEAKRDNFIDDSEGDEVLPEEEEFLFPDNPRQKKSVLEQLKAKRAAKSKKRKRGSDDEELDDSDDDAAKEQRRRKRQEAALARRRKHKSEAFIAASDDESDEEKDRAFFEREAALRKKYAQLNRAQIAAAAAAGDEEVDEAVLAKRRKTVARLRGEIEAEAGDEDVVMVDDQQDAGQNSAPPSSQPRQNDQSSEAESDEETPPSSPPLDEDDGKTRRQVLKELPQGKAQPLTKPMRRLSGKQRDVDVEVEMNSDSELEDEDDDVVPAKIVATQRSRGGFVIDDDDDE

>CF317_000656-T1 CF317_000656

MSYKTQPAFVHQGGWDAETDSHPAMKWMHEYTKEFDKGDHDAIWSSGKYLTSSFQYSKSDGTTYPPGQESWDQVKQTYAFFTKYHHEPYFLVCTVQRASASRSISAKMYGGGMTWHAVYTSPAPPTPPSLVPSNAPLTRSSQPDPPNTYLTVRTPR

>CF317_000657-T1 CF317_000657

MVHLATVSSPAAAGEKSAANRRRSTVPGLEHISLEAPGKDEYSTSIYGSSFAAQDLPYHEMPEKEMPKEIAYRMIKDELSLDGNPMLNLASFVTTYMEDEAEKLMQEALAKNFIDYEEYPQTAEIQNRCVNMIARLYHCPETGDENQNPMGTSCIGSSEAIMLGTLAMKKRWQNKRKAAGKPYDKPNIVMNSAVQVCWEKAARYFDIEEKYVYCTDKRYVIDPEEAVNLVDENTIGICAIVGLTYTGEYEDVQEIDRLLRERNIDCPIHVDAASGGFVAPFVNPNLKWDFQLEKVVSINVSGHKYGLVYPGVGWVVWRSPEYLPQELIFNINYLGADQASFTLNFSKGASHVIAQYYQMIRLGKHGYRSIMLNLTRTADYLAQQLEALGFLIMSPGHGRSLPLVAFRINPDDQYGFDEFAIAHQLRERGWVVPAYTMAPHSEKMKMMRVVVREDFSKQRCDSLVADVKMALSTLTQMDKTSIERFKKHTEEHSRARHGHKKGKAADHLQDNHSLQASSGKSHPIC

>CF317_000658-T1 CF317_000658

MGETREKKASKRSLKMCVGGKKTAVQLVTTPDPASHGVGTSATIEPSSPLPYDPSLVIVKHTPEKGFGLFAARRIREGTLVLAEVPTFRFRSEEEIGDDFDQRLRQRYDELPRNSRNGYLKLHNSKKVGYSKLKSIYFSNCYNLESPRSVHGGSCLGITASRINHSCVPNVQFSFEEEAPAWLFQSLHYDSSASEASGPNSIHSDVTTGTEGVMLFHAIKDIQAGKEIVSNYETAYLLAAGRQLKQQMYYGFICDCQACDGQKSNNKHWWSSDDRRREMITCKRLIDRAEAEWQSAQGHQRPERGAGDIELIIATLERLADLLNKEGLRGVELANTYKELGKWSQRVGDVEAAKRWGLLERKTCITAYGAWGRRVNEVYQRLDGLHGL

>CF317_000659-T1 CF317_000659

MPRSQQLKLIEDSVTIELKQGTALRAKRKLESPGPQDQGVDAATREAIEKYDHLDDDYVPQNEEGLAAKKPKTSRKQEEPSKLYCLGIYSPPEKSKKQSRKDWALVHCRKTIRQLPDPRKVSAAALRAAYESSKALNVVMQGELEYQADVASDLRKKGTRDLQKYQLRIDELQVAVGGVEPTKIPGSKWTDKARLSSFPKDPQADIVIKGRFESISTNLANEVGSLKYQLATEAYDKVAAERELEALKAHVKLHQEAAKQPSAALRNELGEHKEAREQAEKQLKEALSYRSKAESEITRLKEEIEDRLTTAALCHNKKYQLLEATFEGLKEKLSKQDGGLRSMEEKVKVQQAAITRAKACLMDL

>CF317_000660-T1 CF317_000660

MFVVVANCNFVPGKYDDWQAAYDELAEYVWQEEPTTMTYYFGLPIHFWTNSSATTHMFAFEIYADRQALYTTHFSSATMATFLSKIPETMTTGLDLSHYEDVGGFLDRYGDKQECEAMVDLRITCNPAMRVKVLEKLTLLAKDIAQPSLKGTLTFFVLKSLDCDQGIRVFQRFETWKALTEQTSSKSVLDFWRGSKEEILSMESQCYVPNAFMIAGANTDLLGRRWFLIMGQIICFLGHLITGTAQSNTHIIAGMAIEGFGAALCQMAAFALPELLPNKWRHTGVVLADLGVYFTIIIIPVTARYGYYAANWRGNFYAAAALQALSAAGLYFMYYPPAHPLGIPFGTAIKELDYIGMFLFTAGALPVLIGIVYSATYAAASPRVVATLVVGFIFLAIFALWETFGTAKHPLTPPRVFKRSFGRDFTAPCIALAIINMFYYSSSIIWPTAISSFYTVGGLPWQKAALYSLPQGLAITAGALGLTYCGSKIRYWQWQQTVSVTVMVVFGSLLALCTPDNFGLMCAFVVLSLFGYGWAIYLCIAFTQMGVPQEELGISGGLSGCVRFAGGSIAQAVYLTVMQNDIAKKTPVYVTRAALNAGVPQDKITQLLTELTNTKQLTVDFGSEVVAAVGYAQQLAIAHGIKLVALTSMGFGIVGIIACLCCKDVDAKMNNQIEVFLENDKFADRNKHH

>CF317_000661-T1 CF317_000661

MGADLAKDLIQAFFVKCDVADYNDQARAFTEVWNKWGRLDALLLNAGIVDKSSIYILKHRGKKEIPPAPNVATTQVDYLGVVYGTQLAIHFMRQNPTPGGQIVATASIAAVHGHESYPEYSGAKAAVMQFCRTVAPVLKVKENITINVVLPGIVPTSIIPQAMIDAVSAECLTPSSTIVSAYNMFLDDKSRTGQVVECSAKDRFFLPDPPLLNGRITKRATTVWDPLFKMMHGENSGLEEAIP

>CF317_000662-T1 CF317_000662

MPPNNTDTWRRAIYEDPEYAIQEIEKELASAHEENARLNVSLQDISKRRTQDRKSGAAERMRRIALDQEIKRLNSTITAMAAEKVMLSSKALSRDTAGNSWTDSTKAADAEERQSDEDGDVPPELENERLKSQLLLFADIRRENETLHLEKSSTAQMITDLEEKVDATSRDLKEVTKEHERTMGLSLTRMRLITQGDEERCKLRHRVDELVLKLNKYKGRDKKQDGRKRKLVECLKDERSTDDDLGDDEPQIKKLTPAAAEPATDAAVAKSARLT

>CF317_000663-T1 CF317_000663

MDSANGSYMHFNNSNDNDLSQFLHGVQDFADLDGDHQTHFDPALFADNGLVSFPQQAQPSRPTQSPFNQAQRQTHSQSPALPQFKPDQNTYVPQNYGQSAYNQRPLGQSFDPQLLSRPTPSPGPFDQYAYQPQHMNYGQPQFDYSYNAFQQQRQSSTPSQAFRPQVNQQTQHYMNTARPSPQPQAHMQQSQPSNNMSYPFPQVHQSQHRFVEPSMLNAQGQLHQSMSQVQQPRQMEASPYFTKAGQGATLDPRALQMQQYAQLQQAQGQNIQPRQQNATHPQSSMQSVMQFPAPGTGAIKGGNAMSGVERPVKSSGSVSDSDDDLDIEDEEPAETRPAVITIAKPMNDERGKLLWEVVDAVWTPKNKPAPPDKIRSAVRFVGEAVRGLRDKWKETNDKLKKAENANQPTEGLKVAVGTYREIMEALAGRVTNFGHPSILKRLGENLILLSAMYSFLVDRANAEDYDSPLIVAILQLGTKLETVDEDQLEKVKWTKVLVSRFAKKSTNASKSYAQQIILNAKKATARKRAEAAGQGPASPSGSSTGQIAGVKRAREGSEPLQQAKKAAVKPSSKPLSVQIAEREKKLKEKEKAEKAAAAKDAKPVGTNSANAAAVAPRPKATQALPPKASPFASLMSAKKRPGTTNAERAAKEKTDATKTAVTAPATNVPKKEPVVARSEAPVAKPFVSAAPSSSFLGSLFADMEKPKENPKAKVEIDLDETPEERAKRLRKESRRKLRVTWKPESELVQMKIFEHDSDEDTGHDDSQMRDVGDTGKEGEMLKHRITVEEDEDSDSSDEEPSMPPMEYFTPSEVDFSSMKQKPEDDFDTTNFIKGGGNVRPESSSSAAQDKYESGNLMRMYAAGEQPSSPTEPPEADDDDFSPVIDFGEPGEDRVRRREQRYLASKQAYQQPQPPQANGFNLASAMSQLQPQQQNVPQMDWLRALGMANQQPQQQPQSQQQNPQLDLSKILAVAQQMQQQPMYPGPTQPVPPPAASNATVTPDVAAILSQLSGGMQQPQPGLGTPLASGFGDNSNTFPGTSSSKPERRDKKEKKRGKNGVPLTDDGLPVNYKTKVCQFWLEGKCTKGNSCTYKHEPESI

>CF317_000664-T1 CF317_000664

MATRTQSLKDRQIASIERILSLNHQHSDEDGTAADGKAQQSAPLLDENGDPIWKVLVFDNLGRDVISSVLRVNDLRAQGVTIHLNINSPRYPIPDVPVLYLVEPTAVNVQLICSDLARGLYSPAYVNFISSISRPLLEDLAAQIASTNTAEHIAQVFDQYLNFVVAEPDLFSLSMGKETYYTFSSAKVEESVQEAAIDRIVSGLFSVAVTMGQVPIIRCAPSELAKLIATKLDRKLRDHVLNSKDNLFNSAQKQQNPYTQTSRPVLIILDRNVDLVPMLSHSWTYQSLVHDVLKMHLNRITVNNPDEKPKAYDLNSSDFFWARNAGQPFPNVAEDIDKELSKYKTDAENVTRQTGVTNIDDLDESGAASAQHLKAAITLLPELRERKATLDMHMNIATALLRGIKDRKLDELFELEEAITKQTADQMLQLIKSSEKGNNPLDKLRIFIIWYLSVDKEPSKSELVEFEHALVSAGVGDAVAAIRHISNVRFETRGTMMTTAQPVAAQQSSSPFGGLASLSKNFTDKLSSTGLGNAVTAVNLESLVSGVKNFLPANRDLTVTRIVESIMDPPAASSSAIAKTENYLYFDPRSAHSRGTTGGPAGAPGRNGPQSSANTTPSFGQRRQGYSEAIVFTVGGGSMDEYGNLQEWVKRTSGQTGTGAAGSAKARRVIYGSTELVNGEDFLAELVKLGSTG

>CF317_000665-T1 CF317_000665

MGKNSAKGAAENMARSFPNIKLCLLVGICGIVPKIKGQETAFGDVVVSTMVKQYDLGRLYPSGFQSKDTVDSSLRKAPIELGTFISYLEASDELHPRTIEHLAALEEKASRDYRLSEYLYPYCDADHVEDHLYKPHYIHRHKDSACNLCDTGSFCPVAAEKDCEELGCDTSELINRKRVKQIADICNATIDNPSKRRQELAELLPRVFLGVVASGDTVMKSGDVRDEIAKKHKIVAFEMEGAGVWDSLPTVVVKGACDYGDSHKNKKFQEYAALTAAACAKALLDIVQMGIPRNVEYGPDDVSQPTQHAQAQQLPAANTPRSNGGHVFSGNFIANNVEMGGTHTAQGDMYFGGSSRYGNRRS

>CF317_000666-T1 CF317_000666

MQDGRSRSFDIPAAPLKSARSPPGIQYNLLLEVTDANVTMAYPGAGGYGSHQHSGHQYGPPPQSYSPQPQYGQQQYNQGYPPQQGYGYPPQQPYGQPSYGTPPPQVAPYGYNNPPPPQQAGYGGYGQPPPQPNGYPGPQAGIANRYQNQGGPPPPPTSAQQFGHGAPSQYSFQYSNCTGRRKALLIGINYFGQKGQLRGCINDVKNMSTYLNQNFGYAREDMVILTDDQQNEMSQPKKRNILRAMHWLVKDARPNDSLFLHYSGHGGQTPDLDGDEEDGYDEVIYPVDFRTEGHIVDDEMHRILINPLKPGVRLTAIFDSCHSGSALDLPYIYSTQGVLKEPNLAKEAGQGLLSIVSSYARGDLGGMASSAMGLFKKATSGNAQYERAKQTKTSPADVIMWSGSKDEQTSQDAVISGQATGAMSWAFVTALRKNPHQSYVQLLNSIRDELSTKYSQKPQLSCSHPLNTDILYVM

>CF317_000667-T1 CF317_000667

MAAFGGQTPTIVVLKDGTDDSQGKGQVISNINACLAVQGTIKSTLGPYGGDLLLVDGNGRQTITNDGATVMKLLDIVHPAARILTDIARSQDAEVGDGTTSVVVLAGEILKEIKDHVETGVSTQTIIKGLRRASAMAVNKIKEISVSTTEGSEYETLRKLAATAMSSKLIHRNADFFTKMVVDAVMSLDQEDLNEKLIGMKKIQGGALQDSMFVKGVAFKKTFSYAGFEQQPKSFKDPKIVCLNVELELKAEKDNAEVRVDQVSEYQAIVDAEWQIIFNKMEALYKTGAKVVLSRLPIGDLATQYFADRDVFCAGRVAGDDLERVCQATGAATQSTCTDIKPEHLGTCGSFEERQIGGERFNLFSDCPQAKTCTLVLRGGAEQFIAEVERSLHDAIMIVKRAIKNKTIVAGGGATEMEISSYLHRHADKNVPSKQQAIVKAFAKALEIIPRQLCDNAGFDSTDILNRLRVEHRKGNTWAGVDFDHEGVRNNMDAFVWEPALVKVNAIQAAVEAACLILSVDETIKNQQSEAPQAPQRGLPPGAAQRALRGRGRGMPRR

>CF317_000668-T1 CF317_000668

MVLLWSLAGYERLAETLSLRMILLLWAAVCYVMVNHVFHHSLGTNIEKDQKTVYNWVHELQKTFMFLMIACAIIFFQGIILELASVTYIQGWMGPRSQRASDELATVIELQHLVNPYVSVDDIGFVSKICKKLFLPIDSNDLYYQISHGEGDEEMWTQYATKIWNSIFRRKQALTRFNVDQRFRDMNRDPSRGHDLFMQTDESCDGNVTEEELEKLVQRIGQPLNVRAQAQHGIQSLLRKLKAILSIVMLGIILCLYIQFFAKSVEKDLGTFWTGLTGLSFAFGGVLLEFTNSCVFVFGKHPYDVGDYIEAKGKKLIVNKIFLTHTNFEEVGDPDERGIVAQISHASLASEVIINWTRTMEAVVEKKKAKMQEDDRKAKKKAEKEAKEKEEDRDLILLRTARMRNADGLYRS

>CF317_000669-T1 CF317_000669

MRAGSSIATANKTIVLVTGGNTGIGYEIVKSLAFLRSDYQVLLGCRNTTEGEIAVSSMGAPANVNPIQLDITDDQSIENCVKAVEQHFGRVDVLINNAGTAGKDLVGTELEPTRRQVWQHVYNVNVISTAVFTERLIPLLQQSRDPRMIFVSAEIASIGKMLESKQPNEPQLVPFCSSKAAVNMMAVDYAMRYPEFKVNASCPGHRATGSNDAEMSDDKNPAKGAENTVRLATEANVQSATFTNSEGTLPW

>CF317_000670-T1 CF317_000670

MASNALRPSAILDHLRRIHNYNVDTFRRQPLSEISGSLGDLGTFLPLLIALASSSTQPHQISLSTTLILTGLYNIATGVLFGIPLPVQPMKAIAAIAIAKDLSRGEVMAAGIFVASCIGLMSVTRLLEVMNRWVPVPVVKGIQVGAGLSLVVAAGGKALGGLEWEGPTWADNRLWLGVTFLFLLACNIGPRMKRVPCALVVTVVGVVFAIVLTVQNNHRLPGIRAWRPEASVPNAAEWRVGIIDAGLGQLPLTTLNSVIAVVALASDLLPDVPTPTVTHVGLSVAAMNLIGCWFGGMPTCHGSGGLAAQHRFGARSGSSIIFLGILKLLLGLIFGNSLTGLLDKFPTALLTVMVIAAGLELVGVGESLNSTTRARDLKQERIEEIHENESKERWTVMMVTAGLLLAFKNDAVGFLAAT

>CF317_000671-T1 CF317_000671

MASTPSIETAAATPRRFAVSGIIALFVGKKRKKFEIHRDLLIAKSTYFQAALAADWEESRTKEVYWDDEDLETVSVFVDWLYRLLDTSQLRCKSLLFCYELAHKRDITGFKNDVMDAFRRRRADRKTRCHATMSIS

>CF317_000672-T1 CF317_000672

MSKDSVDLVEGTEMGSQQDFKGGSTNTHSHETAETNFIHEHTAGRYSQSGVTNIKSEAFRAALSDDWKKKGSIVSEIVWEDEDPDVVETVIDWIYHDTLPTLTTTKLDFNLCLMCYVFAETRMMFGLKNTIIDILRKDYVQHKHIIEPTEVRKAYELGLDNTQMGKSLLESTVYKIMGNQIKESCKAWTDQLVEACEDNCFAKDIVKEIVSYRKSAYGAPYEGEGCFYHDHADGSVCER

>CF317_000673-T1 CF317_000673

MKLLYPTSIVLDVDSLKGFPASLHAYDVKQPVPEDLADAEIMVTWTNSADNLKDAAKKMKNLKWIQSLAAGPNDILNAGFDKSIKVTTGSGLHDHTVAEHTLALLLNGARKFYEMRDYQLQGKWPGHLGGPQPDRPKGKFTSLRGANVTIWGFGNIAKTLTPHLQSLGANVRGIARSNGIRNGIEVYGEDKIGELLGKTDALVMILPGSESTKNALNKERLGMLPDHAWIVNVGRGTSVDEEALANALDQGTIGGAALDVFETEPLPEDSRLWKTPNTIISPHAAGGRPQDAEALIADNLRRFCAGQELKNVI

>CF317_000674-T1 CF317_000674

MLSSLESSFTLLLLLLPLLSCALKFDLQAESRPQERCIRNFVNRDTLVVVTATIDGNRGDGQRVDMHIKDAVGNEYGRPKDVTGEKRMAFTSLADTAFDVCFTNTMTGRSSYTPSRHVELDIDIGADARDWSAVQAAEKLRPVDTELRRIEENVNEIVTEMEYLRMREQKLRDTNESTNERVKWFAFGTMGMLAGLGAWQIDEFTQGEIVTIFVGSKRKRFNIYKDLLRAKSPYFAASLKFCWDGDMGETYPDEDPNAFGHFTNWIFRETIPAVDLGTLEGLAAITAFYKLADFLLIEDLRNVIMDSILKYLKDNTWDLNFFSLSFLRERNLRTTPLYRLALRSAVRMYIHCPERFEEGAANDIEFLIDKPELMMDILEGVREYNKKPYDQVWKCHRCEFHEHEDSAICKR

>CF317_000675-T1 CF317_000675

MSGDRSKRNSVSRPNDSKSKDKSKVQKDAKKGKDNSGDEEMTVVVPPSKTNDADQNGDVTMDGTADEKEEAPIDPKVKAMNDIKNNLVLLERAVSQFDPRFTLRVLRSIPSIRKQLTAELLSHLIIETYATPTKTSFALLKSLGDEEGFPRSEADAWAKKKDTQHAAQKGQGPPKEVLPEVDIYIAILLQVYFYDTKNIEAGAEFSQGLVKHLRTLNKRTLDSLAARVYFYYSLFFEETKPFPPSPNAAVVEIRQTLLDALRTATLRKDQDTQASVTTLLLRNYLSTSHITQADLLIQHSQFPESAANSQVARYLYYLGRIRAIQLSYSEAHEHLIGATRKAPTSYKASGFYQISTKLLIVVELLMGDIPDRAIFRQPSLEKALAPYLQLVQAVSSGDVSGFQKLTEKYSSTFRSDSTYTLILRLRQNVIKTGIRMMSLSYARISLRDMCIRLGLDSEESAEYIVAKAIRDGVIEATLDHEHGYMKSKDVGDVYGSQEPFDAYSSRIRACLALHDESVKAMRFPMNQHRLELKNAQEARERERELAKEIVDGDLDEEDGPGGDFEGL

>CF317_000676-T1 CF317_000676

MLRQIRSSNRDAGRGGGVDIEILLQGAERLCAAYQTPGTMDRIAALRQRHDKISTSIRSYEGQISQQQSSMNRFNTGSVEEDVAFDANDEIRMIFNEQDLAAEDAAIKELEAKKKALEARVAGMERDLGGLMR

>CF317_000677-T1 CF317_000677

MAKRAHRLNGLDVAQMYPERSSSFSSDGRSNSTRSASTAHTVPRAGLSPAYIAQAEASDLLSSELDRKVWVTLPALTLLNEFLDHVLYSILSISHSVSLGQLKTAVPVVLKPRLGKAALRVAEEELKEYIEDEEAEELYSKGESEQPRRDFDTDLVWKLARLRCMVYARMGDLEEEDEEEWLEKEHLLEQAAASSLNARQSMAVTPGSAIFLTSVIEYLGEQALYYAAQYAQRRHDNAQINNETTATPENHITDRNSDILLEGKDMNHSSEQLNATVGTLAGALGAVGVHYVTKDRKPTETDQTHELAEERSEPYERSLANASIKQPRDLDTMYAPEGHQSSGKQEQQRVDEMSTLETDNYHGRSKNDTDPIRYPEYSERSEGLHEMGTPSIHSNFRSIVMKPITTLRLLRRLVSWKLMTAHPPINIGMGATHQHQEATNQTVQTPQSSSNNVTSQKLPARRSKNPLGEARDAQVPRTTNVMDLADYVRSTGPSSDDQLPKAITEGPPREPGDHRINRHIAPFRTTMDSDDLNALAPSLPKNSSDTNGNTTVNSNTPLISNAARVAPSQQPQIQAAPLQQTAVHDRVTVDREHNMPRRTRRRVKDPYAIDESDEEALEHTTPNQPQEESLVDFLRNTAPSTSMTAQPILASPQSSKSLNRAASVEKLKEFVRNRSTTSSSDRQEHLRAQSRARAESPHLTQNGSKLDSYRPTQPTHAKHVDRERASKAGYRAESRTESRAESRLESRAPARTSSATADLADYLRNTGPPPSSEETQPFSFSNQQTDGVQRQEGGLRKFFSRRGKV

>CF317_000678-T1 CF317_000678

MPSPTDILSDPEIAQAYDDVRSDKSPTTWMVLKYESAMKDNLKLDTRGEGDIAEMCEALGQDEAAYAYVRMKLGNDEYSERVKFVFVVWAGPDTKVMRKAKMSFQSGQVKQVIRTYAVEIQTSDKRDLAADAVTMKLRKAMGANYDRQSSAY

>CF317_000679-T1 CF317_000679

MFTTTTTTTTTTTTTATNFRKTYMDNHENLGAPAPARGDQPHDEHDLSPQLPEGTTWEEHETLTNAALLADLPTDKPNIGFDMEANNLGHNSELSYLQIRDYHNNVTYLVDLLVLQKAAWKTTGADNATTLKTIFEDPKRTKLIFDCRQDSACLYAKAGVKLRGILDCQYLHMLTMDRYPTFRLGLVAAVKQMAGLSPQALAAWIATKTSQRSHGIWEQRPVPAECKAYAVGDVEILRAMYDTAEAMLEPYALDLGAQWSAFEVGRTWCSAEDYTTIAGRTWEGFAQCWASKLLKDGIVPHAAPTESVDAY

>CF317_000680-T1 CF317_000680

MKTTLIAASALLGSATAGVHHMKLEKVPLDQQFETASVREHVRALGHKYSQKFMNAETEDIFKHTAIDLDGHYDVPVENFLNAQYFSTIGLGTPPQDFKVVMDTGSSNLWVPGSECGSIACYLHSKYDASSSSSYKKNGSEFGIRYGSGEVSGYISEDTLRIGDLKVKKQLFGEATNEPGLAFAFGRFDGILGLGYDTIAVNKIPPPFYNMIDQGLLDEPVFAFYLGDTSKGTDSVASFGGIDKNAYEGKMIKLPLRRKAYWEVNLDSITFGDETADMDNTGAILDTGTSLIAMPTDYAELLNKQIGAKKGFNGQYTVECDKRDSLPDMTFTLSGYNFTISAYDYILEVQGSCISSFQGIDLGGNIGPLFILGDAFLRRWYSVYDLGNDAVGLAKAT

>CF317_000681-T1 CF317_000681

MQNLSPGGRITNILPFLRHLPTFLSTDKHNEAMRLDLEDRVWYGSFDKAKVAYEKGTLRPSYAKYYFDHREKTGLTEHEALHGLAMMATVSILTLIAPLQRWLVAMAEHPEWQKSVQAELDEVLQGRMADYDDSPKLPVLRATILESIRYVSPLPTGIPHRLEADMQYNGFHLSKNSNLLACDWSMCRSDDFYKDANTFNPSRYLDPASPQYREPLTEFPKIQGHTVFGWGRRVCIGMEYAATQMLIVCAAVSYAFNIGIAIDPTTGEKAKISIENATANVIPILAEEAPLLFHPRSASQAQRLRDIYKLQREQDEEKEEEECF

>CF317_000682-T1 CF317_000682

MSPIHNLDKGKERIPYGREAIRRSFGSETTRNIWSFDAPRIEANNKAMTEQHVSRREQLASTQSEMALQQQVIECKAEENKRLKKDLGVIRAEAYEARVEAYEAKQLLHATEGNFCDLLQETEELRLKNEQLRDQNEQLLAHLQGWMKMANAYLRPSKLVDQAKTV

>CF317_000683-T1 CF317_000683

MTTWILGEKFNETYPHLSGVKALWETKWKFPCSKSVYPFHHGKYEDFEPIFQKLIEDNVNDAYTDTYTQYFLPTARRLVDEAMSVQGSDRKKAIELYERAACVFRISRFPSVDGDETGFKRGIFHEQCQVYLRGASLWNTPLKEVVIPHTAGDGQDGKEIPLFARVPNGASAVAKCPVVLLLTGLDGHRPDNTGRTDEFLSRGWASVIAEIPGTADCPAERRDPKSPDRLWTSILDWIRSQPAFDTSHIVVWGLSAGGYYAIRLAHTHHDQLIGAIGQGAGSHHFLGREWLSRVDYHEYPFTLSHAYVKKYGYKDWEELLDKAQNDFSLLNSGLLNGPSCRLLLVNGTWDGLMPIEDSMLLMNYGKPKEGRFYDKMLHMGYPPANECVYPWMESVMASR

>CF317_000684-T1 CF317_000684

MRVRLPRNISDEILDLGNTTEDLPLTQPTPMSYPLQRVRMGEISRNIVDTLRFGISGVSEHDYGQIMVLDKRIQHFLEDLPVFLKLDNESLQKSQYILERFPYFAMQRYIINIGAHSNRCKLHQPFLVRESPKTKYTESANICLTSAMEVIKINKAIRHDPTHYIPEKVKLVGLLHHMFLATVVLVMDLCFNRMDNKDDLRSKEVANAIKMLEEAKDYSVSVQKFLDSLMEALKKHQVGLGDDSRKTAETTQKTQNSGPAMPAWSSTTSQQQAAPALQGPSMGNAAQPPTLDIDWAWKDVLDQGDMNSLPDWDQLFSELDAFIA

>CF317_000685-T1 CF317_000685

MMAVGDGSSEPDGDTASTPELVEPRPEDARPITPQHSDHGNDSHKADNDEDPDEGEEDEEQEEEDEEEEEPRLKYAPLTKNLAGVYRNGDATSSFFVAGDKLIVGTHNGKIHVYSMPMLHNIKSYGAHTASVSSVSMSPFPPPLHLPTKLDATQRLAAESADATSASSQASPNAKNSPRQASINRSPSNDIYIATSSIDGHVCVQSLLDPKDVQLRNFGRPVQAVALSPDFKSDKTYLSGGQAGSLVLTVGGQSGKSVNASTTGSAAAASGWLGSIGLGGHNGTDKVLHSGEGIISTIKWSLSGKYVLWVNEHGIKIARSHLKLDNAETGLEWKRISHVSRPTRPGWEDLAAVQKARAEWVDRSSLDSDQDPALQREPSNGGIKASDAAGVEEVVVGWGDVTWIIRVHPGDGGSMAKAAAPAGRAEVISVIRHPDCMIAGVSLYTPTLMLILAYMEKKSASSKQSNDSTPRSRSKRHNALKPELRLIDINSHEEIETDSLPVSRFESLASSDYHLGVLYPTKIPAQLAQKGYLGHVGTGMAAVGSGLVTGTEVVAQGMWDATMYGPRMLGANRLFTSDTGSIRNGKPAADRSPGPAAKTSNYLTGWIPGMGSSIFGNENEDLKAVATTQGMKIFLMSPYDCIVAVKRNLTDRIQWLEQQEQYQKAWELLDEHPEAAGSNSEPSEASIPPTPSKTSSMAQSTSDSVASPSRQAQKPTLAEFFADSASMFGSTQAVEKDRYSTAEKEKRRIGELWLQQLTKAANWTEAGEVASKVLNTTTRWEHWAWMFIRNKKFDEISPYIPAMELTPPLSPSIFEVVLGHYVLTDRKRFQELVDIWPSDLFEISSITSAILEQLSSGDVQKDSDDWRRLQECLAKLYLADGHYREALRCYVRLQDAETALSLVKDHHLVEAIGDDIPGFVLLRIGRQQIKTSSRDDLEEAASEPIKLLVDEASLGVVDPDEVVEQLNKPDLRIFLFFYFKHLWQGTGHGTAKTASRQRHRFSTAKLEADEGKLLVDRYPELAVELFAEYDRDLLMEFLQTSTLYDFSTALRVCENRRYIEEEVYLLSKTGSLKKALFLIIDELQDVSKAISFAKEQNDRGLWDDLLDYSMSRPRFIAGLLAQVGTAVDPITLVKRIPSGIEIEGLKDGLKKMLREYDLQDSISSGASKILSSEVAINMEILRRGRRRGIKFDIPPAPRKAVRDDSQLDNTIMPADAESGPKPGHCARCDKAFIGEDSEMLVGFACGHVYHTECLFKDENKAPLPRTEQEEDDDLGYGYTRSIATKVTNARLLRDRIKLAGGCKVCRDRQAQIDVVLQ

>CF317_000686-T1 CF317_000686

MAPVTLRPFSNAAGSLIGASCRRGAGTGTFDLSKQPGLRPLHASPIFQRPRRRDFFSSNALLANSNQRSETSVKTDADAAAEILQARKRKAGGAAAAKTSLRRVAVEAQRSKDGQLARSQPPSHATPAPKLVTAYAVADRFDLAKVVDLLRSKGYEPDPLSTGLYPQVVHVQIPISSIYRSTNPAARDLSSSEIGDVFIFPSGTVVTWALPEGFTSYLATRSLLPAAEQPHKEPIETEDLEYIEDSSREHSAIRGETIILGTRLSSSSRAHFDDHPPDHSQTQTVDTVLTKIAFSSGFARSTKLAILETSLSTYLASTSQIPQLLARGSRLPYSISRRFILRKTGELLLLRAQLNLYSELTDSLPDLFWDSRHELNLESYYDQVGKALDVGIRIKLLNEKMDYASEIATVLRERLSEKHGLFLEWTIIVLIAIEVGFEMLRLWKEGFWTEVFDQNHQQKLLSTGERRPLLEERPQ

>CF317_000687-T1 CF317_000687

MRSVQRLTQLANHTFKQSDNMAPSADTSFAAHVQERFASYQQDRQASSKDVIYATSNGVPMPHPYETQRVGENGPLLLQDFHLVDLLSHFDRERIPERVVHAKGSGAHGFFECTDPMPDLSYADLFATKGKKCPITVRFSTVGGESGSHDCARDPRGFSVKFRTDDGNWDMVANNTPVFFLRDPAKFPHFIHTQKRDPSTHLTHADDSTAFWDYLSQNPESIHQVMILMGDRGIPDGYRFMHGYAGHTLKLVNKDGDWVYGQMHMKSMQGTKFITQEDSANKSPDYSQKDLYEAIQRGDYPKWSVEWQTMTPKQAEELWETQKINIFDLTHVWPQDQFPRRKVGEFTLNENAVNYFAEIEQVAFNPAHLVPGLEPSADPVLQSRLFSYPDTHRHRIGVNYQQLPVNTSKTSYQFGNFQRDGQMAFYNQGARPNYLSSIDPIKFRTRTTDLDKTHGHFIGQAVSFLSEIRPEDFNAPRALWQKVFDEPARERFINNVAGKMEVCKDQEILKRQIAIFREVDDDIAVRLEKATGIKGYDGIANLQFNGTHNGMAKDASLRAANGIETNKGKSISDNNGAPAKGTHKGLNN

>CF317_000688-T1 CF317_000688

MASTPKNPLTTTPSRANASPRPNLGALGNKTVAAKSPAVKTPTPANLHGHGHTHKISMSSHPTSTPLAASTLPDDLMNLNTPAAQLMASIGPTNLTPLPHPSTQDGLGISTGIIGAPSTQDGPASKNPQKERHDRLKDIAQTLKGRTQARGISRRNVEDLARINGFELFYDEDEPDILNLFGEKHVVLDIVFKGHQSDVVDKARLKLNDSANAEEEVVQEGASQVLTRNLTEDSNDRLPWHDLTDFSANLEYLTQVERVSTAASTSCFKVIDGLYDTFQKIWIEEKKRMKWRHDLHHLCQSNTGEPVKDANRRLGLMVKYWTTGQRFYTGQGPEIHAVSAKDSDYTATFSVESGSPSIAASQKWLAEDTLASTVRAEDIFQESSVDKPSWQDPASSQPEPANNADPMDVDPSSTLAKTLDMHFVCTFEPEVLLPGHVIQSLRGTGQMLVVRSEQGTTYQQLLHGNETANLKPGARWTRTQYRFNKSGNHEVRTHSYNLYTAGQYYVYPVHKLSFSHPRQFADALPVLRQYALVNALIQSIAPLRAAAPDTPTIGSIFTSLRGEGNELVFRDGKQLRVRSNKSRLESTLKSVMKPKASVVPVTGCPLPIDVQLDTVSYATTTKTCRLDISVPISETIFPAPATRRLRAKKFLKFEIEILLNGIVEVVNVVGVELERPKLDEFKKCFSSIIRCSEGDLGSAVAWAITDLETS

>CF317_000689-T1 CF317_000689

MADPSTCLPLTPDSIRQTHDRIKAYIHRTPLITSRTMNRIASSPDPRAYLADDPPSIWDDGEMNGSGSGAPRFNLYFKCENHQKIGAFKARGAFSALTHLASPPGADLPGIGIEALRQRGVVTHSSGNHAQALALAASTLDVPAWVVMPTISTKSKISGTKGHKGVTVVFSGSTAPEREAVVNEVIEKRRKETGGEGPVLVPPYDHVDILLGQGTAGYEAEEQLGAMKRSDRICSGAAEGGHGAADGGREGEVEERFDAVLTPLGGGGLLGGTATWYSRTHGSDKKILVFGCEPSFEGANDGERGLAAEPPKRIEHVKTLTIADGLRTPVGMIPWSVVSDKEKVEGVFSVSEDEIKMALKLMMERVKVVIEPSSAVPVAVILFNQRFRQLVAKKQKEEGAGRPWDVGVIISGGNTTVEGLSKLFSEGWNKFEAERQTGQVGIDGSKTVEDVAG

>CF317_000690-T1 CF317_000690

MASKSFARTALRASKQKVAAPAVPKRSFVSAPASRPVVSRSTRAVAAPIQQQTRGVKTVDFAGHKEQVFERSDWPREKLHEYFKNDTLALIGYGSQGHGQGLNLRDNGLNVIIGVRKNGASWKEAQQDGWIPGTNLFDVDEAISKGTIIMNLLSDAAQSETWPAIKPQLTKGKTLYFSHGFSPVFKDLTKVDVPTDIDVILVAPKGSGRTVRSLFREGRGINSSIAVYQDVTGKAEEKAIAMGVAVGSGYLYKTTFEKEVWSDLYGERGCLMGGIHGMFLAQYEVLRERGHSPSEAFNETVEEATQSLYPLIGANGMDWMYAACSTTARRGAIDWSSRFKDSLKPVFNDLYDSVKDGSETKRSLEYNSQPDYREKYEQELQEIRDLEIWRAGKAVRSLRPENN

>CF317_000691-T1 CF317_000691

MLKVWSMKQKQQQDAQAQGDGQKKKKVTAAQLRIQKDLSELSLGSTMRTDFPNPDDILNFTLTIEPDEGMYKGGSFKFTFAISNNYPHDPPKVKCTQKIYHPNIDLEGNVCLNILREDWKPVLALNAVVVGMQFLFLEPNASDPLNKEAAEDLRTNRENFRRNVKTSMTGGSVKGITYDRVLMK

>CF317_000692-T1 CF317_000692

MAYNNQPGGYPLQDQGAAYGRPPVDDDEAERSLIAGQTYPGHYDDHSRNLTQNDSVYNLQESYMSEAKGPDVSYNNAPDPYAGGFPQAGATPNPYVRSDSTEAWRARQNQQTIKRYATRKVKLVQGSVLSIDYPVPSAIQNAIQPKYRQDLEGGSEEFTHMRYTAATCDPDDFTLKNGYNLRPAMYNRHTELLIAITYYNEDKVLTARTLHGVMQNIRDIVNLKKTEFWNKGGPAWQKIVVCLVFDGIDPCDKDTLDLLATVGIYQDGVMKKDVDGKDTVVHIFEYTTQLSVTPNQQLIRPTNNDATDLPPVQMIFCLKQKNSKKINSHRWLFNGFGRILNPEVCILLDAGTKPGPKSLLALWESFYNDKDLGGSCGEIHAMLGHGWKKLLNPLVAAQNFEYKISNILDKPLESSFGYVSVLPGAFSAYRFRAIMGRPLEQYFHGDHTLSARLGKKGIEGMNIFKKNMFLAEDRILCFELVAKAGSKWHLTYVKASKGETDVPEGAAEFIGQRRRWLNGSFAASLYSLMHFGRMYKSGHNIIRMFFFHVQMVYNIVTVIMSWFALASYWLTTKVIMDLVGQPPGADNPAQTKAAFPFGNTVTPIVNTLLQYGYLGFLLLQFILALGNRPKGSRIAYMISFVVFGIIQAYVIVDAFYLVYRGFTSGNGVVLNEGAEEFFTSFFSGGGAGIIIIALAATFGLYYVASFLYLDPWHMFTSFFQYLALMPSFINILMIYAFSNWHDVSWGTKGSDKADVLPSAQTKKDDKGKSAVIEEVDKPQADIDAQFEATVRRALSPYNPPPEKEEKNLDDSYRNFRTRLVSTWIFTNALLAAGITSTSLSTFGFTSTATQRSSGFFSTLLR

>CF317_000693-T1 CF317_000693

MNTLQRRCVECPSETPSCPECPNGQTCSLVPPSCDVCASTICIKENQLPGQITETKHSTPVGPIVGGVVGAVAVLAVGLFCLWYFCIRSRKRKDTWDPPEKRDQSNLARNGSRAHSIASTVLTRASNVIQIAYIPGVTGRSPPVSPSVPPMPIISGFNTASSTPQPDTHFFMADDLRNSTWSDTSVGPRISLAPSLARASSATTVYYENAIVPPVPAQHAFRAQANMVSVKQTGSSGTSTASTATAARASPLAQNGQLGAQILKPVTINNSSIVARNVTARPIEVRKTPSNTRVPTLGNLARASSIKSGRNANMFDEKEVVVSPGTDRLDSPIAPPSVLSTKPSDMSLGGASASGISAVIPAEGFLSRQSPIQRPDSGGLTAIIEDAIKNASADSWHSPRPGAHRQDSGPFSDIHELPSSTP

>CF317_000694-T1 CF317_000694

MESLTRQMFAEDPLESEGRKDAEFLKMEAELFAKLTRAGVSSVSTTAMMKLPGAAIEGLLGWLGDKNPFVKNASAQENEVWILDNTGFRASDRSSWRAEVVACFFQHGRGDITAAVAAIADTIGLDGEPGHQAATRALIAERLKPFVDAIAPARTLPVVVKSDEHDPRKCRLGPSNTSGISSQVFEVGVRGQGSGEANLITTNPSVSGLPQARGVTRLYAPEGWGVISDIDDTIKITQTPDPTGILRTTFAEPAQYTSHMPDFYKILDEQLQKPPWFYLSASPYNLYQFLHRFIADYFPPGTIMLRDGSWMTLGGLFSTLTQGTKEYKTSRVDKIHDWLPNRKFICIGDSTQTDPEAYAASYKKYPGWISAIYIRKVTDAPFMEKKNKPGRFEKAFEGVPANVWKVFVLPDELKDHVTQVVGEAHMSILSQLSAYSCGNDRGRQQLDKDKVKH

>CF317_000695-T1 CF317_000695

MPYAFDKCPERPASIEQILNGLDRYNPETTTVFQEYVNQQCEEKFFDCYASLALLKLYQFNPHLIHPETITNILVKSLTVFPSPAFSLALALLPPSTIPFGNTSGAAIPTTELTESIQKLTRLSTLLESAQYDVFWSTLNSDDVYADLYSDVAGFEDLIRIRIASEVGKTFREIDLRVLSAWLDLRGEALTKFVQTACGWSVSGEKVNIPSNSENEARAEVKGERVGVEQFGRVFRRGMEAPA

>CF317_000696-T1 CF317_000696

MPLPAYSTIDSVNLGALARSKVARECNRPERDLRLLLGHIRILDVLEQSDAELSDSSEDESNQQSDSQERPGAQPRPCATQRTSKDAEEDDLADLFVFEYDGKPRVAMATAVISVSEVAVED

>CF317_000697-T1 CF317_000697

MMRNKHPMQSMQKTYDECYLICSTAVYFESRNNEAEALRSWREALNQIHQHNATKLPAAWTPKTETERALWDALKQMENQCKERVDLLKALRQSRADAGEREVHAPNNLSTVSLLANNGSSTWLGHDSVPQAQYSDIPSSSSSAAAARQPIPLRRKSSGSGSVKDHPALSAPPALPKPTSKESRSPSPDHRKPMRTTLRSEKKGFRNARSNQARPQTLKAAGLAWDTTVPQQKPPPKAGVDPTIEARLSAQAARRSLDLPRPDREQRTNNVVTMPDPADPSARSNSIAAHASQLSYDARPPIDVPKRSYNKSAPDLRSERLDPEKQLSSPPQPSSKKVPPPPPPPPHRIKPAEMKYRREVFPTTQNVPASRANNILNRAYAQPDPSPASEPKHTGLSRKPVASPSQPSKTRPPVPRARRSSRDDLPTSSKAQHSRPRIDRAQESGTITPPSTDEDSSPEDPDAPAKTEFDLRVEALLANLPKGIDPLFAAQIMNEIVVKGDEVHWDDVAGLHLAKKALKEAVVYPFLRPDLFSGLREPARGMLLFGPPGTGKTMLARAVATESKSTFFAITASTLTSAGTEYHFVDEIDSLLSSRGGGTEHEASRRSKTEFLIGWSDLQRAAAGKDSSVGDASRVLVLAATNCPWDIDEAARRRFVRRQYIPLPEPETRETQIRTLLKSQNHRLSDQDILTLTILTDGYSGSDMTALCKDAAMQPLRHLGEALLTTPIDQIRPISLEDFRASMDNIRPSVGKKGLEQFEDWAKEFGERGG

>CF317_000698-T1 CF317_000698

MSSSSEVKRSTSASKKLQKRHGNPHRLPSYQLPSRLRFSEDVQEDVAAPTRGNGAPAQYMNQSIFGMIAAAGSRTDFNQRFDESSDSDGETAVNAERLSGRRPSKKGTKAVNKAQPPEDDLTPSDAKGSKRLSLHRLTQSVPKLSLKTYKLRQPTQPRDLGQSGEQSTDEDTGDPRDAPMMSRLLTAEAEFEATEGAVEEQGTLPTAAERARSRAESPATLLATRLKEIFGFERPETVVAEFPCWLLQSVLLQGYLYLTRHHICFYAYLPKKFNAATKTGHLSKRGKSNPKYRRYWFELKGDVLSYYSNPSARYFPQGNIDLRYGISASLIEDKDKECKDFSVTTDHRTYYLKADSAVSAREWVKTIQKTIFRSHNDGDSVKISLPIENIIDIEESPVVDLAETIKVRVVENDDSYAIDEYFFSFSFGLDVMRTLRTLFENSSSRRASMDLLSPTIHDGGSASARPPPGQRMPMQGTRNASPSPIREGVRATLTPYTRGHDGKMTPRMSGEFSRSGHSPARVSLDQQAEYGRQSFDRGRRSESTSRLEPGRNERRQARSPLARPAEDSHDSYVAASIEKETSSSTAPVSPAADADMSASQILSRSDVFHAPPVRRMSTLVSDDTEDENKTSEDDQGPVPKPGNGRRIALQAEAVSDHPTASSIIPRTASPTLQDIVKAGAYPLQRAGAFAGYLKNRSEKMSKLLTTESMSYLEKVSGMWAGQRKHYGENENVVPDDRDVDPEDEEANVGHGDRFRAHFALPSTEKLQATYFGFFHRVLPLYGKVYISNNKFCFRSLLPGTRTKLILPLKDIENVDKEKGFRFGYHGLVLVIRGHEELFFEFSSVDNRDDCTVTLLKSLESVRYLAESGLLSQLEAEAAEAAKIEHIMLQEARRGSGTADPDILPIAKDGTTLVGEETTPILFDDGKASIINFKPPTGLRITCLTIGSRGDVQPYIALCKGLLAEGHKPRIATHAEFGPWVEKHGIDFVSVGGDPAELMRICVENGMFTYSFLKEATSKFRTWIDELLGSAWSACQDSDILIESPSAMAGLHIAEALGIPYFRAFTMPWTRTRAYPHAFAVPEHKYGGAYNYLSYVMFDNVFWKGVSGQINRWRKRDLGLGSTNLERMQQNKVPFLYNYSPHVVPPPLDYSDWVRVTGYWFLDEGGDYDPPKELADFIRKARGDGKKLVYIGFGSIVVADPAALTKTVVEAVQKADVRCILSKGWSDRLGDPSAQKIEVPLPPEIFKIKAAPHDWLFKQIDAAVHHGGAGTTGASLRAGIPTVTKPFFGDQFFFGSRLEDLGVGICLKRINVSVLARALWEAVHSQRMITKARLLGEQIRSENGVQNAIQAIYRDLEYAQTLIRARKIQDQDAVESSSATNNAQSGCREPAATGDQDEESWTFVDDLDESCHKPIPGAVHTLAASKRKS

>CF317_000699-T1 CF317_000699

MPTRFRTVEDLTATLGKLARYETLLDDIFPLVTDSVRSMIDQARLQESGPLDGSDAAESATASSGSTFDVDVKSGSPLGGRLPLPLPVSLTQSSSSSASTQRTFPPPPMFRSSLSPTRSGLSIPRSDKSPSDDISSNTAPRLPSITRHGFLVGGALCSAPSQMVGTAGFETKRVVEKRSEGVQSKPPRPG

>CF317_000700-T1 CF317_000700

MNGMSNMYDQAYSGLESQAPMDGYPQDMPLEGLDTSMMAHDHQPEQPRVDEKAADSLPASISSHPDGAQDQAQEQAQNHARRASMIEFGSYNNGDLTDFEFDPAPSHPAMSTQFTNPALMPQKPLDPRRVRSREDLSLDTSFPQMPDFQAGSTSAYPASMLPATAMGMDGSNGYMPHNVSIEGDFDNMPANMSATTMPSSQAQQGMYTISPVAANFPMQYPNTGHDIGGGTPDNKNLMRMHQGTAPNASSPQRFFGRSQSQIRRNPMMPSPLSVSAQNSSSSTMASPAQMPSTPTIAQNQQSQSRRGSLDMQSNSYPNPASDNTQMDQGQSASHFNASRWTNAYSATGFDMLAVLMRVATRRNPQISIGPVDLSCAFVVCDCQKHDLPIIYCSDMFERLTGYSRHEILGRNCRFLQAPDGKVQSGIQRKYVDDKSVLYLKNQINKRAEAQLSLINYRKGGQPFMNLLTMIPITWDTGEYKYFVGFQVDLVEQPNSVSAKNPDGTYEINYNRSALPAYRLPAPDPSSGLENMGGQTIPREEVSQVLATIGNGETDLSKRIWDKILLENTDDVVHVLSLKGLFLYLSPACKSVLEYEPSELVGTALSAVCHPSDIVPVTRELKDTTNGSPVNVIYRIRRKNAGYTWFEAHGSLHTEQGKGRKCIILVGRQRPVFALARNDVVLTDGAGDAELWSKMSTTGMFLYVSSTSRSMLDRTPDDLVGTSMQTLMRPESRRKFGRMLELARMGERSTFKHDLQNRRGQVLQAQTTIYPGDAKKGFKPTFLLAQMRLLKMTRSMLLQQKNNTSIPRSDALSDMGPIASTPQPIAPSEPQEKSSPQVGGQSPSAQDAFIGGSGILTMAGSGGVPIGSQDEALASEDNIFDELKTTRSTSWQFELRQMERQNRLLAEDLQTLLQRRKKRKRRKGITNLEKDCANCHTRVTPEWRRGPSGQRDLCNSCGLRWAKQNGRVSPRKSSMSDKSAASPGHTGNVQQRATGNPSGGSKENSADVKPGPASMAEESSKTPTRLTPSRIKNERDDDAEASNMPTIMEEGPEPPQSDVLPT

>CF317_000701-T1 CF317_000701

MEALDRSSSNDLSSAPSSDVEGPFLSRPPSAHSNTIVVSREGPGPSSSSTAANLKSGAAPPSATRQESAPVKQRKPRKKKEVDPNAPPASEKEKPPRKSRPRPATTGNARKKLKTEGASASAILPQHSAQITDLSSSTLVQPASFVSDTKPVLNEAAKTTQNGFSNQNPATNSQPLSPSMSRYQSTPQPQAAAPRSRNIFDPVRGIERASEHTQTVSYPNSNATPPRPAVFKPSASPAISSIINHPNVQEPAPSAFQPSWSSTRPEGPVPTNGTDSTPVKLDGAADSEMSRKTSPPAEKAAAEAEAKPKRAKEQPPPMASGSGLLNSTFFGGDLSSDKAEKGGKGVSIVLNLELQKGESKIFNFARMAEEKYGFAAVYPRQAAQKERLAKVAAAGAALEKSASNSKRGETSLGESGDEDLSVDIDRDSDNDGDVNMTGVNGTNENSGTDGPARKQRRKRRDEYDADDPFVDDSEMLWEAQAAATKNGFFVYMGPLVAETDKAPADKNEGASKRGGRGRGRGGGPGSRGGRGGAASAAGTEGSSRGGGAGSRGSGITRKPRITKAERQQRELEKKQREEAASALAVKTPTAST

>CF317_000702-T1 CF317_000702

MAVHGQRFELNLDADNWQFDVDENAAAPSPFTAVKEIKERELSGVPAAPTLKSTKTGFPEHRNRRAQSAFKQQKQRDTDSNNAVHQPGPSDRAILNHAAKKHGVDLASSKQQADIDAENRSKINHMSLDEIEEARAELMAQFNPDKLQAFLKRANVSDEQHQQQKEWDEHEAGRAVDPSKKSVAFAEPPSPSEETAVEDNVEEDLGEEDDDNDTTIKPHPPDGPSSSTTHFPAPPRNKSDYKPLDPTSASFLSDLKEHYFPDLPHDPSSLSWLQDPTEEEQKASSYNPDRTGFAPSALRFGFNGMLIPPSESLEIPTTKGLHHHGDAPSSAGYTVPELALLSRSTLANQRCVAYQVMGRILYRLGRGDFGPRGHELSEGLWTCIERERVVEVMMADANRDKGHVSAKAYATEALWLWRRGGGGDRGVLKQGETRAK

>CF317_000703-T1 CF317_000703

MTTNTVSFDTDGAGSDGNSTRRGNALSNKLTRVLSSSYADVEIRDALRLYDGRYAHGQDEVDRDLDLKYEAQKEVIEANARIVDDFAKVAKQLNRVGSLITTLNQTCDDIRKHVLAAKQETAPMLEEASTLLAQKQETEKKQQLLHSFCKHFLISDEDLTTLASSAGPVNERFFDLLARVKQIHKDCELLLGYENQRLGLELMDQTTRNLDAAYKKLFNSTLRSFKGLDLEDPHISGSIRRSLRALSERPALFQSCLDSFAEARQSTVSGAFQKALTDSTMGSARAIEFSTHDPLRYIGDMLAWVHSATVSEMEALEGLFISDADEISQGLHSGKSADPFSMPTEGDEEDTAFDGYAALNSLISRNMSSVSHTLSQRISVTVRNLSDPVDIYKAYNVLCFYHDMFFKLIRRSPQTAPASLQENAILSTLANLQSQTFKHFETTTFENLHSSIDDEPAADLSPPPALSETLSQFTKIAQTRGPNLDTAEFAKLYNTLLKPILDSCNALSDDLQSPAADQDEPPAAPPLIYKLNCLSLVRDTLSSLTAGPSPIEAATPPLEHAQAEINTLSSTLTSQLSRTFTSTSGLDELQHLIATDAAPTGTATQSSAHSQKKRLLRSSEARFPSSSAAAPADQDPGPGSALESLAGTLDAFLASALMDAQDELGKLVDREVAKHVIRHAVEAFCGVFERMVEGLEEIDEGVERETLARRMGKGRERGVDGETREGSQSDDEAEGDGGDEEQLVTLREIYPRTLDEVRALLS

>CF317_000704-T1 CF317_000704

MSYKQSWLDFEKVSGGRMVLKGSPEEIKKQYEDLVAMLMPHMPPPSENVESKDGDVDGVKYRVYNPKHASGPLPIALWTHGGGYMTGDLNSDDVLCRAVCEHANSVVVNIDYSLTPEAKWPTQLNECVKVYRWAHDNASSIGGDAEKMYTIGGSAGGALALQICNAVLKDPSLKDSMKGVAALVPCTTHPDNVPDKYKPKYKSYTDNAEGVPIIDRGSMDIFYEHLGTDPKDPSCYTLLATDNHKNFPPVYFTSCEFDPLRDDAYVMEAALKEAGVETKHDHYEGLPHYFWIFPGVPEGQEFVGNMLQGIGWLQSKM

>CF317_000705-T1 CF317_000705

MPLRVRIRGPSGQSTASFDDNATVETLRKQITESTSLSAFDVKCGYPPKPFHLDQYERTKLLTELDIKLNGEQLLVTRADGPTTTKQASDVKNQRSHVESPNKVPQPLRSKQNASKAASAEPAAPLSLTRKQNLEMTDPPEIFIPDIGGTLVLRIMPDDNSCLFRAISSAVMSDLDAVTELRSIVAETIQADPDTFSKAVLDNKSPDSYCRWIRSQDSWGGQVELVILSQHFGVEICSIDVQSLRVDRYNEGASTRCFVVYSGIHYDTIALSLPGMLPEEDVKQFEAPIKDEVLPKAMDLCRKLQERHYFTDTSGFQLKCNDCGAKLVGERGATEHAASTGHYNFGEAA

>CF317_000706-T1 CF317_000706

MADDGKKADATGAPDAVESIEVDEPVTKEEDAGEGEGDGDGEGSDNDAPGEDDDVAADAGDGKVSNSLYKVYKAITEVLLNFKIKTKNNEEYFPAGLFKRLPNKRLLPEYYEVIKEPSAISTLRGKIQRKQYTGTSEFVHDFALVVHNAQVFNRPNSQPVRDVLKLEEVFKAELQKLVESGFATEEEIKWPDLGEIPYSTPEPDPVTEEEEDDDDEDDEVDDSDDDKRRKRGRKGNKAPGKKGEEDEEEDKAAEAEQKRRGRPPKVATPMEHRIDRILKSLKKPKAANGLPMIAPFERLPDKAELPDYYQVITNPMAFDILKKKAKRKKYASVDEFMKDVELMFHNAMHFNEDGSDIHQAAQELLEEARKAEAEERAKPDSEYLQAAEGRIPLPHIIYKGDMWKVGDWIHIQNPNDITKPIIAQIYRTWKTENDEEWINACWYYRPEQTVHQYEKHFFPNEVVKTGQYRDHKIEEVLNKCFVMFYTRYSRGRPRNLPADIEVYVCEARYNEDKMKFNKIKTWASCLPDEVRDKDYEMDLFETPRKVKKVPSPLLSRLKEDSKATDELPQPEWKHTNAPPLVGGIHKRPRDENQSPPPEPTPPPQPAPTPVARTPSVSQPVNMRVPMYPQHSSNVENRTQTGRPTPSMTPQTSFNTNTAQYSRQTSYTGAHQYTNSTHSMAPQSQPYQYTPQATPSAPLVAAHPGPYSASTPAYNRTLSNTAQPVAYNVQQATNGIPHRIAEAYVLSDAANAAIPSNIREQFPRDDQGRVLFFSTPPLDTRHIVSGSSEAEQGRPLEHSDQYQRAQALRKRKHDHRDGEDGMEIDDNINTMSKTKQAEFAEPTSGTQVTRAVSEKAMSKLAEQIKESTDDEYKAQYGDEWRQILLADLKWGEERRRREQQQDRAAEQKRTAFRNAHLGNLNGQYALNVQGYIMGWQKNFFTGTYLDDHDSRLP

>CF317_000707-T1 CF317_000707

MAPYGTNWLVDFFTAGQEKASVTANAAKTDVAQARKSTSSTASKAASTAKADMAQARKSTSSATSTATSTTGNKANEVGDGAANLANYPTSNTEQLAGPAADKTEQTTELAGDPTPSAANNTTDKVKRPGFKSHDSGRDKIKHVFGLDHRQHKAEAATAYDA

>CF317_000708-T1 CF317_000708

MSSSTSPQKHNHNHNHNHKRTKSSTATPITPLRKPNLTHNPDTGYFPHASTTTSNSSPQPIISPTKAAASARLHHELTALTTWLESLFPNQSPLPPHLLKWRDEALSTPPPPSTSGYYAEDGLGNLVQSNPVLEALKALKQANLSADHLRALLHQAEVSELAWLDGLRARSQQQRDDGVPARMVLDQVYVSLNVSGRQALDSMARSAAMLGLEFRPDADAVGCDGNEAEDLRNAFSRRILRQAHRKLVAQEQVRELELLQKVMVGRMTADVNRELLTTLSDGSMVDEEAEHIHARTTQLSRDTKQINLKIMEYEDRVKSLERQLAGLRTDSTNMQEVLEARKRVEESKASVAALQQRVAVFHGLPPDLDASREEIRRTMNELEALKRKRGGLFEMIGNG

>CF317_000709-T1 CF317_000709

MAEESSRFLVMPIEIRKLVYDLLFQPTSRIAVQAAPQREYEYSPVTETDSTTWPVDFESWAITPHPQSSQFLRVCSQVHVEATPFLYSSRVFDLTYRDSFKLLLHNIGPSRFAHIRHVSLDWDALEEVARALNKDNYQSGLAGLRSVETASWRIRHLGGTSMRWRNVKASERSICQATRDITEKHPLLRVVAEQKYQRRSCSEASSSTPSSCKVKWRILADEEDMTEDEAVVDIKCDLERLRATKDEASDNGFSLPMIDPF

>CF317_000710-T1 CF317_000710

MNRALSIRGKGRKDNKDANKHTFSMATFRGMKESELSKKLFKLIKTENHAIGAYENAGRETVSIASQLSDWGESTGDDSISDLSDKIGVLLSEIGEQEDLYAQNLEDHRSVLKQIRNTESSVQPSRDQKAKISDEIAKLKYKEPTSPRLVTLEQELVRAEAQNLVAEAQLTNITRQKIKEAYDLNFAATIERAEKQIILARYGRRLLNLLDDTPVVPGDVRQPFANAHEARQILNEAEVELQRWSPNHEPINSNAGGMGANLMPAEDMTTTASEAPMSDAGVEESPMTGPQPPMISQATTTGHHSSAVEDVNNPVQGEGSHLEKAVAA

>CF317_000711-T1 CF317_000711

MLASWVGPDGKGTRTVDRLMDDTMRLSLNVISAAGFGRKMDWPTLETKSRQDDSGYVDMSKIKNEDRDIDPGHTMSYTYAIHCLLDNILLQFLLPRWLLSRLPGVTPRKANEAYREWGNYMRELLINKKADLESGGASNDPNDILTLLVKQQLLNTDNKTKGQLTENEILGNMFVLILAGHETAANSIHFSAIYLALFPDSQRRLQKDLDQIFDGKPPNEWDYERDLPQLFGGMAGAVLAEELRLVPPVVGIPKSTMEGVGPQKLTVEDKECLVPANTYISLSTGAAHRHPGYWPTCKPRLAGGHTTHPIANIDNDLEEFRPERWILSESDNGTTVTADGTPVDTLGKEDGLDVNESPDTSDRLFKPIKGSYLPFSDGYRACLGRRFAQVEVLAVLAVLFQNYSVELSVDKYASDAELEKMNDDEKAEIWHKTAEDVRELLLNGLGVIITLQIRKGTVPFRFVPKGQERFPDNVDEIWKRNHPDQVSSKGVPGWSTWAGAPRTQRRSGAFDPLSAMKNHGTTKVPRGDVDTD

>CF317_000712-T1 CF317_000712

MDILVKSSTLCIHARKCQRAQVFLEAKSLCALQKVESSRNPLHPWSEPISYLGPDGIKHKEPANKTTTCNIFGNASSSPGNVFVSWPSSHQSATPTPRATTTTTGQLGSTLPWAEPPWYTGRPSPYYNDSHRHLRNTVRKWVEDNVRTEEWEAAGMVPQEVYTKCAEDGLLMPIAAGRKIPKEWYGYPIIGGIKPEEWDGFHDLVLWDELYRGGAISSIFIGLTVGAPPIRQYASTELRSEIEPQILSGQKRISLAITEPSAGSDVRNITATAEKTSDGKHYIVNGEKKWITNGMFSDYFCTAVRTGGPGAEDLSFLLIDRHLPGINCRKIEIGAGKLSATTYITFEDVAVPAKYLVGNEGGGFKFIMSNFNHERIWIVYQALRGARICLEDALAWAEKREVYGQPLITQPVVRHKLGICGKKVDALQAWTEQLVYEIDNLSEKDGNRLLGGTTALLKVEAGMVDKYVADECVKIMGGLGLTKTGQGARIEAISRGVIGLIVPGGSEDVMIDLGVREALKLSRARKTAEAGRSRL

>CF317_000713-T1 CF317_000713

MSFFLKAQPILRQAAVVRPAPRLFSTAVVYQKSATDSVKDGLKAVDRTVSDTVVAGIDKGVEMKDKAASTAGMKANEVQGEAQQKMGEAQGKYAEVKGEAKGKAQEIQNKAQS

>CF317_000714-T1 CF317_000714

MFVTTREPGVRRRNRVYTNSKPARSSKISILSAFTVATTGSGGSNSTITQESYNRSQQKPKRRRSKEHRSHRSYQDRNTSVDVFDFLVAEEAKSKETLAGTDDIAENESQASDGGTATTEPEPIAHMDESDPEGFYRNLSDSGISMGSSSSTSGFARRNQLPALPEEPLGRPSPQPYPGNELALTDPRWMWPTSSPRPYPEGYIPPPCPPPPPAVIYDVPTYPYPPYTPYGTPPPAPTEYARRPAIIIREPASRDVKPQCFRSFSKLSTRLMLQMQDDIVSLEEELKLLDEEDDAAEECSESGSPPSHREMQDRKAREEEITLSK

>CF317_000715-T1 CF317_000715

MSDGMYQSQHDRAVLTNLAADFEQVRRLQQERNAAGAGKKDSKTFDASNLRTDTSTKASLSEAFDKDLYGSNGDKFAGYHTSLAPDGEDEEMPDSDNSRRLIGQYTASKSQMSEFAHGNGVEEEDILLGREKSNRIADRETDYQKRRFDRGPLTPTRADPFAANQQPGAAADGSTYREVMQLQDIEREEERVKKLIAEKQANGDAVEHKPTLKDEADKEYTEAGSTVEATGRKRKKRWDVAGEPATDGNTDAQEKKKSRWDKAAAPEGAVQAPTKRSRWDQAPSLNGAAAPVAAPPQQVMPAFGTDISARNAPLSDEELDMMLPSEGYKVLEPPPGYEPIRNYAKRAQVPSAAASTGTIGGFMMQEPENPRSMGKQLPTEIPGVGDLQFFKAEDMTYFGKLVDGAEENTLSVEELKERKIMRLLLKVKNGTPPMRKTALRQLTDNARQFGAGPLFNQILPLLMEKSLEDQERHLLVKVIDRVLYKLDDLVRPYTHKILVVIEPLLIDQDYYARVEGREIISNLSKAAGLAHMISTMRPDIDHVDEYVRNTTARAFAVVASALGIPALLPFLRAVCRSKKSWQARHTGVKIVQQIPILMGCAILPHLKGLVDCIADNLNDEQAKVRTVTSLAIAALAEAANPYGIESFDDILNPLWTGARKQRGKGLAGFLKAVGYIIPLMDEEYANYYTSQIMEILLREFASPDEEMKKVVLKVVSQCASTDGVTAAYLKENVLQDFFKSFWVRRMALDKRNYRQVVETTVDLGQKVGVSEIVERIVNNLKDESEAYRKMTVETIEKVIASLGAADIGERLEERLVDGMLFAFQEQSVEDIVMLNGFGTVVNALGTRCKPYLPQVVSTILWRLNNKSATVRQQAADLISRIAMVMKQCGEDALMGKLGVVLYEYLGEEYPEVLGSILGALRSIVTVVGISQMQPPIRDLLPRLTPILRNRHEKVQENTIDLVGRIADRGPEEVNAREWMRICFELLDMLKAHKKGIRRAANNTFGFIAKAIGPQDVLATLLNNLRVQERQSRVCTAVAIGIVAETCAPFTVLPALMNEYRVPELNVQNGVLKSLSFLFEYIGDMAKDYVYAVTPLLEDALIDRDQVHRQTAASVVKHVALGVVGLGCEDAMVHLLNLLYPNLFETSPHVIDRIIEAIEAVRMAVGAGVVMNYVWAGLFHPARKVRTPYWRLFNDAYVYGADSIVPYYPDLKSEGVPRHELNIVL

>CF317_000716-T1 CF317_000716

MARNKAFRSERDPFANNEHVSKEIPHGPFSFLNPTIAHLELKLRDHAPEQWSSENEVTNEKTESTPNVRLLWRSRDNRKGRHPLLVRKEEVGKDTTPRPTSHWPEVFKTIGRMFIHYPVWDISWLIAYIFTWGSIVWVINGFFAFLPYVRPSADFGGESLYGGGITAFVGACLFFEIGSILLMFEAVNENRTGCFGWAVEKLLEEESGRERLQLRPTLEQCTHHHPNKRNFVGKSPAFNESATHAQEELKDNGKTWQWFPTRNALKTHYFHELGFLASFSQFLGATIFSIAGITALPGIINNMSQPLTDGIFWVPQIVGGSGFIVSGTLYMLETQKNWYTPAFDVLGWHIAFWNLIGGIGFTLCGALGPAAGNHGAQYQANLSTFWGSWTFLIGSLLQLYESLQKHPVEVEKGPQS

>CF317_000717-T1 CF317_000717

MADPPRVPRHGNNGRPMSGAEKVYEAMKQHEDYKPGNVFETSEVLAGGMQAQGNVVTGNPDAPYRSHFFDHTTVSGFNIQGDMPPEIALEFAKLNAPQPQQHQLRLHQQDQVAGATPPEPPRYRIIGGEKIPLPTNIEVSSLPHYAPKTRRHAKSLSRDRAVTLQDGAAKTFDGHGYMLKPDEFP

>CF317_000718-T1 CF317_000718

MSSIKISALLGAAILLAKANAHGIVSGILAEGKYYPGQSHYSTDPSPGWLADNGDNGFASSLTDENIICHNNAVAGTQYVTVAAGSSIELQWTAWPESHKGPMLDYLAPCGDDCTTVDKTKLQFTKIDEAGLVDATSSPQVWASDEMISNNNTWVTTIPDSIAPGKYVLRHETIALHAAGESNGAQAYPQCINLEVTGSGTNSLSSGTLGTKLYSLTDPGILVNIYYPVLTSYDIPGPEVMAGGSSNTQPSASGSVSNSTSVAASSTSASASSAAPTSVAVTATPTTGNAVASSTTSGAAVTATAGGRKFVCYEEL

>CF317_000719-T1 CF317_000719

MSRYGQDKSASLSPQPHNKRQRDEGDSYGRSSHREGRREGRRERDHGRSHRRDEERPRKRRSLSSDSDEQHDTRRNGRKYRDRSPRSARRNHNHHRRSPSHSRTRSRSPKALQRSRAPLPPQNRAFEQEHDPEAPVVEKQKPNFAPSGKLAAASNTVQTAGQTIVLKYHEPAEARKPPARDAWRMYVFKDDEIVDTIQLFEQSCWLFGREMAVCDIAIEHPSSSKQHAVVQFRYIEKRNEFGDKIGKVKPYVLDLESSNGTWVNEERIPEAKYVELRDKDVVKFGHSRREYVIQLPKG

>CF317_000720-T1 CF317_000720

MAHPDVESQRIASSPDELLSRARSNGSVHIPADIFEQMYLAPKTRVAGHLRQTFANPTAISIGGFLLCTTPLSMSLLGWQGSDRLGIATVGSYFWIGGLLLILGAIGEWIIGNTFPATVFATFGKIVHIFKISSGNCLQILGGFFLTFGATLVPSYNAYGAYGLSPSNVAADLASIRMFHSTFAFFLVAMTLLATIYCIASVRTNIAFFLIFLSLIPCFATLAASYFAYGHGHVASAALLQNVGAGLLLAVSLIGWYIFAALVLLSVDFPFMLPLGDLSTRIKGYTEKHKVTKGE

>CF317_000721-T1 CF317_000721

MAATNGESFKDFHIEALIIGGGMGGIYSLHRMRQEGYQAKIIEAGSYFGGVWYWNKYPGARVDSECPLYQLNIPEVWKGWNFSQRFPDHEELKKYFKYCDETLDLSKDTIFNTIIEGVERQDGHWKCNVRGGGVITCKYLLLCTGSSYKKHYPNFPGLNNFKGQLVHSAIYPDGGIDVKGKKVGVIGSGATGVQVVQELGKQDCQLTSFVRTPNAALPMGQRDMTVQEQNVLKQFYKMLLEGGRRTASGFPINTYPEKWHDLTHEQRLQRMEENWKLGGFSFLLSSPREFLTDKEVNAEFYNFWKEKVRARMSDPEKKEIMAPYKQEHYFGTKRPSLEQDYYEVLDRPNVKLVNLKKTPIEEFTETGIKAGGHHEFDLIILCTGYDSMTGSLMDLYIKDKDGKLLQEKWQKGVETYLGIMIHKMPNMYMVYSPQAPTALSNGPPIIESQIEWIIKAMNKMRDEGISEMDAQDEPAKKWRQAIQDMNDKTLYPLENSWYMGDNIPGKVREQLIYLGGVDLYNRQIHEALDSWAGFDLTKGQTVPVR

>CF317_000722-T1 CF317_000722

MASPGQSLSAQNSKQITTVGLTEVFSSPDPQVADIVFVHGLNGNPEKTWTADNGVFWPRDLLPKALGDIRCRILTYGYDARLNDAVERPIIFVAHSLGGLVTKQCLIYSRSIEHQNSVHLRSIYLSTYGILFMGTPHNGSDLAKWGSLLQSIAAATLPRKLFDSSSHLVEDLKKNNETLQNINRQFTEIMPRLHVFYFYEARPMDLKGTRQFIVDEDSAAPNFPGAERMGIERDHSTMCKFENAEQSAGFPEVTEAIRRYAVAAPESIAIRWLEERQHRYIRQELGAPESLLGERRSSRGTSPDLRLGGTPALPSNSNTPNLLESGLPARVTPWEIEEQEDPARNPSSARLSYRMPAHATNQSSPKSPNSASSASVPKLTRPRLVAPLGFRPNTHFIGFDIELARLVRKLADERRSQIGARSALLYGPPGSGKSHIARQYVWQHVEEYPNGVFWIDCKTPESLNKGFWNVAQTLGCYDELQSSRPDGFIDAVRLHLARQEKWLMVFDGIIFRTEEDLEAFKRYVPDGKGSSIIYTTLDRTLANRQRLLDPPGIKVYPLSVEQGCVFLYKSLHMRDDRQPSERQQKKARQLVKHYDGLPLGIHAAAHMLLARGRALEKYTPGPSDNRLAAPFISILDALESSHQLEAVHLLKIICFLNHEVPVAMLKFGQGALADLGAEICSTDRNGGSTRRELDNSIATLIRNGLIERRLQTHSTASSAGRSSPEATRAPPLSSVERQNSDPSSRTGHEDTLGHDNDSQKSSATFVGIDVIQVHTVVQQVYLDYLSTYPQQDFNGWLTIVAKFMIASWTYAHEKIKSNAGRGSMSDFREFESHAEKVWSHFPARPDGVPQALRQARHDLHMVRRMIKREIDNQSPSQSSDMSGRPVFASVFERSGSSSDEGPTTPTSGLSRITTWSMEPVVDPSESPVNYQGPVFTGLPVALSDSWQGDNGYLSDYEQRLSKDRTLSTSTEKGPDERQSALRAIFEGRGNFEAWKPAPVVGSISSGEIEPAHSRSNSLSSQTPRPGSSASLAEAALSAMHRISPTSSKISHPSVQQIERRPLSEMPSNSPTGRQTSDPLLTRHSSTSPHLRKAVLTNTMRPDQLPFDENINISRRSQPLTIAGRPWKPSLPTLESHSMPAEHGQIPMARDPSHESTLSTQTEPADNSSSIGDEYVTPQMSPVDRPTPSPTTRLGKIGTRIKSDGPPYPIINSPDTVPLPILSPREEEMKGLGISNE

>CF317_000723-T1 CF317_000723

MRVSLRPSLLQPQRFATIQKRFLTADPSPIVVQHHPAPHSGGIRVLLLDRPANRNALSRRLIADLRKHIQHIKDEGGVGGTRALIIASNNDNAFCAGADLKERRGMSQDETKQFLHDLRSTFKDLASLPIPTISAISSTALGGGLELGLCTTFRVFGSSAIVGLPETRLAIIPGAGATYRLPALIGVGRARDLILTGRRVSGPEAYFLGLCDRLVEILPEEQTAEGKARGKVLDVSVQLAKDICEGGPVALREAMRAVNGWQRGEVSENEAYENILNTEDRLEALKAFAEKRKPVFKGR

>CF317_000724-T1 CF317_000724

MDEAPVKEVIARLEDQTWRVLKTSGKDLLPFLSSDCMMAMPLGMQLSAVSVPSIEEVMTSKAFVPWTSYRLKDVVVTPVGSYGAVINYRVKAKKPEPKGEDSTFRALVASVWRKDAESGAWQMCFHQQTPYDLTPEDLI

>CF317_000725-T1 CF317_000725

MYDDLLYDACIINTRWVWLLKTEHAYVIDSKQEPGNRSPHQQKGAYILPTLSSPATSLFFCDKTVPLATVPHSIFSLLSKHPSSATMKFLVLLTTLATLQAVNAWPMRWVQRSAAIDNVVPVAPTSFLNPREMLVRRRVIEMNRNRLSPEEIDEMCAEDPDPWLCIKLWSNQG

>CF317_000726-T1 CF317_000726

MPNKGVGKVARAGASSLCTVADLLRILFESSDVAKKCITIEAELQKELDKLKSKSKRHVLKKAFAALYREQKIREIQARLVQNSKTLELGLSSQTNLQVKAQMVMQEQRFTNLGGKLQQFISNVAQGHTRLEDLIRSSAADVKVDIRNEGTENRAVVLNEGSKTRAAIFDLANDIQNQGTTANHYDLLSCFHYKEMKHREADIDESFSGTYQWIFRDTTKHTWATFSAWLAGPEKVYWISGKPGSGKSTPMKYILSSKELLTKLSEARPNVQITIICHFIWLAGSEAQHTRRGLLAALIYELAHKDKDVRTFVESTVKLHPSRRDLRDWPVDDLEDLLIDGIKQHQGLVYISIDGYDESIHSEHKPKLLQLLKRLTEMENIKLCVSSRPEKWLVDHFHGCPMLKVQDLNEGDIKTMVTETLEKQHSLSRAVLGNDEKMQRLSSAILWKAEAVFLWDDTEILHARLDLLPGDLSDLYAHMWKRTNDDRELYREQATEIFSFHEFLPCDLTLMAFAYDTSLQRLPYERPGAIADARVLQSCFELSKLINVRTAGLLEVRTRSLDQLHSLAEFLEQHEAGSYTDGQRTVSLDTASAKTKSVDVSLSLVSHPNSVTIFGTDLKNPSPNMSGTASHVHTLGGRPVVRKSIDRVDFVHRSAYDFLKTHELGYEVLGSPRTTRAERLANMLIAIFMRYVAMSAYASKTHRSGALEEIKELTYRLDKLMGESLSARCDLKETETEHAWMRLESFLCQWPALREGSSETEVNGREENSRYSAPLAVLAIVLAPKNYTSPSYHGRFWKSFCGRSSDQFIAYTIAAMFARRKPYEAVHSFLSALTSRYRPSPKIWEEVWMSDLLSGVCYPLEAYSSMAHALLYRRLYDDRFGNKLPESYAAVAESLVALGIVNTGCLQQQILLWLVVEKESLNLFDQCKFSVVNLFDDTYYLVVLETSLYDVLSFLPEQDRKLLGVDQNNDLLVEQHRRIKEIVQVKTEWTRRGSVKRTATDIDMSVEKQEDYKESCLALFRTLSTHPTLSASTLTRYKLDLTSNEASFHLAEKVGLARKAPPVREDDRYNWFTPGMYEERKNYVDDFLANSEFSTKQRSIMPANTEPVPRSQVSFKSNPSEVPAPKDIIHHVESPPTSSGTTISMLLSYLRAFGRATITMASSFVSGATRTQLCSIIAVVVLLVSIMARFVLG

>CF317_000727-T1 CF317_000727

MTLTILPPLPDTDDYVPSRSKHYNASSPSASDSEGGMSLDSDPSDIDSRPTKRMKTGSKPSISRREIVVPGEPITDETQWMRGHGTTTTLSPAATLITATLAGPITPTNKLLSVLPVRARYTPEVGDLVLGRIVSVDKSQWRVDVAAPLLAKLSMSSINLPGGALRRRTANDELQMRRYFQEGDLLVAEVQSVGSTDGAATLHARSLRYGKLRNGTFLAVSGAGGGGGVVRSRRQQFTVNTNTGATGGGEVDVILGVNGYVWLSKHIEDDTAAVGSKGNTPGASKAGGVDLGISNLDEAVSSEAYSSQNDMIEEGSRREIARLSEVIRALAEGAVRVDEDGVRRGYEVAVELGLSTEGATVESSGRELLDHEFRRRVVEGVL

>CF317_000728-T1 CF317_000728

MAASKTALKAIKTSIDGGDFAKASLLASDLLKDDPKNYNALMSLGFAEEKLKHLGEAENALRRALDLKPQDVQPYKGLIRLYEQQGSDKVDSYHDLATNLATIYAQQEDREQCQNVVNQYELFVKKNASRSQYRRALELMLPASPLYSTLEGRVPHPSHTYQRILESSQAEEKQWIDSQIGERRTRLGARLDRVTQEVKNEAVEKFQVELHYQHLIEWARDDDVRHVLDQELLQRMLDVLLAMPQEQKPSQRDKVLEKANGMVIIKQEFALAWDIALEWVDTEDLAEWDPAILHQYIDFFPDNGLAKVMRGFLYGNTSPFPLPKSSDPEKPVEKLSETDQLIVMMEGLEDCKESMLAHRIMAVTYLALNEHERAVEMARTSAKLYQKAQKDYAMPLQDSLDAVNLILGRSLVVFQSPRHHPEARTLFENILARKPQLTDALLGIGLVYEEDEDYSEAVKFLGKAMERDPVNTRIRLEYAWCRALDKDLQGGLDLLDGILSHVEEEKNPDLNMKAELLYRIAYCKWHIDSSPTARKDKSGSYRYLIDALKANSSYAPAYTLLGFYFLDYTRNKARSRVAFQKAFELSTSELSAAEQLAQAFAMEAEWDLVELVAQRVVESGKARPAPGSKKKAYSWPYAALGVVQMSRSQYSLSIVSFQQALRISPNNYHCWVGLGESYHNSGRHVAASRAFLKAESLDHGLPADEAWFAQYMLANVQRELGNYEEAVKAYERVLTNKPEELGVLLSLLQSLVEFGWARIHQGHFGHAAELGTKAVQTAAIIAKDKTDIFNLWKATGDACSVLNAAKAYVESSTIEAVKDLLSMNTNPDVFDVLADTDQVSIKDLEQDAEDHTKMAHLLVRAAVLAHKRGIDASSNDMHAQAVAWYNLGWAEHDAYVSVASRGGKRPRQLLKGAIKCFKLAIELEAGNSDFWNALGVATLTLNPKVSQHSFIRSLHLNDHSARSWTNLGILYIHNSEHELANEAFTRAQSTDPEYAAAWVGQGLLATLYGKQKEAAGLFKHAFEIAESSSLAAKRYYAASAFDHLLVDLNAASDVNMLIQPLFALRQLRTQSPTDIITKHFLSLFAERVQEYNTAQSNLSAVLEDAEAQYEKSESNESLVRFAQAKADHARQLLALGEYSEAIDSAQFALDVTEDDNLGPAYATRRSRWRLSAQVTAGLAYSFTKQMSESIKCLQSAAETSKALNNDKVDPNVTALLAQVLWASGSTKEKEAARTQLFECIEENPDHVGAATLLAVVAVLDEDTETLEVAADDLKSLRSKSRTSVVDKLRISKVLAAVLLLKSSSGTSEEEVRSDALGGVMLNPEQPQGWLQLAQVGGATEGDGAGYASGMAVKNALRQIPPGGRLTSEDLARTYEVTGQGEDMKKAAY

>CF317_000729-T1 CF317_000729

MAPSPLRIDGRRLRDDRNREVTFRGINVSGDAKFPRKPDLPSRVRDHFFDGDNVSFVGRPFNEDEAHTHFARLKRYGYNSIRYVFTWEAIEHAGPGQYDDEWLQHTINTLRLAKSYGFHVFMDPHQDVWGRHSGGSGAPMWTLYAAGLDPQQFTKTHAAMVHADWPDPADFPKMLWPTNYQRLACFTMFTLFWAGRDYAPKAIINGVNIQDYLQGHFINACKHLALKIHQAGDLEDVCVIGWESMNEPNRGLVGWENMDAIPKELNMKKGTCPTPWQSLLTGSGRAVEVDTYEFGNFGPYKSGTQLIDPEGASAWLLPEASRDDHYGFKRDANWRLGVCLWAQHGIWDPETDSLLKPEYFDKSPSTGEKMTYEKYTNLYFMQHFRNFRDAIRDAHKDCMMLVQGPVLEIPPTIKGTADDEDRLIFATHYYDGLTLLTKHWNKLYNVDVFGVLRGKYWSPAFAVKIGQHAIRNCLRDQLIAIREEGEKYTGVRPTLFTEIGIPFDMDDKHAYKTGDYSSQIAALDANYFALESSGAAGSTLWVYVASNNHLWSDNWNGEDLSICSVDDLLPPGPPQDEAPLTNAADTDLTRQDSAKSGSQVHPDNLDAKLPKPSIRRVPTKTPPELGNHPGLRAAEAFLRPSPVATHGDVNSYGFDLKSATFTFSLTAPSKTPDDAPTEIFLPHFHFPAGQTTVEVSGGKYVINLRDVDGEGMQWMRWWHAEGEQTMKVMGVVRKNNGQDTGSSADDEYGYLEVMRKVGENCSIM

>CF317_000730-T1 CF317_000730

MESEERPTKLRKLSHDNDGNFAPAPVEASSHAQSQSASTQDAASELADKVEADAGPEPEKSANSTAGGLKSALGAQMDRTSDAEGQPAEPVLSKNQLKKLKRKAEWEAGREDRKLKRKEKSKEKKARKRAQRDEGAEQEEGTGNVELHGDDKHKPKPRMPPRKRRQRLPVTFVLDCGFDDLMVEKEHISLGSQITRAYSDNSKAPFQAHLYVSGWTEDSELRKRFEGLLKAMYRNWKGMIFTEKDFVTASEMANQEMTGKYGGKMLGAFEKYATVKGDGTAVLAEESTSTNGDQSVEKTNAAEAKPETEGVNNTGGAANRSQTNGAQDSGESPFSDLQEAGEVIYLTSDSPYTLTELKPYHTYIIGGLVDKNRHKGICYKTALDKNAALASSVNSSATSNPAASTDNLANSRSTVKQIKTAKLPIGEYMTMTARHVLATNHVVEIMLKWLETGDWGEAFLSVMPKRKGGMLKNAKAKDEDEGQGSTTGVVGVRDSEVNSDDEHEHDVEVQSSDSDPLEADVELAT

>CF317_000731-T1 CF317_000731

MKGKVFTARLDGAIRTHAHRSKVKQGGLSRKVALQAASTPAPTRPFQPSPHELETLQLAHKLLQSDIAVGNRQYPHAFRNYGRLALRHRGPYYEFPLLSHSKFYDGTQNRNPGSKRIVLTGKGEFANIVLHDDGVKHGFRWLA

>CF317_000732-T1 CF317_000732

MIVTNVTRLRTSQALLSQLVQYIPSFLVAYLQSIAAVSNVTIEQYLPGEAPYQDLNEVGTYNNYTILYRTHHCQSISASKYPDIHITSIEKVEFDFALMYLTLGFYHLNTLNEPYKLIPECRSCLSTFASDGCGTGKTYTTLFSIVADAELLENQMRDSSTSDEEKAALDCRPHIILCPNGCLTTWVTELKEKFDGLLTVSIAYAHAEGQSMDPWMKSRTLPTNCAEAVLALRRLYPFNDIKGARHVILCSYSTYVKRFITKMPDYDESKQNDCSPTSGRPANTDAVLSDVNFNDPWDLKQYYPPFFRKAPKQEEIQLIIEQESAKKALKKGDVFTCALRYMALCMHLDEGHAIKNPATQRHRAPYCTYAPKVRAYLRDHLQ

>CF317_000733-T1 CF317_000733

MTNLSSKLAELEATGRPVQVGLIGAGKFGSMFISQSHRVRGMRLAGIADLSAERALASLKRTGYPAERYDETLSMSLEEGIKAGKTVVTTDSEKLISQPGIDVILEVTGNPAAGVRHALLCCEHKKHIVMVNVEADVLAGPLLTRKAKEAGIIYSMAYGDQPALIAEMVDWARTAGFNVVCAGKGTKHLPEYHYSTPDTVWSHYGFTDEQLASGDFNKQMFNSFLDGTKSALEMSAVANGCDLNPPSKGLKFPPCGTHDLPQVLKPISEGGQMEKYGTVEVVSCMETDGRWVVGDLRWGVYVVIEAPGPYQKECFAQYGLKTDKSGRFAAQFKPYHLIGLELGISIATIMCRGEPTGQCKTFKADTVATGKRDLKAGEKLDGEGGFMVYGRLMTAEDSLAIEGLPIGLAHGLVLKRDVKKDQRVSWQDVEWSLKSQVVAVRREMEDLYRKEFSERPQKANGVNGMSNGH

>CF317_000734-T1 CF317_000734

MGAKLSRTGPAPPAFNQQEWVERISAWSPEERAEKEKRFVRRIDKHLLPILIFVYILNYIDRNALPAARVQGLDTDLGLTGDEYNIAISVLFVGYVSMQVPSNMILGIVRPSWYLGGCMAVWGIVSGCTGAVQGFGGLVACRFFLGITEAPFFVGVAFLFSGWYTRKELGTRLGIFFCGAMLSGAFGGLFAAGIAAAFKNNRIESWRWLFIIEGAATVVFAVATAYIIPNWPATTKWLSEEEKALGIIRLIEDAGEEEDEIKTMAALKMAATDHRVWLCILGQICVQAVASLTNFLPTLVRNFGFSTIHTLLLTAPPYVLTAAVCVFNTWNSDRTSNRSMHIIVPTLAAMTGIIITMATTNIAARYFALFLMLPGTYSCFQISNAWMSNIAARPRKKRAIALAMNNAFGNTAITWTPYLYPASQGPRYTIAWSVNLGLSVVLLVATVALSLLLRRDNKRLGNNEVTREVDPATLEKMGSTTVLENVEPEKSAVLCPRTVGEERYSGTKSTAIDYRVRLSI

>CF317_000735-T1 CF317_000735

MRNFNLEIDQWRIKWHARQRNNNFIGTFPPKGIILYGYFAKLQLNSFAIRGINTRSGELATERKEFVNIAVSSAMSALTFVLEEEDMRRALVGTPLYVHTMIAFASVFLMKVATIKSQNTALGPHFSFDPPSVWSVLERMIDLLKNTITSTRHLLYHVAAGIEKMLRRARQGTNEWSSGLFKGDGVAQQPGADARQSPQAMTQCHQSSSEYHDWQPPNDQWPNTNNVSLEDAGQQFMANNELPHNQMMYMHDSVFLDAFGNEAANDVYSLLSSQFSH

>CF317_000736-T1 CF317_000736

MSVTKGSSKQIPASEPALIGLCSKAVNLTNNINTHLTDYNETHETQHDPLRSFTAKFGRLREIIWQIQAGTSRLTHGDIFVPYEASQVLSTKLQLTVRDLISCDLLVVKLISNSRKTGFGKFMRSFKQTSLDAEISMLGDSLGQHREALRTGWVVFSEALSKADQSNTPEPTTDPISKDVACEVKTLPKSFPAQSAVRIDQKRTNIAPILQATNSPRLDLMTSICPTTPKSSQEPATRIPVRRAASAKSSTETDEPRTPRVAEQEWNKPIIWSSGRSGSIDSFSRTSSFQFSPVKSPSKAFPTTLADPEARSNSGPTPEKAALIAALQRRDHKTLQQLLRGVSYGDSIPSSLLSQAVGANDISSVRLLLTYGVPVNRIDDDGNSALLVAVALCSIDMTSLLLENGADPDFGSGDADMTPFTLAAQQNQVELIQVMLEHGANVNKPSSNGITPINACIWHGVRSEIVELLLAAGANPNQKTRDGKTPLIEALTIRRVDLVRLLLDHGANPNLAGPKHPLWPATYLPEALKLLIERGAQPKLASGNMELAASINSIESVQILLDAGVSPDLKKDGVYTPLCSAIRDDRDDIFALLLERGANPNLPASEYPAWKCISHDRLHYLPPLLVAGADLRSPPGIAELAVAFNNKDALMYLLLNGVDVNAANEEGRTALTTAIRDNRGPLLDVLLAHGAKVTARGEDWPLCMALKNPQLLQRLLEHVEGTKGVSKGIIELAVQANQLESVKLLVNAGISVEDRTGGVFSPLTSAIRENRKDIVRYLVDEAGADVNSPGEHLPLIKAIRRRTIPNDNEVIEYLLSRGADINLIYRGWNAIMQAIEKGDKKLVHLLVEKGNGIDLQVSDPDSGQTVYEMIQDRGWSEGIEMLMKNQHQLRMVTDGSITKD

>CF317_000737-T1 CF317_000737

MMGNNNKAEDPVIHDGTIGGVAPDVAELKVEHHHKEKLSSTDEQDSEEGTEQLQDSQSYYSNFRPIERLGLADWRLTEKQIVRALDMALLPTLWVTYLNNYLDRTNIAQAMLGGLDEDLNLTGDAYSTALSILTAGYMIGQIPSNMLLTRVRPSIYLPRVVIVWSIVSACTALAKTPEQLFVIRSFLGVTEAPLFPGAVYSGLVLAQAVSGLLAAGIFAGLEGVSGIRGWQWLFIIEAAMSTLCGIVGLFTLPDYPHTKTGSQRWSMNHDQRLLAEARMEADRVTGSVARGGVLQGLKLALMNIKLYLFIILNTSRTCAYGFNFFFPILVEGLDLSANNIVALLLTAPTYFTAATVSFVVAWFSDRKKEGG

>CF317_000738-T1 CF317_000738

MPVMTDDVVPLYNEPLLMPGVKAYKQARRAQTRFENGHVNTCSKRENWPVFPSSRKMATNIALQTDLVPDFIVQNPSRRFIPGAFSVISLSRVGSQPQTAMGTNYTLHRARPINSGRSWHPVADTSKPLLASSFDALPPYRPPNSTQ

>CF317_000739-T1 CF317_000739

MNLLPSPFPFFNVEQQMLLLSILHCPSPSSSAAPYQPTQSRSSLFMGISTQRASLAGENSGADGYQNDRTVYSGGGSIDNNGKDFETDQIIVDTTNDLKKGVLSALDNNEKRQQRAIDKDHANIPGRVVPSLPDFIPFWCRQYYYDLHGRRHVHDIAAVTTSYAGVS

>CF317_000740-T1 CF317_000740

MATSLSPTSQSHSSGWFQQPLQCPEQITQRFAFDGKRLLPKHIPTFGLLHYRQFFNNLAVSLTLLVLYSINTATTGYQDQHRGHKSGETDKETEHKT

>CF317_000741-T1 CF317_000741

MGQSLSKLLPAPSATATNAGVAVATPGLPAGPPTFKFPSEPCLDKNPHPVLEFVKPIGARMNSTGNIRHKIQHEWPAFKDFQRKTKTDVCRELCRQLEAHYGHTWHKGPDTSQGKPRNFGVATATPGLTNVGKKRSAATAELSAPTASKRPRTLLEQAAKTPRPRSVTVQADSSMPPVATASGSSPQLTSQLTSHGRQLPMSSSCQPSSDAVKPIFDIQMVRRDQTASMLMKKLLSTYRNDPEKIWIIDFEFMSTSGTSPVPFQVSIVTMNDNNIVTSPINYNMSVGQLFDTILPHTFKPNKRYLARRGNERLQNTIQKTYGPHRSTASSTISQIRSQIVGMLTDDVVLVHWGGPAVELLTRVMVGGDHPISPKSRSRYEGIDACVLARAGMDRDVTGENNSLKEIHEAIFERQNIRFHTALGDVRATREIVEYFISQA

>CF317_000742-T1 CF317_000742

MDRLHKDNVAVDLTVPGATTINSTEDSENTVDEKYQGWLTAIGWQVYLASVAFLVGTVIQGSIALNYPDYGWEQYHGTLLTITVILCAAIFNTALASRLPLLEGSVLVLHVVGFFAVIIPLWVMGPHARPGDVLLVFTNNGGWPTKGLSAMVGLLAPQAVLTGYDCSVHMSEEIRDASITLPRAIMGSMAINASLALLMSVILIFTLGDVESILSTSTGYPFIQVFYNTTQSYAGTNVLLAIVIILLTACCISEVATASRQIWSFARDRGLPGSTWLSHVSPNWNIPLPAVTVSIGVTSLISLINLGSSVALNAITSLGALAVLVSYFLTISCVVHRRIRGPALPTRKWSLGRFGLLINVAALCFLTPLIFFLTWPLTTPVVASTMNWSSVMLCGTLLIAMTLYMFKGRHEYTGPVVHVKRDE

>CF317_000743-T1 CF317_000743

MAQAEFRFITVDRAGQDGSIYIITLNKPPENRLNVETCQEIIRALRAVESAVGQDAEGAVILTSFSPKFFTTGLDLNEREVNKFSSSDGFYPLLHTILDFPLPTICCITGHVFGGASLVTLACDYRVMNSQRGYFQMPPVNAGLHHDGMGSLLRLKLAPRVARRVLLEAHKYTGEEALADGIVDWIGPHQTLMDKAIEVAEQWKGKAKMGVYGLLRNELWGAATDYYARNSYVHRQETAKEPKVKL

>CF317_000744-T1 CF317_000744

MSHATQRHVPKAAVETPLGLFLWGGREKFDRRKEVLEVLSNNPIFSKGDVVYPSLARKEVWERTALQSRELIRIYLTRKWPYRTFMDAIRMTDWMLPVQPQFRIFMSNLERQMTDEQKAIWIPKAERFEIFGSYCQTELGHGSNVKGIETTATFDLDKDEFVINSPTVSSTKYWIGATGVWATHGIVVARLIIKEKDYGNHLFLTQLRDLGTQRLMPGVEIHELGPKVFQAMLGTDNGALQFHHVRVPRSQMLMRNAKVLSDGTYVKPKNEKHSYGSMVTVRALMAEITAHDLVRAAVTAYHYTTFRKQFKKARGDSSTEETTVFDYASVRFRLLPLLAQATTLAIVGQNIKRAYDDYTASMLSTGDTSQLEDLHLQTVGAKVYATEITGKGVETCRIACGGHGYNALSGFGRMYGHAINAVTYEGDNYVIGQQVPRAILKHYKAGTYSTIPSLSYLSILSLSAPPPTPNLCRPSSWLSPSCQKLALELRLANMVRQHIADTEAGIDTSYTSHALTMAHSDYVYWRSLQSVLSQISSQPYFTAMKSLAAVFGLSILFDPHHPSLAQALPLTQPQLSSLRTAYNDAIIDMAENQTASIVEAWGLTEYELDSALARSDQTPYEALLDGARKSEMSGANMSHMWPMMVDTRMMASRLSEEETATKAKL

>CF317_000745-T1 CF317_000745

MEKVPDGSVGPKSAGVDMRLGQLEGEGGSQREGNLQEGEILVRNSIMFSKYLFDEAATRKALDKNGYFKTGDIARREGEMYWILGRASVDIIKSGGYKISALDIEREILGLDYVSEVMVVGVEDEEFGQRVAAAVVLRDNGPQTLSIDKLRQDLRTSLAGYKMPTLLRIVKELRKNATGKVMKKVLASEYFPKEKDGCVEVWEGRKSRL

>CF317_000746-T1 CF317_000746

MSLPPDDRCSFAPKHNGPNVFPNFYLFSRLVRLAHKPSLSAINDVTFGYKATYQQFLTDILHFRNVLRQTLHPDVIAKLDCDEEVFINLLGPGGYEFAVAFFSLMALGAVIVPISPDIPVKEATYFATKCRSIGVVTAAKCTKLAKDLQSDMVKSSDHGFHCIQVTEHLMQPPLRPDELVISSDAYLDINKPGLIIFTSGTTGPPKGAVKRRGFFYDVATIFADAYDMREGDLVLHVLPVHHATGVTLTLLPFLFAGACIEFRSGGFDAAWTWERIKRADLDFFSGVPTIYMRLMQMYENKLVDLPRLREGPTWKGPHG

>CF317_000747-T1 CF317_000747

MANFQRSRAAVETEGVFIGGWSIKQAFVPTKVDGHIESSQDRAEHVSAVQDWSNAEEKALVRKLDLRVLFPCCIVYFFAYLDRANMGFAAVMQAGTSDNIEENLHLEGIDFNWAVSITYFMVTLLLLPSNLLMKRFSGKRFFPIVMCLFGTVVACISTVKGPPGFLAARFFLGIPESGVVPASIMYFSFWYKPSERAWRIGVFHAANSLASGVGGFLAIGVSKINGHLGLEGWRWLFIIEGCMPIAMSIPVYFLLLTFPEDSKALNERERYIAINRFGRGATRQTDVTFSWPAFRQVMSRPSTYIFFFSYICLLIVAVSLGTFLPVILKNFAGFSNNRSNAYSSTIYFVAIVLYLVWSWHSDWTRERMWHYLLPVMAAIPCFAIYTHVASKQSFDGIKPISLYGLAFLGNLVSIAQPAALAYRSSTLYGASEQAIGGATAIASLSIASIIGPQIFPIPDKPWYLSGFSAACATLACTLIGYASLPLWLLWEARRRKRKYGHAMPLRALEDVQHAQISAAARAKEQEQAMREEKHGEDIESVTHLEQVEMK

>CF317_000748-T1 CF317_000748

MDWSKDLEPAASADGAKILAEERQKSQIDVDKLSHHLFGHAYLERQDRVLKIVQKEKIFSKTNQANLSRPDRYALGLARGKRMRQLMDVYNWTEDDLLMAEYLVDDVQPYHLHMSLFAGAMKEQCSQEQRRYWQPKIESWEIIGAYAQTELGHGSNVRGIELEARWDASSREFVLHSPTLTASKWWNGTLGRTATHAVVVAQLILLERNVQGVMEEVSVGPRPFIVQVRDKSTHQPLDGIIVGDIGPKYGYAPMDNAYMLFRKHRIPHSALLCRYASLDPETGVYTRPKSSSSVYGQLTRGRSIIVMNARLVIARAVTVAVRYLAIRRQFRDQDSTDLTSRETQVLDYSTVQVRILPLLATAFALHHSGFAMRELYERTRSSSTLDSDNAQLAELHSTSAGLKSLATELAANSVETCRRAMGGHGFGGGTGLIQLNNDYLSKPTVEGDNWMITQQVARFLIKKVKDRAENPSARVLSRTEQNLQVFWNNRHEQTSFVVLENDEDILKAFNWRASWLAFTAYEAREVQRRPWNSLLLTLHKLSKAYSQAMLVANFFDTLQAGTSLDQATMAVLRNLFHLFSFYTMDAEAREFQTSGAVGTDILDKLPGKILSLMEDIRPHAVRLVDSFALPDYLLDRWAKQYYHERLLTFAVQWVDMMDVCMRISSVVLTPSTLSTKSLSIPTTAAMRL

>CF317_000749-T1 CF317_000749

MSSQNPGLEQSKTNTGRKRQRFESYAHHDPEAETIGSTAAAPPLQNDTDQNLTAPPLFVAYDTTFASSPSTTADAAISSVDSFYNSSYLSRSAILGNDFPDIDHSHVEQSVKRRELSSTEIKVLELYSAFDLPELPLQQSLIEAFQEKCWTWMPVVDLPADAQGFISNGLSLLPLQAVMLVGALMRPGVCTKATCDTYYYRVRSLVHSGYERNPLSLLAALCLVQYYTPTAPKDVSVDTPRFWWSAALGIAQQVGLHRQPAQRDRDHSLRRRLWWTLYARDSLMSSAHGRPRLLRLTNSTMKPPSIHDFPDPNSVRAHIFVSYVGITEIMCDLCELLVSSNHLAAETRGRIEQRLVEFVQKLPASLRLHDSKGVQRPYDFELAQLHIPLLTTIIILYRPCSVFSLSSPNAIGVVAAFLNFRIFQAIELREQTRSLSSTFSWYLLVTAIPHLSSLRIPALCKEASSVLDAIEGVLRTLGTVRPSATTNLQNVKAIRKALNTTGTSSSRSSRPSDMDTQKHDPPPPHLVRELLGIYGPRTLSNFDAIASAIDQASHDPGPSPQRSNVADLDGMTEEHMRTRTHVMQYQNDAPDLAADDAVDDGLDSLFGTHFQESMWMRDWIDELQHFPE

>CF317_000750-T1 CF317_000750

MSSSEPTSDSTVADPQPSLSIDNNGTISNKLETTKVAFATPGFKNDNDSDAITSPEFPEKEHALTSSIKKAFHLEHIHATGPSSFFHPTNVRMHVRWLHSEEGQQQEHTLLWRARDNRKGRNSIAVTHKSHVPLRSLLPTKAVEVGKNILTMCMTFPYYNMAFWSGWSYAFGSVLFVIDGAFSWGPTAFPRTKFAGEATYGVPLCFFFGAILYQIGATMAYLEAINDGSFQGSALKRTLEGHVEEQKAMLDEKLHTFFGHMAPHHRSSNDTDVPVVDPEAGWKEKDRHERPGSMYSTGDRRGGMDMGPAEEGEVSEYLT

>CF317_000751-T1 CF317_000751

MAGWVPPIGPPPLLTPKQVNQLIDQGHLNVSLPLHIQNSLAKLSAEGSAFFDRPEQDKSSAFPASHGTQCGYYSIEGQKEYLSLRHSPPNTNSDLHSYARDLWAVVAGYLHRILGDISTALDIPQEAWAPLLEDSLAMQKNESVDPSLPTLLRLFRYAPNEGVADEHIDNGFLTLCVGDSKGLQVLSRPANCVQPDQTPEPPQRQTEAGWAEAEWADAAGPTVLVGGMLHVLSLGRCRAGKHRVVANPVGRSSTVFAFRPCLRYDIDLEQFGGYGSVHAKELYGEVKGNRESVNARKYCNQEQIASS

>CF317_000752-T1 CF317_000752

MKFSNTTILILWGFVSNTLARTGHNGSAPVYKDPSASIDVRVADLLSRMTIEEKTAQLMQGDLTNWLNITNDAFNASGLADMSANKSGSFYVGHPIPQDWLANGIHQGQQYLVENTSLGIPAFVQSEGIHGFLIGNATIFTSPIGQAASFNPDLVKQSGRIVAQEAKALGVNQIFAPVQDLARELRHGRVEETYGEDAYLAGEMGHAYVKGLQENNVSAMVKHFAGFMVTEQGLNTGPAHYGERQLRTTFLPSYHRAIIDGGALAIMAAYHSIDNVPTVADHHLLTEILRDEWDYTGFVMTDAGGSDRVCDAFKMCQSEPIDSEAVTQYILPAGCDVEMGGGSFNYRSIPELVFSGKLDISVVDTAVSRLIRAKFALGLFEDPYASHAINDTAHVIHTPASIEMERQLDAESIILLENRNATLPLDKNANIAVIGPMADYTNFGDYVVYRSQYDPQNVNPLQGIKTASNGTVTFAQGCRRDSNDQSGFAEAISAARAADVSVVIVGTWSRDQNELWAGLNATTGEHVDVAHLNLVGAIGPLVQAIIDTGKPTVVVFSSGKPVSEPWIYEHASAVVQQFYPGEQGGNALADVLYGNVNPSGRLPVGIPQSVGTLPTYYDYVNSGRASLEPGYVADNGTIVFGHAYVIGDPDPTYPFGYGLSYSTFNYSNVTLSRSKAGVNDTLTATVTLTNNSTMDGMEVVQLYVSDLIASVVVPNKELKGFKKVLVPAGNSVDVSIDLDVSKLGLWNIRMKYVVEPGEFKIMMGPNSADETLTGFAMLSVK

>CF317_000753-T1 CF317_000753

MLVFVIALLASCVLSAPTVIDVRNNVTYKGLTRNGLDVFLGIRYGEDTSGENRFRPPRPYTPASGSTVNANSYGTACPQVPDSLPPPLTLTKTTNTSEDCLNLNIVRPNGTLPPSKLAVLVFIHGGGFWTGSNREITTAPDGLIIQSVDAGLPIIHVTMNYRLGAFGFAQSTALKNERSENAGLRDQRLAIEWVRDNIAQFGGDPQKITIHGQSSGGLAIGMQTLAYGGDRPVPFQQGICESQALEPGITGNFTIVQMQLLADATGCNTTDLNSNETIACLRQLDTNTVAQASFDTYASDVAHNIGDVWLPVVDGDFLPAPPSQLLAEGRFANVTTMILWVENDLQYFTPMDITTEQDTYDFVASYLPGFTNQSIQDMLALYPSSDFHGNPAANLSQEFYRSARIFRDVLMTCQPIGYGEHLARMGNLVYLVDQNQTMLSPILAHFVKDGKRFDIRGVVYQPNRSTESVHRDEDYDSLRDECLDDLQKDILVFKELGINAIKVYSILPHLSHDAVLEALAAAGIYVLAGLFSRCHCISRKRPYESYNTRLVNEYMQAVDCFSKHDNVLGVVAADGLMFDAESTQAAEVIRAVIRDTKLYMERQHQSKGQRILPIGIGDAMYASESTDSIDYFTAGDVSEQIDFYSFAHYDKVVEPSSHWSQVIQRFGGRNIPIFVSEYGNNITRPRQFRETTSLYYQDALQVLSGGFAYDFAHGGNEYGLVTAGHQKLGDFEFLKQRFQEAATTEETSTSELKETATAQPVHFPPLSESWRASSNIPAPVLQTYEQPWS

>CF317_000754-T1 CF317_000754

MATEVAIFQLKAGKSPEDANSATGQVLKDTLNTLTEQKGFQRAYWGTESENPGTFRLFVDWESVDDHLNFTKDEQYKPFLDRFGQIAEIDSAQLFHCHFTPHPPSEALSDHVSPTTEIRVYYFASDFNDRDGAADMVKQQVANMEKEAKAHTASAGGWSEEEVPIPGTSDKGKAYVALIGWQSMEAHMEWRATPAHEENLKIMEKYKDQIKHHSVTHFSGTLVQKGAGGVGDVTGDAQEEILNPQDAGKNAPKTKADGTTTKNNDDLSGAANSNKKSRVGG

>CF317_000755-T1 CF317_000755

MATEVLLDTTMGPISIELYNEHAPKTCKNFVTLAQRGYYDNVIFHRIIPDFMIQGGDPTGTGRGGSSIYGEKFEDEIKPTLKHTGAGILSMANSGPNTNGSQFFITLAPTPWLDGKHTIFGRVKGGMKLVQRMGLVKTNTEDRPLDEVKIVKASVVEKEND

>CF317_000756-T1 CF317_000756

MSDTRCRRCHRKKPARHAYTEHSYERARPLPSRQSDEPPPPPPPPLPPATSHQHVVPSAISTVPPRPPAPTAPPPVFHYPQTPTSAQAQSIVNTTYHIKQFSADFRQPKYEKALDILRSLQFLCQNIMTKHNLRVDHLWELDPSHHGVNGCNVGQGRQIMVRLRNETNSGFRSMEEIMDTMLHELAHNHYPEHDRHFYSFWNTLRGEYEGFHASDYAHQESVRGISFFKSRPQYRKLPSRSAFPPKSCRVGPFPPSITTTTSDVRSYLPAVEPPSAIAGGPSYALGQVYQTVAGPYPQSVRPPLGQATGFSSTPFLSTTSPYLYAPHSAVPNSVEPTFFDPRNHFAGRRG

>CF317_000757-T1 CF317_000757

MARKLLSSSAKKLCSSYKGQSRQYSAPIDSSIPGSKLKFVPTSGTYPKGFKVSATHVGVKPSNTKNADLAFITSDAPAFGAAVFTTNRFQAAPVQVSRKILETRGGAGIRSIIINSGCANAVTGQGGLEDAQAMSDTASSMFQQKDQPEDAQGSNSLVMSTGVIGQRLPIKKILSKISTAYKHLGDDHNAWLSTAKAICTTDTFPKLLSTSFRLPGKHHDGIEYRLAGMTKGAGMIHPNMATLLGVLCTDAPISQRALKPLLVEATKKSFNAISIDGDTSTNDTLAILANGAAASSMDTPPIEPGTEDYKAMQAVLTDFATKLSQLVVRDGEGATKFVEVRVRNALTENDAKLIASTIARSPLVKCALYGKDANWGRILCAIGYTQGLSSPDSVVPASTNVSFMPAENSATKEEGVLKLLVDGEPEAVDEERAARLLEDEDLIIDVNLGTGKEEASYWFCDFSHEYVTINGDYRT

>CF317_000758-T1 CF317_000758

MADQNAVLSQILSALQKLETNQTALAATVEAMQSKIDTSSSVSQLRTQHVPRPTDSPSVDPKDVNGHSSSPPTRSSTDGHRSSIDLGSPAKATSTDRRPSITSRIILTTYPGQSGIDPIPMSWGEADPAKRGPVVVSRHQKTIRRRNAIGAHGGSYAIYNALAVASHHLDLEHKPDFTNTEPAATIGPFPAWGDKKKIVAMDPYGHLAPWLYKDLMQSQDLDIRPTIAVTKAHMKIPELEQSVMNGRLVPDGKYCLNESGELAVTKFAVEPVWYLPGVAERFGITEADLRRALFEHTGGSYPELITRNDIKVFMPPIGGLTVYCFGDPAKMSDPNVKLALRVHDECNGSDVFGSDICTCRPYLIFGIEEAVKMAQQGGSGVVIYFRKEGRALGEVTKYLVYNARKRGSDRASEYFKRTENIAGVKDMRFQALMPDILHWLGITKIDRMMSMSNMKHDAIVEQGIPIHERVEIPPEMVPEDSRVEIDAKIHAGYFTNGKVMTVEELNAVQGRAWEDIEH

>CF317_000759-T1 CF317_000759

MGKRKKSSAKPAGPKKRGPLDTQFTCLFCNHEKAVQVKLDKKAGIGDLYCKVCGQKFQSNINYLSAAVDVYSDWIDACESVAQDAVAQEKEATAEDKDFSNYATERPKATAAGANDDEDGGYDEDDY

>CF317_000760-T1 CF317_000760

MPTRRSEPIHFGFRFGAQEGIGFRNPFKRKPSLHPGPPGISKYTDGDDSITRTICSGYAPSRTGRTTVLCDERAPLLGDTWTPSDIAPGTMVICDGPPQYAEPDGCIKRPSSSFSHDRLTSDLASSERPSSIASSTQSPHSHIESWATDVRARSTKSPRLRAQSAVSFPSGRSATADDVSSSREALKKSRGDSGYRSPFVS

>CF317_000761-T1 CF317_000761

MSDKKRKASSAPVARPQKKQQTTSAATVEHVPSPDILKPVIASTPGASFPEVRFQSFSRKTQDGIQMLIHSSDHPTIDYTAAEGTEPNEAHIKHYVAVFDPATNKLKVTEAKRLTVRGALRQRPKLDESDDEDTQNFLALQGTRSALTEAFGSKKSKKAVQAIAENRQLGQGLEGATIANAMASQIADDEDEEEPTTATAAARSNKPLPQPNLRTDDIEEVYSLSALVQPSPASNTLRNMPIEQWRARMRANKEVIGLRSRYVANRVTYIGKQVLESHDSRYVQQLQLLRYIELLIEIAQFAGKQDRRRRMKFVDEWPEHTLTQGVPPAIVKETVNQFFPDNTPSDRAMTLLRTSIFALTLHILPPSGKTGGSQLVAELTDIQLDLAMEAPEVRKLYHELGCKVAPATDRDLTSWGYTKLVDKKKRKTEDGTTLPKPQFAVLKFPLNFPRASGQRRTKR

>CF317_000762-T1 CF317_000762

MTSRAETETTERDDLGRLRRRRQTKTRGRVSFPLFNAWQARLRTAHKTADTSPATLPFLLLKSLPHIFTQSSTSSEDESQVEKEESPKTQAKEAQDESQI

>CF317_000763-T1 CF317_000763

MSQTPSQACCNTPAVVSKGYKEKGSFTTVDGLKTYTTGPKSTTGILVVYDIFGFFPQTLQGADILAGDENSDSSLKHQIFMPDFFEGKPADISWYPPDTDEKGQKLGEFFKTTAAPPKTVERLGKVMKELKDKNPDIKNWGVLGYCWGGKIVNLVSQQNTVFKAAAACHPAMVDANDAPEVTIPIMMLPSKDESKDDVNKYQQNLKVKNQVEWFDNQVHGFMAARGDLEDESVKKAYEKGYSLLTKWFAENLSSDSSKL

>CF317_000764-T1 CF317_000764

MKVIEFTPTAQNVTHLPDEVLDMVISFLRWSRSAKAAQRDMWACCLVSRDWYASAVKYLYQAPVLSPRNFAEFARTLSPPVASRSRRVGLEDFVQHLDMGMLAYESKKSMTSRLISRTKHSLRTFVGPAVSFSTTSLAPLSKCGQLQQLDLSRDDYDFDLSQLMRSIKSLTSLLWLNLSKNCLTFHYERNYDLMAQQKAGFWPPNLTFLQLNESHLHTTVGLWNIFLTSLPACLESLSFRNCADYDPFDTIARVEARLPQITTLSISVHRSDDTYYFNHLTEPFPNVTKVTVPAMTSWLLKNFLFMSNDTSWSVMTDNTSIPRPPRNLEVLELEESADWPSANHIRIPDLKQFVELCPQLLHIDVPETYLNVDDDEDENDMDLDDLNEILVKRAAEMAKDKISSREVPMHQVGIFATERAQNAGVGMKRSFRYREG

>CF317_000765-T1 CF317_000765

MAAPQRRKRPREEAFETPTVAQLLTPRVAPGTPLTVKSISARLWQALRGHGKDAYYVTQFLRGMPWEDTKEEQEEWKVWRFLGAGGHGGAAVWIKNDEEGHLLDEVVLKEQRNPRPQFCYDYRAHIPSEAVLQNLVNVKQGCENIVHLRDFKTHFRQEPKHEVWRFYLEYAPHGSLENLRVRYRAFHRYMPEHFLWHVFNSLAKAITTLHDVPYDKEYIIHADIKPDNVFLGYEELHDQFKPTGGLQSGEYPTIKLGDFGCARETYDEESVDDNFRRFLKVGTRGFHPPEQPLEMRDDQWGYGHDFNPPVNDEKPRMTEKANVWQVGKVMYDLYSLSDQERYDDLKAKSGHEYHVVNRSDTLLDWMTTPFVDDLHRQRSPYSPTLGQLISRCMAANSADRPTPDQLYNETLQCLRSSSQHSQSVDPVSVRNATLYYRGNEINNMAIDEMNCYHHLPLWDYFRLLQPANNDPSEPRLRIYPGQTRDTLVGLDDHEFRLALAYNVSLEENVQAYRWRKHEVKQDRIRRHDDRILFEDIQSDEPPTSDGASSSSFDTDDDDYNEDFDDPSKKDMERRWLDAIANRQQHKRNKQQRPLNEQRRQSNGQQRRANGQQNQANELHRDMSYSRAAAGIREGMPPAKDMTPMRHIEEMEEAMLFPTRPITDQLSTVPLTGLEDPKYVSRRTLNALQRLGFPLEPSRPRPVATSSALRPSQARDNVQPEAGSYIVRETNVPQRPENEPIPSIETTYHDERLTTQAGLVDVGAVETISSGATSDRAPSVTRIRTPSEEPRTSWVLRMLSEQASTGETPEAYHQNLQAQTRGRNMVPQDPVSHSRSRSIRPEDGQAADLRQSAGQTLATQHGGHSAPTLQSISVASGLSASTSTPIDGDRGRGRSPGRGRGLGRGRGPGRGRGRGVGRGQADNAAPRGRTGGQSTASRATARVKRATTAAAANTRNAGHQTRERIVDEQAAARAARAARRALR

>CF317_000766-T1 CF317_000766

MGFTTGFLGGLTLTYSLAYLSVYVHRSNRTYQSLLLRQQAQLLNSKVDPPETEYEPPAYRIEKAGLEEQMKDRWNREVEGLVRTAQTTDWEAVRIRWENRIKSAFDSLRSTEKAQELEQKFQENVVDPLSNPQLLKESIKEAVGGKRILEENVGGTESNPQLVKDSIREAVGGKRILDEK

>CF317_000767-T1 CF317_000767

MTEFDALPQDILLLILQHFEACDLRCLQLVSRSFGATFSEPVYLRTILKGYSYAREVRRLLQEDYQALSSSQSRLQEHDHDPINLQTTFSTIATRYYHLAQGRSRSIQRINLRALDQSGHWLPTPQWDYHESQPGGRLYHEHASHLRGRSVPGISKPFLFRPTLWSYDDGLIVFAPAQTEEQHGWTDRATSRVGTEQKSGKLQDRCLVVLDLESNAQTEIPFDVHGKIIRNVRLKEGVLIVEWAEKDAFHDLNMVDRVHRHFATAFRITRTRTNDNPQPADTFPPHETSTTHPQSTLKLDVHFHSEWRIHFLGFPLTSRDYFFSTHTASHYCLYYWQPNRSLWTGDEDQPIEALQVWDISQACPYRPSEDPGNVHRDKFGKDGPNMVAKFAMSMLGFLGIRQQGRISLLGLGLDSQMGTVSWRENVFESGQGYFDPAERNWCATVTAFPFVGMGPVHRREGHVELPSYRGHCSMESDEIEEIEKWFLPIMDVVDRSADVRFNLIETCFTGMMAENRVVLRLKVRDEWTTVIDELAKEVGAMGRIAGDERWVIGQNERLQVVVARFQ

>CF317_000768-T1 CF317_000768

MTINDNGTASSIAIPTSSSNTATTTNGNNSFTVKAGLAQMLKGGVIMDVVNAEQARIAEEAGACAVMALERVPATFERTGIQEAVTIPVMAKARIGHFVECQVLEALGVDYIDESEVLTPADDTYHVEKNNFGVPFVCGCRNLGEALRRIAEGAAMIRTKGEAGTGDVVEAVKHMRTVNRDIARAQGVLATEGEVGIRALARELGTDAALLKQTAELGRLPVVNFAAGGVATPADAALMMQLGCDGVFVGSGIFKSGNAAARAKAIVTAVTHYKDAKIIAECSAGLGEAMVGINCGSMAPGEKLAGRGW

>CF317_000769-T1 CF317_000769

MSKNTDVSSTSRKARRNLRRASRIREAKAQAVAKLQKPGSNTDQGTQKLSFFDLAPEIRNEVYEQLFPPTTKFTVRSSSFLSTRRLWRPSKKTIFSVLRLSQQIRYEAAGIVAKNSHCHLIIDAAFDLSSLPAWLRGSVRTFEVEGFLLRGLPPQLPAYVLGFSNVERIIAATYMVNRELAYNTSQNIPETDTVAITQSYFDSIRSRKGKPLRMQDLEGNLMESVPERVRKITGPAASWPADRKYPTIEITVYFVNKWSSTGYYSAGMVATYDVATRTVTSASTLWLFRENPIMDLSEMTSPDTPDTDHRRGERDPYGKRKRTMVLNYNWSNPKGWLYFSQDQYEEFLRRRKWMREYLGLD

>CF317_000770-T1 CF317_000770

MALQASNAGAENGASEFEMRRAEIVGQIGDSLEQVLTQINALNRSLEGIIEIGNEFAQVEALWSQFENVMGNGQAAAEEEEGRNQDQHRPGENGES

>CF317_000771-T1 CF317_000771

MATQTQSHSQQFQSSIMSEKAASSPSAPLSPRKSTRAPSTRTSNATASTVNSSARMSEQTNITQPPAYSKKFVVVGDGGCGKTCLLISYSQGYFPEKYVPTVFENYITQVPHAATGKTVELALWDTAGQEEYDRLRPLSYPETDLLFVCFAIDCPNSLENVMDKWYPEVLHFCPTTPLILVGLKSDLRTKRACIELLRTQGLTPVTPEQGQAVAKQMGARYLECSAKEQKGVTEVFETAINTAVQVEETSYETKPSAPGKKTKKAKKRSCKIL

>CF317_000772-T1 CF317_000772

MEARNGKIDAGTAGLDHYKSRLPPWRYVVREKLLPITRAETPYVALLQERMRSPALDTYFAMTANLGTHTCFMLVLPILFWCGNPFLGRAMTISLAAGVYLSGFIKDLLCLPRPLSPPLTRITMSGSAALEYGFPSSHSTNAVTVAVFSVLLLRNPSTTISADMSMYLQVSAYLYAFSIVLGRVYCGMHGFFDVVCGSLLGAAIAFGQFYLGPVKDDWMFSSAGQAVVVIALVICILIRIHPEPADDCPCFDDSVAFTSVVLGVDIGGWHFARTHYADASATYPGTVPFSISGLGWPKIIARILLGVLVVFVWRAIMKPLLLGGLPPIFRMVENLGLDLPRRYFKPASQYKTVPDQRDDDNVIPSARDIPHLLTNLRRRRAETVGPQSEADAYEALAYRQKRRRDSLLSPGPAAPPASATGVAEAGQDYFELSKAEGPRSRKRSLNLEEFRAQMGASADMMSPEAVATPHQAQDNASMRMNTDDEKDRSELFATVQKPRVRYDVEVITKLIVYCGIGWLAVEGNPILFHHVGLSI

>CF317_000773-T1 CF317_000773

MANSIHLYRDDPNQMQFLWTSDDEDERKPSPEPTPAREEQPAQESNTKVEDTSHTTLDPPKEPTPPPSDSDPDWDPASEADPEEALKWRRFLRAQPESLDERGFNMPIHGVISPETQEWNEQHGKRRELQRAAEPQTPSRPPPTPLLHSTPAQFPGTRTLESAEAVSSPLKSFWNGLVSIFSPRDERQQPQKETRPAVKEESPVSTPIEGVSKENKSISGVAAIDDGTTITDTPSSPTSSEDGTPSLEPATQTESSTNSSTPATSIKAEESTEVKPAEKEPNPFETPAKQLASLTLRDTRTPLQHSRTPKASFHQSVKTDRKTRAQKLLDEREARKDAYKLVPLSKEWDAKVRHAVQHGVKDNEGYTKYNDTDLARVVPQYARGGVHDNWLNDAVVNDYVALCVKHGNKDDRPTQVPTFAAFSSQTWQKIQSDPKSIPTRWWKRQGIQGKRALECDTIFMPINTGSHWTLAVIHPKLKNITMYNSMGYGRNQAVAEIILMFMKQEVGASLLEEEWTINARGLSPQQRNSDDCGVFSITTARQVVLGKMDKEPYSADVIPVQRKRIVAELVNEGLLSTEASAGDASVKGK

>CF317_000774-T1 CF317_000774

MEQLIRDLNSPQNQGRPHVINTIQRQIQALQREPTAWQAALNLLNSDDHILQFYGALTLEQKVNADWETDSIGENRDHVSQLLRQLVTRYVTIATSTESEVVLSKLSSALAAIFGAAMVEDEVCQAALDIMATIAEGFSDWSEPSQFDESMTALIQDVCLATLVKVRYPPEELDKTTAIWDEDDNTRFEDFRFSANDFFQTAFGILGPPLIEDIARSVSDASESNWAEFEAAIWVLGSVSDALSNEPERCDPSLNKIFSSSLWHGAISASEGTPGRVRKSIITLLAETTSYLQRNSQHLIGSLDFLFRSLQIRAHSNHAARAIYSLCDSQRSFLVQALPQFLQTLTTLEDIPLHSGCKVLSAVSALVQALPSEADKIEPLQKMLQLVQNLEAAHATRASLTQDDESQCPLLERLSMLAAVARGLQGAAETPVDLEATKSTATTFWADGAGRQTQQVVLQMLAEPWSRLLEPDQADLVSAACELLKAGFKEDHPTPFKFPLHVSTELLCTLIDTRNANLDQTMNTASCYVSSEPAPTPETSPQAEALISRVLTLTKESTERLTDPATSNTFSAPTCILDFVIRSLPKYGTHILTHPSAIDIMSAFIDYALLLLRSTTDTLPRRSAAAFFTSFLELTDPSSTLMSNPNPAVAANINALLTRSSPPTLALTLHLLSGECARSEIDSLTLLLRTYISKQPLRAKPILTAAMRPESGVLTPKAMSATKTEQRARFVAQIESLRGARKTNDVVRDFWVSCRGGQFGYVT

>CF317_000775-T1 CF317_000775

METVDTSKRLSQLRDLMKEHKVDVYIVPSEDSHQSEYIAPCDARREHISGFSGSAGTAVITLEKAALATDGRYFNQASKQLDSNWLLLKQGLEDVPTWQEWTTEQAEGGKTVGVDPTVITAADARKLTKSLKKTDAKLVGVNKNLVDKIWTDRPARPAEPVIVLSDEYAGKDFKEKLTDLRKELDKKKTAGMVVSMLDEVAWLFNLRGSDIPYNPVFFSYAAVTHDSATLYVDSSQLDDKVYEHLHGTVQIKPYDALFSDLNVLSQGPGQNGADTKPNKHSKILVSNKASWALSLGLGGEEKVEELRSPIADAKAIKNSTELEGMRQCHIRDGAALIEFFAWLEDQLLTKKATLDEVQAADKLEAIRSKGKHFKGLSFDTISSTGPNAAVIHYKPEPGNCSTIDPNAVYLCDSGAQYLDGTTDTTRTLHFGQPTEMEIKAYTLVLKGVIALDRAVFPKGTTGFAIDVLARQALWREGLDYRHGTGHGVGSFLNVHEGPIGIGTRSAYSEVSLNIGNVISDEPGYYEDGNFGIRIENMIMAKEAKTNHKFGDKPWLAFEHVTMVPMCRKLIDVSILSPEEKQWLNEYHQEVWDNTHGYFKDDLTLTWLKRETGRIG

>CF317_000776-T1 CF317_000776

MPLNQLVNFLTERSPQHAYSEKLIDRISEIVKSTWYLGFTSFGGPAVHFQIFHKKFVEEKEGRTPWIDEQTYQQLFALCQGLPGPASTKFFFAIALVHAGWIPAILAFLIWSIPGAVGMFALALGVRQISDTLPEPGYAFLSGLNASTVGIVAVAAVQLAEKCIKDPLSRLLVIFGACAGLCYGALWYYPVLVVAGGSACVTWDLWLGRRARKLKQAWKARRSHAADQAAAAEDGAAGNPPRQQDIELRVPEAAASTKHNTGLVQRRQIQPEPTADRSTEATAESSTESATTTDVQQQRRQHPQSDSEPFRVPYGVSVRTGLTVLAIFTVSFIIILVTRSELPQDHRPLSLFANMYLAGTIIFGGGPVVIPLLREYVVSPGWVSPRDFLIGLAIIQAFPGPNFNFAVYLGALAVAGSVAGISAGHALLGAVLGFIGIFLPGLVFAIFSQGLWRILRTQPLFLALLRGINATAVGLVFTAVYRLWEVGYLTSESTSGDNVDTISGGRVSGRSLADEPWFVVIAVLTYSGNKWFNIPAAVGIAAGGILGLAWWGVTQQ

>CF317_000777-T1 CF317_000777
[truncated: 4,684,349 more chars]
